# Bridging the pyridine-pyridazine synthesis gap by skeletal editing

**DOI:** 10.1126/science.adx4762

**Published:** 2025-07-17

**Authors:** Mikus Puriņš, Hikaru Nakahara, Mark D. Levin

**Affiliations:** Department of Chemistry, University of Chicago, Chicago, IL, USA.

## Abstract

Pairs of heterocycles differing by a single constitutive ring atom can exhibit stark differences in the retrosynthetic disconnections available for their preparation. Such a synthesis gap is exemplified by pyridine and pyridazine. Pyridine (a six-membered C_5_N ring) has risen to prominence in discovery chemistry, its ease of assembly spurring further synthetic development. Despite a host of favorable properties, pyridazine (an analogous C_4_N_2_ ring) has comparatively lagged behind—a discrepancy attributable to its often-challenging preparation, which arises from an electronically dissonant heteroatom arrangement. In this work, we achieve a single-atom skeletal edit that produces pyridazines from pyridines by direct carbon-to-nitrogen atom replacement: Azide introduction at the ortho position enables a photoinitiated rearrangement of *N*-amino-2-azidopyridinium cations. This transformation links the two heterocycles such that the richness of pyridine retrosynthesis becomes available to pyridazines.

## Editor’s summary

Six-membered rings with five carbons and one nitrogen are very common motifs in pharmaceuticals. By contrast, analogs with two adjacent nitrogens and four carbons are seen much less often in drug research, notwithstanding their appealing properties. The discrepancy likely stems from the comparative synthetic challenge of incorporating the extra nitrogen. Two groups now report complementary approaches to conveniently access these compounds. Puri#š *et al*. used photochemistry to slice into six-membered pyridine, add in a nitrogen, and expel the carbon. Li *et al*. started with five-membered pyrrolidine and introduced the nitrogen through ring expansion. —Jake S. Yeston

Functional molecules, including medicines, are optimized through iterative synthesis campaigns (1), wherein subtle changes to the lead structure, such as skeletal C-to-N replacement (2), can markedly affect the physiochemical properties of a drug candidate. However, such campaigns do not sample chemical space (3) uniformly in every direction because each new structure requires a new, often disparate synthesis. Chemists will naturally be biased toward compound families with well-established syntheses and functionalization protocols (Fig. 1A); other, less-studied compound classes consequently are at risk of being overlooked owing to this synthesis gap, which could result in missed opportunities during lead optimization (4). Skeletal editing reactions (5) offer an opportunity to address synthesis gaps by leveraging well-trodden synthetic routes of easily assembled compounds with an appropriate modification to provide access to orphaned core structures. By linking the different scaffolds retrosynthetically with a single chemical transformation, syntheses of the former become applicable to the latter scaffold. This study discusses such a transformation—linking privileged pyridines to underutilized pyridazines.

Pyridazine is a six-membered aromatic heterocycle with two adjacent nitrogen atoms that lead to specific properties (6–9). Chemically, it will exhibit similar hydrogen-bonding affinity to that of pyridine (p*K*_BHX_, 1.7 versus 1.9), but its lower basicity (p*K*_BH_, 2.0 versus 5.2) extends this capacity to more acidic environments. In addition, the pyridazine ring has substantially decreased lipophilicity (cLogP, −0.51 versus 0.84) and increased dipole [μ(D), 4.2 versus 2.2], which imparts increased water solubility to pyridazine-containing drugs. It is therefore surprising that although pyridine is the most popular heterocycle in Food and Drug Administration (FDA)–approved drugs in the past decade (10, 11), pyridazine is present in only six such therapeutics (Fig. 1B). Some recent examples of approved medicines containing pyridazines include glutaminase ALK/MET inhibitor ensartinib (approved in 2024) (12) and TYK2 inhibitor deucravacitinib (approved in 2022) (13). On the basis of the above analysis, we posit that this discrepancy between the two heterocycles is due to a synthesis gap. Whereas pyridine syntheses have been the subject of several books (14–16), pyridazine synthesis is far less well developed. Pyridine can be traced to consonant (17) starting materials, such as 1,3- or 1,5-dicarbonyl compounds. By contrast, pyridazines are usually synthesized from dissonant starting materials, such as 1,4-dicarbonyls/hydrazine (18) or 1,2,4,5-tetrazines (19). In the retrosynthetic sense, atom transmutation can flip the polarity of one of the nitrogen atoms and thus is the most simplifying skeletal edit. Considering the wide availability of pyridines, a synthetic method that achieves the direct pyridine C(2)-to-N replacement would facilitate the evaluation of pyridazines in discovery chemistry settings.

The toolbox of available C-to-N transmutations is limited in ways that prevent application to the pyridine C(2) position. For example, quinoline C(2)-to-N(2) (20) and C(3)-to-N(3) (21) transmutation relies heavily on the photochemistry of quinoline *N*-oxides through benzoxazepine intermediates; the analogous photorearrangements of pyridine *N*-oxides do not afford isolable oxazepines owing to parasitic secondary photo-processes (22). The recently reported benzenoid to pyridine nitrene internalization (23) is not applicable to 2-azidopyridines owing to the azide-tetrazole tautomerism, which necessitates the use of high-energy light for photochemical activation (24). Moreover, putative diazepines formed by this method would require a challenging N-N bond-forming C(2) deletion to form pyridazines. Pyridine-to-pyridazine reactivity has only previously been observed in trace amounts by oxidization of *N*-aminopyridines (25) or of tetraaryl substituted *N*-aminopyridones (26).

We were drawn to a study indicating that 2-azidopyridine *N*-oxides generate cyano-oxazines under irradiation with 350-nm light (Fig. 1D) (27, 28). The product is proposed to form through the formation of an acyclic nitrosodiene that is formed by concomitant N_2_ elimination and C-N cleavage (a heterocyclic aza-Zbiral fragmentation) (29). We postulated that by irradiation of the related 2-azido *N*-aminopyridines, the analogous cyano-diazine would be formed, perfectly positioned for electrocyclization followed by cyanide elimination to form the desired pyridazine. In this work, we report the successful implementation of this concept (Fig. 1C).

## Reaction development

The key 2-azido-*N*-aminopyridinium intermediates cannot be accessed by electrophilic amination of azidopyridines because the azide-tetrazole equilibrium (30) masks the pyridine while also introducing other potentially nucleophilic nitrogen sites. To circumvent these issues, we instead opted to perform an electrophilic amination directly on 2-chloropyridines **1**. 2-Halopyridines are notoriously weak nucleophiles (31–33), but the desired *N*-amination could be achieved with the use of our recently disclosed hydroxylamine reagent **2** (34), which serves as a highly reactive in situ generated source of ^+^NH_2_. The installation of the *N*-amino moiety activates the pyridine ring toward S_N_Ar, enabling reaction with sodium azide at room temperature (35). Both the 2-chloro-*N*-aminopyridiniums and 2-azido-*N*-aminopyridiniums are base sensitive, such that superior yields were observed in the presence of a buffering pyridinium *p*-toluenesulfonate (PPTS) additive. Finally, irradiation of the 2-azido-*N*-aminopyridines **3** with 390-nm light-emitting diodes (LEDs) delivers the corresponding pyridazines **4**, and this process is accelerated by the addition of benzophenone as a triplet sensitizer (vide infra). Critically, this whole protocol can be telescoped into a sequence that requires no purification of the intermediates.

Several aspects of the optimized procedure merit discussion from a safety perspective. First, the combination of PPTS buffer and sodium azide has the potential to produce toxic and explosive hydrazoic acid (though this is somewhat alleviated by pyridine’s higher basicity compared with azide) (36). Second, the C(2) is extruded in the form of cyanide, which requires effective ventilation and quenching procedures. Finally, both reagent **2** and intermediate **3** have the potential for exothermic decomposition, which limits the scale at which the reaction can be conducted. Nevertheless, by taking appropriate precautions, the reaction is practicable on the small scales typically used in discovery chemistry.

The reaction proceeds in synthetically useful yields when at least one electron-withdrawing group is present on the starting pyridine (Fig. 2A). For example, a pyridine bearing a trifluoromethyl group in the 4-position gives 4-trifluoromethyl pyridazine (**4a**), which was assayed by nuclear magnetic resonance (NMR) because of its volatility. Notably, the sequence could be also performed without solvent exchanges (see the supplementary materials) to give pyridazine **4a** with only a slight decrease in yield. Other electron-withdrawing groups, such as esters and amides, were also compatible to give products **4b** to **4e**. In addition, morpholine sulfonamide and substituted piperazinyl amide could also be placed in the 5- or 6-position to give **4f** and **4g**, respectively; however, substrates bearing an ester in the 3-position did not provide pyridazine upon irradiation (for other scope limitations, see fig. S2). Our subsequent investigations determined that this requirement for electron-withdrawing substituents could be offset by electron-donating substituents to a substantial extent. Alkyl substituents could be introduced at each remaining position, notably giving pyridazines bearing a 3-alkyl substituent (**4h**), an isoxazole (**4i**), a Weinreb amide (**4j**), and a functionalized benzyl ester (**4k**). 3,6-Disubstitution proved to be the most effective, giving the corresponding pyridazine **4l** in 66% yield. Further investigation of the 3,6-disusbtitution pattern revealed that phenyl, benzyl, and propanoate substituents as well as, notably, a carbamate-protected nitrogen were all compatible, affording products **4m** to **4p**. This latter amino pyridaz ine product is a particularly important substance class in medicinal chemistry (compare ensartinib and deucravacitinib, above). In addition, other di- and trisubstituted pyridazines **4q** to **4s** could be obtained, each requiring only a single electron-withdrawing group.

To explicitly demonstrate the bridging of the pyridine-pyridazine synthesis gap addressed by our method, we performed several classic pyridine syntheses, each of which represents a distinct ring disconnection pattern (Fig. 2B). These were followed by installation of the 2-chloro substituent and then the title method. For example, using Meldrum’s acid and ethyl acetoacetate, a [2+1+2+1] cyclization (37) could be used to access chloropyridine **1t**, which was converted to the trisubstituted pyridazine **4t** in 49% yield. Alternatively, Ru-catalyzed [2+2+2] of a bis-alkyne and ethyl cyanoformate provides pyridine **1u** bearing a fused ring (38), which, after atom replacement, gave the pyridazine **4u** in 57% yield. Pd-catalyzed [4+2] cycloaddition of an alkynoate and bromo-tert-butylimine (39) provided pyridine **1v**, which could be converted to tetrasubstituted pyridazine **4v** in 55% yield. Finally, we performed a [3+2+1] condensation of ethyl trifluoroacetoacetate, NH_4_OAc, and a substituted cinnamaldehyde (40) to afford fully substituted pyridine **1w**, which then could be converted into the tetrasubstituted pyridazine **4w** in 59% yield.

## Mechanism

Our mechanistic evidence is most consistent with a proposed sequence from 2-azido-*N*-aminopyridinium **3** to pyridazine **4** consisting of five key steps: (i) formation of a triplet nitrene, (ii) C-C bond cleavage forming a diazatriene concomitant with intersystem crossing back to the singlet surface, (iii) proton transfer, (iv) electrocyclization, and (v) cyanide extrusion (retro-Reissert reaction) (41). The intermediacy of triplet states is supported by observable luminescence of isolable intermediate **3a**, which is quenched by exposure to air. In addition, benzophenone, a known triplet sensitizer (42), accelerates the reaction, and comparison of the phosphorescence energies of the two species suggests that an energy-matched sensitization is possible. Alternative mechanisms of photocatalysis (electron transfer or hydrogen atom transfer) are unlikely given that the reaction proceeds in the absence of a photocatalyst, and the observed product distributions (side-products originating from reduction of the azide and oxidation of **IV**) are similar in the catalyzed and uncatalyzed processes (see the supplementary materials for details).

Computationally (Fig. 3), we have found that the ground-state surface has a prohibitively high barrier (**TS1**^+^) for N_2_ extrusion, which is substantially reduced in the triplet excited state (**TS1**^3^). However, subsequent ring opening of the triplet nitrene **I**^3^ is similarly prohibitive; by contrast, the corresponding singlet species (**I**^+^) is not bound, exhibiting barrierless ring opening. This indicates that the triplet nitrene may undergo inter-system crossing to the singlet surface to give ring-opened diazatriene intermediate **II**^+^. This cationic intermediate cannot energetically proceed with electrocyclization (**TS4**^+^); however, the electrocyclization is energetically accessible for the neutral species, through **TS4**. Thus, we posit that a proton transfer between the cationic species and a sulfonate anion (either from the aminating reagent or PPTS buffer) is necessary. Finally, a cyanide elimination provides the final pyridazine. This final step is experimentally supported by a cyanide trapping experiment, where treatment of the crude photolysate with *N*-benzylidene aniline provides an isolable Strecker adduct **5** (43).

## Synthetic applications

Several further demonstrations of the application of this protocol were undertaken (Fig. 4). First, we performed the pyridine C(2)-to-N reaction on a 1-mmol scale to provide pyridazine **4t** in 50% yield (Fig. 4A). A Boger-type nitrogen deletion reaction (44) afforded pyrrole **6** in 53% yield, resulting in a net C(2) deletion starting from pyridine **1t**. Next, we examined the title reaction’s use in a nitrogen scan. Previously, our group has reported the C-to-N conversion of benzene to pyridine (23), which, when combined with C-H azidation, enabled a prototypical direct nitrogen scan of a simple benzene substrate **7** to give pyridines **8** and **9** (Fig. 4B). This sequence was extended using AgF_2_-mediated pyridine fluorination chemistry (45), affording two separable isomeric 2-fluoropyridines **1x** and **1y**. Each of these was converted regiospecifically into the corresponding pyridazines **4x** and **4y** using our developed reaction conditions. This sequence highlights the synergy of our method both with advances in C-H functionalization and with other skeletal editing methods; continued developments in each will extend and improve the ability for such direct nitrogen-scan trees to be conducted. Next, we capitalized on the differing symmetry and selectivity of pyridine and pyridazine in C-H functionalization. By changing the order of functionalization and atom replacement steps, we can access multiple isomers of a disubstituted pyridazine selectively (Fig. 4C). Specifically, Minisci reactions proceed preferably at the 2-position for pyridines and at the 3-position for pyridazines (46). Therefore, starting from methyl isonicotinate, a Minisci reaction provides pyridine **10** (47), and 2-chlorination, followed by atom replacement, gives pyridazine **4z** with the added radical in the 6-position. Alternatively, by conducting the chlorination and atom replacement first, followed by Minisci alkylation, the alkyl radical is incorporated into the 5-position of pyridaz ine **11**. Finally, our method differs critically from classical hydrazine-based pyridazine syntheses in the differentiated origin of the two ring nitrogens. By using a labeled aminating reagent, preparation of nonsymmetrically ^15^N-monolabeled pyridazine ^15^**N(2)-4r** is possible (Fig. 4D). By contrast, an ^15^N-labeled starting material and nonlabeled reagent afford the other ^15^N-isotopomer ^15^**N(1)-4r** with full control of the selectivity. These results are notable because even if a selectively monolabeled hydrazine were prepared (48), control of regioselectivity in a condensation reaction (not to mention separation) would likely be impossible.

## Supplementary Material

Supplementary Text

Reproducibility Checklist

science.org/doi/10.1126/science.adx4762

Supplementary Text; Figs. S1 to S39; Tables S1 to S4; NMR Spectra; References (51–63); Reproducibility Checklist

## Figures and Tables

**Fig. 1.**
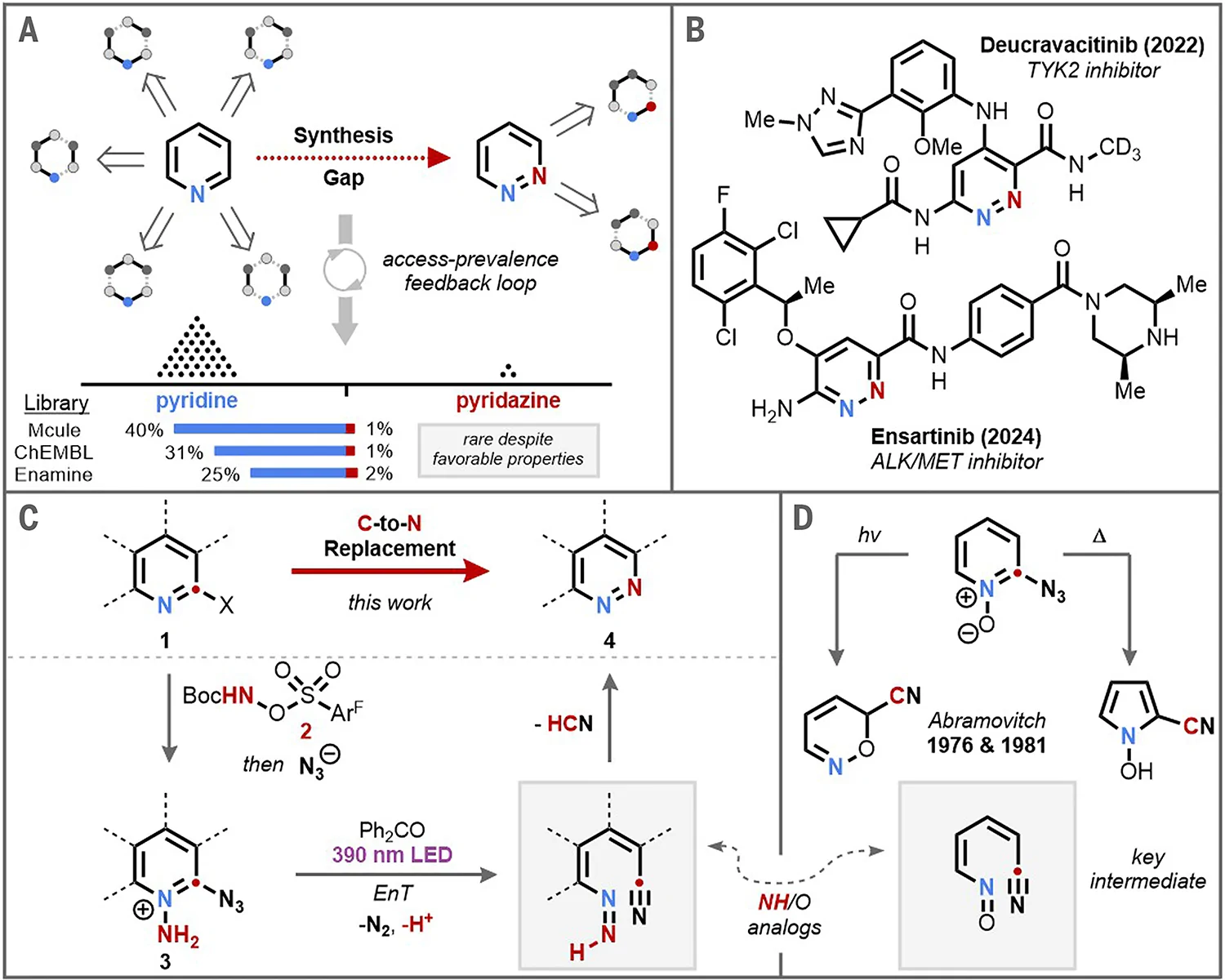
Rationale and reaction conception. (**A**) Synthesis gap between pyridine and pyridazine. Filled atoms represent nucleophilic centers, and faded atoms represent electrophilic centers. Library composition was reproduced from the literature (49). (**B**) Examples of recently approved pyridazine pharmaceuticals. (**C**) Pyridine C(2)-to-N atom replacement reported in this work. (**D**) Relevant precedents of thermal and photochemical rearrangements of 2-azidopyridine *N*-oxides. Ar^F^, 3,5-(CF_3_)_2_C_6_H_3_; Me, methyl; X, halogen; Boc, *tert*-butyloxycarbonyl; Ph, phenyl; EnT, energy transfer.

**Fig. 2.**
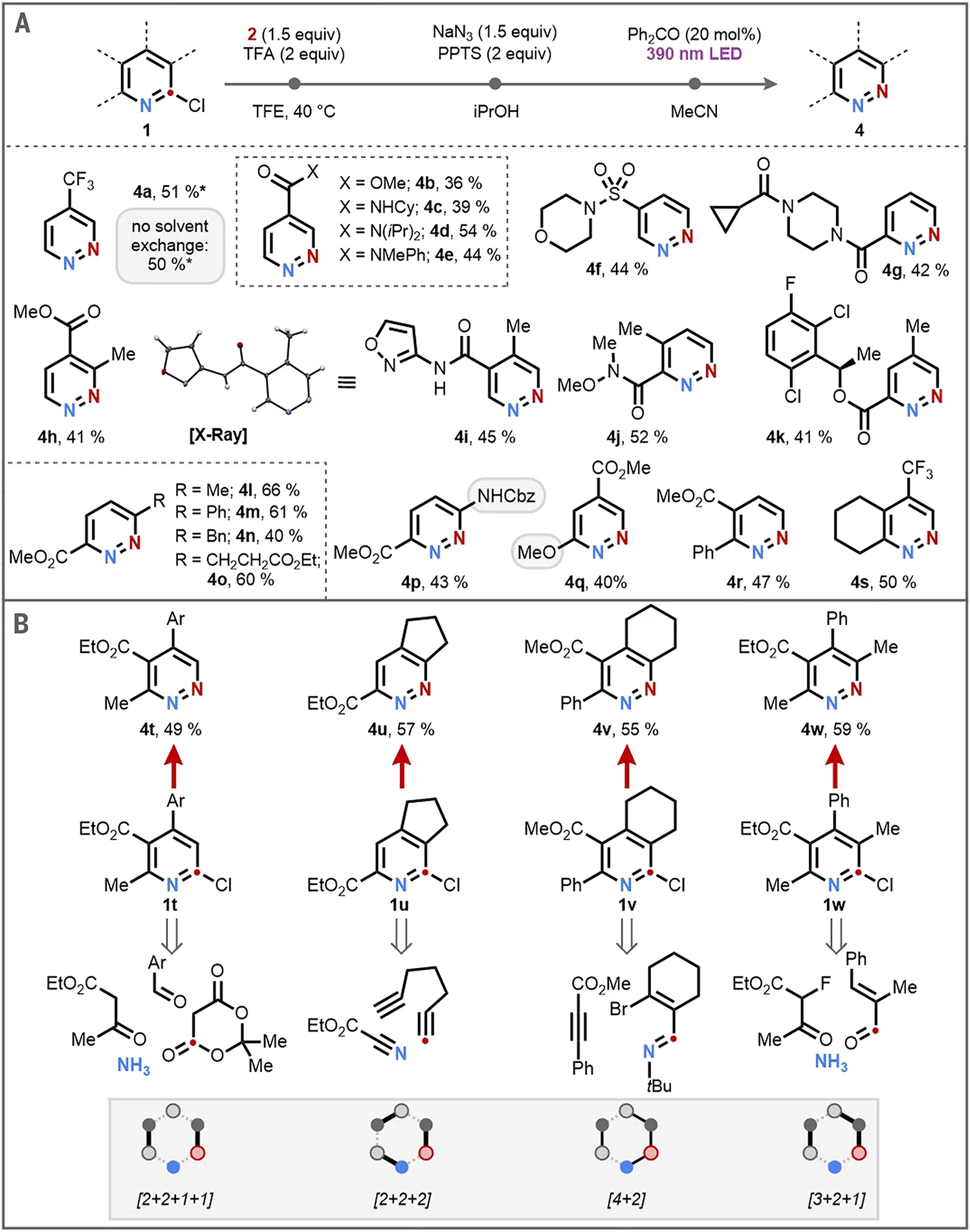
Scope addressing the synthesis gap. (**A**) Functional group and substitution pattern tolerance. (**B**) Examples of pyridine syntheses mapping onto pyridazines; shaded box below the precursors represents the disconnection pattern used in ring synthesis. Isolated yields unless otherwise noted, 0.3-mmol scale. Amination, 16 hours; azidation, 2 hours; photolysis, 20 hours; temperature, 22°C unless otherwise noted. See the supplementary materials for detailed conditions. *NMR yield. TFA, trifluoroacetic acid; TFE, 2,2,2-trifluoroethanol; PPTS, pyridinium *para*toluenesulfonate; Cy, cyclohexyl; Pr, propyl; Et, ethyl; Bn, benzyl; Cbz, benzyloxycarbonyl; Ar, 4-*tert*-butyl-C_6_H_4_.

**Fig. 3.**
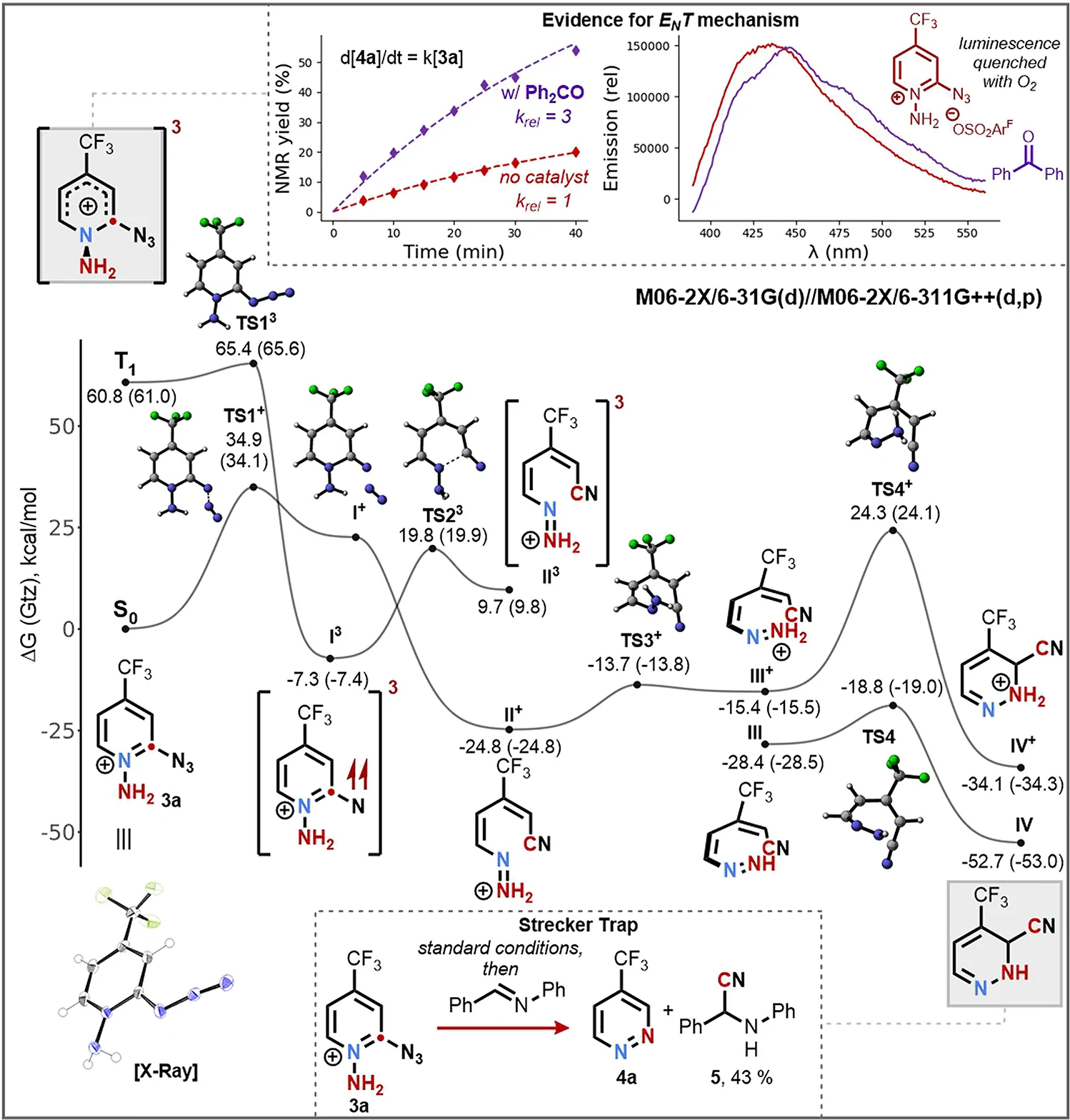
Computational and experimental mechanistic analysis. Kinetic data fit numerically to first-order kinetic model by least squares. Energy diagram was created using EveRplot (50).

**Fig. 4.**
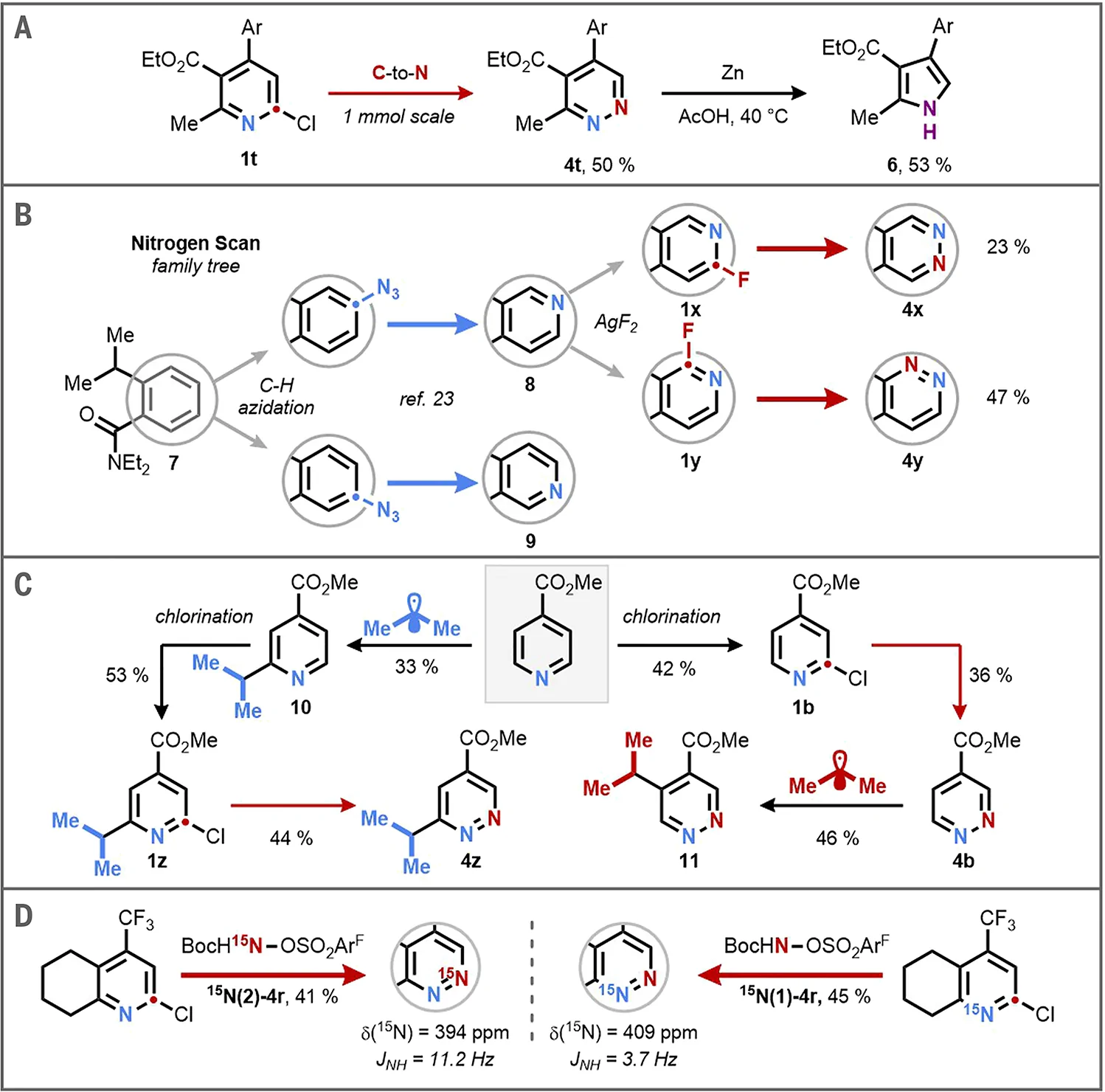
Applications. (**A**) A 1-mmol scale reaction and net formal C(2) deletion. (**B**) Nitrogen scan family tree. (**C**) Capitalizing on selectivity; chlorination conditions: (i) *meta*-chloroperoxybenzoic acid and (ii) POCl_3_. Minisci conditions: isobutyric acid, AgNO_3_, Selectfluor. (**D**) Selective ^15^N monolabeling.

## Data Availability

Crystallographic data are freely available from the Cambridge Crystallographic Data Centre under CCDC 2431452 and 2431454. All other data are provided in the supplementary materials.
