# Supplementary material for: Bridging the pyridine-pyridazine synthesis gap by skeletal editing
Source: Science. Author manuscript; Available in PMC 2026 Oct 26. (PMC13698566; doi:10.1126/science.adx4762)
Supplement: Supplementary file 1 [file NIHMS2215399-supplement-Reproducibility_Checklist.pdf]

Article Metadata

**Title**	Bridging the Pyridine-Pyridazine Synthesis Gap via Skeletal Editing
**Publication URL**	https://doi.org/10.1126/science.adx4762
**Data Availability Statement**	Data Availability Statement<br>All data are in the Supplementary Information or are available from the corresponding author upon reasonable request.<br>Crystallographic Data has been deposited to the CCDC (2431452 and 2431454)

Materials:

Newly created materials	Materials Availability Statement	Article Subsection	DataSeer Notes	Author Response
The manuscript includes a dedicated "materials availability statement" providing transparent disclosure about availability of newly created materials including details on how materials can be accessed and describing any restrictions on access.			Please include a Materials Availability Statement	

Antibodies

output type not detected in text

DNA and RNA Sequences

output type not detected in text

Cell Materials

output type not detected in text

Experimental Animals

output type not detected in text

Plants and Microbes

output type not detected in text

Human Research Participants

output type not detected in text

Design:

Study protocol

output type not detected in text

Laboratory protocol

output type not detected in text

Ethics

output type not detected in text

Analysis:

Data availability

**No Action Required**	*Done - Shared/Cited*

Research outputs are appropriately shared/cited						
**Datatype**	**Associated sentence from text**	**Article Subsection**	**URL**	**Accession/DOI/PID**	**DataSeer Notes**	**Author Response**
X-Ray Diffraction Data	X-Ray Crystallography: X-ray crystal structures were measured at 100 K on a Bruker D8 VENTURE diffractometer equipped with a microfocus Mo-target or Cu-target X-ray tube (λ = 0.71073 Å for Mo and λ = 1.5406 Å for Cu) and PHOTON 100 CMOS detector.	General Considerations		2431452	This dataset will be made public in CCDC upon acceptance.	
X-Ray Diffraction Data	X-Ray Crystallography: X-ray crystal structures were measured at 100 K on a Bruker D8 VENTURE diffractometer equipped with a microfocus Mo-target or Cu-target X-ray tube (λ = 0.71073 Å for Mo and λ = 1.5406 Å for Cu) and PHOTON 100 CMOS detector.	General Considerations		2431454	This dataset will be made public in CCDC upon acceptance.	

No Action Required	*Optional*					
Quality control and/or representative media are recommended but not required to be uploaded to a repository						
**Datatype**	**Associated sentence from text**	**Article Subsection**	**URL**	**Accession/DOI/PID**	**DataSeer Notes**	**Author Response**
Chromatography: Thin Layer Chromatography	Analytical thin-layer chromatography (TLC) was carried out using 0.2 mm commercial silica gel plates (silica gel 60, F254) and visualized by UV irradiation or staining as indicated.	General Considerations				
Chromatography: Flash Column Chromatography	Flash chromatography was accomplished using an automated system (monitoring at 254 nm and 280 nm) with silica cartridges (60 Å porosity, 20-40 μm).	General Considerations				
Mass Spectrometry	High resolution mass spectra were recorded on either an Agilent 6224 TOF High Resolution Accurate MS with electrospray ionization (ESI) or atmospheric pressure chemical ionization (APCI) All mass spectra were processed with Agilent MassHunter.	General Considerations				
Spectrometry: Biomolecular Nuclear Magnetic Reso	NMR spectra were recorded on a Bruker 600 MHz Avance Neo equipped with an Ascend 14.1T magnet and a Prodigy BBO, low fluorine probe, a Bruker Avance III HD 500 MHz equipped with an Ultrashield Plus 11.7 T magnet and a Prodigy BBO, low fluorine probe, a Bruker Avance 500 MHz equipped with an Ultrashield Plus 11.7 T magnet and a SmartProbe BBO probe, a Bruker Avance III HD 400 MHz, Nanobay equipped with an Ascend 9.4 T magnet and a SmartProbe BBO probe and a Bruker 500 MHz Avance II+ equipped with an Ultrashield 11.7 T magnet and a QNP probe.	General Considerations				

**Code availability**
output type not detected in text

**Links or citations for software**						
No Action Required	*Optional*					
Software objects are recommended but not required to be uploaded to a repository						
**Name or Type of Software**	**Associated sentence from text**	**Article Subsection**	**URL**	**RRID**	**DataSeer Notes**	**Author Response**
APEX4	The data reduction and integration were performed with the Bruker APEX4 software package (Bruker AXS, version 2017.3-0, 2018).	General Considerations				
SHELXL	The structures were solved by SHELXT and refined by a full-matrix least-squares procedure using SHELXL.	General Considerations				
OLEX2	X-ray crystal structures were rendered using OLEX2 software (1).	General Considerations				
Gaussian	Ground and transition state geometries were optimized with frequency calculations using density functional theory (DFT) in Gaussian16 (Revision A.03) (16) at the M06-2X/6-31g(d) level of theory.	Computational Methods				
Paton's GoodVibes Python package	Quasi-harmonic corrected free energies (Gtz) for each compound were computed using Grimme entropic corrections (18) and Head-Gordon enthalpic corrections (19) as implemented in Paton's GoodVibes python package.	Computational Methods				
Python	Quasi-harmonic corrected free energies (Gtz) for each compound were computed using Grimme entropic corrections (18) and Head-Gordon enthalpic corrections (19) as implemented in Paton's GoodVibes python package.	Computational Methods				

To Be Completed by Authors

Specify N/A for sections that are not applicable.

Design:

Experimental study design (statistics details)

For in vivo studies: State whether and how the following have been done	In vivo Study Information	Article Subsection	Author Response
Sample size determination	No in vivo data		
Randomisation	No in vivo data		
Blinding	No in vivo data		
Inclusion/exclusion criteria	No in vivo data		

Sample definition and in-laboratory replication	Sample Information	Article Subsection	Author Response
State number of times the experiment was replicated in laboratory.	A statement regarding the number of replicates has been added to the Figure 2 caption		
Define whether data describe technical or biological replicates.	N/A	N/A	N/A

Dual Use Research of Concern (DURC)	Authority and Reference Number	Article Subsection	Author Response
If study is subject to dual use research of concern regulations, state the authority granting approval and reference number for the regulatory approval.	N/A	N/A	N/A

Analysis:

Attrition	Article Subsection	Author Response
Describe whether exclusion criteria were preestablished. Report if sample or data points were omitted from analysis. If yes report if this was due to attrition or intentional exclusion and provide justification.	N/A	N/A

Statistics

Describe statistical tests used and justify choice of tests.

Statistical Test	Justification	Article Subsection	Author Response
N/A	N/A	N/A	N/A

Reporting

MDAR framework recommends adoption of discipline-specific guidelines, established and endorsed through community initiatives. Journals have their own policy about requiring specific guidelines and recommendations to complement MDAR.

Adherence to community standards	Guideline/Checklist	Article Subsection	Author Response
State if relevant guidelines (e.g., ICMJE, MIBBI, ARRIVE) have been followed, and whether a checklist (e.g., CONSORT, PRISMA, ARRIVE) is provided with the manuscript.	N/A	N/A	N/A
