# Supplementary material for: Bridging the pyridine-pyridazine synthesis gap by skeletal editing
Source: Science. Author manuscript; Available in PMC 2026 Oct 26. (PMC13698566; doi:10.1126/science.adx4762)
Supplement: Supplementary file 2 [file NIHMS2215399-supplement-Supplementary_Text.pdf]

Science
AAAS

Supplementary Materials for

Bridging the pyridine-pyridazine synthesis gap by skeletal editing

Mikus Puriņš, Hikaru Nakahara, Mark D. Levin

Corresponding author: Mark D. Levin, marklevin@uchicago.edu

Science **389**, 295 (2025)
DOI: 10.1126/science.adx4762

The PDF file includes:

Supplementary Text
Figs. S1 to S39
Tables S1 to S4
NMR Spectra
References

Other Supplementary Material for this manuscript includes the following:

Reproducibility Checklist

General Considerations

Unless noted otherwise, all reactions were performed in oven-dried or flame-dried glassware under an atmosphere of dry N_2. CH_3CN, THF, Et_2O, CH_2Cl_2, toluene, and Et_3N were dried by passing these previously degassed solvents through a PPT Solvent Purification System, and all other solvents were dried over molecular sieves (4 Å) and degassed prior to use or purchased anhydrous and sealed under N_2 (e.g. VWR Dri-solv or equivalent). Reaction temperatures were reported as the temperatures of the bath surrounding the flasks or vials. Sensitive reagents and solvents were transferred under nitrogen into a nitrogen-filled glovebox with standard techniques. Unless otherwise noted, all reagents were used as received. Analytical thin-layer chromatography (TLC) was carried out using 0.2 mm commercial silica gel plates (silica gel 60, F254) and visualized by UV irradiation or staining as indicated. Flash chromatography was accomplished using an automated system (monitoring at 254 nm and 280 nm) with silica cartridges (60 Å porosity, 20-40 μm). High resolution mass spectra were recorded on either an Agilent 6224 TOF High Resolution Accurate MS with electrospray ionization (ESI) or atmospheric pressure chemical ionization (APCI) All mass spectra were processed with Agilent MassHunter.

NMR spectra were recorded on a Bruker 600 MHz Avance Neo equipped with an Ascend 14.1T magnet and a Prodigy BBO, low fluorine probe, a Bruker Avance III HD 500 MHz equipped with an Ultrashield Plus 11.7 T magnet and a Prodigy BBO, low fluorine probe, a Bruker Avance 500 MHz equipped with an Ultrashield Plus 11.7 T magnet and a SmartProbe BBO probe, a Bruker Avance III HD 400 MHz, Nanobay equipped with an Ascend 9.4 T magnet and a SmartProbe BBO probe and a Bruker 500 MHz Avance II+ equipped with an Ultrashield 11.7 T magnet and a QNP probe. Chemical shifts are reported in parts per million (ppm) and the spectra are calibrated to the resonance resulting from incomplete deuteration of the solvent ($CDCl_3$: 7.26 ppm; CD_3OD: 3.31 ppm; CD_3CN: 1.94 ppm) Chemical shifts are reported in ppm with the solvent resonance as the internal standard ($^{13}CDCl_3$: 77.16 ppm; $^{13}CD_3OD$: 49.0; $^{13}CD_3CN$: 118.3 (nitrile signal)). Data are reported as follows: chemical shift δ, multiplicity (s = singlet, d = doublet, t = triplet, q = quartet, p = pentet, sext = sextet, sept = septet, br = broad, m = multiplet or combinations thereof (^{13}C and all other nuclides except ^{1}H are singlets unless otherwise stated), coupling constants J, number of nuclides (signals for all other nuclides except 1H refer to one nuclide unless otherwise stated), assignment. ^{1}H and ^{13}C NMR spectra are assigned as fully as possible, using $^{1}H^{13}C$-HSQC and $^{1}H^{13}C$-HMBC where appropriate to facilitate structural determination.

X-Ray Crystallography: X-ray crystal structures were measured at 100 K on a Bruker D8 VENTURE diffractometer equipped with a microfocus Mo-target or Cu-target X-ray tube (λ = 0.71073 Å for Mo and λ = 1.5406 Å for Cu) and PHOTON 100 CMOS detector. Data were collected using ϕ and ω scans. The data reduction and integration were performed with the Bruker APEX4 software package (Bruker AXS, version 2017.3-0, 2018). Data were scaled and corrected for absorption effects using the multi-scan procedure as implemented in SADABS. The structures were solved by SHELXT and refined by a full-matrix least-squares procedure using SHELXL. Crystallographic data and details of the data collection and structure refinement are listed in Table S3 - Table S4. X-ray crystal structures were rendered using OLEX2 software(*51*).

SAFETY NOTE: The aminating reagent shows decomposition at 60 °C. Safety tests and appropriate precautions are available with the original disclosure (*34*). Azides are potentially shock sensitive explosives, especially when concentrated. Appropriate precautions should be taken when working with azides. The procedures below also bear the potential to produce hydrazoic acid, a potent toxin and explosion hazard (49). The reaction produces highly toxic hydrogen cyanide. The following reactions should be conducted exclusively in a well-ventilated fume hood, with reaction scales minimized as appropriate, and conducted behind a blast shield.

Synthetic Procedures

General Procedure for the pyridine C(2)-to-N swap

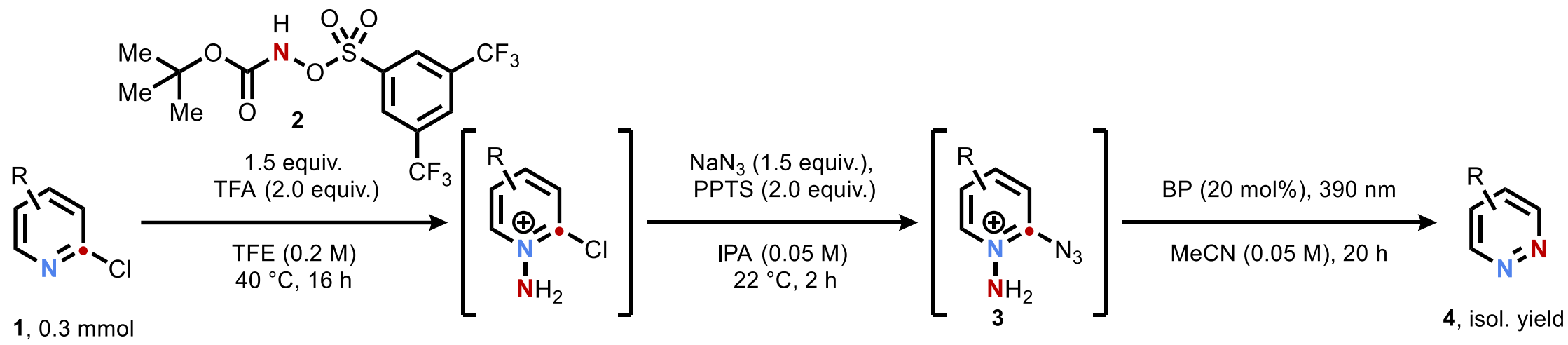

Figure S1. Pyridine skeletal editing.

A 8 mL screw-cap vial equipped with a Teflon coated stirring bar was charged with 2-halopyridine (0.3 mmol), *tert*-butyl (((3,5-bis(trifluoromethyl)phenyl)sulfonyl)oxy)carbamate and TFE (1.5 mL). Then, TFA (46 µL, 69 mg, 0.6 mmol, 2.0 equiv.) was added and the resulting mixture stirred for 16 hours at 40 °C. The reaction mixture was cooled down to room temperature then concentrated *in vacuo*. The vial was charged with PPTS (151 mg, 0.6 mmol, 2.0 equiv.), NaN_3 (29 mg, 0.45 mmol, 1.5 equiv.) and IPA (6.0 mL). The vial was then vigorously stirred for 2 h at 22 °C. The resulting suspension was filtered and concentrated *in vacuo*. The residue was dissolved in MeCN (2 mL) and again concentrated *in vacuo* to remove residual IPA. The residue was dissolved in MeCN (2.0 mL) and filtered through a 0.20 µm syringe filter, which was then washed with MeCN (2 × 2 mL). The filtrate was charged with benzophenone (10.9 mg, 60.0 µmol, 20 mol %), sparged with N_2 gas for 1 minute and then photolyzed with 390 nm Kessil LED lamp for 20 hours. Then, the mixture was vigorously sparged with N_2 gas for 5 minutes and then concentrated *in vacuo*. The crude mixture was purified by silica gel chromatography, usually followed by a preparative TLC.

Characterization of Products

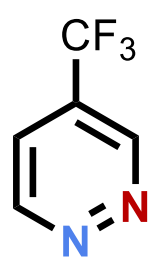

4a

4-(Trifluoromethyl)pyridazine (4a)

Prepared according to the general procedure using methyl 2-chloro-4-(trifluoromethyl)pyridine (**1a**, 54 mg, 0.30 mmol, 1.0 equiv.). The yield of pyridazine **4a** was determined by qNMR to be 51 % using mesitylene (14 µL, 0.1 mmol, 0.333 equiv.) as an internal standard. The identity of the product could be verified by comparing against an authentic standard after addition of Et_3N (42 µL, 0.3 mmol, 1.0 equiv.) to the crude mixture.

No solvent-swap protocol: A 8 mL screw-cap vial equipped with a Teflon coated stirring bar was charged with 2-chloro-4-(trifluoromethyl)pyridine (**1a**, 54 mg, 0.30 mmol, 1.0 equiv.), *tert*-butyl (((3,5-bis(trifluoromethyl)phenyl)sulfonyl)oxy)carbamate, PPTS (151 mg, 0.6 mmol, 2.0 equiv.) and TFE (1.5 mL). Then, the resulting mixture was stirred for 1 hour at 60 °C. The reaction mixture was cooled down to room temperature then MeCN (1.5 mL) was added, followed by tetra-butyl ammonium azide (128 mg, 0.45 mmol, 1.5 equiv.) dissolved in MeCN (3.0 mL). The resulting mixture was stirred for 1 hour at 22 °C. Then, benzophenone (28 mg, 0.15 mmol, 50 mol %) was added, the mixture sparged with N_2 gas for 1 minute and then photolyzed with 390 nm Kessil LED lamp for 1 hour. The yield of pyridazine **4a** was determined by qNMR to be 51 % using mesitylene (14 µL, 0.1 mmol, 0.333 equiv.) as an internal standard.

^{1}H NMR (500 MHz, $CDCl_3$) δ 9.58 – 9.32 (m, 2H, N=N-C*H*-CH and N=N-C*H*), 7.71 (dd, *J* = 5.4, 2.4 Hz, 1H, N=N-CH-C*H*).

^{19}F NMR (471 MHz, $CDCl_3$) δ -64.74.

^{13}C{^{1}H} NMR (126 MHz, $CDCl_3$) δ 151.7, 146.9 (q, $J_{C\text{-}F}$ = 3.3 Hz), 129.3 (q, $J_{C\text{-}F}$ = 35.5 Hz), 122.2 (q, $J_{C\text{-}F}$ = 3.8 Hz), 122.2 (q, $J_{C\text{-}F}$ = 273.6 Hz).

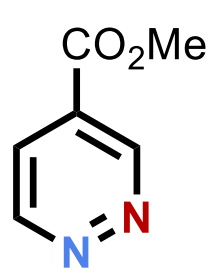

4b

Methyl pyridazine-4-carboxylate (4b)

Prepared according to the general procedure using methyl 2-chloroisonicotinate (**2b**, 51 mg, 0.30 mmol, 1.0 equiv.). The crude material was purified by flash column chromatography (SiO_2, EtOAc/Hex 20 to 100 %) and then preparative TLC (100% EtOAc) to give the corresponding pyridazine **4b** (13 mg, 0.10 mmol, 36 % yield) as a white-solid.

^{1}H NMR (400 MHz, $CDCl_3$) δ 9.66 (dd, *J* = 2.2, 1.3 Hz, 1H, N=N-C*H*-C(CO_2CH_3)), 9.42 (dd, *J* = 5.2, 1.3 Hz, 1H, N=N-C*H*-CH), 7.98 (dd, *J* = 5.2, 2.2 Hz, 1H, N=N-CH-C*H*), 4.01 (s, 3H, OCH_3).

^{13}C{^{1}H} NMR (101 MHz, $CDCl_3$) δ 164.3, 152.2, 149.8, 127.9, 125.6, 53.4.

HRMS (APCI/QTOF) m/z: [M + H]$^+$ Calculated for $C_6H_7N_2O_2^+$ 139.0502; Found 139.0502.

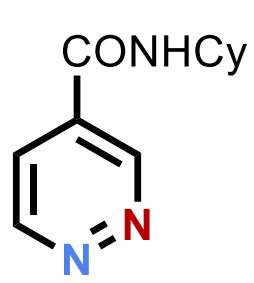

4c

N-Cyclohexylpyridazine-4-carboxamide (4c)

Prepared according to the general procedure using 2-chloro-*N*-cyclohexylisonicotinamide (**1c**, 72 mg, 0.30 mmol, 1.0 equiv.). The crude material was purified by flash column chromatography (SiO_2, EtOAc/Hex 20 to 100 %) and then preparative TLC (100% EtOAc) to give the corresponding pyridazine **4c** (24 mg, 0.11 mmol, 39 % yield) as a pale-yellow oil.

^{1}H NMR (400 MHz, $CDCl_3$) δ 9.47 (dd, *J* = 2.4, 1.3 Hz, 1H, N=N-C*H*), 9.32 (dd, *J* = 5.3, 1.3 Hz, 1H, N=N-C*H*-CH), 7.80 (dd, *J* = 5.3, 2.3 Hz, 1H, N=N-CH-C*H*), 6.45 (br. s, 1H, N*H*), 4.10 – 3.89 (m, 1H, C*H*-NH), 2.12 – 1.89 (m, 2H, cyhex), 1.81 – 1.74 (m, 2H, cyhex), 1 .71 – 1.62 (m, 1H, cyhex), 1.51 – 1.33 (m, 2H, cyhex), 1.32 – 1.14 (m, 3H, cyhex).

^{13}C{^{1}H} NMR (101 MHz, $CDCl_3$) δ 162.7, 152.0, 148.4, 132.4, 124.1, 49.6, 33.1, 25.5, 25.0.

HRMS (APCI/QTOF) m/z: [M + H]$^+$ Calculated for $C_{11}H_{16}N_3O^+$ 206.1288; Found 206.1290.

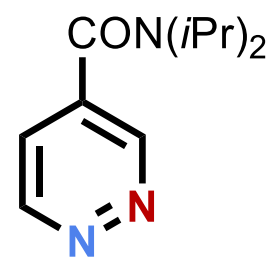
4d

***N*,*N*-Diisopropylpyridazine-4-carboxamide (4d)**

Prepared according to the general procedure using 2-chloro-*N*,*N*-diisopropylisonicotinamide (**1d**, 72 mg, 0.30 mmol, 1.0 equiv.). The crude material was purified by flash column chromatography (SiO_2, EtOAc/Hex 20 to 100 %) and then preparative TLC (100% EtOAc) to give the corresponding pyridazine **4d** (34 mg, 0.16 mmol, 54 % yield) as a pale-yellow oil.

^{1}H NMR (400 MHz, $CDCl_3$) δ 9.24 (dd, *J* = 5.2, 1.3 Hz, 1H, N=N-C*H*-CH), δ 9.11 (dd, *J* = 2.3, 1.3 Hz, 1H, N=N-C*H*-C(CON(CH(CH_3)$_2$)$_2$), 7.37 (dd, *J* = 5.2, 2.2 Hz, 1H. N=N-CH-C*H*), 3.58 (br. s, 2H, 2×CH(CH_3)$_2$), 1.51 (br. s, 6H, 2×C*H*$_3$), 1.17 (br. s, 6H, 2×C*H*$_3$).

^{13}C{^{1}H} NMR (101 MHz, $CDCl_3$) δ 165.5, 151.6, 148.1, 136.3, 122.7, 51.5, 46.7, 20.9, 20.6.

HRMS (APCI/QTOF) m/z: [M + H]$^+$ Calculated for $C_{11}H_{18}N_3O^+$ 208.1444; Found 208.1438.

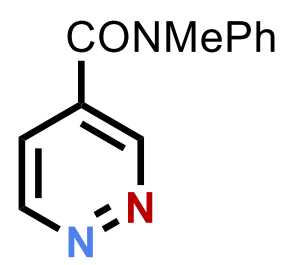
4e

***N*-Methyl-*N*-phenylpyridazine-4-carboxamide (4e)**

Prepared according to the general procedure using 2-chloro-*N*-methyl-*N*-phenylisonicotinamide (**1e**, 74 mg, 0.30 mmol, 1.0 equiv.). The crude material was purified by flash column chromatography (SiO_2, EtOAc/Hex 20 to 100 %) and then preparative TLC (100% EtOAc) to give the corresponding pyridazine **4e** (28 mg, 0.13 mmol, 44 % yield) as a pale-yellow oil.

^{1}H NMR (400 MHz, $CDCl_3$) δ 9.07 (dd, *J* = 5.3, 1.1 Hz, 1H, N=N-C*H*-CH), 8.98 (s, 1H, N=N-C*H*), 7.35 – 7.21 (m, 4H, ArH and N=N-CH-C*H*), 7.10 – 7.03 (m, 2H, Ar*H*), 3.52 (s, 3H. NC*H*$_3$).

^{13}C{^{1}H} NMR (101 MHz, $CDCl_3$) δ 165.7, 151.2, 149.7, 143.0, 134.1, 130.1, 128.3, 127.2, 125.1, 38.4.

HRMS (APCI/QTOF) m/z: [M + H]$^+$ Calculated for $C_{12}H_{12}N_3O^+$ 214.0975; Found 214.0965.

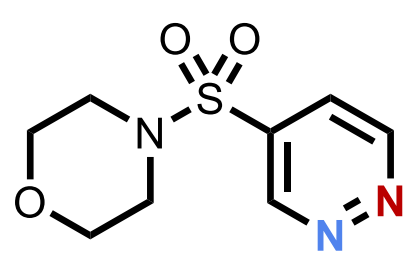
4f

4-(Pyridazin-4-ylsulfonyl)morpholine (4f)

Prepared according to the general procedure using 4-((6-chloropyridin-3-yl)sulfonyl)morpholine (**1f**, 79 mg, 0.30 mmol, 1.0 equiv.). The crude material was purified by flash column chromatography (SiO_2, EtOAc/Hex 20 to 100 %) and then preparative TLC (100% EtOAc) to give the corresponding pyridazine **4f** (30 mg, 0.13 mmol, 44 %) as a colorless oil.

^{1}H NMR (400 MHz, $CDCl_3$) δ 9.53 (dd, *J* = 5.3, 1.2 Hz, 1H, N=N-C*H*-CH), 9.47 (dd, *J* = 2.4, 1.2 Hz, 1H, N=N-C*H*), 7.75 (dd, *J* = 5.3, 2.3 Hz, 1H, N=N-CH-C*H*), 3.83 – 3.73 (m, 4H, 2×O-C*H*$_2$-CH$_2$-N), 3.18 – 3.07 (m, 4H, 2×O-CH$_2$-C*H*$_2$-N).

^{13}C{^{1}H} NMR (101 MHz, $CDCl_3$) δ 152.2, 147.4, 135.7, 123.6, 66.1, 45.9.

HRMS (APCI/QTOF) m/z: [M + H]$^+$ Calculated for $C_8H_{12}N_3O_3S^+$ 230.0594; Found 230.0595.

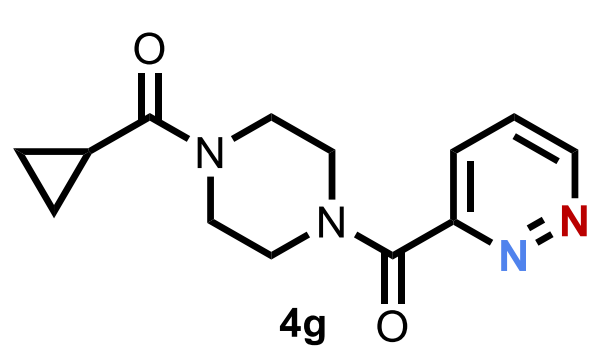
4g

(4-(Cyclopropanecarbonyl)piperazin-1-yl)(pyridazin-3-yl)methanone (4g)

Prepared according to the general procedure using (4-(6-chloropicolinoyl)piperazin-1-yl)(cyclopropyl)methanone (**1g**, 88 mg, 0.30 mmol, 1.0 equiv.). The crude material was purified by flash column chromatography (SiO_2, EtOAc/Hex 20 to 100 %) and then preparative TLC (100% EtOAc) to give the corresponding pyridazine **4g** (32 mg, 0.13 mmol, 42 % yield) as a as a colorless oil.

^{1}H NMR (400 MHz, $CDCl_3$) δ 9.25 (dd, *J* = 5.0, 1.8 Hz, 1H, N=N-C*H*), 7.92 (br. s, 1H, N=N-CH-CH-C*H*), 7.64 (dd, *J* = 8.5, 5.0 Hz, 1H, N=N-CH-C*H*), 3.99 – 3.65 (m, 8H, piperaz), 1.75 (br. s, 1H, C*H*-CO), 1.11 – 0.92 (m, 2H, cyprop), 0.92 – 0.68 (m, 2H, cyprop).

^{13}C{^{1}H} NMR (101 MHz, $CDCl_3$) δ 172.5, 165.1, 156.6, 152.0, 128.3, 127.5, 47.6, 43.1, 11.2, 7.8.

HRMS (APCI/QTOF) m/z: [M + H]$^+$ Calculated for $C_{13}H_{17}N_4O_2^+$ 261.1346; Found 261.1346.

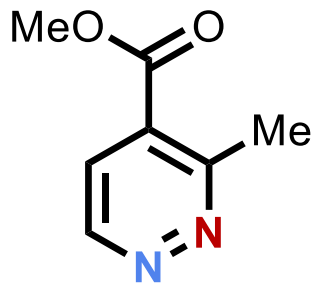

4h

Methyl 3-methylpyridazine-4-carboxylate (4h)
Prepared according to the general procedure using methyl 2-chloro-3-methylisonicotinate (**1h**, 56 mg, 0.30 mmol, 1.0 equiv.). The crude material was purified by flash column chromatography (SiO_2, EtOAc/Hex 20 to 100 %) and then preparative TLC (100% EtOAc) to give the corresponding pyridazine **4h** (19 mg, 0.13 mmol, 41 % yield) as a white-solid.

^{1}H NMR (400 MHz, $CDCl_3$) δ 9.24 (d, *J* = 5.1 Hz, 1H, N=N-C*H*), 7.83 (d, *J* = 5.1 Hz, 1H, N=N-CH-C*H*), 3.97 (s, 3H, COOCH_3), 2.99 (s, 3H, HetArCH_3).
^{13}C{^{1}H} NMR (101 MHz, $CDCl_3$) δ 165.6, 159.1, 150.5, 128.0, 126.3, 53.1, 22.3.
HRMS (APCI/QTOF) m/z: $[M + H]^+$ Calculated for $C_7H_9N_2O_2^+$ 153.0659; Found 153.0661.

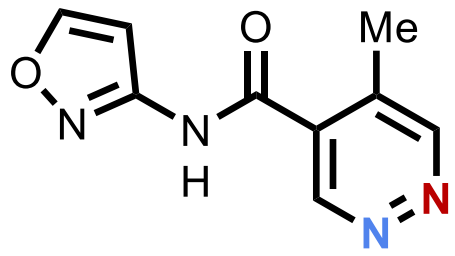

4i

***N*-(Isoxazol-3-yl)-5-methylpyridazine-4-carboxamide (4i)**
Prepared according to the general procedure using 6-chloro-N-(isoxazol-3-yl)-4-methylnicotinamide (**1i**, 71 mg, 0.30 mmol, 1.0 equiv.). The crude material was purified by flash column chromatography (SiO_2, EtOAc/Hex 20 to 100 %) and then preparative TLC (100% EtOAc) to give the corresponding pyridazine **4i** (28 mg, 0.14 mmol, 45 % yield) as a white-solid.

^{1}H NMR (400 MHz, MeOD) δ 9.24 (s, 1H, N=N-C*H*), 9.22 (s, 1H, N=N-C*H*), 8.63 (d, *J* = 1.8 Hz, 1H, O-C*H*), 7.08 (d, *J* = 1.8 Hz, 1H, O-CH-C*H*), 2.53 (s, 3H, HetArCH_3).
^{13}C{^{1}H} NMR (101 MHz, MeOD) δ 165.0, 161.1, 158.5, 155.5, 149.0, 138.4, 135.0, 100.3, 16.5.
HRMS (APCI/QTOF) m/z: $[M + H]^+$ Calculated for $C_9H_9N_4O_2^+$ 205.0720; Found 205.0720.
X-ray crystallography: Crystals suitable for single crystal X-ray crystallography were grown through slow evaporation of a saturated solution of the compound in CD_3OD. The crystal structure is deposited in the Cambridge Crystallographic Data Centre CCDC 2431452.

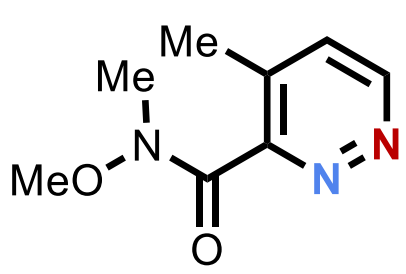

4j

***N*-Methoxy-*N*,4-dimethylpyridazine-3-carboxamide (4j)**
Prepared according to the general procedure using 6-chloro-N-methoxy-*N*,3-dimethylpicolinamide (**1j**, 64 mg, 0.30 mmol, 1.0 equiv.). The crude material was purified by flash column chromatography (SiO_2, EtOAc/Hex 20 to 100 %) and then preparative TLC (100% EtOAc) to give the corresponding pyridazine **4j** (28 mg, 0.15 mmol, 52 % yield) as a colorless oil.

^{1}H NMR (400 MHz, $CDCl_3$) δ 9.05 (d, *J* = 5.3 Hz, 1H, N=N-C*H*), 7.34 (d, *J* = 5.3 Hz, 1H, N=N-C*H*), 3.61 (s, 3H, OCH_3), 3.42 (s, 3H, NCH_3), 2.35 (s, 3H, HetArCH_3). Note: minor rotamer (confirmed by 1D-NOESY) peaks appear at 3.95 (s, OCH_3), 3.25 (s, NCH_3), 2.44 (s, HetArCH_3).
^{13}C{^{1}H} NMR (101 MHz, $CDCl_3$) δ 167.1, 151.2 (2C), 135.8, 127.8, 62.0, 32.1, 17.5.
HRMS (APCI/QTOF) m/z: $[M + H]^+$ Calculated for $C_8H_{12}N_3O_2^+$ 182.0924; Found 182.0921.

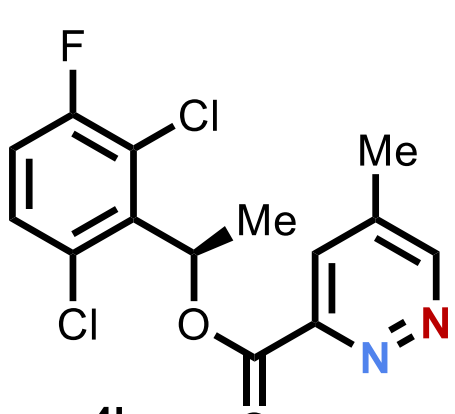

4k

(*R*)-1-(2,6-Dichloro-3-fluorophenyl)ethyl 5-methylpyridazine-3-carboxylate (4k)
Prepared according to the general procedure using (*R*)-1-(2,6-dichloro-3-fluorophenyl)ethyl 6-chloro-4-methylpicolinate (**1k**, 104 mg, 0.30 mmol, 1.0 equiv.). The crude material was purified by flash column chromatography (SiO_2, EtOAc/Hex 20 to 100 %) and then preparative TLC (100% EtOAc) to give the corresponding pyridazine **4k** (40 mg, 0.12 mmol, 41 % yield) as a colorless oil. The enantiomeric excess was confirmed to be >99% e. e. by chiral HPLC analysis (Daicel CHIRALPAK IA column hexane:IPA 80:20, 0.8 mL/min, λ = 230 nm, τmajor = 11.7 min, τminor = 13.1 min).

^{1}H NMR (400 MHz, $CDCl_3$) δ 9.18 (d, *J* = 2.2 Hz, 1H, N=N-C*H*), 8.00 (dd, *J* = 2.2, 1.0 Hz, 1H, N=N-CH-C(CH_3)-C*H*), 7.29 (dd, *J* = 8.9, 4.9 Hz, 1H, Ar*H*), 7.06 (dd, *J* = 8.9, 8.0 Hz, 1H, Ar*H*), 6.76 (q, *J* = 6.9 Hz, 1H, C*H*-CH_3), 2.45 (s, 3H, HetAr-CH_3), 1.88 (d, *J* = 6.9 Hz, 3H, CH-CH_3).
^{13}C{^{1}H} NMR (101 MHz, $CDCl_3$) δ 163.4, 157.6 (d, J_{C-F} = 248.9 Hz), 154.7, 150.9, 138.5, 137.0, 129.8 (d, J_{C-F} = 7.5 Hz), 129.2 (d, J_{C-F} = 3.8 Hz), 128.1, 122.3 (d, J_{C-F} = 19.4 Hz), 116.6 (d, J_{C-F} = 23.1 Hz), 71.7 (d, J_{C-F} = 2.3 Hz), 18.6, 18.2.
HRMS (APCI/QTOF) m/z: $[M + H]^+$ Calculated for $C_{14}H_{12}Cl_2FN_2O_2^+$ 329.0254; Found 329.0255.

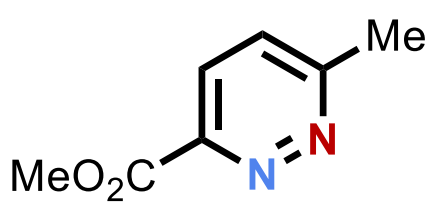
4l

Methyl 6-methylpyridazine-3-carboxylate (4l)
Prepared according to the general procedure using methyl 6-chloro-5-methylpicolinate (**1l**, 56 mg, 0.30 mmol, 1.0 equiv.). The crude material was purified by flash column chromatography (SiO_2, EtOAc/Hex 20 to 100 %) and then preparative TLC (100 EtOAc) to give the corresponding pyridazine **4l** (30 mg, 0.20 mmol, 66 % yield) as a white-solid.
^{1}H NMR (400 MHz, $CDCl_3$) δ 8.07 (d, *J* = 8.6 Hz, 1H, C(CO_2Me)-C*H*), 7.46 (d, *J* = 8.6 Hz, 1H, C(CH_3)-C*H*), 4.06 (s, 3H, OCH_3), 2.81 (s, 3H, CH_3).
^{13}C{^{1}H} NMR (101 MHz, $CDCl_3$) δ 165.0, 162.8, 149.8, 127.6, 127.3, 53.3, 22.7.
HRMS (APCI/QTOF) m/z: $[M + H]^+$ Calculated for $C_7H_9N_2O_2^+$ 153.0659; Found 153.0650.

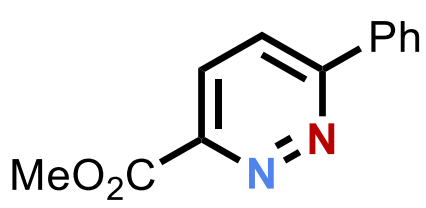
4m

Methyl 6-phenylpyridazine-3-carboxylate (4m)
Prepared according to the general procedure using pyridine **1m** (64 mg, 0.30 mmol, 1.0 equiv.). The crude material was purified by flash column chromatography (SiO_2, EtOAc/Hex 20 to 100 %) and then preparative TLC (100 % EtOAc) to give the corresponding pyridazine **4m** (39 mg, 0.18 mmol, 61 % yield) as a white-solid.
^{1}H NMR (400 MHz, $CDCl_3$) δ 8.24 (d, *J* = 8.8 Hz, 1H, C(CO_2Me)-C*H*), 8.19–8.14 (m, 2H, C(Het)–C*H*), 8.00 (d, *J* = 8.8 Hz, 1H, C(Ph)-C*H*), 7.60–7.52 (m, 3H), 4.10 (s, 3H).
^{13}C{^{1}H} NMR (101 MHz, $CDCl_3$) δ 164.9, 160.9, 150.1, 135.5, 131.1, 129.3, 128.2, 127.7, 124.0, 53.4.
HRMS (APCI/QTOF) m/z: $[M + H]^+$ Calculated for $C_{12}H_{11}N_2O_2^+$ 215.0815; Found 215.0814.

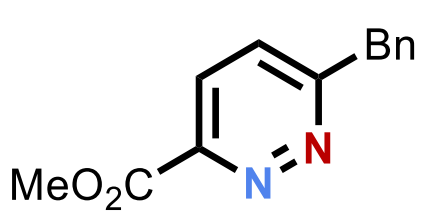
4n

Methyl 6-benzylpyridazine-3-carboxylate (4n)
Prepared according to the general procedure using pyridine **1n** (68 mg, 0.30 mmol, 1.0 equiv.). The crude material was purified by flash column chromatography (SiO_2, EtOAc/Hex 20 to 100 %) and then preparative TLC (100 % EtOAc) to give the corresponding pyridazine **4n** (27 mg, 0.12 mmol, 40 % yield) as a white-solid.
^{1}H NMR (500 MHz, $CDCl_3$) δ 8.07 (d, *J* = 8.5 Hz, 1H, C(CO_2Me)-C*H*), 7.39 (d, *J* = 8.5 Hz, 1H, C(Bn)-C*H*), 7.37–7.32 (m, 2H), 7.31–7.25 (m, 3H), 4.48 (s, 2H), 4.08 (s, 3H).
^{13}C{^{1}H} NMR (126 MHz, $CDCl_3$) δ 165.30, 164.90, 150.15, 137.56, 129.36, 129.12, 127.98, 127.28, 127.12, 53.38, 42.60.
HRMS (APCI/QTOF) m/z: $[M + H]^+$ Calculated for $C_{13}H_{13}N_2O_2^+$ 229.0972; Found 229.0970.

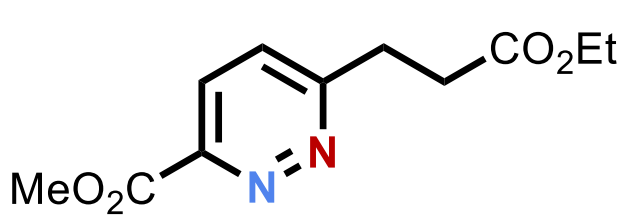
4o

Methyl 6-(3-ethoxy-3-oxopropyl)pyridazine-3-carboxylate (4o)
Prepared according to the general procedure using pyridine **1o** (71 mg, 0.30 mmol, 1.0 equiv.). The crude material was purified by flash column chromatography (SiO_2, EtOAc/Hex 20 to 100 %) and then preparative TLC (100 % EtOAc) to give the corresponding pyridazine **4o** (43 mg, 0.18 mmol, 60 % yield) as a white-solid.

^{1}H NMR (500 MHz, $CDCl_3$) δ 8.06 (d, J = 8.6 Hz, 1H, HetArH), 7.52 (d, J = 8.6 Hz, 1H, HetArH), 4.07 (q, J = 7.1 Hz, 2H, OCH_2CH_3), 4.02 (s, 3H, OCH_3), 3.32 (t, J = 7.1 Hz, 2H, HetArCH_2-CH_2), 2.93 (t, J = 7.1 Hz, 2H, HetArCH_2-CH_2), 1.19 (t, J = 7.1 Hz, 3H, OCH_2CH_3).
^{13}C{^{1}H} NMR (126 MHz, $CDCl_3$) δ 172.5, 164.8, 164.4, 150.1, 127.6, 127.4, 60.8, 53.3, 32.6, 31.1, 14.2.
HRMS (APCI/QTOF) m/z: $[M + H]^+$ Calculated for $C_{11}H_{15}N_2O_4^+$ 239.1026; Found 239.1025.

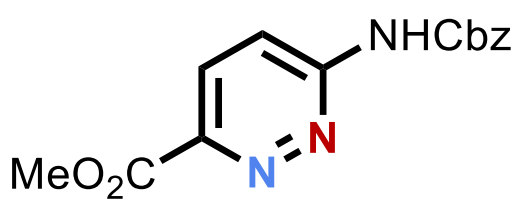

4p

Methyl 6-(((benzyloxy)carbonyl)amino)pyridazine-3-carboxylate (4p)
Prepared according to the general procedure using pyridine **1p** (86 mg, 0.30 mmol, 1.0 equiv.). The crude material was purified by flash column chromatography (SiO_2, EtOAc/Hex 20 to 100 %) and then preparative TLC (100 % EtOAc) to give the corresponding pyridazine **4p** (37 mg, 0.13 mmol, 43 % yield) as a white-solid.
^{1}H NMR (500 MHz, $CDCl_3$) δ 8.38 (d, J = 9.0 Hz, 1H, C(CO_2Me)-CH), 8.33 (brs, 1H), 8.18 (d, J = 9.0 Hz, 1H, C(NHCBz)-CH), 7.44–7.33 (m, 5H), 5.27 (s, 2H), 4.05 (s, 3H).
^{13}C{^{1}H} NMR (101 MHz, $CDCl_3$) δ 164.3, 156.4, 152.8, 148.2, 135.2, 130.0, 128.9, 128.8, 128.5, 116.8, 68.1, 53.3.
HRMS (APCI/QTOF) m/z: $[M + H]^+$ Calculated for $C_{14}H_{14}N_3O_4^+$ 288.0979; Found 288.0981.

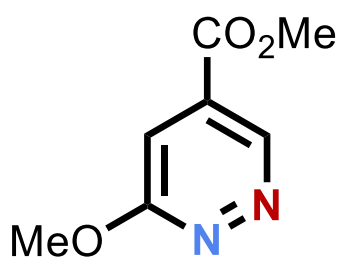

4q

Methyl 6-methoxypyridazine-4-carboxylate (4q)
Prepared according to the general procedure using methyl 2-chloro-6-methoxyisonicotinate (**1q**, 60 mg, 0.30 mmol, 1.0 equiv.). The crude material was purified by flash column chromatography (SiO_2, EtOAc/Hex 20 to 100 %) and then preparative TLC (100% EtOAc) to give the corresponding pyridazine **4q** (20 mg, 0.12 mmol, 40 % yield) as a white solid.
^{1}H NMR (400 MHz, $CDCl_3$) δ 9.28 (d, J = 1.7 Hz, 1H, N=N-CH), 7.47 (d, J = 1.7 Hz, 1H, N=N-C-C-H), 4.18 (s, 3H, CO_2CH_3), 3.98 (s, 3H, OCH_3).
^{13}C{^{1}H} NMR (101 MHz, $CDCl_3$) δ 165.9, 164.1, 145.6, 130.9, 117.4, 55.6, 53.3.
HRMS (APCI/QTOF) m/z: $[M + H]^+$ Calculated for $C_7H_9N_2O_3^+$ 169.0608; Found 169.0611.

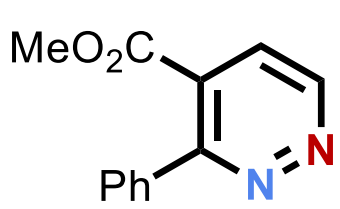

4r

Methyl 3-phenylpyridazine-4-carboxylate (4r)
Prepared according to the general procedure using methyl 6-chloro-2-phenylnicotinate (**1r**, 74 mg, 0.30 mmol, 1.0 equiv.). The crude material was purified by flash column chromatography (SiO_2, EtOAc/Hex 20 to 100 %) and then preparative TLC (100 % EtOAc) to give the corresponding pyridazine **4r** (30 mg, 0.14 mmol, 47 % yield) as a colorless oil.
^{1}H NMR (400 MHz, $CDCl_3$) δ 9.33 (d, J = 5.1 Hz, 1H, N=N-CH), 7.76 (d, J = 5.1 Hz, 1H, N=N-CH-CH), 7.70 – 7.57 (m, 2H, ArH), 7.58 – 7.46 (m, 3H, ArH), 3.76 (s, 3H, OCH_3).
^{13}C{^{1}H} NMR (101 MHz, $CDCl_3$) δ 166.9, 158.9, 150.1, 136.7, 129.8, 129.7, 129.0, 128.6, 125.9, 53.1.
HRMS (APCI/QTOF) m/z: $[M + H]^+$ Calculated $C_{12}H_{11}N_2O_2^+$ 215.0815; Found 215.0820.

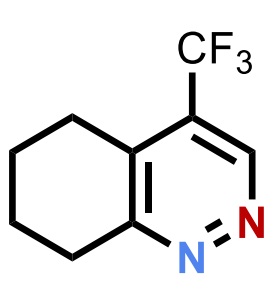

4s

4-(Trifluoromethyl)-5,6,7,8-tetrahydrocinnoline (4s)
Prepared according to the general procedure using chloropyridine **1s** (71 mg, 0.30 mmol, 1.0 equiv.). The crude material was purified by flash column chromatography (SiO_2, EtOAc/Hex 20 to 100 %) and then preparative TLC (50% Hexanes/EtOAc) to give the corresponding pyridazine **4s** (30 mg, 0.15 mmol, 50 % yield) as a pale-yellow oil.

^{1}H NMR (400 MHz, $CDCl_3$) δ 9.12 (s, 1H, N=N-C*H*), 3.26 (t, *J* = 6.5 Hz, 2H, N=N-C-CH_2), 2.98 (dt, *J* = 6.9, 3.5 Hz, 2H, C*H*-C-C-CF_3), 2.02 – 1.92 (m, 2H, CH_2-CH_2-CH_2-CH_2), 1.92 – 1.84 (m, 2H, CH_2-CH_2-CH_2-CH_2).
^{13}C{^{1}H} NMR (101 MHz, $CDCl_3$) δ 161.9, 144.2 (q, J_{C-F} = 5.2 Hz), 135.4 (q, J_{C-F} = 1.9 Hz), 126.8 (q, J_{C-F} = 31.9 Hz), 123.0 (q, J_{C-F} = 275.4 Hz), 30.6, 24.8 (q, J_{C-F} = 1.8 Hz), 21.7, 21.4.
^{19}F{^{1}H} NMR (471 MHz, $CDCl_3$) δ -63.1.
HRMS (APCI/QTOF) m/z: $[M + H]^+$ Calculated for $C_9H_{10}F_3N_2^+$ 203.0791; Found 203.0794.

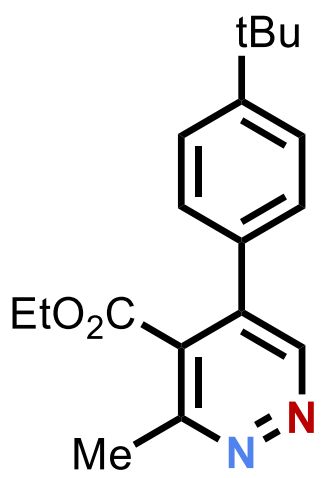

4t

Ethyl 5-(4-(*tert*-butyl)phenyl)-3-methylpyridazine-4-carboxylate (4t)
Prepared according to the general procedure using chloropyridine **1t** (100 mg, 0.300 mmol, 1.0 equiv.). The crude material was purified by flash column chromatography (SiO_2, EtOAc/Hex 20 to 100 %) and then preparative TLC (100% EtOAc) to give the corresponding pyridazine **4t** (44 mg, 0.15 mmol, 49 % yield) as a pale-yellow oil.
1 mmol scale: A 20 mL screw-cap vial equipped with a Teflon coated stirring bar was charged with chloropyridine **1t** (331 mg, 1.00 mmol, 1.00 equiv.), *tert*-butyl (((3,5-bis(trifluoromethyl)phenyl)sulfonyl)oxy)carbamate (614 mg, 1.50 mmol, 1.50 equiv.) and TFE (5.0 mL). Then, TFA (0.15 mL, 0.23 g, 2.0 mmol, 2.0 equiv.) was added and the resulting mixture stirred for 16 hours at 40 °C. The reaction mixture was cooled down to room temperature then concentrated *in vacuo*. The vial was charged with PPTS (0.50 g, 2.0 mmol, 2.0 equiv.), NaN_3 (0.10 g, 1.5 mmol, 1.5 equiv.) and IPA (20.0 mL). The vial was then vigorously stirred for 2 h at 22 °C. The resulting suspension was filtered and concentrated *in vacuo*. The residue is dissolved in MeCN (5 mL) and again concentrated *in vacuo* to remove residual IPA. The residue was dissolved in MeCN (5.0 mL) and filtered through a 0.20 µm syringe filter, which was then washed with MeCN (2 × 5 mL). The filtrate was charged with benzophenone (10.9 mg, 60.0 µmol, 20 mol %) and extra MeCN (5 mL), sparged with N_2 gas for 1 minute and then photolyzed with 390 nm Kessil LED lamp for 20 hours. Then, the mixture sparged with N_2 for 20 minutes, was concentrated *in vacuo*. The crude mixture was purified by silica gel plug (SiO_2, EtOAc), followed by silica gel chromatography (SiO_2, 0-60 % EtOAc/Hexanes) to give pyridazine **4t** (149 mg, 0.500 mmol, 50 % yield) as a pale-yellow oil.
^{1}H NMR (400 MHz, $CDCl_3$) δ 9.16 (s, 1H, N=N-C*H*), 7.49 (d, *J* = 8.4 Hz, 2H, Ar*H*), 7.35 (d, *J* = 8.4 Hz, 2H, Ar*H*), 4.23 (q, *J* = 7.1 Hz, 2H, OC$H_2$$CH_3$), 2.78 (s, 3H, C$H_3$), 1.35 (s, 9H, C(C$H_3$)$_3$), 1.07 (t, *J* = 7.1 Hz, 3H, OCH_2CH_3).
^{13}C{^{1}H} NMR (101 MHz, CDCl3) δ 166.6, 155.8, 153.2, 150.2, 136.3, 131.4, 130.3, 128.1, 126.2, 62.2, 35.0, 31.4, 20.6, 13.8.
HRMS (APCI/QTOF) m/z: $[M + H]^+$ Calculated for $C_{18}H_{23}N_2O_2^+$ 299.1754; Found 299.1750.

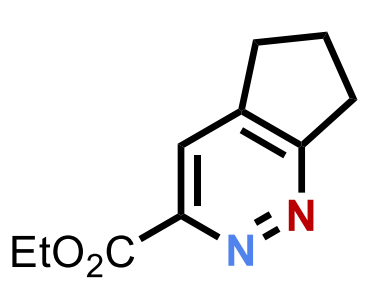

4u

Ethyl 6,7-dihydro-5H-cyclopenta[c]pyridazine-3-carboxylate (4u)
Prepared according to the general procedure using ethyl 1-chloro-6,7-dihydro-5H-cyclopenta[c]pyridine-3-carboxylate (**1u**, 68 mg, 0.30 mmol, 1.0 equiv.). The crude material was purified by flash column chromatography (SiO_2, EtOAc/Hex 20 to 100 %) and then preparative TLC (100 % EtOAc) to give the corresponding pyridazine **4u** (33 mg, 0.17 mmol, 57 % yield) as a white-solid.
^{1}H NMR (400 MHz, $CDCl_3$) δ 7.99 (t, *J* = 1.3 Hz, 1H, HetAr*H*), 4.52 (q, *J* = 7.1 Hz, 2H, OC$H_2$$CH_3$), 3.30 (t, *J* = 7.7 Hz, 2H, -CH_2-CH_2-CH_2-), 3.04 (td, *J* = 7.7, 1.3 Hz, 2H, -CH_2-CH_2-CH_2-), 2.21 (p, *J* = 7.7 Hz, 2H, -CH_2-CH_2-CH_2-), 1.46 (t, *J* = 7.1 Hz, 3H, OCH_2CH_3).
^{13}C{^{1}H} NMR (101 MHz, $CDCl_3$) δ 170.8, 164.9, 149.9, 143.4, 123.6, 62.5, 32.6, 30.8, 22.9, 14.4.
HRMS (APCI/QTOF) m/z: $[M + H]^+$ Calculated for $C_{10}H_{13}N_2O_2^+$ 193.0972; Found 193.0974.

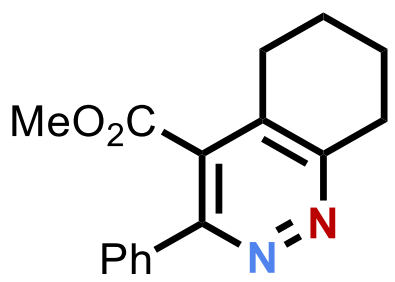

4v

Methyl 3-phenyl-5,6,7,8-tetrahydrocinnoline-4-carboxylate (4v)

Prepared according to the general procedure using methyl 1-chloro-3-phenyl-5,6,7,8-tetrahydroisoquinoline-4-carboxylate (**1v**, 90 mg, 0.30 mmol, 1.0 equiv.). The crude material was purified by flash column chromatography (SiO_2, EtOAc/Hex 20 to 100 %) and then preparative TLC (100 % EtOAc) to give the corresponding pyridazine **4v** (45 mg, 0.17 mmol, 56 % yield) as a white-solid.

^{1}H NMR (400 MHz, $CDCl_3$) δ 7.70 – 7.57 (m, 2H, Ar*H*), 7.53 – 7.36 (m, 3H, Ar*H*), 3.70 (s, 3H, OC*H*$_3$), 3.24 (t, *J* = 6.4 Hz, 2H, -CH_2-CH_2-CH_2-C*H*$_2$-), 2.83 (t, *J* = 6.4 Hz, 2H, -C*H*$_2$-CH_2-CH_2-CH_2-), 2.02 – 1.92 (m, 2H, -CH_2-CH_2-C*H*$_2$-CH_2-), 1.92 – 1.82 (m, 2H, -CH_2-C*H*$_2$-CH_2-CH_2-).

^{13}C{^{1}H} NMR (101 MHz, $CDCl_3$) δ 167.4, 159.4, 154.9, 137.0, 133.9, 130.6, 129.4, 128.7, 128.5, 52.7, 30.4, 25.7, 22.1, 21.7.

HRMS (APCI/QTOF) m/z: $[M + H]^+$ Calculated for $C_{16}H_{17}N_2O_2^+$ 269.1285; Found 269.1289.

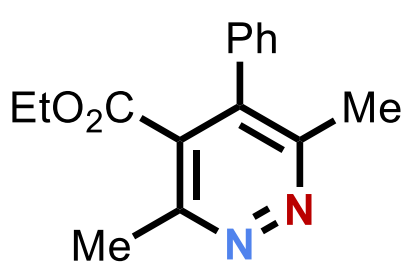

4w

Ethyl 3,6-dimethyl-5-phenylpyridazine-4-carboxylate (4w)

Prepared according to the general procedure using ethyl 6-chloro-2,5-dimethyl-4-phenylnicotinate (**1w**, 87 mg, 0.30 mmol, 1.0 equiv.). The crude material was purified by flash column chromatography (SiO_2, EtOAc/Hex 20 to 100 %) and then preparative TLC (100 % EtOAc) to give the corresponding pyridazine **1w** (45 mg, 0.18 mmol, 59 % yield) as a white-solid.

^{1}H NMR (400 MHz, $CDCl_3$) δ 7.47 – 7.41 (m, 3H, Ar*H*), 7.25 – 7.18 (m, 2H, Ar*H*), 4.05 (q, *J* = 7.1 Hz, 2H, OCH_2CH_3), 2.72 (s, 3H, HetArC*H*$_3$), 2.52 (s, 3H, HetArC*H*$_3$), 0.94 (t, *J* = 7.1 Hz, 3H, OCH_2C*H*$_3$).

^{13}C{^{1}H} NMR (101 MHz, $CDCl_3$) δ 166.3, 157.1, 153.9, 136.5, 134.6, 131.5, 129.0, 128.8, 128.4, 61.9, 21.1, 20.3, 13.7.

HRMS (APCI/QTOF) m/z: $[M + H]^+$ Calculated $C_{15}H_{17}N_2O_2^+$ 257.1285; Found.257.1289.

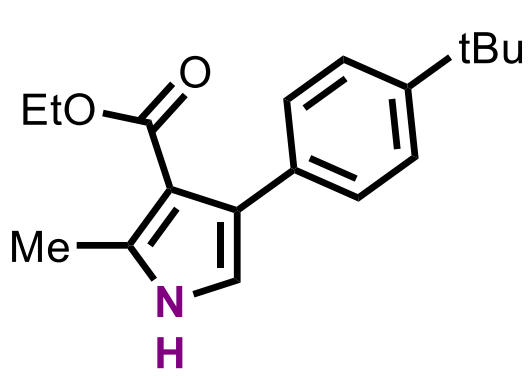

6

Ethyl 4-(4-(*tert*-butyl)phenyl)-2-methyl-1H-pyrrole-3-carboxylate (6)

An 8 mL screw-cap vial equipped with a Teflon coated magnetic stirring bar was charged with pyridazine **1t** (30 mg, 0.10 mmol, 1.0 equiv.), Zn dust (65 mg, 1.0 mmol, 10 equiv.) and AcOH (1 mL). The resulting mixture was then stirred at 40 °C for 16 hours. Then, the mixture was cooled down, filtered through a pad of celite (eluting with 2× 1mL MeOH). The filtrate was concentrated in vacuo, then dissolved in DCM (3 mL) and washed with aq. NaOH (1M, 3 mL) and brine (3 mL). The organic layer was dried over Na_2SO_4, filtered and concentrated in vauco. The cure was purified via preparative TLC (20% EtOAc/Hexanes) to give pyrrole **6** (15 mg, 0.053 mmol, 53 %) as a white solid.

^{1}H NMR (500 MHz, $CDCl_3$) δ 8.21 (br. s, 1H), 7.40 – 7.29 (m, 4H, Ar*H*), 6.57 (d, *J* = 2.4 Hz, 1H, HetAr*H*), 4.18 (q, *J* = 7.1 Hz, 2H, OC*H*$_2$$CH_3$), 2.53 (s, 3H, HetArC*H*$_3$), 1.34 (s, 9H, C(C*H*$_3$)$_3$), 1.16 (t, *J* = 7.1 Hz, 3H, OCH_2CH_3).

^{13}C{^{1}H} NMR (126 MHz, $CDCl_3$) δ 165.9, 149.1, 136.0, 132.9, 129.1, 127.4, 124.6, 115.4, 110.3, 59.5, 34.6, 31.6 (2C), 14.2.

HRMS (APCI/QTOF) m/z: $[M + H]^+$ Calculated for $C_{18}H_{24}NO_2^+$ 286.1802; Found 286.1801.

N,*N*-Diethyl-5-isopropylpyridazine-4-carboxamide (4x)

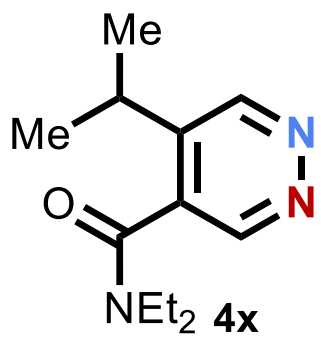

4x

Prepared according to the general procedure using fluoropyridine **1x** (71 mg, 0.30 mmol, 1.0 equiv.). The crude material was purified by flash column chromatography (SiO_2, EtOAc/Hex 20 to 100 %) and then preparative TLC (EtOAc) to give the corresponding pyridazine **4x** (15 mg, 0.067 mmol, 23 % yield) as a yellow oil.

<u>^{1}H NMR</u> (500 MHz, $CDCl_3$) δ 9.20 (s, 1H, N=N-C*H*), 8.89 (s, 1H, N=N-C*H*), 3.69 (br. s, 1H, $CH_aH_bCH_3$), 3.49 (s, 1H, rot. $CH_aH_bCH_3$), 3.10 (br. s, 2H, rot. CH_2CH_3), 2.98 (p, *J* = 7.0 Hz, 1H, rot. C*H*(CH_3)$_2$), 1.32 (dd, *J* = 7.0, 1.7 Hz, 6H, CH(CH_3)$_2$), 1.10 (t, *J* = 7.2 Hz, 3H, rot. CH_2CH_3), 1.10 (t, *J* = 7.2 Hz, 3H, rot. CH_2CH_3).

<u>^{13}C NMR</u> (126 MHz, $CDCl_3$) δ 165.6, 150.9, 147.2, 143.2, 133.7, 43.1, 39.2, 29.4, 22.9, 22.3, 14.2, 12.8.

<u>HRMS</u> (APCI/QTOF) m/z: $[M + H]^+$ Calculated for $C_{12}H_{20}N_3O^+$ 222.1601; Found 222.1600.

N,*N*-Diethyl-3-isopropylpyridazine-4-carboxamide (4y)

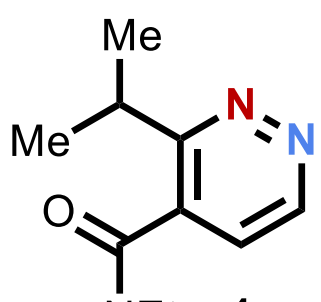

4y

Prepared according to the general procedure using fluoropyridine **1y** (71 mg, 0.30 mmol, 1.0 equiv.). The crude material was purified by flash column chromatography (SiO_2, EtOAc/Hex 20 to 100 %) and then preparative TLC (EtOAc) to give the corresponding pyridazine **4y** (31 mg, 0.14 mmol, 47 % yield) as a yellow oil.

<u>^{1}H NMR</u> (500 MHz, $CDCl_3$) δ 9.09 (d, *J* = 5.0 Hz, 1H, N=N-C*H*), 7.18 (d, *J* = 5.0 Hz, 1H, N=N-CH-C*H*), 3.75 (br. s, 1H, $CH_aH_bCH_3$), 3.40 (br. s, 1H, $CH_aH_bCH_3$), 3.20 – 2.94 (m, 3H, rot. CH_2CH_3 and C*H*(CH_3)), 1.42 (br. s, 6H, CH(CH_3)$_2$), 1.27 (t, *J* = 7.2 Hz, 3H, rot. CH_2CH_3), 1.07 (t, *J* = 7.2 Hz, 3H, rot. CH_2CH_3).

<u>^{13}C NMR</u> (126 MHz, $CDCl_3$) δ 166.4, 164.0, 149.4, 134.2, 122.4, 43.0, 39.1, 32.7, 23.1, 21.7 14.1, 12.7.

<u>HRMS</u> (APCI/QTOF) m/z: $[M + H]^+$ Calculated for $C_{12}H_{20}N_3O^+$ 222.1601; Found 222.1615.

Methyl 5-isopropylpyridazine-4-carboxylate (4z)

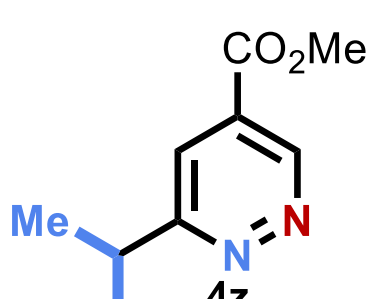

4z

Prepared according to the general procedure using methyl 2-chloro-6-isopropylisonicotinate (**1z**, 64 mg, 0.30 mmol, 1.0 equiv.). The crude material was purified by flash column chromatography (SiO_2, EtOAc/Hex 20 to 100 %) and then preparative TLC (100 % EtOAc) to give the corresponding pyridazine **4z** (24 mg, 0.13 mmol, 44 % yield) as a colorless oil.

<u>^{1}H NMR</u> (400 MHz, $CDCl_3$) δ 9.50 (dd, *J* = 1.9, 0.6 Hz, 1H, N=N-C*H*), 7.85 (d, *J* = 1.9 Hz, 1H, HetAr*H*), 4.00 (s, 3H, OCH_3), 3.43 (p, *J* = 7.0 Hz, 1H, C*H*(CH_3)$_2$), 1.41 (dd, *J* = 7.0, 0.6 Hz, 6H, CH(CH_3)$_2$).

<u>^{13}C{^{1}H} NMR</u> (101 MHz, $CDCl_3$) δ 169.5, 164.8, 147.9, 128.2, 123.8, 53.2, 35.1, 22.4.

<u>HRMS</u> (APCI/QTOF) m/z: $[M + H]^+$ Calculated for $C_9H_{13}N_2O_2^+$ 181.0972; Found 181.0968.

Methyl 5-isopropylpyridazine-4-carboxylate (11)

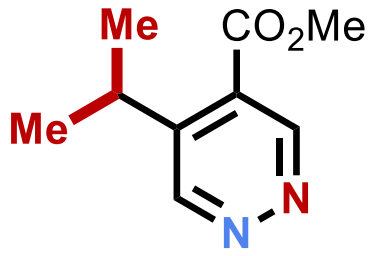

11

Based on a reported procedure,(*46*) a 8 mL scintillation vial equipped with a Teflon coated magnetic stirrer was charged with methyl pyridazine-4-carboxylate (**4b**, 25 mg, 0.18 mmol, 1.0 equiv.), $AgNO_3$ (6.1mg, 36 μmol, 0.20 equiv.), Selectfluor (127 mg, 0.36 mmol, 2.0 equiv.)), DCE (0.45 mL) and water (0.45 mL). Then, TFA (15 μL, 21 mg, 1.0 equiv.) and isobutyric acid (32 mg, 33 μL, 0.36 mmol, 2.0 equiv.) were added and the mixture stirred at 50 °C for 2 h. The mixture was allowed to cool down, dilted with EtOAc (10 mL) and washed with NaOH (1M, 3 × 5 mL) and brine (5 mL). The organic layer was dried over Na_2SO_4, filtered and concentrated *in vacuo*. The

crude mixture was purified via preperative TLC (100% EtOAc) to give pyridazine **11** (14 mg, 0.078 mmol, 44 % yield)
<u>^{1}H NMR</u> (400 MHz, $CDCl_3$) δ 9.34 (s, 1H, N=N-C*H*), 9.33 (s, 1H, N=N-C*H*), 3.99 (s, 3H, OCH_3), 3.82 (p, *J* = 6.9 Hz, 1H, C*H*$(CH_3)_2$), 1.34 (d, *J* = 6.9 Hz, 6H, $CH(CH_3)_2$).
<u>$^{13}C\{^{1}H\}$ NMR</u> (101 MHz, $CDCl_3$) δ 165.3, 151.3, 149.3, 147.3, 126.6, 53.1, 28.1, 22.6.
<u>HRMS</u> (APCI/QTOF) m/z: $[M + H]^+$ Calculated for $C_9H_{13}N_2O_2^+$ 181.0972; Found 181.0976.
Spectral data matched those reported in the literature.(*45*)

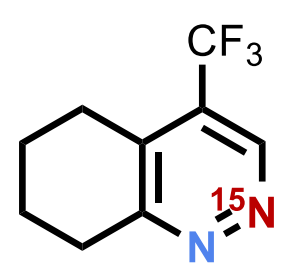

^{15}N(2)-4r

4-(Trifluoromethyl)-5,6,7,8-tetrahydrocinnoline-2-^{15}N (^{15}N(2)-4r)

Prepared according to the general procedure using chloropyridine **1r** (71 mg, 0.30 mmol, 1.0 equiv.) and the labeled aminating reagent (62 mg, 0.45 mmol, 1.5 equiv.). The crude material was purified by flash column chromatography (SiO_2, EtOAc/Hex 20 to 100 %) and then preparative TLC (50% Hexanes/EtOAc) to give the corresponding pyridazine **^{15}N(2)-4r** (25 mg, 0.12 mmol, 41 % yield) as a pale-yellow oil.
<u>^{1}H NMR</u> (600 MHz, $CDCl_3$) δ 9.13 (d, $J_{N\text{-}H}$ = 11.2 Hz, 1H), 3.27 (t, *J* = 6.5 Hz, 2H, C*H*$_2$-CH_2-CH_2-CH_2), 2.98 (t, *J* = 6.5 Hz, 2H, CH_2-CH_2-CH_2-C*H*$_2$), 2.02 – 1.95 (m, 2H, CH_2-C*H*$_2$-CH_2-CH_2), 1.92 – 1.85 (m, 2H, CH_2-CH_2-C*H*$_2$-CH_2).
<u>$^{13}C\{^{1}H\}$ NMR</u> (151 MHz, $CDCl_3$) δ 161.9 (d, $J_{C\text{-}N}$ = 4.9 Hz), 144.2 (dq, $J_{C\text{-}N/C\text{-}F}$ = 6.6, 5.3 Hz), 135.4 (dd, $J_{C\text{-}N/C\text{-}F}$ = 6.3, 1.9 Hz), 126.8 (qd, $J_{C\text{-}F/C\text{-}N}$ = 31.7, 2.0 Hz), 123.0 (q, $J_{C\text{-}F}$ = 275.3 Hz), 30.6 (d, $J_{C\text{-}N}$ = 1.7 Hz), 24.8 (q, $J_{C\text{-}F}$ = 1.7 Hz), 21.8, 21.4.
<u>^{19}F NMR</u> (565 MHz, $CDCl_3$) δ -63.1.
<u>^{15}N NMR</u> (61 MHz, $CDCl_3$) δ 393.3 (value from ^{1}H-^{15}N HMBC)
<u>HRMS</u> (APCI/QTOF) m/z: $[M + H]^+$ Calculated for $C_9H_{10}F_3N^{15}N^+$ 204.0761; Found 204.0765.
^{15}N enrichment calculated according to McNally et al (*52*) to be >99 %

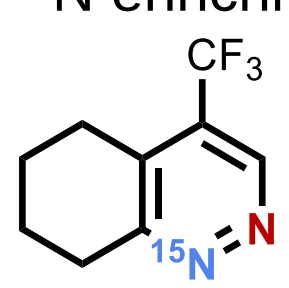

^{15}N(1)-4r

4-(Trifluoromethyl)-5,6,7,8-tetrahydrocinnoline-1-^{15}N (^{15}N(1)-4r)

Prepared according to the general procedure using 2-chloro-4-(trifluoromethyl)-5,6,7,8-tetrahydroquinoline-^{15}N **^{15}N-1r** (71 mg, 0.30 mmol, 1.0 equiv.). The crude material was purified by flash column chromatography (SiO_2, EtOAc/Hex 20 to 100 %) and then preparative TLC (50% Hexanes/EtOAc) to give the corresponding pyridazine **^{15}N(1)-4r** (27 mg, 0.13 mmol, 45 % yield) as a pale-yellow oil.
<u>^{1}H NMR</u> (600 MHz, $CDCl_3$) δ 9.12 (d, $J_{N\text{-}H}$ = 3.7 Hz, 1H, N=N-C*H*), 3.27 (t, *J* = 6.6 Hz, 2H, N=N-C-C*H*$_2$), 2.98 (t, *J* = 6.6 Hz, 2H, C*H*$_2$-C-C-CF_3), 2.01 – 1.94 (m, 2H, CH_2-C*H*$_2$-CH_2-CH_2), 1.92 – 1.85 (m, 2H, CH_2-C*H*$_2$-CH_2-CH_2).
<u>^{13}C NMR</u> (151 MHz, $CDCl_3$) δ 161.8 (d, $J_{C\text{-}N}$ = 6.6 Hz), 144.2 (app. p, $J_{C\text{-}F/C\text{-}N}$ = 5.1 Hz), 135.4, 126.8 (dq, $J_{C\text{-}N/C\text{-}F}$ = 31.9, 6.3 Hz), 123.0 (q, $J_{C\text{-}F}$ = 275.3 Hz), 30.6 (d, $J_{C\text{-}N}$ = 9.4 Hz), 24.8 (q, $J_{C\text{-}F}$ = 1.8 Hz), 21.8 (d, $J_{C\text{-}N}$ = 2.4 Hz), 21.4.
<u>^{19}F NMR</u> (565 MHz, $CDCl_3$) δ -63.1.
<u>^{15}N NMR</u> (61 MHz, $CDCl_3$) δ 408.8 (value from ^{1}H-^{15}N HMBC)
<u>HRMS</u> (APCI/QTOF) m/z: $[M + H]^+$ Calculated for $C_9H_{10}F_3N^{15}N^+$ 204.0761; Found 204.0776.
^{15}N enrichment calculated according to McNally et al (*52*) to be >99 %

Limitations

The reaction scope is limited by:

1) Oxidatively labile groups, such as thiophene etc - not compatible with the aminating reagent.
2) Ketones and aldehydes - not compatible with the aminating reagent
3) Electrophilic groups, such as alkyl halides – not compatible with azide
4) Only electron donating groups on the pyridine – not compatible with the photochemical step
5) Leaving groups in the 3-position, such as bromo or methoxy – not compatible with photochemical step
6) Electron withdrawing groups in the 3-position – not compatible with the photochemical step
7) Fused aromatic systems – not compatible with the photochemical step.

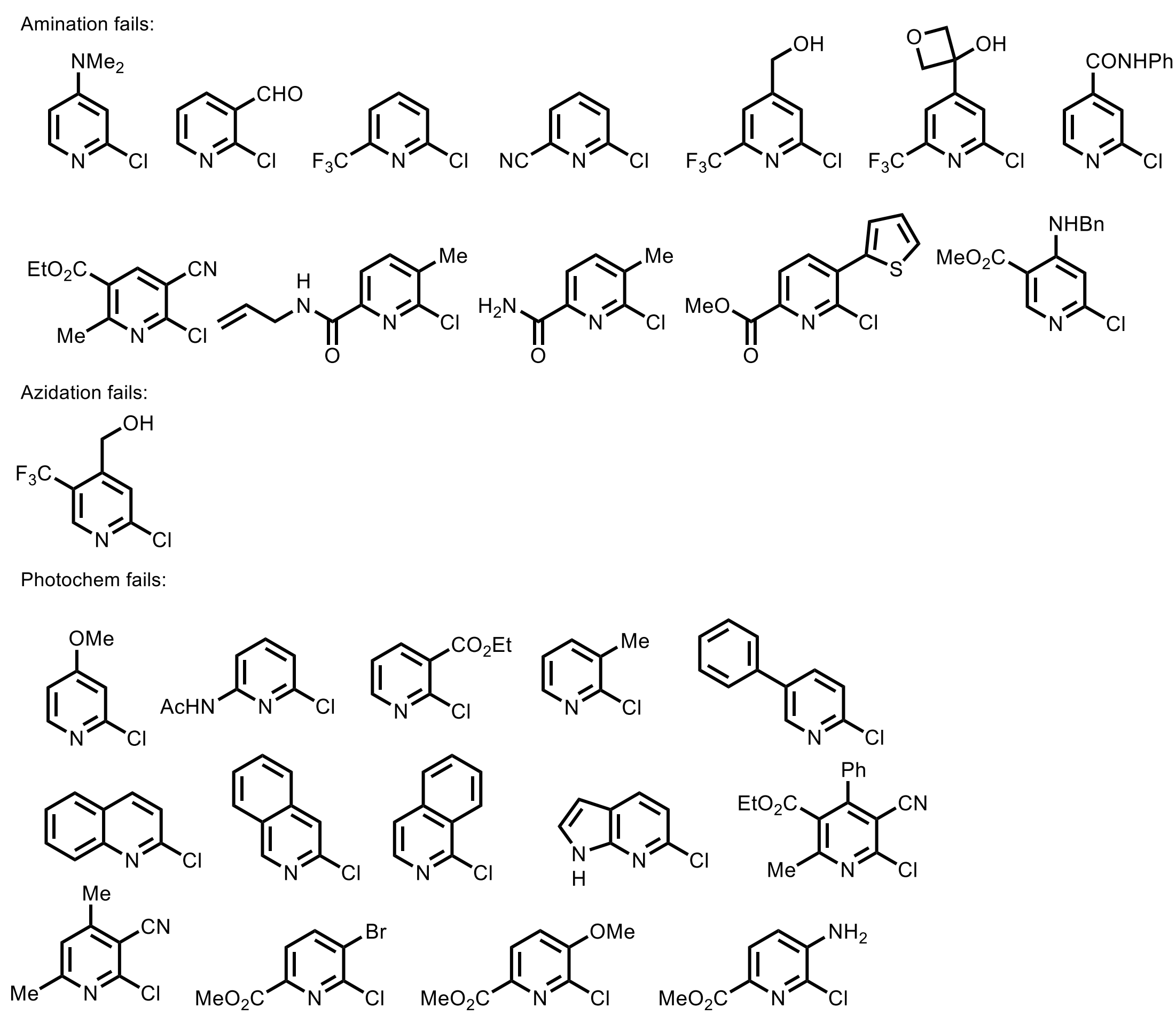

Figure S2. Limitations of the protocol.

Optimization of pyridazine synthesis

Optimization of *N*-amination:

The amination reaction was optimized on the 4-trifluoromethyl-2-chloro pyridine (Figure S3). Out of the aminating reagents tested, only reagent **2** and MSH (*32*) gave meaningful yields of the *N*-aminated product **P1**. Trifluoroethanol was found to be the optimal solvent during solvent screening, and TFA gave the highest yields as the acid additive.

Experimental procedure:

A 8 mL screw-cap vial equipped with a Teflon coated stirring bar was charged 2-chloro-4-(trifluoromethyl)pyridine (13 µL, 18 mg, 0.10 mmol, 1.0 equiv.), *tert*-butyl (((3,5-bis(trifluoromethyl)phenyl)sulfonyl)oxy)carbamate (61 mg, 0.15 mmol, 1.5 equiv.) and solvent (0.5 mL). Then, TFA (15 µL, 23 mg, 0.20 mmol, 2.0 equiv.) was added and the resulting mixture stirred for 16 hours at 40 °C. The reaction mixture was cooled down to room temperature then concentrated *in vacuo*. The crude was analyzed with ^{1}H-NMR using mesitylene (4.6 µL, 4.0 mg, 0.033 mmol, 0.33 equiv.) as an internal standard.

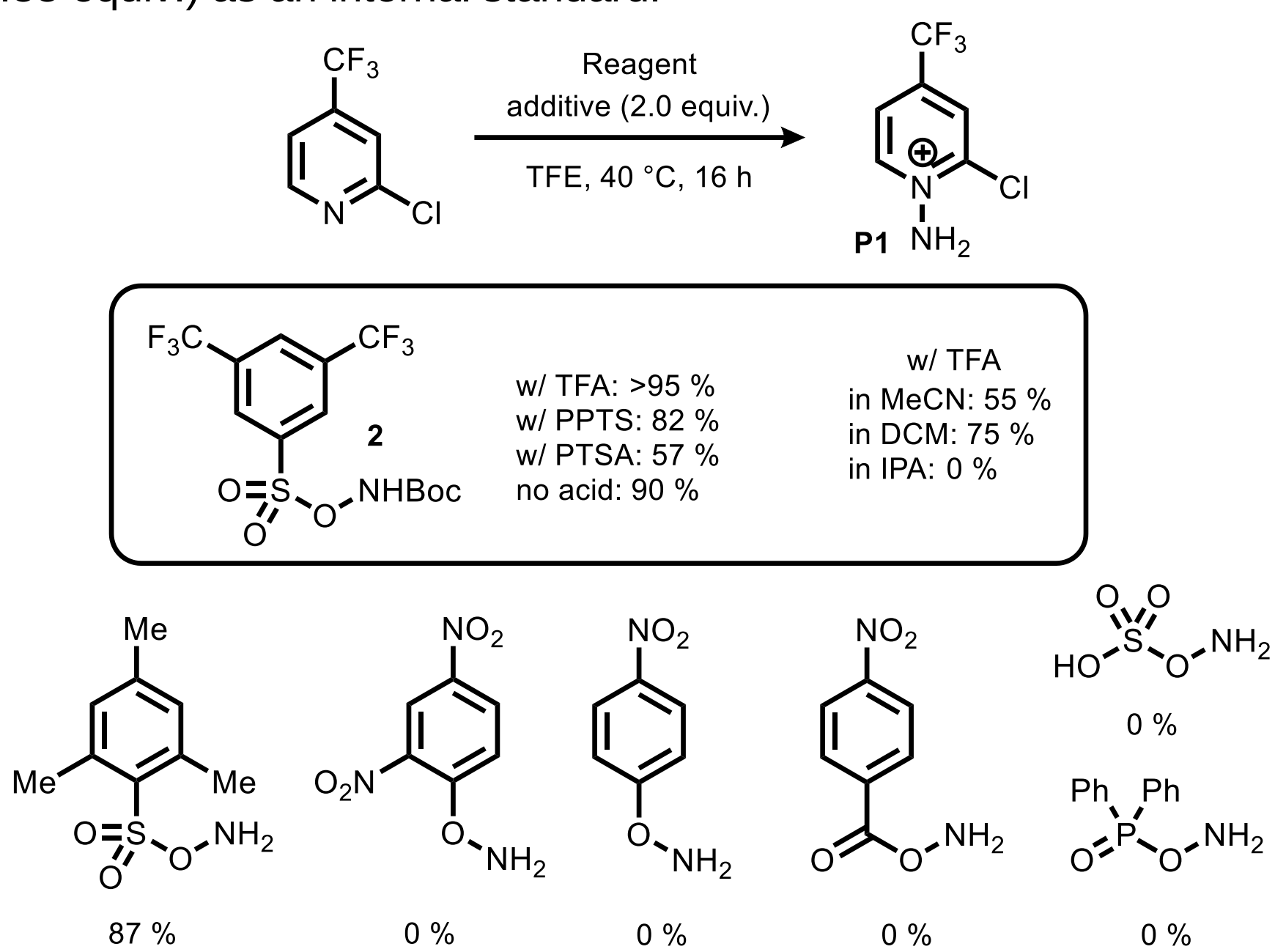

Figure S3. Optimization of the *N*-amination.

Optimization of SN_{Ar} azidation:

Initially a one-pot amination/azidation was pursued (Table S1). The highest yield of the desired 2-azido-*N*-amino pyridinium could be observed by using isopropanol as a cosolvent, likely due to the increased nucleophilicity of azide (*35*). However, in this case sodium azide was present with the TFA used in the previous step, which was deemed to dangerous and thus a solvent swap (thus removing the TFA), was pursued.

Experimental procedure:

A 20 mL screw-cap vial equipped with a Teflon coated stirring bar was charged 2-chloro-4-(trifluoromethyl)pyridine (129 µL, 182 mg, 1.00 mmol, 1.0 equiv.), *tert*-butyl (((3,5-bis(trifluoromethyl)phenyl)sulfonyl)oxy)carbamate (614 mg, 1.50 mmol, 1.5 equiv.) and TFE (5.0 mL). Then, TFA (0.15 mL, 0.23 g, 0.20 mmol, 2.0 equiv.) was added and the resulting mixture stirred for 16 hours at 40 °C. The reaction mixture was cooled down to room temperature then split into 10 equal parts, each of which were concentrated *in vacuo*. Then, NaN_3 (6.5 mg, 0.10 mmol, 1.0 equiv.) and solvent (0.5 mL) was added, and the mixture stirred for 1 h at 22 °C. The resulting mixture was concentrated *in vacuo* and the crude was analyzed with ^{1}H-NMR using mesitylene (4.6 µL, 4.0 mg, 0.033 mmol, 0.33 equiv.) as an internal standard.

Table S1. Solvent effects on the azidation reaction.

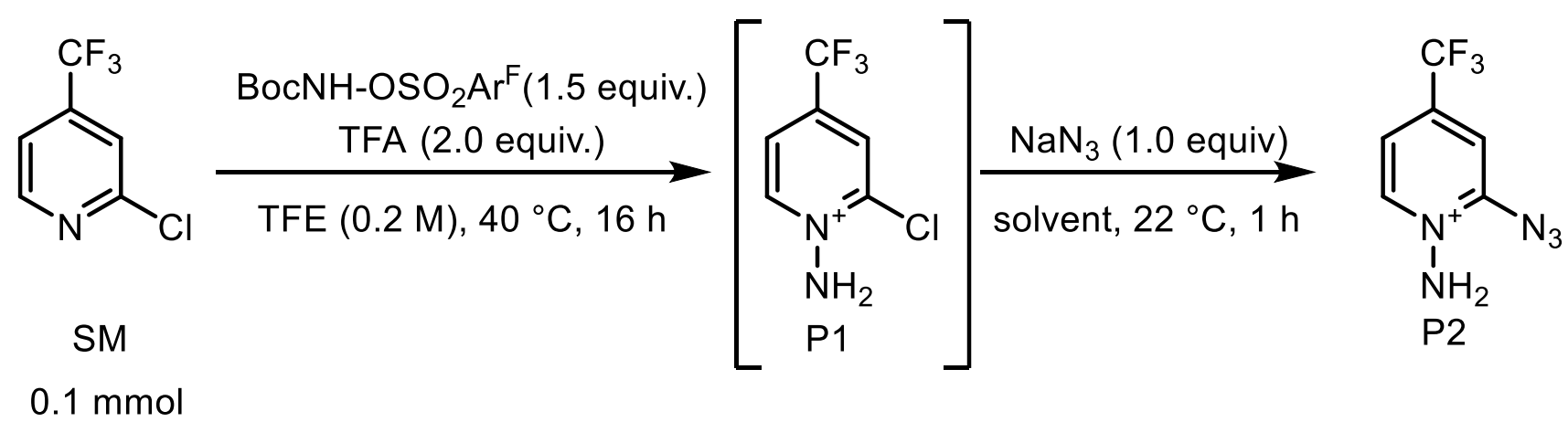

Entry	Solvent	P1, NMR yield (%)	P2, NMR yield (%)
1	Toluene	17	43
2	THF	0	61
3	MeCN	0	67
4	DME	0	68
5	IPA	0	76
6	TFE	64	7

Optimization of SN_{Ar} azidation (PPTS additive):
By solving swapping to isopropanol after the amination reaction, yields of the 2-azido-*N*-amino pyridiniums were diminished (especially for substrates other than **2a**, see Table S2). This likely originated due to the sensitivity of the intermediate **P1** to base. PPTS was carefully selected as the potential additive during the SNAr step, as pyridine is more basic than azide anion, thus limiting the generation of toxic and explosive hydrazoic acid (49). Screening of concentration and equivalents showed that running the substitution with PPTS (2.0 equiv.) in IPA (0.05 M) is optimal.
Experimental procedure:
A 20 mL screw-cap vial equipped with a Teflon coated stirring bar was charged 2-chloro-*N*,*N*-diisopropylisonicotinamide (241 mg, 1.00 mmol, 1.0 equiv.), *tert*-butyl (((3,5-bis(trifluoromethyl)phenyl)sulfonyl)oxy)carbamate (614 mg, 1.50 mmol, 1.5 equiv.) and TFE (5.0 mL). Then, TFA (0.15 mL, 0.23 g, 0.20 mmol, 2.0 equiv.) was added and the resulting mixture stirred for 16 hours at 40 °C. The reaction mixture was cooled down to room temperature then split into 10 equal parts, each of which were concentrated *in vacuo*. Then, NaN_3 (10.0 mg, 0.15 mmol, 1.5 equiv.), PPTS and IPA was added, and the mixture stirred for 1 h at 22 °C. The resulting mixture was filtered, washed with IPA (1 mL), concentrated *in vacuo* and the crude was analyzed with ^1H-NMR using mesitylene (4.6 µL, 4.0 mg, 0.033 mmol, 0.33 equiv.) as an internal standard.

Table S2. Additive effects on the azidation reaction.

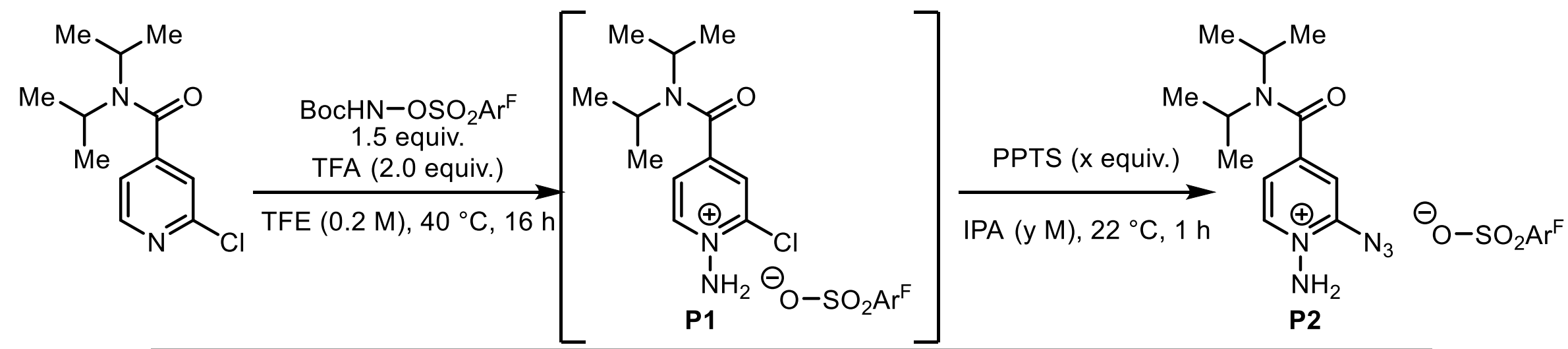

Entry	x, equiv.	y, M	P1, NMR yield (%)	P2, NMR yield (%)
1	1	0.20	0	77
2	2	0.20	0	79
3	4	0.20	34	44
4	6	0.20	24	59
5	2	0.10	8	75
6	2	0.05	0	82

Optimization of photochemistry:

Discussion:

Photocatalyst screening of the photochemical rearrangement revealed that benzophenone accelerates the reaction, and that using 390 nm Kessil LED is the highest wavelength light that still promotes the reaction (Figure S4). In addition, MeCN provided the highest yields of the pyridazine **4a**. Other triplet sensitizers were tested, and a CF_3/OMe-modified benzophenone (*53*) provided slightly faster reaction, but at the cost of added complexity of the photocatalyst. Acetophenones accelerated the reaction slightly, but the low absorption at 390 nm presumably impedes their utility in this reaction. Other tested photocatalysts did not provide higher yields of the desired product.

Experimental procedure:

A 20 mL screw-cap vial equipped with a Teflon coated stirring bar was charged 2-chloro-4-(trifluoromethyl)pyridine (129 µL, 182 mg, 1.00 mmol, 1.0 equiv.), *tert*-butyl (((3,5-bis(trifluoromethyl)phenyl)sulfonyl)oxy)carbamate (614 mg, 1.50 mmol, 1.5 equiv.) and TFE (5.0 mL). Then, TFA (0.15 mL, 0.23 g, 0.20 mmol, 2.0 equiv.) was added and the resulting mixture stirred for 16 hours at 40 °C. The reaction mixture was cooled down to room temperature and concentrated *in vacuo*. Then PPTS (0.50 g, 2.0 mmol, 2.0 equiv.), IPA (20 mL) and NaN_3 (100 mg) were added. The reaction mixture was stirred at 22 °C for 1 hour. Then, the mixture was filtered through a glass filter, the residue washed with IPA (5 mL), and the filtrate concentrated *in vacuo*. The mixture was split into 20 equal parts via MeCN solution. Then, photocatalyst (0.2 equiv.) and solvent (2 mL) were added, and the mixture irradiated with 390 nm Kessil LED lamps for 1 h. The resulting mixture was sparged with N_2, concentrated *in vacuo* and the crude was analyzed with ^{1}H-NMR using mesitylene (4.6 µL, 4.0 mg, 0.033 mmol, 0.167 equiv.) as an internal standard.

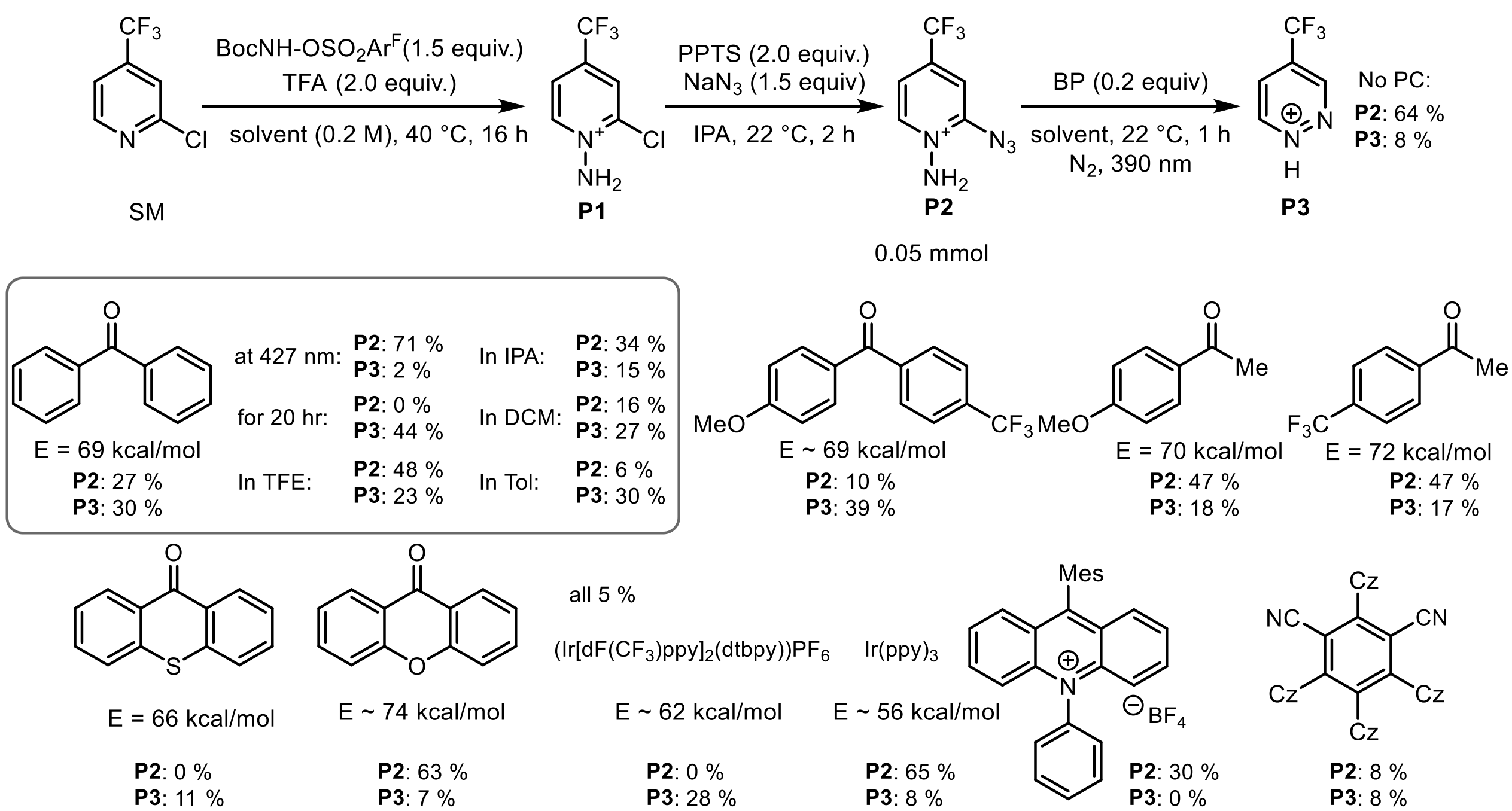

Figure S4. Optimization of the photorearrangement.

Mechanistic studies

1-Amino-2-azido-4-(trifluoromethyl)pyridin-1-ium 3,5-bis(trifluoromethyl)benzenesulfonate (3a×OSO_2Ar^F)

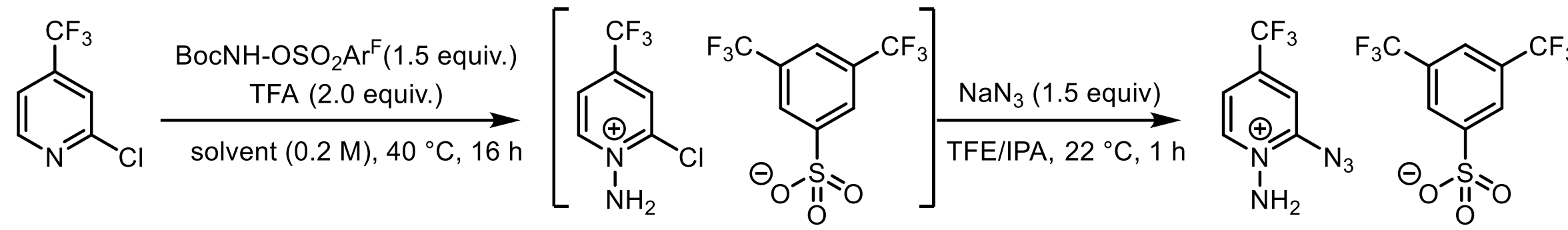

3a•OSO_2Ar^F

Scheme S1. Synthesis of 2-azido-N-amino pyridinium sulfonate **3a×OSO_2Ar^F**.

A 20 mL screw-cap vial equipped with a Teflon coated stirring bar was charged 2-chloro-4-(trifluoromethyl)pyridine (129 µL, 182 mg, 1.00 mmol, 1.0 equiv.), *tert*-butyl (((3,5-bis(trifluoromethyl)phenyl)sulfonyl)oxy)carbamate (614 mg, 1.50 mmol, 1.5 equiv.) and TFE (5.0 mL). Then, TFA (0.15 mL, 0.23 g, 0.20 mmol, 2.0 equiv.) was added and the resulting mixture stirred for 16 hours at 40 °C. The reaction mixture was cooled down to room temperature, and IPA (15 mL) and NaN_3 (100 mg) were added. The reaction mixture was stirred at 22 °C for 1 hour. Then, the mixture was filtered through a glass filter, the residue washed with IPA (5 mL), and the filtrate concentrated *in vacuo*. The crude was dissolved in DCM (20 mL), filtered (filter cake washed with additional DCM (5 mL)). The filtrate was concentrated to ca. 10 mL, placed in a -20 °C freezer for 16 hours. Then, the resulting suspension was filtered and washed with cold DCM (2×5mL), to provide the salt **3a×OSO_2Ar^F** (202 mg, 0.41 mmol, 41 %).

^{1}H NMR (500 MHz, CD_3CN) δ 8.84 (d, *J* = 6.9 Hz, 1H, N-C*H*), 8.23 (s, 2H, 2×ArH), 8.09 (d, *J* = 2.0 Hz, 1H, N-C(N_3)-C*H*), 7.99 (s, 1H, Ar*H*), 7.74 (dd, *J* = 6.9, 2.1 Hz, 1H, Ar*H*), 7.57 (br. s, 2H, NH_2). (note: chemical shifts are concentration dependent)
^{19}F NMR (470 MHz, CD_3CN) δ -63.4 (6F, Ar(CF_3)$_2$), -65.5 (s, 3F, HetArCF_3).
^{13}C{^{1}H} NMR (126 MHz, CD_3CN) δ 151.1, 148.2, 141.2, 140.9 (q, $J_{C\text{-}F}$ = 36.3 Hz), 132.0 (q, $J_{C\text{-}F}$ = 33.4 Hz), 127.2 (q, $J_{C\text{-}F}$ = 4.0 Hz), 124.4 (q, $J_{C\text{-}F}$ = 272.1 Hz, 2C), 124.1 – 123.5 (m), 118.8 (q, $J_{C\text{-}F}$ = 3.5 Hz), 117.6 (q, $J_{C\text{-}F}$ = 3.8 Hz).
HRMS (CI/QTOF) m/z: $[M]^+$ Calculated for $C_6H_5F_3N_5^+$ 204.0492; Found 204.0482; $[M]^-$ Calculated for $C_8H_3F_6O_3S^-$ 292.9713; Found 292.9712;
X-ray crystallography: Crystals suitable for single crystal X-ray crystallography were grown through cooling down a saturated solution of the compound in CH_2Cl_2. The crystal structure is deposited in the Cambridge Crystallographic Data Centre CCDC 2431454.

Thermal stability of salt **3a×OSO$_2$Ar^F** was evaluated with TGA (10 °C/min, Figure S5) and DSC analysis (2.5 °C/min, Figure S6), indicating initial melting and then decomposition at ~ 140 °C.

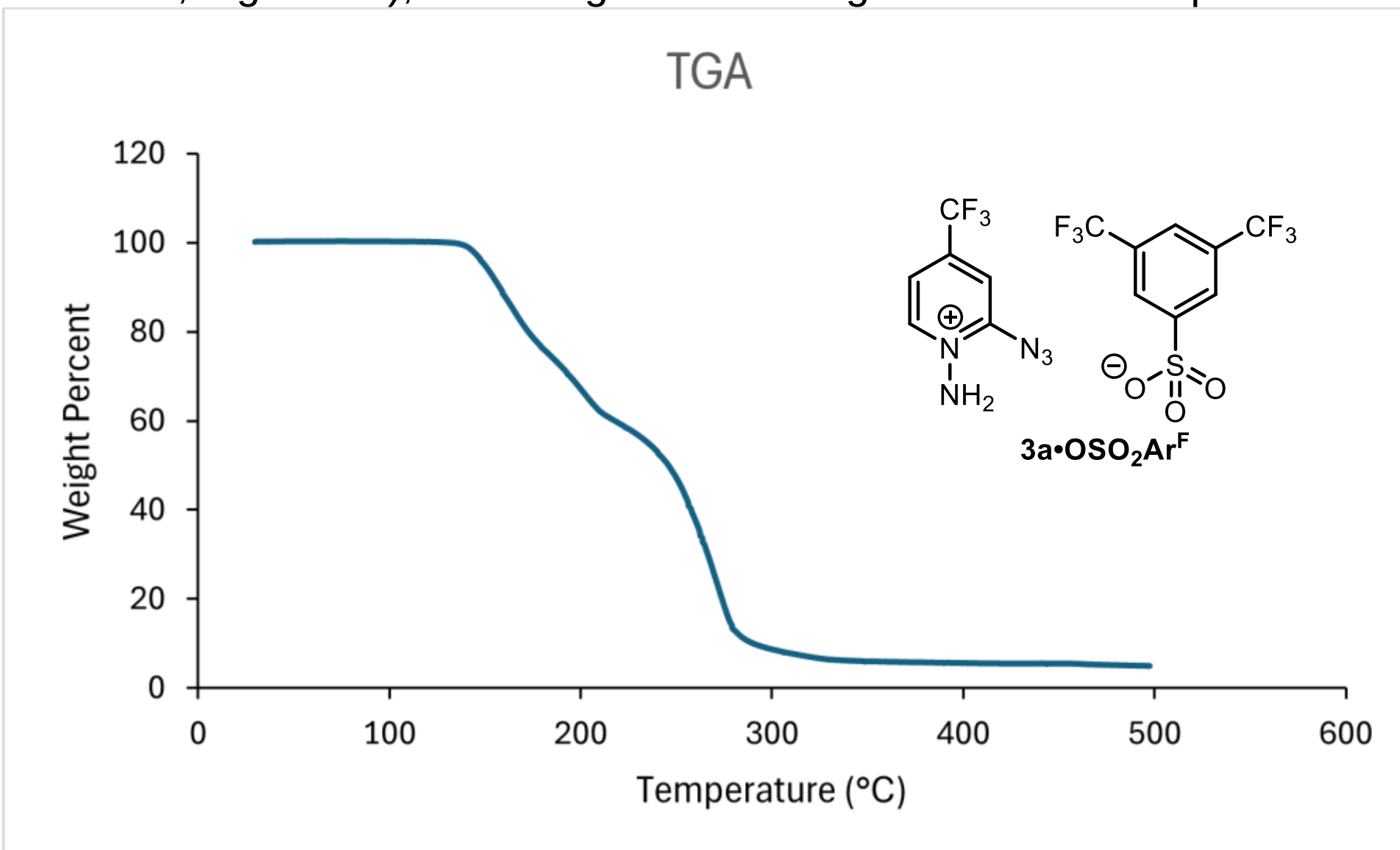

Figure S5. TGA analysis of 3a×OSO$_2$Ar^F.

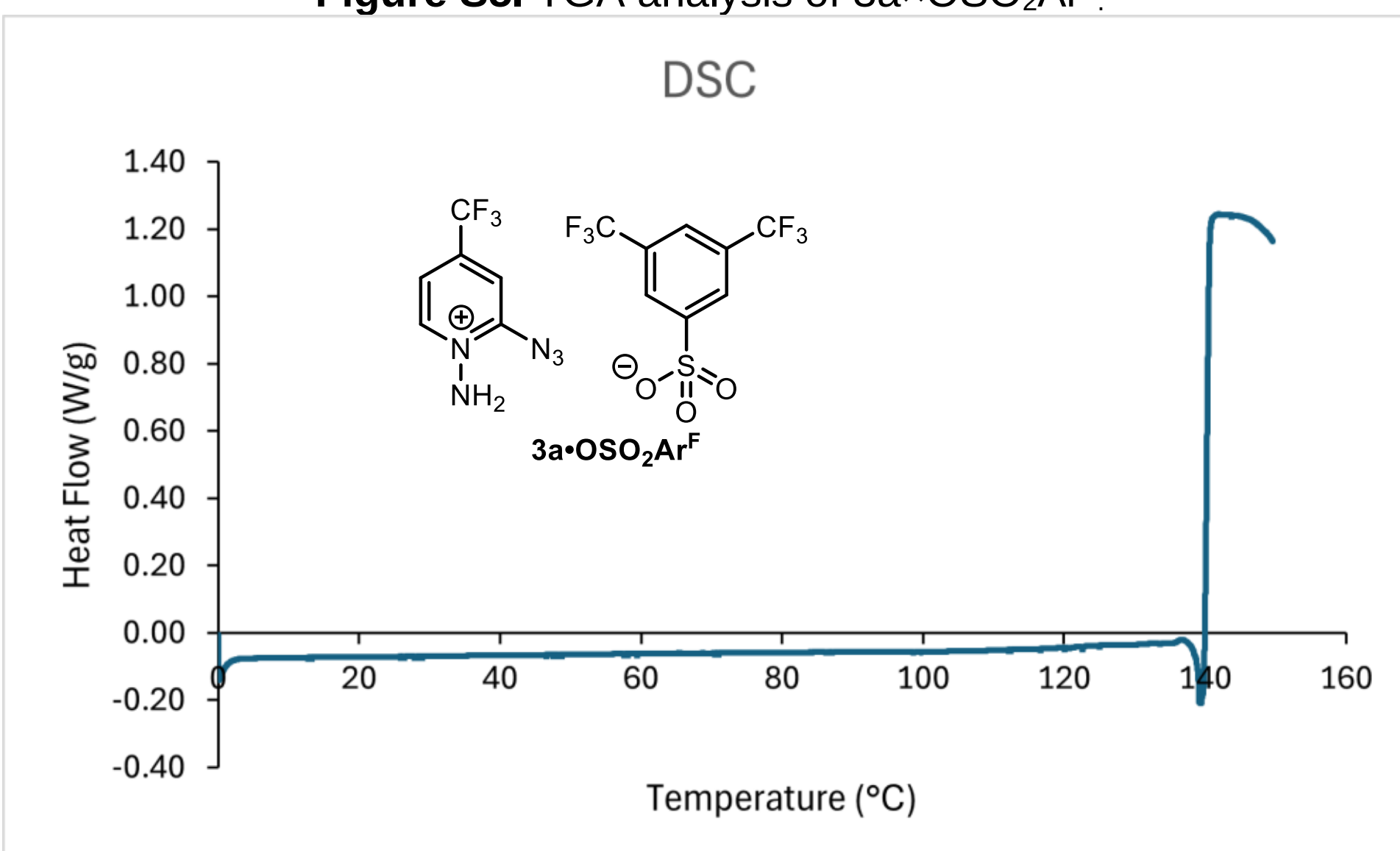

Figure S6. DSC analysis of 3a×OSO$_2$Ar^F.

1-Amino-2-azido-4-(trifluoromethyl)pyridin-1-ium trifluoromethanesulfonate 3a×OTf

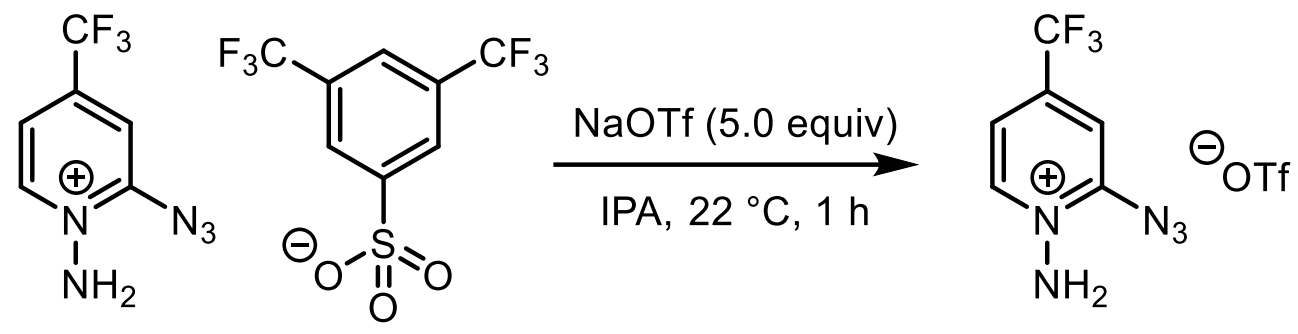

Scheme S2. Synthesis of 2-azido-N-amino pyridinium triflate **3a×OTf**.

A 20 mL screw-cap vial equipped with a Teflon coated stirring bar was charged with 1-amino-2-azido-4-(trifluoromethyl)pyridin-1-ium 3,5-bis(trifluoromethyl)benzenesulfonate (**3a×OSO₂Ar^F**, 50 mg. 0.10 mmol, 1.0 equiv.) and NaOTf (86 mg, 0.50 mmol, 5.0 equiv.) and IPA (1.0 mL). The resulting mixture was stirred at 22 °C for 1 hour, then filtered and washed with IPA (3 mL). The residue was concentrated *in vacuo*, dissolved in MeCN (2 mL), filtered, and concentrated *in vacuo* again. The residue was dissolved again in minimal amount of MeCN (ca. 0.1 mL), and then DCM (5 mL) was rapidly added to induce precipitation. The resulting mixture was filtered and washed with DCM (1 mL). The precipitation procedure was repeated 3 times. The precipitate was then dissolved in DCM (1 mL) containing minimal amount of MeOH (ca 0.2 mL), and layered with hexanes (1.0 mL), and then placed in a -20 °C freezer for 16 hours. Then, the resulting suspension was filtered and washed with cold DCM (2×2 mL), to provide the salt **3a×OTf** (11 mg, 0.031 mmol, 31 %).

^{1}H NMR (500 MHz, CD_3CN) δ 8.57 (d, *J* = 6.9 Hz, 1H, N-C*H*), 8.16 (s, 1H, N-CCl-C*H*), 7.80 (d, *J* = 6.8 Hz, 1H, N-CH-C*H*), 7.01 (br. s, 2H, NH_2).
^{19}F NMR (471 MHz, CD_3CN) δ -65.5 (s, 3F), -79.4 (s, 3F).
^{13}C{^{1}H} NMR (126 MHz, CD_3CN) δ 148.6, 141.3 (q, J_{C-F} = 36.5 Hz), 141.3, 122.1 (q, J_{C-F} = 274.0 Hz), 121.9 (q, J_{C-F} = 320.2 Hz), 118.9 (q, J_{C-F} = 3.3 Hz), 117.7 (q, J_{C-F} = 3.9 Hz).
HRMS (CI/QTOF) m/z: $[M]^+$ Calculated for $C_6H_5F_3N_5^+$ 204.0492; Found 204.0484; $[M\ ]^-$ Calculated for $CF_3O_3S^-$ 148.9526; Found 148.9522;

Tetra-butyl ammonium 3,5-bis(trifluoromethyl)benzenesulfonate (S1)

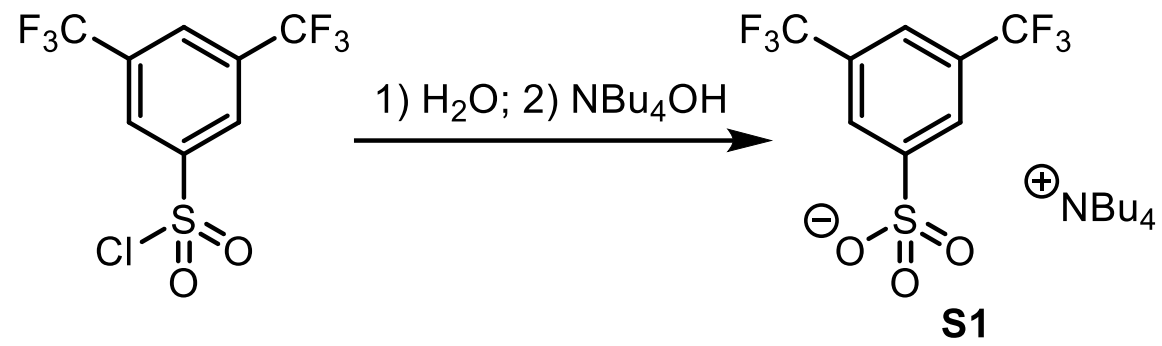

Scheme S3. Synthesis of tetra-butyl ammonium sulfonate **S1**.

According to a reported procedure,(*54*) a 100 mL round-bottom flask equipped with a Teflon coated stirring bar was charged with 3,5-bis(trifluoromethyl)benzenesulfonic chloride (1.0 g, 3.2 mmol, 1.0 equiv.) and water (5.0 mL). The resulting mixture stirred for 16 hours at 105 °C. Then, the water was distilled off at atmospheric pressure, and the residue dried *in vacuo*. The crude was dissolved in MeOH (20 mL), and tetra-butyl ammonium hydroxide (1.5 g, 1.5 mL, 3.2 mmol, 1.0 equiv., 55 wt% mass purity) was slowly added. The mixture was concentrated *in vacuo* and then recrystallized from water/EtOH to give the salt **S1** (0.41 g, 0.77 mmol, 24 %) as a white solid.

^{1}H NMR (400 MHz, $CDCl_3$) δ 8.40 (s, 2H, Ar*H*), 7.81 (s, 1H, Ar*H*), 3.41 – 3.08 (m, 8H, 4×N-CH_2), 1.77 – 1.52 (m, 8H, N-CH_2-CH_2), 1.44 (app. hex, *J* = 7.4 Hz, 8H, N-CH_2-CH_2-CH_2), 1.00 (t, *J* = 7.3 Hz, 12H, N-CH_2-CH_2-CH_2-CH_3).

^{19}F NMR (376 MHz, $CDCl_3$) δ -62.8 (s, 6F, 2×CF_3).

Spectral data matched those reported in the literature (*54*).

2-phenyl-2-(phenylamino)acetonitrile (5)

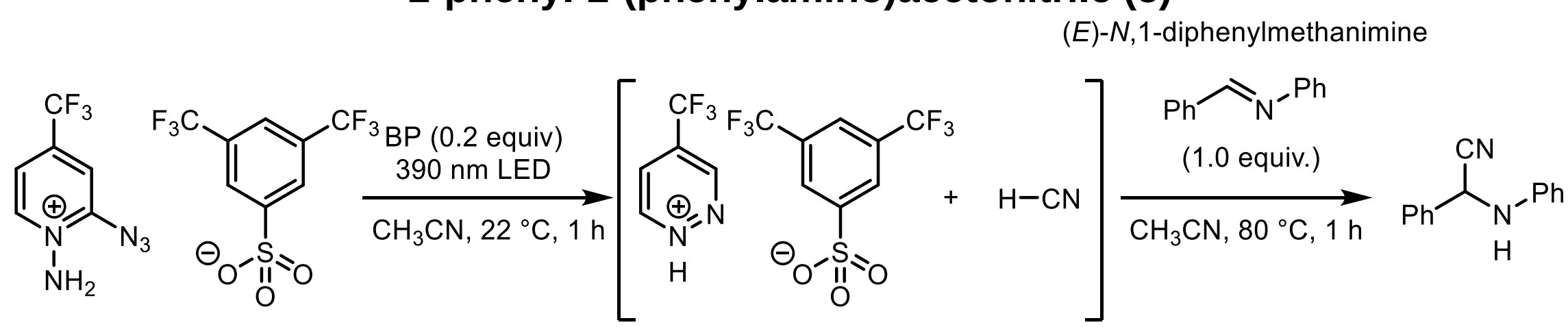

Scheme S4. Strecker trapping experiment.

A 8 mL screw-cap vial equipped with a Teflon coated stirring bar was charged with 1-amino-2-azido-4-(trifluoromethyl)pyridin-1-ium 3,5-bis(trifluoromethyl)benzenesulfonate (**3a×OSO₂Ar^F**, 50 mg, 0.10 mmol, 1.0 equiv.), benzophenone (3.6 mg, 0.020 mmol, 0.20 equiv.) and CH_3CN (2.0 mL). The resulting mixture was sparged with N_2 for 1 minute and then irradiated with 390 nm Kessil LED for 1 hour. Then, the vial was uncapped and N-benzylidene aniline (18 mg, 0.10 mmol, 1.0 equiv.) was quickly added. The resulting mixture was then stirred at 80 °C for 1 hour. After cooling down, the mixture was concentrated in vacuo, and the crude was purified via preparative TLC (50 % EtOAc/Hexanes) to give the Strecker adduct **5** (9 mg, 0.04 mmol, 43 %) as a colorless solid.

^{1}H NMR (400 MHz, $CDCl_3$) δ 7.64 – 7.58 (m, 2H, Ar*H*), 7.51 – 7.41 (m, 3H, Ar*H*), 7.31 – 7.26 (m, 2H, Ar*H*), 6.97 – 6.86 (m, 1H, Ar*H*), 6.85 – 6.69 (m, 2H, Ar*H*), 5.44 (d, *J* = 8.4 Hz, 1H, C*H*), 4.02 (d, *J* = 8.5 Hz, 1H, N*H*).

^{13}C{^{1}H} NMR (101 MHz, $CDCl_3$) δ 144.8, 134.1, 129.7, 129.7, 129.5, 127.4, 120.5, 118.3, 114.3, 50.4.

HRMS (CI/QTOF) m/z: $[M + H]^+$ Calculated for $C_{14}H_{13}N_2^+$ 209.1073; Found 209.1079.

Spectral data matched those reported in the literature (*42*).

4-(trifluoromethyl)pyridazine-3-carbonitrile (4a-CN)

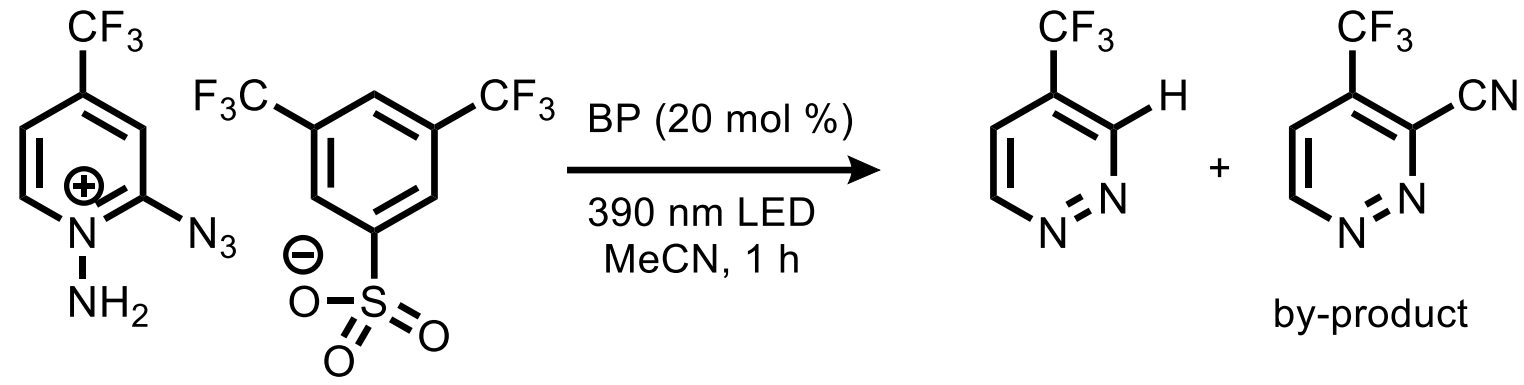

Scheme S5. Isolation of cyano-pyridazine byproduct.

A 8 mL screw-cap vial equipped with a Teflon coated stirring bar was charged with 1-amino-2-azido-4-(trifluoromethyl)pyridin-1-ium 3,5-bis(trifluoromethyl)benzenesulfonate (**3a×OSO$_2$Ar^F**, 150 mg, 0.30 mmol, 1.0 equiv.), benzophenone (11 mg, 0.060 mmol, 0.20 equiv.) and CH_3CN (6.0 mL). The resulting mixture was then irradiated with 390 nm Kessil LED for 1 hour. Then, the mixture was concentrated *in vacuo*, and the crude was filtered through a pad if silica eluting with DCM. The filtrate was concentrated in vacuo and purified via preparative TLC (20 % EtOAc/Hexanes) to give the cyano pyridazine **4a-CN** (1 mg, 0.006 mmol, 2 %) as a colorless solid.

^{1}H NMR (500 MHz, $CDCl_3$) δ 9.63 (d, J = 5.4 Hz, 1H), 7.90 (d, J = 5.4 Hz, 1H).

^{19}F NMR (470 MHz, $CDCl_3$) δ -64.2.

Spectral data matched those reported in the literature (*55*).

2-Imino-4-(trifluoromethyl)pyridin-1(2H)-amine (3a-NH$_2$)

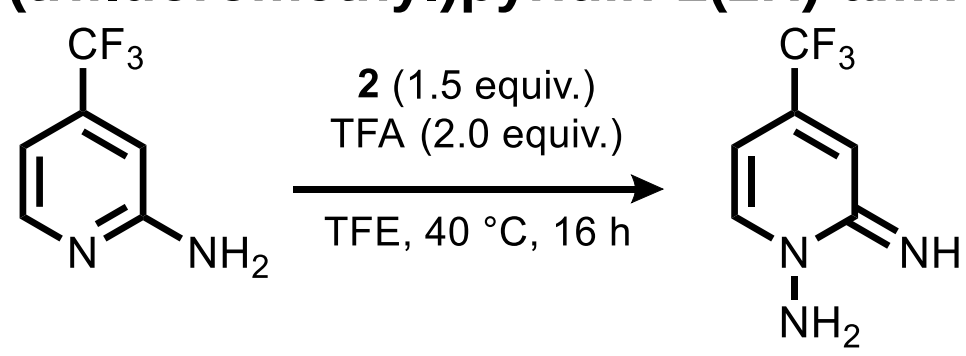

Scheme S6. Synthesis of aminated byproduct **3a-NH$_2$**.

A 20 mL screw-cap vial equipped with a Teflon coated stirring bar was charged 2-amino-4-(trifluoromethyl)pyridine (162 mg, 1.00 mmol, 1.0 equiv.), *tert*-butyl (((3,5-bis(trifluoromethyl)phenyl)sulfonyl)oxy)carbamate (614 mg, 1.50 mmol, 1.5 equiv.) and TFE (5.0 mL). Then, TFA (0.15 mL, 0.23 g, 0.20 mmol, 2.0 equiv.) was added and the resulting mixture stirred for 16 hours at 40 °C. The reaction mixture was cooled down to room temperature and concentrated in vacuo. Then, diethyl ether (15 mL) and hexanes (5 mL) was added to induce precipitation. The precipitate was filtered and then washed with hexanes (2×5 mL), after which it was partitioned between DCM (20 mL) and aq. NaOH (1M, 20 mL). The organic layer was washed with brine (20 mL), dried over Na_2SO_4 and concentrated *in vacuo* to give the aminated pyridine **3a-NH$_2$** (100 mg, 0.565 mmol, 56 %) as a white solid.

^{1}H NMR (500 MHz, CD_3CN) δ 8.15 (d, J = 5.3 Hz, 1H, HetAr*H*), 6.79 (dd, J = 5.3, 1.6 Hz, 1H, HetAr*H*), 6.73 (d, J = 1.6, 1H), 5.25 (br. s, 2H, N*H*$_2$).

^{19}F NMR (470 MHz, CD_3CN) δ -65.94.

^{13}C{^{1}H} NMR (126 MHz, CD_3CN) δ 160.9, 150.7, 139.8 (q, $J_{C\text{-}F}$ = 33.0 Hz), 124.3 (q, $J_{C\text{-}F}$ = 271.8 Hz), 108.7 (q, $J_{C\text{-}F}$ = 3.4 Hz), 104.5 (q, $J_{C\text{-}F}$ = 4.2 Hz).

HRMS (CI/QTOF) m/z: [M]$^+$ Calculated for $C_6H_7F_3N_3^+$ 178.0587; Found 178.0588.

Spectroscopic studies

All concentrations 5.0×10^{-4}M.

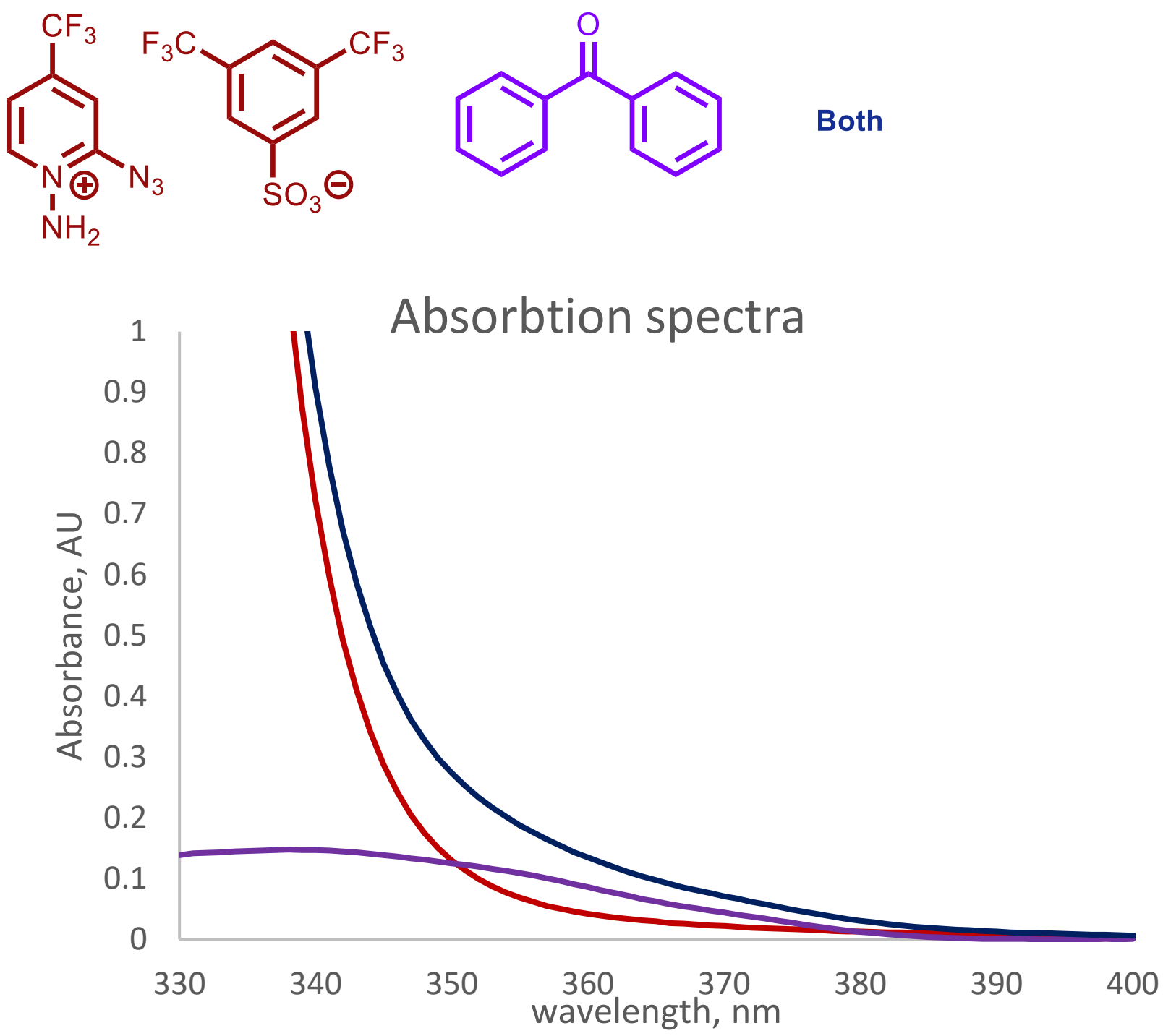

Figure S7. UV-absorption studies.

All concentrations 5.0×10^{-4}M.

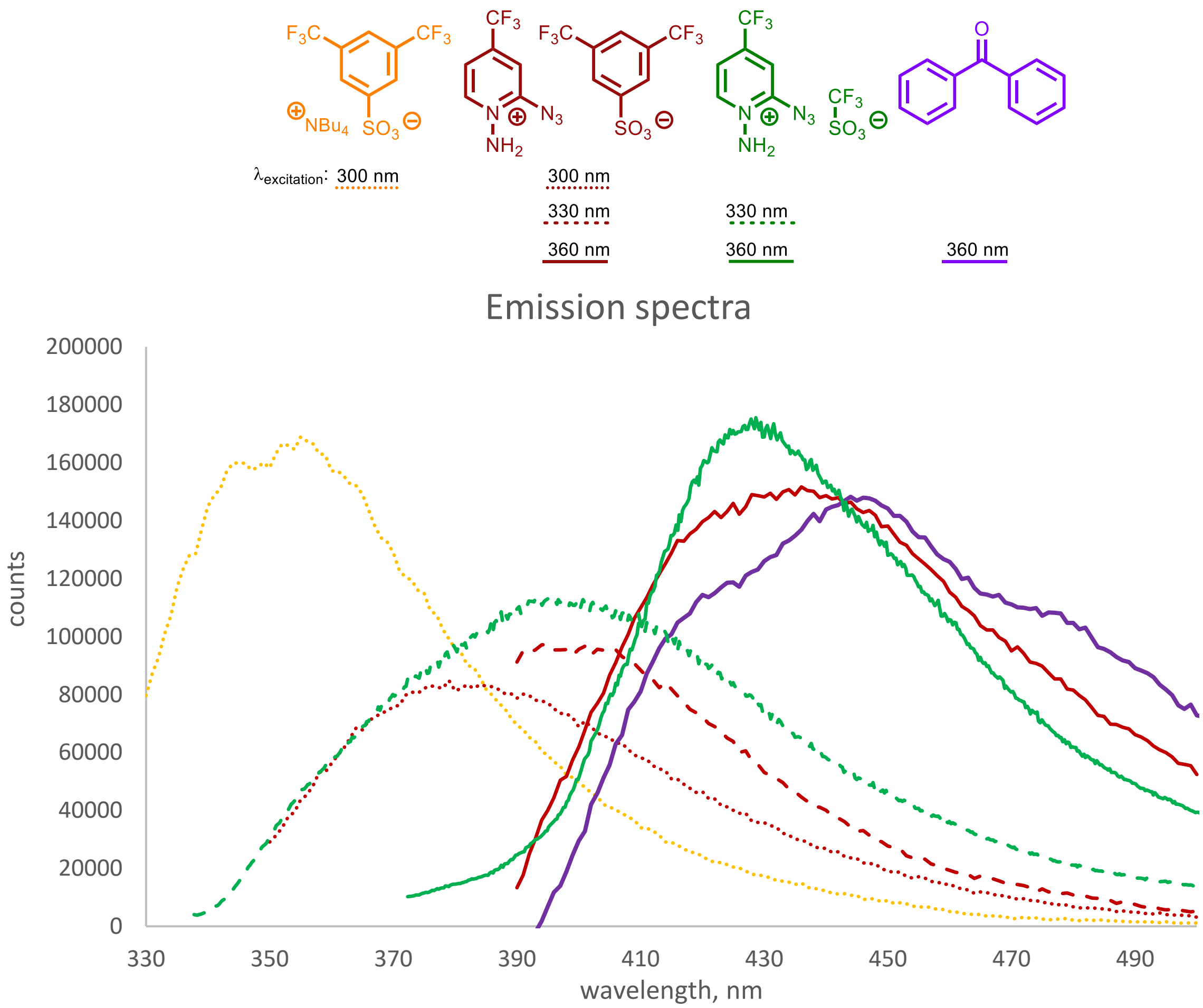

Figure S8. UV-Vis emission studies.

NMR studies

A 5 mm NMR tube was charged with 1-amino-2-azido-4-(trifluoromethyl)pyridin-1-ium 3,5-bis(trifluoromethyl)benzenesulfonate (**3a×OSO$_2$Ar^F**, 15 mg. 0.030 mmol, 1.0 equiv.) and CD_3CN (0.6 mL). The resulting solution was irradiated with 390 nm Kessil LED, taking NMR spectra every 5 minutes. The residual CHD_2CN signal was used as an internal standard.

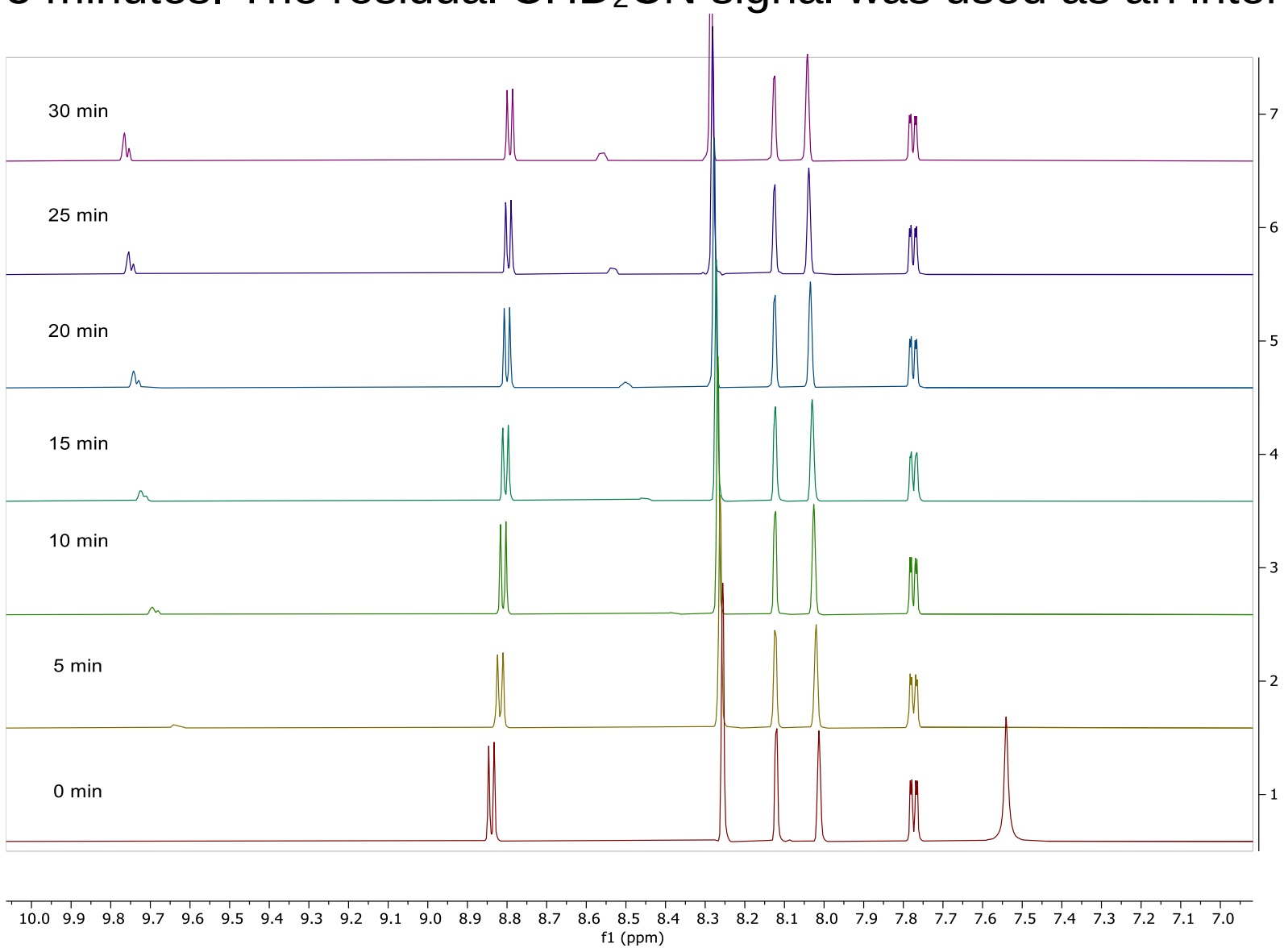

Figure S9. ^{1}H NMR (500 MHz, CD_3CN): Product evolution over time without photocatalyst.

The same procedure was repeated with benzophenone (20 mol%) as the photocatalyst.

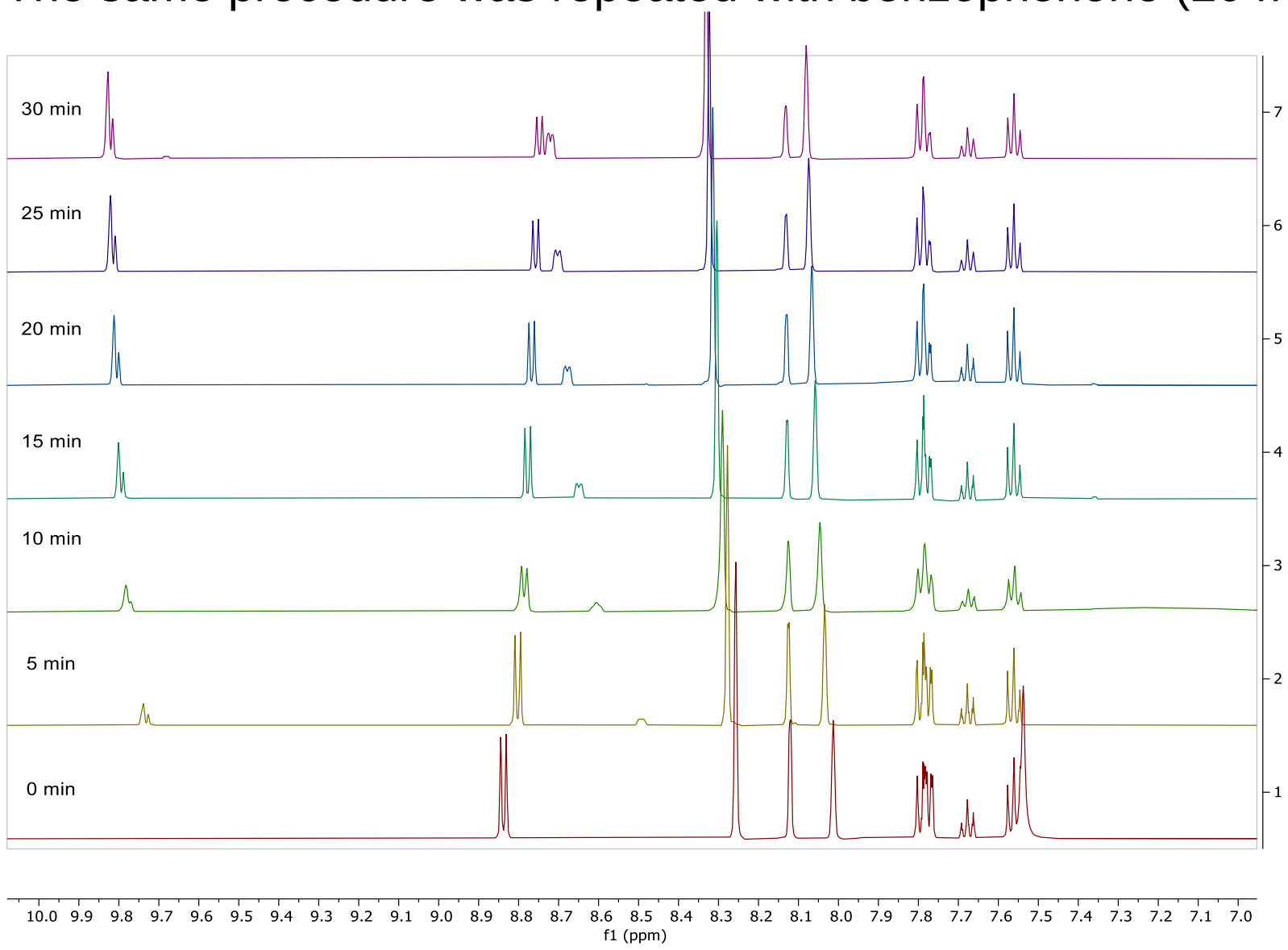

Figure S10. ^{1}H NMR (500 MHz, CD_3CN): Product evolution over time with photocatalyst.

Discussion:

Irradiation of **$3a\times OSO_2Ar^F$** was conducted with and without the benzophenone, and the observable side products are formed in similar distributions as seen in Figure S11. By-product **4a-CN** likely originates from oxidation of intermediate **IV** as opposed to cyanide extrusion. By-product byproduct **$3a\text{-}NH_2$** likely originates from a competitive reduction of intermediate **I**[3].

Experimental procedure:

A 8 mL screw-cap vial equipped with a Teflon coated magnetic stir-bar was charged with 1-amino-2-azido-4-(trifluoromethyl)pyridin-1-ium 3,5-bis(trifluoromethyl)benzenesulfonate (**$3a\times OSO_2Ar^F$**, 15 mg, 0.030 mmol, 1.0 equiv.) and CD_3CN (0.6 mL). The resulting solution then sparged with N_2 for 1 minute and was irradiated with 390 nm Kessil LED for 1 hour. The same procedure was repeated with benzophenone (20 mol%) as the photocatalyst.

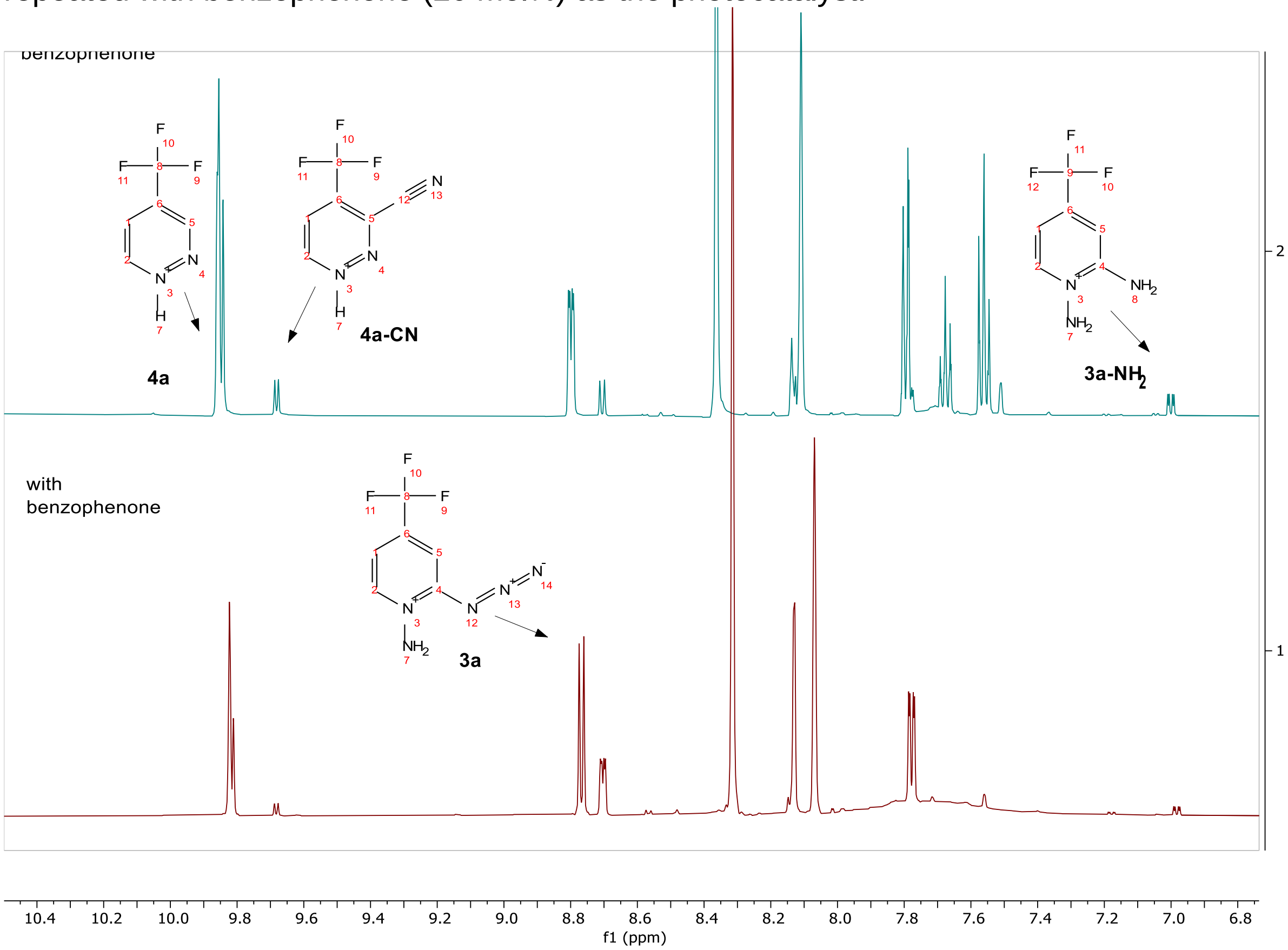

Figure S11. ^{1}H NMR (500 MHz, CD_3CN): comparison of impurity profile with and without photocatalyst.

Synthesis of starting materials

***Tert*-butyl (((3,5-bis(trifluoromethyl)phenyl)sulfonyl)oxy)carbamate (2)**

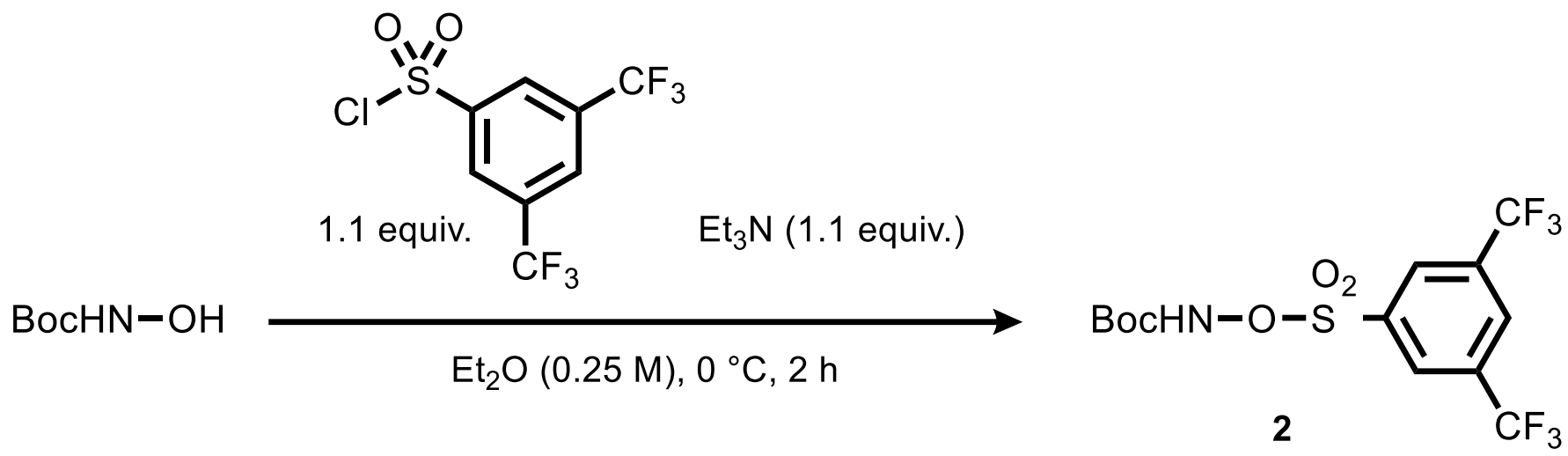

Figure S12. Synthesis of aminating reagent **2**.

According to the reported procedure (*34*), a 250 mL round bottom flask equipped with a Teflon ocated magnetic stirring bar was charged tert-butyl hydroxycarbamate (3.3 g, 25 mmol, 1.0 equiv.), 3,5-bis(trifluoromethyl)benzene sulfonyl chloride (8.6 g, 27.5 mmol, 1.1 equiv.) and Et_2O (200 mL). The flask was stirred at 0 °C for 10 minutes, after which Et_3N (3.9 mL, 2.8 g, 27.5 mmol, 1.1 equiv.) was added, immediately forming a white precipitate. The resulting mixture was stirred at 0 °C for 2 hours, then filtered (filter cake washed with Et_2O (2×50 mL). The filtrate was concentrated in vacuo. Pentane (~200 mL) was added to the crude mixture to induce precipitation, and the mixture stirred vigorously at 0 °C for 1 hour. The suspension was filtered and washed with pentane (3×50 mL) and dried on the frit to give the aminating reagent **2** (6.73 g, 16.5 mmol, 66 %) as a white solid.

^{1}H NMR (500 MHz, $CDCl_3$) δ 8.46 (s, 2H, Ar*H*), 8.19 (s, 1H, C Ar*H*), 7.81 (s, 1H, N*H*), 1.29 (s, 9H, $C(CH_3)_3$).
^{13}C{^{1}H} NMR (151 MHz, $CDCl_3$) δ 153.7, 136.7, 133.2 (q, J_{C-F} = 34.9 Hz), 130.0 (q, J_{C-F} = 3.7 Hz), 128.1-128.3 (m), 122.4 (q, J_{C-F} = 273.5 Hz), 85.1, 27.7.
^{19}F NMR (377 MHz, $CDCl_3$) δ – 63.0 (s, 6F, 2×CF_3)
Spectral data matched those reported in the literature (*34*).

Note: DSC data show decomposition onset at around 60 °C. The reagent should be stored in the fridge (4 °C).

***Tert*-butyl (((3,5-bis(trifluoromethyl)phenyl)sulfonyl)oxy)carbamate-^{15}N (^{15}N-2)**

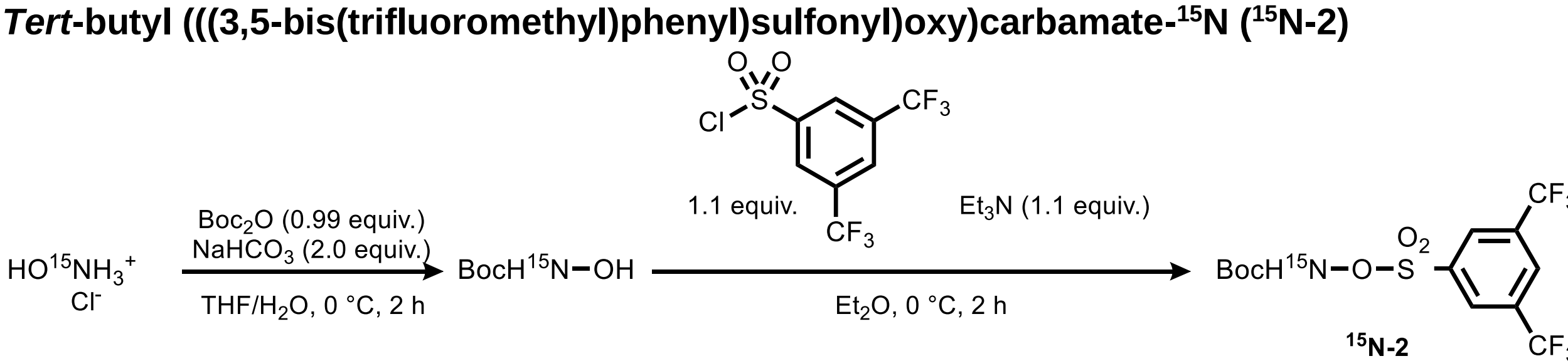

Figure S13. Synthesis of labbelled aminating reagent **^{15}N-2**.

According to the reported procedure (*34*), a 50 mL round bottom flask equipped with a Teflon coated magnetic stir bar was charged with hydroxylamine^{15}N hydrochloride (493 mg, 7.00 mmol, 1.0 equiv.), Boc_2O (1.51 g, 6.93 mmol, 0.99 equiv.), THF (7 mL) and water (7 mL). The reaction mixture was cooled to 0 °C and $NaHCO_3$ (1.18 g, 14.0 mmol, 2.0 equiv.) was added. The resulting mixture was stirred at 0 °C for 2 hours, and then the volatiles were removed by a stream of compressed N_2. The resulting mixture was extracted with EtOAc (3×20 mL). The combined organic extracts were washed with sat. aq. $NaHCO_3$ (20 mL), brine (20 mL), dried over Na_2SO_4,

filtered and concentrated *in vacuo*. To the resulting crude product was added 3,5-bis(trifluoromethyl)benzene sulfonyl chloride (2.2 g, 7.0 mmol, 1.0 equiv.) and Et_2O (56 mL). The mixture was cooled down to 0 °C, and then Et_3N (1.1 mL, 7.7 mmol, 1.1 equiv.) was added. The resulting mixture was stirred at 0 °C for 2 hours, filtered (filter cake washed with Et_2O (2×30 mL)) and concentrated *in vacuo*. The crude residue was loaded on a Buchner funnel containing SiO_2 (30 g). The funnel was eluted with 0-2 % EtOAc in hexanes until a yellow impurity was removed, followed by EtOAc to recover the aminating reagent. The EtOAc fraction was concentrated in vacuo, triturated with pentane and dried in air to afford the labeled aminating reagent **^{15}N-2** (0.95 g, 2.3 mmol, 33 %) as an amorphous white solid.
^{1}H NMR (600 MHz, CDCl3) δ 8.46 (s, 2H, Ar*H*), 8.19 (s, 1H, Ar*H*), 7.90 (d, $J_{N\text{-}H}$ = 87.6 Hz, 1H, N*H*), 1.29 (s, 9H, C(CH_3)$_3$).
^{13}C NMR (151 MHz, CDCl3) δ 153.7 (d, $J_{C–N}$ = 15.2 Hz), 136.7, 133.2 (q, $J_{C–F}$ = 35.0 Hz), 129.97-130.04 (m), 128.1-128.3 (m), 122.4 (q, $J_{C–F}$ = 273.5 Hz), 85.1, 27.7
^{15}N NMR (61 MHz, CDCl3) δ 163.3 (s, 6F, 2×CF_3).
^{19}F NMR (565 MHz, CDCl3) δ – 63.0 (s).
Spectral data matched those reported in the literature (*34*).

2-Chloro-*N*-cyclohexylisonicotinamide (1c)

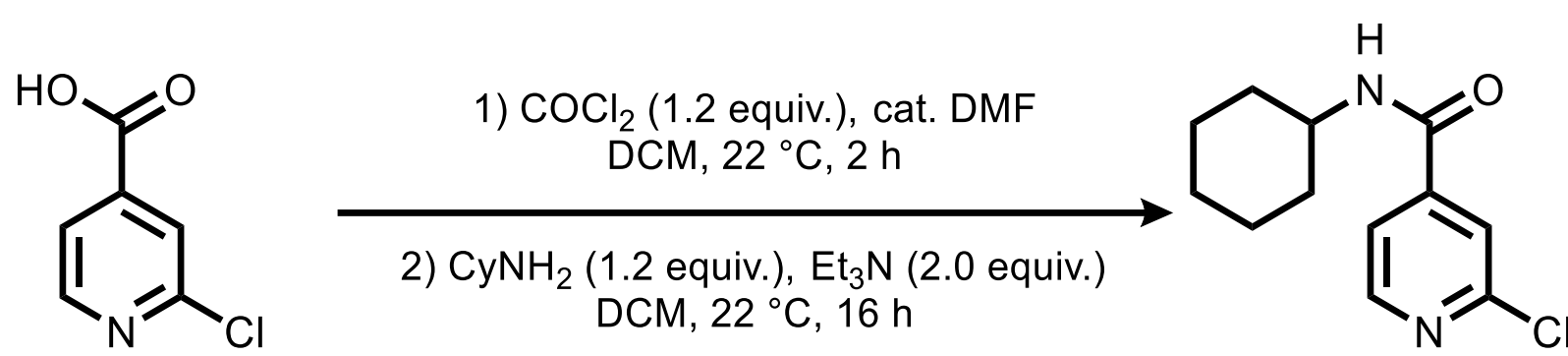

1c

Figure S14. Synthesis of bromo isonicotinic amide **1c.**

An oven-dried 40 mL scintillation vial equipped with a Teflon-coated magnetic stir-bar was charged with 2-chloroisonicotinic acid (0.78 g, 5.0 mmol, 1.0 equiv.) and DCM (10 mL). Then, oxalyl chloride (0.50 mL, 6.0 mmol, 1.2 equiv.) was added followed by 2 drops of DMF. The resulting mixture was stirred open to air for 2 hours at 22 °C. Then, triethylamine (1.3 mL, 10 mmol, 2.0 equiv.) was slowly added followed by cyclohexylamine (0.69 mL, 6.0 mmol, 1.2 equiv.). The reaction mixture was stirred at 22 °C for 16 hours. The resulting mixture was washed with aq. NaOH (1 M, 3 × 20 mL) and brine (20 mL). The organic layer was dried over Na_2SO_4, filtered and concentrated *in vacuo* to give amide **1c** as a white solid (0.61 g, 2.8 mmol, 54 % yield). This material was used in further steps without an addition purification.
^{1}H NMR (400 MHz, Chloroform-*d*) δ 8.49 (dd, *J* = 5.1, 0.8 Hz, 1H, HetAr*H*), 7.62 (dd, *J* = 1.5, 0.8 Hz, 1H, HetAr*H*), 7.51 (dd, *J* = 5.1, 1.5 Hz, 1H, HetAr*H*), 5.97 (br. s, 1H, N*H*), 4.06 – 3.85 (m, 1H, C*H*NH), 2.09 – 1.95 (m, 2H, cyhex), 1.83 – 1.73 (m, 2H, cyhex), 1.74 – 1.61 (m, 1H, cyhex), 1.51 – 1.34 (m, 2H, cyhex), 1.33 – 1.12 (m, 3H, cyhex).
^{13}C{^{1}H} NMR (101 MHz, Chloroform-*d*) δ 163.4, 152.6, 150.6, 145.4, 122.1, 119.9, 49.4, 33.2, 25.6, 25.0.
HRMS (CI/QTOF) m/z: $[M + H]^+$ Calculated for $C_{12}H_{16}ClN_2O^+$ 239.0946; Found 239.0946.

2-Chloro-*N*,*N*-diisopropylisonicotinamide (1d)

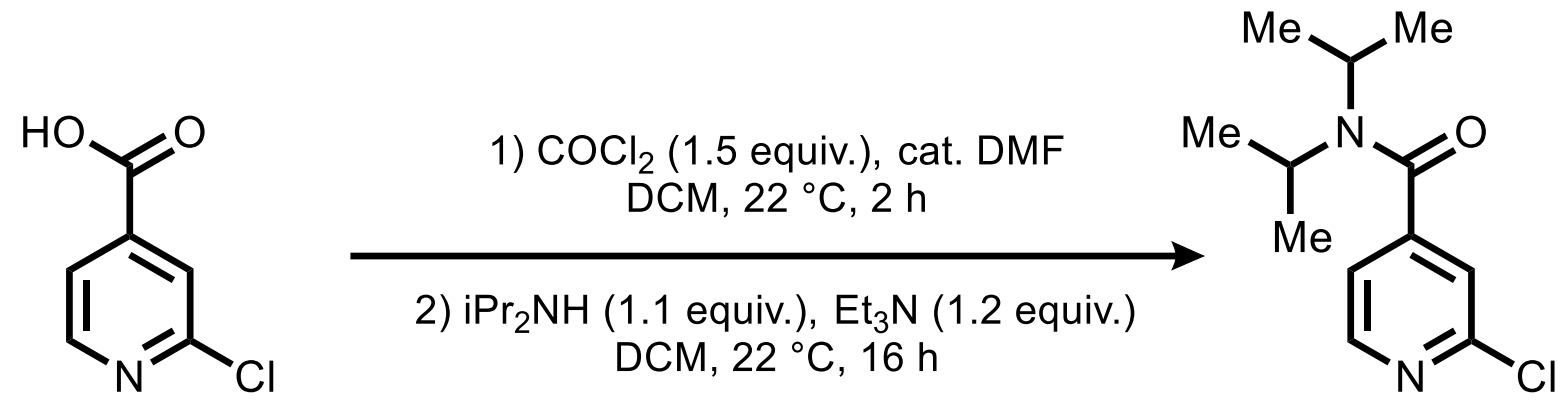

1d

Figure S15. Synthesis of bromo isonicotinic amide **1d**.

An oven-dried 250 mL round-bottom flask equipped with a Teflon-coated magnetic stir-bar was charged with 2-chloroisonicotinic acid (4.74 g, 30.0 mmol, 1.0 equiv.) and DCM (30 mL). Then, oxalyl chloride (3.8 mL, 45 mmol, 1.5 equiv.) was added followed by 2 drops of DMF. The resulting mixture was stirred open to air for 2 hours at 22 °C and then concentrated *in vacuo*. Then, DCM (50 mL) and triethylamine (5.0 mL, 36 mmol, 1.2 equiv.) were added, followed by slow addition of diisopropylamine (4.5 mL, 33 mmol, 1.1 equiv.). The reaction mixture was stirred at 22 °C for 16 hours. The resulting mixture was washed with aq. NaOH (1 M, 3 × 50 mL) and brine (50 mL). The organic layer was dried over Na_2SO_4, filtered and concentrated *in vacuo.* The crude residue was crystallized from hexanes/DCM by layering method and washed with hexanes to give to give amide **1d** as a white solid (5.4 g, 22 mmol, 75 % yield).

^{1}H NMR (500 MHz, Chloroform-*d* δ 8.45 (d, *J* = 5.0 Hz, 1H, HetAr*H*), 7.30 – 7.23 (m, 1H, HetAr*H*), 7.14 (dd, *J* = 5.0, 1.4 Hz, 1H, HetAr*H*), 3.68 (br. s, 1H, C*H*$(CH_3)_2$), 3.55 (br. s, 1H, C*H*$(CH_3)_2$), 1.54 (br. s, 6H, CH(CH_3)(CH_3)), 1.18 (br. s, 6H, CH(CH_3)(CH_3)).

^{13}C{^{1}H} NMR (126 MHz, $CDCl_3$) δ 166.8, 152.2, 150.3, 149.1, 120.9, 118.9, 51.2, 46.4, 20.8, 20.6.

HRMS (CI/QTOF) m/z: $[M + H]^+$ Calculated for $C_{12}H_{18}ClN_2O^+$ 241.1102; Found 241.1104.

2-Chloro-*N*-methyl-*N*-phenylisonicotinamide (1e)

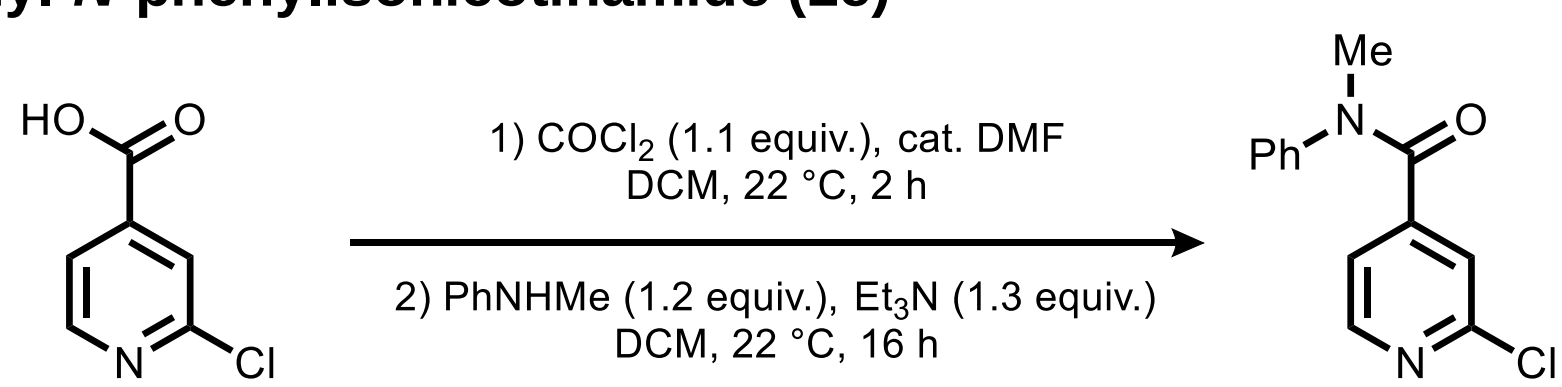

1e

Figure S16. Synthesis of bromo isonicotinic amide **1e**.

An oven-dried 40 mL scintillation vial equipped with a Teflon-coated magnetic stir-bar was charged with 2-chloroisonicotinic acid (0.78 g, 5.0 mmol, 1.0 equiv.) and DCM (10 mL). Then, oxalyl chloride (0.50 mL, 6.0 mmol, 1.2 equiv.) was added followed by 2 drops of DMF. The resulting mixture was stirred open to air for 2 hours at 22 °C. Then, triethylamine (1.3 mL, 10 mmol, 2.0 equiv.) was slowly added followed by *N*-methylaniline (0.65 mL, 6.0 mmol, 1.2 equiv.). The reaction mixture was stirred at 22 °C for 16 hours. The resulting mixture was washed with aq. NaOH (1 M, 3 × 20 mL) and brine (20 mL). The organic layer was dried over Na_2SO_4, filtered and concentrated *in vacuo* to give amide **1e** as a brown oil (1.15 g, 4.66 mmol, 93 % yield). This material was used in further steps without an addition purification.

^{1}H NMR (400 MHz, $CDCl_3$) δ 8.19 (d, *J* = 5.1 Hz, 1H, HetAr*H*), 7.35 – 7.12 (m, 4H, Ar*H* and HetAr*H*), 7.04 (d, *J* = 7.5 Hz, 2H, Ar*H*), 6.98 (d, *J* = 5.1 Hz, 1H, HetAr*H*), 3.49 (s, 3H, CH_3).

^{13}C{^{1}H} NMR (101 MHz, $CDCl_3$) δ 166.9, 151.7, 149.6, 146.7, 143.4, 129.9, 127.9, 127.1, 123.6, 121.3, 38.3.

HRMS (CI/QTOF) m/z: $[M + H]^+$ Calculated for $C_{13}H_{12}ClN_2O^+$ 247.0633; Found 247.0632.

4-((6-chloropyridin-3-yl)sulfonyl)morpholine (1f)

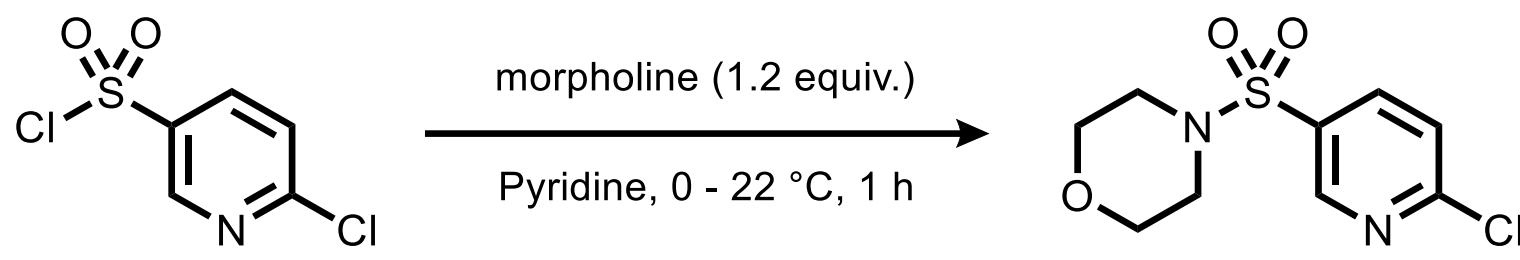

Figure S17. Synthesis of sulfonamide **1f**.

An oven-dried 40 mL scintillation vial equipped with a Teflon-coated magnetic stir-bar was charged with 6-chloropyridine-3-sulfonyl chloride (1.06 g, 5.00 mmol, 1.0 equiv.) and pyridine (10 mL), and cooled to 0 °C. Then, morpholine (0.52 mL, 6.0 mmol, 1.2 equiv.) was added dropwise. The resulting mixture was warmed up to 22 °C stirred for 1 hours. The resulting mixture was poured into water (100 mL). The mixture was filtered, and the residue was dissolved in DCM (100 mL) and washed with water (50 mL) and brine (50 mL). The organic layer was dried over Na_2SO_4, filtered and concentrated *in vacuo* to give sulfonamide **1f** as a white solid (1.31 g, 5 mmol, quant. yield). This material was used in further steps without an addition purification.
^{1}H NMR (400 MHz, $CDCl_3$) δ 8.76 (dd, *J* = 2.5, 0.7 Hz, 1H, HetAr*H*), 7.98 (dd, *J* = 8.3, 2.5 Hz, 1H, HetAr*H*), 7.53 (dd, *J* = 8.3, 0.7 Hz, 1H, HetAr*H*), 3.89 – 3.61 (m, 4H, 2×OCH_2CH$_2$N), 3.15 – 2.92 (m, 4H, 2×OCH$_2$CH_2N).
^{13}C{^{1}H} NMR (101 MHz, $CDCl_3$) δ 156.2, 149.0, 138.0, 131.4, 125.0, 66.1, 46.0.
HRMS (CI/QTOF) m/z: $[M + H]^+$ Calculated for $C_9H_{12}ClN_2O_3S^+$ 263.0252; Found 263.0256.

(4-(6-Chloropicolinoyl)piperazin-1-yl)(cyclopropyl)methanone (1g)

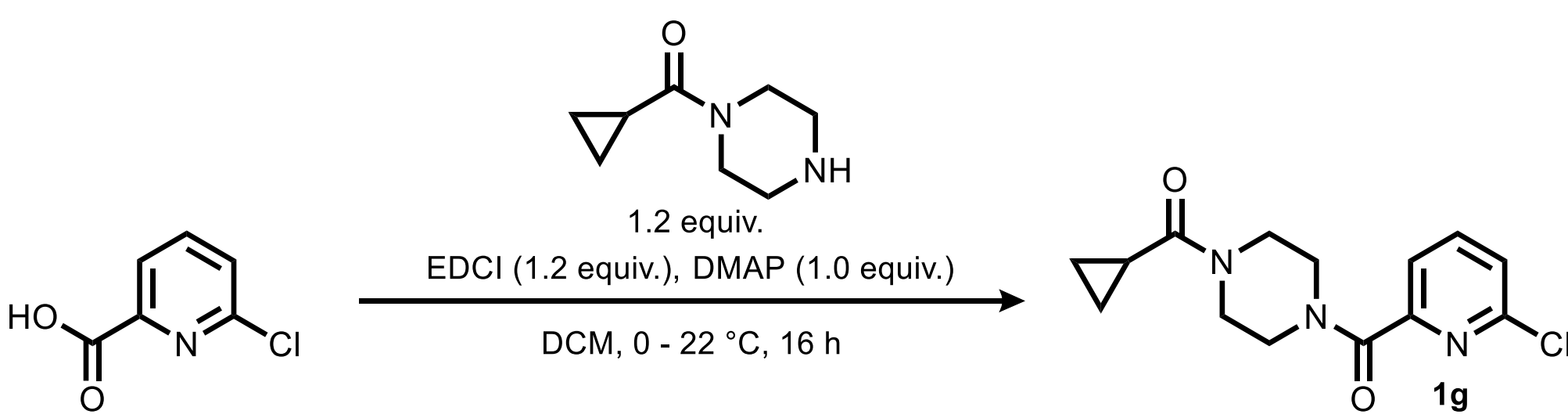

Figure S18. Synthesis of pyridine **1g**.

An oven-dried 40 mL scintillation vial equipped with a Teflon-coated magnetic stir-bar was charged with 6-chloropicolinic acid (0.78 g, 5.00 mmol, 1.0 equiv.), 1-ethyl-3-(3-dimethylaminopropyl)carbodiimide (0.93 g, 6 mmol, 1.2 equiv.), DMAP (0.61 g, 5 mmol, 1.0 equiv.) and DCM (20 mL), and cooled to 0 °C. Then, cyclopropyl(piperazin-1-yl)methanone (0.85 mL, 6.0 mmol, 1.2 equiv.) was added dropwise. The resulting mixture was warmed up to 22 °C stirred for 16 hours. The resulting mixture diluted with DCM (50 mL), and washed with HCl (0.1 M, 50 mL), water (50 mL) and brine (50 mL). The organic layer was dried over Na_2SO_4, filtered and concentrated *in vacuo.* The residue was purified by flash-column chromatography (SiO_2, 50 – 100% EtOAc/hexane) to give amide **1g** as a colourless oil (1.02 g, 3.5 mmol, 69 %).
^{1}H NMR (500 MHz, $CDCl_3$) δ 7.78 (d, *J* = 7.8 Hz, 1H, HetAr*H*), 7.65 (br. s, 1H, HetAr*H*), 7.41 (d, *J* = 8.0 Hz, 1H, HetAr*H*), 3.95 – 3.50 (m, 8H, 4×N-CH_2), 1.75 (br. s, 1H, C*H*(CH$_2$-CH$_2$)), 1.01 (br. s, 2H, CH(CH_2-CH$_2$)), 0.80 (br. s, 2H, CH(CH$_2$-CH$_2$)).
^{13}C{^{1}H} NMR (126 MHz, $CDCl_3$) δ 172.5, 165.9, 153.8, 140.0, 125.8, 123.3, 122.9, 47.5, 47.2, 45.8, 45.2, 42.8 (2C), 42.4, 41.8, 11.2, 7.8. (note: rotameric carbon signals present)
HRMS (CI/QTOF) m/z: $[M + H]^+$ Calculated for $C_{14}H_{17}ClN_3O_2^+$ 294.1004; Found 294.1005.

6-Chloro-*N*-(isoxazol-3-yl)-4-methylnicotinamide (1i)

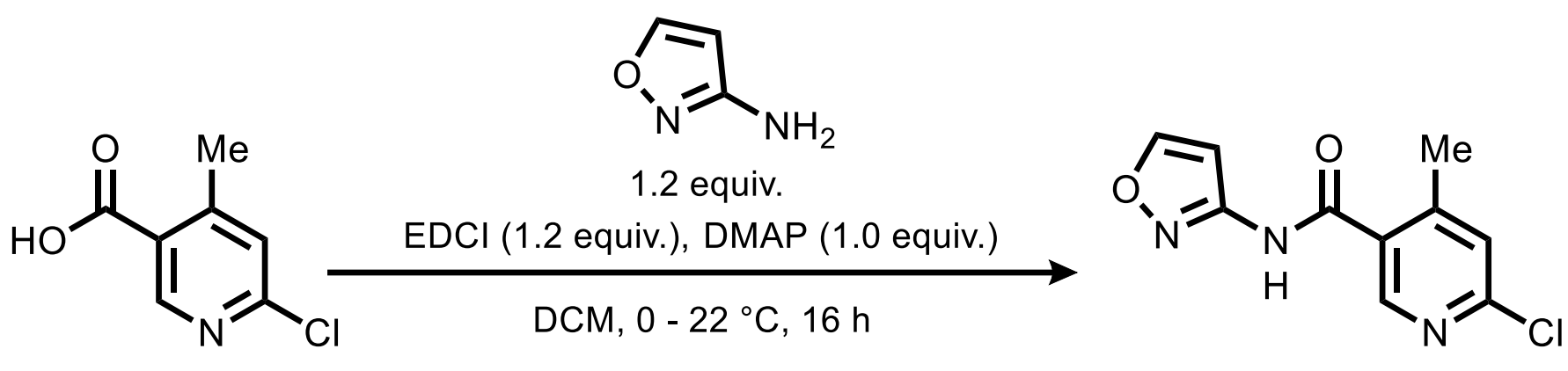

Figure S19. Synthesis of chloropyridine **1i**.

An oven-dried 40 mL scintillation vial equipped with a Teflon-coated magnetic stir-bar was charged with 6-chloro-4-methylnicotinic acid (0.86 g, 5.00 mmol, 1.0 equiv.), 1-ethyl-3-(3-dimethylaminopropyl)carbodiimide (0.93 g, 6 mmol, 1.2 equiv.), DMAP (0.61 g, 5 mmol, 1.0 equiv.) and DCM (20 mL), and cooled to 0 °C. Then, isoxazol-3-amine (0.44 mL, 0.50 g 6.0 mmol, 1.2 equiv.) was added dropwise. The resulting mixture was warmed up to 22 °C stirred for 16 hours. The resulting mixture diluted with DCM (50 mL), and washed with HCl (0.1 M, 50 mL), water (50 mL) and brine (50 mL). Upon standing, in the organic layer white crystals were formed. These crystals were filtered and washed with cold DCM to give amide **1i** as a white solid (0.60 g, 2.5 mmol, 50 %).
<u>^{1}H NMR</u> (400 MHz, $CDCl_3$) δ 10.10 (br. s, 1H, N*H*), 8.63 (s, 1H, HetAr*H*), 8.34 (s, 1H, HetAr*H*), 7.30 (s, 1H, HetAr*H*), 7.19 (s, 1H, HetAr*H*), 2.55 (s, 3H, HetArC*H*$_3$).
<u>^{13}C{^{1}H} NMR</u> (101 MHz, $CDCl_3$) δ 164.5, 159.6, 157.7, 154.0, 150.7, 148.1, 129.7, 126.7, 99.6, 19.9.
<u>HRMS</u> (CI/QTOF) m/z: $[M + H]^+$ Calculated for $C_{10}H_9ClN_3O_2^+$ 238.0378; Found 238.0375.

6-Chloro-*N*-methoxy-N,3-dimethylpicolinamide (1j)

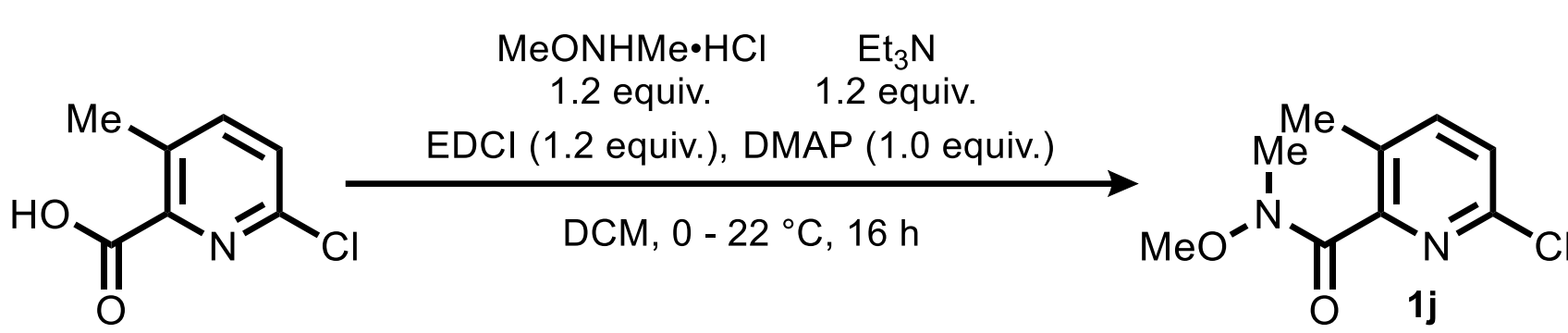

Figure S20. Synthesis of Weinreb amide **1j**.

An oven-dried 40 mL scintillation vial equipped with a Teflon-coated magnetic stir-bar was charged with 6-chloro-3-methylpicolinic acid (0.86 g, 5.00 mmol, 1.0 equiv.), 1-ethyl-3-(3-dimethylaminopropyl)carbodiimide (0.93 g, 6 mmol, 1.2 equiv.), DMAP (0.61 g, 5 mmol, 1.0 equiv.) and DCM (20 mL), and cooled to 0 °C. Then, N,O-dimethylhydroxylamine hydrochloride (0.59 g 6.0 mmol, 1.2 equiv.) was added, followed by dropwise addition of triethyl amine (0.84 mL, 0.61 g, 6.0 mmol,1.2 equiv.). The resulting mixture was warmed up to 22 °C stirred for 16 hours. The resulting mixture diluted with DCM (50 mL), and washed with HCl (0.1 M, 50 mL), water (50 mL) and brine (50 mL). The organic layer was dried over Na_2SO_4, filtered and concentrated *in vacuo.* The residue was purified by flash-column chromatography (SiO_2, 50 – 100% EtOAc/hexane) to give amide **1j** as a white solid (0.71 g, 3.3 mmol, 66 %).
Note: only major rotamer reported.
<u>^{1}H NMR</u> (500 MHz, $CDCl_3$) δ 7.50 (d, *J* = 8.2 Hz, 1H, N-CCl-CH-C*H*), 7.25 (d, *J* = 8.2 Hz, 1H, N-CCl-C*H*-CH), 3.60 (s, 3H, OC*H*$_3$), 3.35 (s, 3H, NC*H*$_3$), 2.30 (s, 3H, HetArC*H*$_3$).
<u>^{13}C{^{1}H} NMR</u> (126 MHz, $CDCl_3$) δ 167.8, 153.5, 147.9, 141.0, 129.6, 124.5, 61.7, 32.0, 17.1.
<u>HRMS</u> (CI/QTOF) m/z: $[M + H]^+$ Calculated for $C_9H_{12}ClN_2O_2^+$ 215.0582; Found 215.0588.

1-(2,6-Dichloro-3-fluorophenyl)ethyl 6-chloro-4-methylpicolinate (1k)

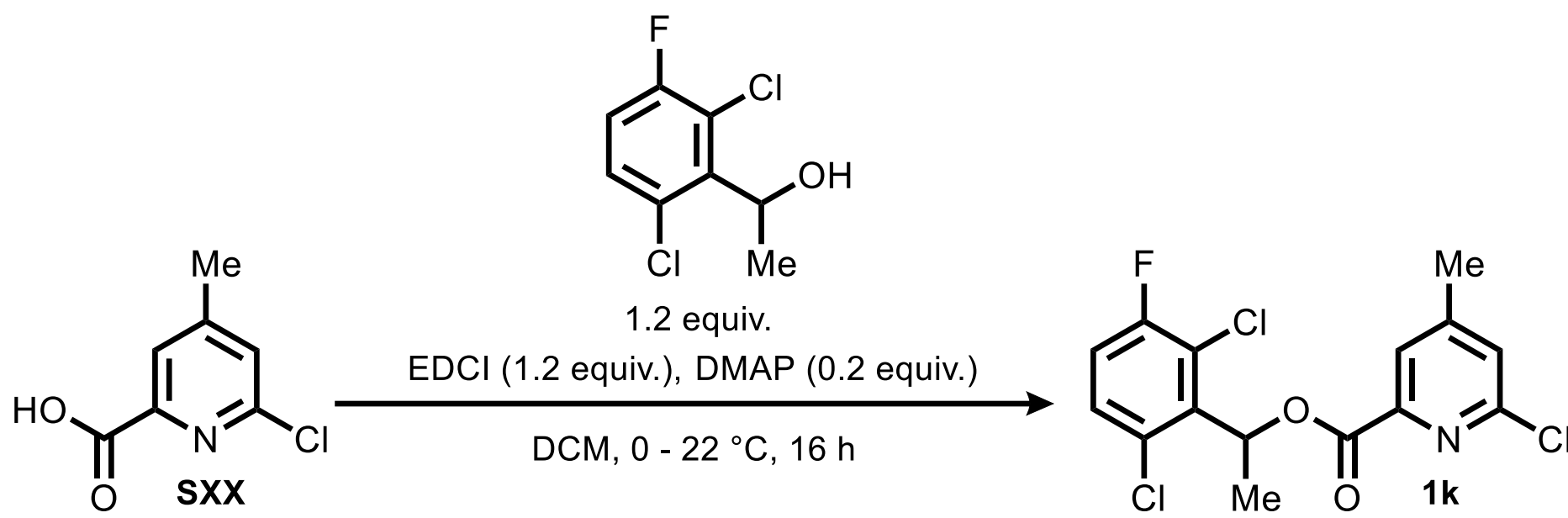

Figure S21. Synthesis of ester **1k**.

An oven-dried 40 mL scintillation vial equipped with a Teflon-coated magnetic stir-bar was charged with 6-chloro-4-methylpicolinic acid (0.86 g, 5.00 mmol, 1.0 equiv.), 1-ethyl-3-(3-dimethylaminopropyl)carbodiimide (0.93 g, 6 mmol, 1.2 equiv.), DMAP (0.12 g, 1 mmol, 1.0 equiv.) and DCM (20 mL), and cooled to 0 °C. Then, 1-(2,6-dichloro-3-fluorophenyl)ethan-1-ol (1.25 g 6.0 mmol, 1.2 equiv.) was added. The resulting mixture was warmed up to 22 °C stirred for 16 hours. The resulting mixture diluted with DCM (50 mL), and washed with HCl (0.1 M, 50 mL), water (50 mL) and brine (50 mL). The organic layer was dried over Na_2SO_4, filtered and concentrated *in vacuo.* The residue was purified by flash-column chromatography (SiO_2, 50 – 100% EtOAc/hexane) to give amide **1k** as a colourless oil (0.30 g, 0.83 mmol, 17 %). The enantiomeric excess was confirmed to be >99% e. e. by chiral HPLC analysis (Daicel CHIRALPAK IC column hexane:IPA 80:20, 0.8 mL/min, λ = 254 nm, τmajor = 12.7 min, τminor = 21.2 min).

^{1}H NMR (500 MHz, $CDCl_3$) δ 7.87 (s, 1H, HetAr*H*), 7.31 (s, 1H, HetAr*H*), 7.28 (dd, *J* = 7.6, 5.0 Hz, 1H, Ar*H*), 7.04 (dd, *J* = 8.4, 7.6 Hz, 1H, Ar*H*), 6.71 (q, *J* = 7.0 Hz, 1H, C*H*-CH_3), 2.42 (s, 3H, HetArCH_3), 1.83 (d, *J* = 7.0 Hz, 3H, CH-CH_3).

^{13}C{^{1}H} NMR (126 MHz, $CDCl_3$) δ 163.1, 157.5 (d, J_{C-F} = 248.7 Hz), 152.1, 151.6, 147.7, 137.2, 129.8 (d, J_{C-F} = 7.6 Hz), 129.2 (d, J_{C-F} = 3.8 Hz), 128.5, 125.2, 122.3 (d, J_{C-F} = 19.4 Hz), 116.5 (d, J_{C-F} = 23.3 Hz), 71.1 (d, *J* = 2.2 Hz), 21.0, 18.1.

^{19}F NMR (470 MHz, $CDCl_3$) δ -112.91 (dd, *J* = 8.4, 5.0 Hz).

HRMS (CI/QTOF) m/z: $[M + H]^+$ Calculated for $C_{15}H_{12}Cl_3FNO_2^+$ 361.9912; Found 361.9914.

Methyl 6-chloro-5-phenylpicolinate (1m)

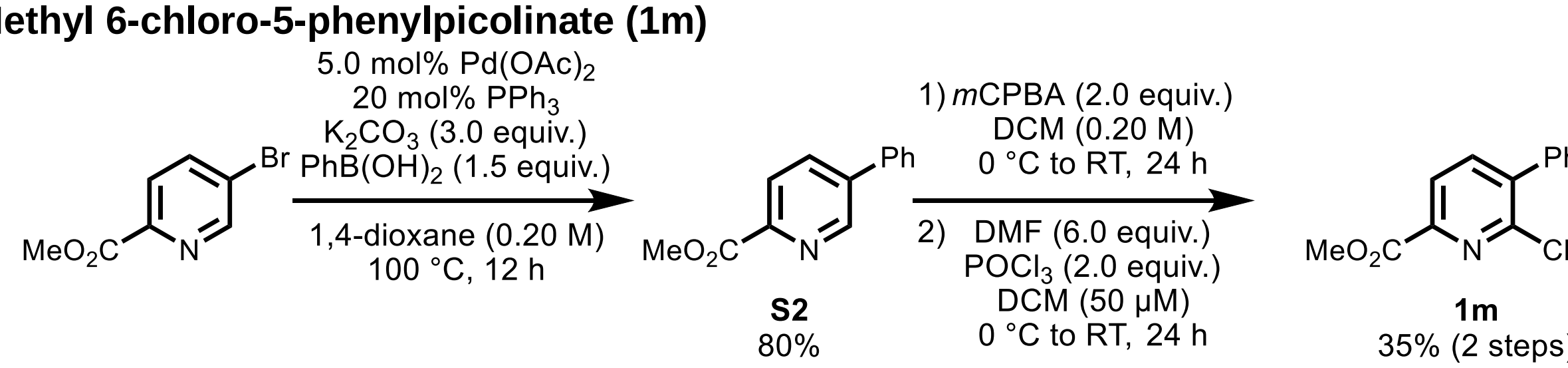

Figure S22. Synthesis of phenylpyridine **1m**.

Arylation

An oven-dried 40 mL scintillation vial equipped with Teflon-coated magnetic stir-bar was charged with methyl 5-bromopicolinate (1.1 g, 5.0 mmol, 1.0 equiv.), $PhB(OH)_2$ (914 mg, 7.5 mmol, 1.5 equiv.), K_2CO_3 (2.1 g, 15 mmol, 3.0 equiv.), $Pd(OAc)_2$ (561 mg, 2.5 mmol, 5.0 mol%), and PPh_3 (262 mg, 1.0 mmol, 20 mol%). The vessel was placed under *vacuum* and refilled with N_2 gas three times. To this were added 1,4-dioxane (25 mL). The vessel was heated at 100 °C for 12 h. After cooling the reaction mixture to room temperature, the mixture was passed through a short silica-gel pad with EtOAc as an eluent. The filtrate was concentrated in *vacuo*. The residue was purified by flash column chromatography (SiO_2, EtOAc/Hex 20 to 100 %) to afford methyl 5-phenylpicolinate (**S2**) as a white solid (855 mg, 4.0 mmol, 80% yield).

^{1}H NMR (500 MHz, $CDCl_3$) δ 8.95 (d, *J* = 2.2 Hz, 1H, HetAr*H*), 8.19 (dd, *J* = 8.1, 2.3 Hz, 1H, HetAr*H*), 8.00 (ddd, *J* = 8.1, 2.4, 1.0 Hz, 1H, HetAr*H*), 7.64 – 7.57 (m, 2H, Ar*H*), 7.53 – 7.46 (m, 2H, Ar*H*), 7.46 – 7.41 (m, 1H, Ar*H*), 4.02 (s, 3H, OCH_3).

^{13}C{^{1}H} NMR (101 MHz, $CDCl_3$) δ 165.7, 148.4, 146.6, 139.9, 136.7, 135.2, 129.4, 129.1, 127.4, 125.3, 53.0.

Spectral data matched those reported in the literature.(*56*)

Chlorination

An oven-dried 40 mL scintillation vial equipped with a Teflon-coated magnetic stir-bar was charged with methyl 5-phenylpicolinate (1.1 g, 5.0 mmol, 1.0 equiv.) and DCM (25 mL), and the solution was stirred at 0 °C for 15 min. Then, *m*CPBA (75wt%: 2.3 g, 10 mmol, 2.0 equiv.) was added slowly and stirred at 0 °C for 1 h. The ice-water bath was removed and stirred additional 24 h at room temperature. DCM was added, and the mixture was washed with saturated $NaHCO_3$. The aqueous layer was extracted by EtOAc three times and dried over Na_2SO_4. The filtrate was concentrated in *vacuo*. The crude product was used for the next step without further purification. An oven-dried 40 mL scintillation vial equipped with a Teflon-coated magnetic stir-bar was charged with crude product, DMF (2.3 mL, 30 mmol, 6.0 equiv.), and DCM (100 mL). The solution was stirred at 0 °C for 15 min. Then, $POCl_3$ (336 µL, 10 mmol, 2.0 equiv.) was added slowly and stirred at 0 °C for 3 h. The ice-water bath was removed and stirred additional 24 h at room temperature. To this was added saturated $NaHCO_3$ and extracted with DCM three times and dried over Na_2SO_4. The filtrate was concentrated in *vacuo*. The residue was purified by flash column chromatography (SiO_2, EtOAc/Hex 20 to 100 %) to afford methyl 6-chloro-5-phenylpicolinate (**1m**) as a white solid (433 mg, 1.8 mmol, 35% yield in 2 steps).

^{1}H NMR (500 MHz, $CDCl_3$) δ 8.12 (d, *J* = 8.0 Hz, 1H), 7.81 (d, *J* = 8.0 Hz, 1H), 7.50–7.40 (m, 5H), 4.01 (s, 3H).

^{13}C{^{1}H} NMR (101 MHz, $CDCl_3$) δ 164.6, 149.9, 146.7, 140.7, 140.5, 136.7, 129.3, 129.0, 128.6, 124.0, 53.2.

HRMS (CI/QTOF) m/z: $[M + H]^+$ Calculated for $C_{13}H_{11}ClNO_2^+$ 248.0473; Found 248.0478.

Methyl 5-benzyl-6-chloropicolinate (1n)

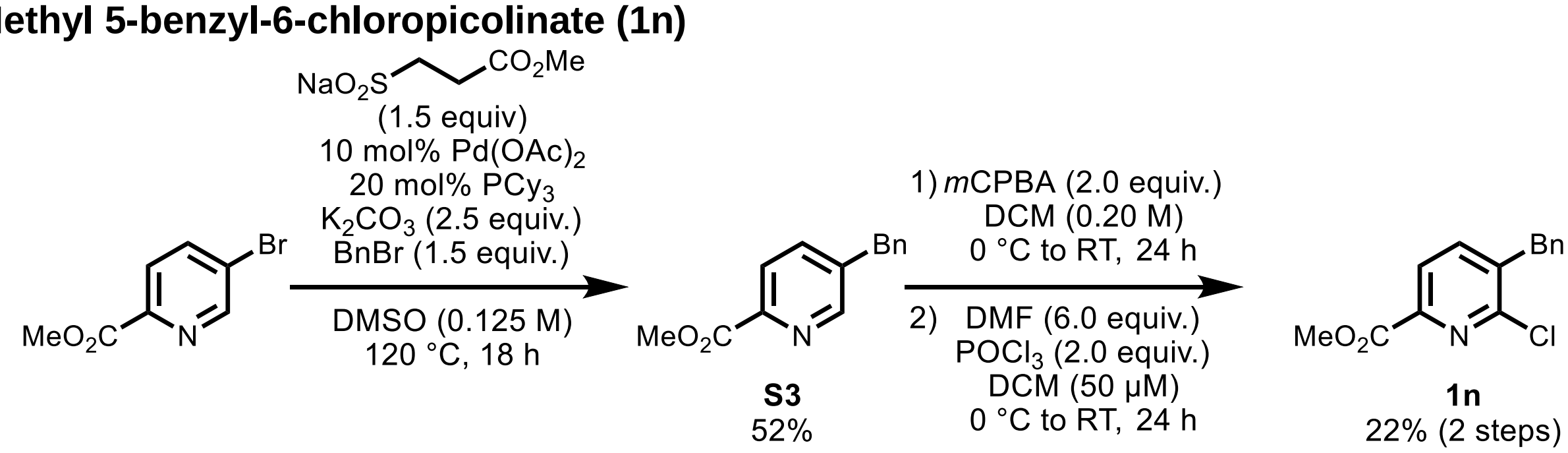

Figure 23 Synthesis of benzylpyridine **1n**.

Alkylation
An oven-dried 40 mL scintillation vial equipped with a Teflon-coated magnetic stir-bar was charged with methyl 5-bromopicolinate (216 mg, 1.0 mmol, 1.0 equiv.), sodium 3-methoxy-3-oxopropane-1-sulfinate (261 mg, 1.5 mmol, 1.5 equiv.), BnBr (178 µL, 1.5 mmol, 1.5 equiv.), K_2CO_3 (346 mg, 2.5 mmol, 2.5 equiv.), $Pd(OAc)_2$ (22.5 mg, 0.10 mmol, 10 mol%), and PCy_3 (56.1 mg, 0.20 mmol, 20 mol%). The vessel was placed under *vacuum* and refilled with N_2 gas three times. To this were added DMSO (8 mL). The vessel was heated at 120 °C for 18 h. After cooling the reaction mixture to room temperature, to this was added EtOAc, and washed with brine three times and dried over Na_2SO_4. The filtrate was concentrated in *vacuo*. The residue was purified by flash column chromatography (SiO_2, EtOAc/Hex 20 to 100 %) to afford methyl 5-benzylpicolinate (**S3**) as a white solid (118 mg, 0.52 mmol, 52% yield).
^{1}H NMR (500 MHz, $CDCl_3$) δ 8.63 (dd, *J* = 2.3, 0.9 Hz, 1H, HetAr*H*), 8.05 (dd, *J* = 8.0, 0.8 Hz, 1H, HetAr*H*), 7.60 (ddd, *J* = 8.1, 6.3, 2.3 Hz, 1H, HetAr*H*), 7.35 – 7.27 (m, 2H, Ar*H*), 7.26 – 7.21 (m, 1H, Ar*H*), 7.19 – 7.12 (m, 2H, Ar*H*), 4.06 (s, 2H, CH_2), 3.99 (s, 3H, OCH_3).
Spectral data matched those reported in the literature.(*56*)

Chlorination
An oven-dried 40 mL scintillation vial equipped with a Teflon-coated magnetic stir-bar was charged with methyl 5-benzylpicolinate (300 mg, 1.3 mmol, 1.0 equiv.) and DCM (6.5 mL), and the solution was stirred at 0 °C for 15 min. Then, *m*CPBA (75wt%: 607 mg, 2.6 mmol, 2.0 equiv.) was added slowly and stirred at 0 °C for 1 h. The ice-water bath was removed and stirred additional 24 h at room temperature. DCM was added, and the mixture was washed with saturated $NaHCO_3$. The aqueous layer was extracted by EtOAc three times and dried over Na_2SO_4. The filtrate was concentrated in *vacuo*. The crude product was used for the next step without further purification. An oven-dried 40 mL scintillation vial equipped with a Teflon-coated magnetic stir-bar was charged with crude product, DMF (613 µL, 7.9 mmol, 6.0 equiv.), and DCM (26 mL). The solution was stirred at 0 °C for 15 min. Then, $POCl_3$ (247 µL, 2.6 mmol, 2.0 equiv.) was added slowly and stirred at 0 °C for 3 h. The ice-water bath was removed and stirred additional 24 h at room temperature. To this was added saturated $NaHCO_3$ and extracted with DCM three times and dried over Na_2SO_4. The filtrate was concentrated in *vacuo*. The residue was purified by flash column chromatography (SiO_2, EtOAc/Hex 20 to 100 %) to afford methyl 5-benzyl-6-chloropicolinate (**1n**) as a white solid (76.1 mg, 0.29 mmol, 22% yield in 2 steps).
^{1}H NMR (500 MHz, $CDCl_3$) δ 7.99 (d, *J* = 8.0 Hz, 1H), 7.55 (d, *J* = 8.0 Hz, 1H), 7.36 (t, *J* = 7.5 Hz, 2H), 7.32–7.27 (m, 1H), 7.21 (d, *J* = 7.5 Hz, 2H), 4.17 (s, 2H), 4.01 (s, 2H).
$^{13}C\{^1H\}$ NMR (101 MHz, $CDCl_3$) δ 164.7, 151.5, 146.1, 140.04, 139.97, 137.3, 129.3, 129.0, 127.2, 124.2, 53.2, 39.1.
HRMS (CI/QTOF) m/z: $[M + H]^+$ Calculated for $C_{14}H_{13}ClNO_2^+$ 262.0629; Found 262.0631.

Methyl 6-chloro-5-(3-ethoxy-3-oxopropyl)picolinate (1o)

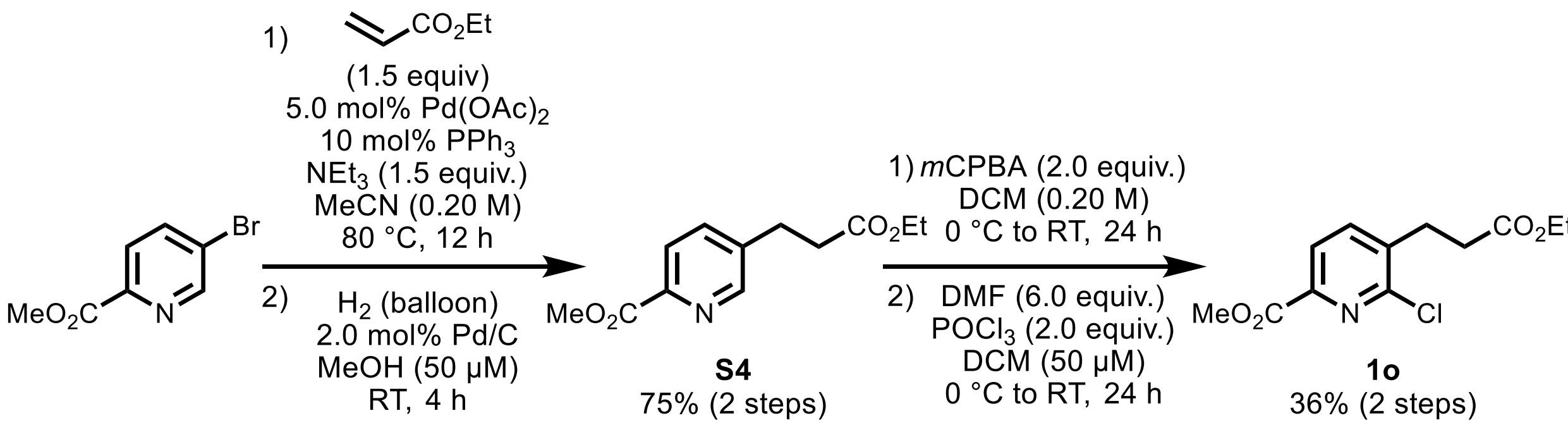

Figure S24. Synthesis of alkylpyridine **1o**.

Alkylation

An oven-dried 200 mL round-bottom flask equipped with a Teflon-coated magnetic stir-bar was charged with methyl 5-bromopicolinate (2.2 g, 10 mmol, 1.0 equiv.), ethyl acrylate (1.6 mL, 15 mmol, 1.5 equiv.), $Pd(OAc)_2$ (112 mg, 0.50 mmol, 5.0 mol%), and PPh_3 (262 mg, 1.0 mmol, 10 mol%). The vessel was placed under *vacuum* and refilled with N_2 gas three times. To this were added MeCN (50 mL) and NEt_3 (2.1 mL, 15 mmol, 1.5 equiv.). The vessel was heated at 80 °C for 12 h. After cooling the reaction mixture to room temperature, the mixture was passed through a short silica-gel pad with EtOAc as an eluent. The filtrate was concentrated in *vacuo*. The residue was purified by flash column chromatography (SiO_2, EtOAc/Hex 20 to 100 %) to afford methyl (*E*)-5-(3-ethoxy-3-oxoprop-1-en-1-yl)picolinate (**S4**) as a white solid (2.3 g, 9.8 mmol, 98% yield). An oven-dried 500 mL roud-bottom flask equipped with a Teflon-coated magnetic stir-bar was charged with methyl (*E*)-5-(3-ethoxy-3-oxoprop-1-en-1-yl)picolinate (2.1 g, 9.5 mmol, 1.0 equiv.) and Pd/C (10wt%: 202 mg, 0.19 μmol, 2.0 mol%). The vessel was placed under *vacuum* and refilled with N_2 gas three times. To this were added MeOH (190 mL) and NEt_3 (2.1 mL, 15 mmol, 1.5 equiv.). The nitrogen in the reaction vessel was replaced with hydrogen from a balloon, and the reaction was conducted at balloon pressure. After stirring for 4 h at room temperature, the reaction mixture was filtered through pad of Celite® and concentrated in *vacuo*. The residue was purified by flash column chromatography (SiO_2, EtOAc/Hex 20 to 100 %) to afford methyl 5-(3-ethoxy-3-oxopropyl)picolinate as a white solid (1.7 g, 7.3 mmol, 77% yield).

<u>^{1}H NMR</u> (500 MHz, $CDCl_3$) δ 8.57 (d, *J* = 3.7 Hz, 1H, HetAr*H*), 8.03 (dt, *J* = 7.0, 3.2 Hz, 1H, HetAr*H*), 7.66 (dd, *J* = 7.6, 3.4 Hz, 1H, HetAr*H*), 4.12 – 4.05 (m, 2H, OCH_2CH_3), 3.96 (s, 3H, OCH_3), 3.00 (td, *J* = 7.5, 3.5 Hz, 2H, HetAr-CH_2), 2.63 (td, *J* = 6.2, 3.6 Hz, 2H, HetArCH_2CH_2), 1.23 – 1.15 (m, 3H, OCH_2CH_3).

<u>^{13}C{^{1}H}</u> NMR (126 MHz, $CDCl_3$) δ 172.0, 165.7, 150.1, 146.2, 140.1, 136.9, 125.1, 60.8, 52.9, 35.2, 28.1, 14.2.

<u>HRMS</u> (CI/QTOF) m/z: $[M + H]^+$ Calculated for $C_{12}H_{16}NO_4^+$ 238.1074; Found 238.1079.

Chlorination

An oven-dried 40 mL scintillation vial equipped with a Teflon-coated magnetic stir-bar was charged with methyl 5-(3-ethoxy-3-oxopropyl)picolinate (1.0 g, 4.2 mmol, 1.0 equiv.) and DCM (21 mL), and the solution was stirred at 0 °C for 15 min. Then, *m*CPBA (75wt%: 1.9 g, 8.4 mmol, 2.0 equiv.) was added slowly and stirred at 0 °C for 1 h. The ice-water bath was removed and stirred additional 24 h at room temperature. DCM was added, and the mixture was washed with saturated $NaHCO_3$. The aqueous layer was extracted by EtOAc three times and dried over Na_2SO_4. The filtrate was concentrated in *vacuo*. The crude product was used for the next step without further purification. An oven-dried 40 mL scintillation vial equipped Teflon-coated magnetic stir-bar was charged with crude product, DMF (2.0 mL, 25 mmol, 6.0 equiv.), and DCM (84 mL). The solution was stirred at 0 °C for 15 min. Then, $POCl_3$ (787 μL, 8.4 mmol, 2.0 equiv.)

was added slowly and stirred at 0 °C for 3 h. The ice-water bath was removed and stirred additional 24 h at room temperature. To this was added saturated $NaHCO_3$ and extracted with DCM three times and dried over Na_2SO_4. The filtrate was concentrated in *vacuo*. The residue was purified by flash column chromatography (SiO_2, EtOAc/Hex 20 to 100 %) to afford methyl 6-chloro-5-(3-ethoxy-3-oxopropyl)picolinate (**1o**) as a white solid (412 mg, 1.5 mmol, 36% yield in 2 steps).

^{1}H NMR (500 MHz, $CDCl_3$) δ 7.98–7.88 (m, 1H), 7.75–7.68 (m, 1H), 4.11–3.98 (m, 2H), 3.95–3.87 (m, 3H), 3.10–2.99 (m, 2H), 2.70–2.57 (m, 2H), 1.21–1.10 (m, 3H).
^{13}C{^{1}H} NMR (101 MHz, $CDCl_3$) δ 171.9, 164.5, 151.2, 146.2, 140.0, 139.0, 124.0, 60.7, 53.0, 32.7, 28.5, 14.1.
HRMS (CI/QTOF) m/z: $[M + H]^+$ Calculated for $C_{12}H_{15}ClNO_4^+$ 272.0684; Found 272.0690

Methyl 5-(((benzyloxy)carbonyl)amino)-6-chloropicolinate (1p)

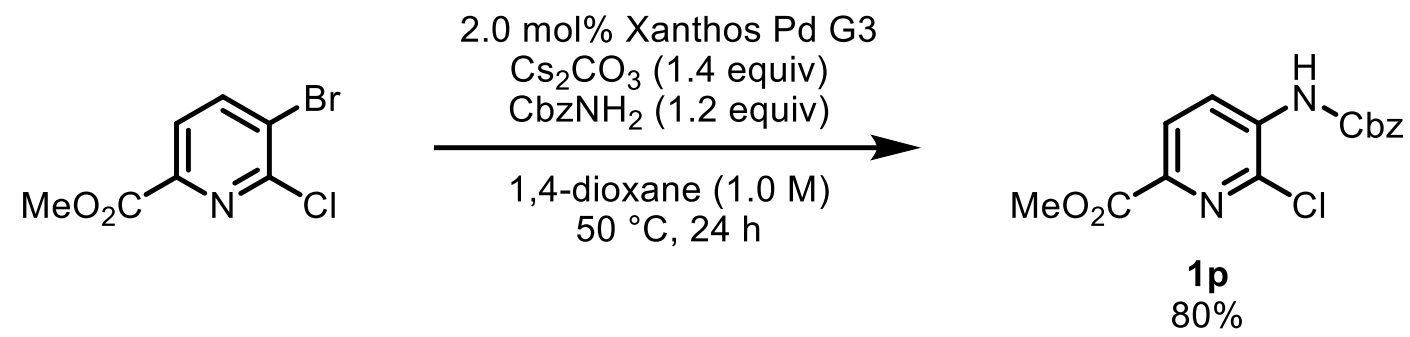

Figure S25. Synthesis of aminopyridine **1p**.

An oven-dried 40 mL scintillation vial equipped with a Teflon-coated magnetic stir-bar was charged with methyl 5-bromo-6-chloropicolinate (376 mg, 1.5 mmol, 1.0 equiv.), Cs_2CO_3 (684 mg, 2.1 mmol, 1.4 equiv.), Xanthos Pd G3 (27.6 mg, 30 µmol, 2.0 mol%), and $CbzNH_2$ (272 mg, 1.8 mmol, 1.2 equiv.). The vessel was placed under *vacuum* and refilled with N_2 gas three times. To this were added 1,4-dioxane (1.5 mL). The vessel was heated at 50 °C for 12 h. After cooling the reaction mixture to room temperature, the mixture was passed through a short silica-gel pad with EtOAc as an eluent. The filtrate was concentrated in *vacuo*. The residue was purified by flash column chromatography (SiO_2, EtOAc/Hex 20 to 100 %) to afford methyl 5-(((benzyloxy)carbonyl)amino)-6-chloropicolinate (**1p**) as a white solid (383 mg, 1.2 mmol, 80% yield).
^{1}H NMR (500 MHz, $CDCl_3$) δ 8.71 (d, *J* = 8.5 Hz, 1H), 8.10 (d, *J* = 8.5 Hz, 1H), 7.50–7.37 (m, 5H), 5.27 (s, 2H), 3.99 (s, 3H).
^{13}C{^{1}H} NMR (101 MHz, $CDCl_3$) δ 164.2, 152.6, 141.1, 138.7, 135.4, 135.1, 128.93, 128.88, 128.7, 126.6, 125.6, 68.2, 53.1.
HRMS (CI/QTOF) m/z: $[M + H]^+$ Calculated for $C_{15}H_{14}ClN_2O_4^+$ 321.0637; Found 321.0633.

2-Chloro-4-(trifluoromethyl)-5,6,7,8-tetrahydroquinoline (1s)

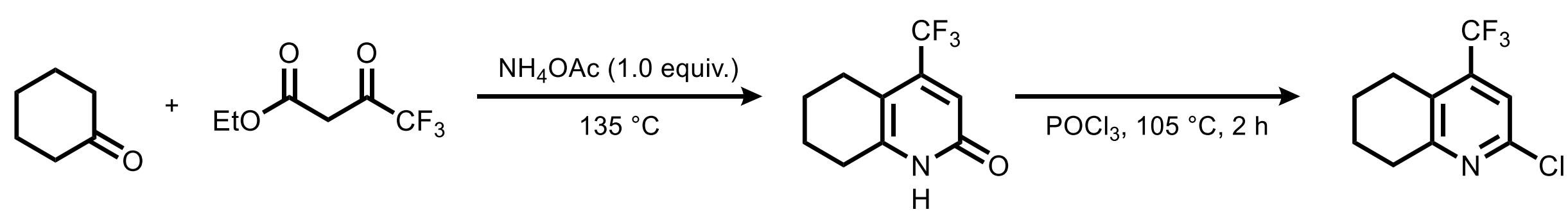

Figure S26. Synthesis of fused chloropyridine **1s**.

An oven-dried 40 mL scintillation vial equipped with a Teflon-coated magnetic stir-bar was charged with cyclohexanone (2.1 mL, 2.0 g, 20 mmol, 1.0 equiv.), ethyl 4,4,4-trifluoroacetoacetate (2.9 mL, 3.68 g, 20 mmol, 1.0 equiv.) and NH_4OAc (1.54 g, 20 mmol, 1.0 equiv.). The resulting mixture was heated at 135 ° for 16 h. The resulting mixture was cooled down and Et_2O (15 mL) was added. The resulting suspension was filtered, washing with more Et_2O (15 mL) to give 4-(trifluoromethyl)-5,6,7,8-tetrahydroquinolin-2(1H)-one (**S5**, 2.3 g, 9.8 mmol, 49 %) as a white solid.
^{1}H NMR (400 MHz, $CDCl_3$) δ 13.30 (br. s, 1H, N*H*), 6.74 (s, 1H, HetAr*H*), 2.74 (t, *J* = 6.2 Hz, 2H, CH_2-CH_2-CH_2-CH_2), 2.60 (t, *J* = 6.0 Hz, 2H, CH_2-CH_2-CH_2-CH_2), 1.79 (dt, *J* = 11.5, 6.3 Hz, 4H, CH_2-CH_2-CH_2-CH_2).
^{13}C{^{1}H} NMR (101 MHz, $CDCl_3$) δ 164.0, 146.1, 142.7 (q, $J_{C\text{-}Fz}$ = 30.6 Hz), 122.7 (q, $J_{C\text{-}F}$ = 275.8 Hz), 115.7 (q, $J_{C\text{-}F}$ = 6.1 Hz), 111.4, 27.5, 23.0 (q, $J_{C\text{-}F}$ = 2.6 Hz), 22.3, 21.2.
^{19}F NMR (376 MHz, $CDCl_3$) δ -64.7 (s, 3F, C*F*$_3$).
HRMS (CI/QTOF) m/z: $[M + H]^+$ Calculated for $C_{10}H_{11}F_3NO^+$ 218.0787; Found 218.0791.

An oven-dried 40 mL scintillation vial equipped with a Teflon-coated magnetic stir-bar was charged with 4-(trifluoromethyl)-5,6,7,8-tetrahydroquinolin-2(1H)-one (2.3 g, 9.8 mmol, 1.0 equiv.)

and $POCl_3$ (20 mL). The resulting mixture was heated at 105 °C for 2 h. The resulting mixture was cooled down and poured into ice cold water (200 mL). Then, aq. ammonia (25 %, 100 mL) was slowly added. The resulting mixture was extracted with DCM (3 ×50 mL). The combined organic layers were washed with brine (100 mL), dried over Na_2SO_4, filtered and concentrated in vacuo to give 2-chloro-4-(trifluoromethyl)-5,6,7,8-tetrahydroquinoline (**1s**, 2.3 g, 9.8 mmol, 91 %). This material was used without further purification.
^{1}H NMR (400 MHz, $CDCl_3$) δ 7.35 (s, 1H, HetAr*H*), 2.98 (t, *J* = 6.3 Hz, 2H, HetArC*H*$_2$), 2.88 (t, *J* = 6.3 Hz, 2H, HetArC*H*$_2$), 2.02 – 1.70 (m, 4H, 2×-C*H*$_2$-).
^{19}F NMR (376 MHz, $CDCl_3$) δ -63.8 (3F, CF_3).
^{13}C{^{1}H} NMR (101 MHz, $CDCl_3$) δ 160.8, 148.4, 139.0 (q, J_{C-F} = 31.5 Hz), 129.0 (q, J_{C-F} = 1.6 Hz), 122.6 (q, J_{C-F} = 275.4 Hz), 118.1 (q, J_{C-F} = 5.8 Hz), 33.0, 24.8 (q, J_{C-F} = 2.3 Hz), 22.0, 21.9.
HRMS (CI/QTOF) m/z: [M + H]$^+$ Calculated for $C_{10}H_{10}ClF_3N^+$ 236.0448; Found 236.0448.

Methyl 6-chloro-2-phenylnicotinate (1r)

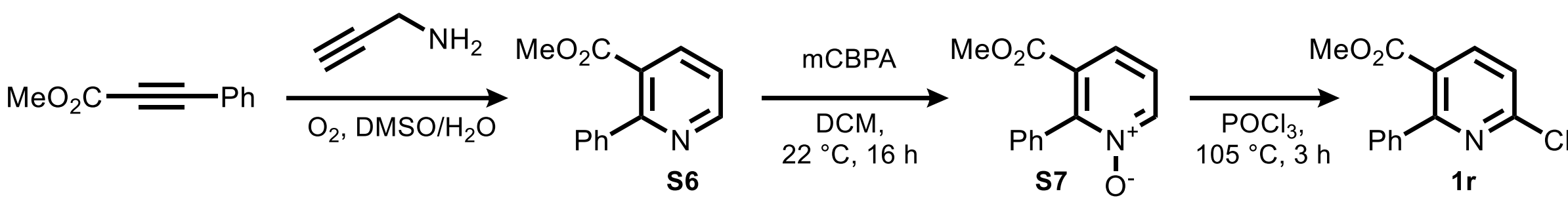

Figure S27. Synthesis of phenyl pyridine **1r**.

According to a reported procedure,(*57*) an oven-dried 40 mL scintillation vial equipped with a Teflon-coated magnetic stirrer was charged with methyl 3-phenylpropiolate (0.64 g, 0.60 mL, 4 mmol, 1.0 equiv.), prop-2-yn-1-amine (0.38 mL, 0.33 g, 15 mmol, 1.5 equiv.), DMSO (18 mL) and H_2O (2 mL). The resulting mixture was sparged with O_2 for 10 minutes, and then stirred stirred for 16 hours at 80 °C. After cooling down, the resulting mixture was diluted with EtOAc (100 mL) and washed with aq. LiCl (5 wt%, 5×20 mL) and brine (50 mL). The organic layer was dried over Na_2SO_4 and concentrated *in vacuo*. The crude material was purified via flash column chromatography (20-100 % EtOAc/Hexanes) to give pyridine **S6** as a brown oil (0.23 g, 1.1 mmol, 28 % yield).
^{1}H NMR (400 MHz, $CDCl_3$) δ 8.78 (dd, *J* = 4.8, 1.8 Hz, 1H, N-C*H*), 8.10 (dd, *J* = 7.8, 1.8 Hz, 1H, N-CH-CH-C*H*), 7.58 – 7.49 (m, 2H, Ar*H*), 7.49 – 7.38 (m, 3H, Ar*H*), 7.33 (dd, *J* = 7.8, 4.8 Hz, 1H, N-CH-C*H*-CH), 3.70 (s, 3H, OC*H*$_3$).
^{13}C{^{1}H} NMR (101 MHz, $CDCl_3$) δ 168.7, 159.0, 151.5, 140.2, 138.0, 128.9, 128.6, 128.3, 127.1, 121.7, 52.5.
HRMS (CI/QTOF) m/z: [M + H]$^+$ Calculated for $C_{13}H_{12}NO_2^+$ 214.0863; Found 214.0869.

A 100 mL round bottom flask was charged with ethyl 2,5-dimethyl-4-phenylnicotinate (0.23 g, 1.1 mmol, 1.0 equiv.), meta-chloroperoxybenzoic acid (0.51 g, 2.2 mmol, 2.0 equiv., 75 % mass purity) and DCM (5 mL). The resulting mixture was stirred at 22 °C for 16 hours. Then, silica was directly added, and the mixture dry loaded for flash column chromatography (SiO_2, 0-10% EtOAc/MeOH) to give 3-(methoxycarbonyl)-2-phenylpyridine 1-oxide (**S7**, 0.17 g, 0.84 mmol, 76 %).
^{1}H NMR (400 MHz, $CDCl_3$) δ 8.43 (dd, *J* = 6.5, 1.3 Hz, 1H, N-C*H*), 7.64 (dd, *J* = 8.0, 1.2 Hz, 1H, N-CH-CH-C*H*), 7.53 – 7.45 (m, 3H, Ar*H*), 7.45 – 7.37 (m, 2H, Ar*H*), 7.31 (dd, *J* = 8.0, 6.5 Hz, 1H, N-CH-C*H*-CH), 3.59 (s, 3H, OC*H*$_3$).

$^{13}C\{^1H\}$ NMR (101 MHz, $CDCl_3$) δ 165.7, 149.9, 142.3, 132.1, 131.6, 129.6, 129.3, 128.4, 125.9, 124.4, 52.8.
HRMS (APCI/QTOF) m/z: $[M + H]^+$ Calculated for $C_{13}H_{12}NO_3^+$ 230.0812; Found 230.0810.

An 8 mL scintillation vial was charged with 3-(methoxycarbonyl)-2-phenylpyridine 1-oxide (0.17 g, 0.84 mmol, 1.0 equiv.) and $POCl_3$ (2 mL). The resulting mixture was stirred for 3 hours at 105 °C. The resulting mixture was cooled down and carefully added to an ice-cold water (100 mL) containing 10 mL aq. NH_3 (25 wt%). The mixture was stirred at 0 °C for 10 minutes and then extracted with DCM (3 × 20 mL). The combined organic layers were washed with brine (50 mL), dried over Na_2SO_4, filtered and concentrated *in vacuo*. The resulting crude was purified via flash column chromatography to give methyl 6-chloro-2-phenylnicotinate (**1r**, 0.11 g, 0.43 mmol, 51%) as a colorless oil.
^1H NMR (400 MHz, $CDCl_3$) δ 8.06 (d, J = 8.2 Hz, 1H, N-CCl-CH-CH), 7.59 – 7.47 (m, 2H, ArH), 7.47 – 7.39 (m, 3H, ArH), 7.36 (d, J = 8.2 Hz, 1H, N-CCl-CH-CH), 3.70 (s, 3H, OCH_3).
$^{13}C\{^1H\}$ NMR (101 MHz, $CDCl_3$) δ 167.8, 159.8, 153.1, 140.7, 138.7, 129.4, 128.8, 128.4, 125.7, 122.4, 52.6.
HRMS (APCI/QTOF) m/z: $[M + H]^+$ Calculated $C_{13}H_{11}ClNO_2^+$ 248.0473; Found 248.0476.

Ethyl 4-(4-(tert-butyl)phenyl)-6-chloro-2-methylnicotinate (4t)

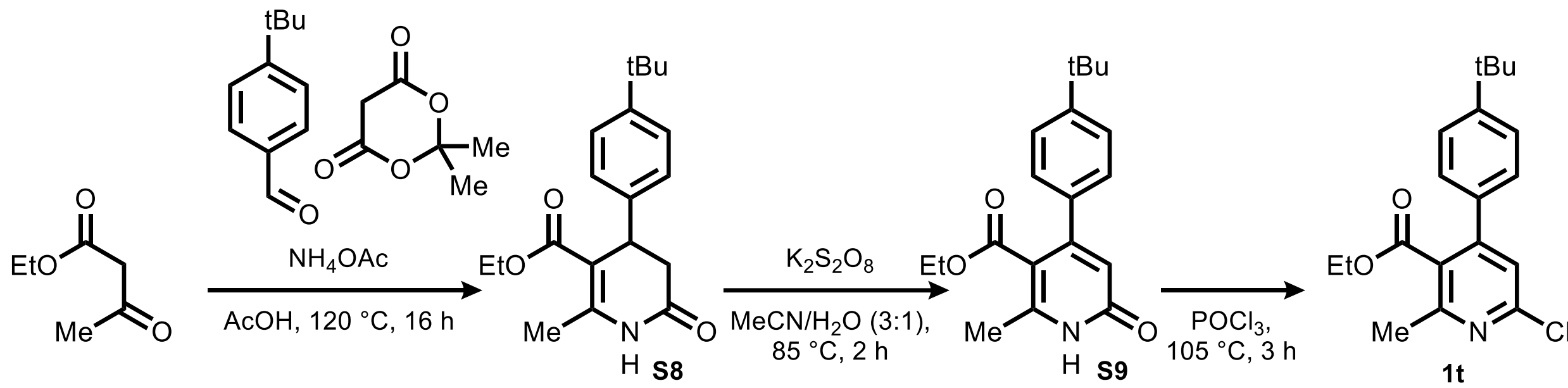

Figure 28. 4-component condensation towards pyridine **1t**.

According to a reported procedure,(*36*) an oven-dried 40 mL scintillation vial equipped with a Teflon-coated magnetic stirrer was charged with ethyl 3-oxobutanoate (1.3 mL, 1.3 g, 10 mmol, 1.0 equiv.), 2,2-dimethyl-1,3-dioxane-4,6-dione (1.5 g, 10 mmol, 1.0 equiv.), ammonium acetate (0.80 g, 10.5 mmol, 1.05 equiv.), 4-(tert-butyl)benzaldehyde (3.2 g) and AcOH (10 mL). The resulting mixture was stirred for 16 hours at 120 °C. After cooling down, the resulting mixture was poured in water (200 mL). The water was decanted, the solids dissolved in EtOAc (100 mL) and washed with water (50 mL) and brine (50 mL). The organic layer was dried over Na_2SO_4 and concentrated *in vacu*o. The crude material was purified via crystallization from MeOH to ethyl 4-(4-(tert-butyl)phenyl)-2-methyl-6-oxo-1,4,5,6-tetrahydropyridine-3-carboxylate (**S8**, 1 g, 3.2 mmol, 32 %) as a white solid.
^1H NMR (400 MHz, $CDCl_3$) δ 7.42 (br. s, 1H, NH), 7.30 – 7.24 (m, 2H, ArH), 7.14 – 7.07 (m, 2H, ArH), 4.24 (ddd, J = 7.9, 2.2, 0.8 Hz, 1H, CHCH$_a$H$_b$), 4.20 – 4.04 (m, 2H, OCH_2CH$_3$), 2.90 (dd, J = 16.5, 7.9 Hz, 1H, CHCH_aH$_b$), 2.71 (ddd, J = 16.5, 2.2, 1.0 Hz, 1H, CHCH$_a$$H_b$), 2.38 (d, J = 0.8 Hz, 3H, CH_3), 1.28 (s, 9H, C(CH_3)$_3$), 1.21 (t, J = 7.1 Hz, 3H, OCH$_2$CH_3).
$^{13}C\{^1H\}$ NMR (101 MHz, $CDCl_3$) δ 170.7, 167.1, 149.8, 145.4, 139.1, 126.5, 125.7, 108.0, 60.4, 38.0, 37.5, 34.5, 31.5, 19.5, 14.3.
HRMS (CI/QTOF) m/z: $[M + H]^+$ Calculated for $C_{19}H_{26}NO_3^+$ 316.1907; Found 316.1902.

According to a reported procedure,(*36*) an oven-dried 250 mL round bottom flask equipped with a Teflon-coated magnetic stirrer was charged with 4-(4-(tert-butyl)phenyl)-2-methyl-6-oxo-1,4,5,6-tetrahydropyridine-3-carboxylate (0.9 g, 2.8 mmol, 1.0 equiv.), potassium peroxydisulfate (0.8 g, 2.8 mmol, 1.0 equiv.), MeCN (90 mL) and water (30 mL). The resulting mixture was stirred for 2 hours at 85 °C. After cooling down, the acetonitrile was removed *in vacuo* and the resulting suspension filtered, washed with cold water (20 mL) to give ethyl 4-(4-(tert-butyl)phenyl)-2-methyl-6-oxo-1,6-dihydropyridine-3-carboxylate (**S9**, 0.82 g, 2.6 mmol, 92 %) as a yellow solid.
^{1}H NMR (400 MHz, $CDCl_3$) δ 7.44 – 7.37 (m, 2H, Ar*H*), 7.25 – 7.21 (m, 2H, Ar*H*), 6.42 (s, 1H, HetAr*H*), 3.94 (q, *J* = 7.1 Hz, 2H, OCH_2CH_3), 2.52 (s, 3H, $HetArCH_3$), 1.34 (s, 9H, $C(CH_3)_3$), 0.78 (t, *J* = 7.1 Hz, 3H, OCH_2CH_3).
^{13}C{^{1}H} NMR (101 MHz, $CDCl_3$) δ 167.3, 164.7, 154.9, 152.0, 147.4, 136.4, 126.9, 125.5, 116.8, 113.2, 61.2, 34.8, 31.4, 18.2, 13.4.
HRMS (CI/QTOF) m/z: $[M + H]^+$ Calculated for $C_{19}H_{24}NO_3^+$ 314.1751; Found 314.1752.

An 8 mL scintillation vial was charged with 4-(4-(tert-butyl)phenyl)-2-methyl-6-oxo-1,6-dihydropyridine-3-carboxylate (0.82 g, 2.6 mmol, 1.0 equiv.) and $POCl_3$ (5 mL). The resulting mixture was stirred for 16 hours at 105 °C. The resulting mixture was cooled down and carefully added to an ice-cold water (100 mL) containing 25 mL aq. NH_3 (25 wt%). The mixture was stirred at 0 °C for 10 minutes and then extracted with DCM (3 × 20 mL). The combined organic layers were washed with brine (50 mL), dried over Na_2SO_4, filtered and concentrated *in vacuo*. The resulting crude was purified via flash column chromatography (SiO_2, 5-20 % EtOAc/hexanes) to give ethyl 4-(4-(tert-butyl)phenyl)-6-chloro-2-methylnicotinate (**1t**, 0.65 g, 2.0 mmol, 76 %) as a colorless solid.
^{1}H NMR (400 MHz, $CDCl_3$) δ 7.50 – 7.44 (m, 2H, Ar*H*), 7.33 – 7.29 (m, 2H, Ar*H*), 7.28 (s, 1H, HetAr*H*), 4.14 (q, *J* = 7.1 Hz, 2H, OCH_2CH_3), 2.63 (s, 3H), 1.36 (s, 9H, $C(CH_3)_3$), 0.98 (t, *J* = 7.1 Hz, 3H, OCH_2CH_3).
^{13}C{^{1}H} NMR (101 MHz, $CDCl_3$) δ 168.2, 156.9, 152.5, 151.3, 151.1, 134.5, 127.7, 127.6, 125.8, 121.9, 61.7, 34.9, 31.4, 22.8, 13.6.
HRMS (CI/QTOF) m/z: $[M + H]^+$ Calculated for $C_{19}H_{23}ClNO_2^+$ 332.1412; Found 332.1418.

Methyl 1-chloro-6,7-dihydro-5H-cyclopenta[c]pyridine-3-carboxylate (1u)

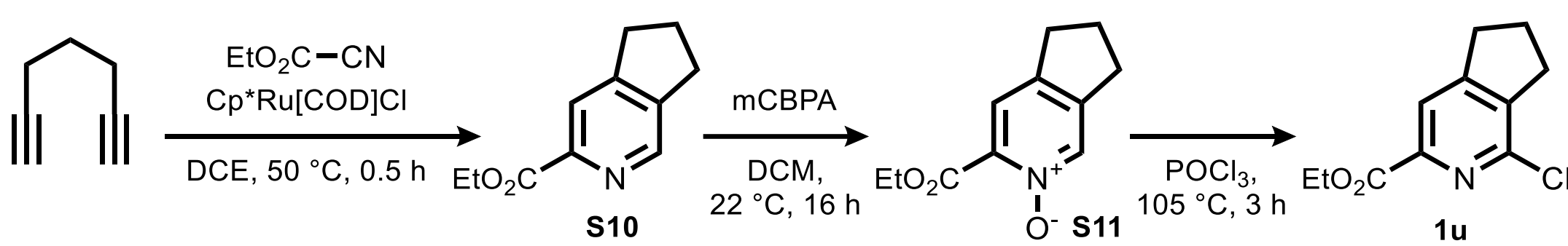

Figure 29. Transition metal catalyzed cycloaddtion towards **1u**.

According to a reported procedure (*37*) an oven-dried 250 mL round-bottom flask equipped with a Teflon-coated magnetic stirrer was charged with ethyl cyanoformate (0.68 mL, 6.0 mmol, 1.0 equiv.), Cp*Ru[COD]Cl (45 mg, 0.8 mL) and DCE (40 mL). In another oven dried 100 mL flask, hepta-1,6-diyne (0.60 mL, 4.0 mmol, 1.0 equiv.) and DCE (60 mL) were added, and the contents were cannulated to the first flask over 30 min at 22 °. Then, the resulting mixture was heated to 60 °C for 30 minutes. After cooling down, the crude was directly dry-loaded on silica gel and purified via flash column chromatography (SiO_2, 30 – 70 % EtOAc/hexanes) to give ethyl 6,7-dihydro-5H-cyclopenta[c]pyridine-3-carboxylate (**S10**, 0.52 g, 3.0 mmol, 74 %) as a colorless oil.
^{1}H NMR (500 MHz, $CDCl_3$) δ 8.58 (s, 1H, HetAr*H*), 8.02 (s, 1H, HetAr*H*), 4.47 (q, *J* = 7.2 Hz, 2H, OCH_2CH_3), 2.99 (q, *J* = 7.6 Hz, 4H, $-CH_2-CH_2-CH_2-$), 2.17 (p, *J* = 7.6 Hz, 2H, $-CH_2-CH_2-CH_2-$), 1.44 (t, *J* = 7.1 Hz, 3H, OCH_2CH_3).
^{13}C{^{1}H} NMR (126 MHz, $CDCl_3$) δ 165.9, 155.0, 146.4, 145.8, 144.2, 121.6, 61.9, 32.7, 30.6, 25.1, 14.5.

Spectral data match those reported in the literature.(*37*)

A 100 mL round bottom flask was charged with ethyl 6,7-dihydro-5H-cyclopenta[c]pyridine-3-carboxylate (0.52 g, 3.0 mmol, 1.0 equiv.), meta-chloroperoxybenzoic acid (1.4 g, 6.0 mmol, 2.0 equiv., 75 % mass purity) and DCM (20 mL). The resulting mixture was stirred at 22 °C for 16 hours. Then, silica was directly added and the mixture dry loaded for flash column chromatography (SiO_2, 0-10% EtOAc/MeOH) to give 3-(ethoxycarbonyl)-6,7-dihydro-5H-cyclopenta[c]pyridine 2-oxide (**S11**, 0.48 g, 2.3 mmol, 77 %) as a colorless solid.

<u>^{1}H NMR</u> (500 MHz, $CDCl_3$) δ 8.13 (s, 1H, HetAr*H*), 7.40 (s, 1H, HetAr*H*), 4.49 – 4.41 (m, 2H, OC*H*$_2$CH$_3$), 2.97 – 2.87 (m, 4H, -C*H*$_2$-CH$_2$-C*H*$_2$-), 2.19 (p, *J* = 7.5 Hz, 2H, -CH$_2$-C*H*$_2$-CH$_2$-), 1.40 (td, *J* = 7.2, 1.3 Hz, 3H, OCH$_2$C*H*$_3$).

<u>^{13}C{^{1}H} NMR</u> (126 MHz, $CDCl_3$) δ 162.3, 145.2, 143.4, 136.9, 121.8 (2C), 62.5, 32.1, 30.9, 25.6, 14.3.

<u>HRMS</u> (CI/QTOF) m/z: $[M + H]^+$ Calculated for $C_{11}H_{14}NO_3^+$ 208.0968; Found 208.0972.

An 8 mL scintillation vial was charged with 3-(ethoxycarbonyl)-6,7-dihydro-5H-cyclopenta[c]pyridine 2-oxide (0.48 g, 2.3 mmol, 1.0 equiv.) and $POCl_3$ (5 mL). The resulting mixture was stirred for 3 hours at 105 °C. The resulting mixture was cooled down and carefully added to an ice-cold water (100 mL) containing 25 mL aq. NH_3 (25 wt%). The mixture was stirred at 0 °C for 10 minutes and then extracted with DCM (3 × 20 mL). The combined organic layers were washed with brine (50 mL), dried over Na_2SO_4, filtered and concentrated *in vacuo*. The resulting crude was purified via flash column chromatography to give ethyl 1-chloro-6,7-dihydro-5H-cyclopenta[c]pyridine-3-carboxylate (**1u**, 0.23 g, 1.0 mmol, 42 %) as a colorless oil that solidified upon standing.

<u>^{1}H NMR</u> (400 MHz, $CDCl_3$) δ 7.92 (s, 1H, HetAr*H*), 4.45 (q, *J* = 7.1 Hz, 2H, OC*H*$_2$CH$_3$), 3.10 – 2.99 (m, 4H, -C*H*$_2$-CH$_2$-C*H*$_2$-), 2.19 (p, *J* = 7.7 Hz, 2H, -CH$_2$-C*H*$_2$-CH$_2$-), 1.42 (t, *J* = 7.1 Hz, 3H, OCH$_2$C*H*$_3$).

<u>^{13}C{^{1}H} NMR</u> (101 MHz, $CDCl_3$) δ 164.7, 157.8, 147.8, 147.0, 143.3, 120.8, 62.2, 33.7, 31.8, 23.9, 14.5.

<u>HRMS</u> (CI/QTOF) m/z: $[M + H]^+$ Calculated for $C_{11}H_{13}ClNO_2^+$ 226.0629; Found 226.0636.

Methyl 1-chloro-3-phenyl-5,6,7,8-tetrahydroisoquinoline-4-carboxylate (1v)

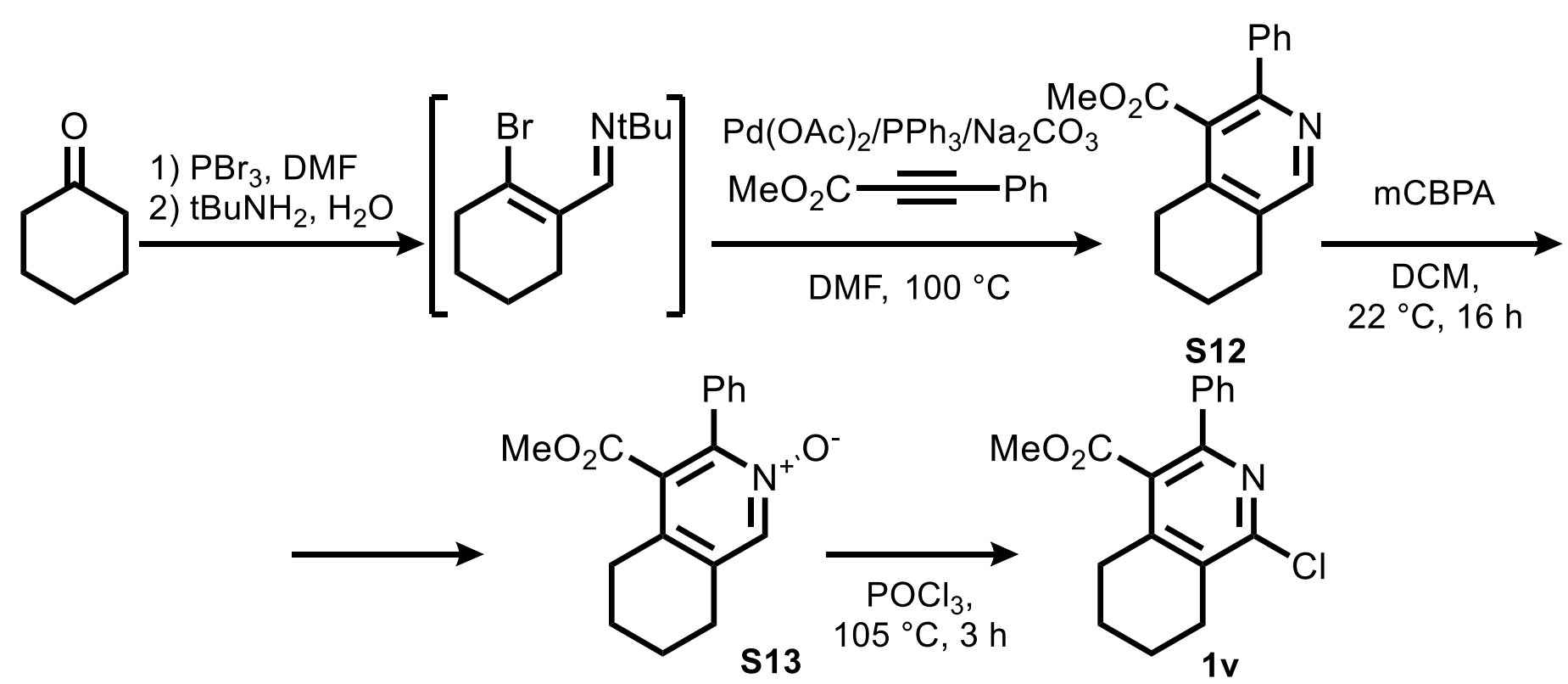

Figure S30. Pd-catalyzed pyridine **1v** synthesis.

According to a reported procedure,(*38*) an oven-dried 250 mL round-bottom flask equipped with a Teflon-coated magnetic stirrer was charged with DMF (2.4 mL) and $CHCl_3$ (20 mL). The mixture was cooled to 0 °C, and PBr_3 (2.6 mL, 7.5 g, 27.6 mmol, 2.3 equiv.) was added. The mixture was warmed to 22 °C and stirred for 30 minutes. Then, cyclohexane (1.24 mL, 1.18 g, 12 mmol, 1.0 equiv.) was added and the mixture was stirred for 16 hours at 60 °C. Solid $NaHCO_3$ was added to neutralize the acids. The organic layer was washed with cold water (20 mL), dried over Na_2SO_4,

filtered and concentrated *in vacuo*. Then, tert-butylamine (4 mL) and water (12 mL) was added and the mixture stirred for additional 16 hours. The solvents were removed in vacuo to give 1-(2-bromocyclohex-1-en-1-yl)-N-(tert-butyl)methanimine (1.0 g, 4.1 mmol, 34 %) which was used further without an additional purification. An oven dried 40 mL scintillation vial equipped with a Teflon coated magnetic stirrer was charged with 1-(2-bromocyclohex-1-en-1-yl)-N-(tert-butyl)methanimine (1.0 g, 4.1 mmol, 1.0 equiv.), methyl 3-phenylpropiolate (1.3 g, 1.2 mL, 8.0 mmol, 2.0 equiv.), $Pd(OAc)_2$ (45 mg, 0.20 mmol, 5 mol %), PPh_3 (105 mg, 0.40 mmol, 10 mol %), Na_2CO_3 (0.42 g, 4.0 mmol, 1.0 equiv.). The vial was evacuated and back-filled with N_2 three times. Then, DMF (20 mL) was added, and the mixture heated at 100 °C for 16 hours. After cooling down the mixture, it was diluted with EtOAc (100 mL) and washed with aq. LiCl (5 wt%, 5 × 20 mL) and brine (50 mL). The organic layer was dried over Na_2SO_4, filtered and concentrated *in vacuo*. The crude material was purified via flash column chromatography (0-40 % EtoAc/Hexanes) to give pyridine **S12** as a pale-yellow oil (0.27 g, 1.0 mmol, 25 % yield).
^{1}H NMR (500 MHz, $CDCl_3$) δ 8.43 (s, 1H, N-C*H*), 7.59 – 7.54 (m, 2H, Ar*H*), 7.45 – 7.35 (m, 3H, Ar*H*), 3.65 (s, 3H, OCH_3), 2.84 – 2.76 (m, 4H, -CH_2-CH_2-CH_2-CH_2-), 1.87 – 1.82 (m, 4H, -CH_2-CH_2-CH_2-CH_2-).
HRMS (CI/QTOF) m/z: $[M + H]^+$ Calculated for $C_{17}H_{18}NO_2^+$ 268.1332; Found 268.1330.
Spectral data matched those reported in the literature.(*38*)

A 100 mL round bottom flask was charged with methyl 3-phenyl-5,6,7,8-tetrahydroisoquinoline-4-carboxylate (0.27 g, 1.0 mmol, 1.0 equiv.), meta-chloroperoxybenzoic acid (0.70 g, 3.0 mmol, 3.0 equiv., 75 % mass purity) and DCM (10 mL). The resulting mixture was stirred at 22 °C for 16 hours. Then, silica was directly added, and the mixture dry loaded for flash column chromatography (SiO_2, 0-10% EtOAc/MeOH) to give 4-(methoxycarbonyl)-3-phenyl-5,6,7,8-tetrahydroisoquinoline 2-oxide (**S13**, 0.22 g, 0.77 mmol, 77 %).
^{1}H NMR (400 MHz, $CDCl_3$) δ 8.19 (s, 1H, N-C*H*), 7.53 – 7.33 (m, 5H, Ar*H*), 3.51 (s, 3H, OCH_3), 2.79 – 2.72 (m, 2H, , -CH_2-CH_2-CH_2-CH_2-), 2.72 – 2.62 (m, 2H, , -CH_2-CH_2-CH_2-CH_2-), 1.90 – 1.77 (m, 4H, , -CH_2-CH_2-CH_2-CH_2-).
^{13}C NMR (101 MHz, $CDCl_3$) δ 166.4, 144.7, 139.9, 135.5, 134.1, 133.2, 131.2, 129.6 (2C), 128.4, 52.6, 26.56, 25.9, 22.1, 21.6.
HRMS (APCI/QTOF) m/z: $[M + H]^+$ Calculated for $C_{17}H_{18}NO_3^+$ 284.1281; Found 284.1285.

An 8 mL scintillation vial was charged with 4-(methoxycarbonyl)-3-phenyl-5,6,7,8-tetrahydroisoquinoline 2-oxide (0.22 g, 0.77 mmol, 1.0 equiv.) and $POCl_3$ (2 mL). The resulting mixture was stirred for 3 hours at 105 °C. The resulting mixture was cooled down and carefully added to an ice-cold water (100 mL) containing 10 mL aq. NH_3 (25 wt%). The mixture was stirred at 0 °C for 10 minutes and then extracted with DCM (3 × 20 mL). The combined organic layers were washed with brine (50 mL), dried over Na_2SO_4, filtered and concentrated *in vacuo*. The resulting crude was purified via flash column chromatography to give methyl 1-chloro-3-phenyl-5,6,7,8-tetrahydroisoquinoline-4-carboxylate (**1v**, 0.12 g, 0.40 mmol, 52 %) as a colorless oil.
^{1}H NMR (400 MHz, $CDCl_3$) δ 7.70 – 7.48 (m, 2H, Ar*H*), 7.46 – 7.33 (m, 3H, Ar*H*), 3.65 (s, 3H, OCH_3), 2.88 – 2.69 (m, 4H, -CH_2-CH_2-CH_2-CH_2-), 1.99 – 1.70 (m, 4H, -CH_2-CH_2-CH_2-CH_2-).
^{13}C{^{1}H} NMR (101 MHz, $CDCl_3$) δ 169.0, 153.3, 152.4, 147.7, 138.8, 130.3, 129.0, 128.6, 128.3, 127.8, 52.5, 27.5, 27.0, 22.2, 21.8.
HRMS (APCI/QTOF) m/z: $[M + H]^+$ Calculated $C_{17}H_{17}ClNO_2^+$ 302.0942; Found 302.0947.

Ethyl 6-chloro-2,5-dimethyl-4-phenylnicotinate (1w)

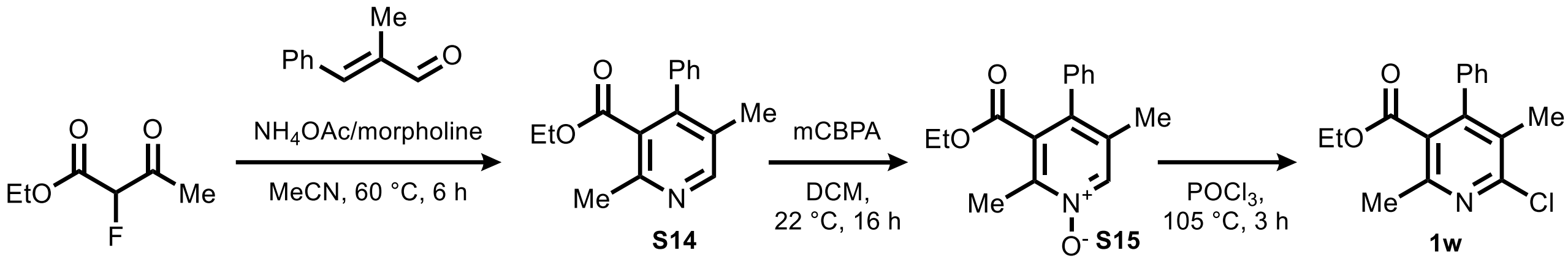

Figure S31. 3-Component pyridine condensation reaction.

According to a reported procedure,(39) an oven-dried 250 mL round-bottom flask equipped with a Teflon-coated magnetic stirrer was charged with ethyl 2-fluoro-3-oxobutanoate (2.7 mL, 20 mmol, 1.0 equiv.), (E)-2-methyl-3-phenylacrylaldehyde (2.8 mL, 20 mmol, 1.0 equiv.), ammonium acetate (3.08 g, 40 mmol, 2.00 equiv.) and MeCN (100 mL). The resulting mixture was stirred for 6 hours at 60 °C. After cooling down, the resulting mixture was diluted with EtOAc (200 mL) and washed with water (100 mL) and brine (100 mL). The organic layer was dried over Na_2SO_4 and concentrated *in vacu*o. The crude material was purified via flash column chromatography (20-100 % EtoAc/Hexanes) to give amide **S14** as a brown oil (2.5 g, 10 mmol, 50 % yield). This material was used in further steps without an addition purification.

^{1}H NMR (500 MHz, $CDCl_3$) δ 8.43 (s, 1H, HetAr*H*), 7.47 – 7.30 (m, 3H, Ar*H*), 7.20 – 7.06 (m, 2H, Ar*H*), 3.97 (q, *J* = 7.1 Hz, 2H, OCH_2CH_3), 2.56 (s, 3H, HetArCH_3), 2.09 (s, 3H, HetArCH_3), 0.90 (t, *J* = 7.1 Hz, 3H, OCH_2CH_3).

HRMS (CI/QTOF) m/z: $[M + H]^+$ Calculated for $C_{16}H_{18}NO_2^+$ 256.1332; Found 256.1338.

Spectral data matched those reported in the literature.(39)

A 100 mL round bottom flask was charged with ethyl 2,5-dimethyl-4-phenylnicotinate (2.5g, 10 mmol, 1.0 equiv.), meta-chloroperoxybenzoic acid (3.5 g, 15 mmol, 1.5 equiv., 75 % mass purity) and DCM (50 mL). The resulting mixture was stirred at 22 °C for 16 hours. Then, silica was directly added, and the mixture dry loaded for flash column chromatography (SiO_2, 0-10% EtOAc/MeOH) to give 3-(ethoxycarbonyl)-2,5-dimethyl-4-phenylpyridine 1-oxide (**S15**, 1.8 g, 6.6 mmol, 66 %).

^{1}H NMR (400 MHz, $CDCl_3$) δ 8.26 (s, 1H, HetAr*H*), 7.48 – 7.33 (m, 3H, Ar*H*), 7.24 – 7.15 (m, 2H, Ar*H*), 3.99 (q, *J* = 7.1 Hz, 2H, OCH_2CH_3), 2.51 (s, 3H, HetArCH_3), 2.07 (s, 3H, HetArCH_3), 0.91 (t, *J* = 7.1 Hz, 3H, OCH_2CH_3).

$^{13}C\{^1H\}$ NMR (101 MHz, $CDCl_3$) δ 166.1, 144.0, 139.8, 135.7, 133.0, 132.6, 128.8, 128.7, 128.6 (2C), 62.0, 17.3, 15.1, 13.7.

HRMS (APCI/QTOF) m/z: $[M + H]^+$ Calculated for $C_{16}H_{18}NO_3^+$ 272.1281; Found 272.1287.

An 8 mL scintillation vial was charged with 3-(ethoxycarbonyl)-2,5-dimethyl-4-phenylpyridine 1-oxide (0.50 g, 1.8 mmol, 1.0 equiv.) and POCl3 (5 mL). The resulting mixture was stirred for 3 hours at 105 °C. The resulting mixture was cooled down and carefully added to an ice-cold water (100 mL) containing 25 mL aq. NH_3 (25 wt%). The mixture was stirred at 0 °C for 10 minutes and then extracted with DCM (3 × 20 mL). The combined organic layers were washed with brine (50 mL), dried over Na_2SO_4, filtered and concentrated *in vacuo*. The resulting crude was purified via flash column chromatography to give ethyl 6-chloro-2,5-dimethyl-4-phenylnicotinate (**1w**, 0.25 g, 0.85 mmol, 47 %) as a colorless oil.

^{1}H NMR (400 MHz, $CDCl_3$) δ 7.50 – 7.30 (m, 3H, Ar*H*), 7.21 – 7.10 (m, 2H, Ar*H*), 3.95 (q, *J* = 7.1 Hz, 2H, OCH_2CH_3), 2.53 (s, 3H, HetArCH_3), 2.13 (s, 3H, HetArCH_3), 0.88 (t, *J* = 7.1 Hz, 3H, OCH_2CH_3).

$^{13}C\{^1H\}$ NMR (101 MHz, $CDCl_3$) δ 167.8, 152.7, 151.9, 150.2, 137.0, 129.1, 128.5 (2C), 128.4, 128.0, 61.5, 22.3, 17.1, 13.7.

HRMS (APCI/QTOF) m/z: $[M + H]^+$ Calculated $C_{16}H_{17}ClNO_2^+$ 290.0942; Found 290.0941.

Methyl 2-chloro-6-isopropylisonicotinate (1z)

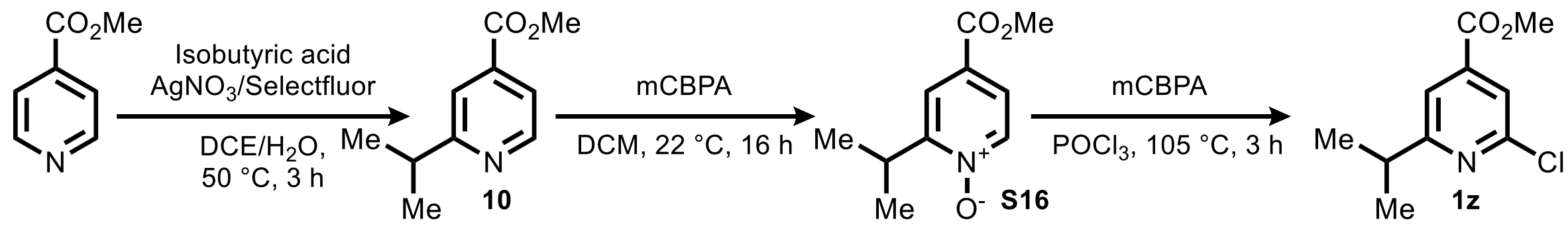

Figure S32. Minisci reaction towards pyridine **1z**.

Based on a reported procedure,(46) a 100 mL round-bottom flask equipped with a Teflon coated magnetic stirrer was charged with methyl isonicotinate (1.3 mL, 1.5 g, 10.9 mmol, 1.0 equiv.), $AgNO_3$ (370 mg, 2.18 mmol, 0.20 equiv.), Selectfluor (7.70 g, 21.8 mmol, 2.0 equiv.)), DCE (30 mL) and water (30 mL). Then, TFA (0.83 mL, 1.24 g, 10.9 mmol, 1.00 equiv.) and isobutyric acid (2.02 mL, 1.92 g, 21.8 mmol, 2.00 equiv.) were added and the mixture stirred at 50 °C for 3 h. The mixture was allowed to cool down, diluted with EtOAc (100 mL) and washed with NaOH (1M, 3 × 50 mL) and brine (50 mL). The organic layer was dried over Na_2SO_4, filtered and concentrated *in vacuo*. The crude mixture was purified via preparative TLC (100% EtOAc) to give pyridine **10** (635 mg, 3.55 mmol, 33 % yield).
^{1}H NMR (400 MHz, $CDCl_3$) δ 8.68 (dd, *J* = 5.1, 1.2 Hz, 1H, N-C*H*), 7.73 (t, *J* = 1.2 Hz, 1H, N-C(*i*Pr)-C*H*), 7.64 (dd, *J* = 5.1, 1.2 Hz, 1H, N-CH-C*H*), 3.95 (s, 3H, OC*H*$_3$), 3.14 (p, *J* = 6.9 Hz, 1H, C*H*(CH$_3$)$_2$), 1.33 (d, *J* = 6.9 Hz, 6H, CH(C*H*$_3$)$_2$).
^{13}C{^{1}H} NMR (101 MHz, $CDCl_3$) δ 168.7, 166.2, 150.0, 137.9, 120.4, 120.1, 52.7, 36.6, 22.6.
HRMS (APCI/QTOF) m/z: $[M + H]^+$ Calculated $C_{10}H_{14}NO_2^+$ 180.1019; Found 180.1015.

A 100 mL round bottom flask was charged with methyl 2-isopropylisonicotinate (0.64 g, 3.6 mmol, 1.0 equiv.), meta-chloroperoxybenzoic acid (1.6 g, 7.2 mmol, 2.0 equiv., 75 % mass purity) and DCM (20 mL). The resulting mixture was stirred at 22 °C for 16 hours. Then, silica was directly added, and the mixture dry loaded for flash column chromatography (SiO_2, 0-10% EtOAc/MeOH) to give 2-isopropyl-4-(methoxycarbonyl)pyridine 1-oxide (**S16**, 0.65 g, 3.3 mmol, 92 %).
^{1}H NMR (400 MHz, $CDCl_3$) δ 8.24 (d, *J* = 6.8 Hz, 1H, N-C*H*), 7.85 (d, *J* = 2.5 Hz, 1H, N-C(*i*Pr)-C*H*), 7.70 (dd, *J* = 6.8, 2.5 Hz, 1H, N-CH-C*H*), 3.94 (s, 3H, OC*H*$_3$), 3.72 (p, *J* = 6.9 Hz, 1H, C*H*(CH$_3$)$_2$), 1.31 (d, *J* = 6.9 Hz, 6H, CH(C*H*$_3$)$_2$).
^{13}C{^{1}H} NMR (101 MHz, $CDCl_3$) δ 164.6, 158.2, 140.0, 126.2, 123.3, 123.2, 52.8, 27.8, 20.2.
HRMS (APCI/QTOF) m/z: $[M + H]^+$ Calculated for $C_{10}H_{14}NO_3^+$ 196.0968; Found 196.0971.

An 8 mL scintillation vial was charged with 2-isopropyl-4-(methoxycarbonyl)pyridine 1-oxide (0.65 g, 3.3 mmol, 1.0 equiv.) and $POCl_3$ (6 mL). The resulting mixture was stirred for 3 hours at 105 °C. The resulting mixture was cooled down and carefully added to an ice-cold water (100 mL) containing 30 mL aq. NH_3 (25 wt%). The mixture was stirred at 0 °C for 10 minutes and then extracted with DCM (3 × 20 mL). The combined organic layers were washed with brine (50 mL), dried over Na_2SO_4, filtered and concentrated *in vacuo*. The resulting crude was purified via flash column chromatography to give methyl 2-chloro-6-isopropylisonicotinate (**1z**, 0.35 g, 1.6 mmol, 48 %) as a colorless oil.
^{1}H NMR (400 MHz, $CDCl_3$) δ 7.68 (d, *J* = 1.2 Hz, 1H, HetAr*H*), 7.64 (d, *J* = 1.2 Hz, 1H, HetAr*H*), 3.95 (s, 3H, OC*H*$_3$), 3.11 (p, *J* = 6.9 Hz, 1H, C*H*(CH$_3$)$_2$), 1.32 (d, *J* = 7.0 Hz, 6H, CH(C*H*$_3$)$_2$).
^{13}C{^{1}H} NMR (101 MHz, $CDCl_3$) δ 169.9, 164.9, 151.6, 140.7, 121.3, 118.7, 53.0, 36.4, 22.4.
HRMS (APCI/QTOF) m/z: $[M + H]^+$ Calculated $C_{10}H_{13}ClNO_2^+$ 214.0629; Found 214.0630.

Methyl 2-chloro-isonicotinate (1b)

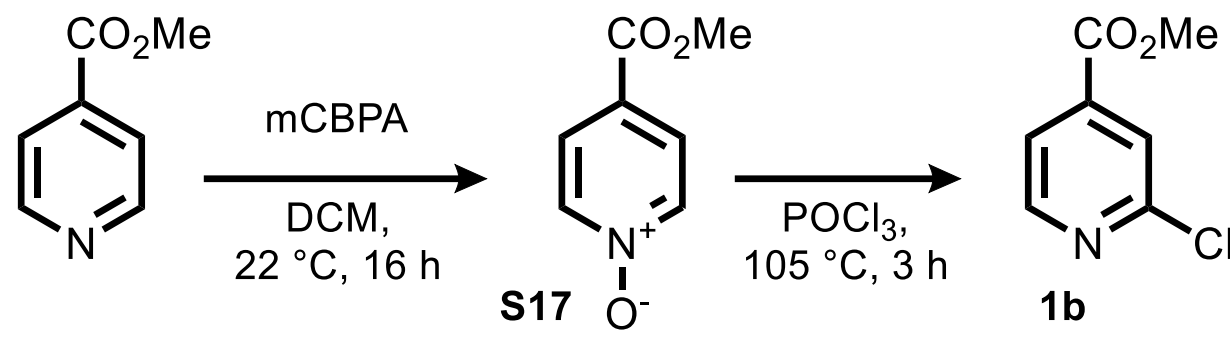

Figure S33. Chlorination of methyl isonicotinate.

A 20 mL scintillation equipped with a Teflon coated magnetic stirrer was charged with methyl isonicotinate (0.90 mL, 1.05 g, 7.7 mmol, 1.0 equiv.), meta-chloroperoxybenzoic acid (3.4 g, 14.8 mmol, 1.9 equiv., 75 % mass purity) and DCM (10 mL). The resulting mixture was stirred at 22 °C for 16 hours. Then, silica was directly added, and the mixture dry loaded for flash column chromatography (SiO_2, 0-10% EtOAc/MeOH) to give 4-(methoxycarbonyl)pyridine 1-oxide (**S17**, 1.0 g, 6.7 mmol, 87 %).

^{1}H NMR (400 MHz, $CDCl_3$) δ 8.22 (d, *J* = 7.2 Hz, 2H, 2×N-CH-C*H*), 7.87 (d, *J* = 7.2 Hz, 2H, 2×N-C*H*-CH), 3.95 (s, 3H, OCH_3).

^{13}C{^{1}H} NMR (101 MHz, $CDCl_3$) δ 164.0, 139.6, 126.6 (2C), 53.0.

Spectral data matched those reported in the literature.(*58*)

An 8 mL scintillation vial was charged with 4-(methoxycarbonyl)pyridine 1-oxide (0.5 g, 3.3 mmol, 1.0 equiv.) and $POCl_3$ (5 mL). The resulting mixture was stirred for 3 hours at 105 °C. The resulting mixture was cooled down and carefully added to an ice-cold water (100 mL) containing 25 mL aq. NH_3 (25 wt%). The mixture was stirred at 0 °C for 10 minutes and then extracted with DCM (3 × 20 mL). The combined organic layers were washed with brine (50 mL), dried over Na_2SO_4, filtered and concentrated *in vacuo*. The resulting crude was purified via flash column chromatography to give methyl 2-chloro-isonicotinate (**1b**, 0.35 g, 2.0 mmol, 61 %) as a colorless oil that solidified upon standing.

^{1}H NMR (400 MHz, $CDCl_3$) δ 8.55 (dd, *J* = 5.1, 0.8 Hz, 1H, N-C*H*), 7.89 (dd, *J* = 1.4, 0.8 Hz, 1H, HetAr*H*), 7.77 (dd, *J* = 5.0, 1.4 Hz, 1H, N-CH-C*H*), 3.97 (s, 3H, OC*H*$_3$).

^{13}C{^{1}H} NMR (101 MHz, $CDCl_3$) δ 164.5, 152.6, 150.7, 140.4, 124.2, 121.7, 53.2.

Spectral data matched those of an authentic standard.

Alternative synthesis for large scale preparation of pyridine 8:

3-Bromo-*N*,*N*-diethylisonicotinamide (S18)

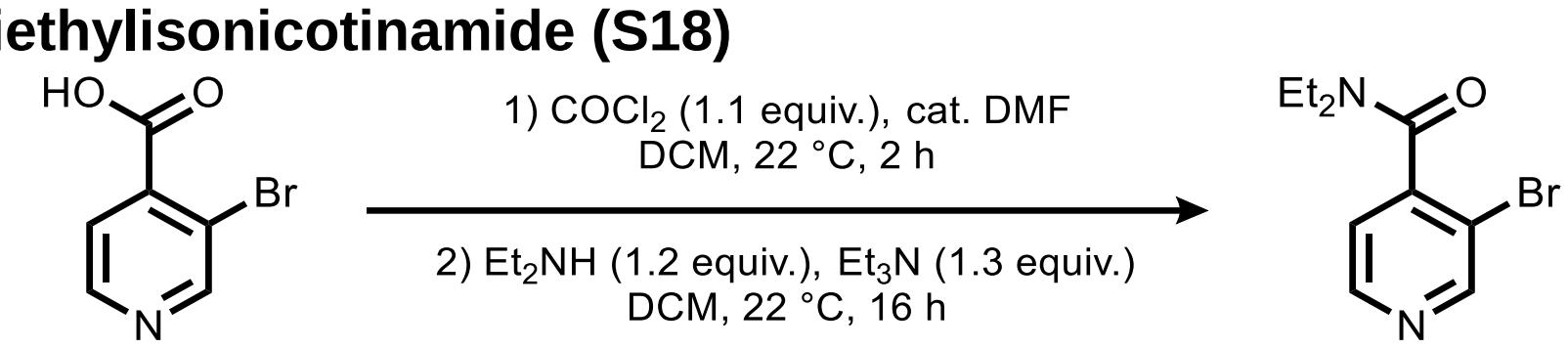

Figure S34. Amide bond coupling towards **S18**.

An oven-dried 250 mL round-bottom flask was charged with 3-bromoisonicotinic acid (10.0 g, 49.5 mmol, 1.0 equiv.) and DCM (100 mL). Then, oxalyl chloride (4.6 mL, 55 mmol, 1.1 equiv.) was added followed by 2 drops of DMF. The resulting mixture was stirred open to air for 2 hours at 22 °C. Then, triethyl amine (8.3 mL, 60 mmol, 1.2 equiv.) was slowly added followed by diethyl amine (6.8 mL, 65 mmol, 1.3 equiv.). The reaction mixture was stirred at 22 °C for 16 hours. The resulting mixture was washed with aq. NaOH (1 M, 3 × 50 mL) and brine (50 mL). The organic layer was dried over Na_2SO_4 and concentrated *in vacuo* to give amide **S18** as a brown oil (6.5 g, 25 mmol, 51 % yield). This material was used in further steps without an addition purification.

^{1}H NMR (400 MHz, Chloroform-*d*) δ 8.76 (d, *J* = 0.7 Hz, 1H, N-C*H*-CBr), 8.58 (d, *J* = 4.9 Hz, 1H, N-C*H*-CH), 7.20 (dd, *J* = 4.9, 0.7 Hz, 1H, N-CH-C*H*), 3.80 (br. s, 1H, $N(C\mathit{H}_aH_bCH_3)_A$), 3.35 (br. s, 1H, $N(C\mathit{H}_aH_bCH_3)_B$), 3.13 (br. s, 2H, $N(CH_a\mathit{H}_bCH_3)_A$ and $N(CH_a\mathit{H}_bCH_3)_B$), 1.27 (t, *J* = 7.1 Hz, 3H, $N(CH_aH_bC\mathit{H}_3)_A$), 1.09 (t, *J* = 7.1 Hz, 3H, $N(CH_aH_bC\mathit{H}_3)_B$).
$^{13}C\{^1H\}$ NMR (101 MHz, Chloroform-*d*) δ 166.1, 152.2, 148.5, 146.3, 122.0, 117.7, 42.8, 39.2, 14.1, 12.6.
HRMS (CI/QTOF) m/z: $[M + H]^+$ Calculated for $C_{10}H_{14}BrN_2O^+$ 257.0284; Found 257.0292.

_N_,_N_-diethyl-3-(prop-1-en-2-yl)isonicotinamide (S19)

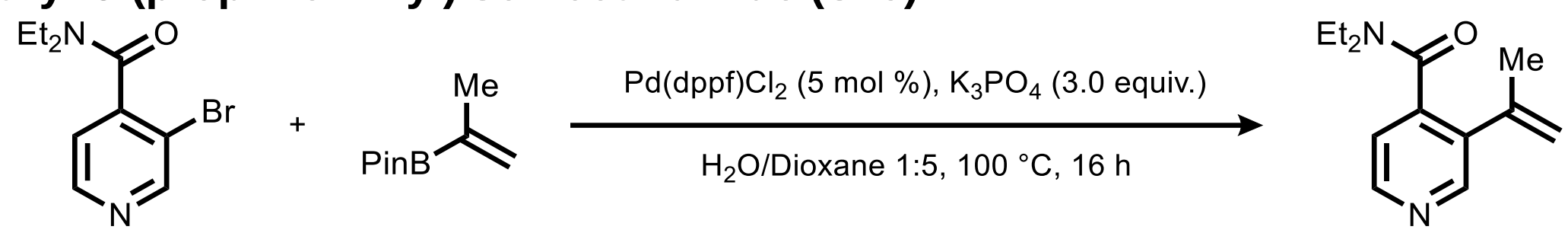

Figure S35. Pd-catalyzed alkenylation.

An oven-dried 40 mL screw-cap vial was charged with $Pd(dppf)Cl_2$ (365 mg, 0.500 mmol, 5 mol%), K3PO4 (6.4 g, 30 mmol, 3.0 equiv.) and amide **S18** (2.57 g, 10.0 mmol, 1.0 equiv.). The vial was evacuated and back-filled with N_2 three times. Then, dioxane (25 mL) and water (5 mL) were added, followed by 4,4,5,5-tetramethyl-2-(prop-1-en-2-yl)-1,3,2-dioxaborolane (2.76 mL, 2.52 g, 15.0 mmol, 1.50 equiv.). The resulting mixture was stirred at 100 °C for 16 hours. Then, the mixture was cooled down to room temperature and diluted with EtOAc (50 mL). The mixture was washed with water (2 × 50 mL) and brine (50 mL). The organic layer was was dried over Na_2SO_4 and concentrated *in vacuo*. The crude product was purified via column chromatography (SiO_2, 50 to 100 % EtOAc/Hexanes) to give amide **S19** as a brown oil (1.6 g, 7.3 mmol, 73 % yield).
^{1}H NMR (400 MHz, $CDCl_3$) δ 8.56 (d, *J* = 0.7 Hz, 1H, N-C*H*), 8.54 (d, *J* = 4.9 Hz, 1H, N-C*H*), 7.13 (dd, *J* = 4.9, 0.7 Hz, 1H, N-CH-C*H*), 5.24 (app. pent, *J* = 1.6 Hz, 1H, $C{=}CH_aH_b$), 5.13 (app. t, *J* = 1.2 Hz, 1H, $C{=}CH_aH_b$), 3.54 (br. s, 2H, rot. $C\mathit{H}_2CH_3$ and $C\mathit{H}(CH_3)_2$), 3.06 (q, *J* = 7.1 Hz, 2H, rot. $C\mathit{H}_2CH_3$), 2.11 (dd, *J* = 1.6, 0.9 Hz, 3H, $C\mathit{H}_3$), 1.23 (s, 6H, $CH(C\mathit{H}_3)_2$), 1.20 (t, *J* = 7.1 Hz, 4H), 1.04 (t, *J* = 7.1 Hz, 3H).
$^{13}C\{^1H\}$ NMR (101 MHz, $CDCl_3$) δ 168.5, 149.4, 148.8, 142.7, 140.6, 135.0, 120.7, 117.9, 42.8, 38.7, 25.0, 23.5, 13.9, 12.4.
HRMS (CI/QTOF) m/z: $[M + H]^+$ Calculated for $C_{13}H_{18}N_2O^+$ 219.1492; Found 219.1483.

_N_,_N_-diethyl-3-isopropylisonicotinamide (8)

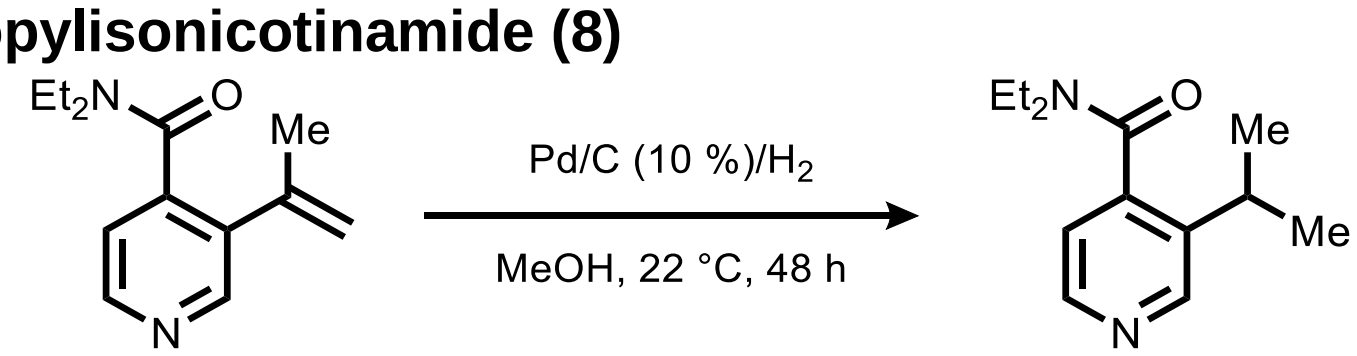

Figure S36. Pd-catalyzed hydrogenation.

A 250 mL round-bottom flask was charged with amide **S19** (1.6 g, 7.3 mmol, 1.0 equiv.) and Pd/C (10 wt%, 778 mg, 10 mol %). The flask was evacuated and back-filled with N_2 three times. Then, MeOH (100 mL) was added, followed and the mixture sparged with N_2 followed by H_2 for 5 minutes each. The resulting mixture was stirred under the atmosphere of H_2 (balloon) for 48 h. Then, the mixture was filtered through a pad of celite washing with EtOAc (150 mL). The filtrate concentrated *in vacuo* to give amide **8** as a pale-yellow oil (1.6.g, 7.3 mmol, 99 % yield).
^{1}H NMR (400 MHz, $CDCl_3$) δ 8.63 (s, 1H, N-C*H*), 8.47 (d, *J* = 4.9 Hz, 1H, N-C*H*), 7.04 (d, *J* = 4.9, 1H, N-CH-C*H*), 3.84 – 3.67 (m, 1H, rot. $C\mathit{H}_aH_bCH_3$), 3.47 – 3.32 (m, 1H, $CH_a\mathit{H}_bCH_3$), 3.20 – 3.02

(m, 2H, 2×rot. $CH_aH_bCH_3$), 2.94 (hept., J = 6.9 Hz, 1H, $CH(CH_3)_2$), 1.31 (d, J = 6.9 Hz, 6H, $CH(CH_3)_2$), 1.26 (t, J = 7.1 Hz, 3H, rot. CH_2CH_3), 1.07 (t, J = 7.1 Hz, 3H, CH_2CH_3).
<u>^{13}C{^{1}H} NMR</u> (101 MHz, $CDCl_3$) δ 168.4, 148.9, 147.4, 143.6, 139.5, 119.8, 42.9, 38.8, 29.5, 25.0, 23.4, 23.0, 14.1, 12.9.
<u>HRMS</u> (APCI/QTOF) m/z: $[M + H]^+$ Calculated for 221.1648 ; Found 221.1639.

_N,N_-diethyl-2-fluoro-3-isopropylisonicotinamide (1x) and _N,N_-diethyl-2-fluoro-5-isopropylisonicotinamide (1y)

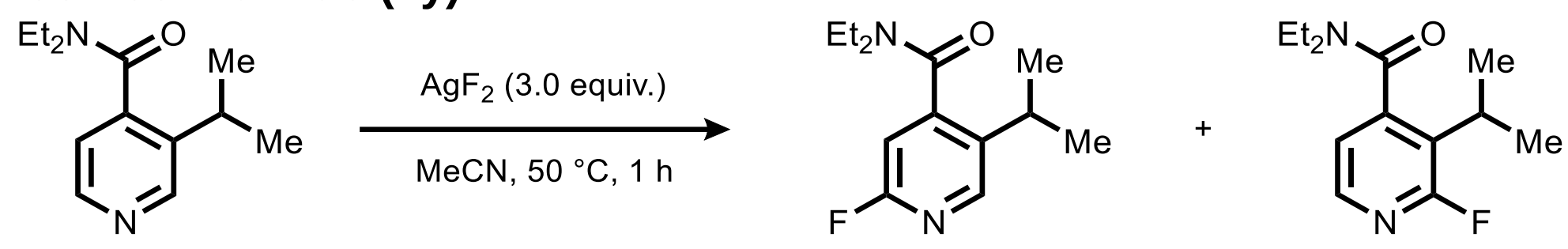

Figure S37. Fluorination of isonicotinic amide **8**.

An oven-dried 250 mL round-bottom flask was charged with amide **8** (0.5 g, 2.3 mmol, 1.0 equiv.), AgF2 (994 mmol, 6.8 mmol, 3.0 equiv.) and MeCN (91 mL). The resulting mixture was stirred for 1 h at 50 °C. The resulting mixture was cooled down to room temperature and filtered through a short pad of silica, eluting with EtOAc (150 mL). The filtrate concentrated *in vacuo*. The crude product was purified via column chromatography (SiO_2, 20 to 100 % EtOAc/Hexanes) to give amide **1x** as a pale yellow oil (58 mg, 0.24 mmol, 11 % yield) and amide **1y** as a pale yellow oil (340 mg, 1.43 mmol, 63 % yield).

1x – elutes first
<u>^{1}H NMR</u> (600 MHz, CDCl3) δ 8.20 (s, 1H, $NCHCCH(CH_3)_2$), 6.69 (d, J = 2.9 Hz, 1H, NCFCH), 3.75 (br. s, 1H, $N(CH_aH_bCH_3)_A$), 3.40 (br. s, 1H, $N(CH_aH_bCH_3)_B$), 3.16 – 3.07 (m, 2H, $N(CH_aH_bCH_3)_A$ and $N(CH_aH_bCH_3)_B$), 2.91 (hept, J = 6.9 Hz, 1H, $CH(CH_3)_2$), 1.29 (d, J = 6.9 Hz, 6H, $CH(CH_3)_2$), 1.26 (t, J = 7.1 Hz, 3H, $N(CH_aH_bCH_3)_A$),), 1.09 (t, J = 7.1 Hz, 3H, $N(CH_aH_bCH_3)_B$),).
<u>^{19}F NMR</u> (565 MHz, $CDCl_3$) δ -71.2 (d, J = 3.0 Hz).
<u>^{13}C{^{1}H} NMR</u> (151 MHz, $CDCl_3$) δ 167.2 (d, J = 3.3 Hz), 162.0 (d, J = 238.3 Hz), 148.9 (d, J = 6.5 Hz), 146.6 (d, J = 14.6 Hz), 137.6 (d, J = 4.8 Hz), 105.6 (d, J = 38.6 Hz), 42.9, 38.9, 28.9, 24.5, 23.1, 14.1, 12.8.
<u>HRMS</u> (APCI/QTOF) m/z: $[M + H]^+$ Calculated for $C_{13}H_{20}FN_2O^+$ 239.1554; Found 239.1550.

1y- elutes second
<u>^{1}H NMR</u> (600 MHz, $CDCl_3$) δ 8.07 (dd, J = 5.0, 1.2 Hz, 1H, N-CH), 6.94 (dd, J = 5.0, 1.5 Hz, 1H, N-CH-CH), 3.77 – 3.67 (m, 1H, rot. $N(CH_aH_bCH_3)_2$), 3.48 – 3.36 (m, 1H, rot. $N(CH_aH_bCH_3)_2$), 3.20 – 3.06 (m, 2H, 2×rot. $N(CH_aH_bCH_3)_2$), 2.91 (p, J = 7.0 Hz, 1H), 1.34 (app. dd, J = 7.1, 1.4 Hz, 6H, rot. $CH(CH_3)_2$), 1.26 (t, J = 7.1 Hz, 3H, rot. CH_2CH_3), 1.09 (t, J = 7.1 Hz, 3H, rot. CH_2CH_3).
<u>^{19}F NMR</u> (565 MHz, $CDCl_3$) δ -64.7.
<u>^{13}C{^{1}H} NMR</u> (151 MHz, $CDCl_3$) δ 167.4 (d, J_{C-F} = 4.2 Hz), 162.7 (d, J_{C-F} = 242.7 Hz), 148.6 (d, J_{C-F} = 6.8 Hz), 145.3 (d, J_{C-F} = 16.0 Hz), 125.9 (d, J_{C-F} = 28.8 Hz), 118.5 (d, J_{C-F} = 4.3 Hz), 42.9, 38.9, 29.4 (d, J_{C-F} = 4.2 Hz), 21.3 (d, J_{C-F} = 2.7 Hz), 20.1 (d, J_{C-F} = 4.2 Hz), 14.1, 12.8.
<u>HRMS</u> (APCI/QTOF) m/z: $[M + H]^+$ Calculated for $C_{13}H_{20}FN_2O+$ 239.1554; Found 239.1554.

2-chloro-4-(trifluoromethyl)-5,6,7,8-tetrahydroquinoline-^{15}N (^{15}N-1r)

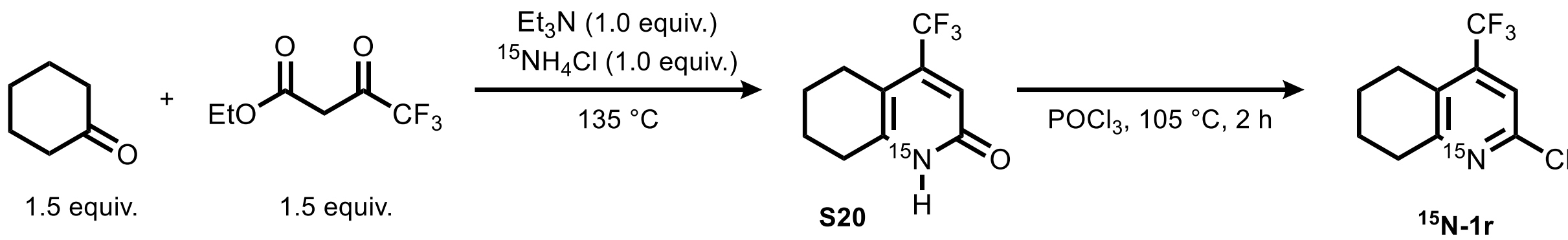

Figure S38. Synthesis of labeled substrate **^{15}N-1r**.

An oven-dried 8 mL scintillation vial equipped with a Teflon-coated magnetic stir-bar was charged with $^{15}NH_4Cl$ (0.43 g, 7.9 mmol, 1.0 equiv.), cyclohexanone (1.2 mL, 1.2 g, 12 mmol, 1.5 equiv.), ethyl 4,4,4-trifluoroacetoacetate (2.9 mL, 3.68 g, 20 mmol, 1.0 equiv.) and Et_3N (1.7 mL,1.2 g, 12 mmol, 1.0 equiv.). The resulting mixture was heated at 100 ° for 16 h. The resulting mixture was cooled down and Et_2O (15 mL) was added. The resulting suspension was filtered, washing with more Et_2O (15 mL), and then H_2O (2×10 mL) to give 4-(trifluoromethyl)-5,6,7,8-tetrahydroquinolin-2(1H)-one-^{15}N (**S20**, 0.63 g, 2.7 mmol, 34 %) as a white solid.

^{1}H NMR (400 MHz, $CDCl_3$) δ 13.24 (br. s, 1H, N*H*), 6.74 (d, $J_{N\text{-}H}$ = 2.3 Hz, 1H, HetAr*H*), 2.74 (t, J = 6.0 Hz, 2H, CH_2-CH_2-CH_2-CH_2), 2.60 (t, J = 5.9 Hz, 2H, CH_2-CH_2-CH_2-CH_2), 1.96 – 1.68 (m, 4H, CH_2-CH_2-CH_2-CH_2).
^{19}F NMR (376 MHz, $CDCl_3$) δ -64.7 (s, 3F, CF_3).
^{13}C NMR (101 MHz, $CDCl_3$) δ 164.0 (d, $J_{C\text{-}N}$ = 12.3 Hz), 146.0 (d, $J_{C\text{-}N}$ = 11.5 Hz), 142.7 (q, $J_{C\text{-}F}$ = 30.7 Hz), 122.8 (q, $J_{C\text{-}F}$ = 275.8 Hz), 117.2 – 114.8 (m), 111.4, 27.5 (d, $J_{C\text{-}N}$ = 1.8 Hz), 23.0, 22.3, 21.2 (d, $J_{C\text{-}N}$ = 1.6 Hz).
^{15}N NMR (41 MHz, $CDCl_3$) δ 177.2 (extracted from ^{1}H-^{15}N HMBC).
HRMS (CI/QTOF) m/z: $[M + H]^+$ Calculated for $C_{10}H_{11}F_3{}^{15}NO^+$ 219.0758; Found 219.0762.
^{15}N enrichment calculated according to McNally et al(*52*) to be >99 %

An oven-dried 8 mL scintillation vial equipped with a Teflon-coated magnetic stir-bar was charged with 4-(trifluoromethyl)-5,6,7,8-tetrahydroquinolin-2(1H)-one-^{15}N (0.50 g, 2.1 mmol, 1.0 equiv.) and $POCl_3$ (20 mL). The resulting mixture was heated at 105 °C for 2 h. The resulting mixture was cooled down and poured into ice cold water (100 mL). Then, aq. ammonia (25 %, 25 mL) was slowly added. The resulting mixture was extracted with DCM (3 × 20 mL). The combined organic layers were washed with brine (50 mL), dried over Na_2SO_4, filtered and concentrated *in vacuo* to give 2-chloro-4-(trifluoromethyl)-5,6,7,8-tetrahydroquinoline-^{15}N (**^{15}N-1r** , 0.47 g, 2.0 mmol, 95 %). This material was used without further purification.

^{1}H NMR (400 MHz, $CDCl_3$) δ 7.35 (s, 1H, HetAr*H*), 2.98 (t, J = 6.4 Hz, 2H, CH_2-CH_2-CH_2-CH_2), 2.88 (t, J = 6.5 Hz, 2H, CH_2-CH_2-CH_2-CH_2), 1.97 – 1.75 (m, 4H, CH_2-CH_2-CH_2-CH_2).
^{19}F NMR (376 MHz, $CDCl_3$) δ -63.8 (s, 3F, CF_3).
^{13}C{^{1}H} NMR (101 MHz, $CDCl_3$) δ 160.9, 148.5 (d, $J_{C\text{-}N}$ = 5.9 Hz), 139.2 (qd, $J_{C\text{-}F/C\text{-}N}$ = 31.5, 1.8 Hz), 129.1, 122.8 (q, $J_{C\text{-}F}$ = 275.3 Hz), 118.3 (qd, $J_{C\text{-}F/C\text{-}N}$ = 5.8, 2.4 Hz), 33.1 (d, $J_{C\text{-}N}$ = 8.9 Hz), 24.9 (q, $J_{C\text{-}F}$ = 2.3 Hz), 22.2 (d, $J_{C\text{-}N}$ = 2.7 Hz), 22.0.
^{15}N NMR (41 MHz, $CDCl_3$) δ 314.1 (extracted from ^{1}H-^{15}N HMBC).
HRMS (CI/QTOF) m/z: $[M + H]^+$ Calculated for $C_{10}H_{10}ClF_3{}^{15}N^{++}$ 237.0419; Found 237.0407.
^{15}N enrichment calculated according to McNally et al(*52*) to be >99 %

Single crystal X-RAY crystallography data

Crystal Data for **4i**: monoclinic, space group Cc (no. 9), a = 10.6558(6) Å, b = 10.9159(6) Å, c = 7.9047(4) Å, β = 106.711(2)°, V = 880.62(8) Å 3 , Z = 4, T = 100(2) K, μ(MoKα) = 0.114 mm -1 , Dcalc = 1.540 g/cm 3 , 40962 reflections measured (5.464° ≤ 2Θ ≤ 61.016°), 2662 unique (Rint = 0.0415, Rsigma = 0.0187) which were used in all calculations. The final R1 was 0.0538 (I > 2σ(I)) and wR2 was 0.1199 (all data).

Table S3. Crystallographic and refinement data for **4i**.

Bond precision:	C-C = 0.0044 A	Wavelength=0.71073	
Cell:	a=10.6558(6)	b=10.9159(6)	c=7.9047(4)
	alpha=90	beta=106.711(2)	gamma=90
Temperature:	100 K		

	Calculated	Reported
Volume	880.63(8)	880.62(8)
Space group	C c	C 1 c 1
Hall group	C -2yc	C -2yc
Moiety formula	C9 H8 N4 O2	0.25(C9 H8 N4 O2)
Sum formula	C9 H8 N4 O2	C2.25 H2 N O0.50
Mr	204.19	51.05
Dx,g cm-3	1.540	1.540
Z	4	16
Mu (mm-1)	0.114	0.114
F000	424.0	424.0
F000’	424.18	
h,k,lmax	15,15,11	15,15,11
Nref	2683[1345]	2662
Tmin,Tmax	0.956,0.973	0.716,0.740
Tmin’	0.934	
Correction method= # Reported T Limits: Tmin=0.716 Tmax=0.740<br>AbsCorr = MULTI-SCAN		
Data completeness= 1.98/0.99	Theta(max)= 30.508	
R(reflections)= 0.0538(2649)	wR2(reflections)= 0.1199(2662)	
S = 1.226	Npar= 141	

Crystal Data for **3a**: monoclinic, space group Cc (no. 9), a = 10.6558(6) Å, b = 10.9159(6) Å, c = 7.9047(4) Å, β = 106.711(2)°, V = 880.62(8) Å 3 , Z = 4, T = 100(2) K, μ(MoKα) = 0.114 mm -1 , Dcalc = 1.540 g/cm 3 , 40962 reflections measured (5.464° ≤ 2Θ ≤ 61.016°), 2662 unique (Rint = 0.0415, Rsigma = 0.0187) which were used in all calculations. The final R1 was 0.0538 (I > 2σ(I)) and wR2 was 0.1199 (all data).

Table S4. Crystallographic and refinement data for **3a**.

Bond precision:	C-C = 0.0043 A	Wavelength=0.71073	
Cell:	a=7.8283(5)	b=7.9681(5)	c=16.7138(11)
	alpha=78.839(2)	beta=84.972(2)	gamma=62.072(2)
Temperature:	100 K		

	Calculated	Reported
Volume	903.71(10)	903.71(10)
Space group	P -1	P -1
Hall group	-P 1	-P 1
Moiety formula	C8 H3 F6 O3 S, C6 H5 F3 N5	0.25(C8 H3 F6 O3 S) F3 N5)
Sum formula	C14 H8 F9 N5 O3 S	C3.50 H2 F2.25 N1.2 S0.25
Mr	497.31	124.33
Dx,g cm-3	1.828	1.828
Z	2	8
Mu (mm-1)	0.300	0.300
F000	496.0	496.0
F000'	496.68	
h,k,lmax	10,11,23	10,11,23
Nref	5190	5113
Tmin,Tmax	0.942,0.979	0.668,0.746
Tmin'	0.917	
Correction method= # Reported T Limits: Tmin=0.668 Tmax=0.746 AbsCorr = MULTI-SCAN		
Data completeness= 0.985	Theta(max)= 29.84	
R(reflections)= 0.0697(3892)	wR2(reflections)= 0.1398(5113)	
S = 1.105	Npar= 297	

Computational Methods

Ground and transition state geometries were optimized with frequency calculations using density functional theory (DFT) in Gaussian16 (Revision A.03)(*59*) at the M06-2X/6-31g(d) level of theory. A polarizable continuum solvation model (PCM)(*60*) in acetonitrile (MeCN) was used to approximate the solvent shell. Single point energy calculations for all structures were conducted using PCM(MeCN) at the M06-2x/6-311+g(d,p) level, with a frequency calculation at the same level of theory. All the optimized geometries were verified by frequency calculations as ground states (zero imaginary frequencies) or transition states (one imaginary frequency). Transition states were verified by intrinsic reaction coordinate (IRC) calculations. Quasi-harmonic corrected free energies (Gtz) for each compound were computed using Grimme entropic corrections(*61*) and Head-Gordon enthalpic corrections(*62*) as implemented in Paton's GoodVibes python package.(*63*) Neutral intermediate species were placed on the energy diagram by considering the thermochemical reaction **III** + **acid** → **III$^+$** + **anion**.

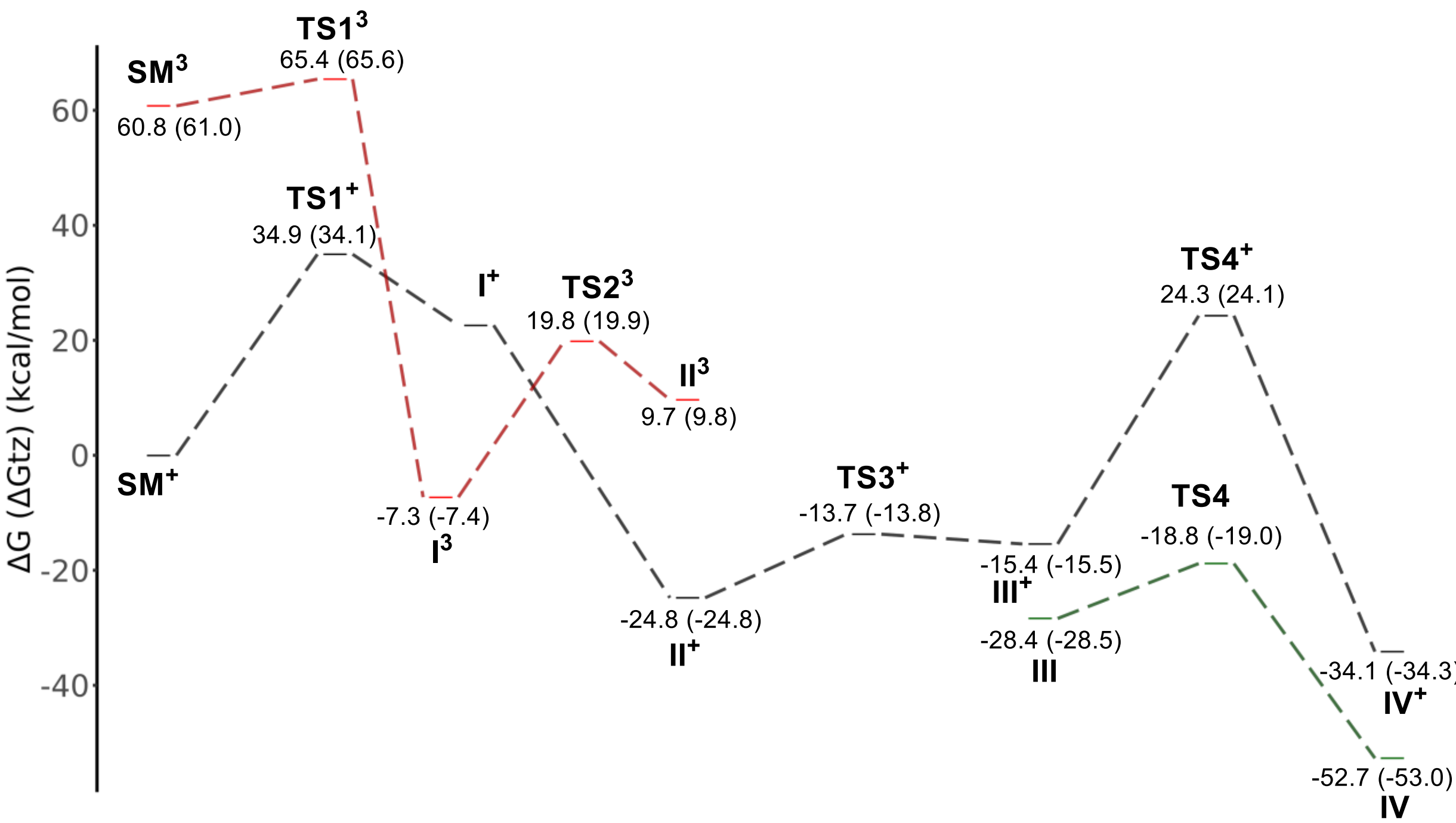

Figure S39. Reproduction of the energy diagram from the main manuscript Fig 3.

Coordinates for all optimized structures:

SM⁺

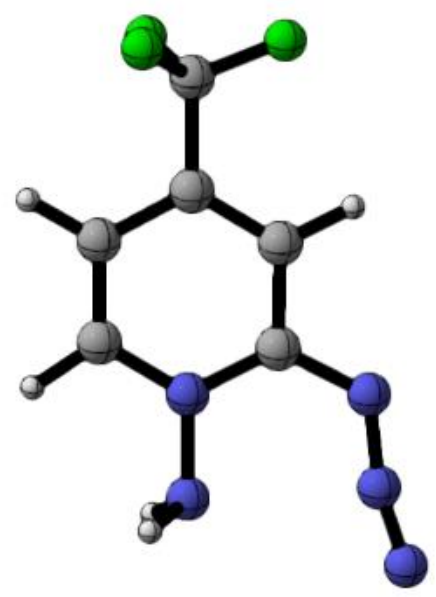

N	-2.73963	1.41744	0.00001
N	-1.39959	0.99988	0.00003
C	-1.17383	-0.33737	0.00004
N	-2.12603	-1.30947	0.00005
N	-3.35935	-1.06422	-0.00003
N	-4.47487	-1.09289	-0.00008
C	0.16136	-0.78711	0.00006
C	1.18278	0.12530	0.00001
C	2.62379	-0.33193	-0.00001
F	2.72404	-1.66043	-0.00018
F	3.25730	0.13733	-1.07807
F	3.25727	0.13705	1.07818
C	0.92081	1.50713	-0.00002
C	-0.38224	1.91059	-0.00000
H	-2.90195	1.98256	-0.83235
H	-2.90206	1.98228	0.83252
H	0.33531	-1.85536	0.00007
H	1.71554	2.24263	-0.00005
H	-0.69412	2.94745	-0.00002

TS1⁺

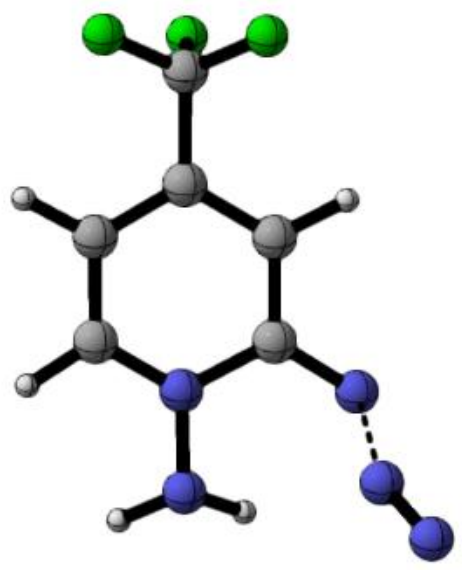

N	2.65664	1.58302	0.19621
N	1.36722	1.15427	0.15182
C	1.15842	-0.21264	0.42350
N	2.13919	-0.93818	0.96857
N	3.45419	-1.09542	-0.40672
N	4.38220	-1.54456	-0.78764
C	-0.14325	-0.70688	0.29504
C	-1.19535	0.14760	-0.01612
C	-2.61095	-0.37905	-0.05889
F	-2.63090	-1.65066	-0.46391
F	-3.16816	-0.32818	1.15342
F	-3.36559	0.34186	-0.88951
C	-0.92558	1.47075	-0.32235
C	0.38129	1.95115	-0.26056
H	3.14853	1.04880	0.91721
H	2.70563	2.58828	0.34471
H	-0.28762	-1.76754	0.46410
H	-1.70997	2.15321	-0.62725
H	0.64170	2.97051	-0.51815

I⁺

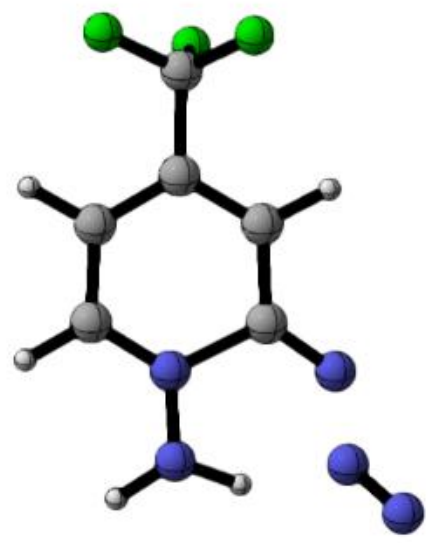

N	2.58784	1.58349	0.25289
N	1.35273	1.21183	0.16047
C	1.12534	-0.25096	0.57294
N	2.02085	-0.83310	1.22454
N	3.65038	-1.04045	-0.65188
N	4.58699	-1.60274	-0.76069
C	-0.19765	-0.71416	0.32133
C	-1.21984	0.12968	-0.03996
C	-2.63184	-0.39690	-0.08754
F	-2.63708	-1.67375	-0.47775
F	-3.19713	-0.34064	1.12131
F	-3.38371	0.31179	-0.92959
C	-0.92718	1.45724	-0.35460
C	0.36815	1.98078	-0.29252
H	3.14373	0.92148	0.81062
H	2.80827	2.57460	0.21157
H	-0.36257	-1.76785	0.51941
H	-1.71526	2.12604	-0.68559
H	0.59380	3.00172	-0.57716

II⁺

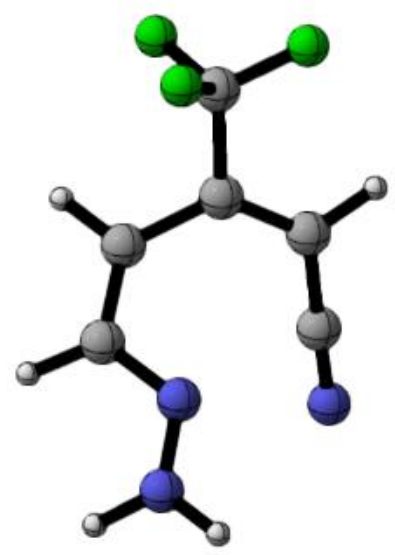

N	-3.31116	-1.06241	-0.62370
N	-2.12357	-0.74681	-0.45609
C	-1.28803	1.85080	0.16124
N	-2.34865	2.24112	0.40855
C	0.03512	1.40331	-0.15640
C	0.51135	0.18667	0.15082
C	1.97161	-0.11661	-0.11599
F	2.59925	0.89658	-0.70732
F	2.59821	-0.38616	1.03507
F	2.08484	-1.19462	-0.89693
C	-0.19424	-0.88594	0.84842
C	-1.46006	-1.29992	0.61685
H	-3.79573	-0.67287	-1.43426
H	-3.82976	-1.69027	0.00449
H	0.68469	2.13711	-0.62317
H	0.36696	-1.40105	1.62529
H	-1.93779	-2.06835	1.21924

TS3^{+}

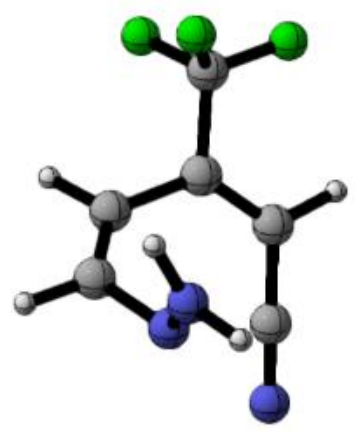

N	-2.36398	-1.69003	-0.98022
N	-2.33968	-0.82650	-0.11558
C	-1.37481	1.95285	-0.04811
N	-2.43059	2.36667	0.18169
C	-0.06555	1.45188	-0.34078
C	0.41297	0.32690	0.20367
C	1.83542	-0.08753	-0.09811
F	2.45255	0.76413	-0.91575
F	2.54256	-0.16279	1.03661
F	1.85775	-1.29873	-0.66421
C	-0.28075	-0.56147	1.15116
C	-1.49441	-1.08757	1.00783
H	-2.98471	-1.54571	-1.78617
H	-1.79504	-2.55208	-0.94224
H	0.54094	2.04488	-1.01682
H	0.27491	-0.86070	2.03545
H	-1.96936	-1.73118	1.74472

III$^+$

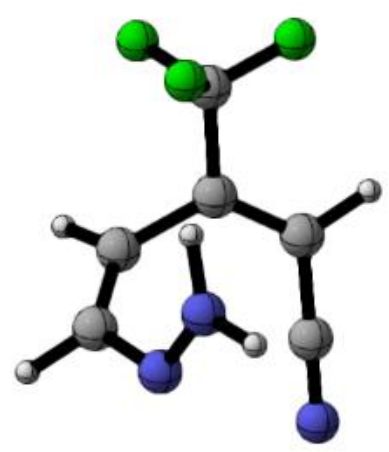

N	-1.34121	-1.58834	-1.20246
N	-2.05209	-1.42231	-0.21147
C	-1.71058	1.75153	-0.08084
N	-2.83729	1.95785	0.07963
C	-0.32434	1.46044	-0.30418
C	0.27429	0.42197	0.28789
C	1.71564	0.10280	-0.02691
F	2.28962	1.02058	-0.79349
F	2.43118	-0.03844	1.08727
F	1.75793	-1.07617	-0.68481
C	-0.36074	-0.49083	1.26261
C	-1.40816	-1.29478	1.03106
H	-1.80080	-1.64758	-2.11733
H	-0.31231	-1.67798	-1.15779
H	0.21570	2.10461	-0.99023
H	0.01738	-0.45431	2.28188
H	-1.93111	-1.81560	1.82517

TS4$^+$

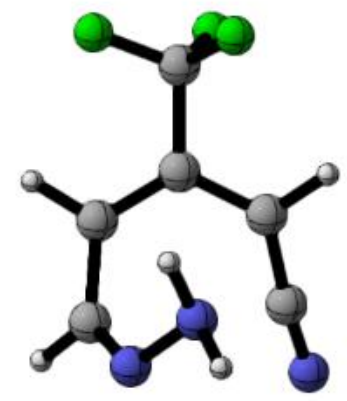

N	1.93970	-0.79460	1.33049
N	2.44281	-1.22550	0.10924
C	1.58407	1.57654	-0.13778
N	2.53338	2.10112	-0.54416
C	0.33559	1.08590	0.32894
C	-0.38707	0.03934	-0.24858
C	-1.88543	0.02113	0.02377
F	-2.48945	1.00324	-0.64082
F	-2.42136	-1.13569	-0.34821
F	-2.10965	0.20631	1.32521
C	0.22646	-1.02263	-0.87552
C	1.68280	-1.24883	-0.92071
H	2.69580	-0.86229	2.00662
H	1.16118	-1.36590	1.66725
H	-0.13264	1.69127	1.10741
H	-0.40748	-1.83368	-1.23391
H	2.11757	-1.56996	-1.86258

IV⁺

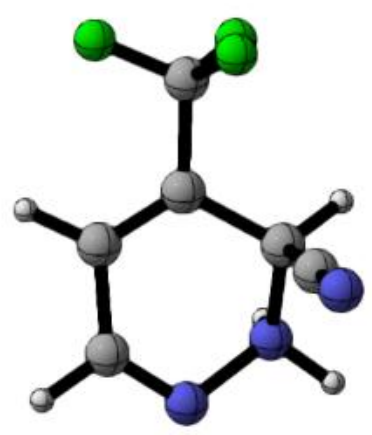

N	-1.31532	2.55673	-0.96131
C	-1.04905	1.69711	-0.24026
C	-0.68415	0.59573	0.66644
C	0.23299	-0.39877	-0.01114
C	1.70426	-0.09475	0.04543
F	2.41262	-0.97620	-0.64899
F	1.92647	1.12836	-0.45061
F	2.13207	-0.09971	1.31079
C	-0.29286	-1.43971	-0.65016
C	-1.75700	-1.54727	-0.71102
N	-2.58318	-0.94189	0.05361
N	-1.91686	-0.14785	1.08611
H	-0.23729	1.00581	1.57794
H	0.31413	-2.17458	-1.16494
H	-2.21922	-2.17000	-1.47136
H	-1.68441	-0.77758	1.87287
H	-2.63132	0.50145	1.44021

SM[3]

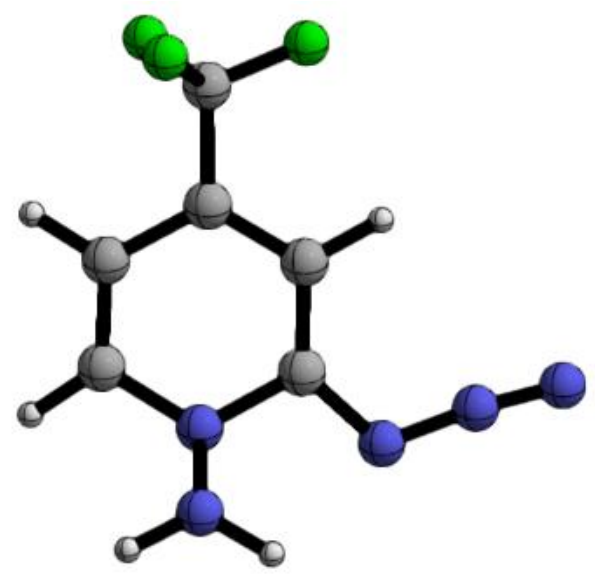

N	2.24534	2.08484	0.73238
N	1.42274	1.52843	-0.14602
C	1.39875	0.07973	-0.12042
N	2.67315	-0.40996	-0.23112
N	2.81656	-1.64766	-0.11372
N	3.06636	-2.73756	-0.03162
C	0.18655	-0.56018	0.00208
C	-0.99015	0.17742	-0.06692
C	-2.32465	-0.47719	0.08638
F	-2.95545	-0.05449	1.19348
F	-3.12855	-0.18230	-0.94732
F	-2.22274	-1.80799	0.16208
C	-0.94846	1.58542	-0.38302
C	0.21763	2.23963	-0.46462
H	2.22063	3.09794	0.83378
H	3.08424	1.55894	0.97858
H	0.16056	-1.64198	0.08058
H	-1.86666	2.11901	-0.60422
H	0.35483	3.27352	-0.75272

TS1[3]

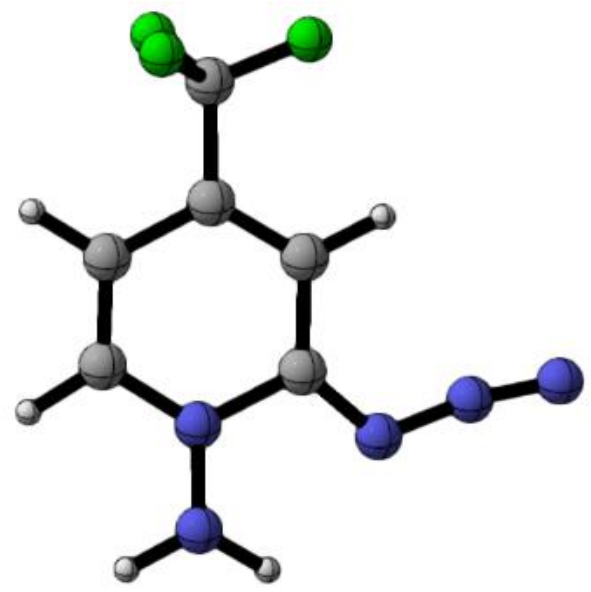

N	2.56788	2.08386	-0.38052
N	1.44351	1.50081	0.00160
C	1.40330	0.04265	-0.03843
N	2.59412	-0.50189	0.09466
N	2.71239	-1.76293	0.24738
N	2.95524	-2.85013	-0.01609
C	0.11676	-0.57165	-0.11094
C	-0.99515	0.18073	0.02277
C	-2.36530	-0.42223	-0.04418
F	-3.04837	-0.17037	1.08107
F	-3.06980	0.10715	-1.05384
F	-2.32347	-1.74454	-0.21766
C	-0.90082	1.61943	0.26297
C	0.29217	2.23925	0.24425
H	2.63978	3.08668	-0.22813
H	3.40914	1.51257	-0.31967
H	0.06751	-1.64771	-0.23823
H	-1.79441	2.19882	0.46029
H	0.43509	3.30227	0.39169

I[3]

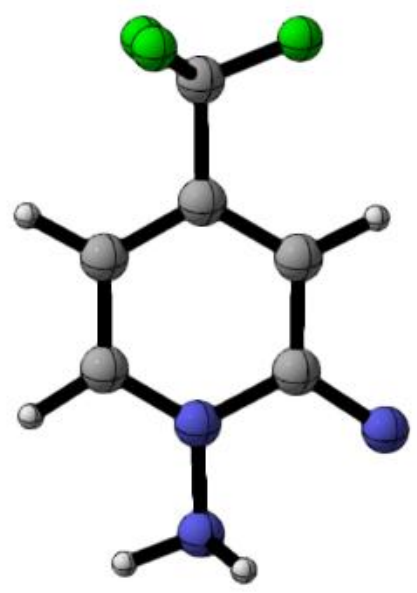

N	3.53639	-0.47698	-0.05956
N	2.15689	-0.39031	0.01247
C	1.62496	0.89010	-0.00651
N	2.45956	1.92173	-0.05285
C	0.21983	1.04885	0.01084
C	-0.58227	-0.07071	-0.00682
C	-2.08948	0.05058	0.00402
F	-2.58924	-0.48995	1.11742
F	-2.47647	1.32364	-0.05615
F	-2.61461	-0.60198	-1.03487
C	-0.00930	-1.34386	-0.02905
C	1.37256	-1.47380	-0.00615
H	3.81557	-1.45509	-0.05266
H	3.94824	0.00429	0.74059
H	-0.18097	2.05545	0.01799
H	-0.61624	-2.24110	-0.04630
H	1.86862	-2.43682	0.00451

TS2[3]

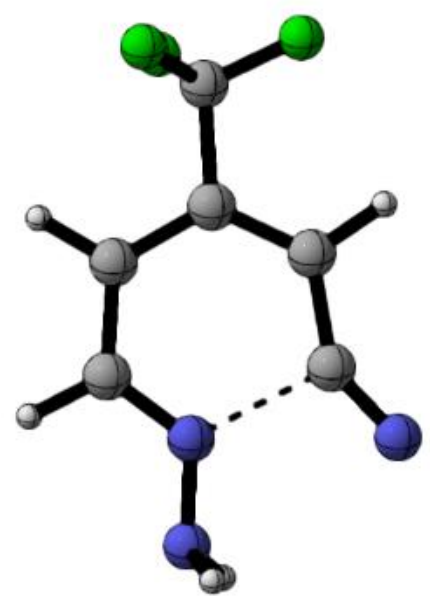

N	3.58149	-0.71485	-0.00005
N	2.26273	-0.54421	-0.00002
C	1.54213	1.26743	-0.00015
N	2.47897	2.01012	-0.00025
C	0.11617	1.15380	-0.00009
C	-0.57923	-0.02619	0.00002
C	-2.09537	0.01877	0.00006
F	-2.57476	-0.60699	1.07754
F	-2.55671	1.26806	-0.00004
F	-2.57484	-0.60721	-1.07727
C	-0.00077	-1.30139	0.00010
C	1.39624	-1.51264	0.00009
H	4.02610	-0.37071	0.85117
H	4.02604	-0.37095	-0.85140
H	-0.40804	2.10321	-0.00014
H	-0.63068	-2.18257	0.00018
H	1.80600	-2.51989	0.00017

II[3]

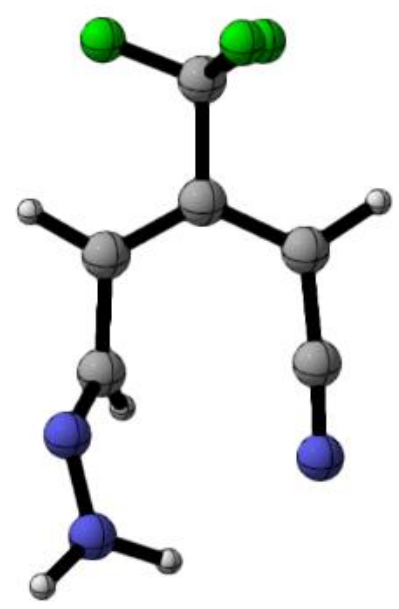

N	3.71166	-0.72704	0.30857
N	2.52105	-1.20599	0.27723
C	1.17203	1.74755	-0.03796
N	2.31799	1.95141	-0.05088
C	-0.19910	1.41065	-0.01459
C	-0.61604	0.06668	-0.11339
C	-2.10353	-0.16426	0.06332
F	-2.79544	0.58793	-0.79798
F	-2.43519	-1.43966	-0.13471
F	-2.48949	0.17986	1.29516
C	0.19718	-1.01242	-0.32120
C	1.63131	-0.84735	-0.58945
H	4.43092	-1.27520	0.77723
H	3.87002	0.26375	0.08453
H	-0.91843	2.21220	0.10644
H	-0.20350	-2.02158	-0.33282
H	1.95609	-0.46596	-1.56234

III

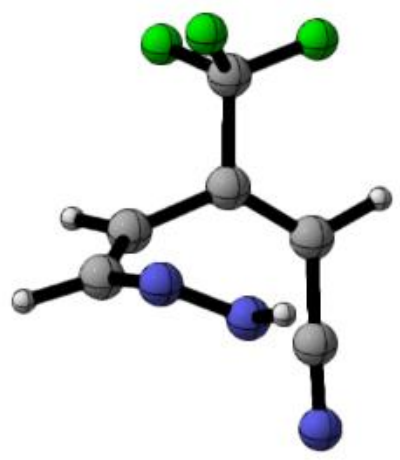

N	-2.14418	-0.93472	-1.08877
N	-1.70554	-1.89872	-0.44323
C	-1.22377	-1.60435	0.85695
C	-0.31350	-0.67414	1.16830
C	0.27043	0.31846	0.24551
C	1.72260	0.07974	-0.09041
F	2.45404	-0.00022	1.03020
F	1.86828	-1.08060	-0.74318
F	2.24646	1.04327	-0.85244
C	-0.33563	1.40212	-0.24898
C	-1.68040	1.75617	0.10244
N	-2.74281	2.10783	0.39571
H	-2.39491	-1.29968	-2.02211
H	-1.52260	-2.34239	1.59451
H	0.11771	-0.70259	2.16645
H	0.19005	2.09380	-0.89893

TS4

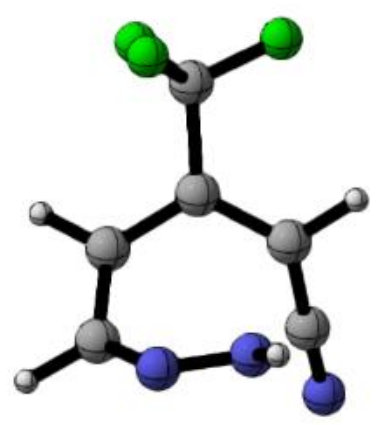

N	1.95806	-0.54114	1.23367
N	1.92495	-1.54794	0.49466
C	1.42242	-1.54528	-0.77054
C	0.22982	-0.91199	-1.07431
C	-0.28226	0.09956	-0.23659
C	-1.75697	0.10435	0.04644
F	-2.45397	0.13900	-1.10037
F	-2.15190	-0.99929	0.70168
F	-2.13061	1.15613	0.78562
C	0.55601	0.95767	0.44637
C	1.72780	1.49134	-0.19813
N	2.64087	1.96685	-0.72705
H	2.02107	-0.84998	2.21563
H	1.78924	-2.35574	-1.38892
H	-0.39506	-1.35071	-1.84730
H	0.16492	1.57564	1.24988

IV

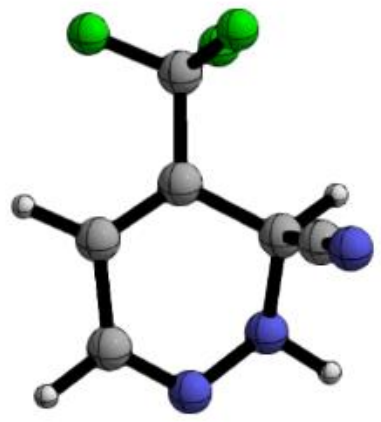

N	1.86681	-0.21086	1.12324
N	2.49810	-1.02597	0.23846
C	1.77201	-1.56704	-0.67941
C	0.32853	-1.37236	-0.74058
C	-0.20688	-0.37983	-0.02595
C	-1.66689	-0.08747	0.04897
F	-2.39167	-0.92051	-0.69950
F	-2.11716	-0.16818	1.31122
F	-1.91510	1.16799	-0.36579
C	0.73104	0.57347	0.68258
C	1.11838	1.69134	-0.23541
N	1.43224	2.54367	-0.94972
H	2.53326	0.28977	1.69878
H	2.26891	-2.25874	-1.34929
H	-0.28486	-2.06081	-1.31138
H	0.26074	1.01955	1.56351

acid

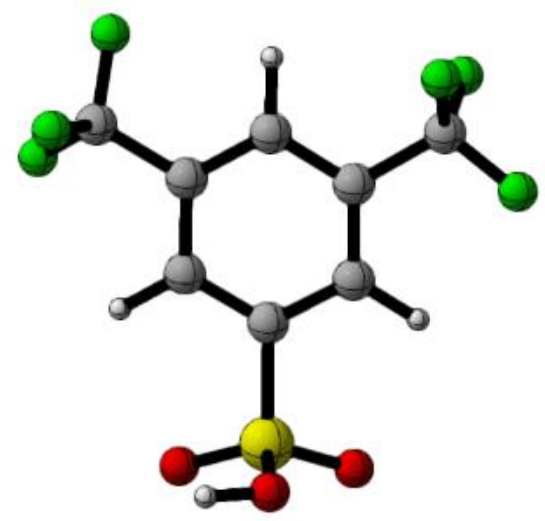

O	-3.36229	-1.26325	-0.78299
S	-2.93651	-0.05014	-0.12079
C	-1.16261	-0.02082	-0.08059
C	-0.50972	1.20299	-0.08769
C	0.88278	1.20860	-0.04199
C	1.60826	2.52671	0.00171
F	0.98425	3.45091	-0.73929
F	1.67566	3.00421	1.25241
F	2.86266	2.41473	-0.44959
C	1.59739	0.01921	0.00301
C	0.91161	-1.19470	-0.00009
C	1.71232	-2.46889	0.01121
F	2.61632	-2.46077	1.00021
F	0.93703	-3.54569	0.17291
F	2.39032	-2.62458	-1.13439
C	-0.47439	-1.23081	-0.04289
O	-3.40882	1.25235	-0.54759
H	-1.07393	2.12848	-0.13969
H	2.68239	0.03443	0.03261
H	-1.01028	-2.17382	-0.05659
O	-3.33460	-0.26254	1.42011
H	-3.35832	0.59755	1.88391

anion

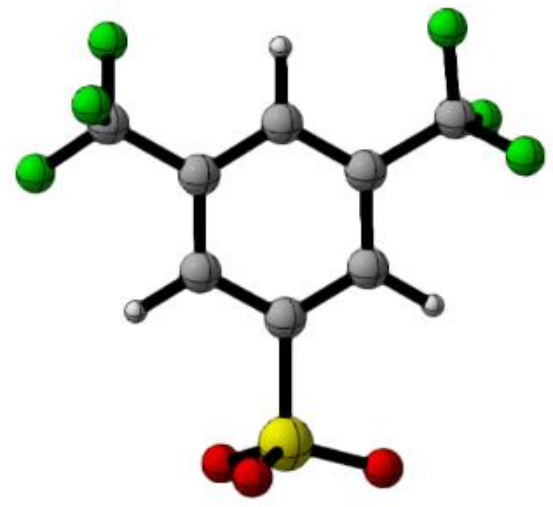

O	3.36328	-0.78899	-1.18449
S	2.98981	0.00692	0.00171
C	1.19210	0.01587	-0.01179
C	0.49364	1.21290	0.01761
C	-0.90186	1.19044	0.01961
C	-1.64162	2.49756	0.00251
F	-1.11989	3.36795	0.87981
F	-1.57820	3.08306	-1.20429
F	-2.93862	2.34860	0.30221
C	-1.59630	-0.01014	-0.00419
C	-0.87643	-1.20446	-0.03119
C	-1.62377	-2.50724	-0.00189
F	-2.77877	-2.43030	-0.67949
F	-0.90740	-3.50706	-0.53259
F	-1.93468	-2.87043	1.25321
C	0.51177	-1.20080	-0.03729
O	3.34719	-0.63919	1.28021
O	3.37825	1.42861	-0.08069
H	1.04137	2.14988	0.03691
H	-2.68080	-0.02091	-0.00509
H	1.06574	-2.13402	-0.06979

NMR Spectra:

¹H NMR (400 MHz, $CDCl_3$)

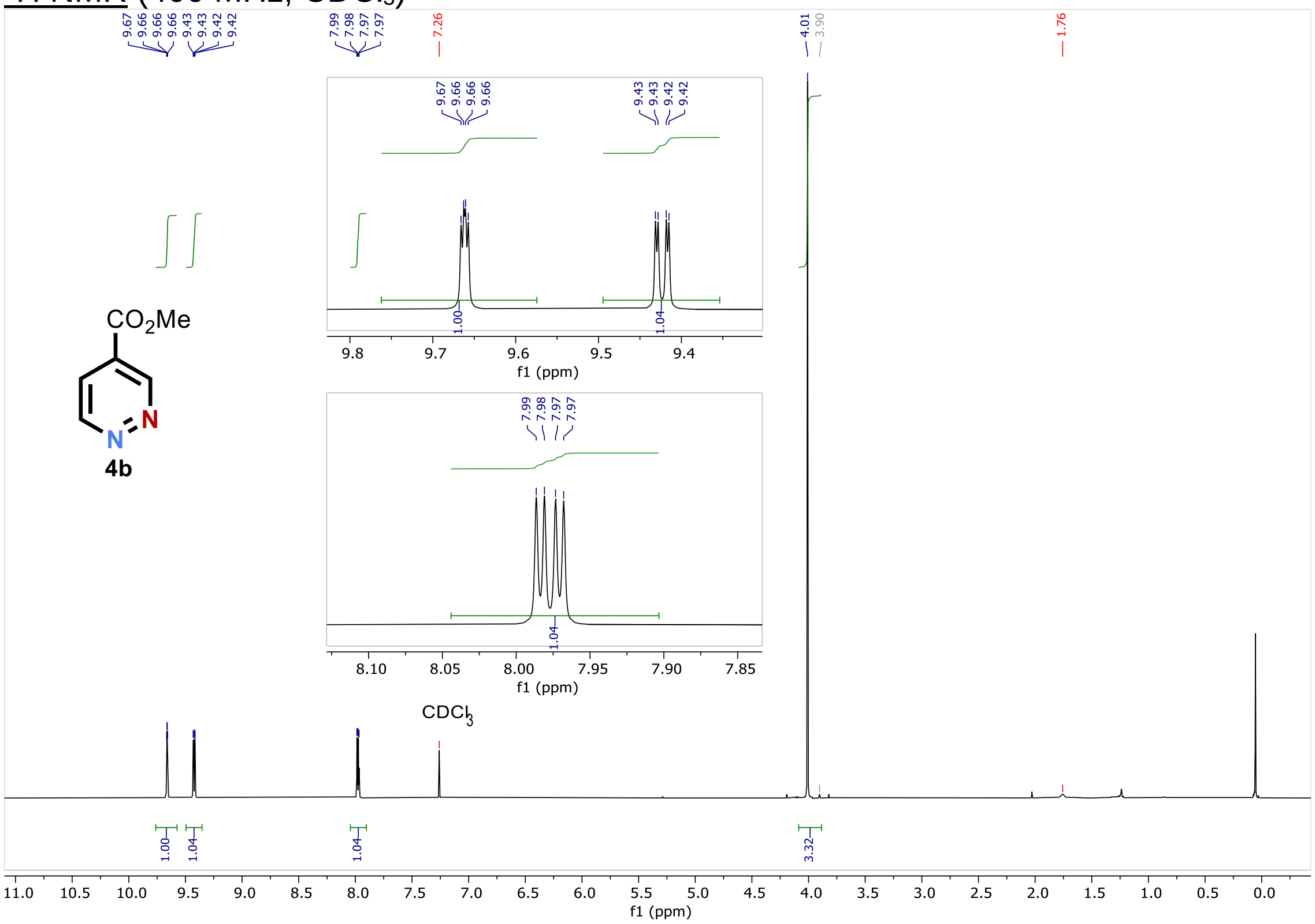

$^{13}C\{^1H\}$ NMR (101 MHz, $CDCl_3$)

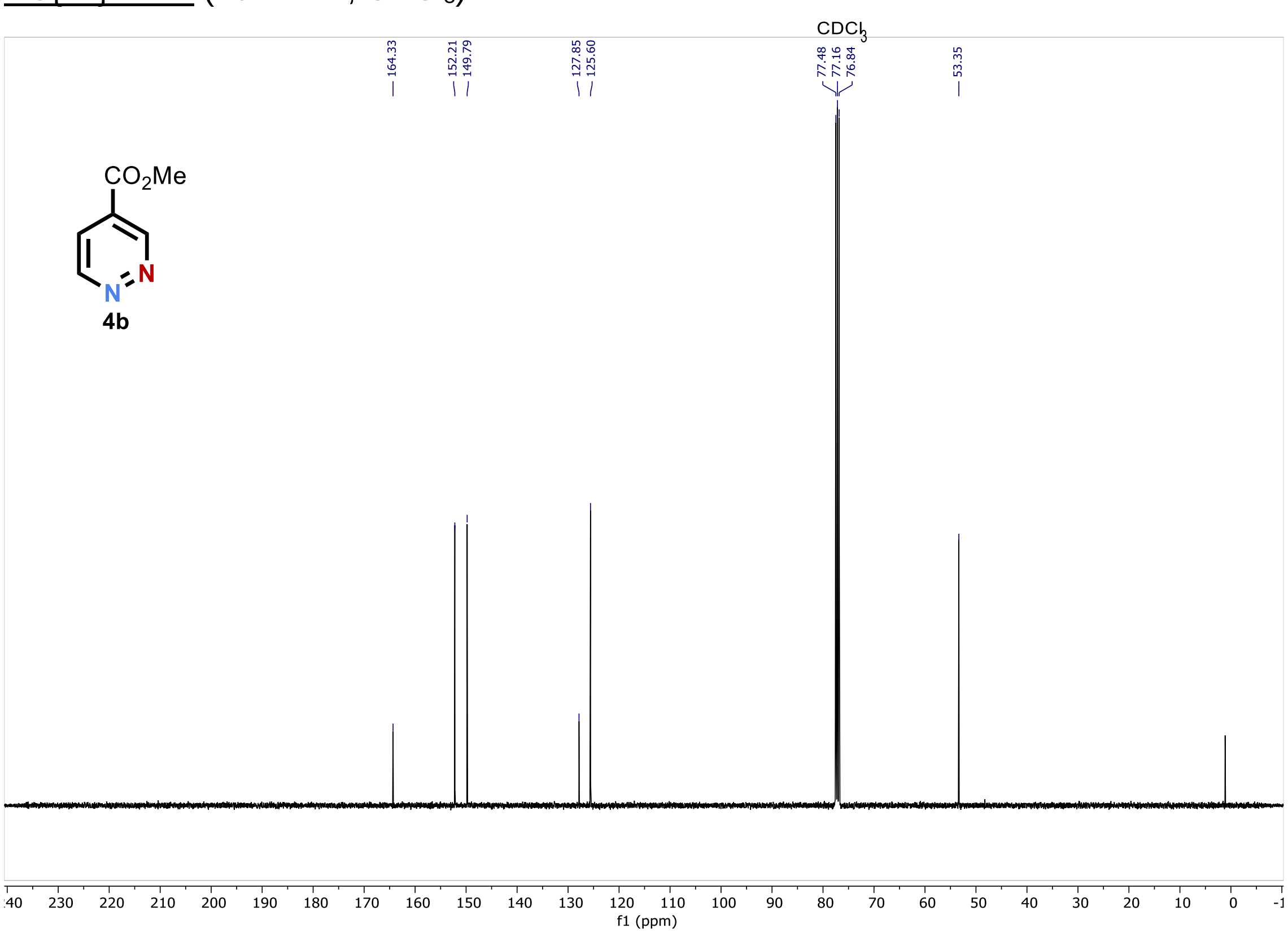

^{1}H NMR (400 MHz, $CDCl_3$)

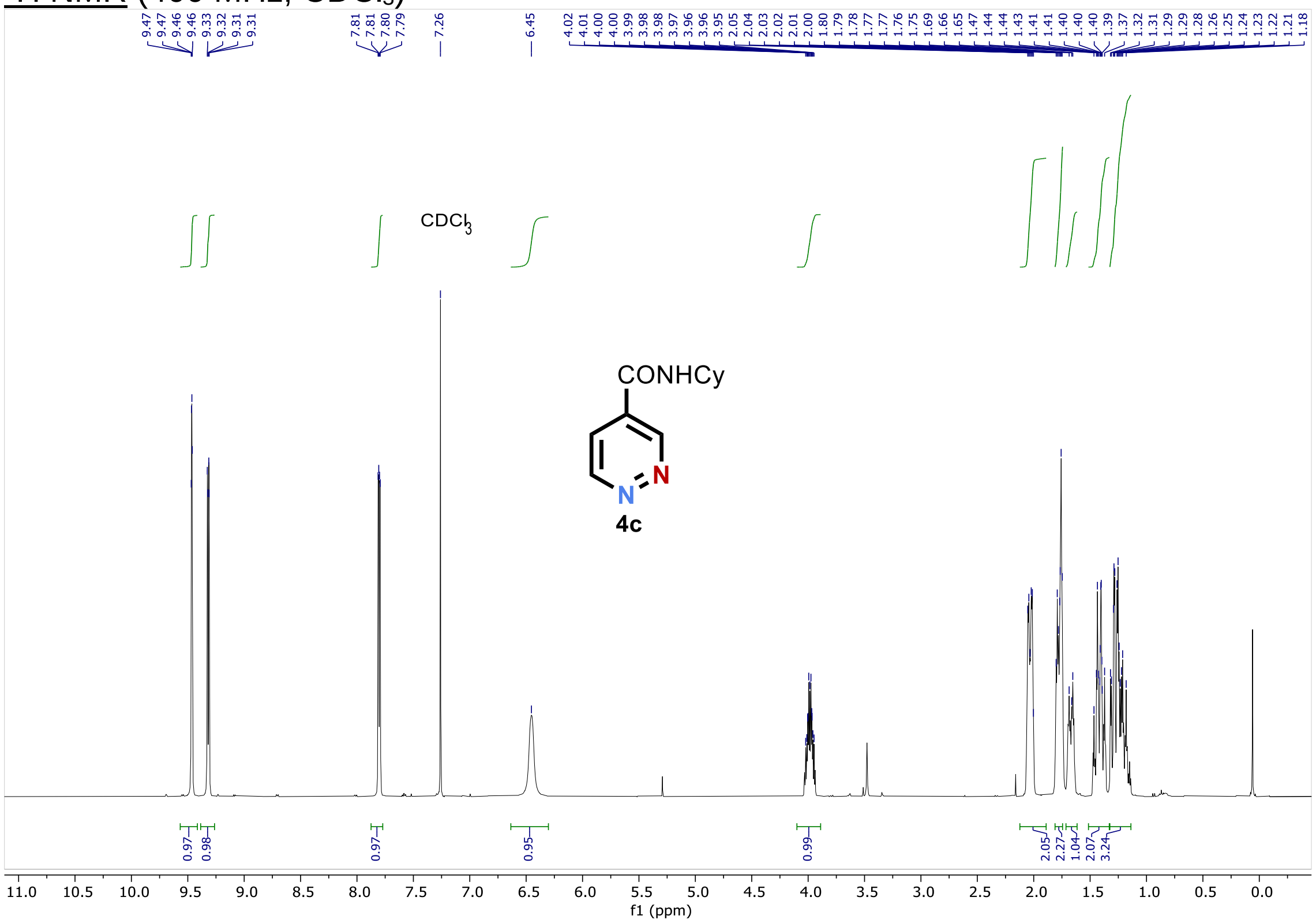

$^{13}C\{^{1}H\}$ NMR (101 MHz, $CDCl_3$)

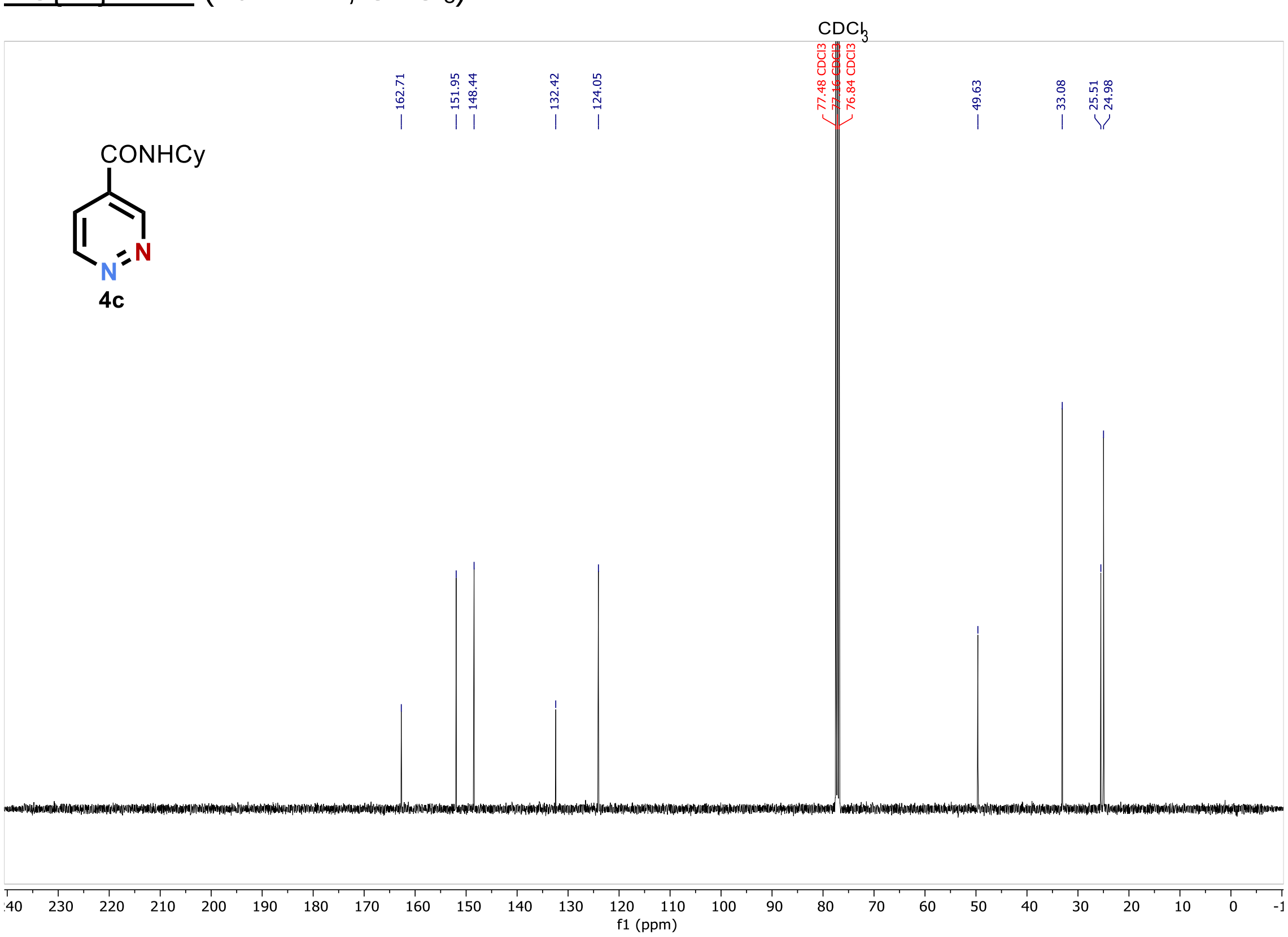

^{1}H NMR (400 MHz, $CDCl_3$)

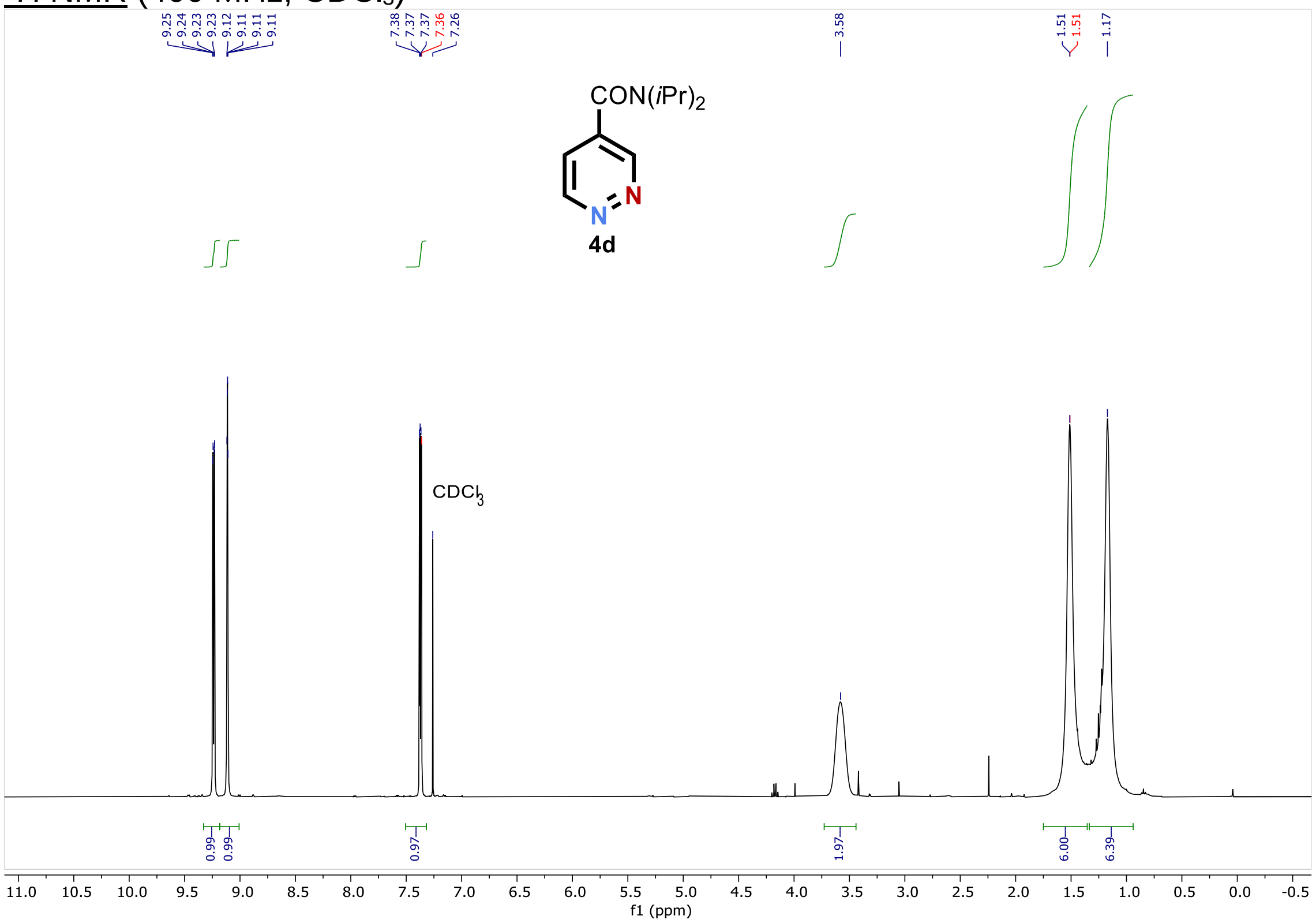

$^{13}C\{^{1}H\}$ NMR (101 MHz, $CDCl_3$)

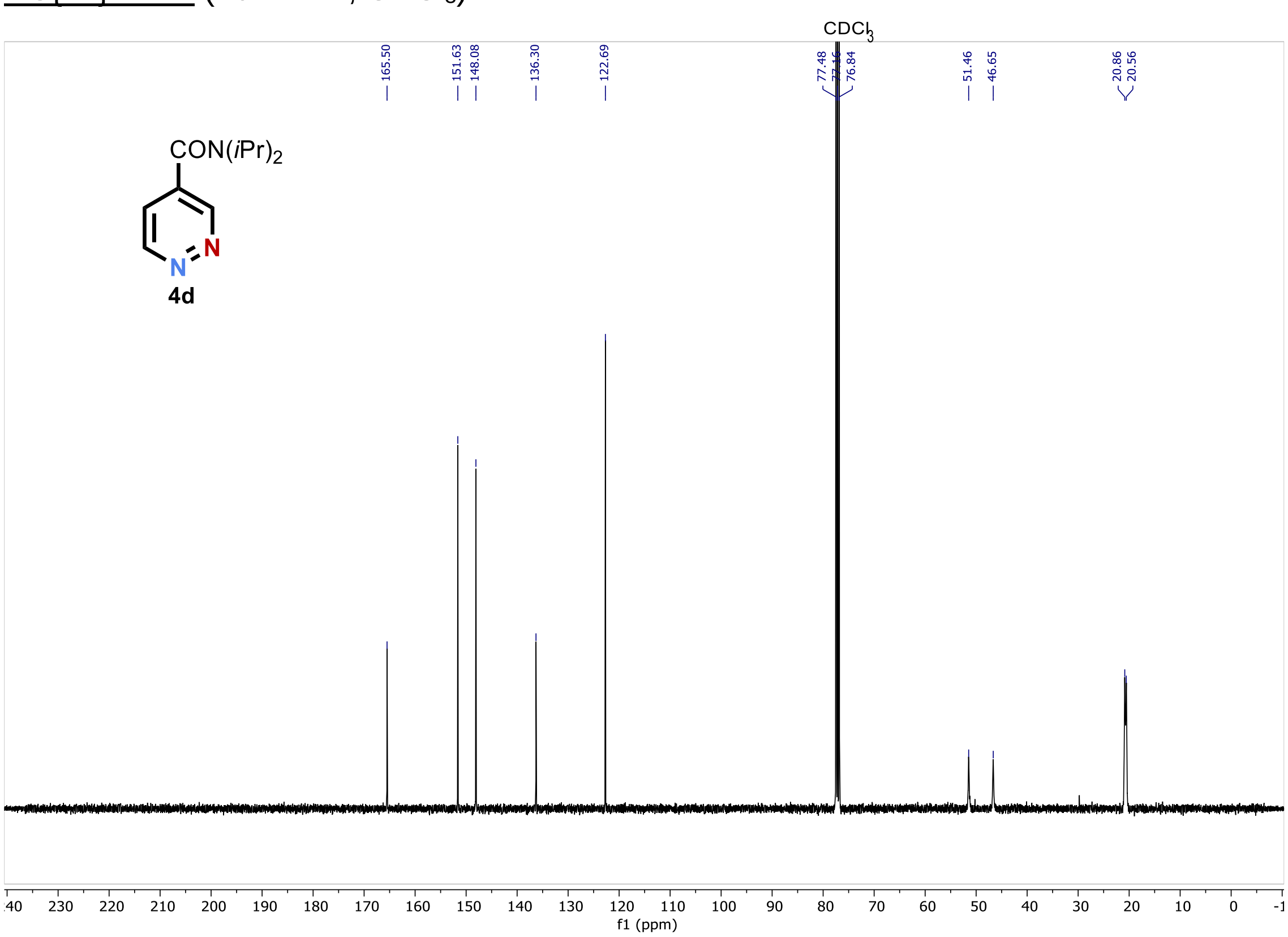

^{1}H NMR (400 MHz, $CDCl_3$)

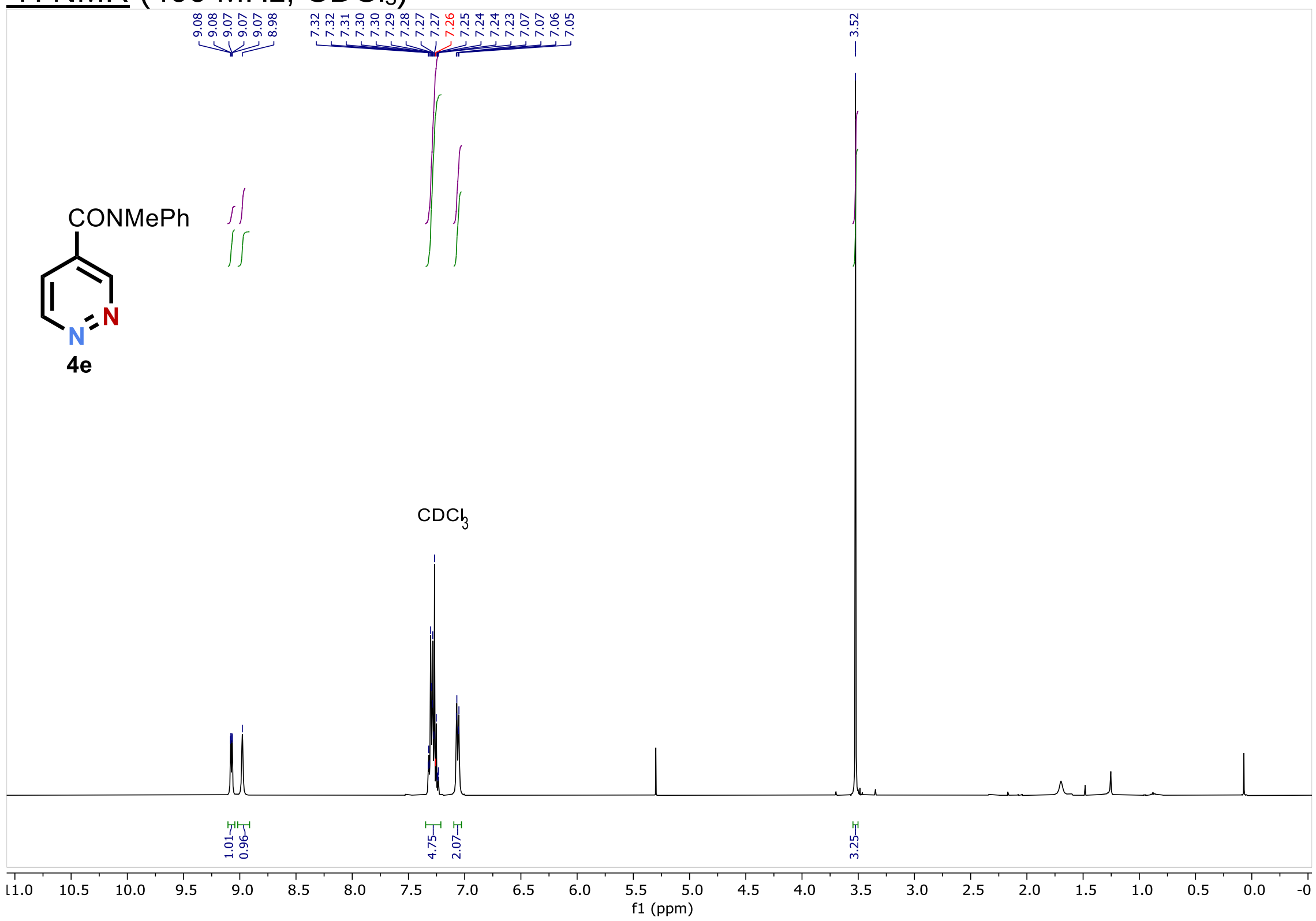

^{13}C{^{1}H} NMR (101 MHz, $CDCl_3$)

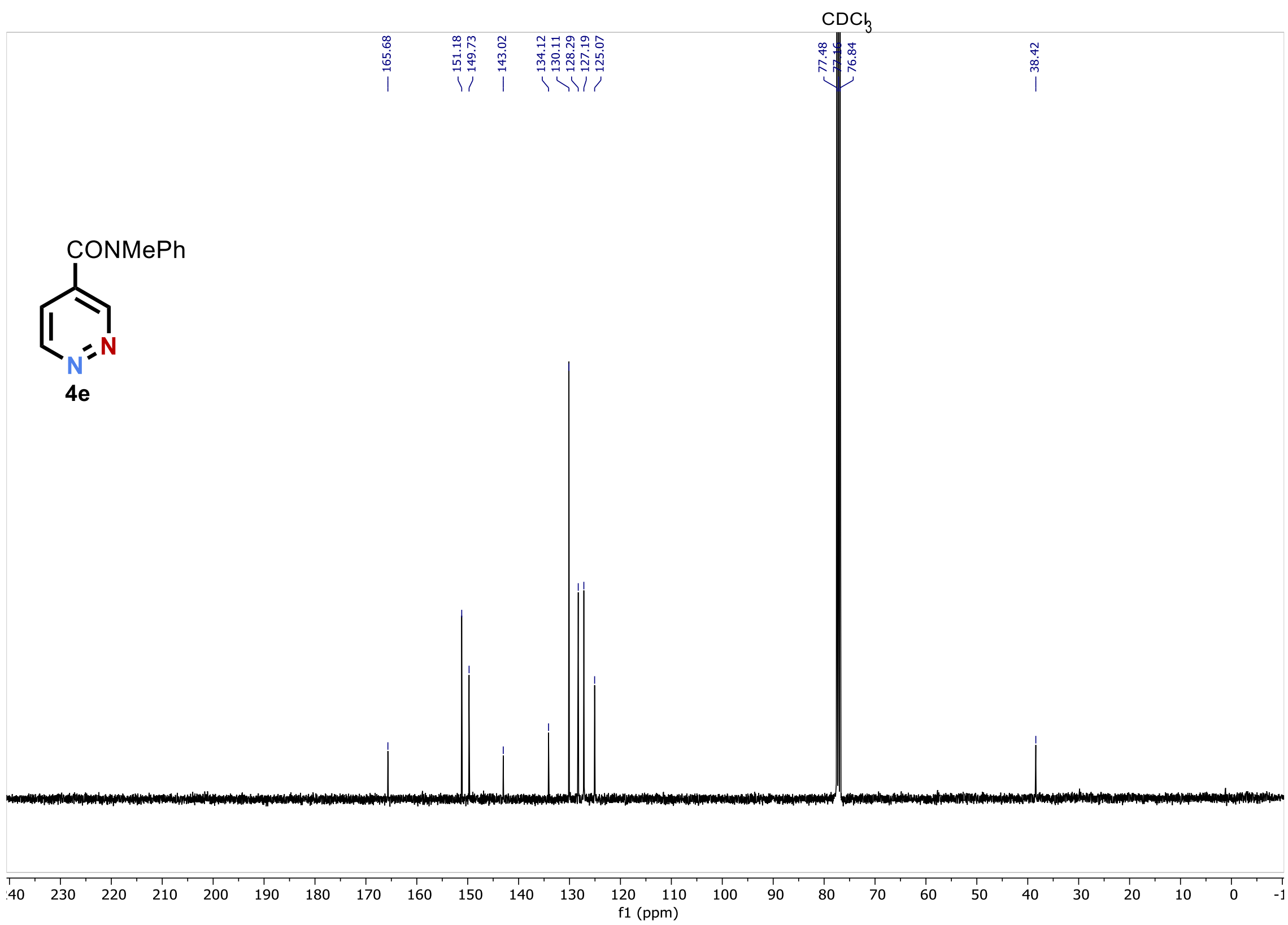

^{1}H NMR (400 MHz, $CDCl_3$)

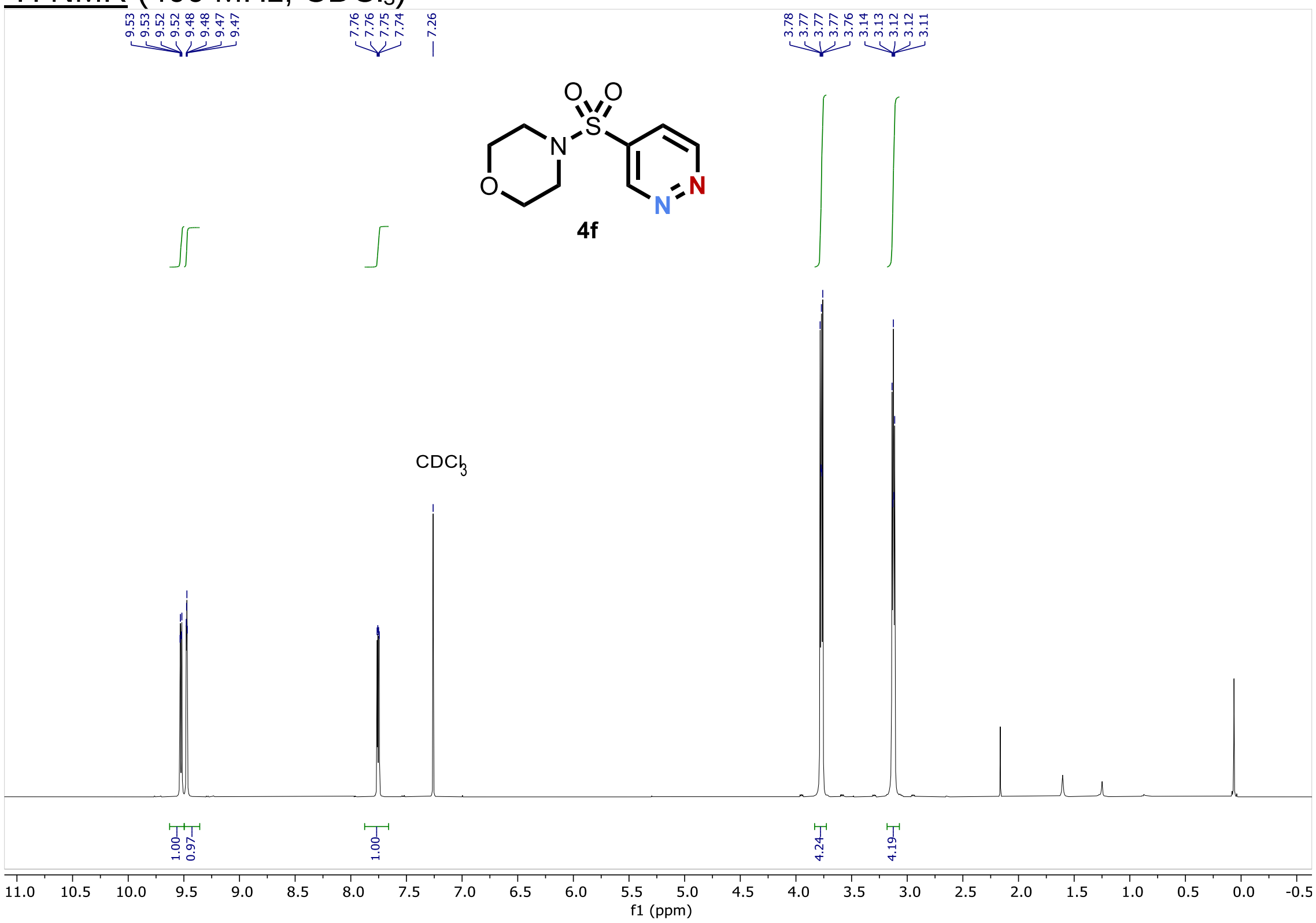

^{13}C{^{1}H} NMR (101 MHz, $CDCl_3$)

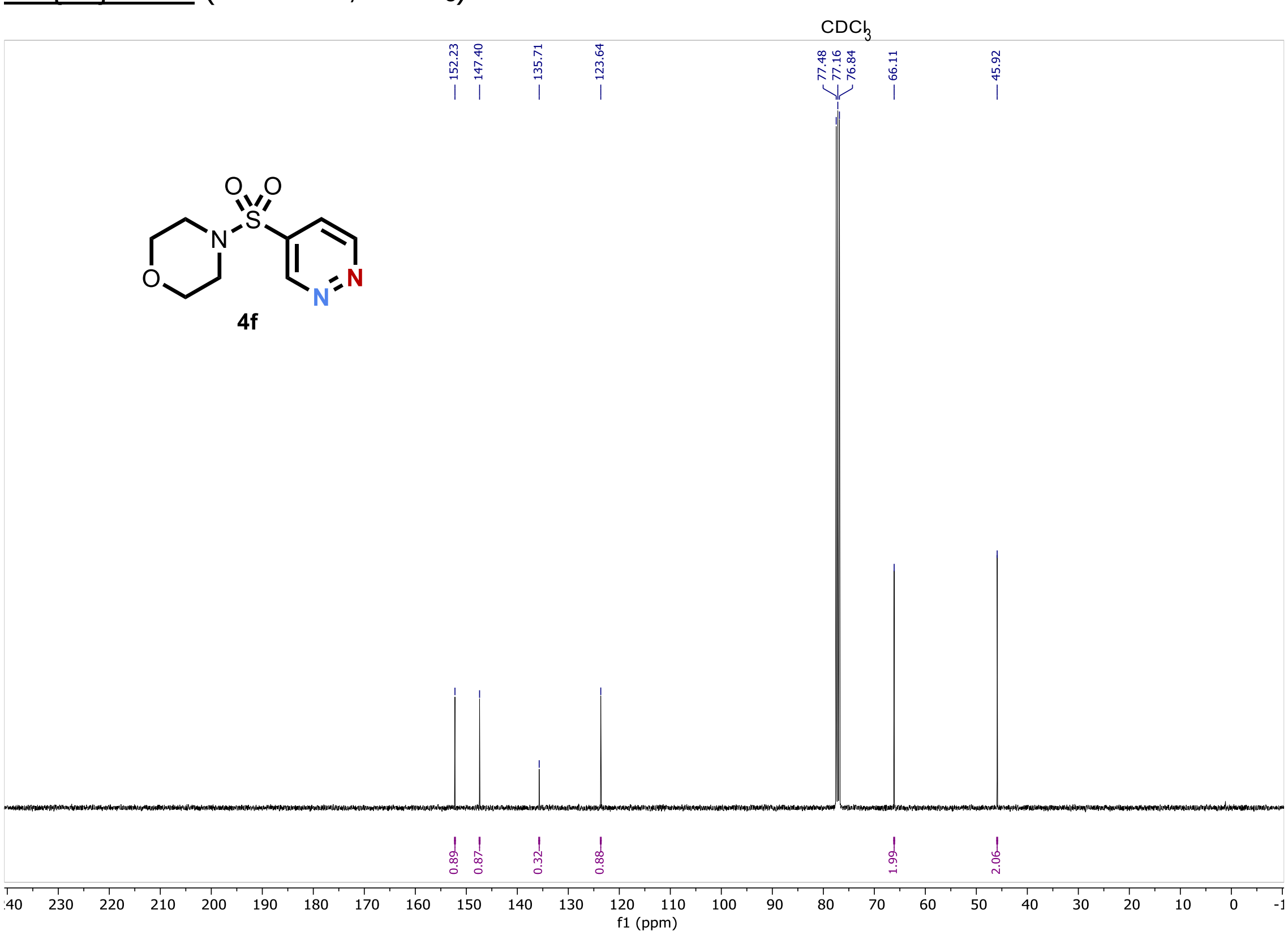

^{1}H NMR (400 MHz, $CDCl_3$)

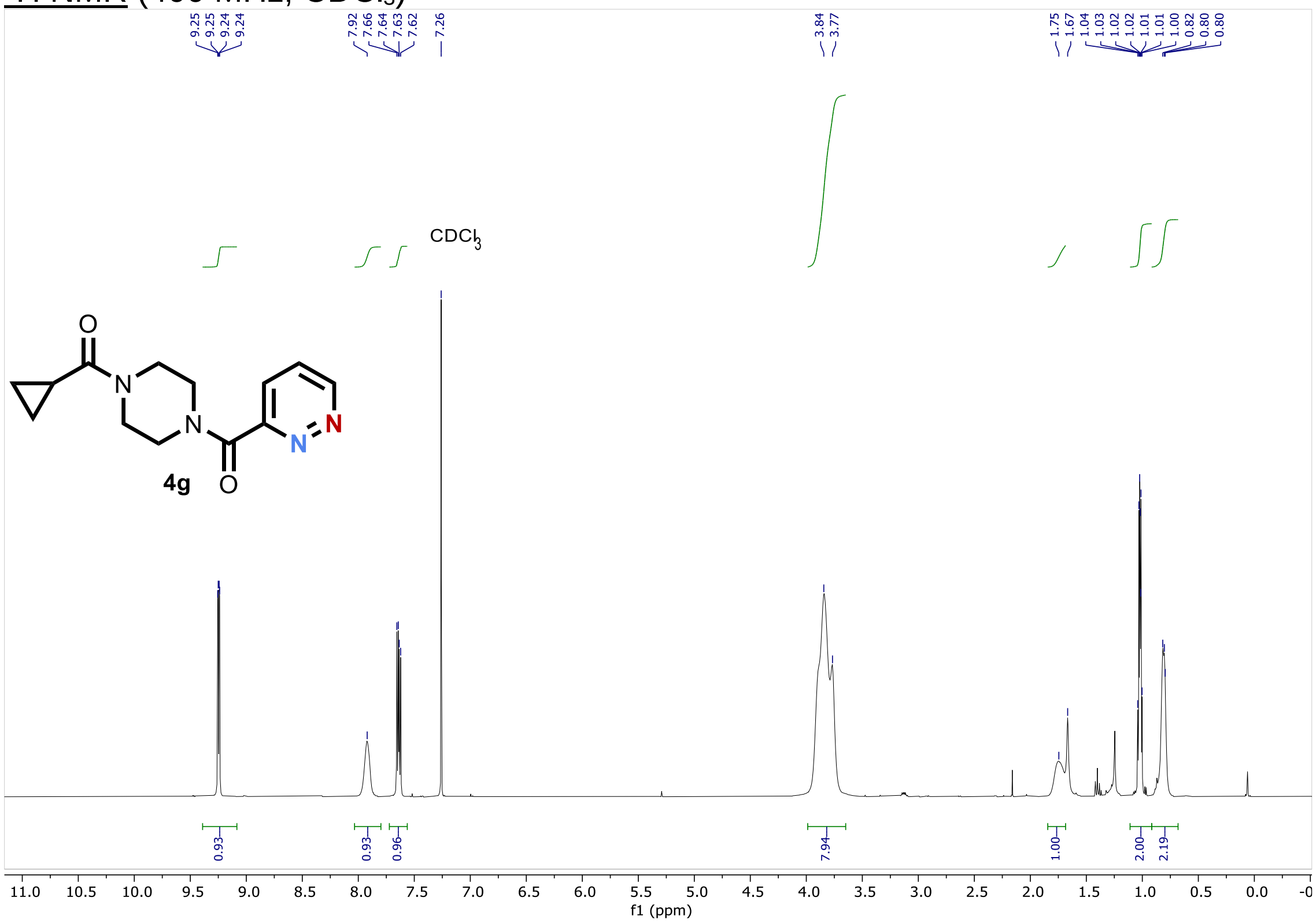

^{13}C{^{1}H} NMR (101 MHz, $CDCl_3$)

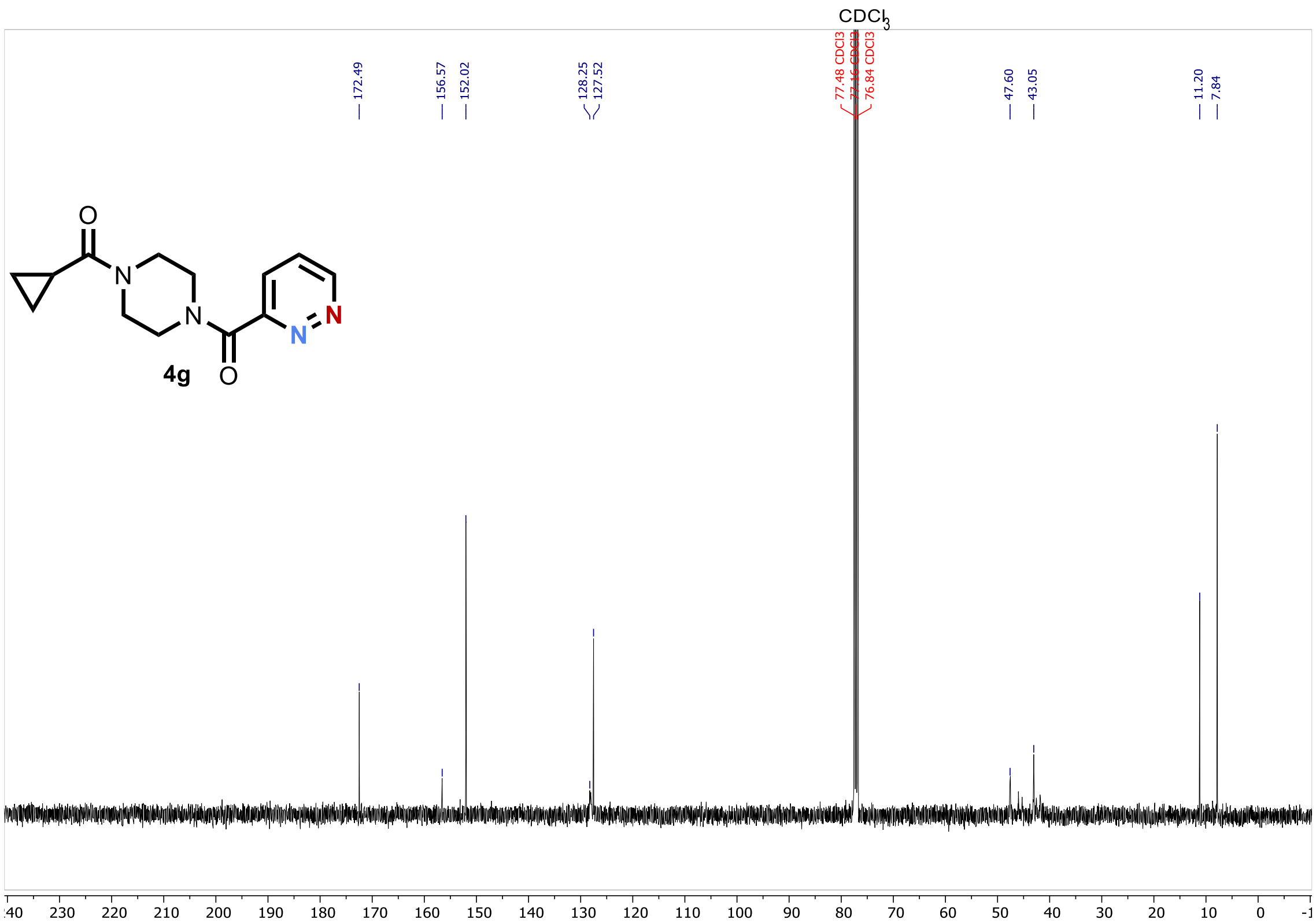

^{1}H NMR (400 MHz, $CDCl_3$)

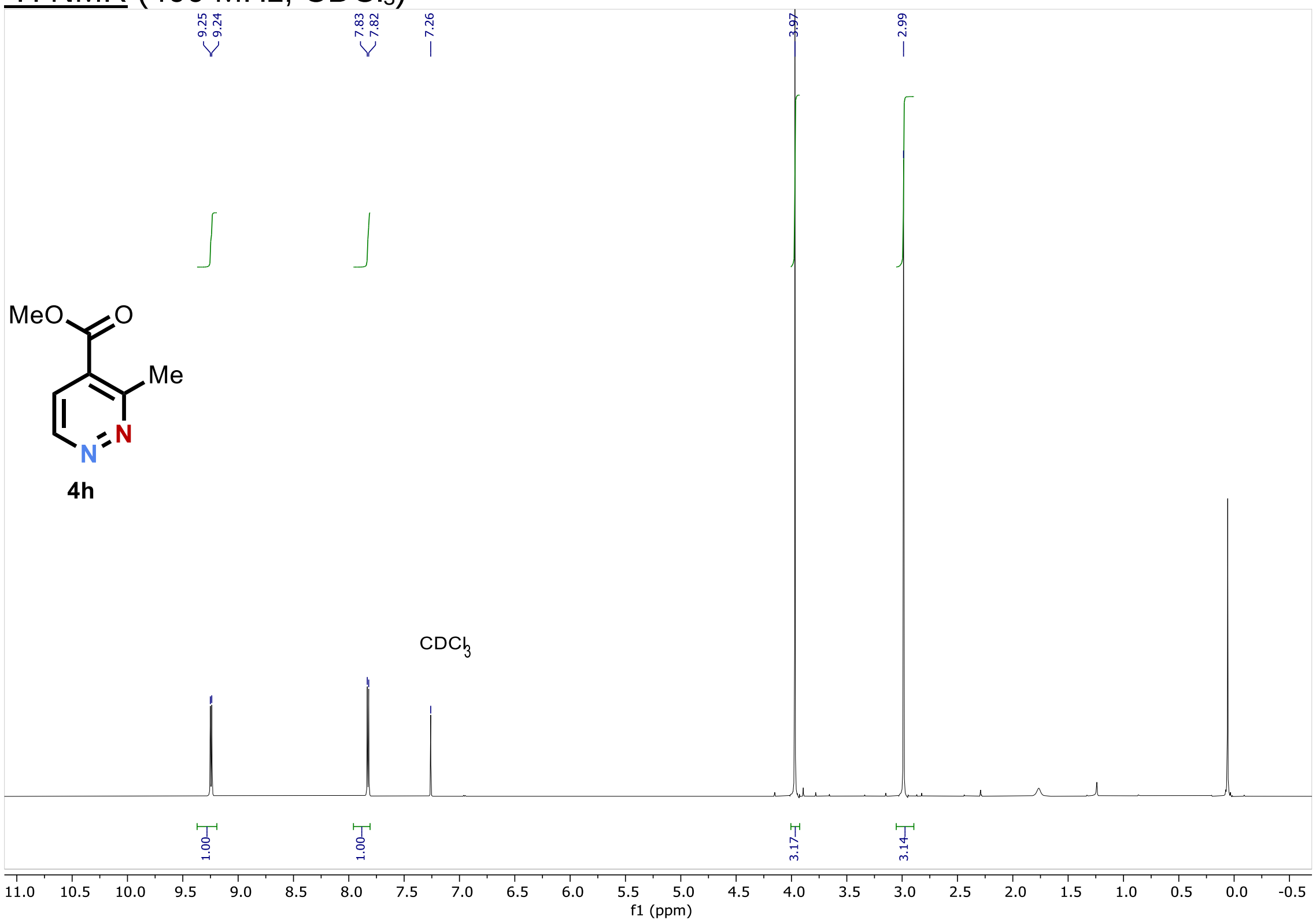

^{13}C{^{1}H} NMR (101 MHz, $CDCl_3$)

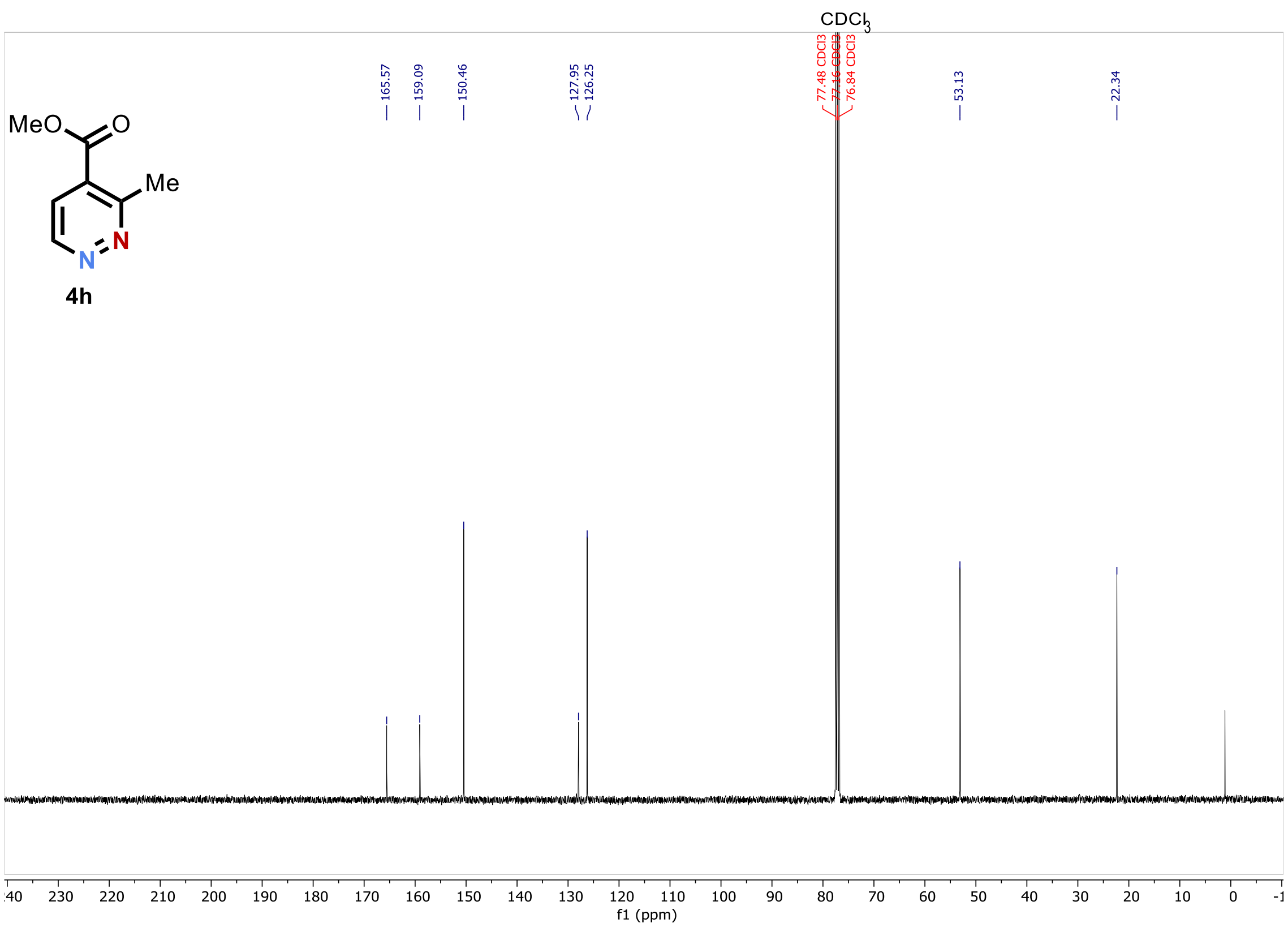

^{1}H NMR (400 MHz, MeOD)

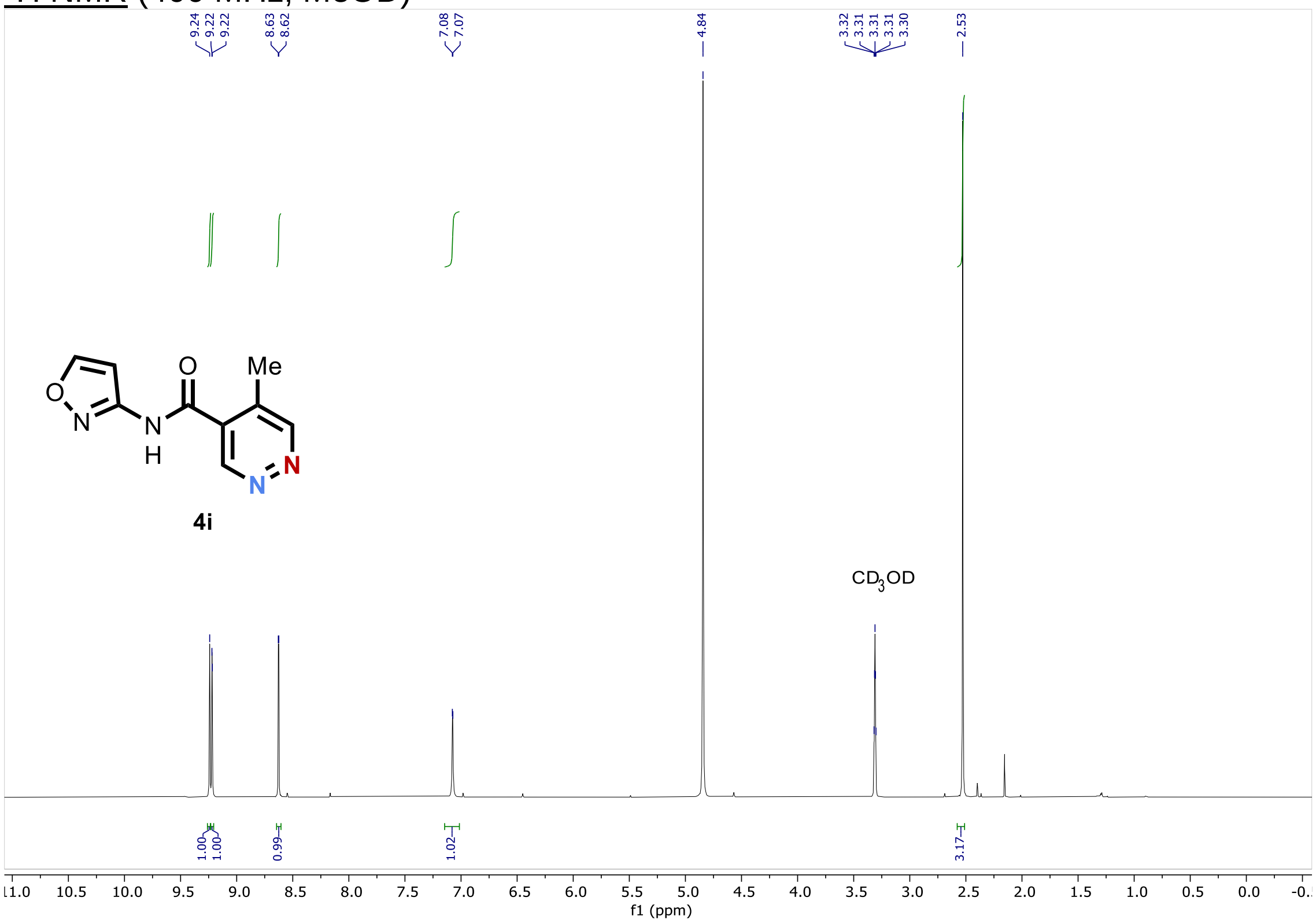

^{13}C{^{1}H} NMR (101 MHz, MeOD)

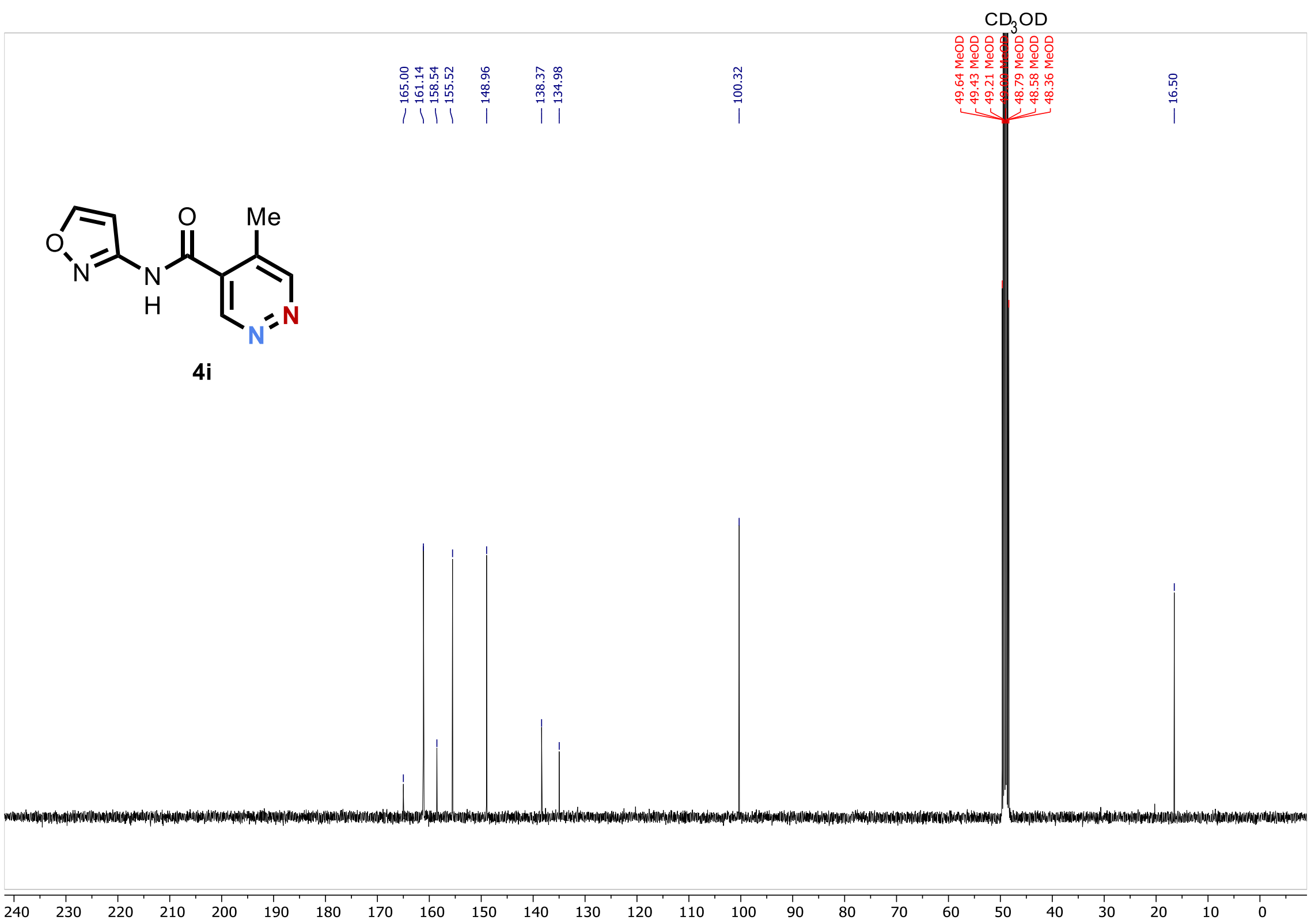

^{1}H NMR (400 MHz, $CDCl_3$)

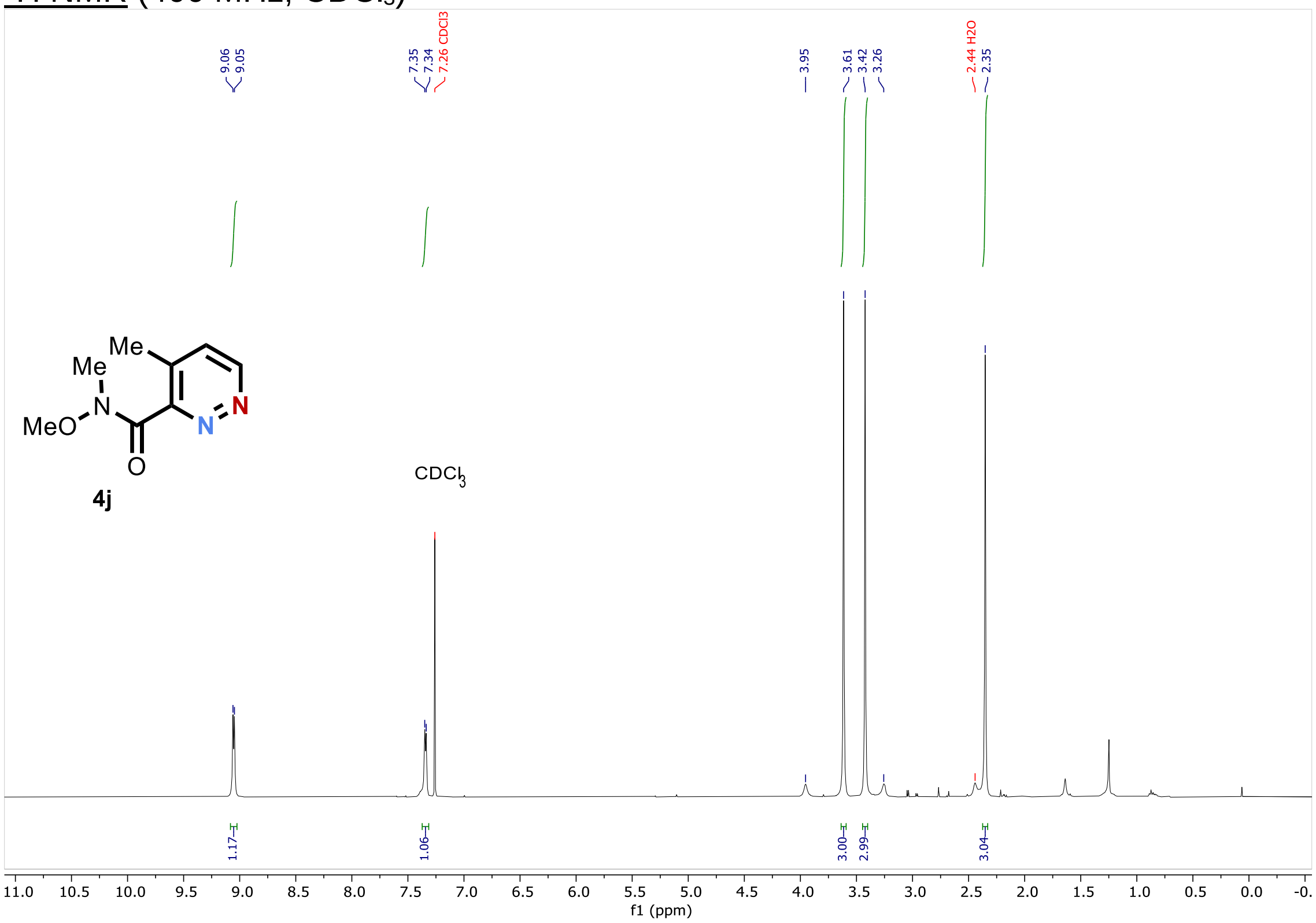

$^{13}C\{^{1}H\}$ NMR (101 MHz, $CDCl_3$)

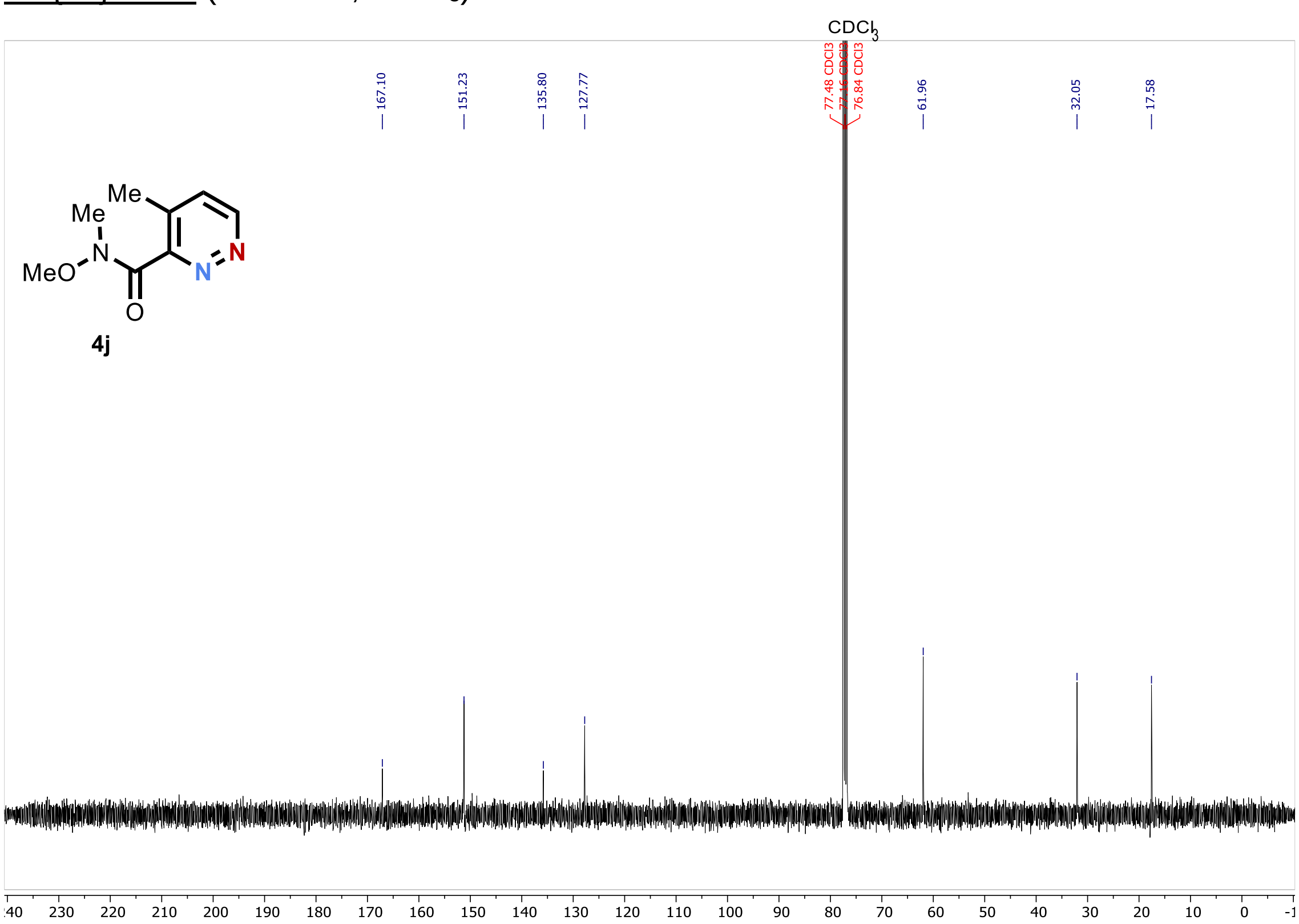

^{1}H NMR (400 MHz, $CDCl_3$)

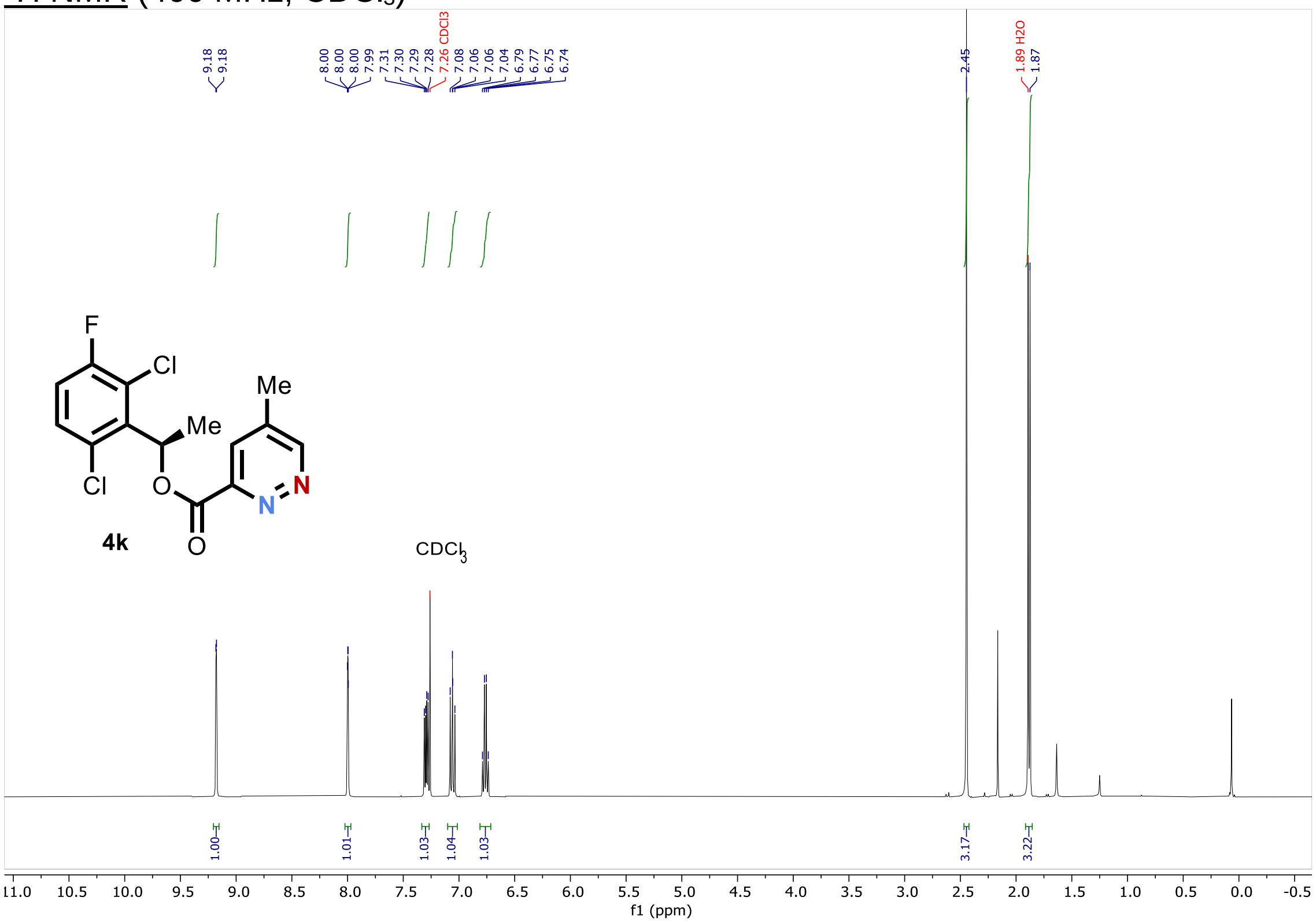

^{19}F NMR (376 MHz, $CDCl_3$)

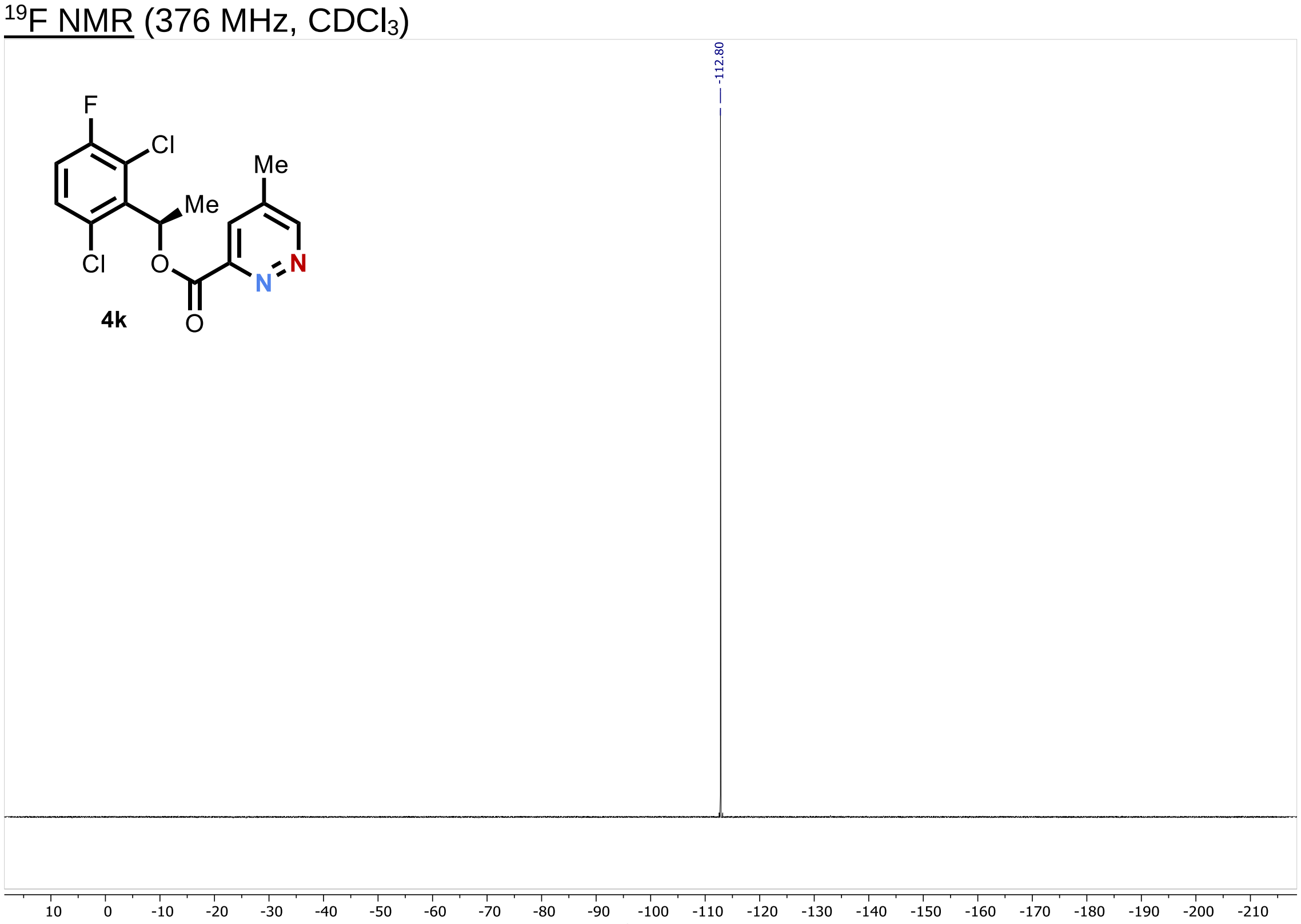

$^{13}C\{^{1}H\}$ NMR (101 MHz, $CDCl_3$)

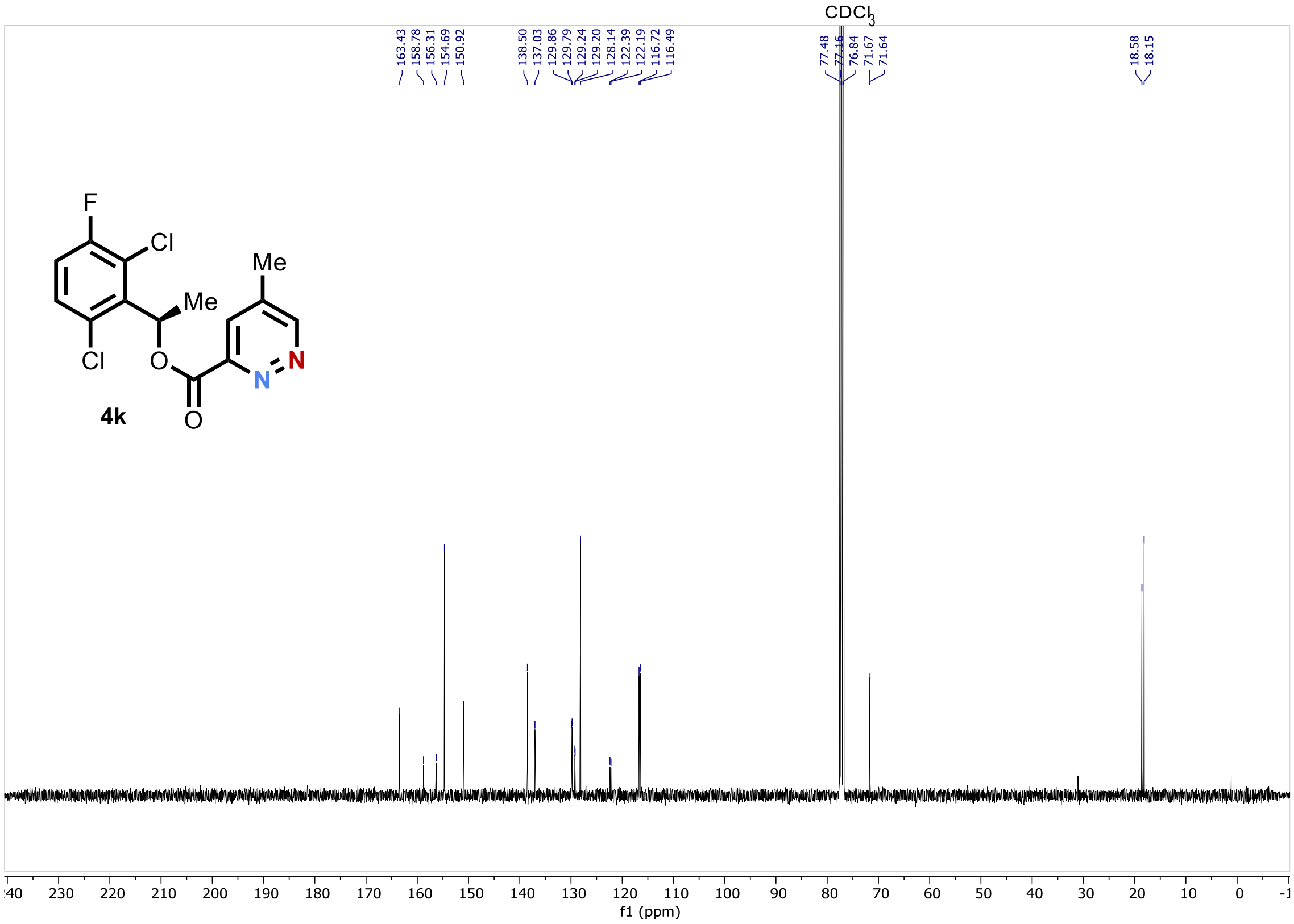

^{1}H NMR (400 MHz, $CDCl_3$)

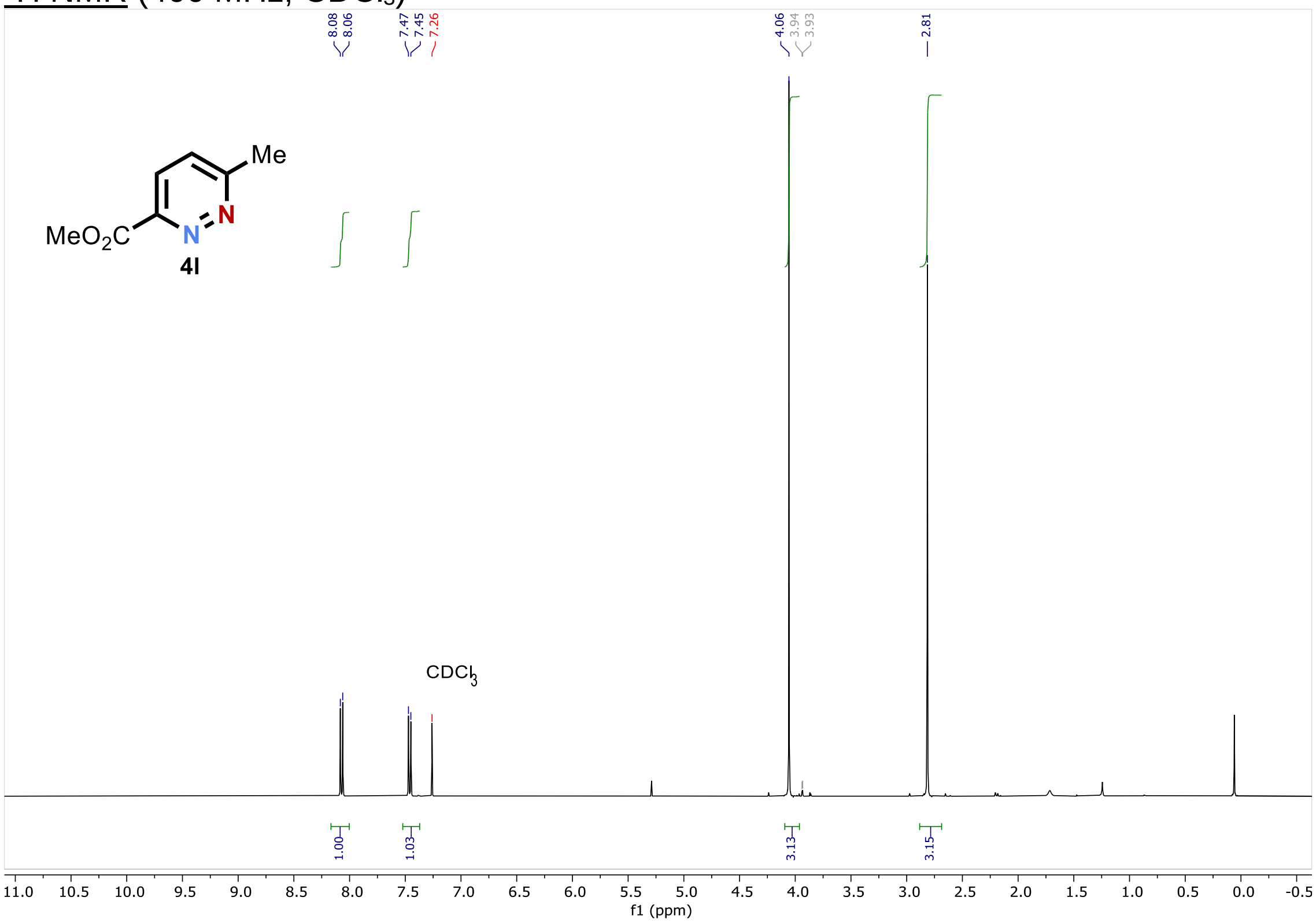

^{13}C{^{1}H} NMR (101 MHz, $CDCl_3$)

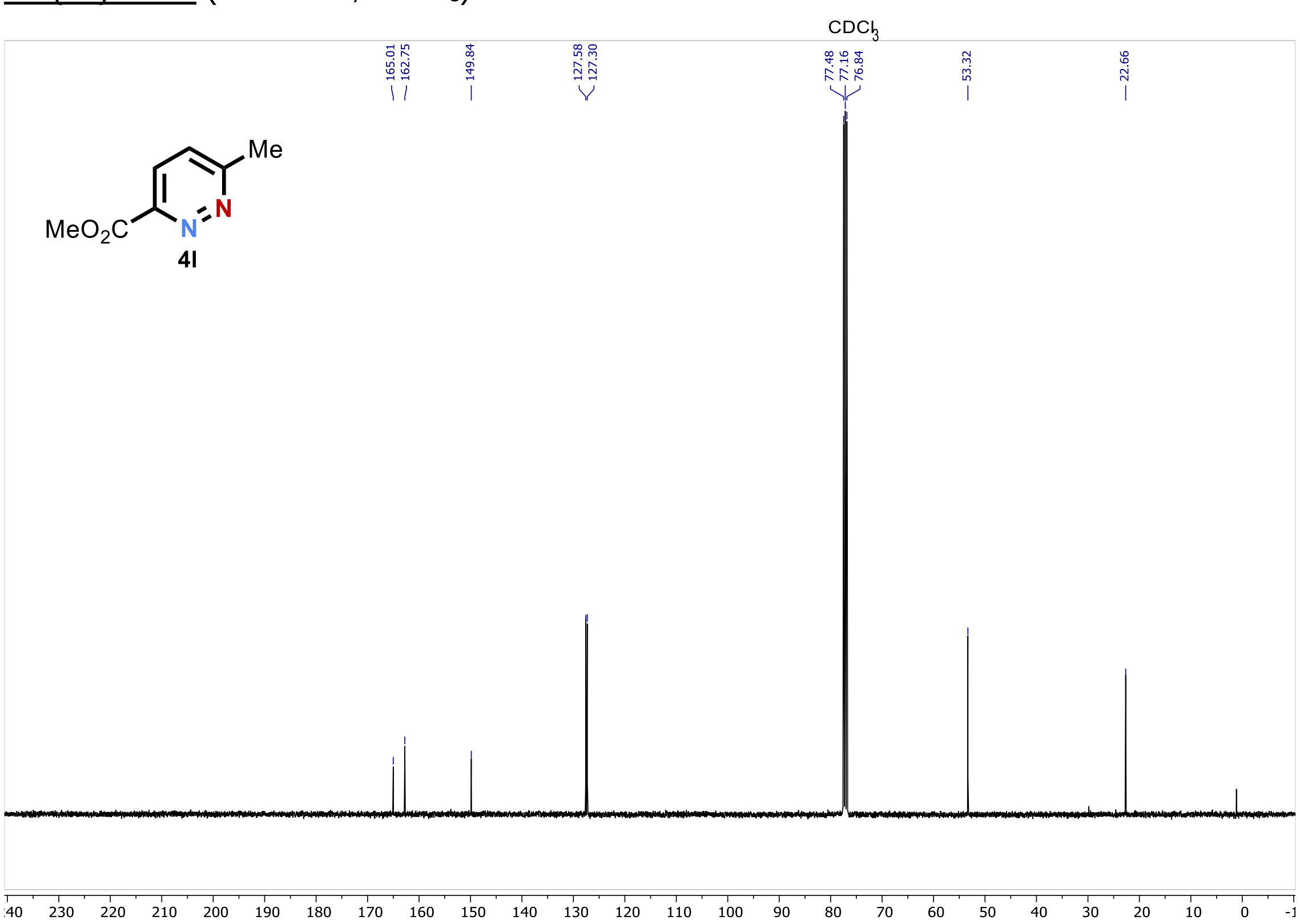

^{1}H NMR (400 MHz, $CDCl_3$)

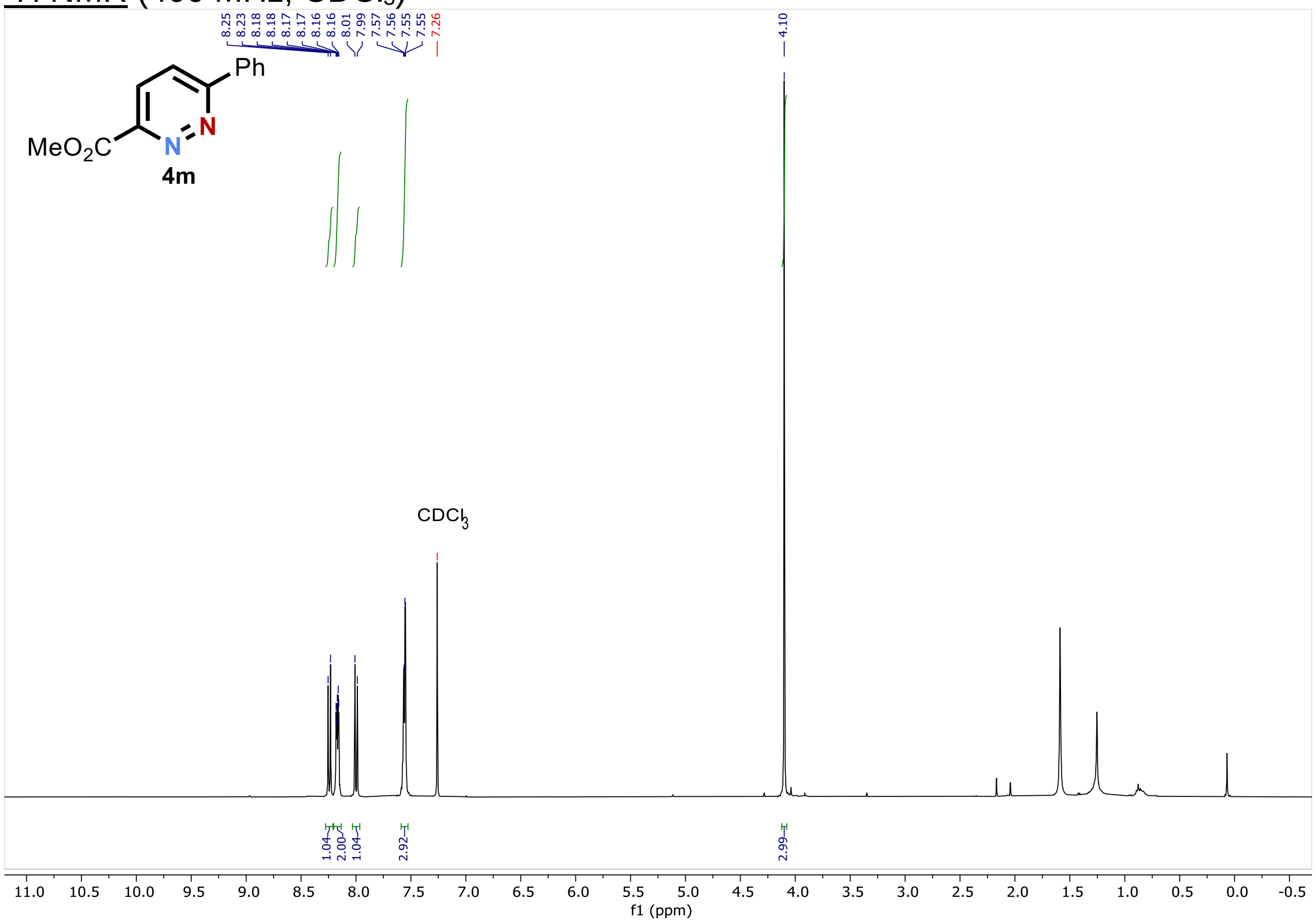

$^{13}C\{^{1}H\}$ NMR (101 MHz, $CDCl_3$)

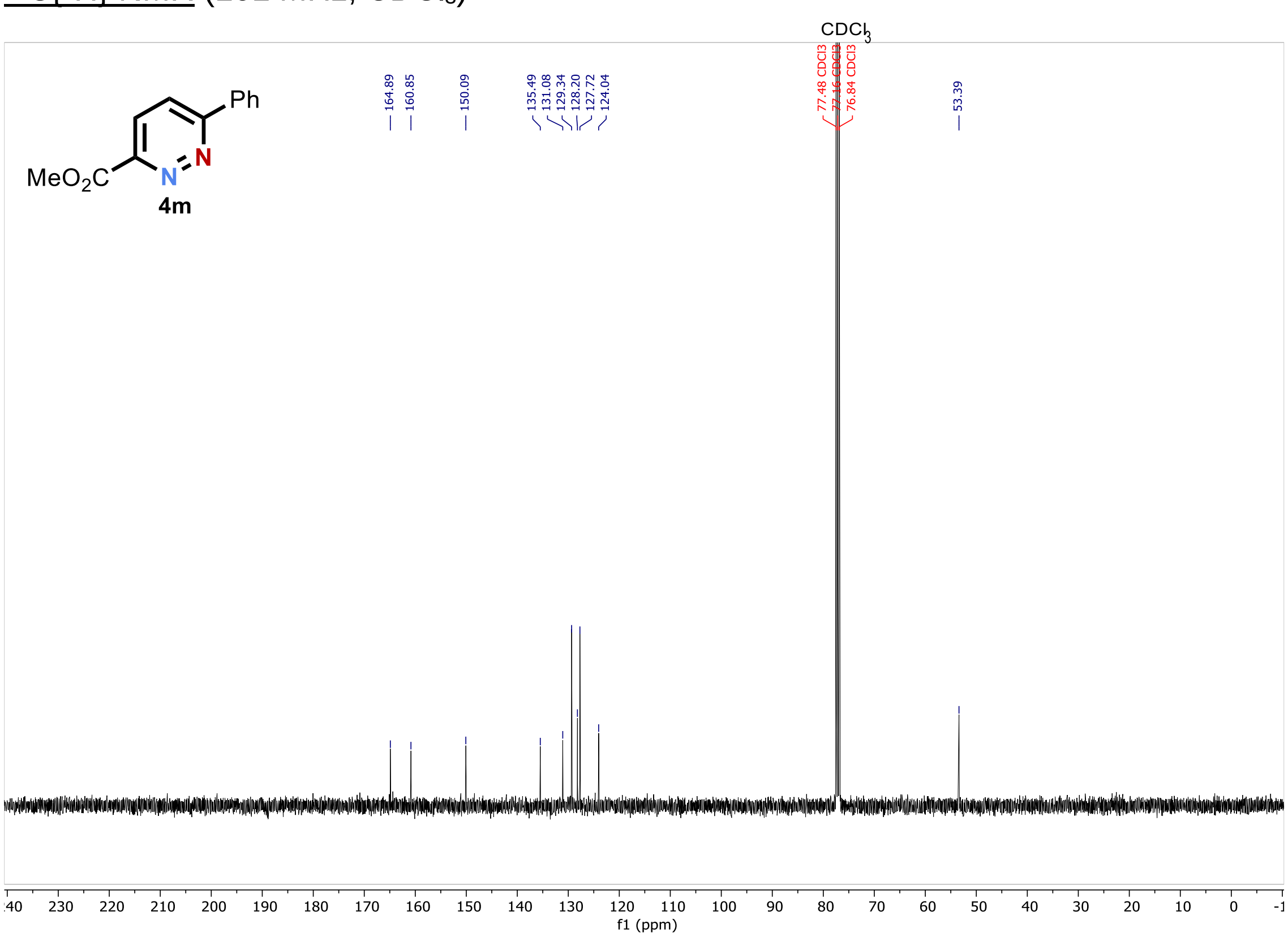

^{1}H NMR (500 MHz, $CDCl_3$)

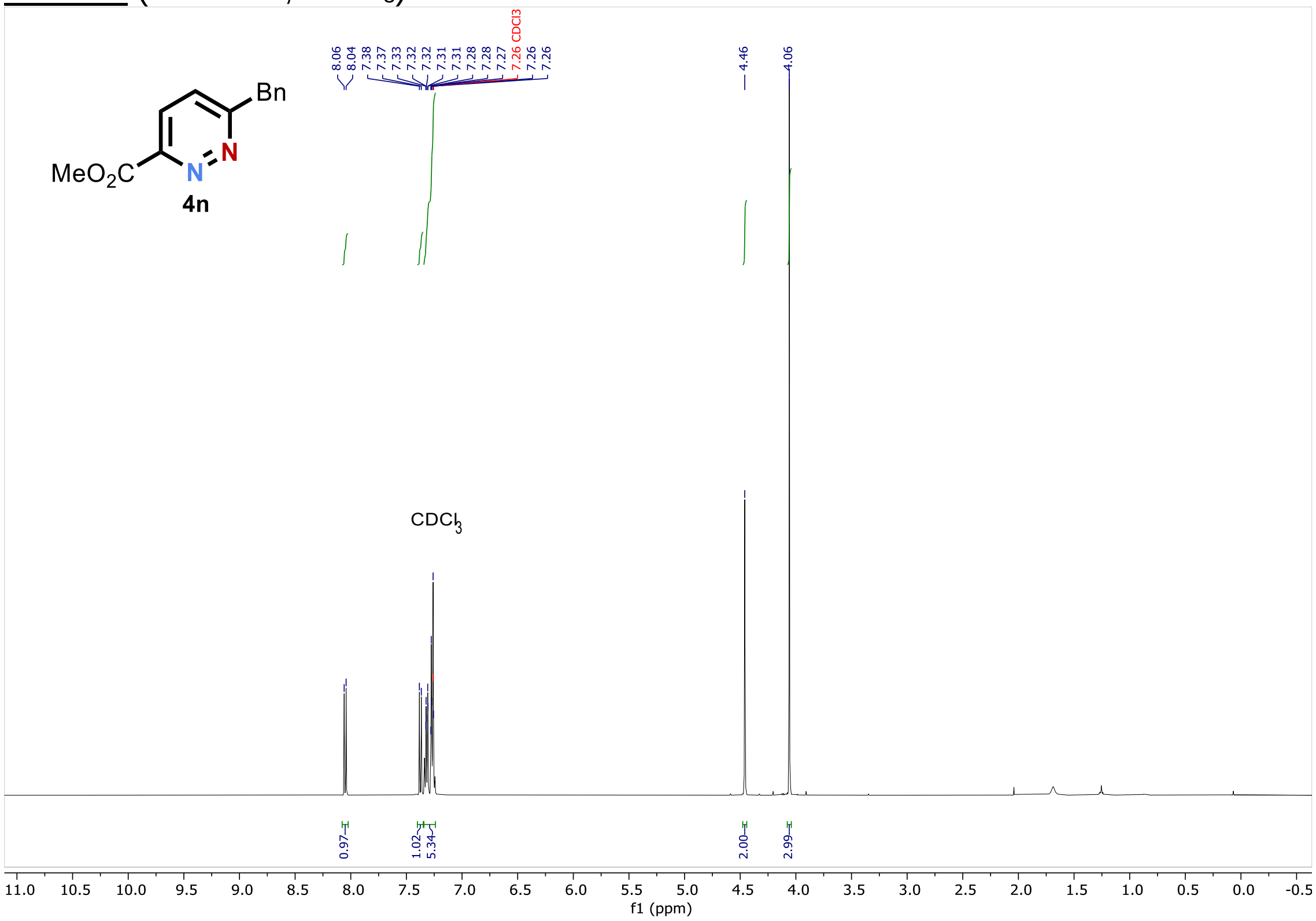

^{13}C{^{1}H} NMR (126 MHz, $CDCl_3$)

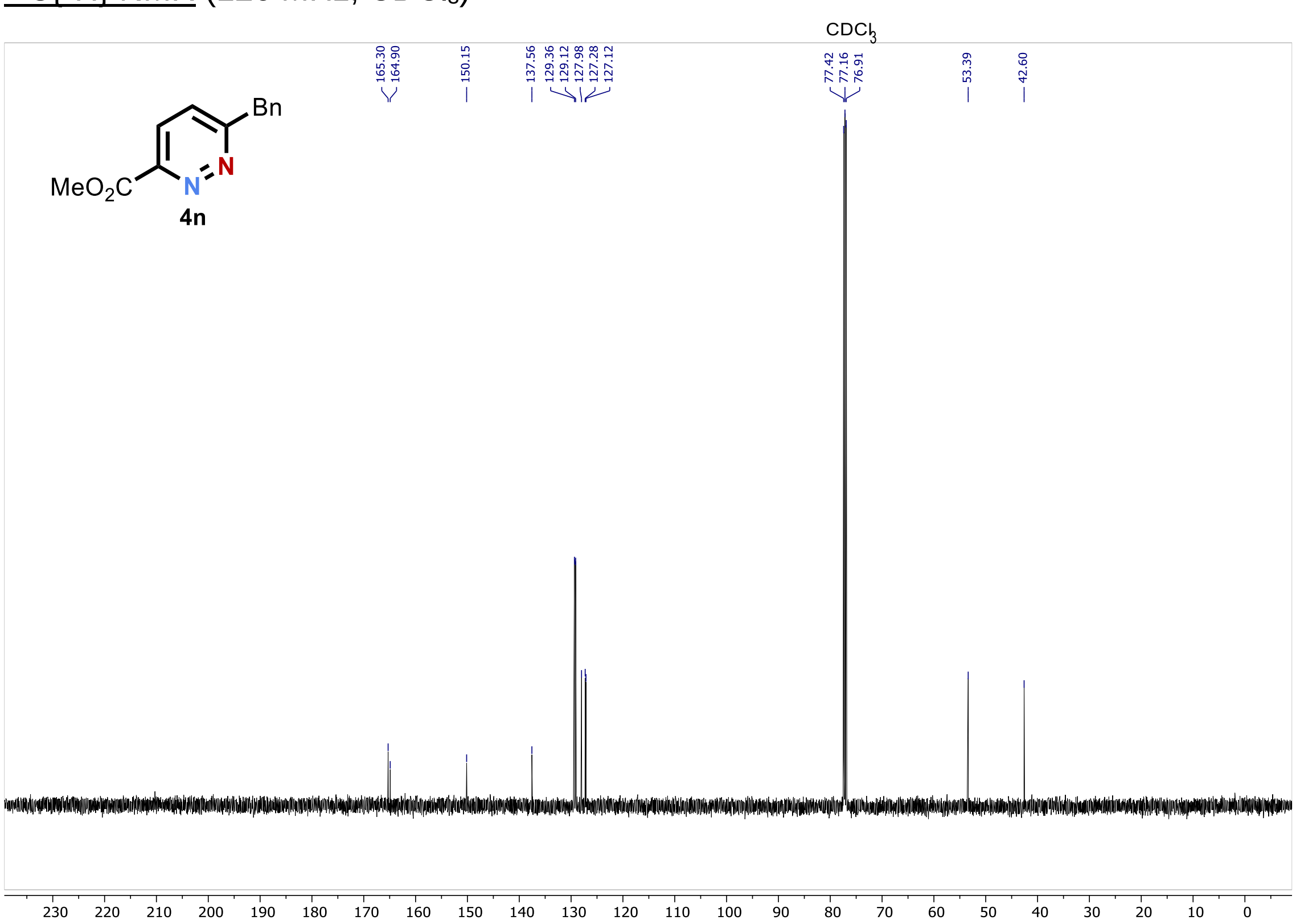

^{1}H NMR (500 MHz, $CDCl_3$)

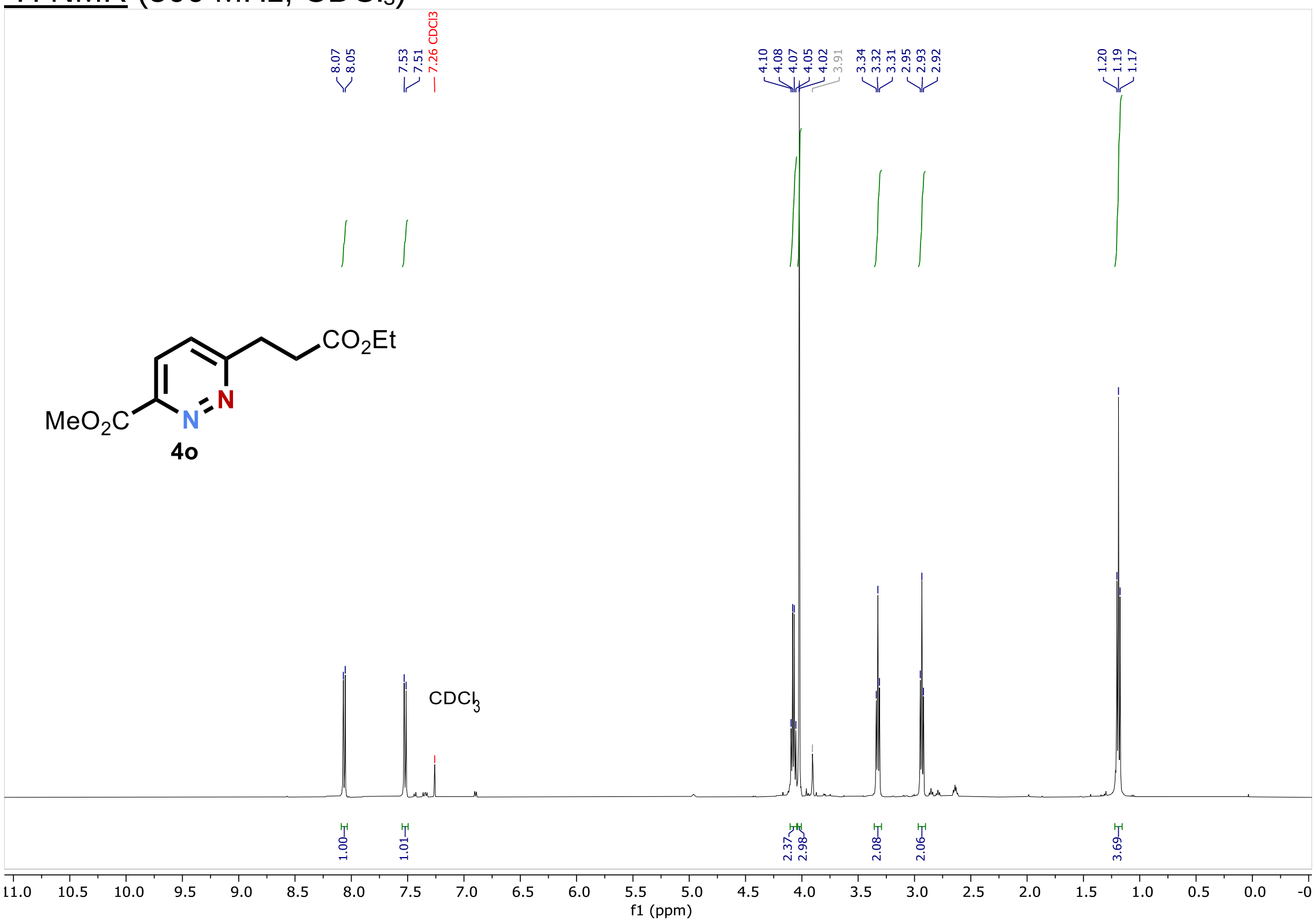

$^{13}C\{^{1}H\}$ NMR (126 MHz, $CDCl_3$)

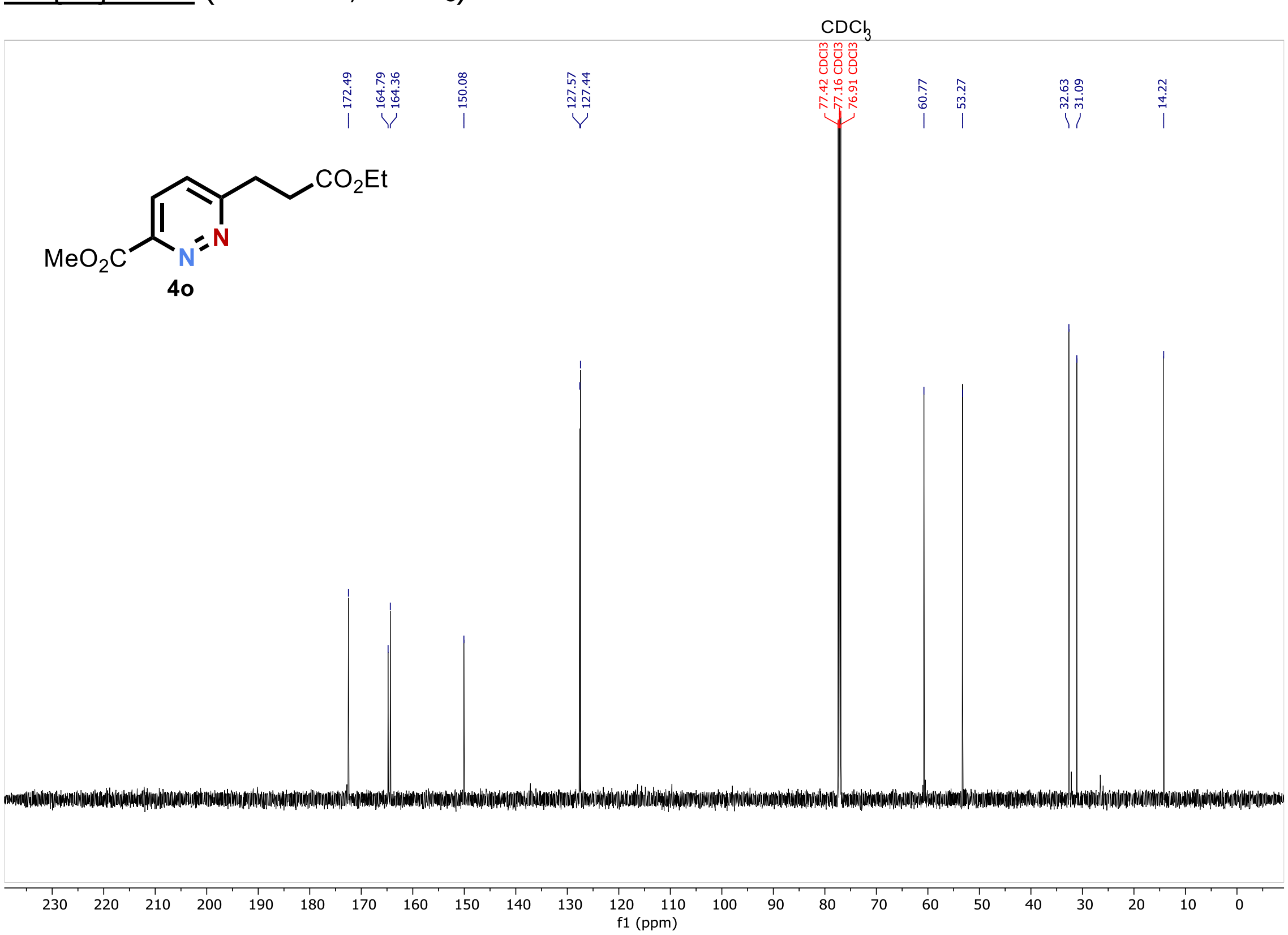

^{1}H NMR (500 MHz, $CDCl_3$)

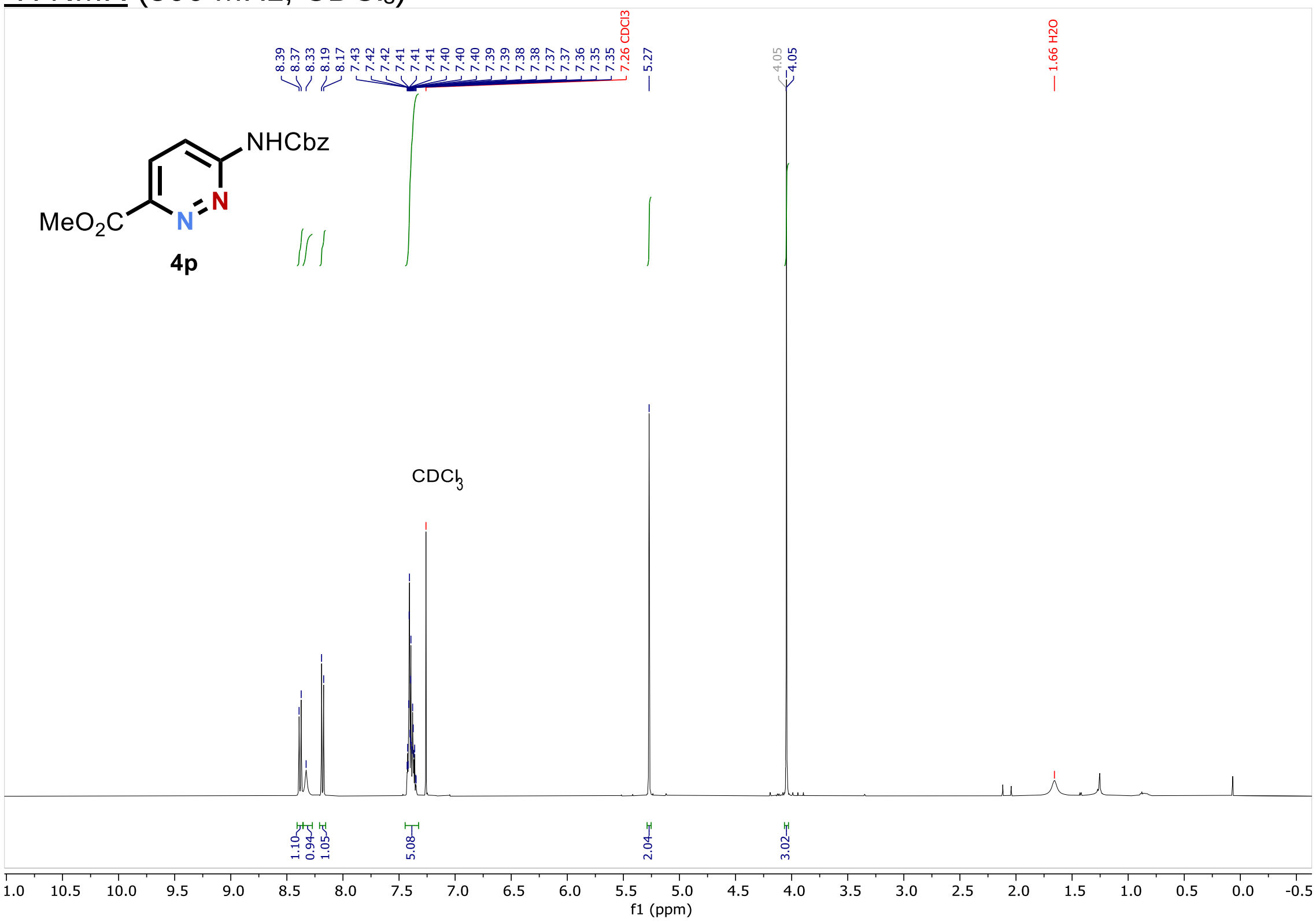

$^{13}C\{^{1}H\}$ NMR (101 MHz, $CDCl_3$)

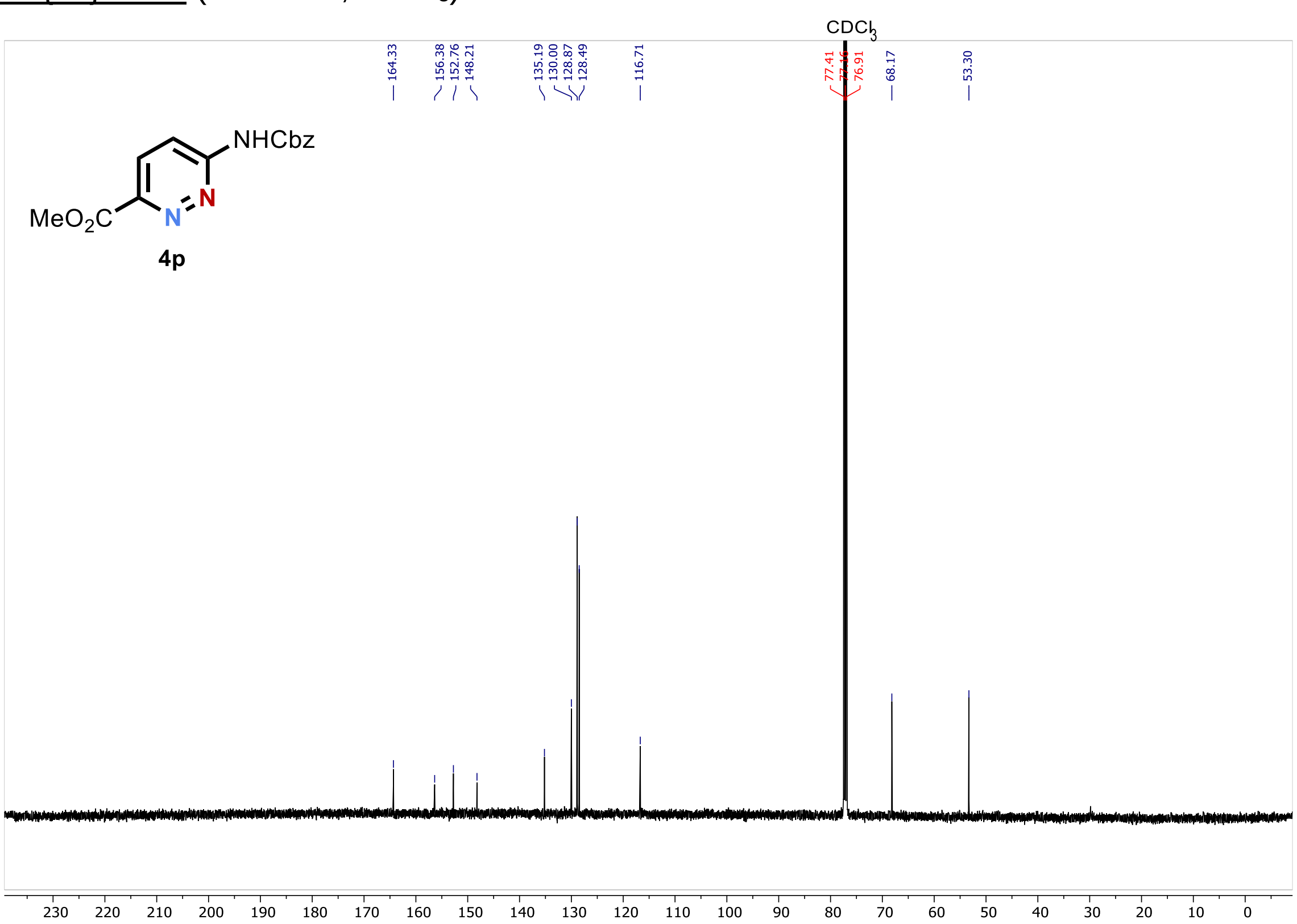

^{1}H NMR (400 MHz, $CDCl_3$)

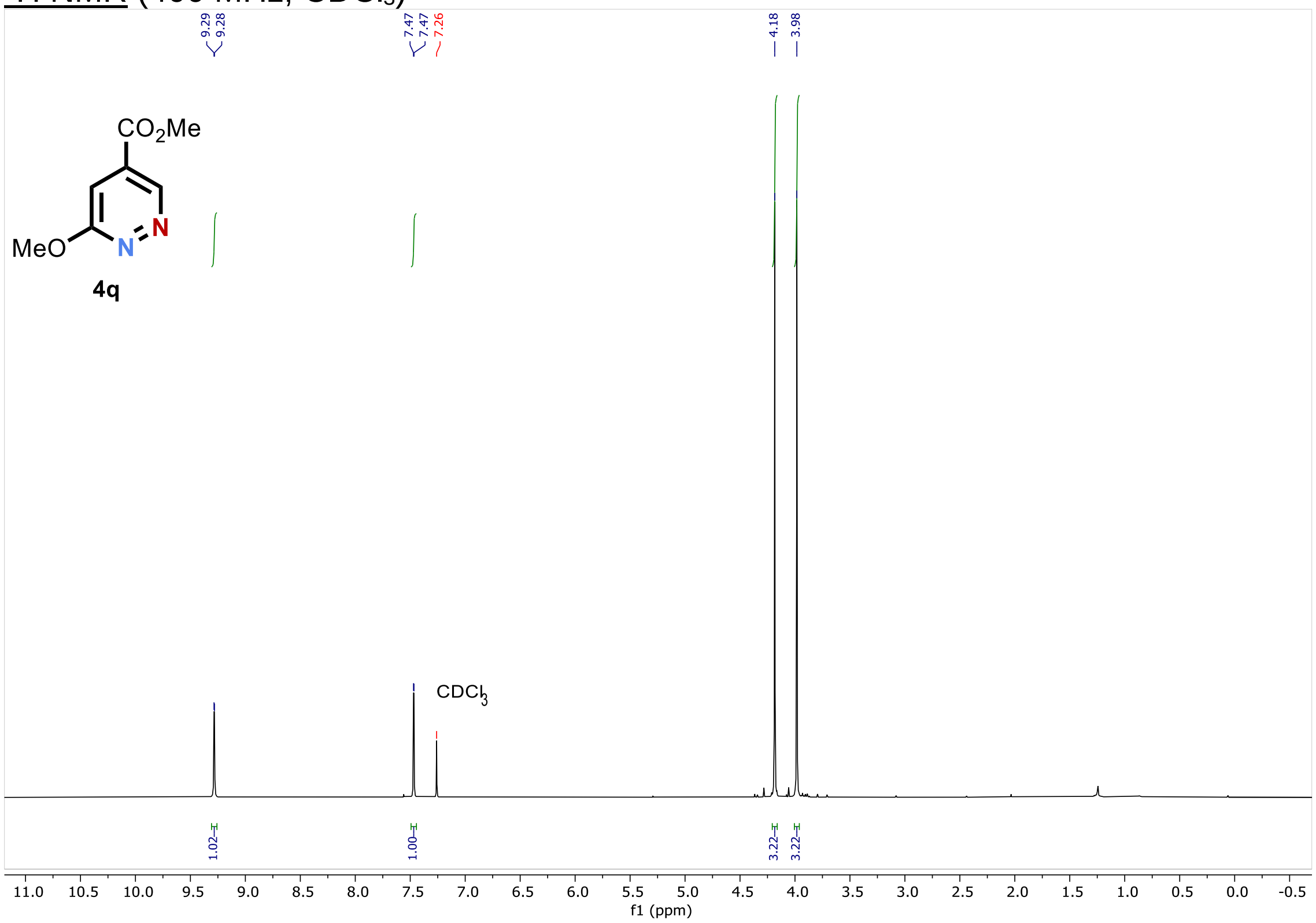

^{13}C{^{1}H} NMR (101 MHz, $CDCl_3$)

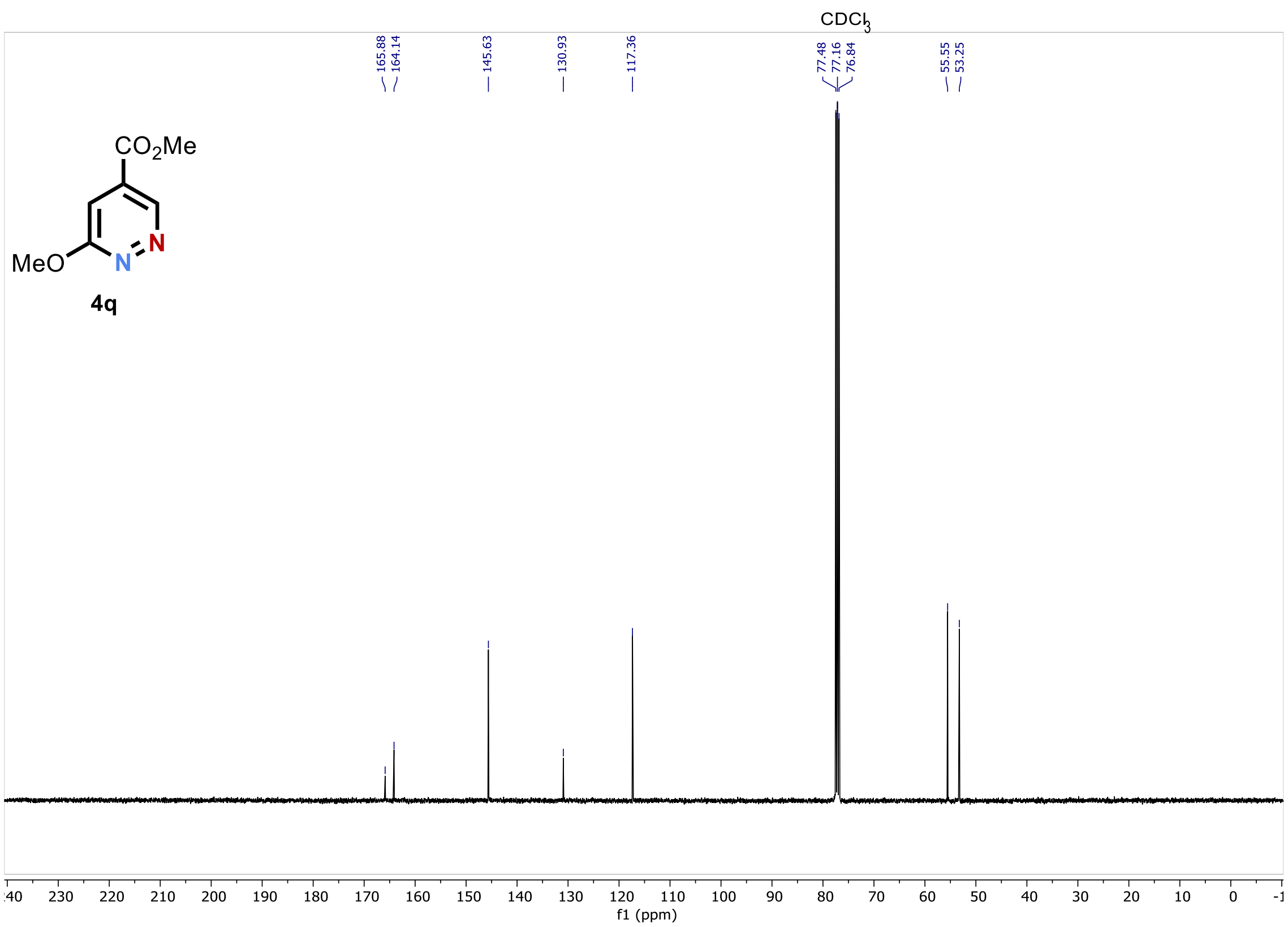

^{1}H NMR (400 MHz, $CDCl_3$)

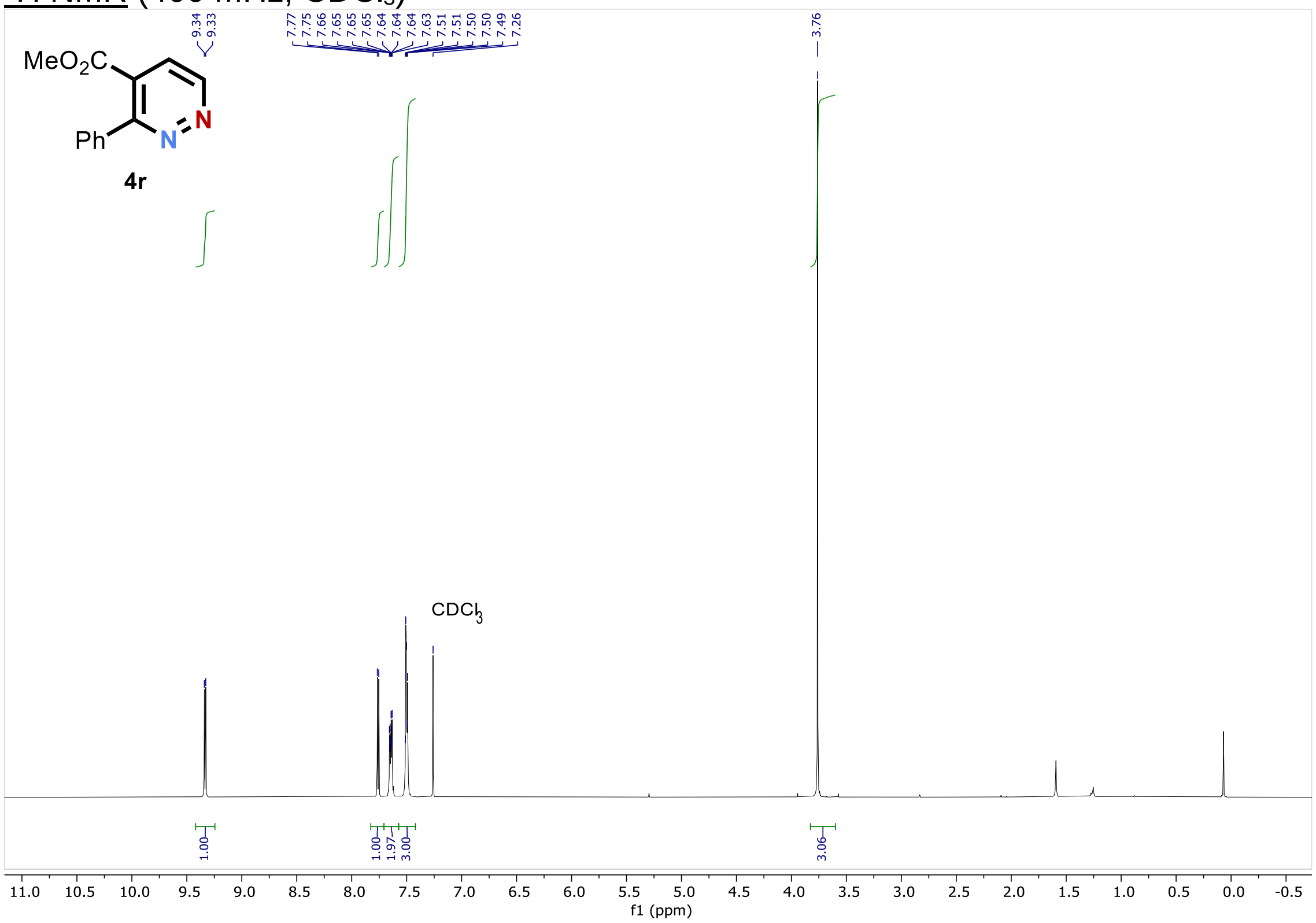

^{13}C{^{1}H} NMR (101 MHz, $CDCl_3$)

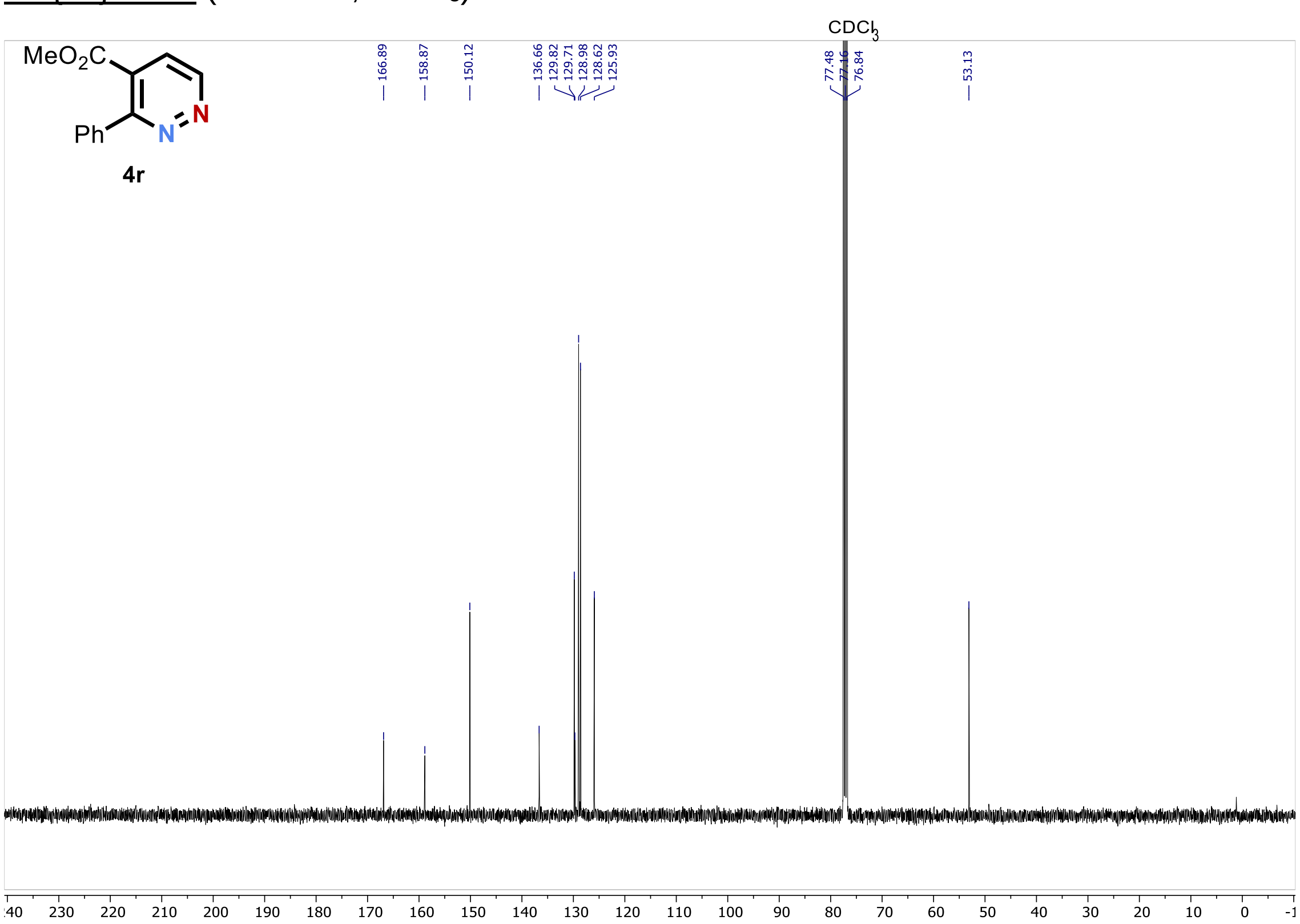

^{1}H NMR (400 MHz, $CDCl_3$)

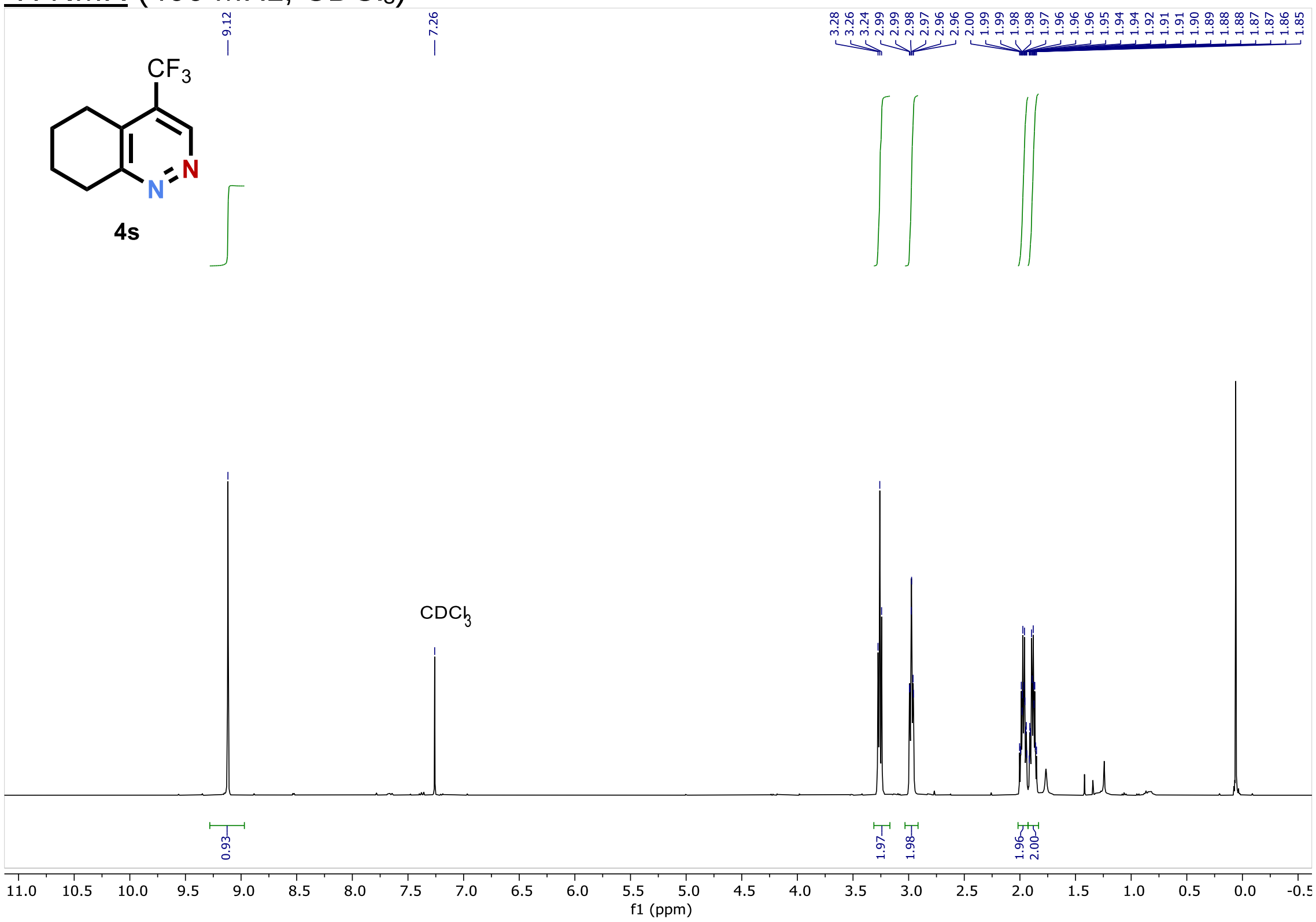

^{19}F NMR (376 MHz, $CDCl_3$)

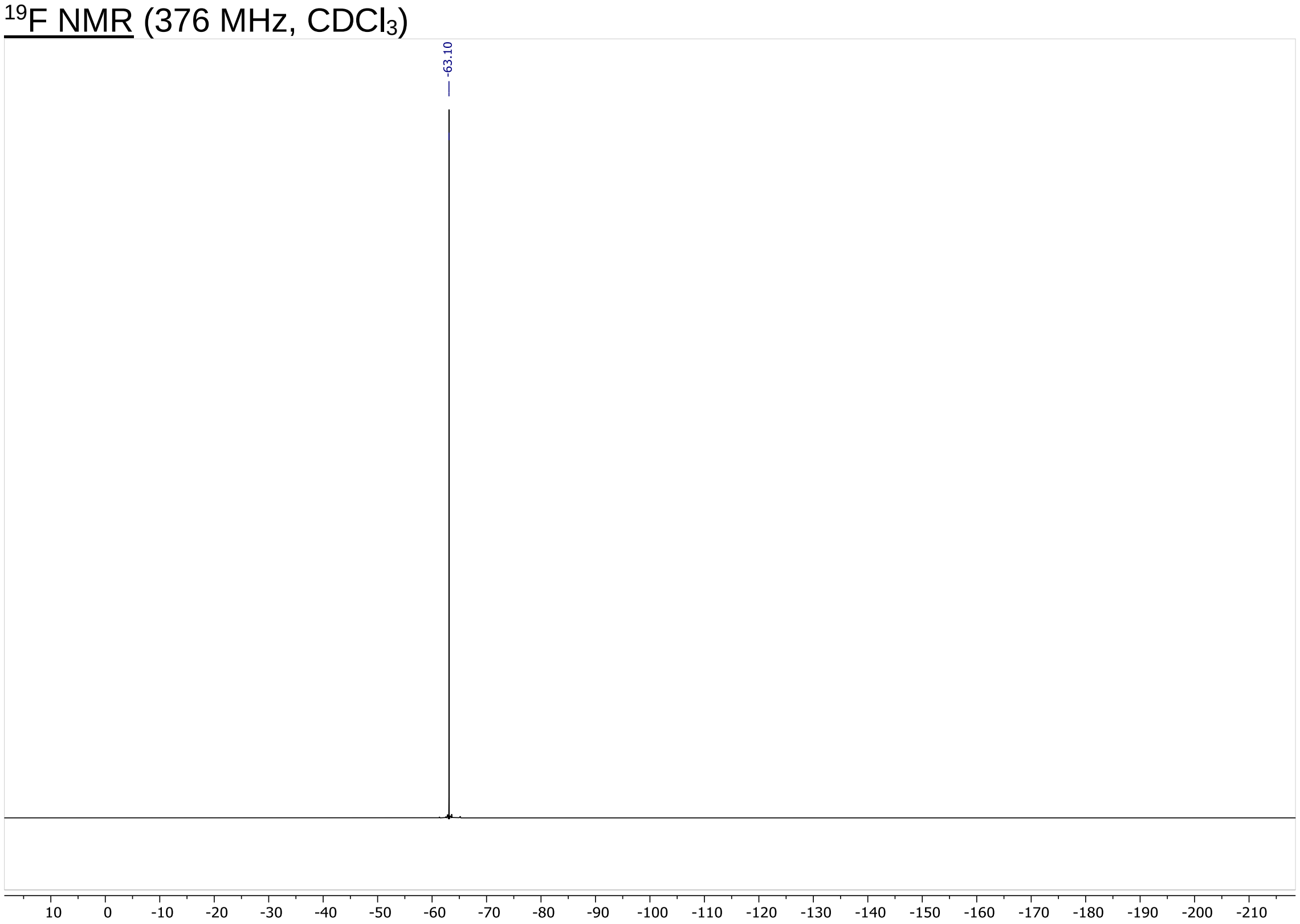

$^{13}C\{^1H\}$ NMR (101 MHz, $CDCl_3$)

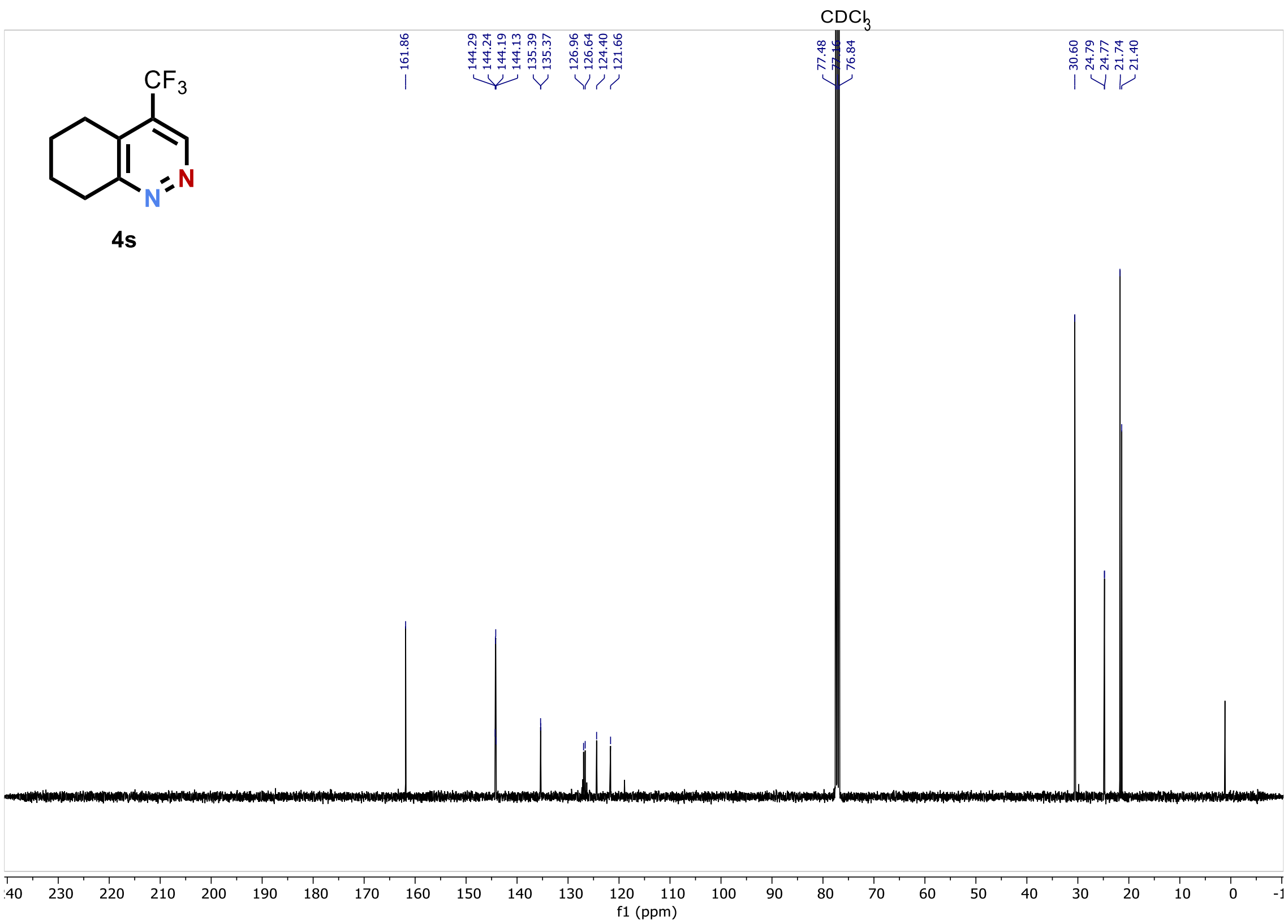

^{1}H NMR (400 MHz, $CDCl_3$)

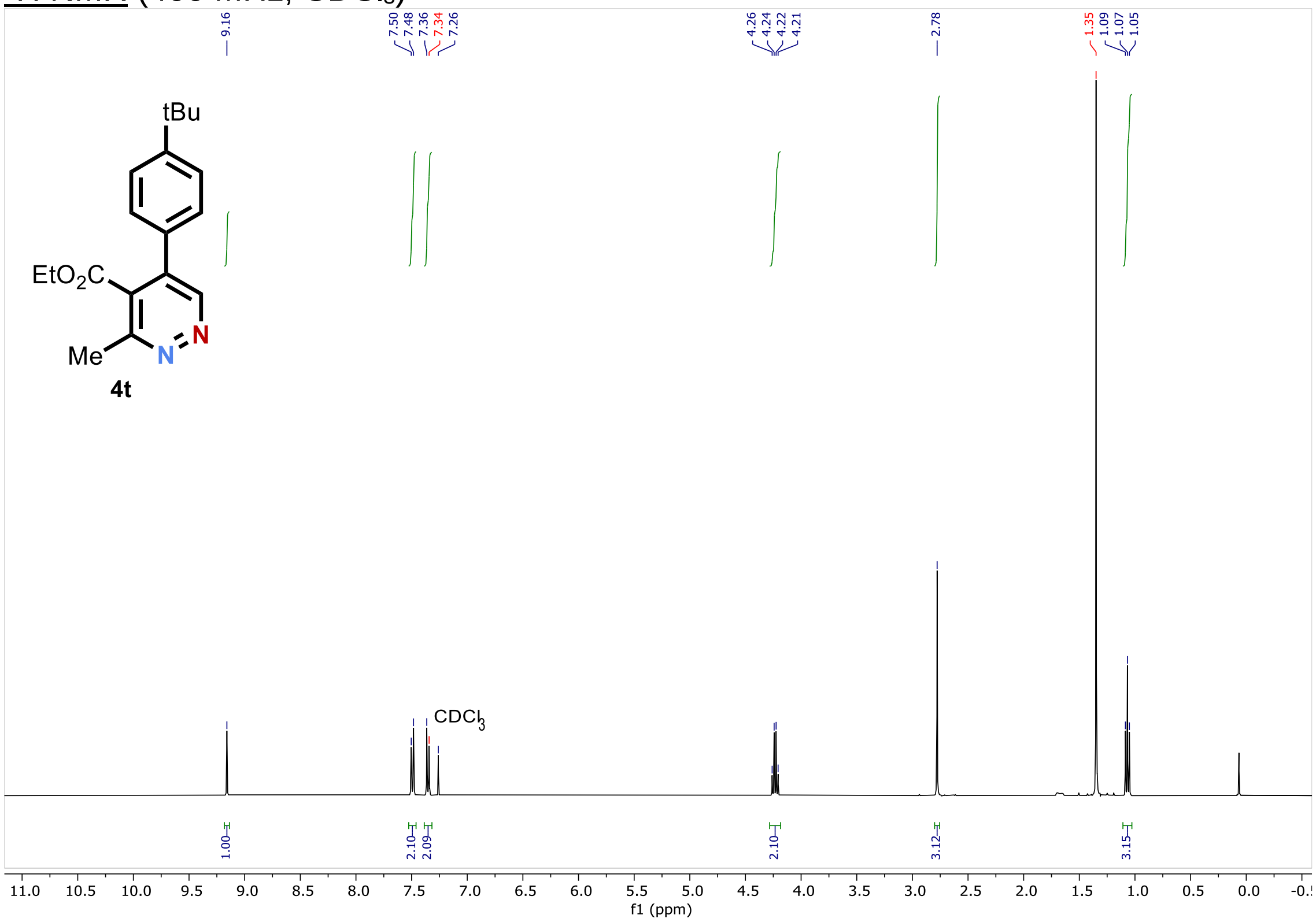

$^{13}C\{^{1}H\}$ NMR (101 MHz, $CDCl_3$)

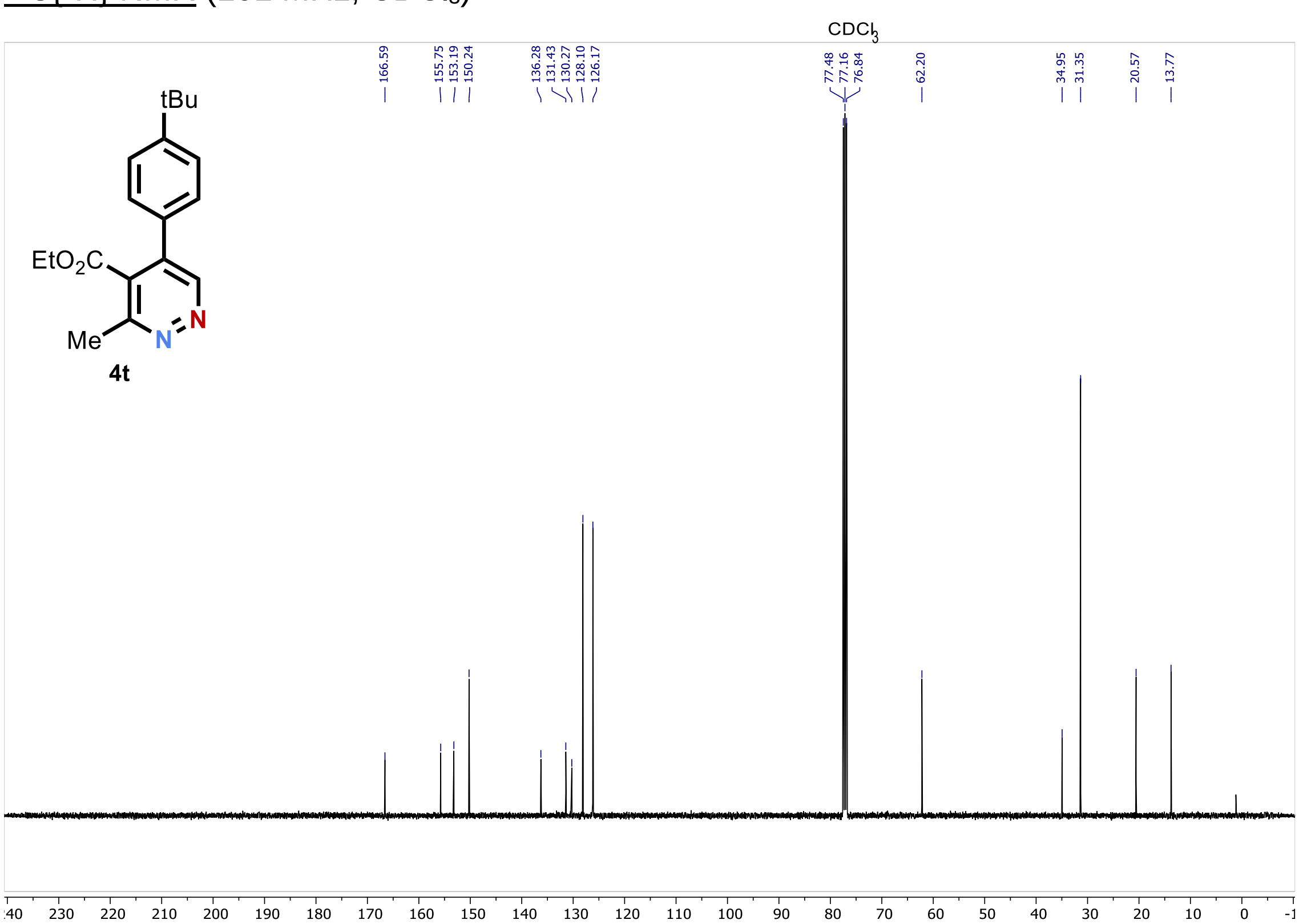

^{1}H NMR (400 MHz, $CDCl_3$)

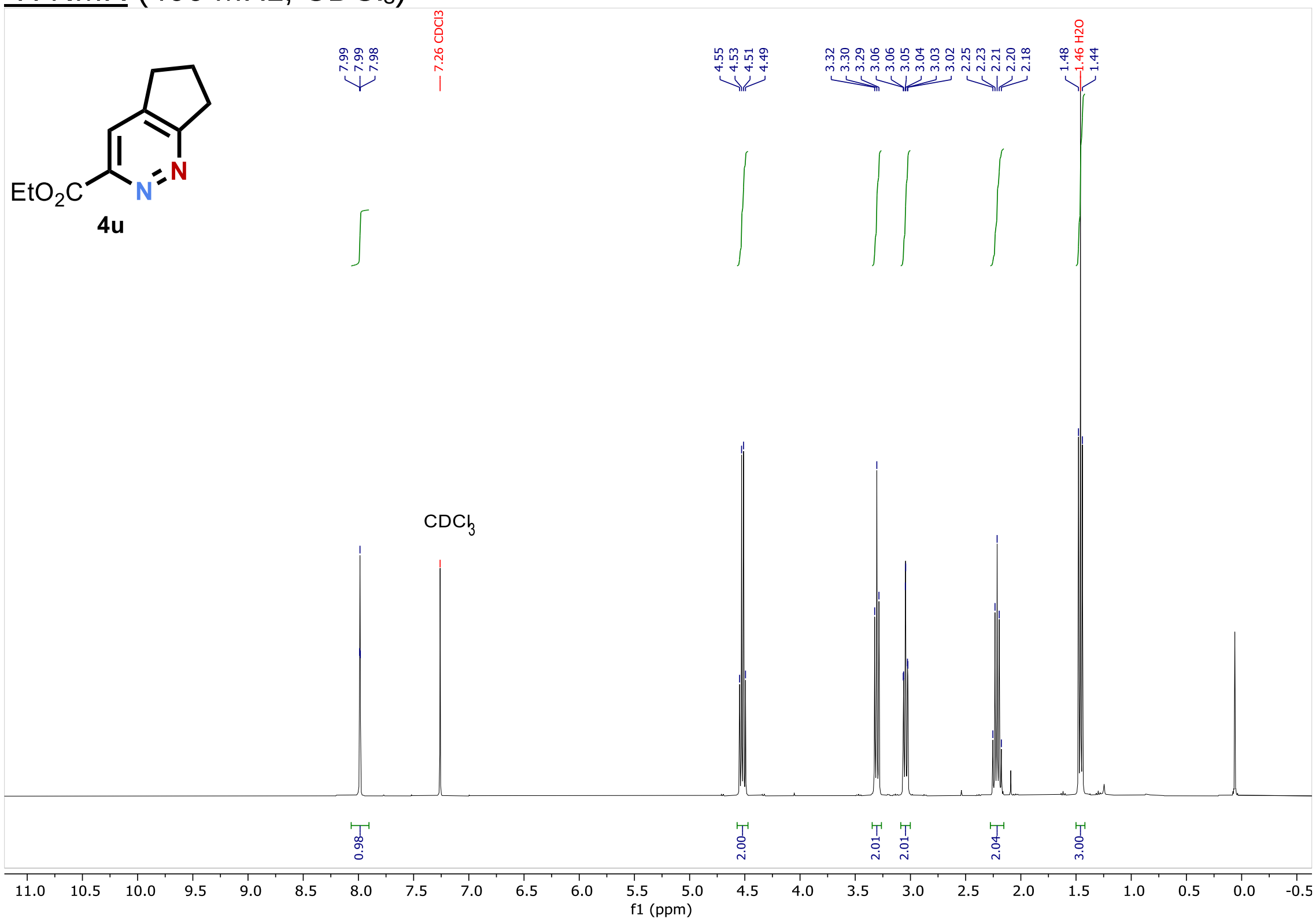

^{13}C{^{1}H} NMR (101 MHz, $CDCl_3$)

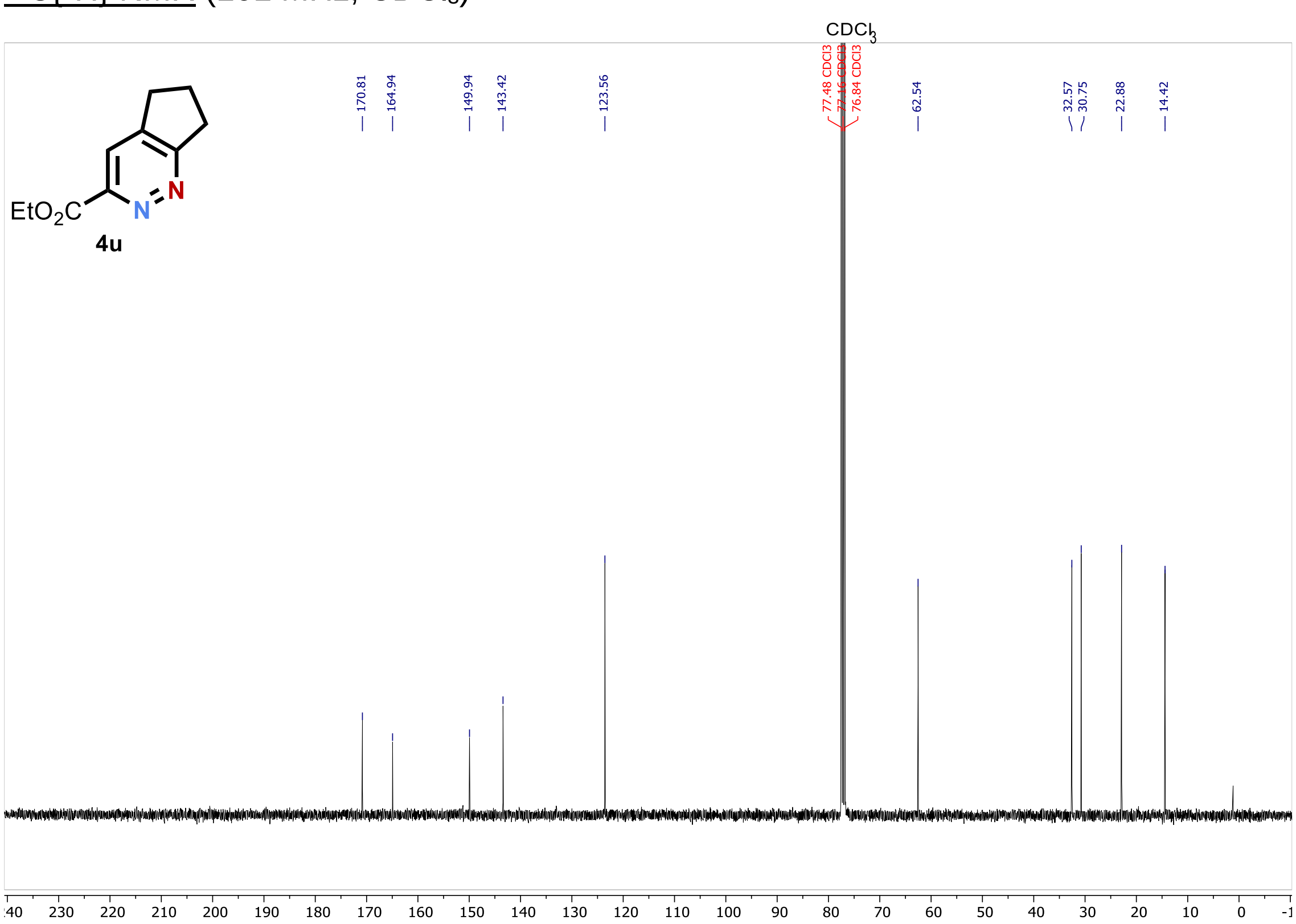

^{1}H NMR (400 MHz, $CDCl_3$)

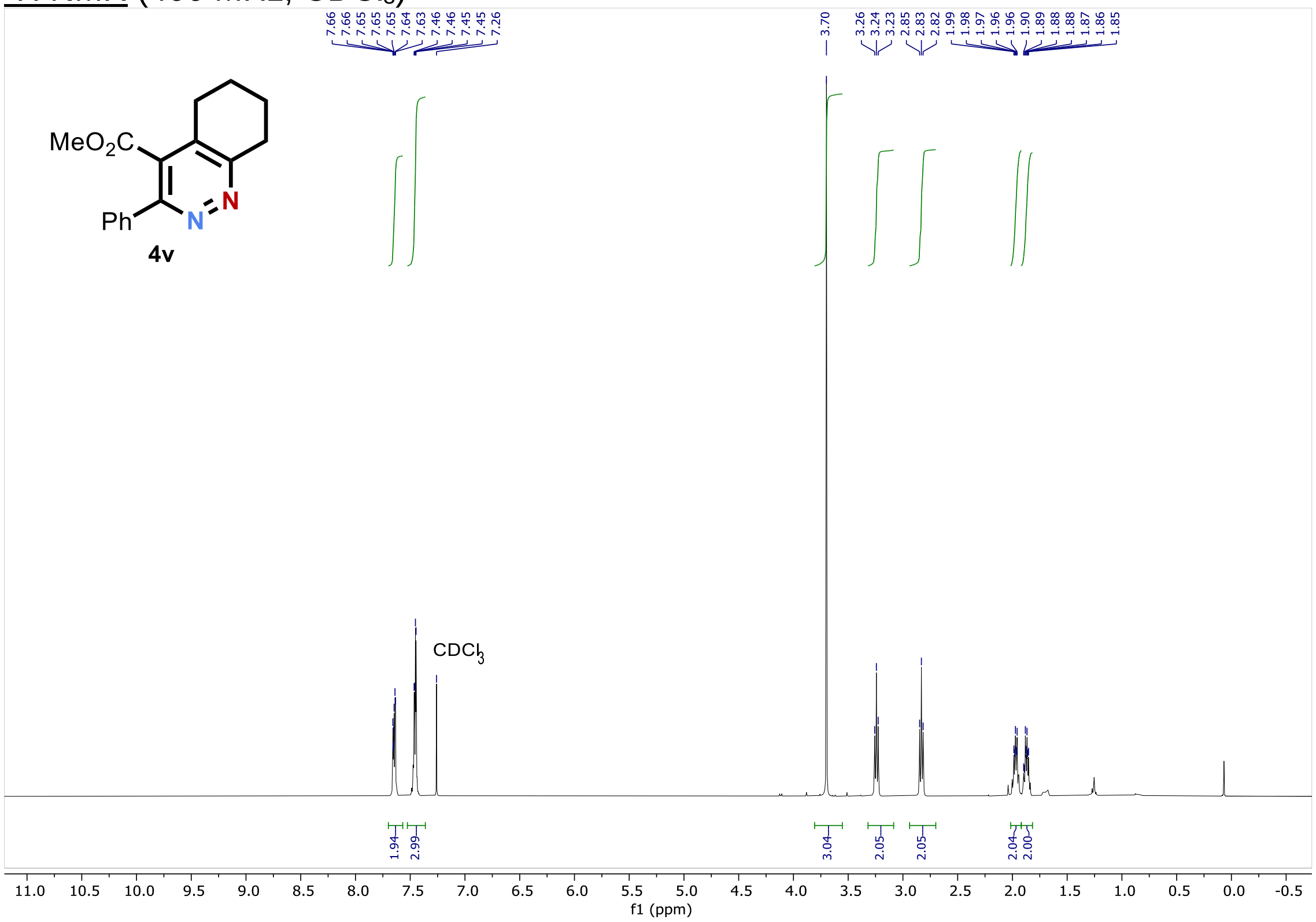

^{13}C{^{1}H} NMR (101 MHz, $CDCl_3$)

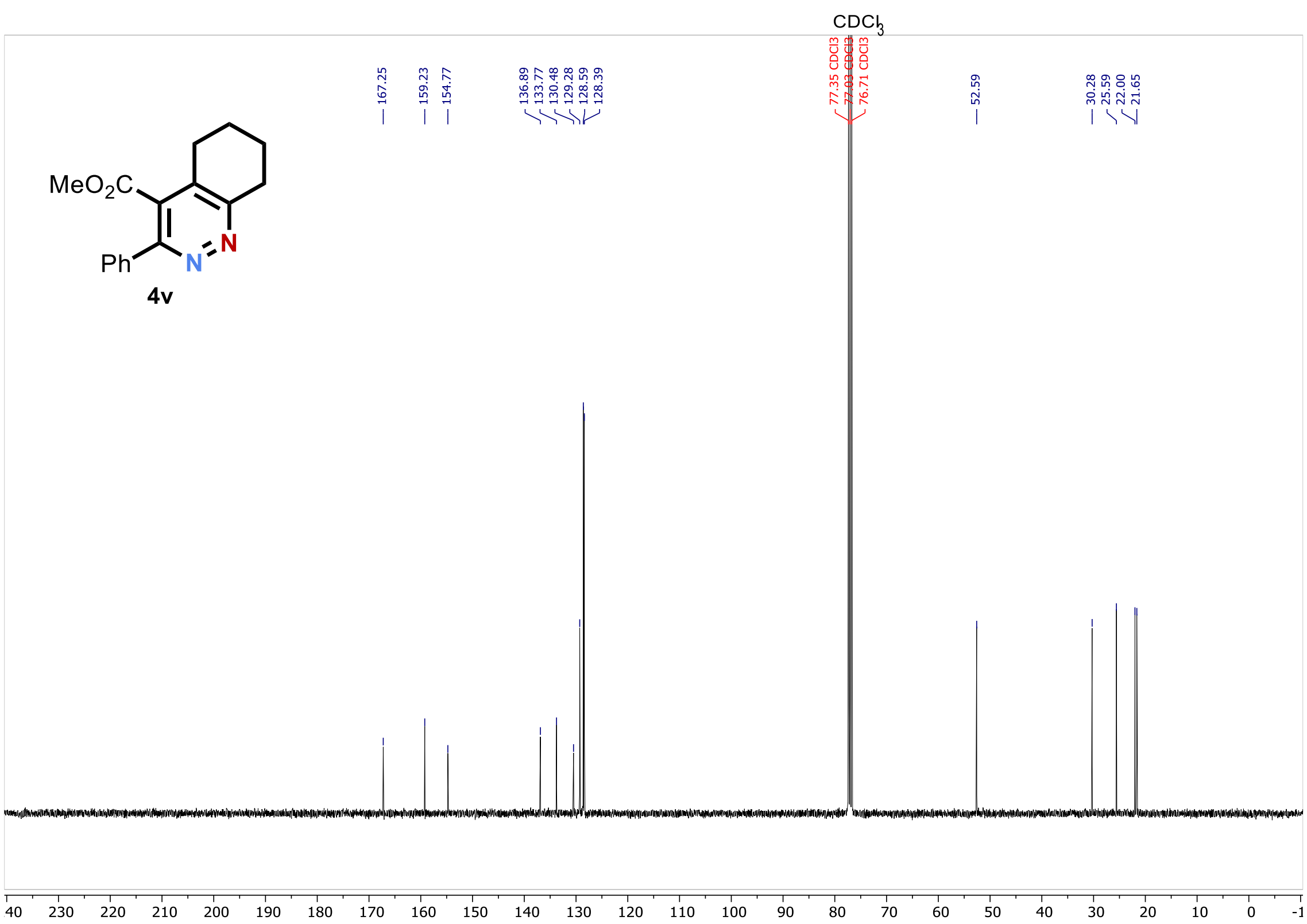

^{1}H NMR (400 MHz, $CDCl_3$)

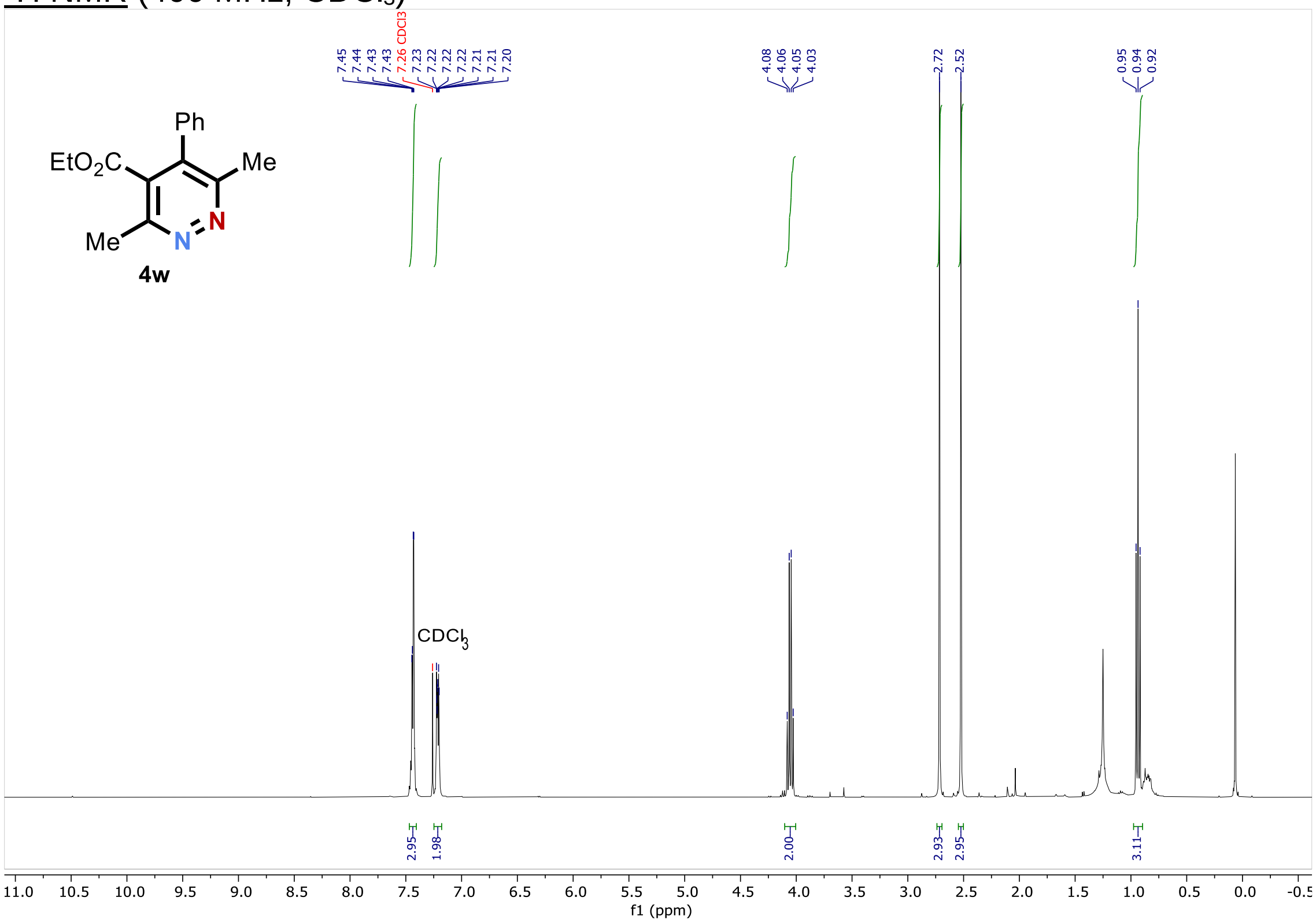

$^{13}C\{^{1}H\}$ NMR (101 MHz, $CDCl_3$)

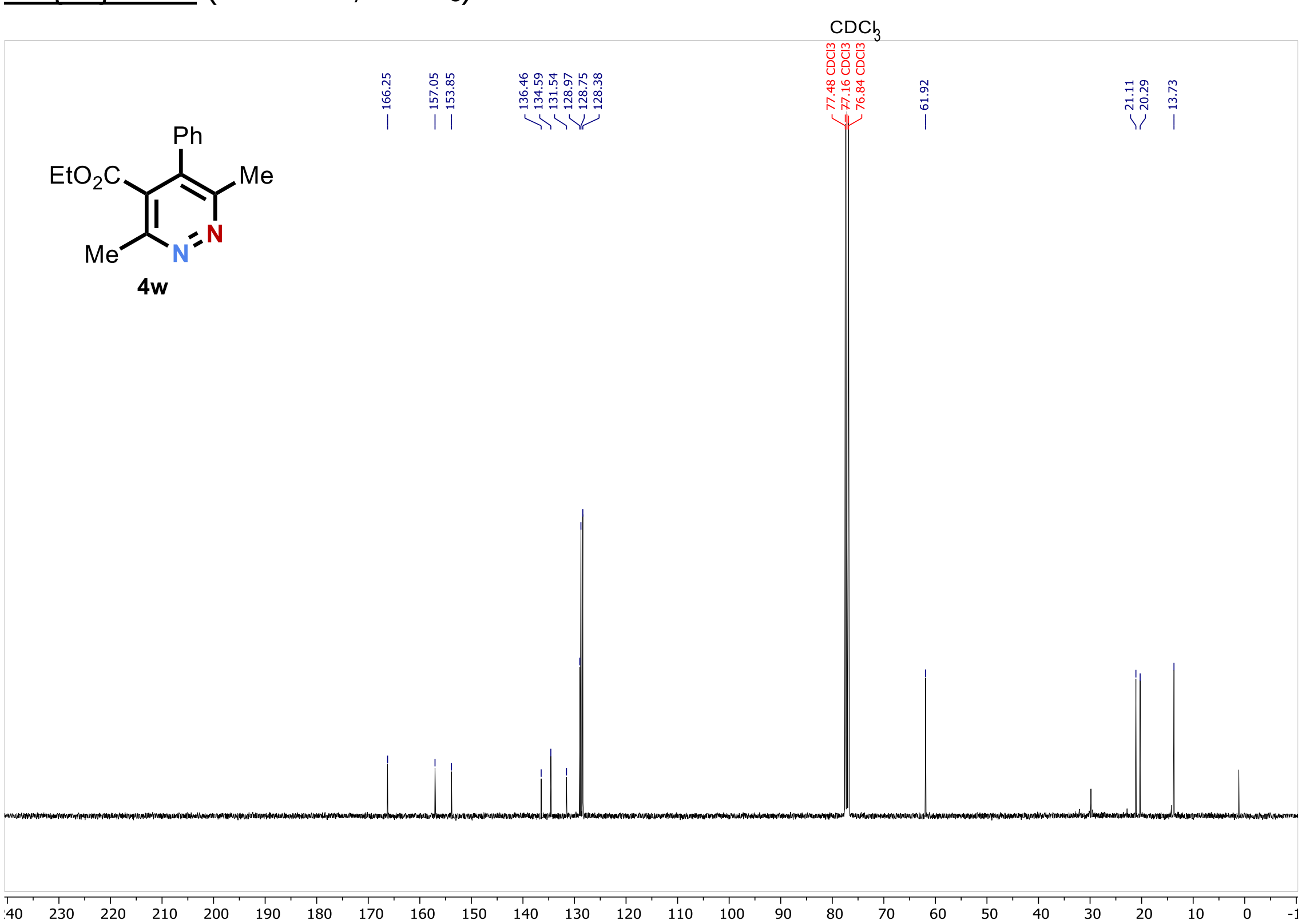

^{1}H NMR (400 MHz, $CDCl_3$)

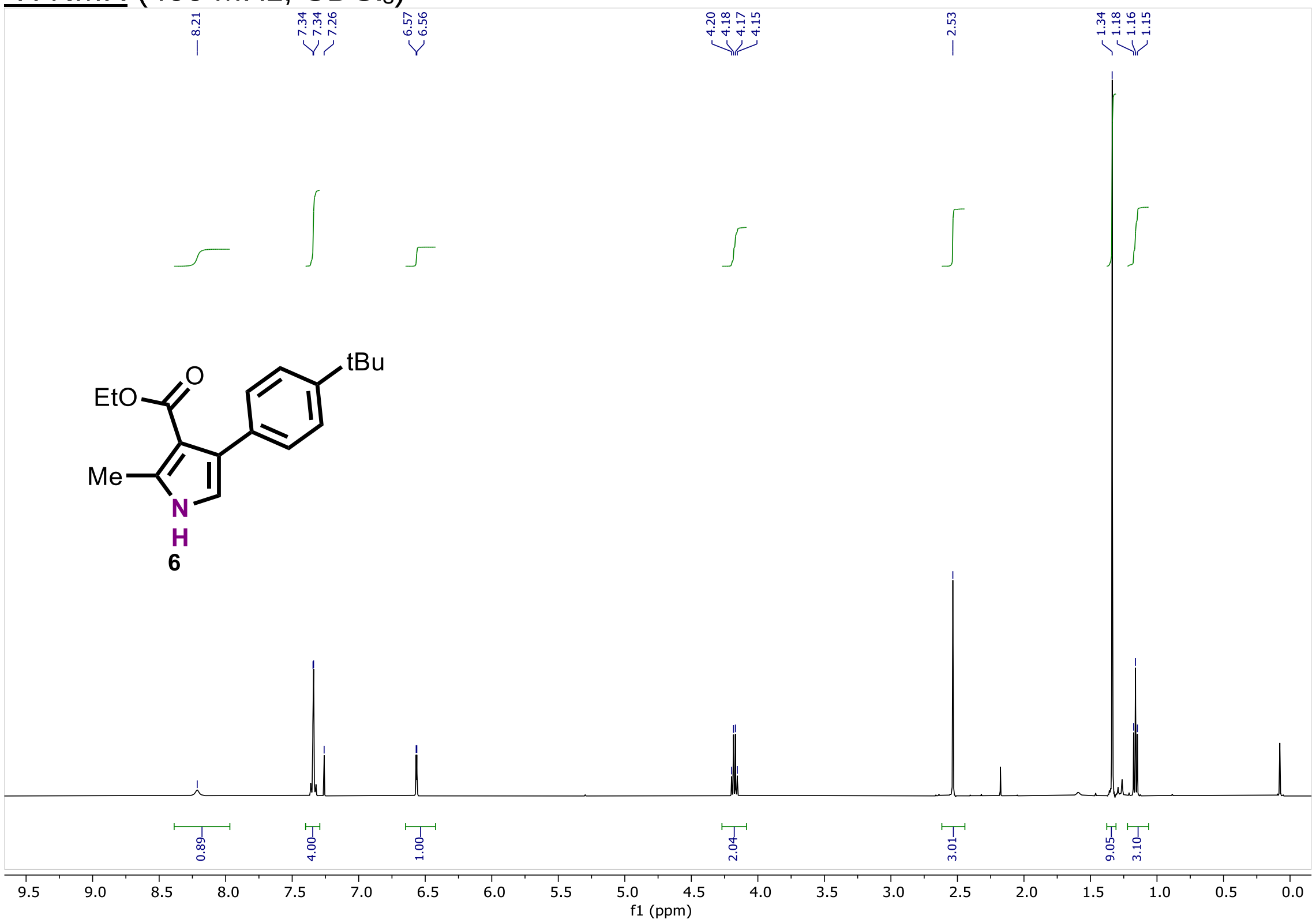

$^{13}C\{^{1}H\}$ NMR (101 MHz, $CDCl_3$)

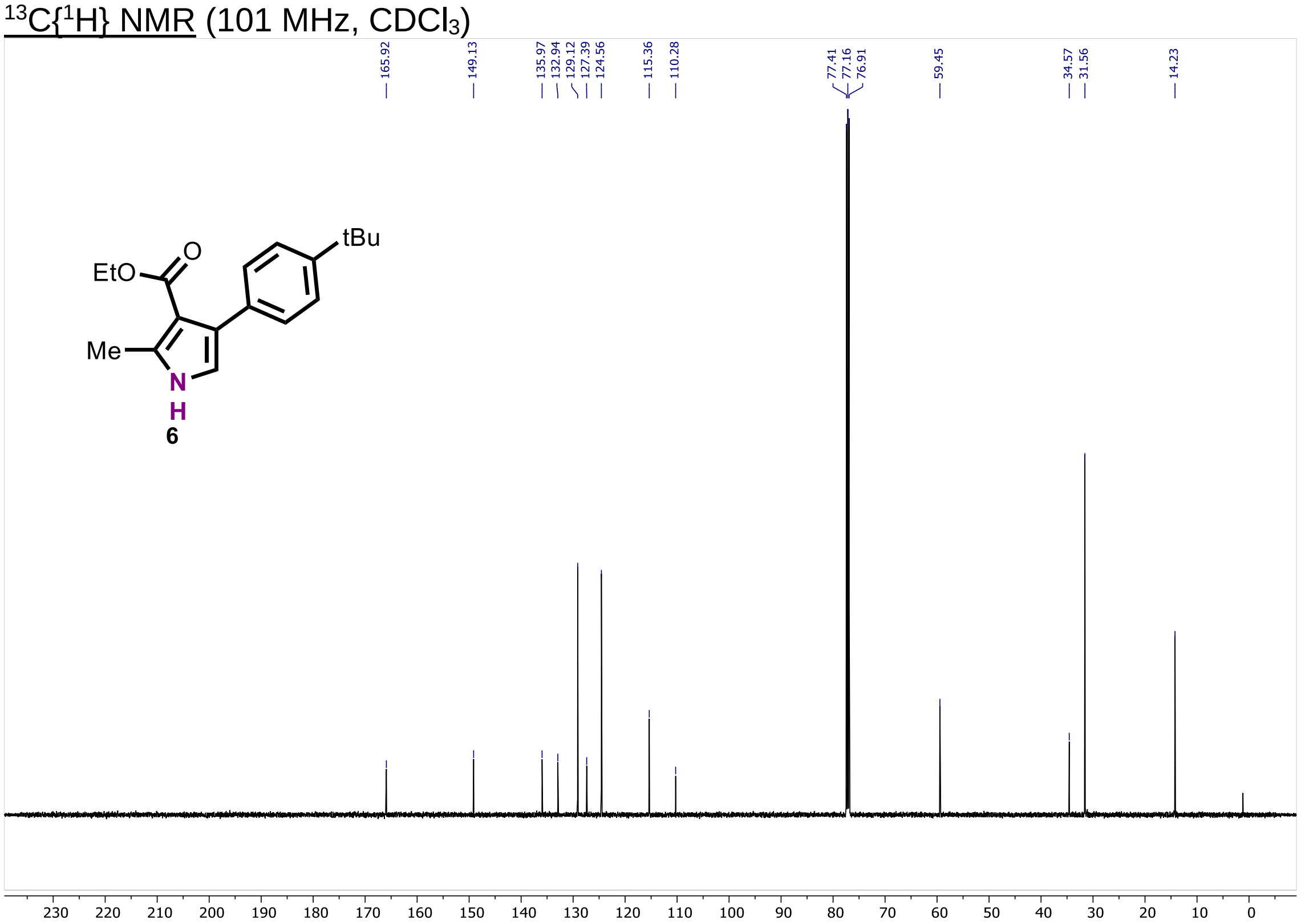

^{1}H NMR (500 MHz, $CDCl_3$)

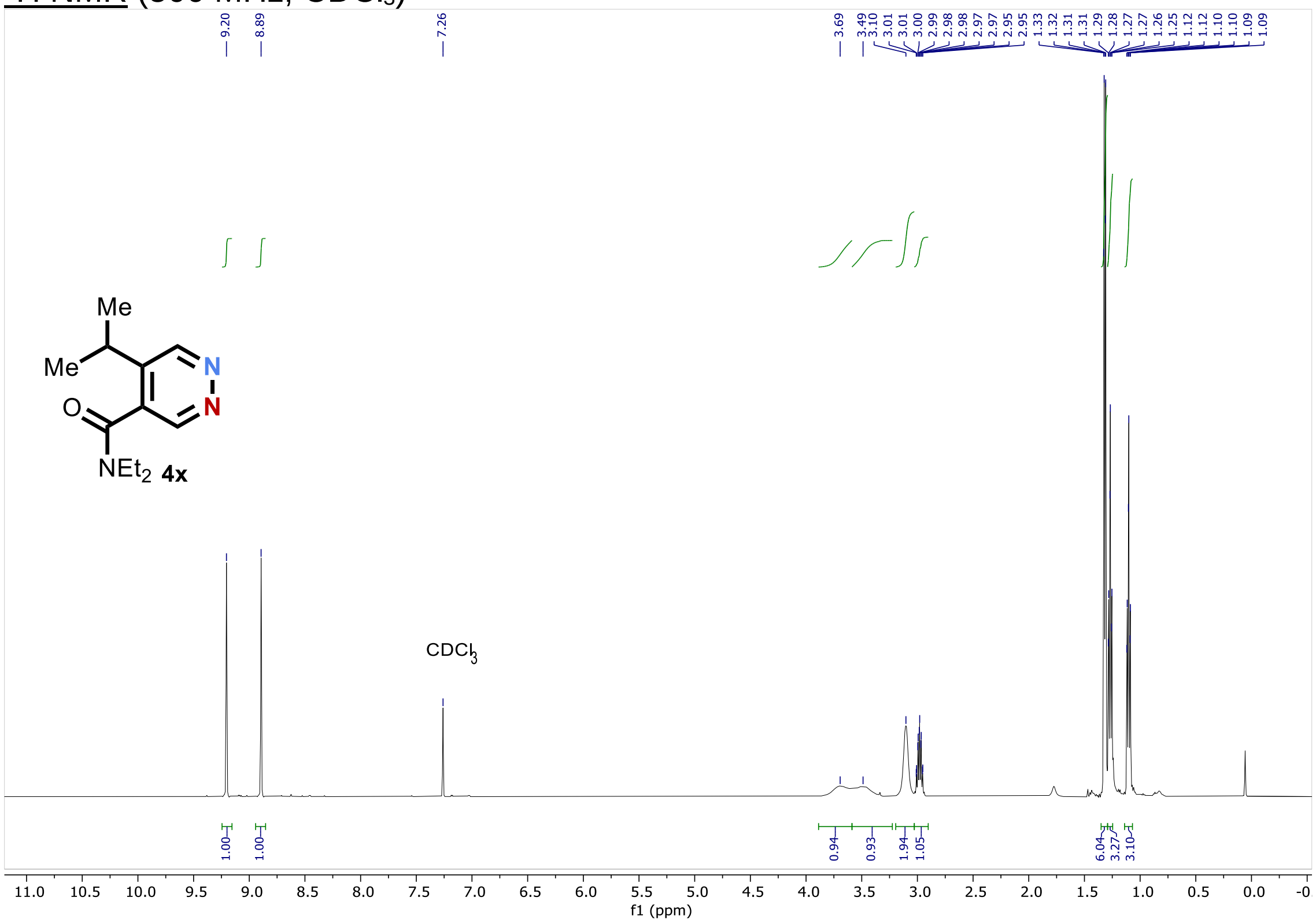

^{13}C{^{1}H} NMR (126 MHz, $CDCl_3$)

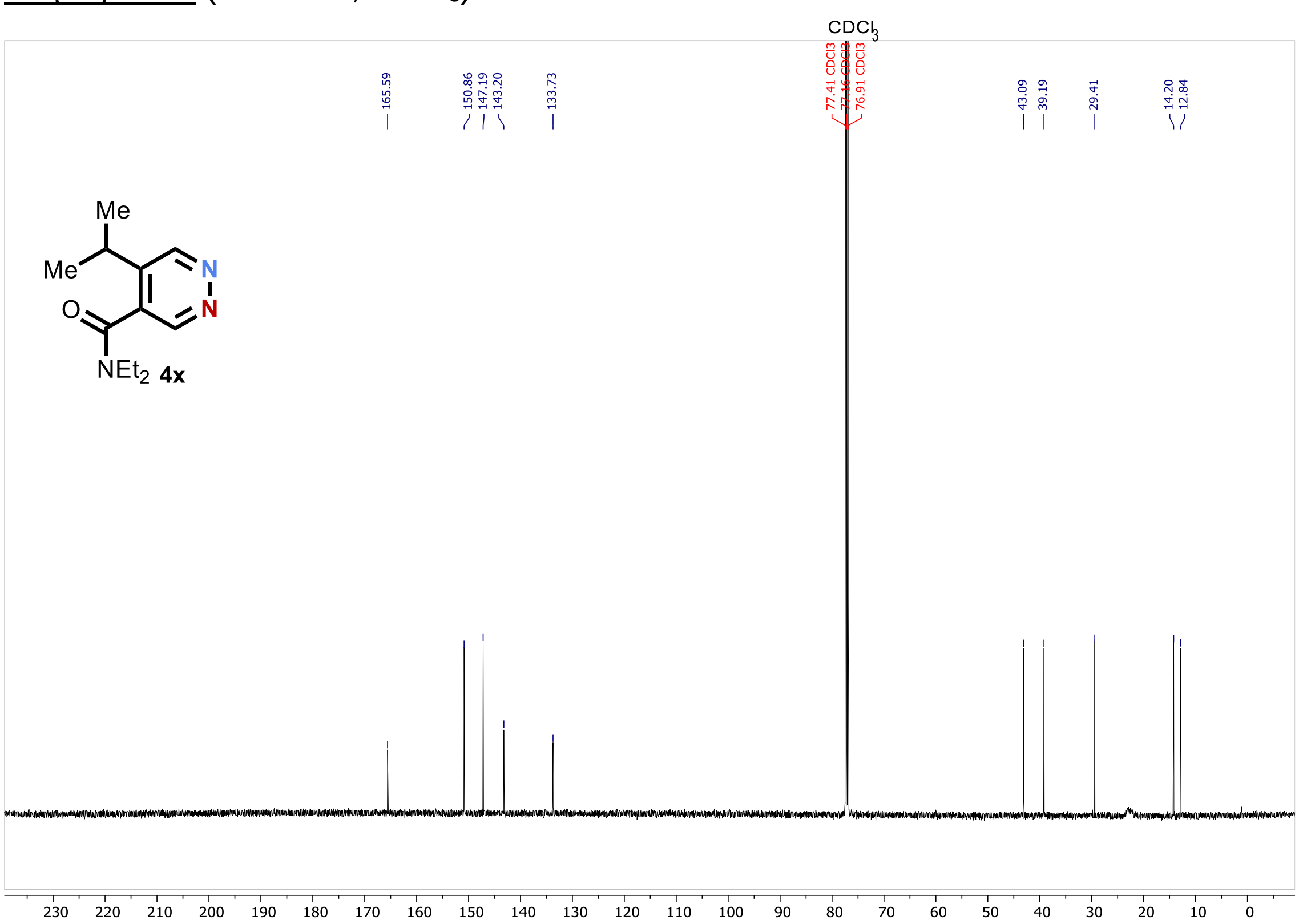

^{1}H NMR (500 MHz, $CDCl_3$)

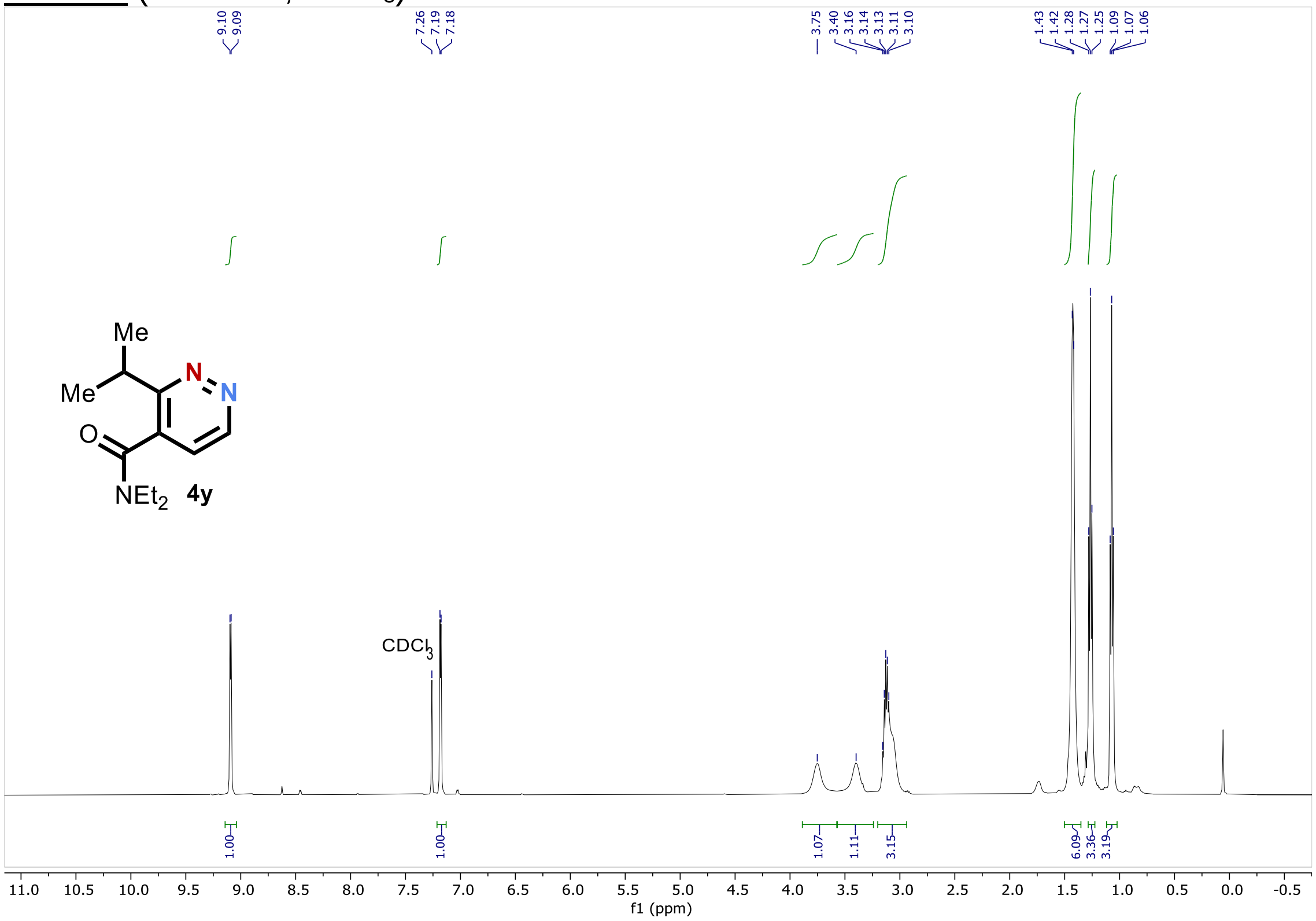

^{13}C{^{1}H} NMR (126 MHz, $CDCl_3$)

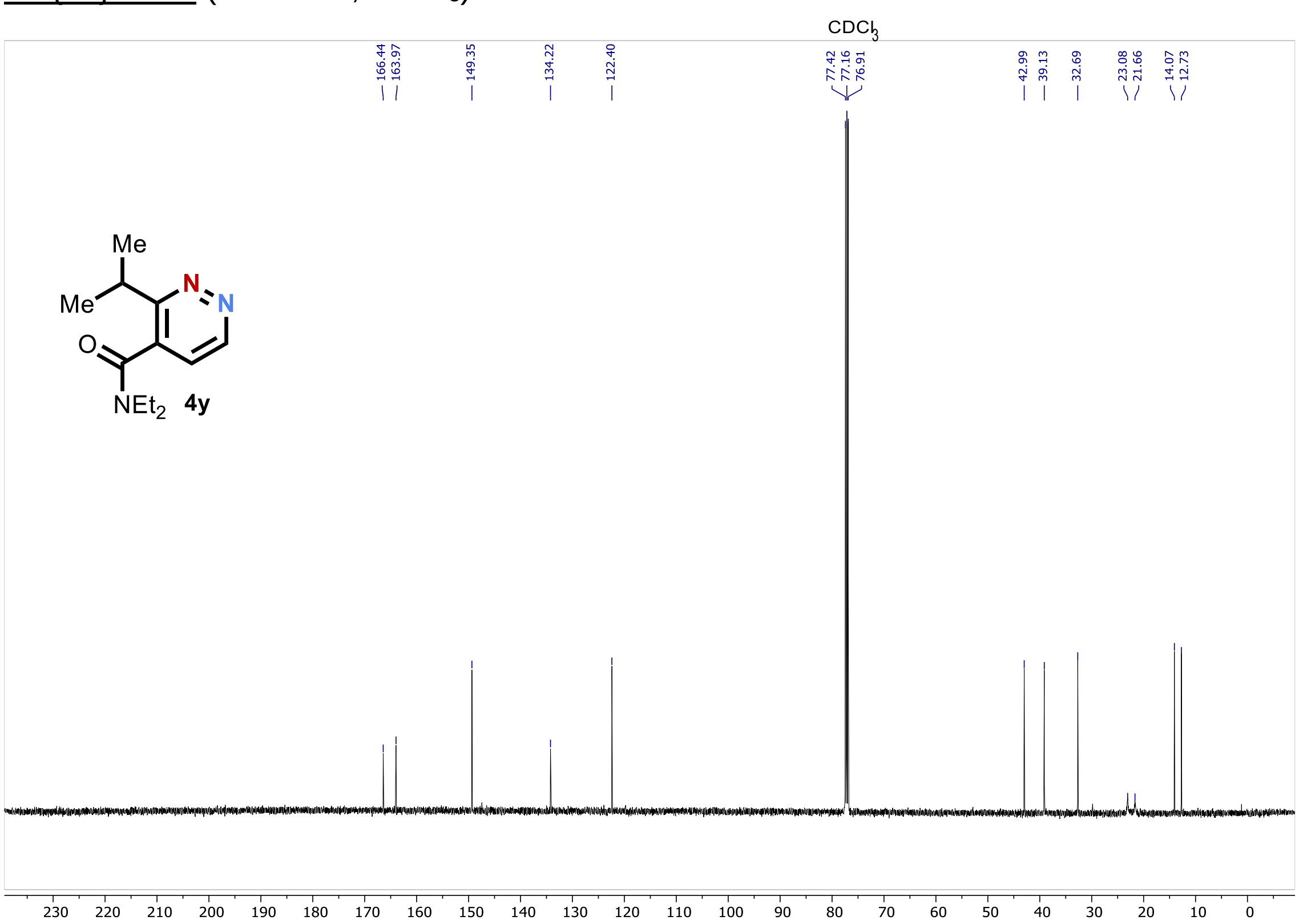

^1H NMR (400 MHz, $CDCl_3$)

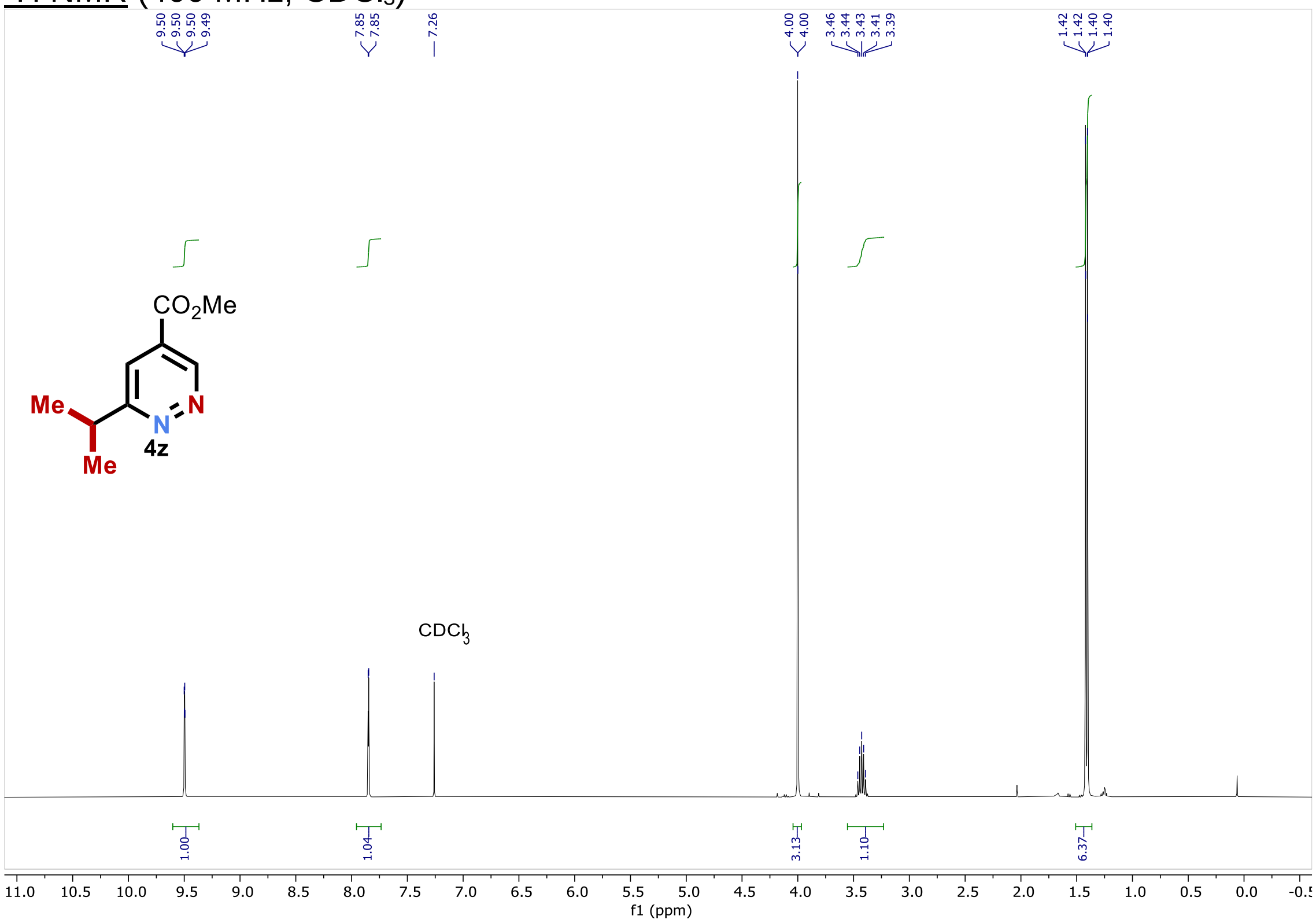

$^{13}C\{^1H\}$ NMR (101 MHz, $CDCl_3$)

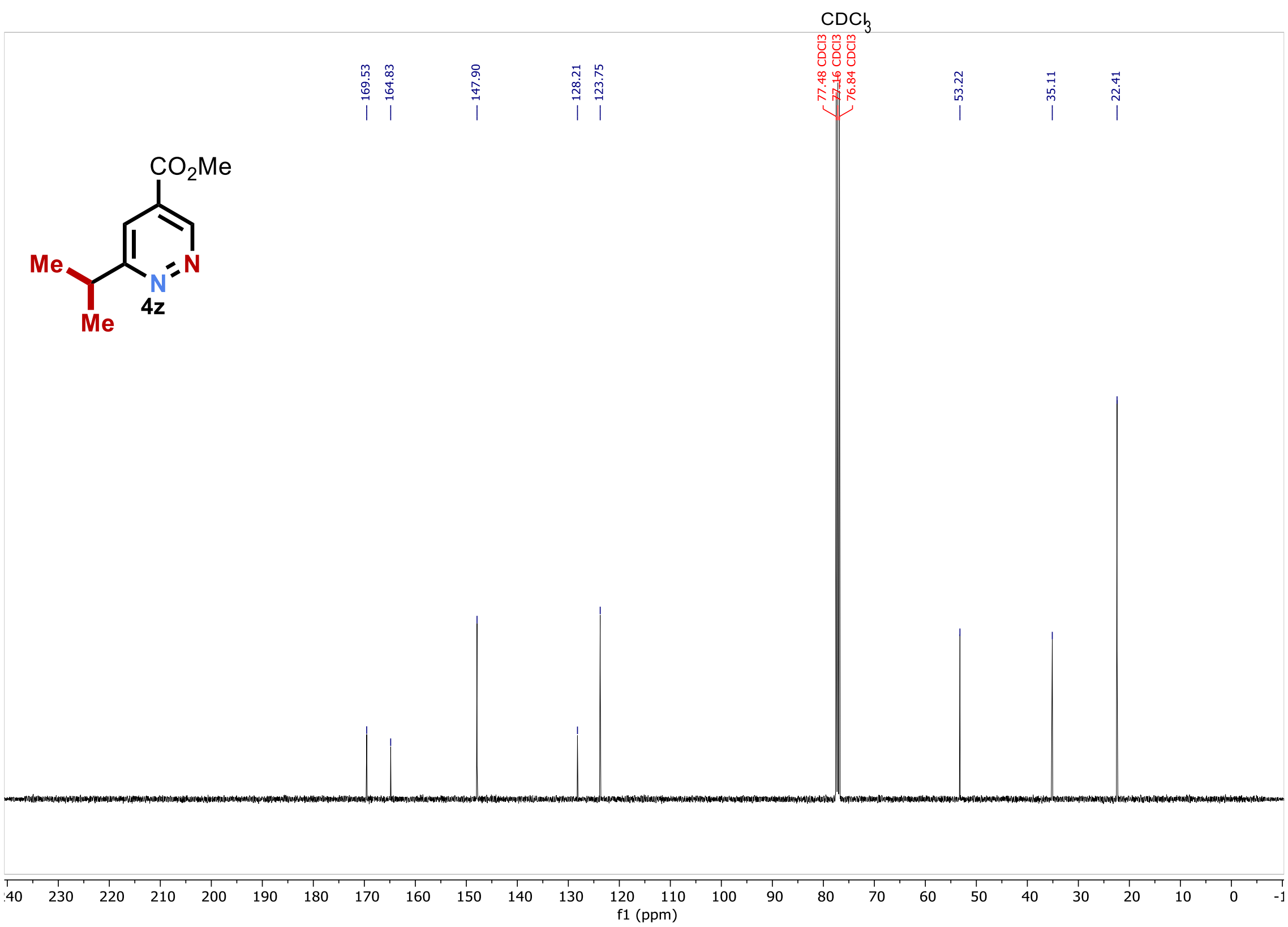

^{1}H NMR (600 MHz, $CDCl_3$)

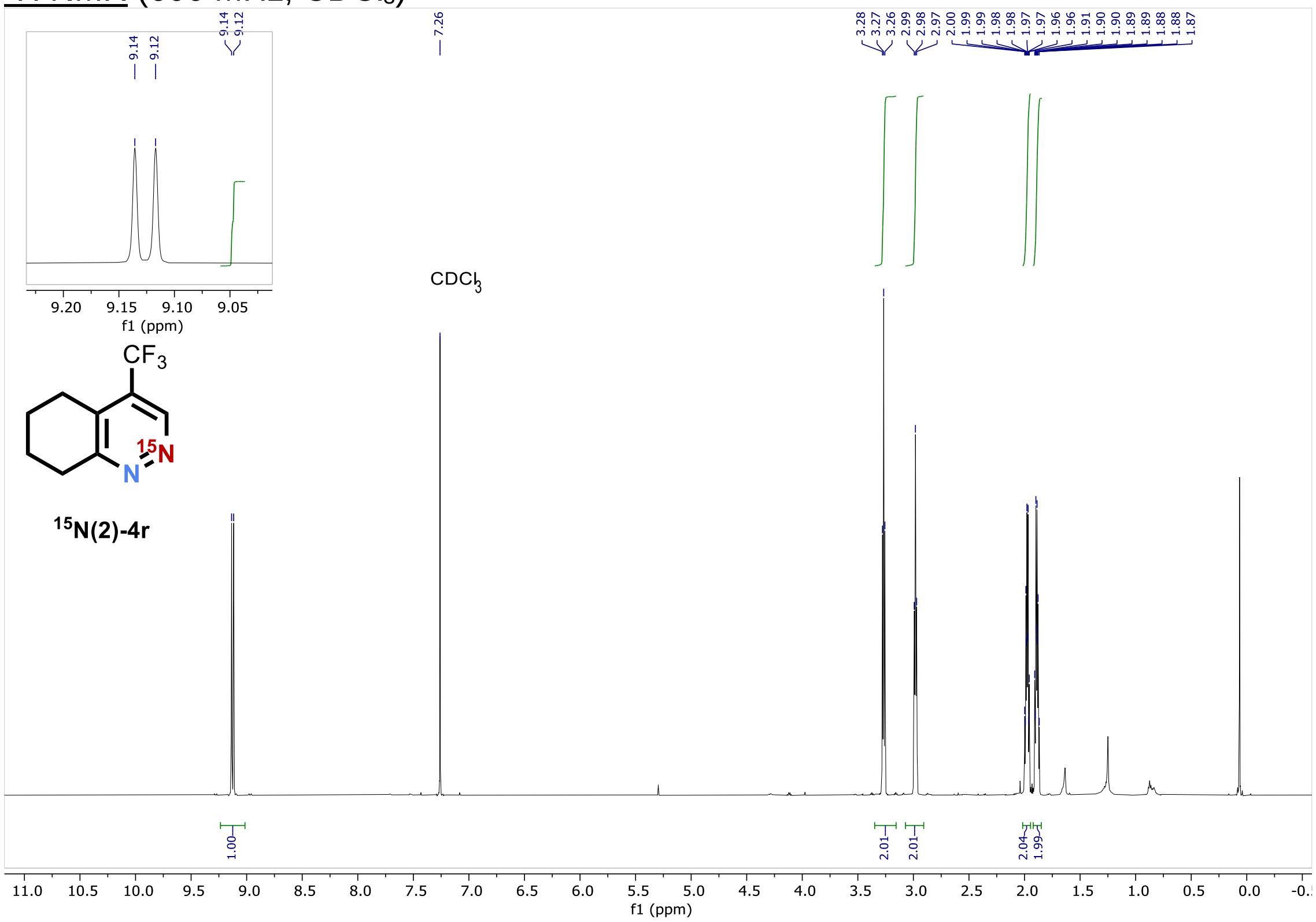

^{19}F NMR (565 MHz, $CDCl_3$) δ -63.1.

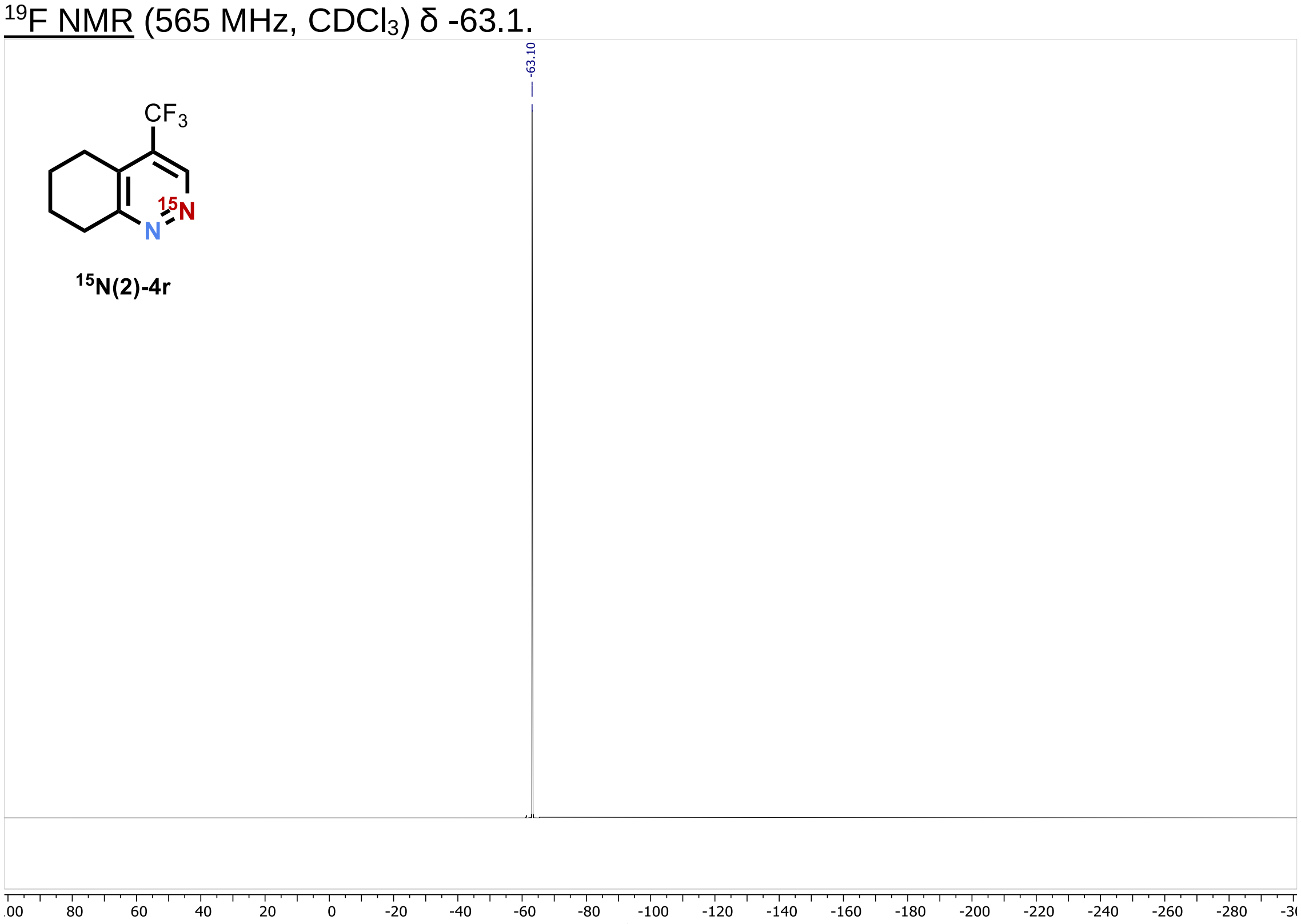

$^{13}C\{^{1}H\}$ NMR (151 MHz, $CDCl_3$)

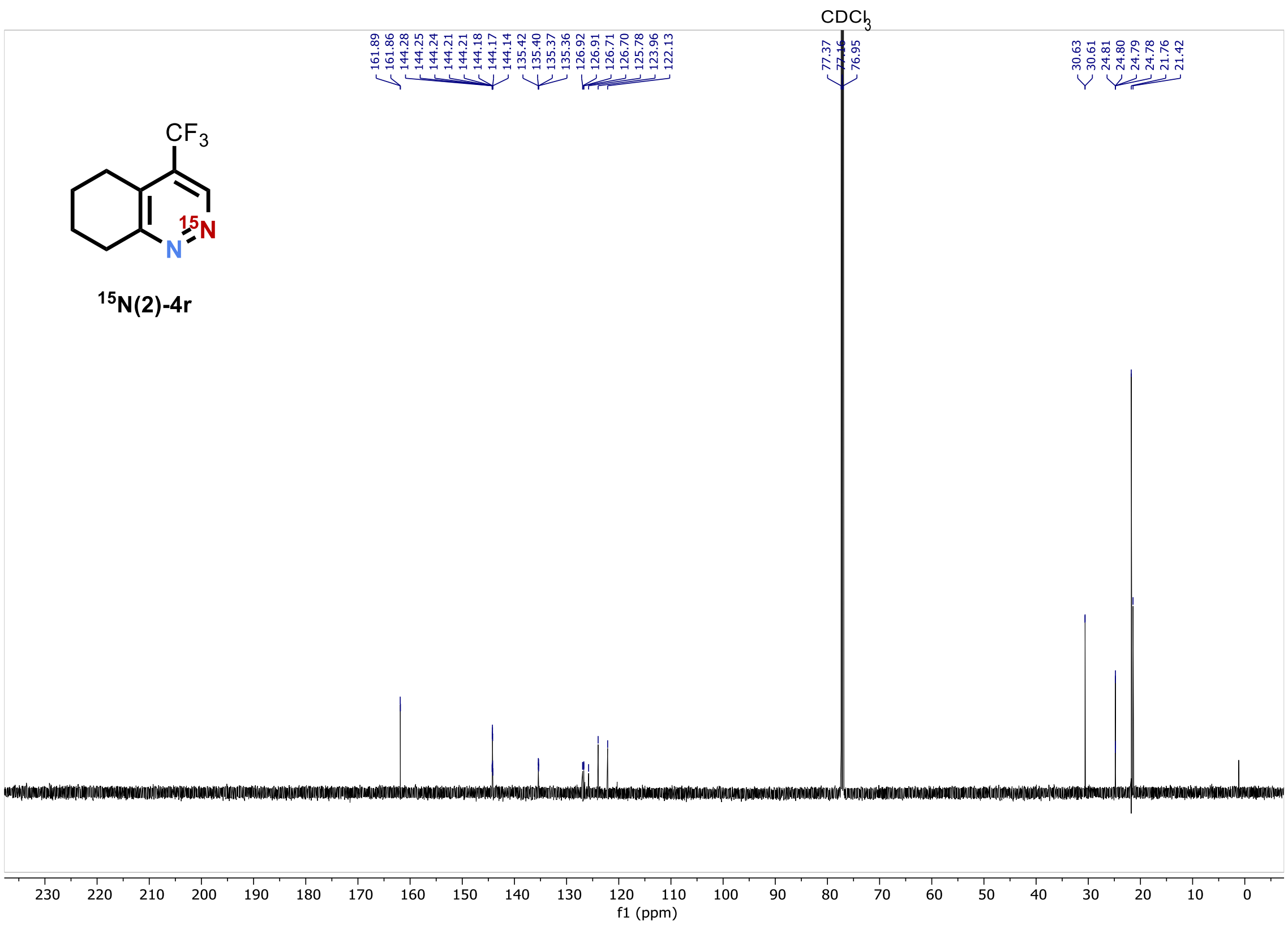

^{15}N-^{1}H HMBC ($CDCl_3$)

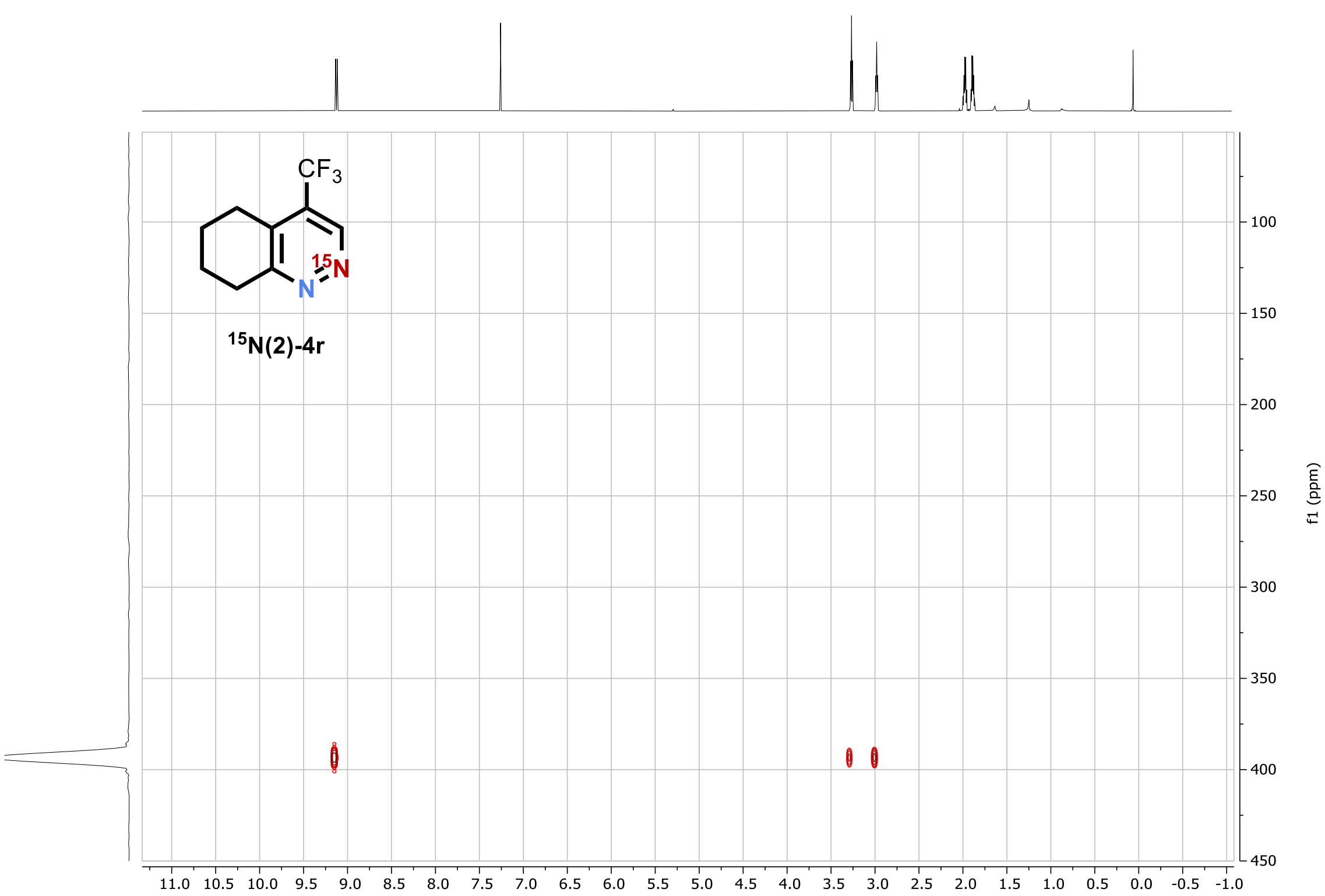

^{1}H NMR (600 MHz, $CDCl_3$)

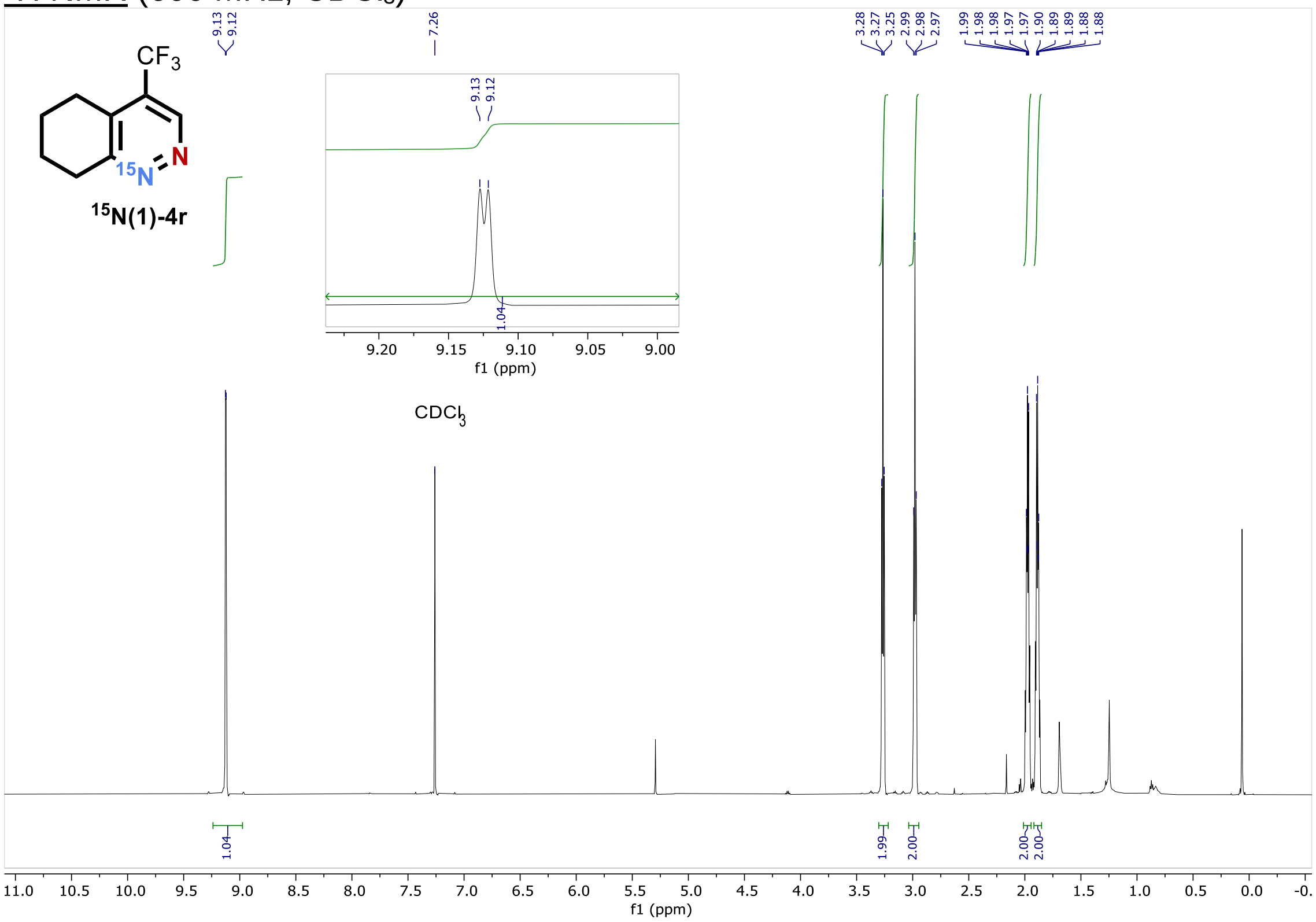

^{19}F NMR (565 MHz, $CDCl_3$) δ -63.1.

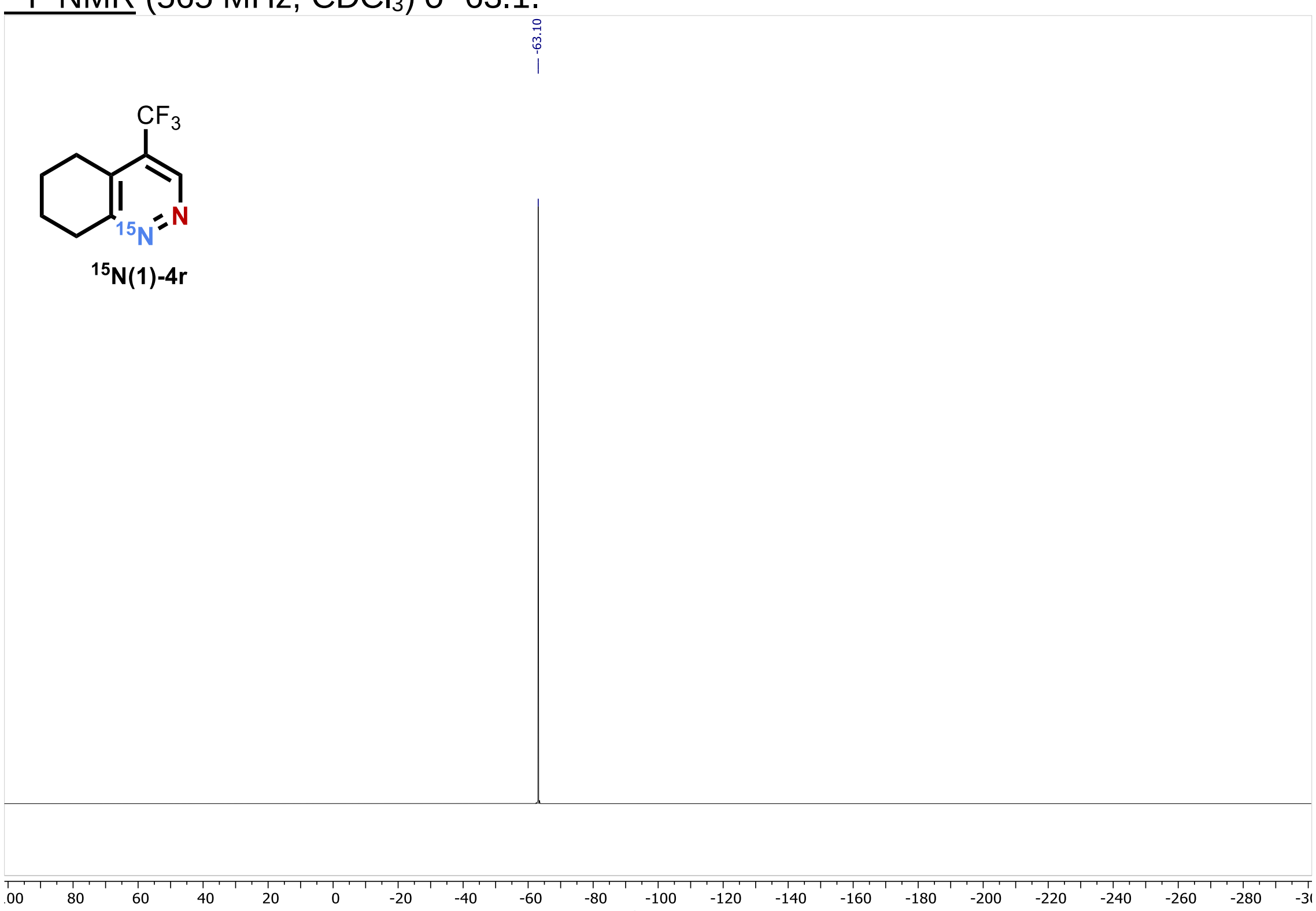

$^{13}C\{^{1}H\}$ NMR (151 MHz, $CDCl_3$)

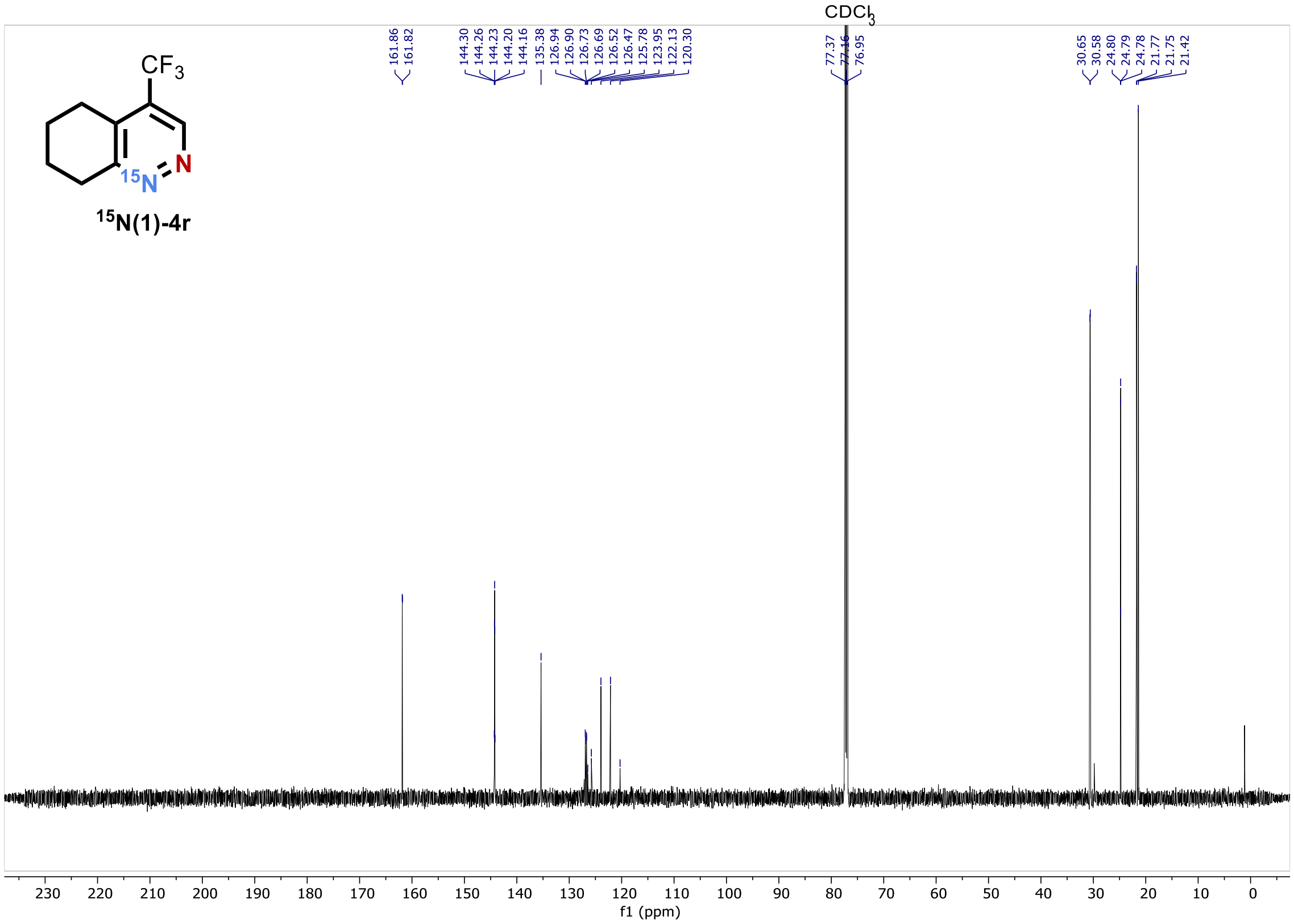

^{15}N-^{1}H HMBC ($CDCl_3$)

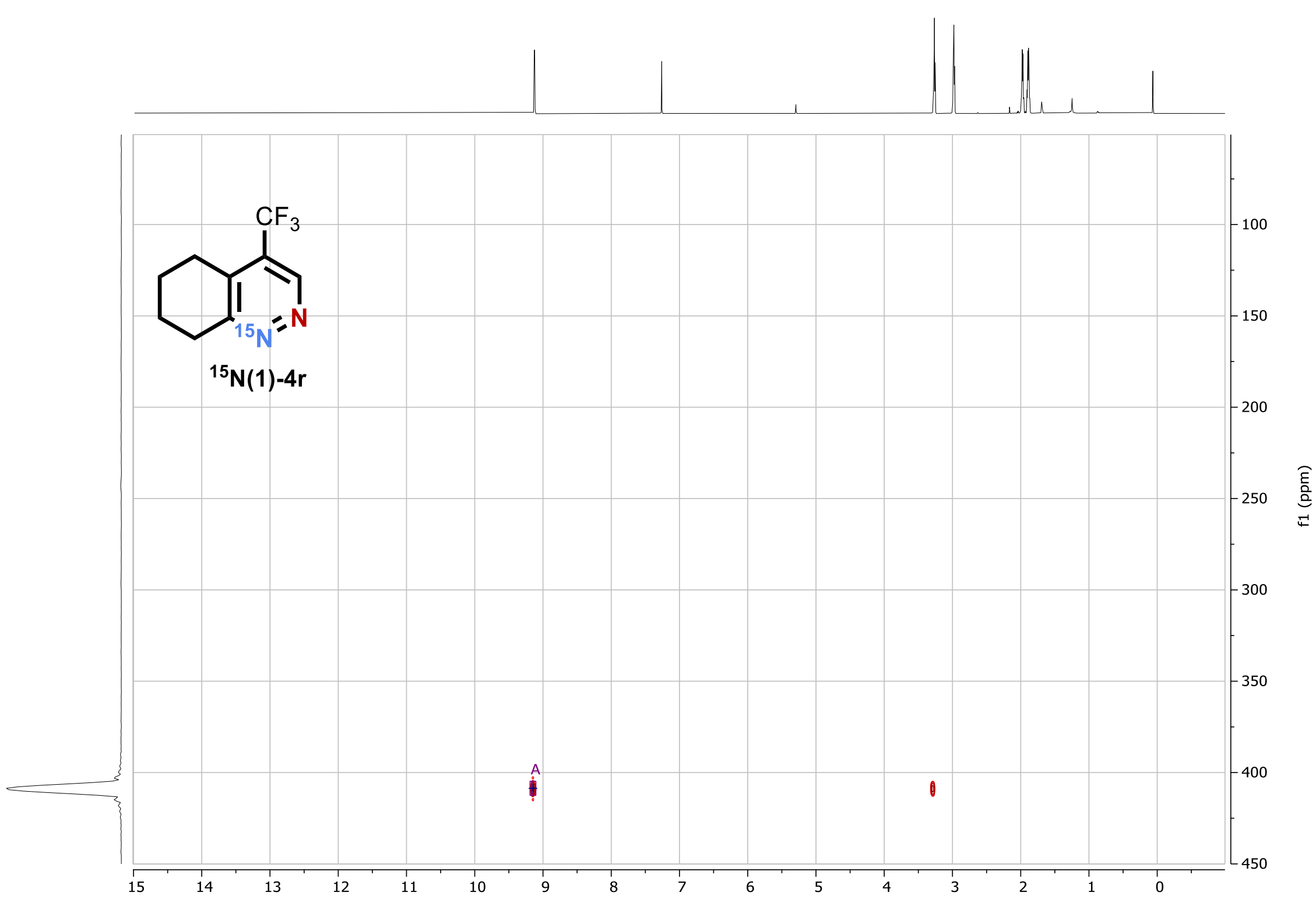

^{1}H NMR (500 MHz, CD_3CN)

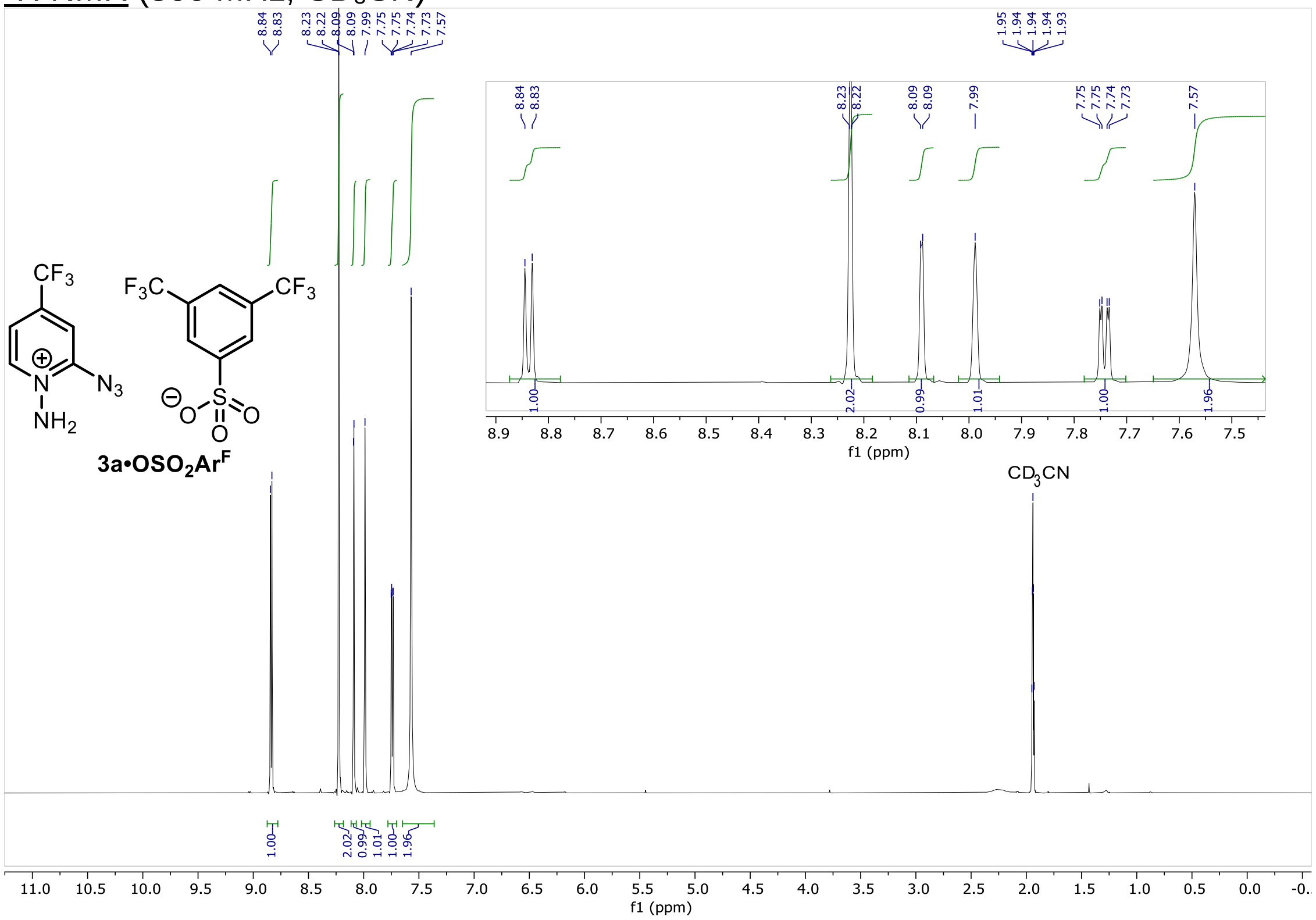

^{19}F NMR (470 MHz, CD_3CN)

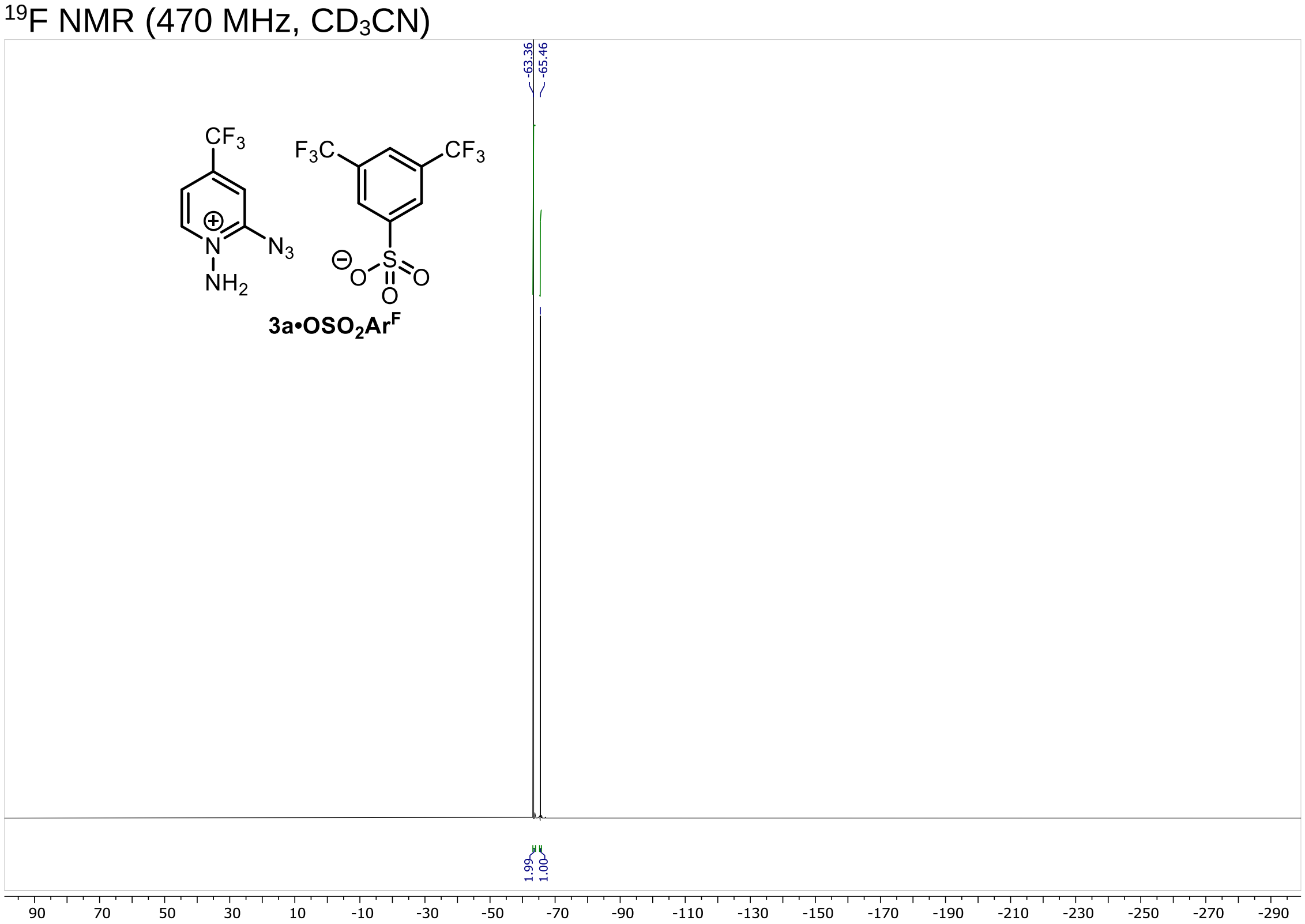

$^{13}C\{^1H\}$ NMR (126 MHz, CD_3CN)

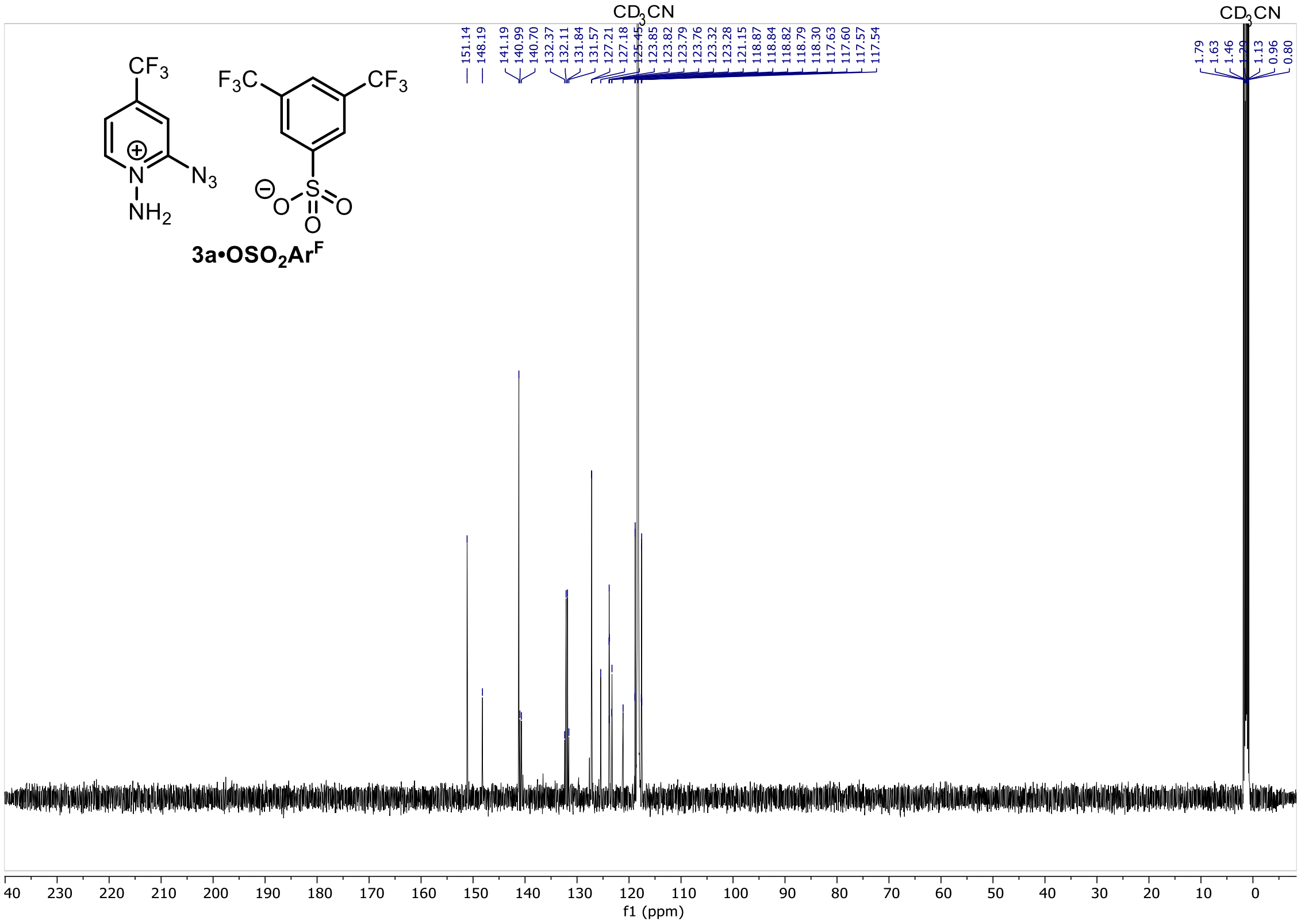

^{1}H NMR (500 MHz, CD_3CN)

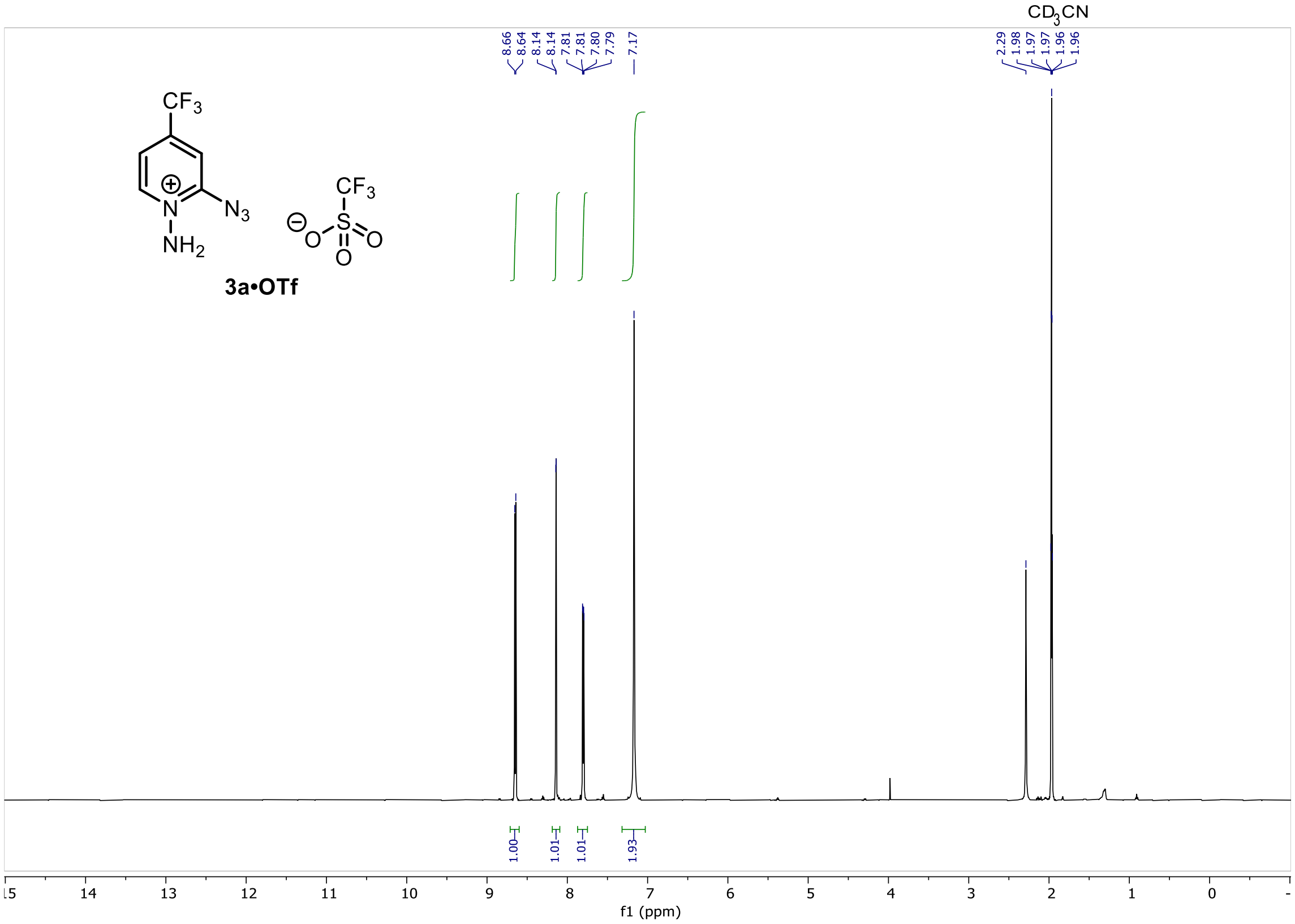

^{19}F NMR (470 MHz, CD_3CN)

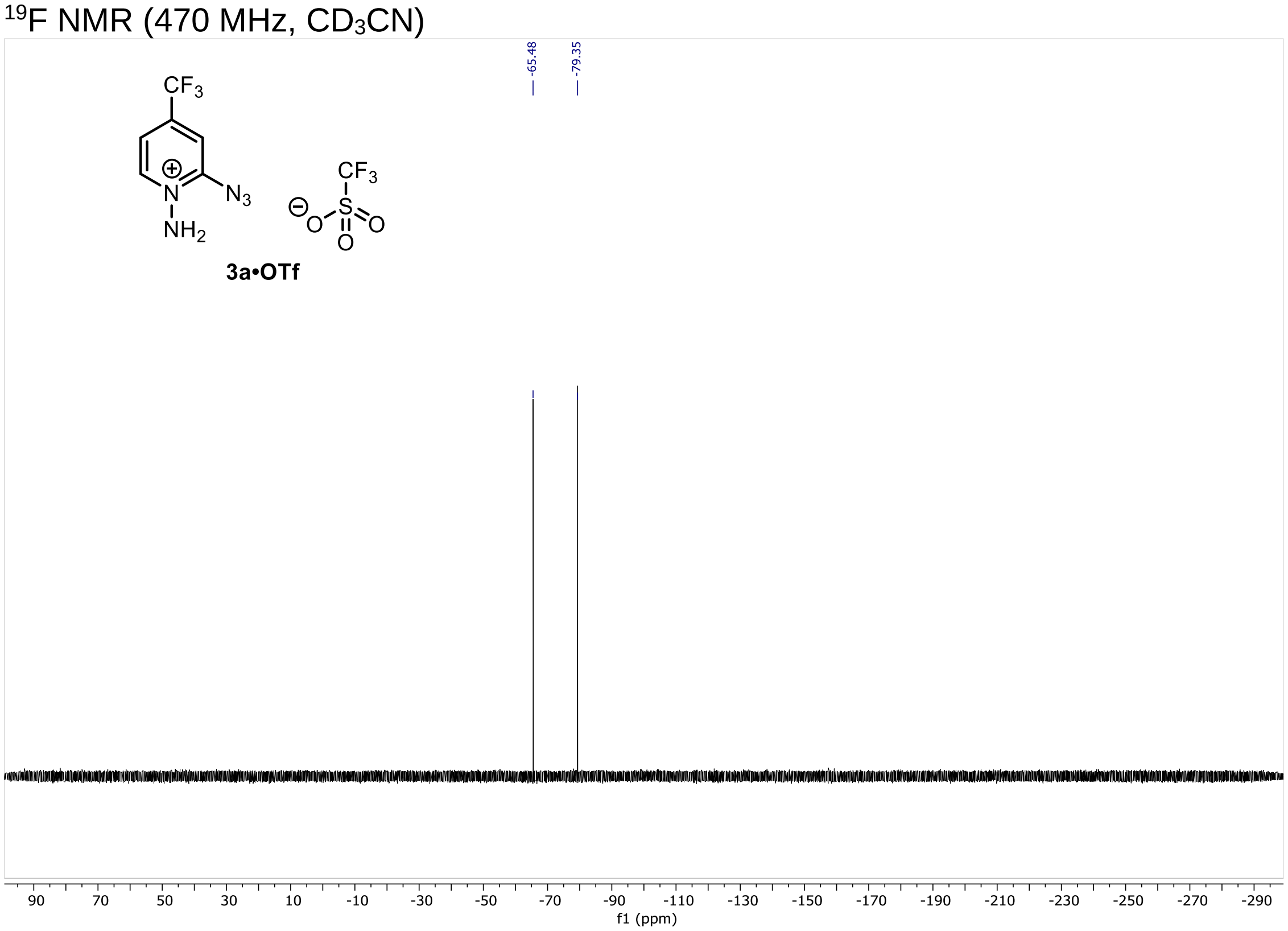

$^{13}C\{^{1}H\}$ NMR (126 MHz, CD_3CN)

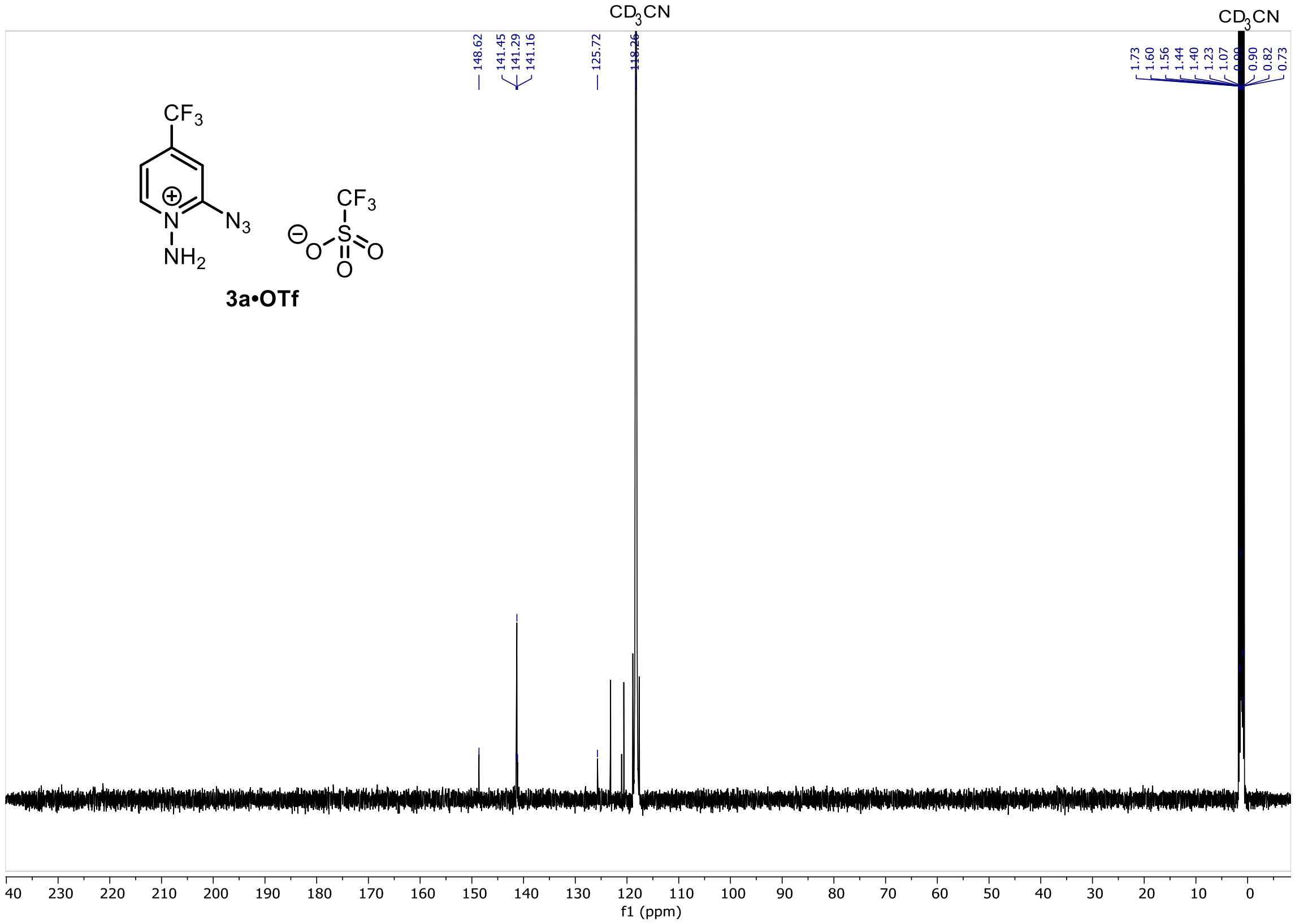

^{1}H NMR (500 MHz, CD_3CN)

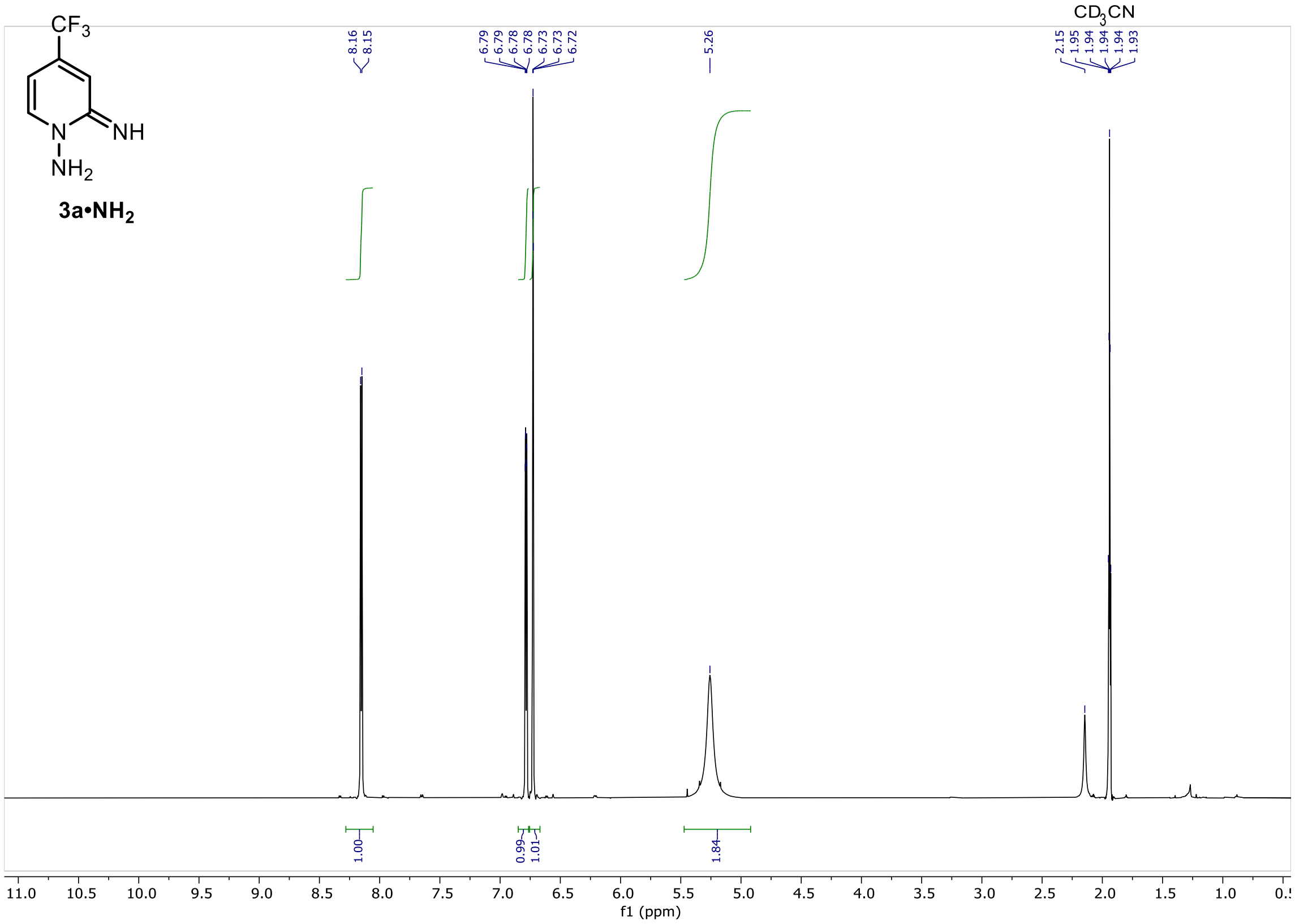

^{19}F NMR (470 MHz, CD_3CN)

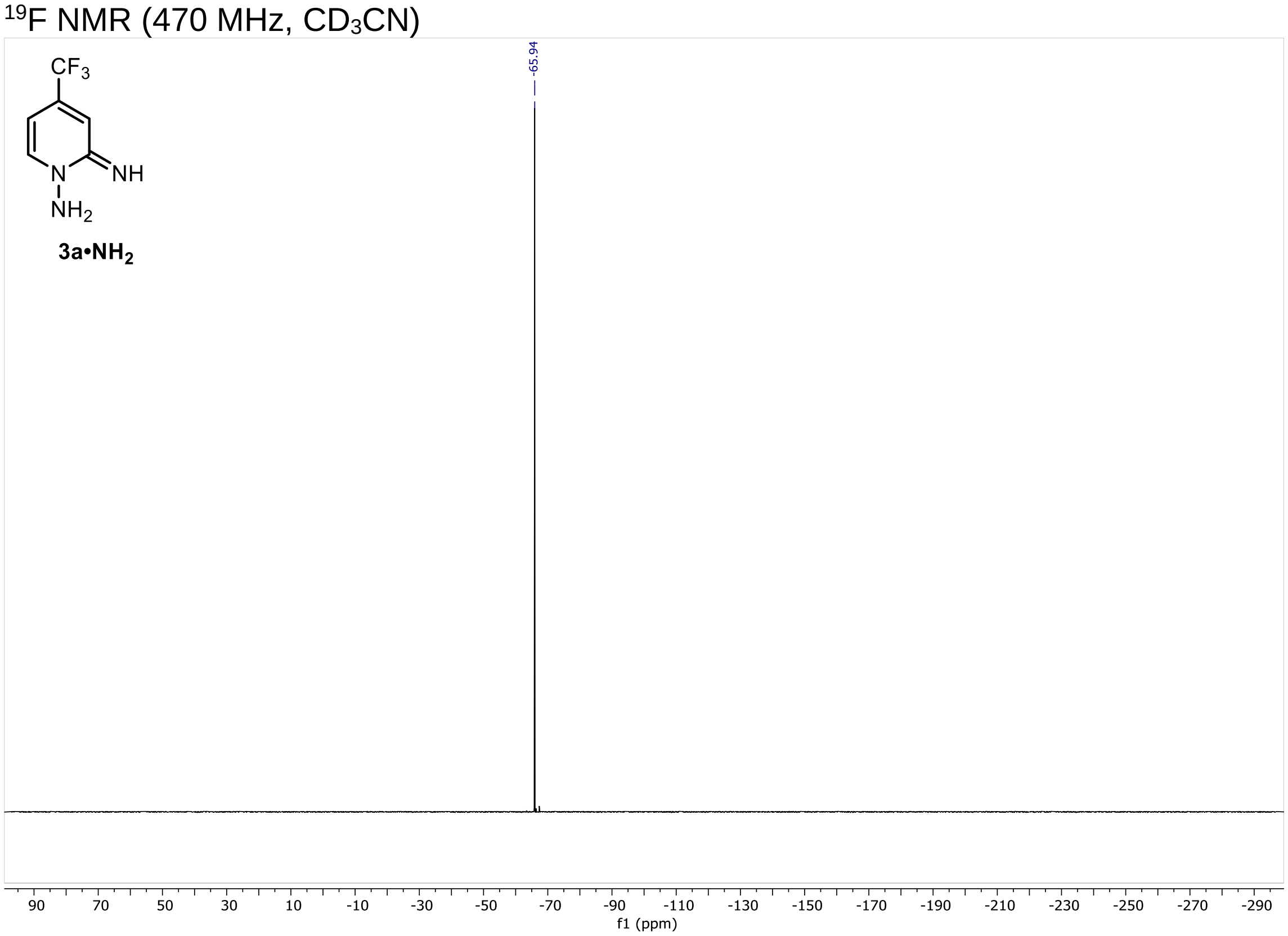

$^{13}C\{^{1}H\}$ NMR (126 MHz, CD_3CN)

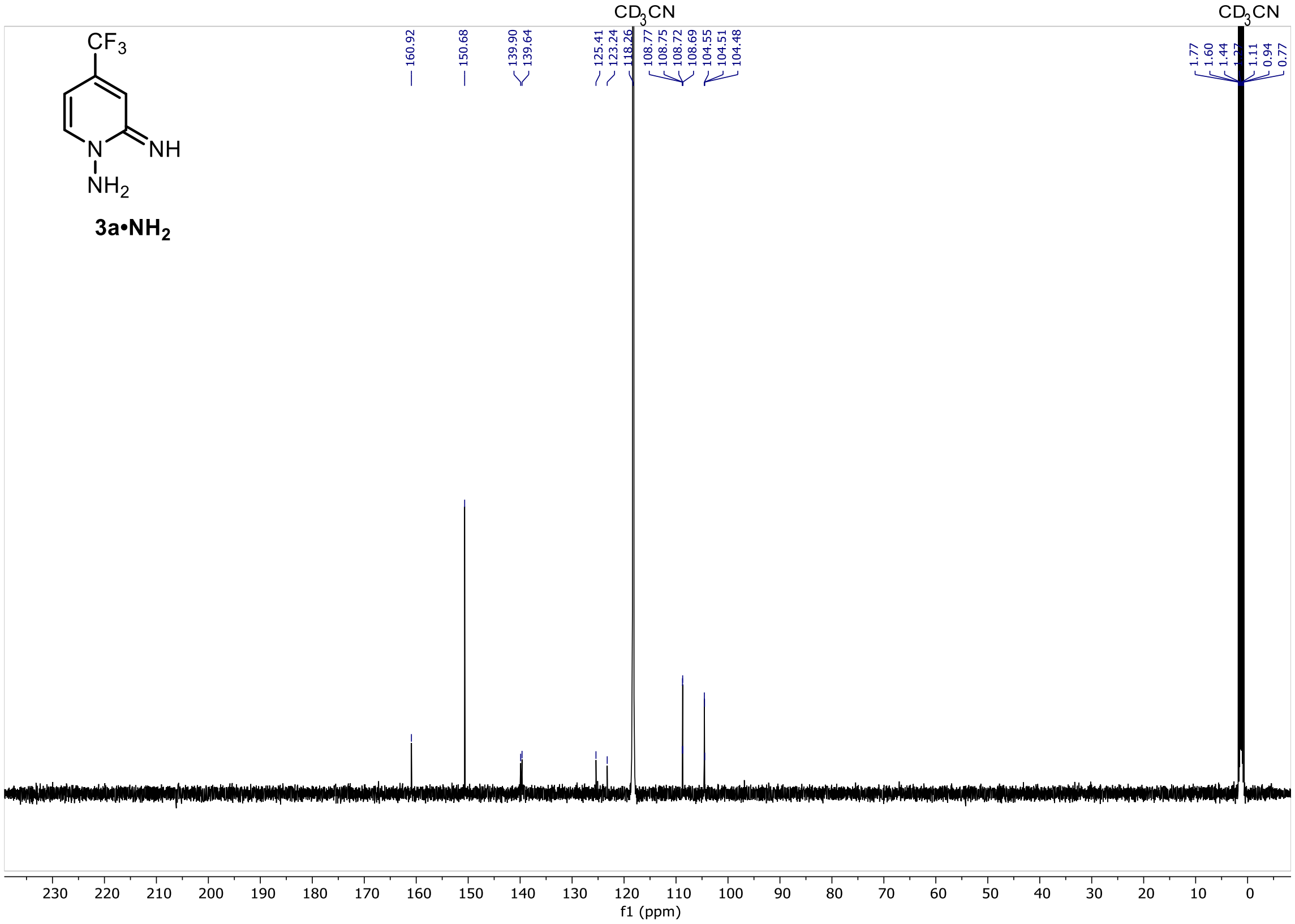

^{1}H NMR (400 MHz, $CDCl_3$)

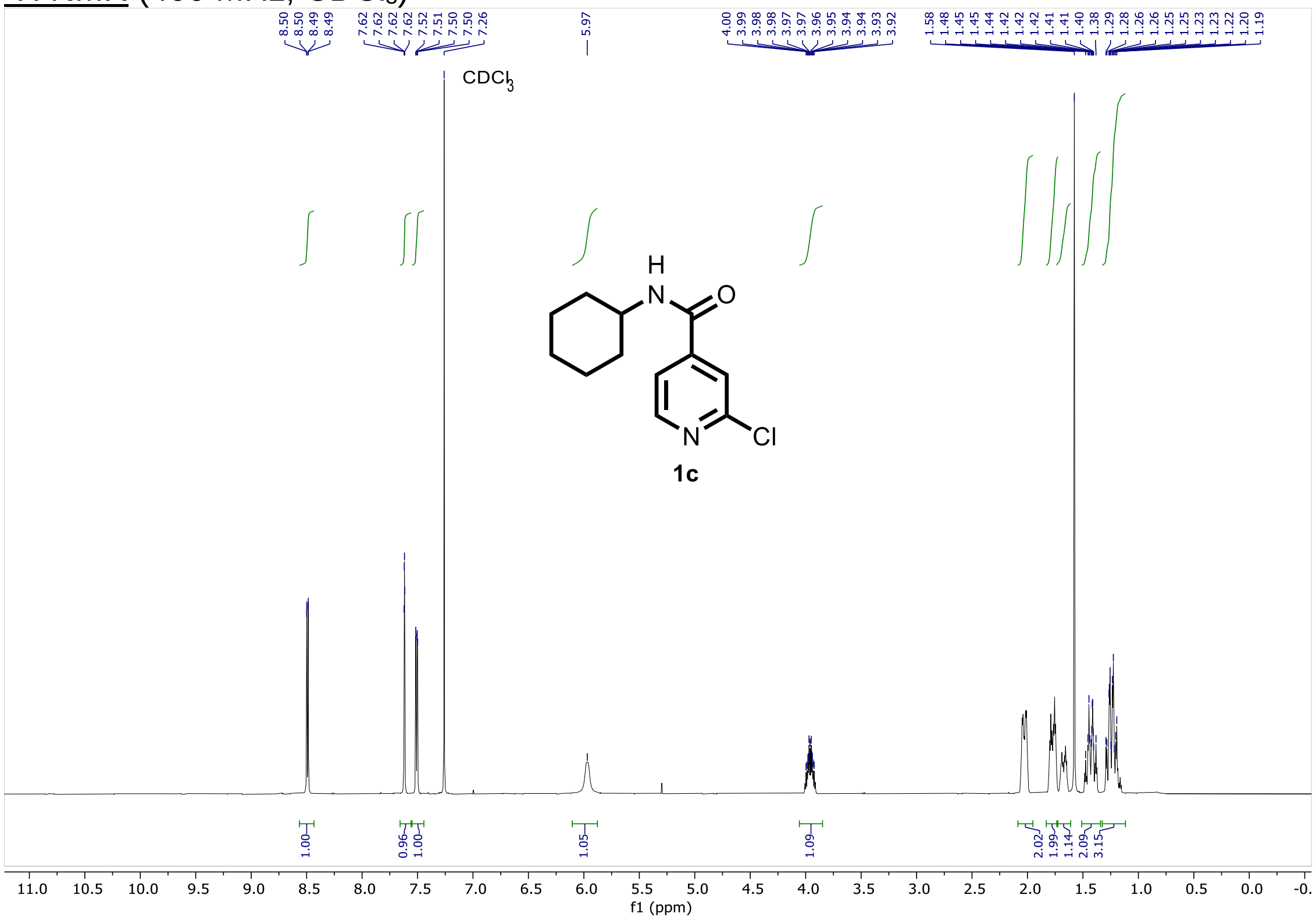

$^{13}C\{^{1}H\}$ NMR (101 MHz, $CDCl_3$)

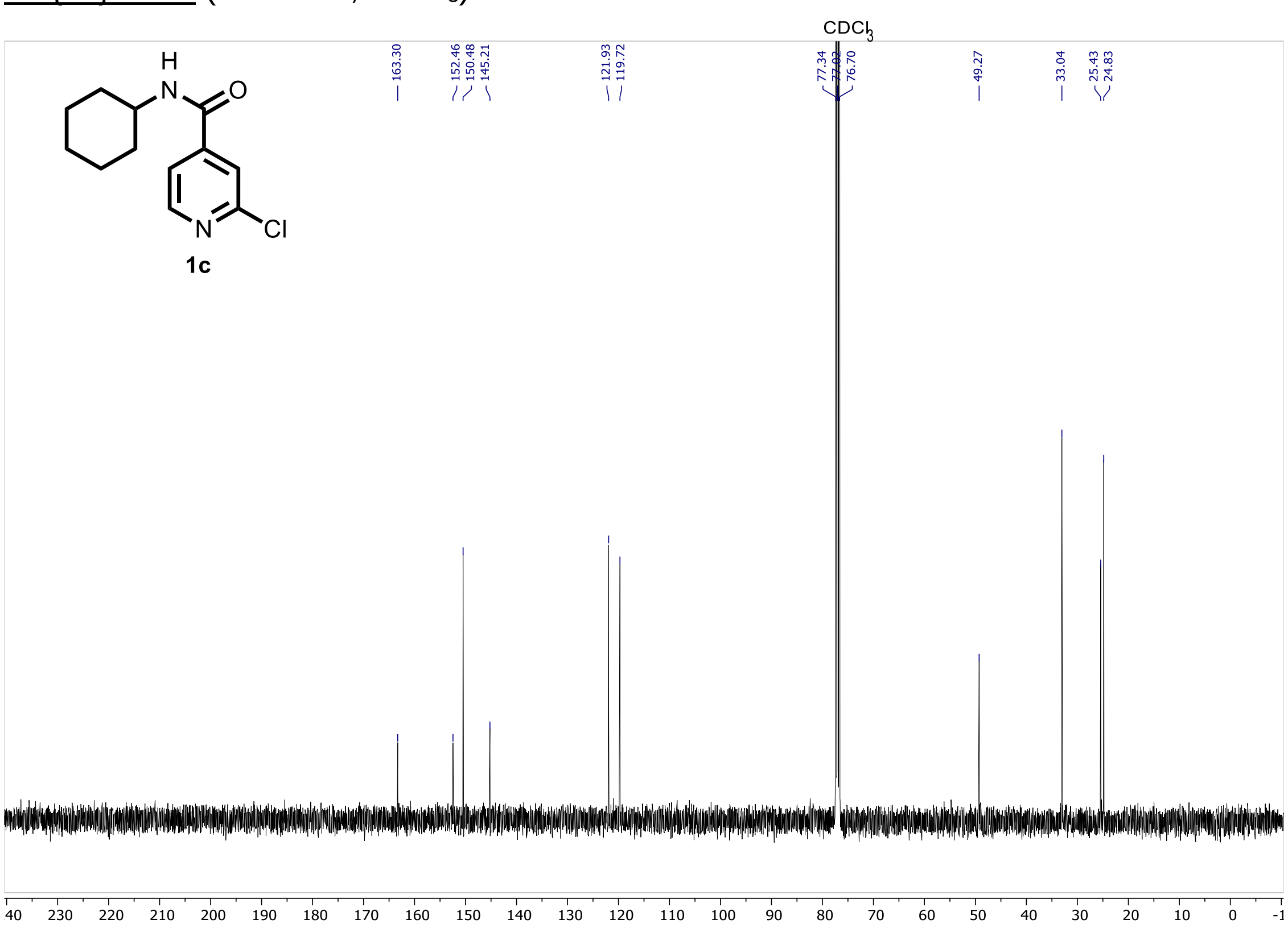

^{1}H NMR (400 MHz, $CDCl_3$)

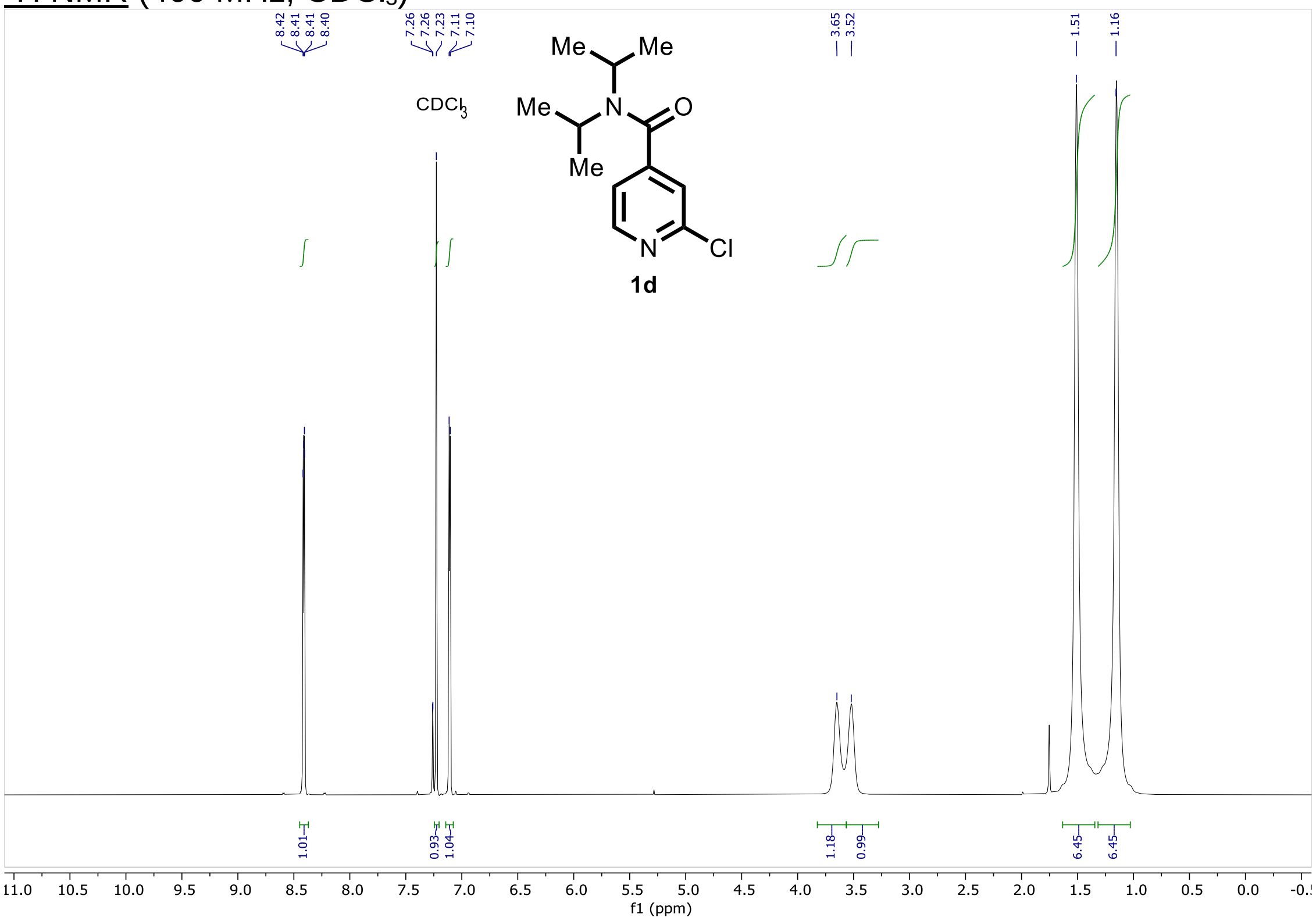

^{13}C{^{1}H} NMR (101 MHz, $CDCl_3$)

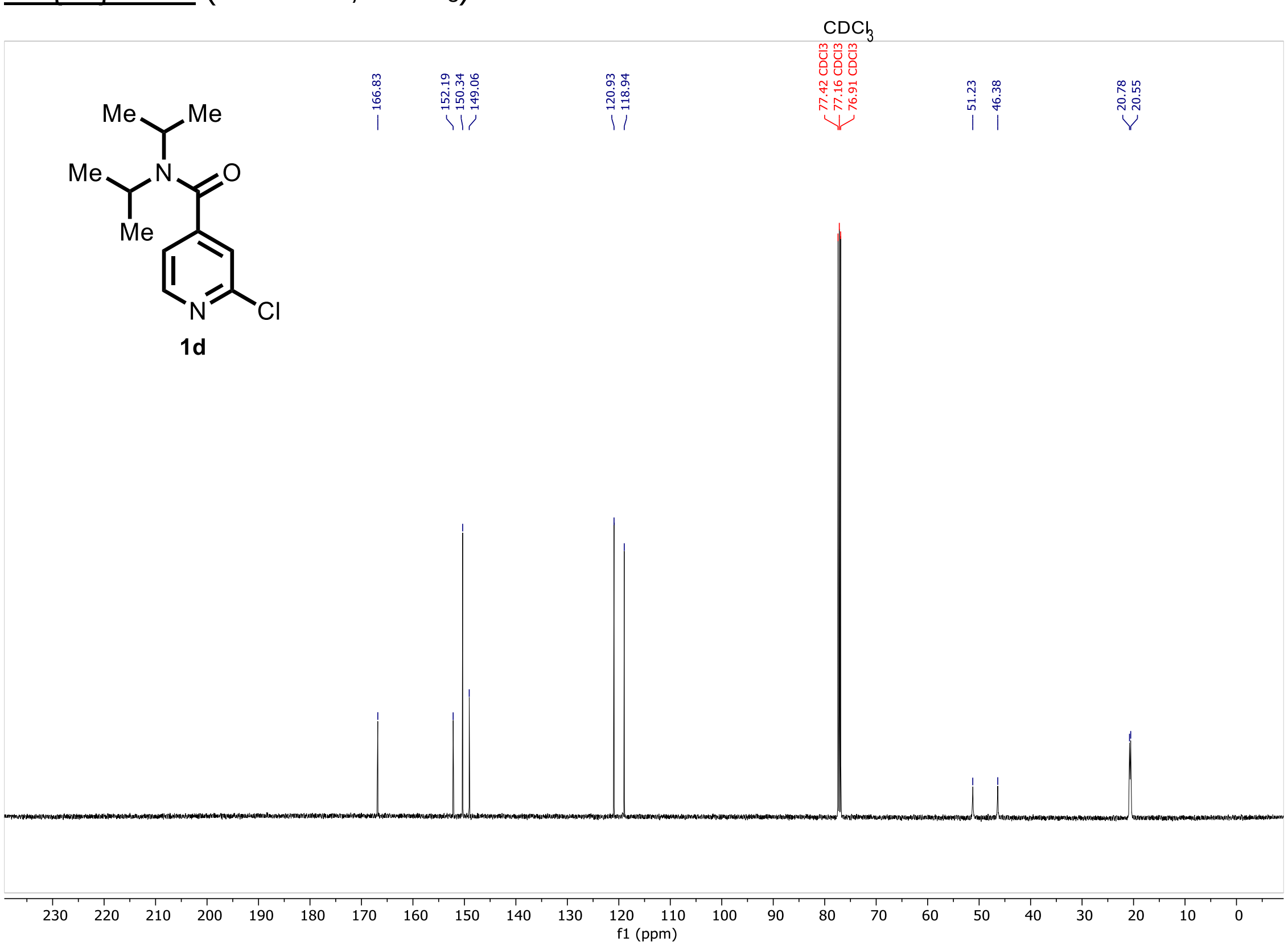

^{1}H NMR (400 MHz, $CDCl_3$)

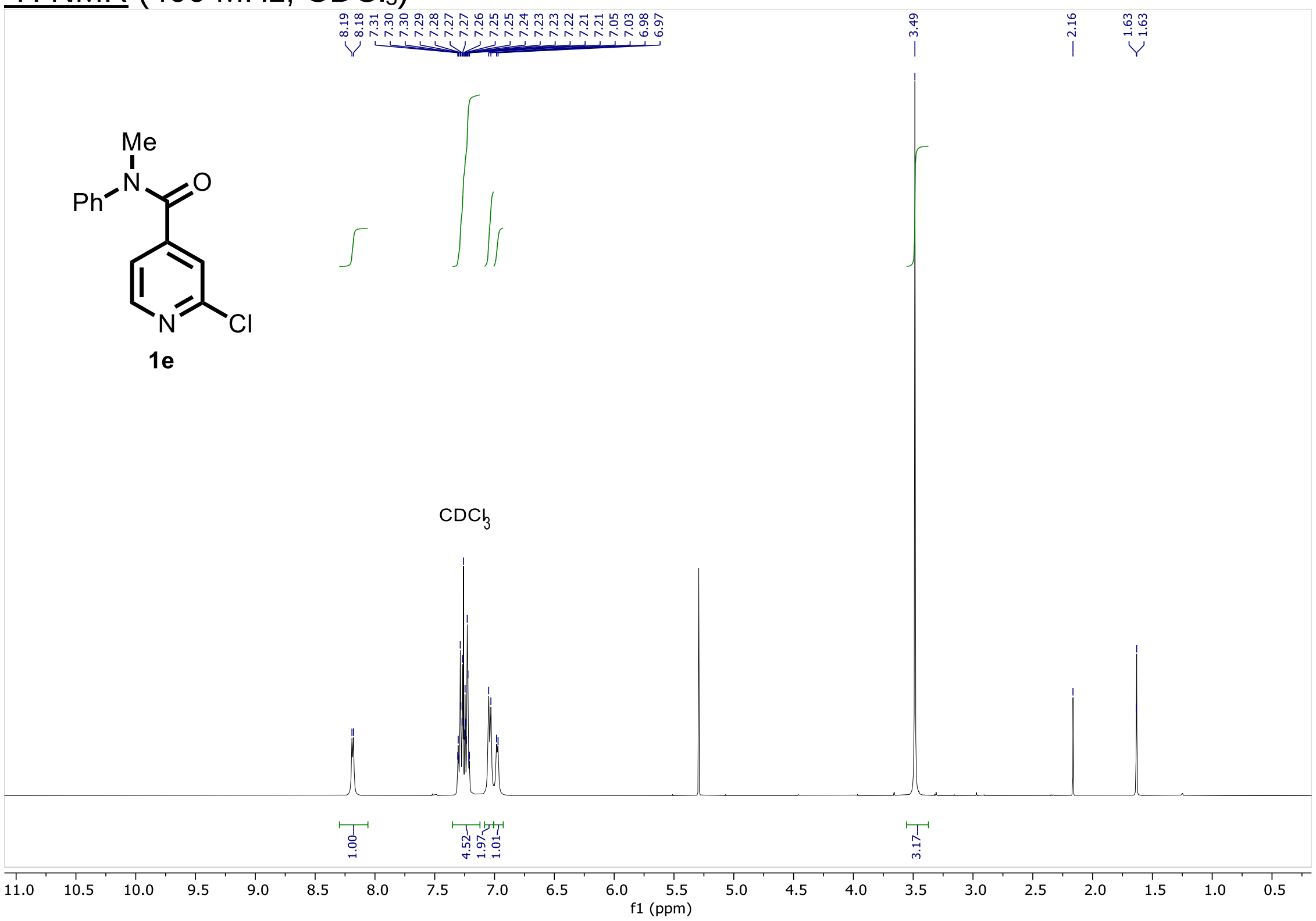

$^{13}C\{^{1}H\}$ NMR (101 MHz, $CDCl_3$)

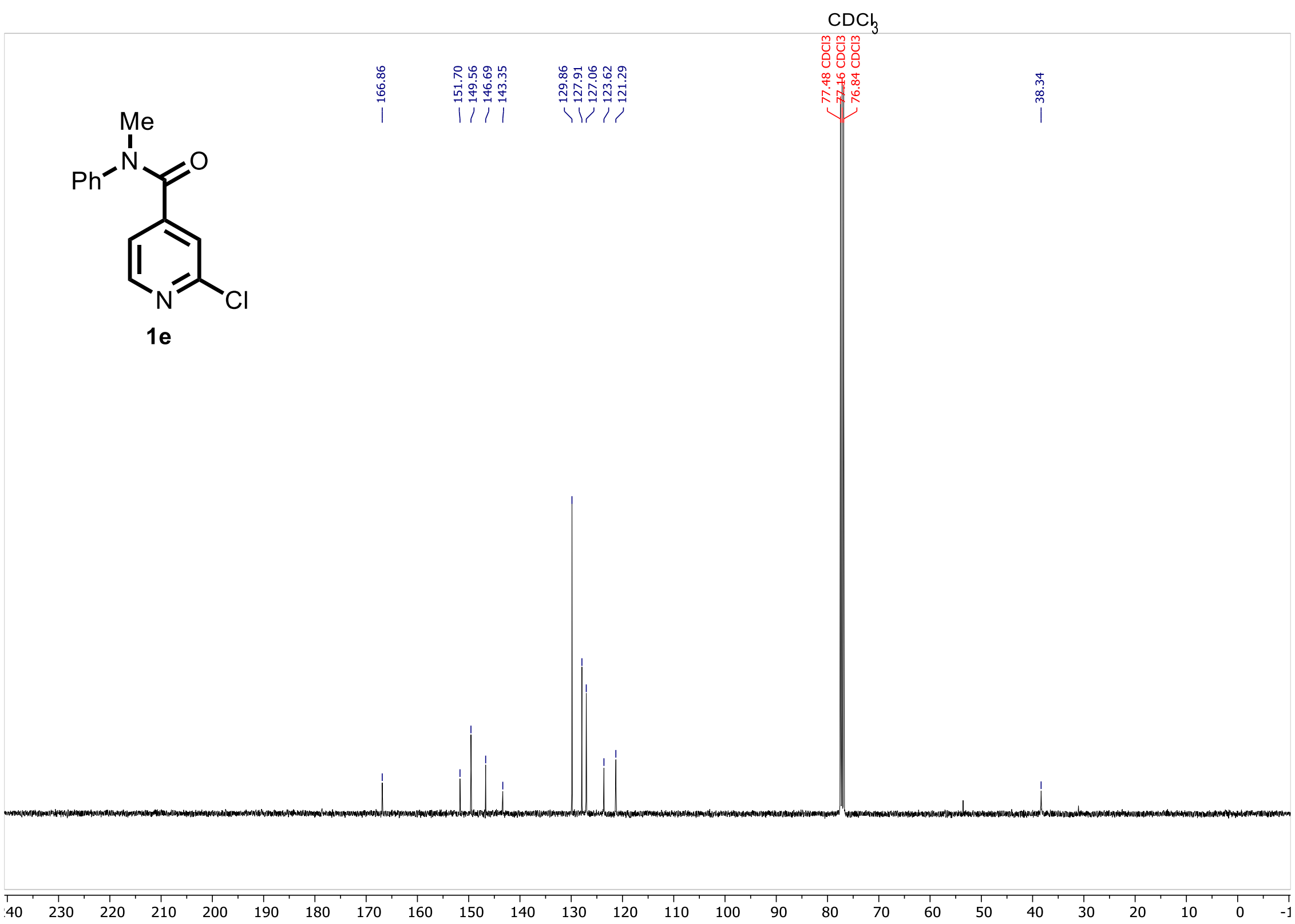

^{1}H NMR (400 MHz, $CDCl_3$)

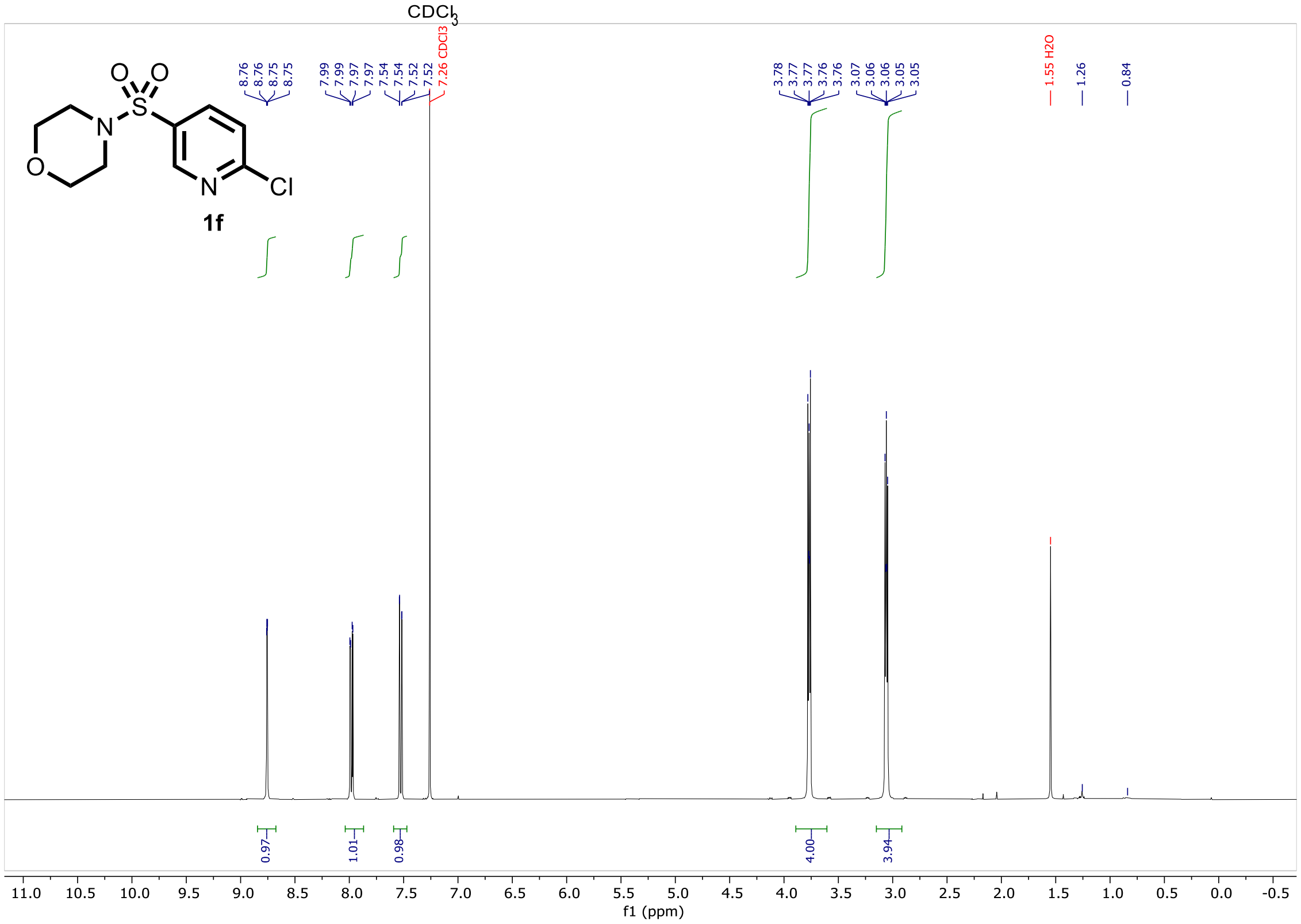

^{13}C{^{1}H} NMR (101 MHz, $CDCl_3$)

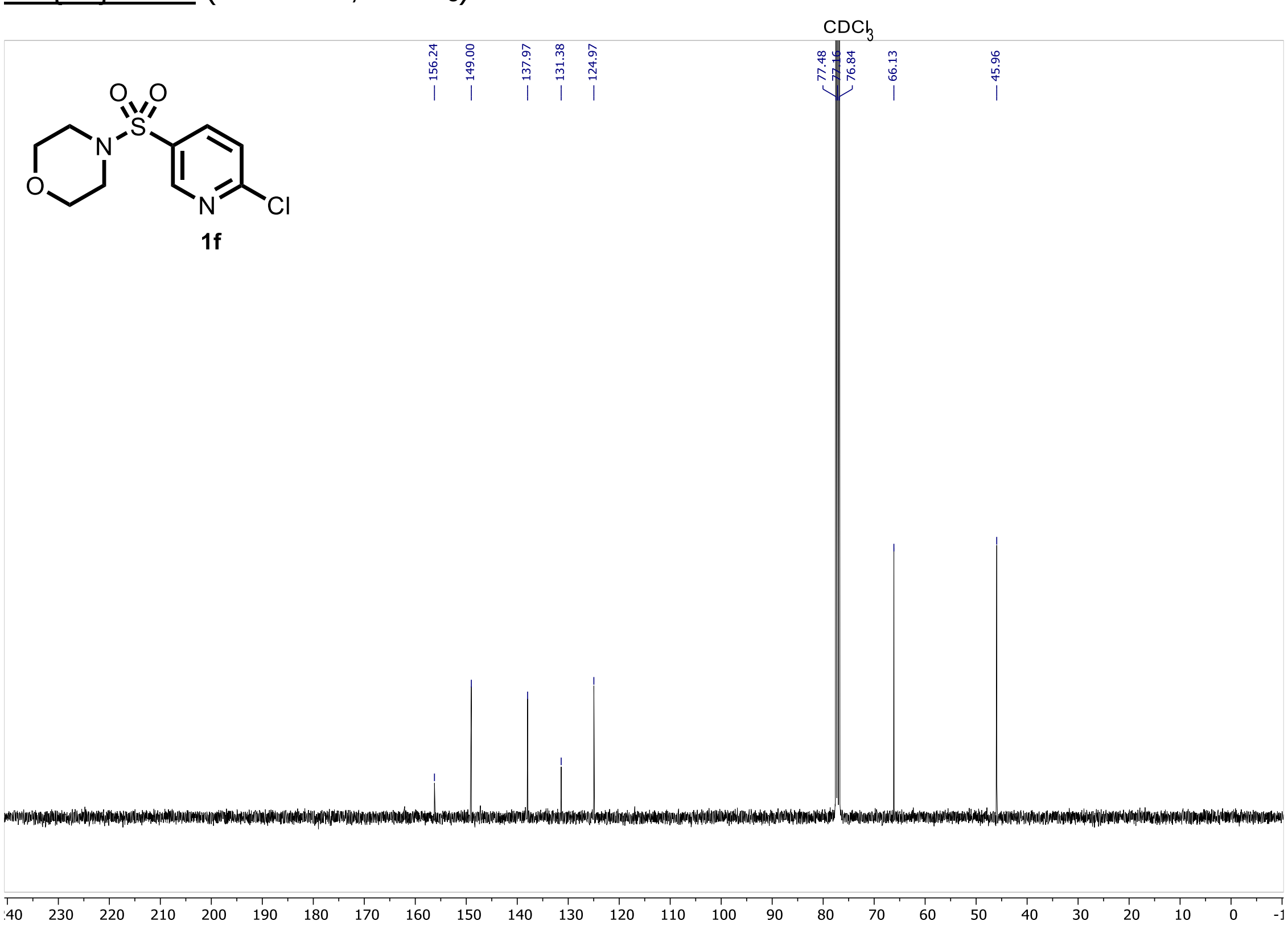

^{1}H NMR (500 MHz, $CDCl_3$)

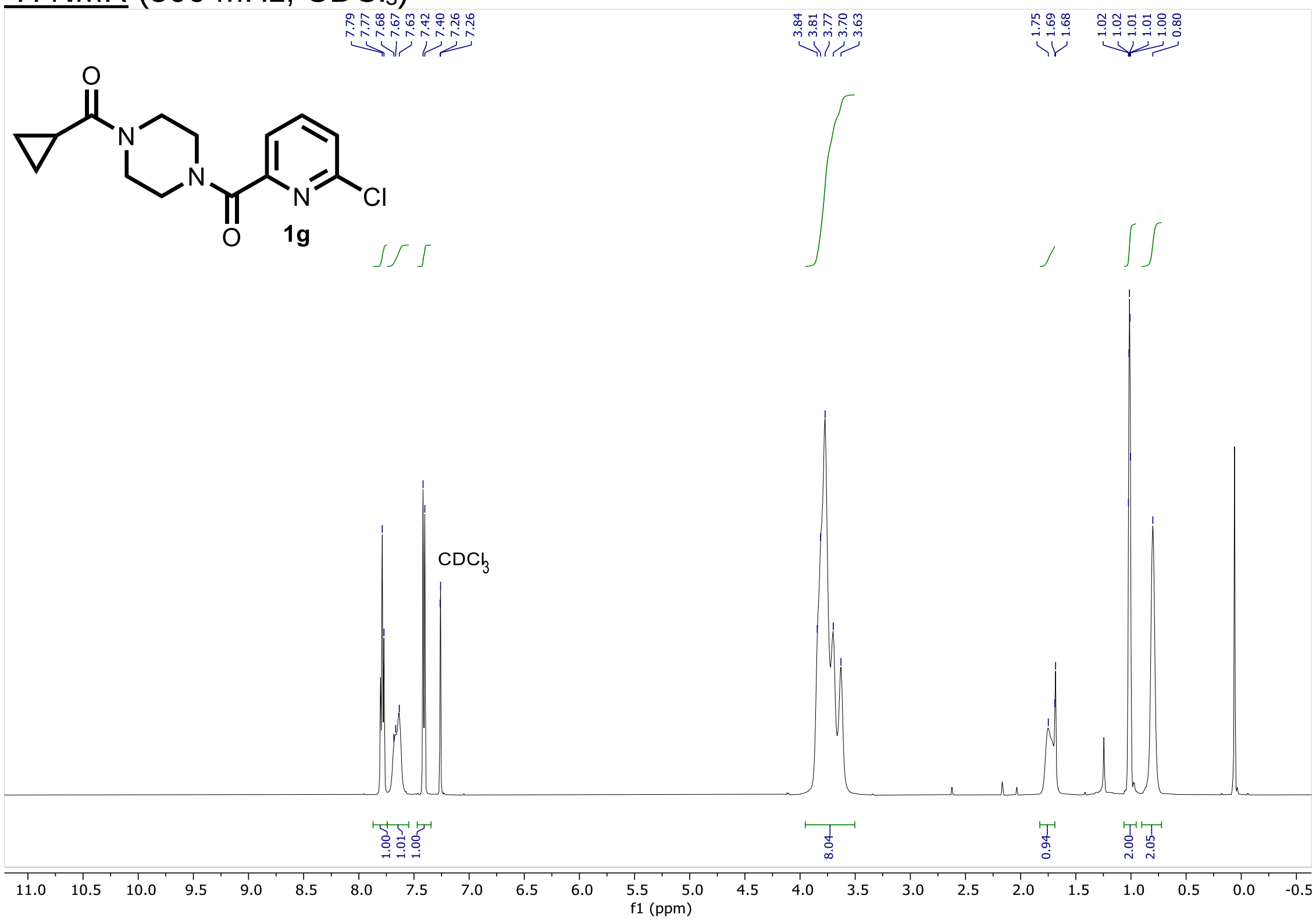

^{13}C{^{1}H} NMR (126 MHz, $CDCl_3$)

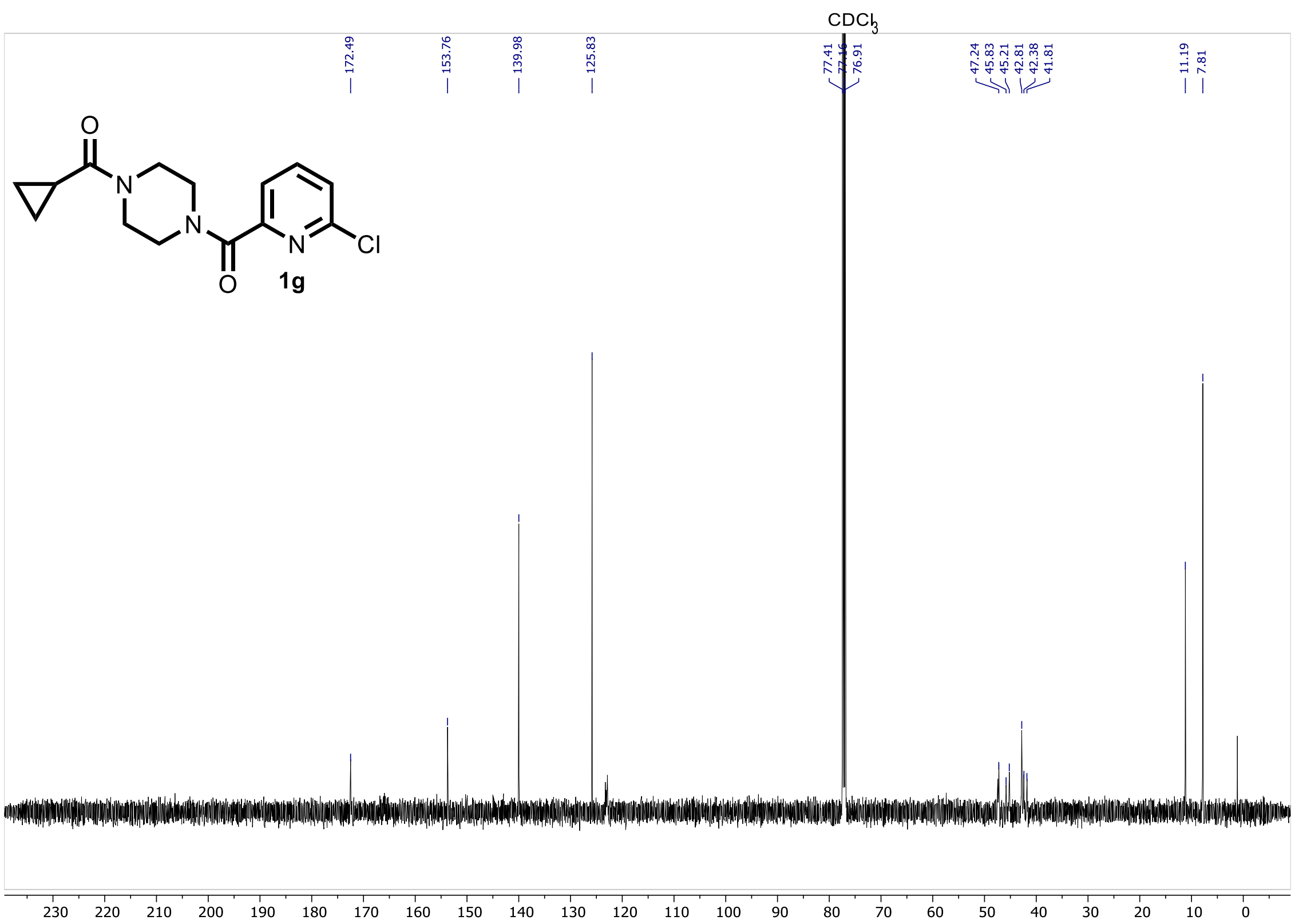

^{1}H NMR (400 MHz, $CDCl_3$)

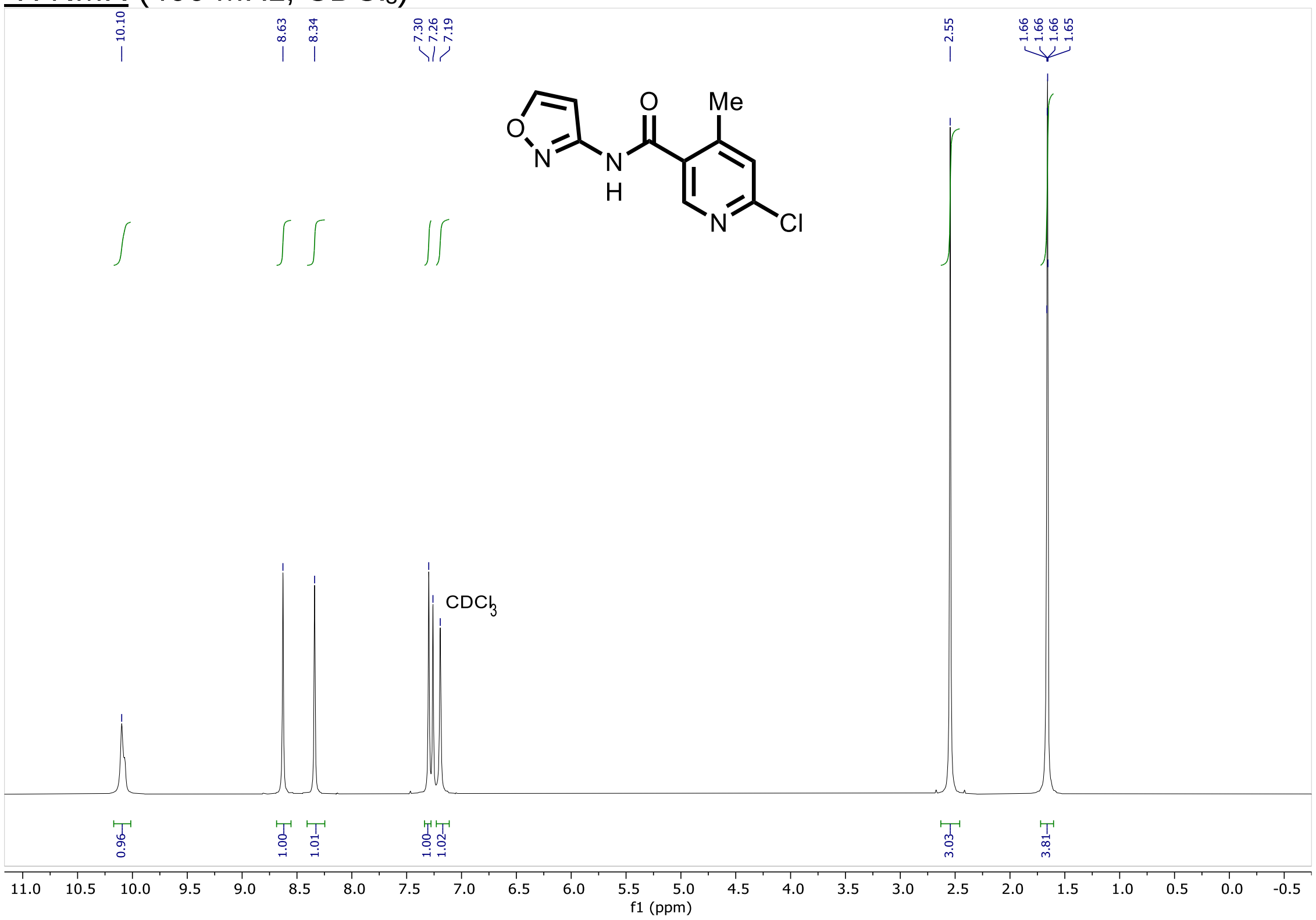

$^{13}C\{^{1}H\}$ NMR (101 MHz, $CDCl_3$)

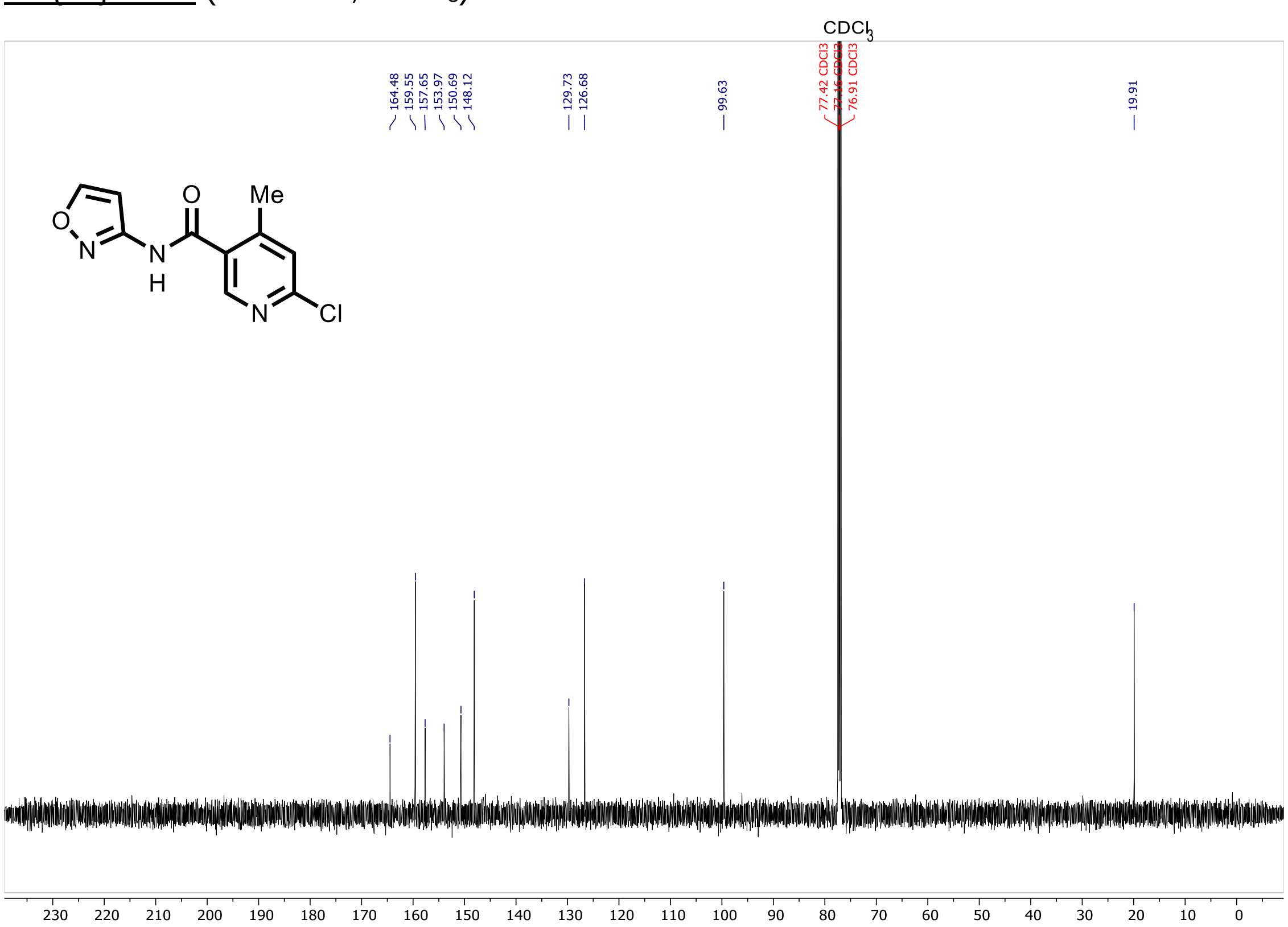

^{1}H NMR (500 MHz, $CDCl_3$)

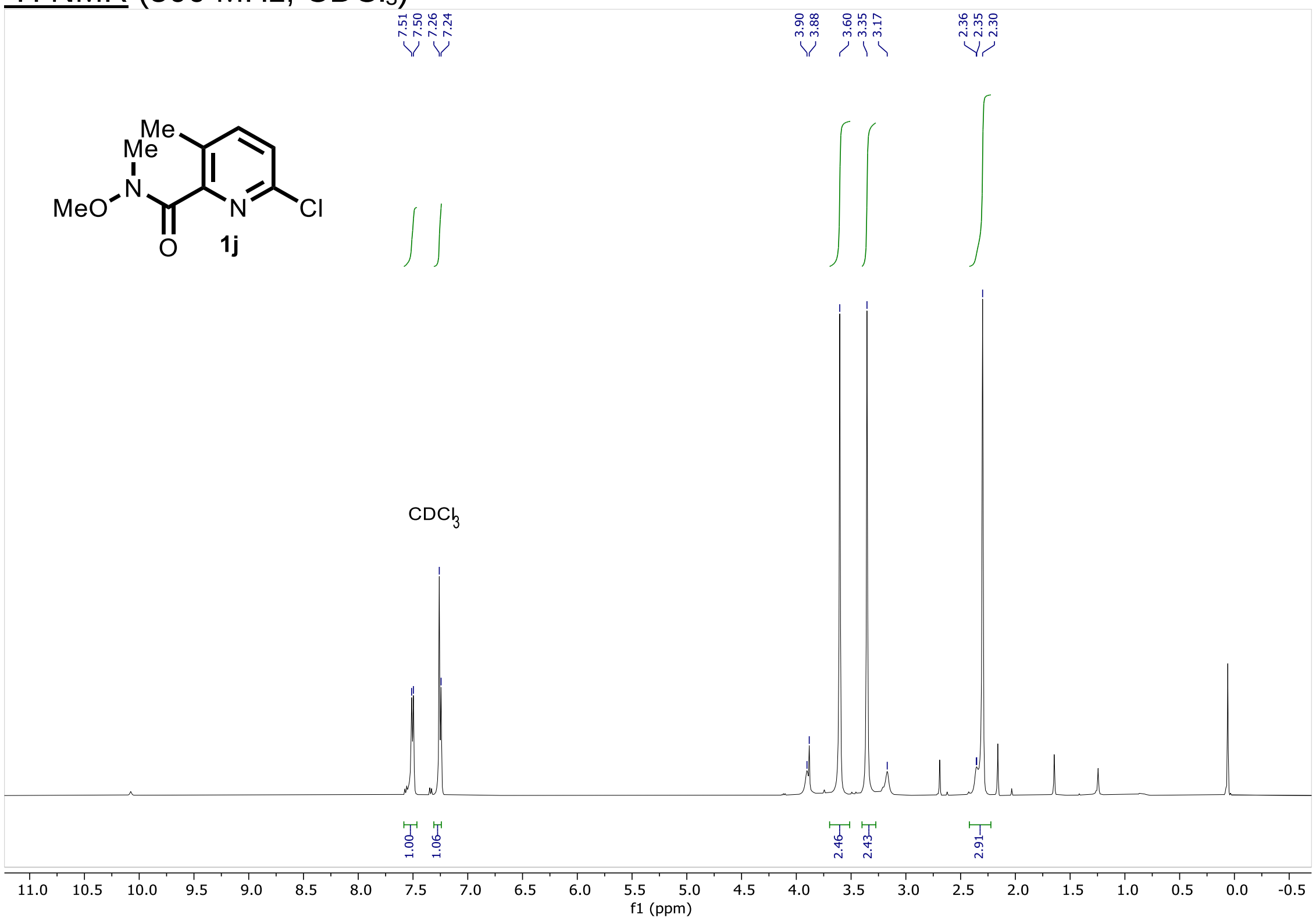

^{13}C{^{1}H} NMR (126 MHz, $CDCl_3$)

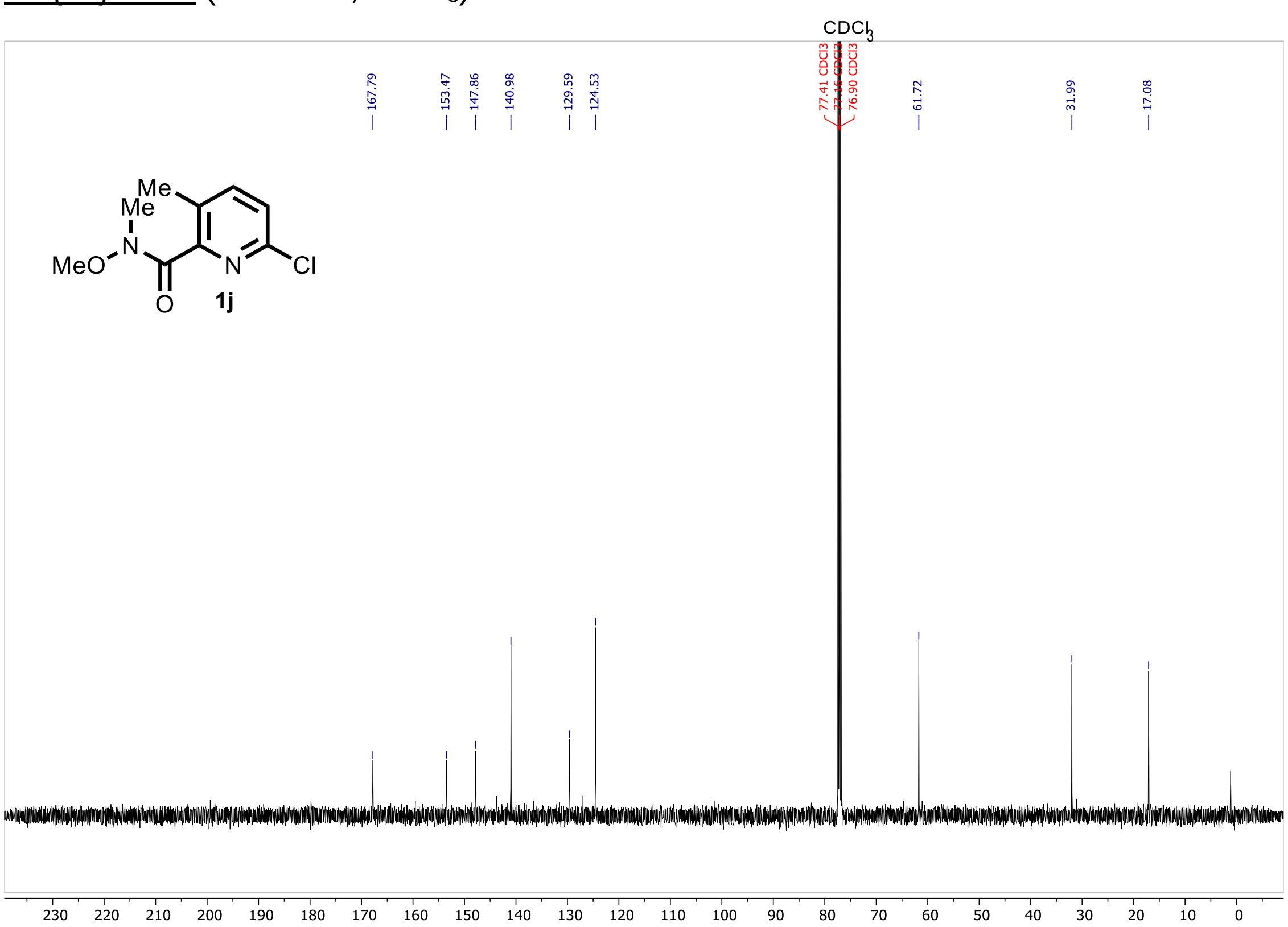

^{1}H NMR (500 MHz, $CDCl_3$)

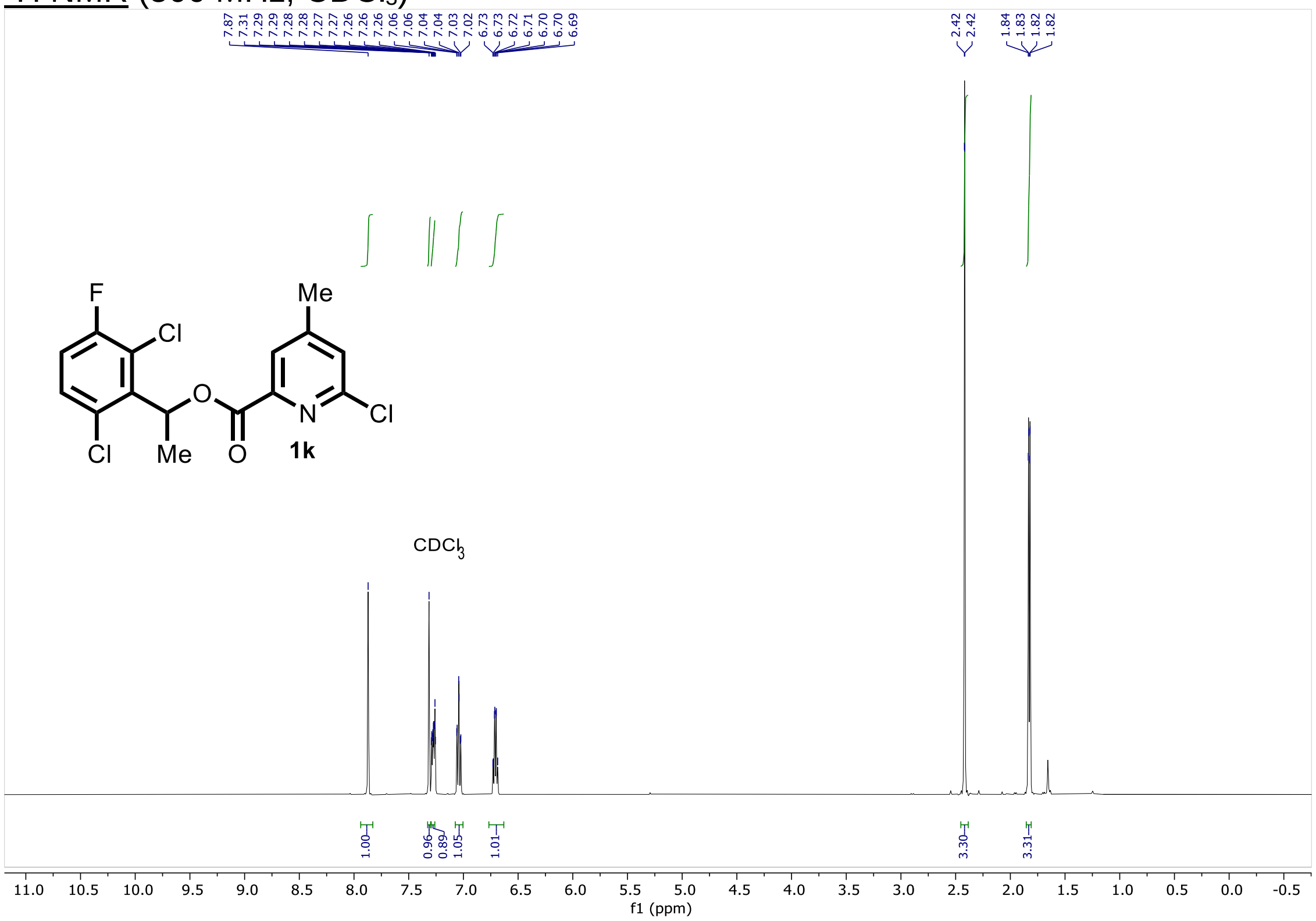

^{19}F NMR (470 MHz, $CDCl_3$)

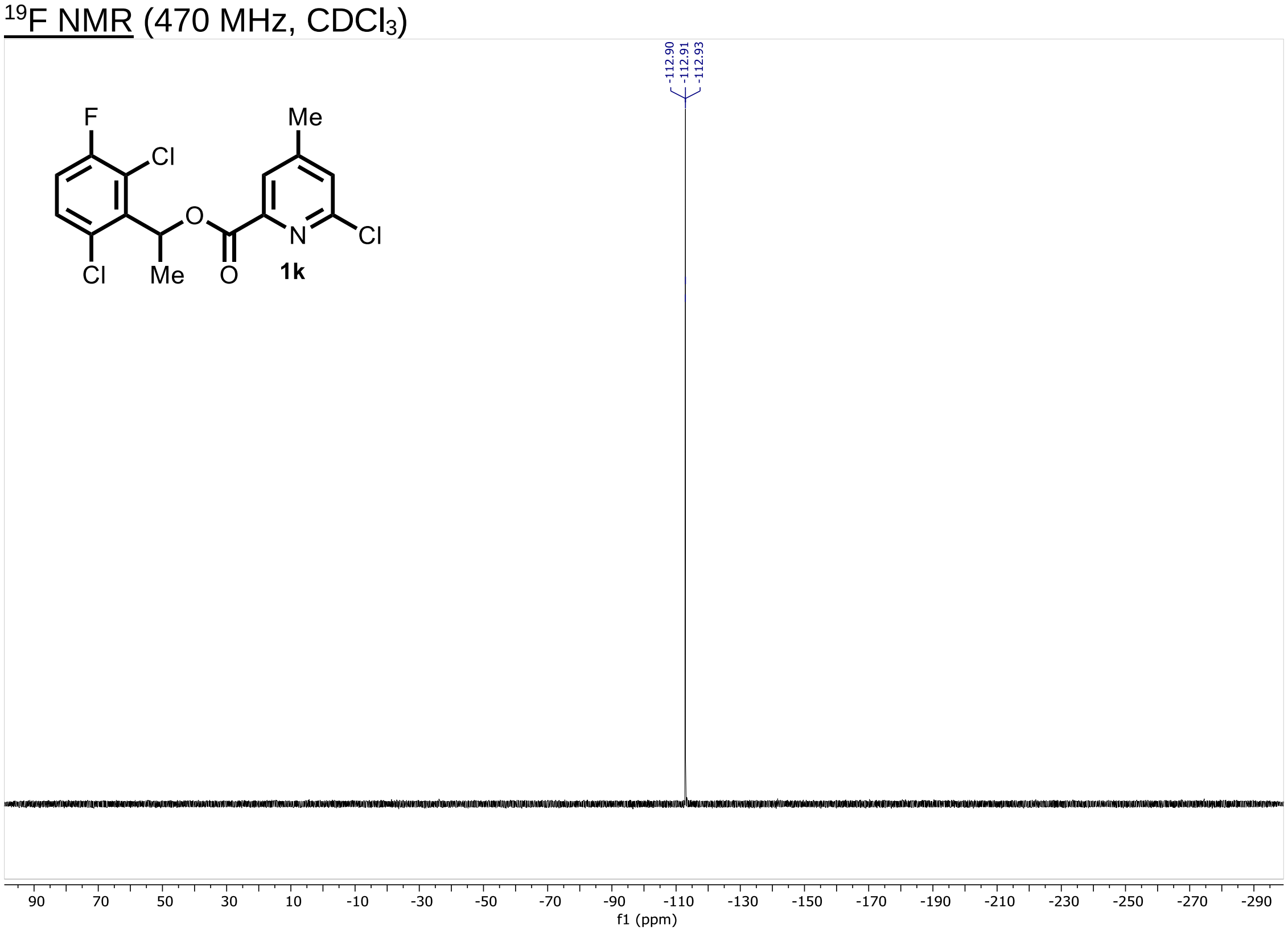

$^{13}C\{^{1}H\}$ NMR (126 MHz, $CDCl_3$)

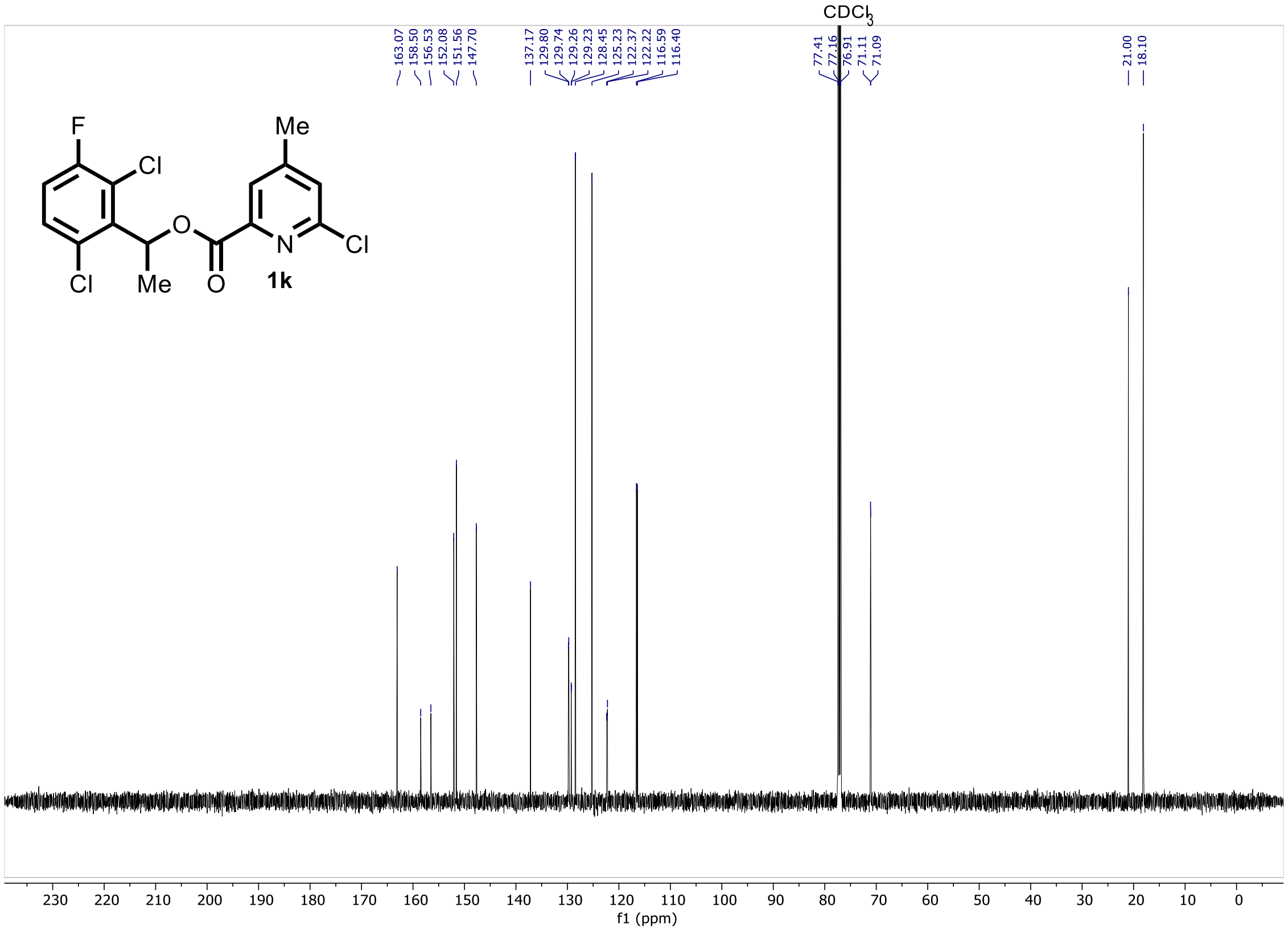

^{1}H NMR (400 MHz, $CDCl_3$)

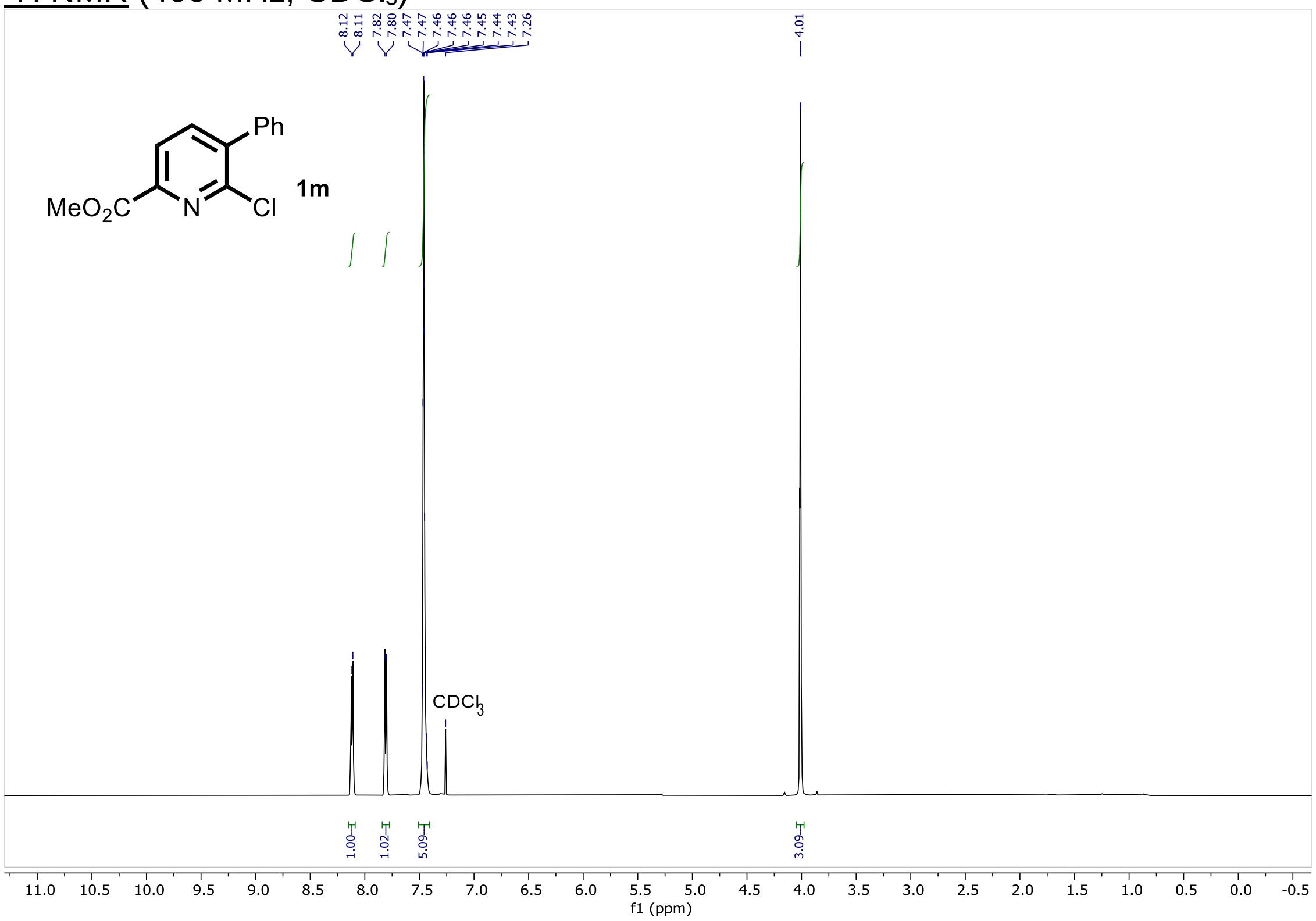

^{13}C{^{1}H} NMR (101 MHz, $CDCl_3$)

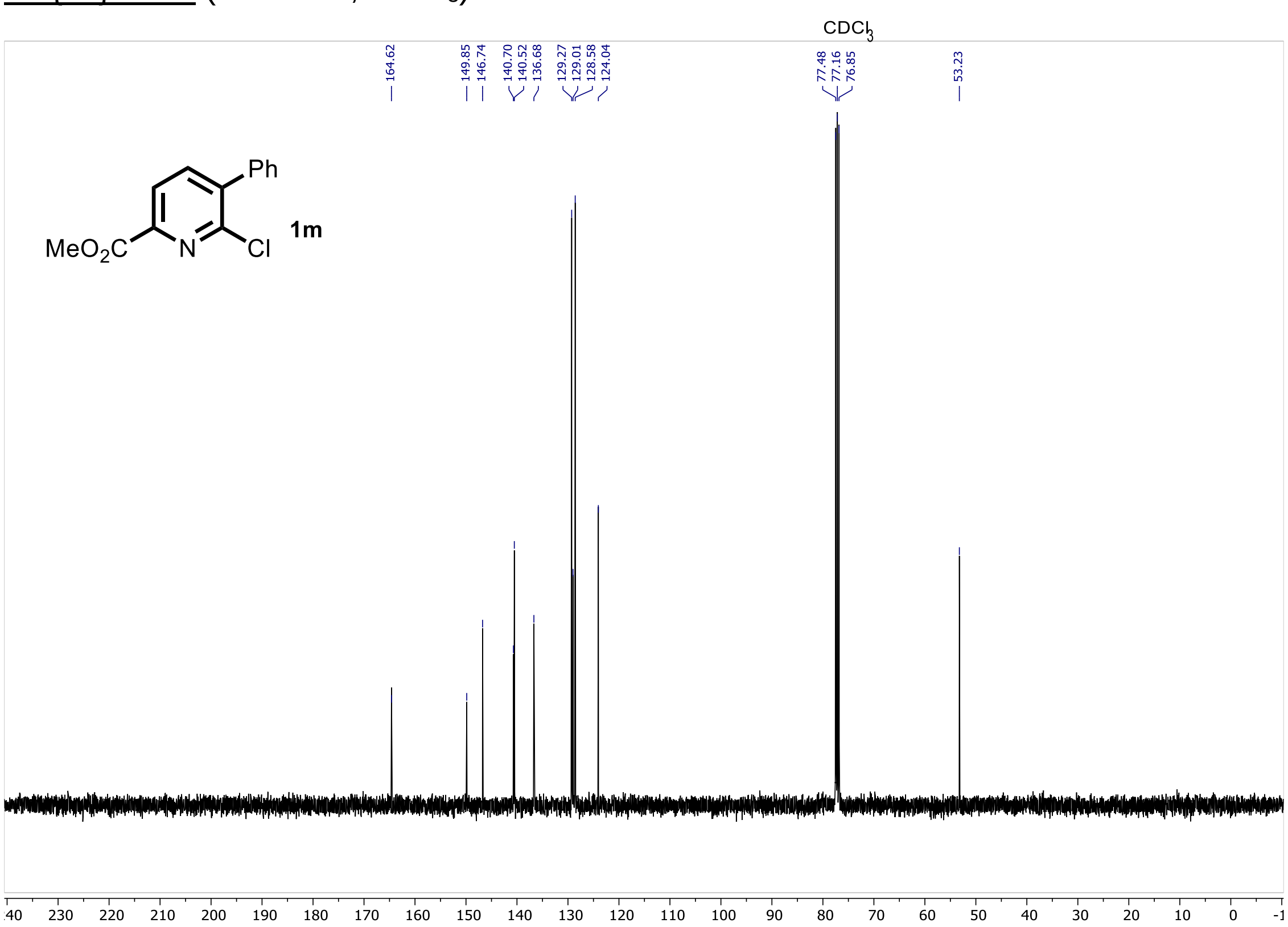

^1H NMR (400 MHz, $CDCl_3$)

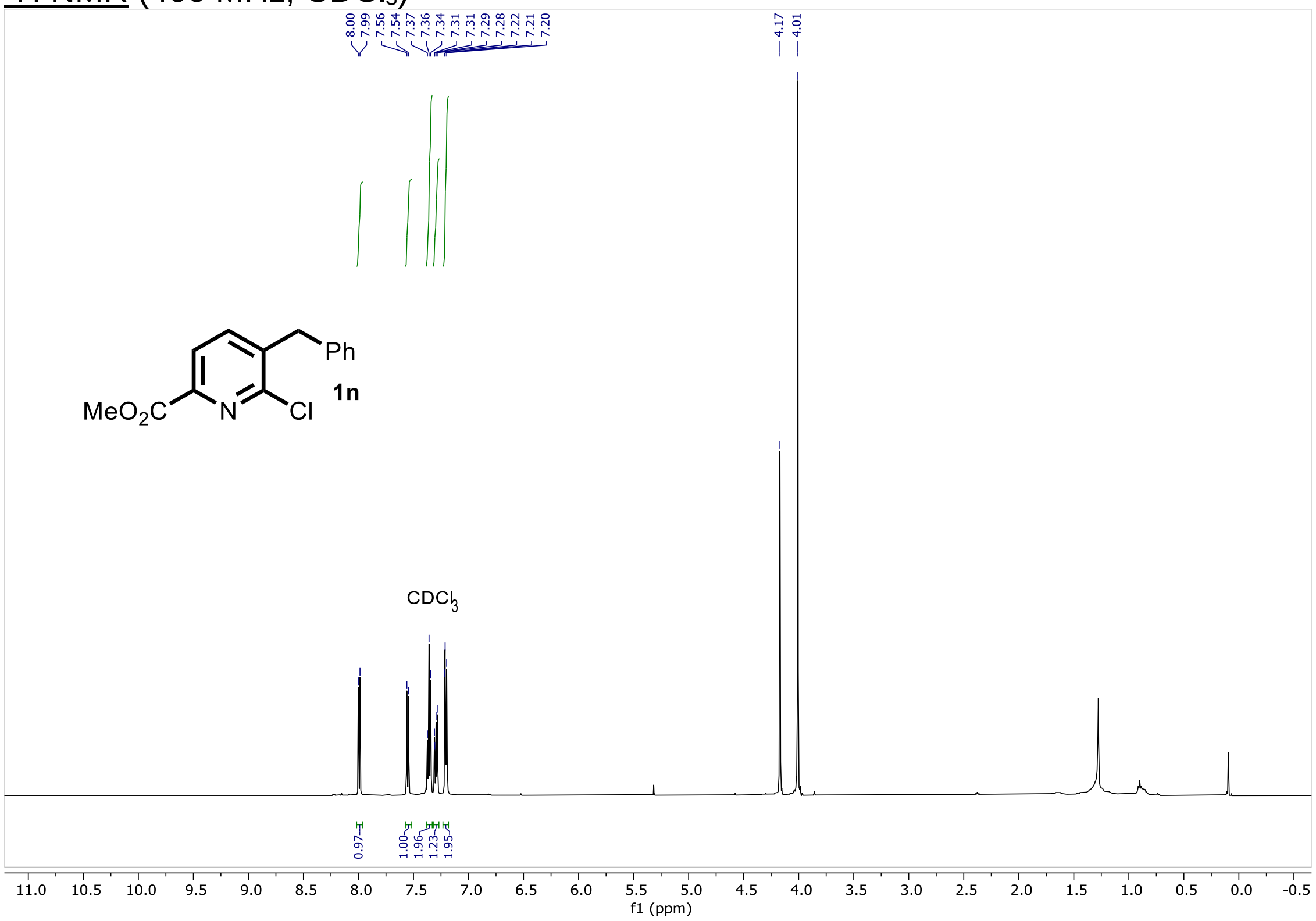

$^{13}C\{^1H\}$ NMR (101 MHz, $CDCl_3$)

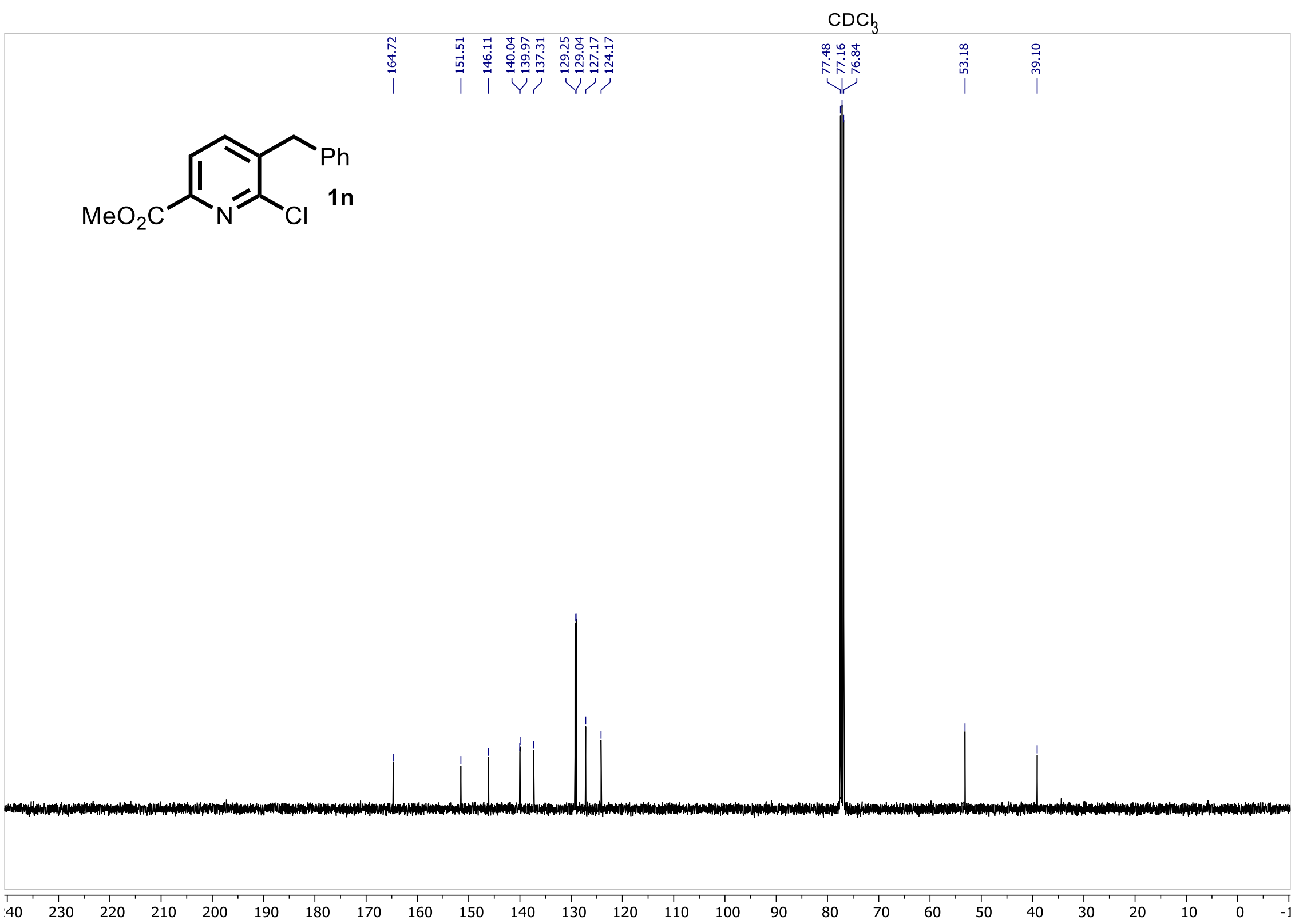

^{1}H NMR (400 MHz, $CDCl_3$)

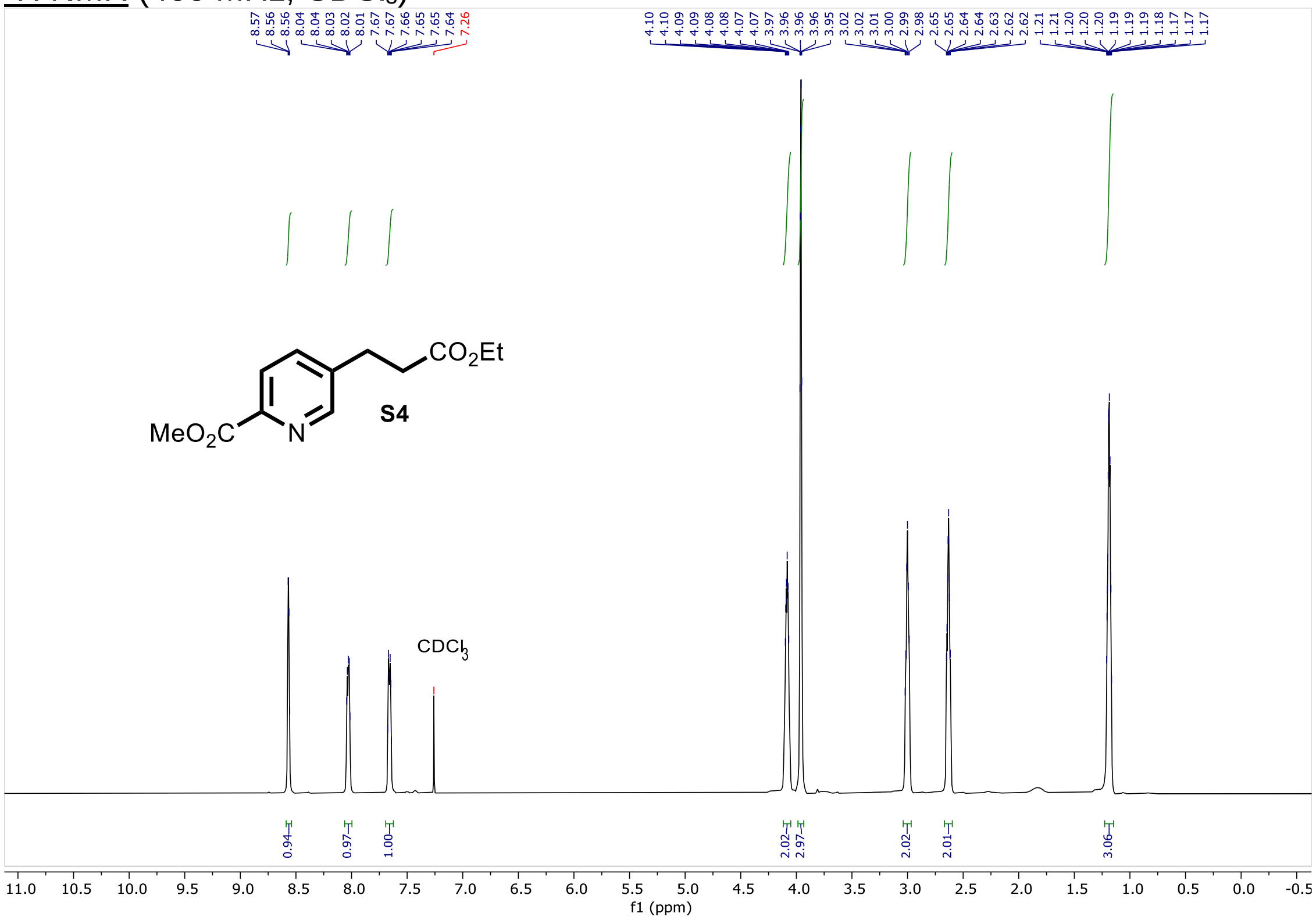

$^{13}C\{^{1}H\}$ NMR (101 MHz, $CDCl_3$)

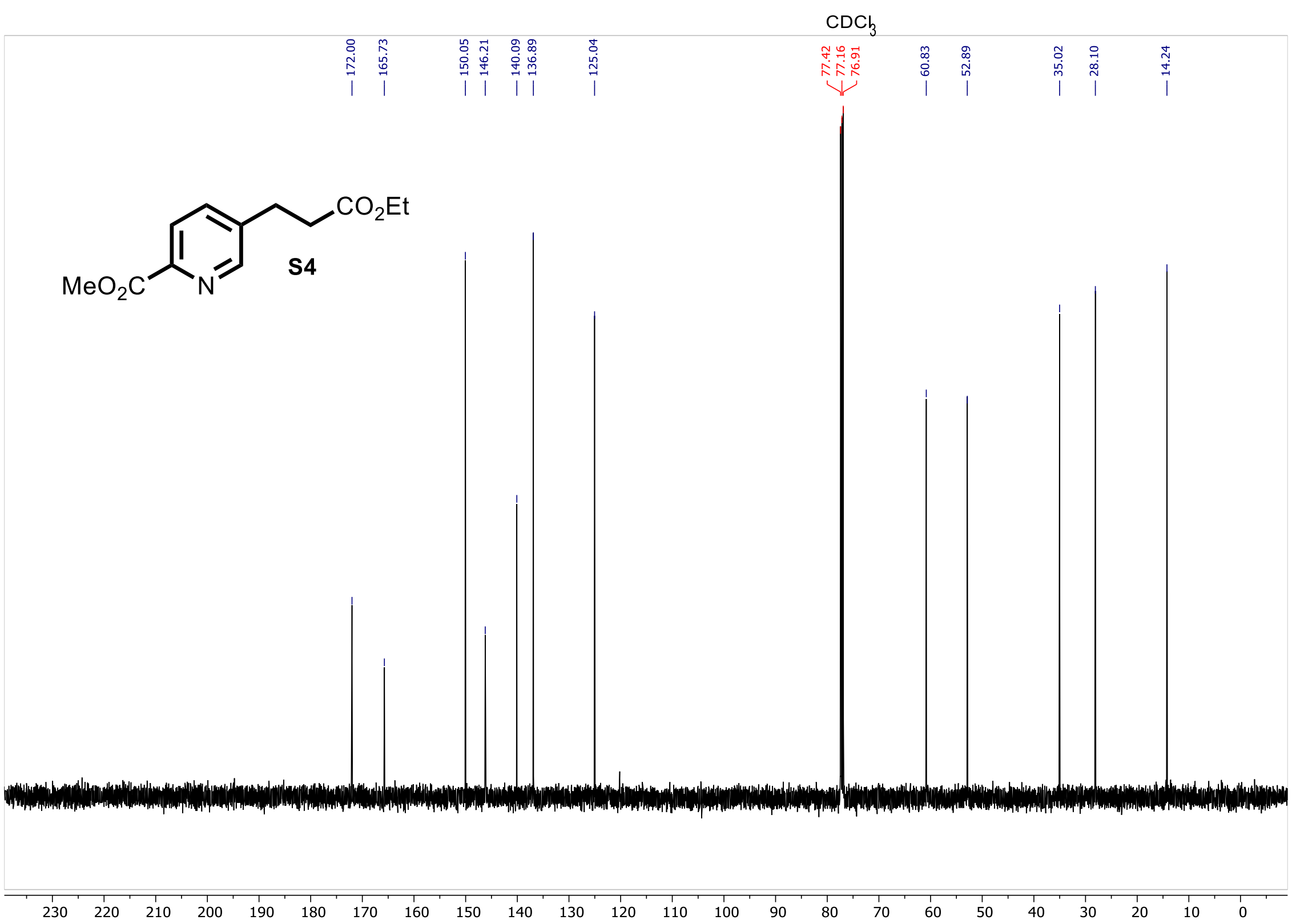

^{1}H NMR (400 MHz, $CDCl_3$)

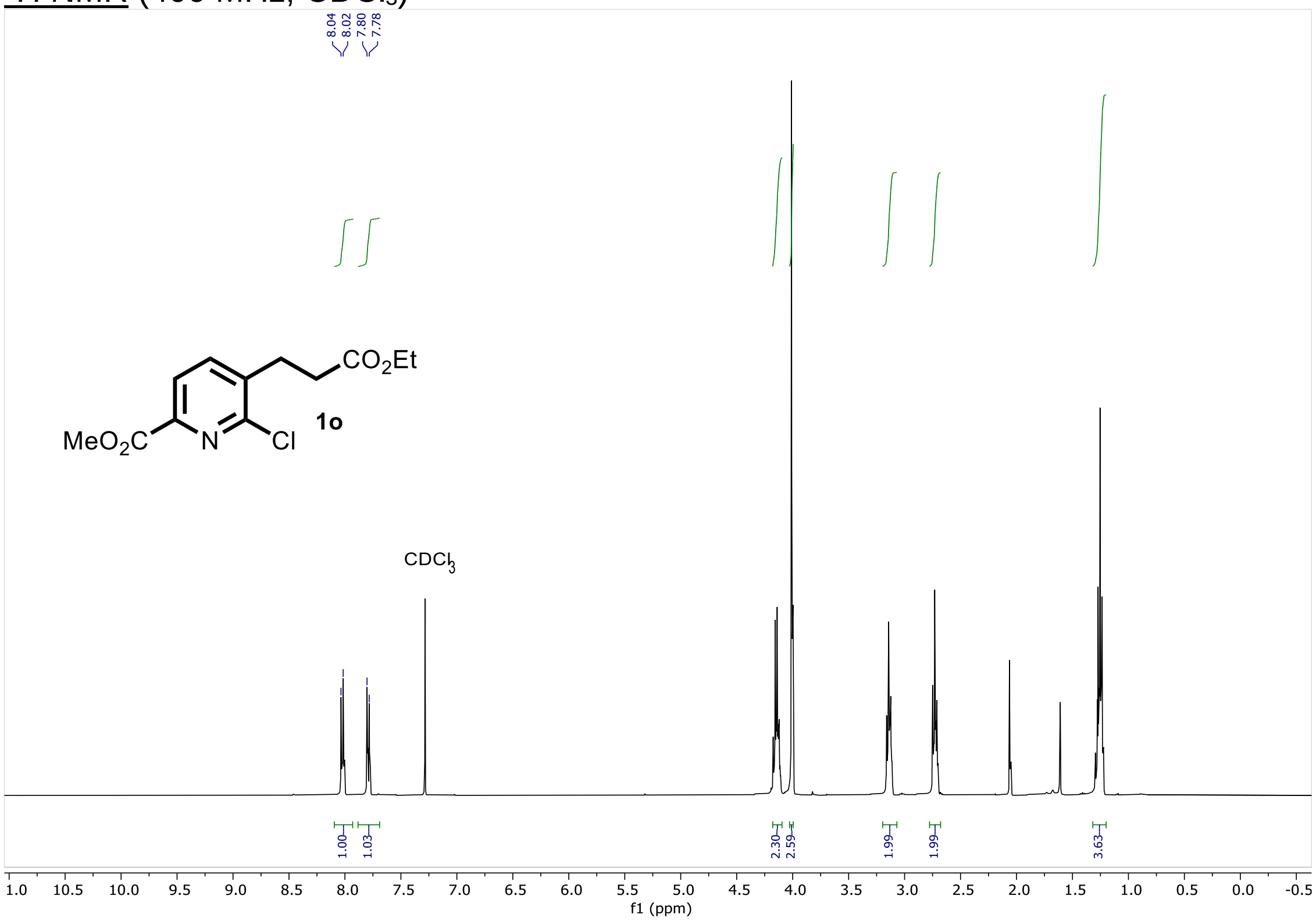

^{13}C{^{1}H} NMR (101 MHz, $CDCl_3$)

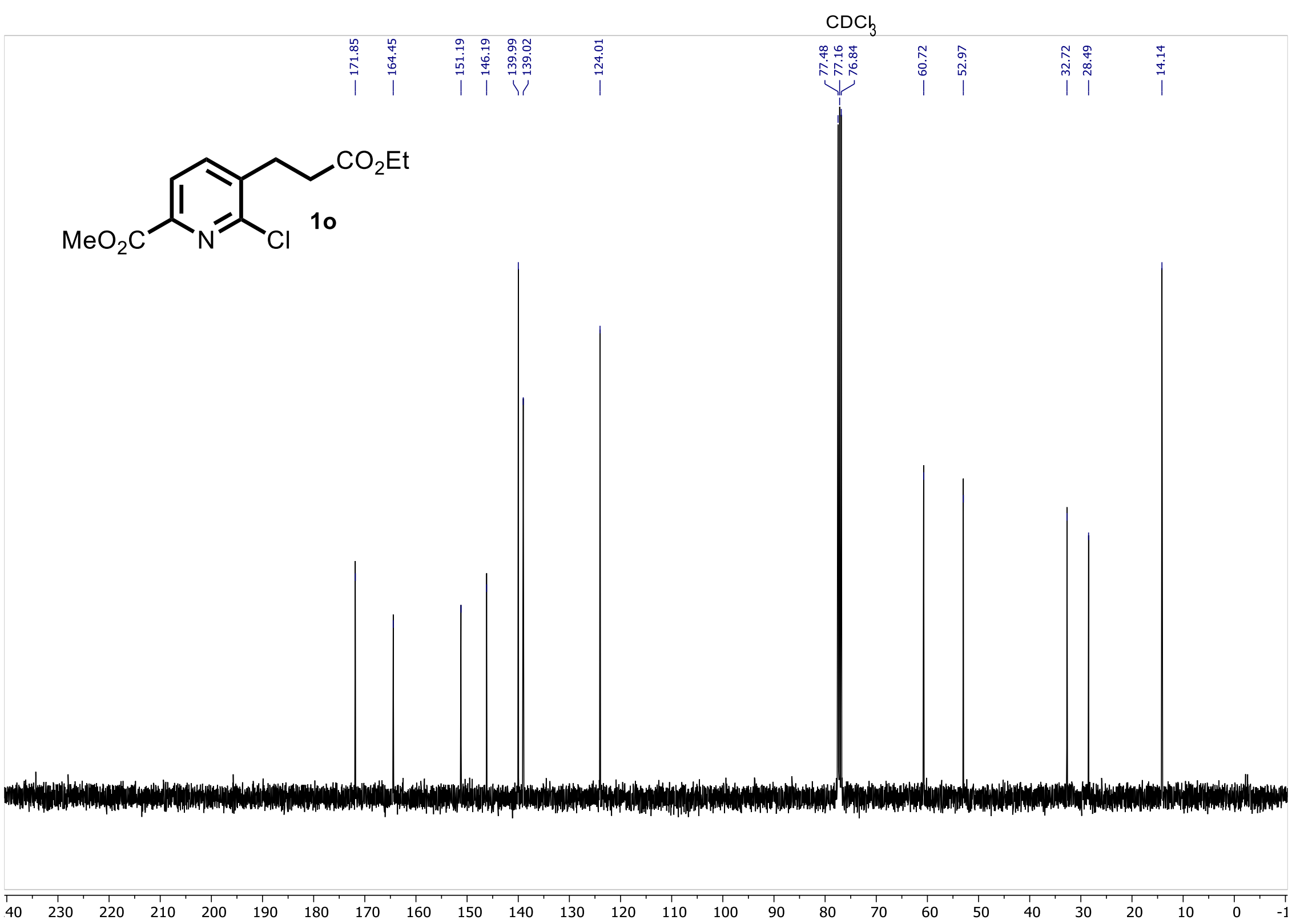

^{1}H NMR (400 MHz, $CDCl_3$)

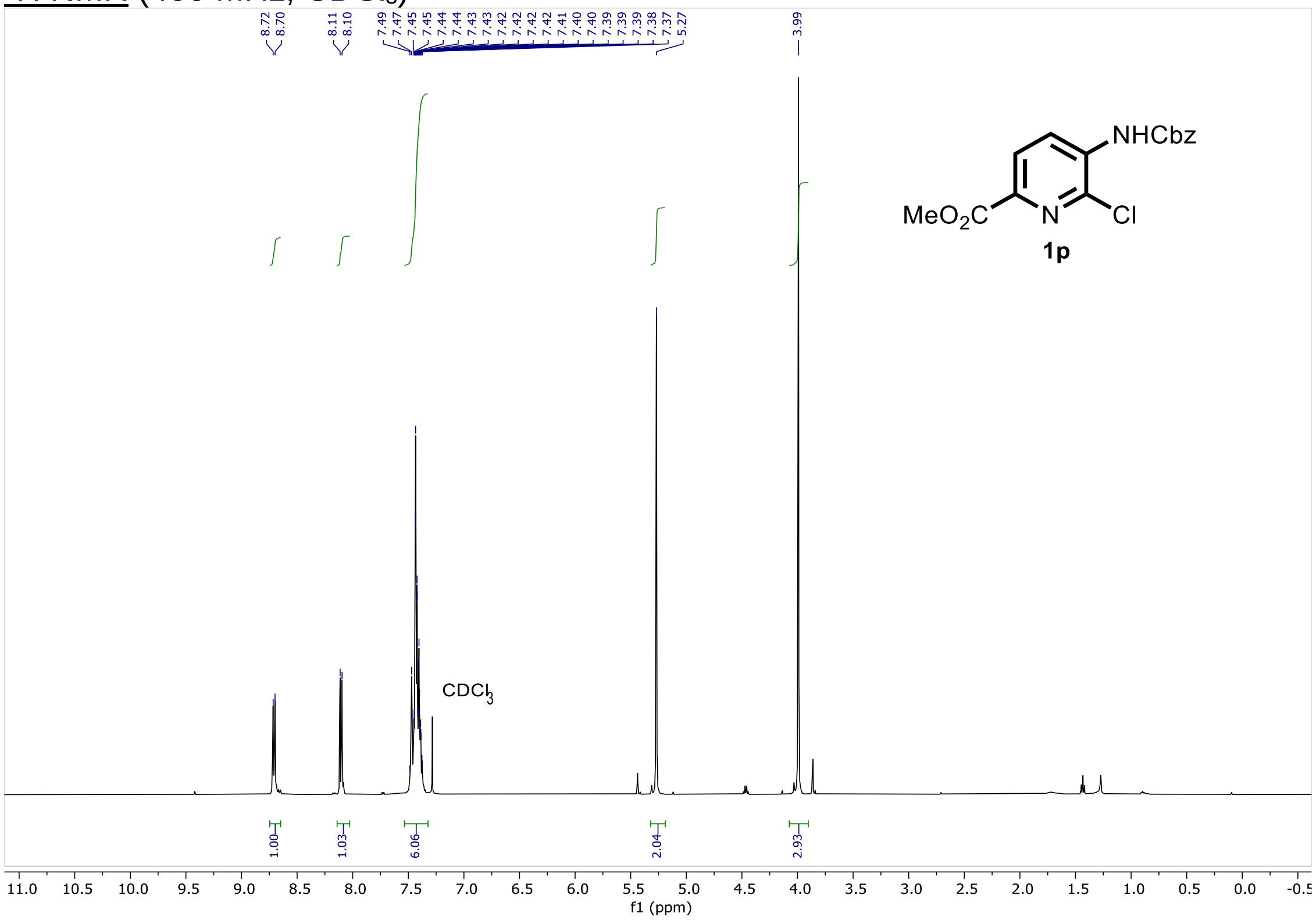

$^{13}C\{^{1}H\}$ NMR (101 MHz, $CDCl_3$)

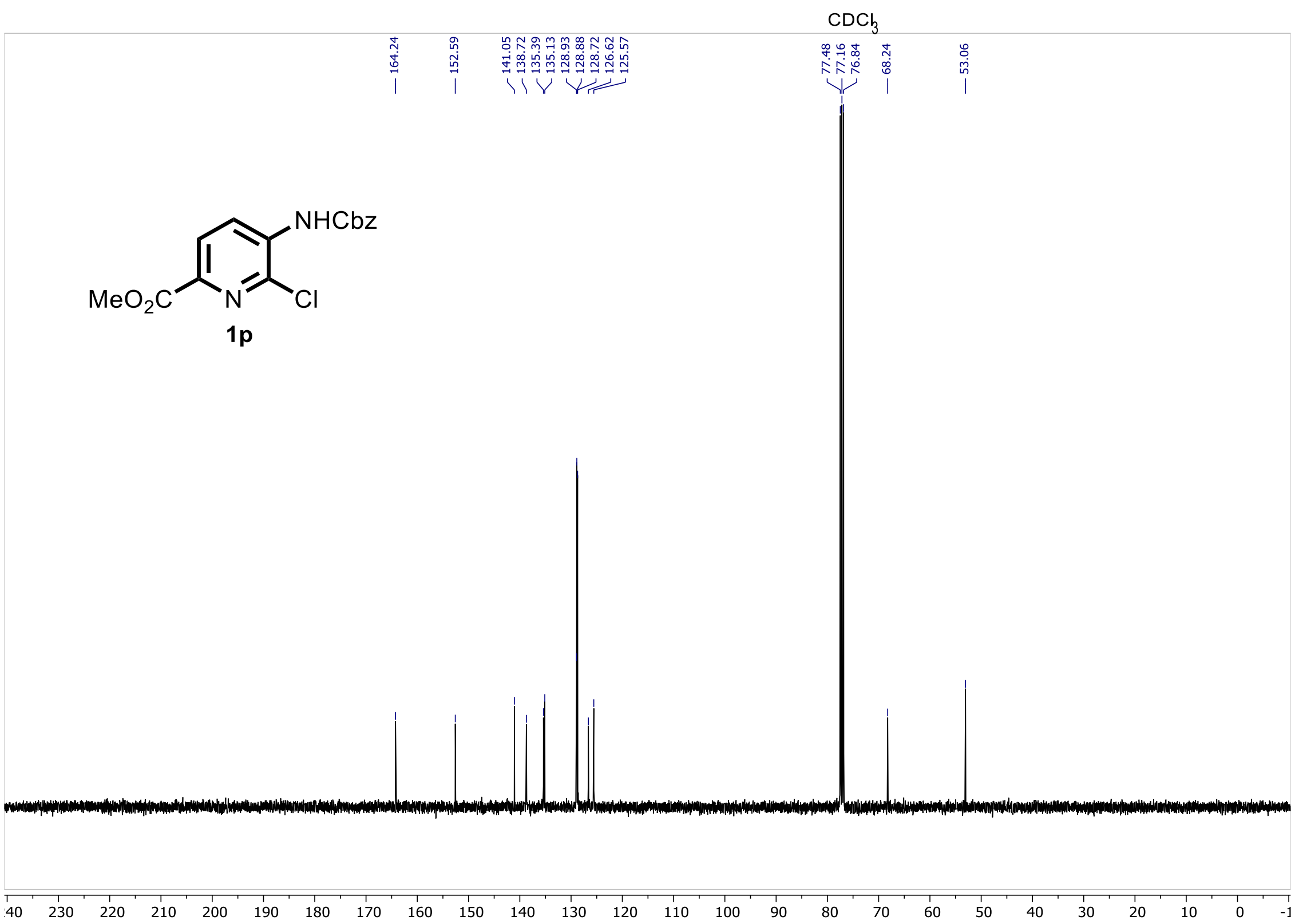

^{1}H NMR (400 MHz, $CDCl_3$)

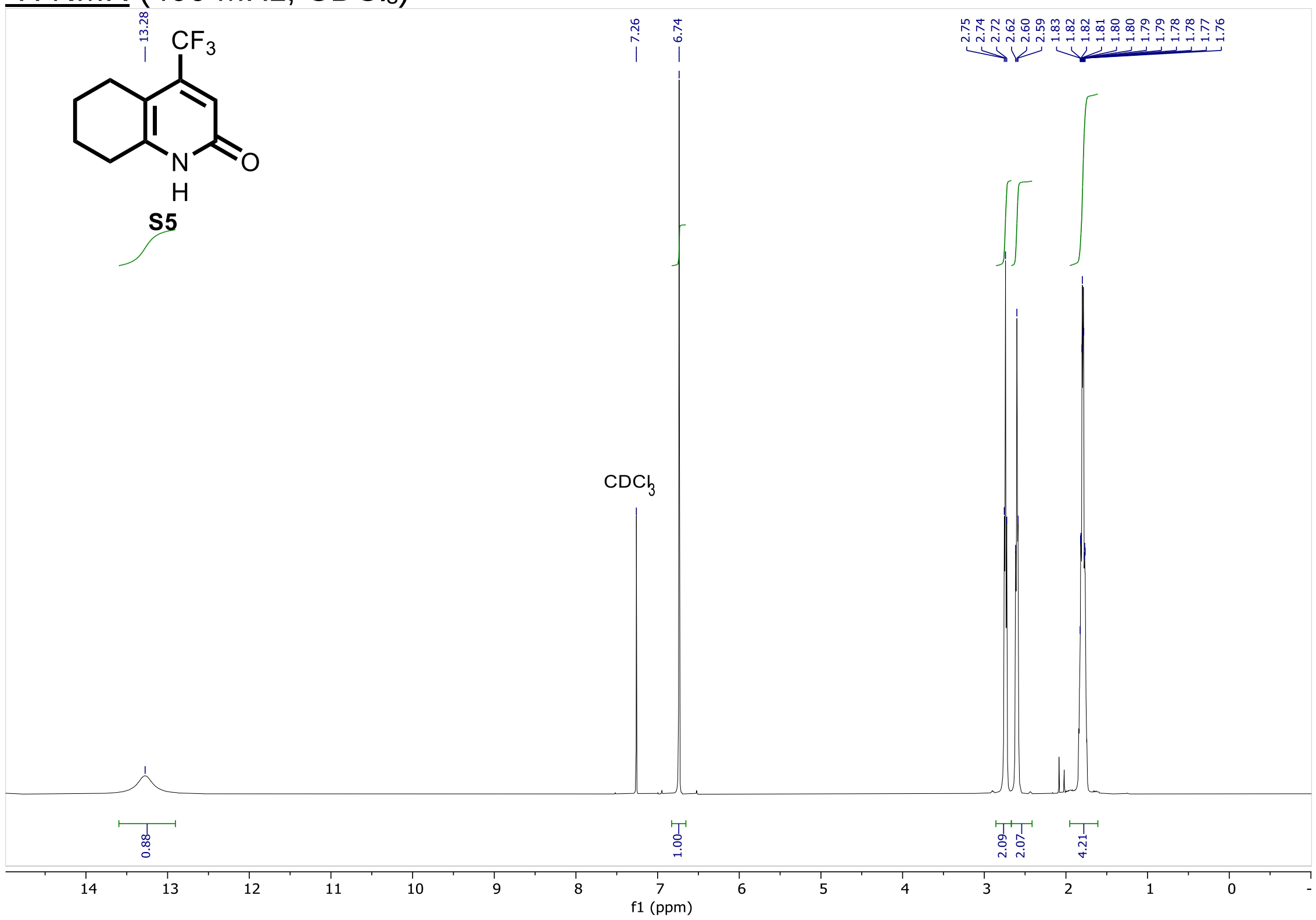

^{19}F NMR (376 MHz, $CDCl_3$)

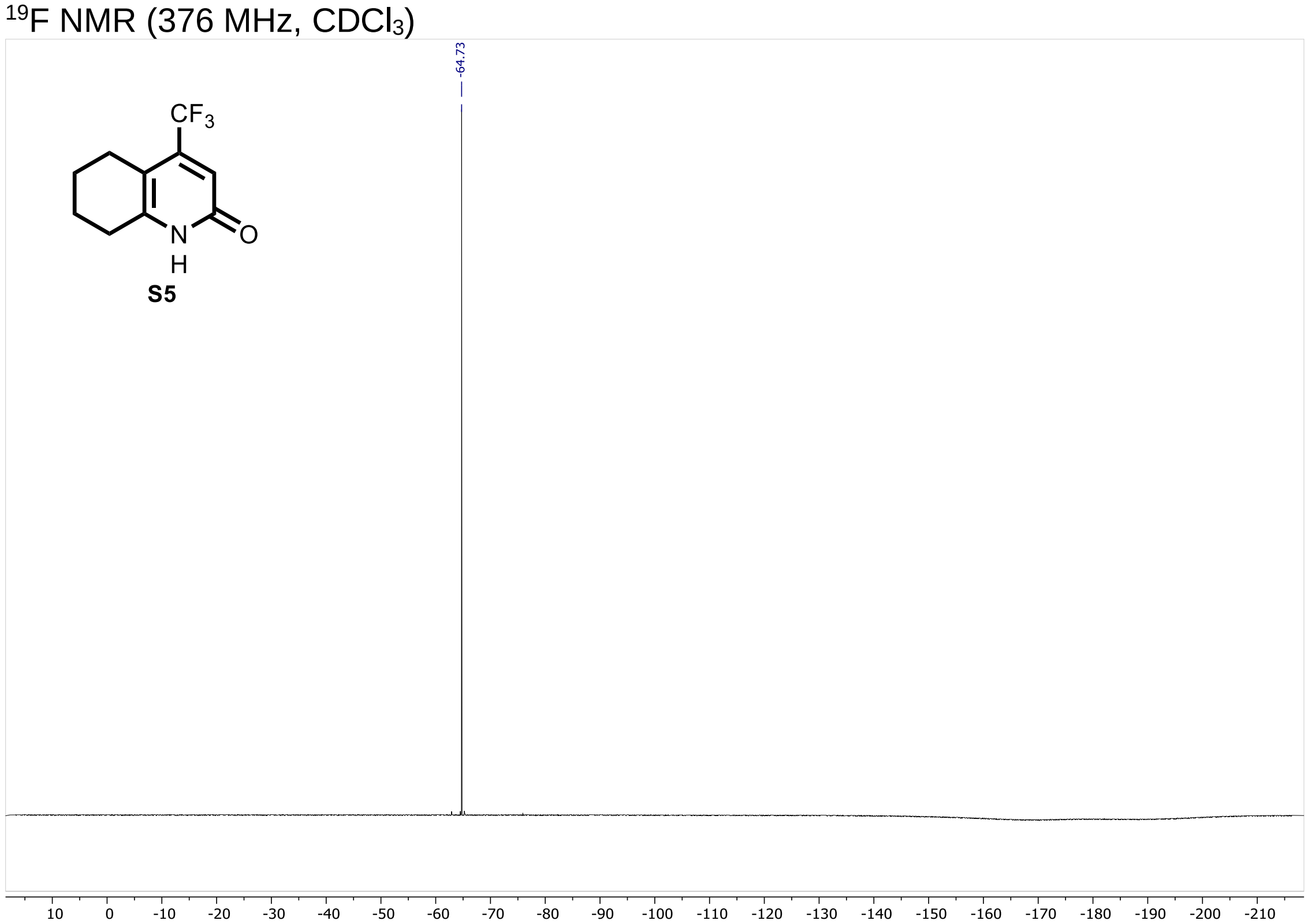

$^{13}C\{^{1}H\}$ NMR (101 MHz, $CDCl_3$)

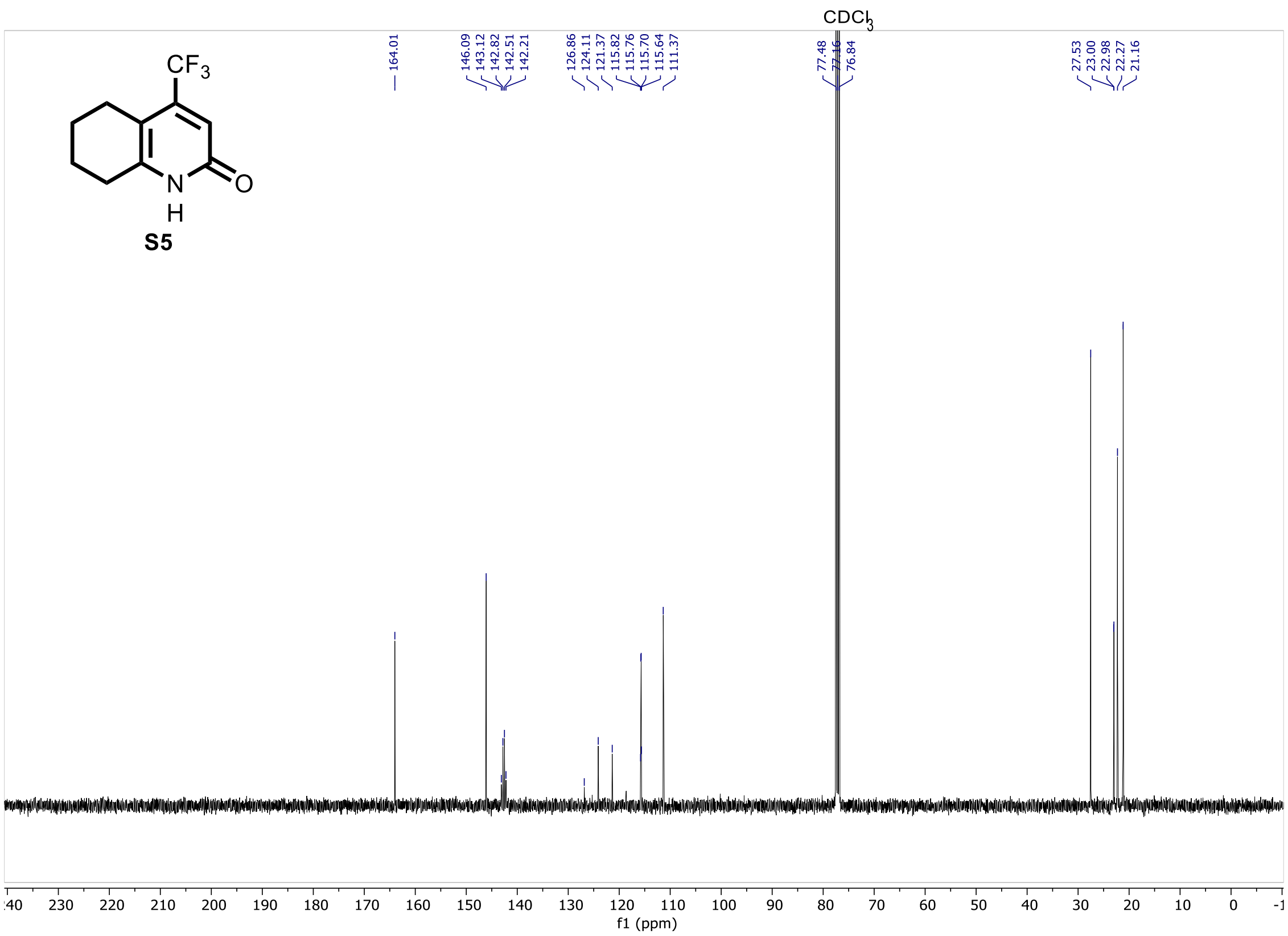

^{1}H NMR (400 MHz, $CDCl_3$)

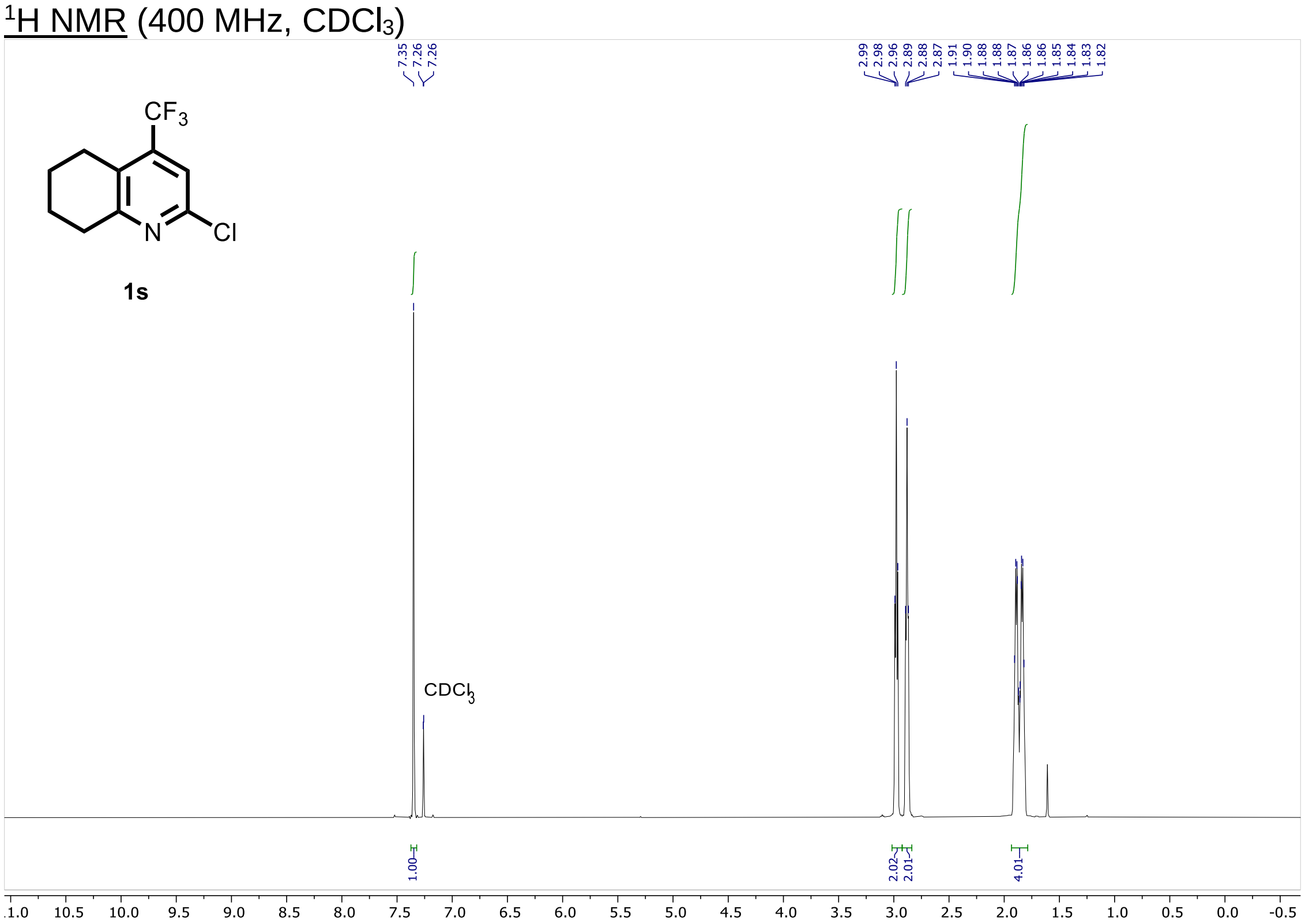

^{19}F NMR (376 MHz, $CDCl_3$)

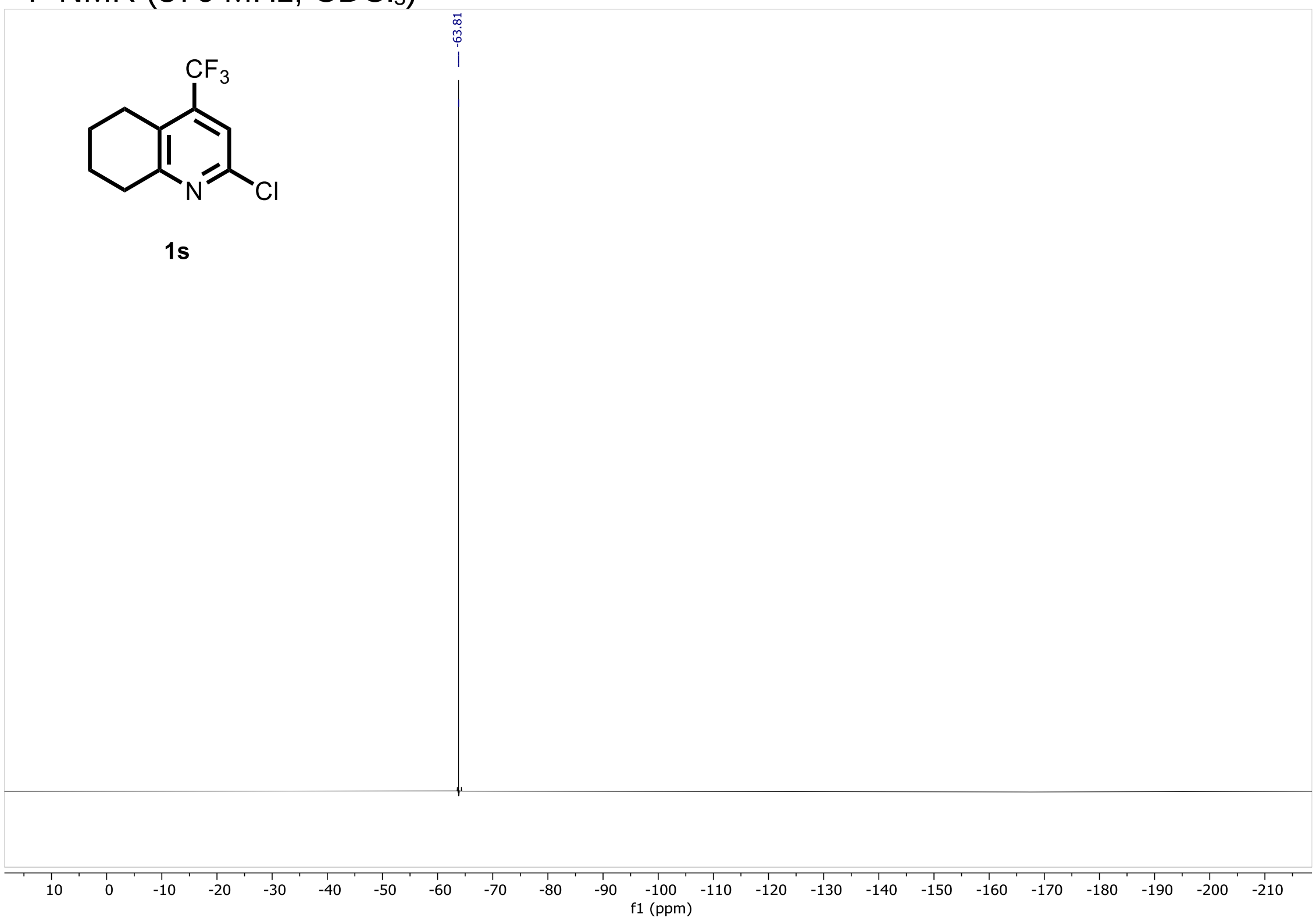

^{13}C{^{1}H} NMR (101 MHz, $CDCl_3$)

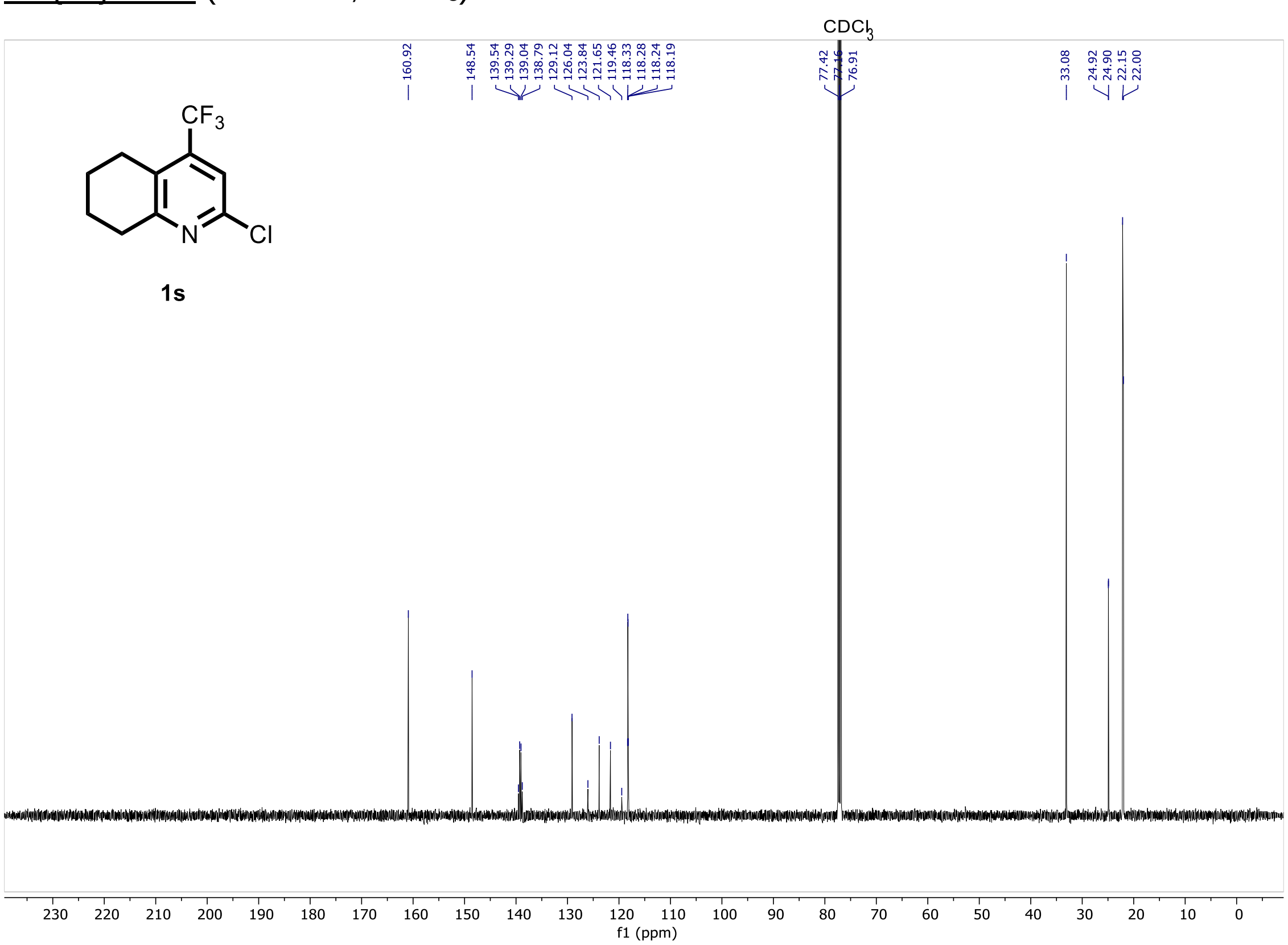

^{1}H NMR (400 MHz, $CDCl_3$)

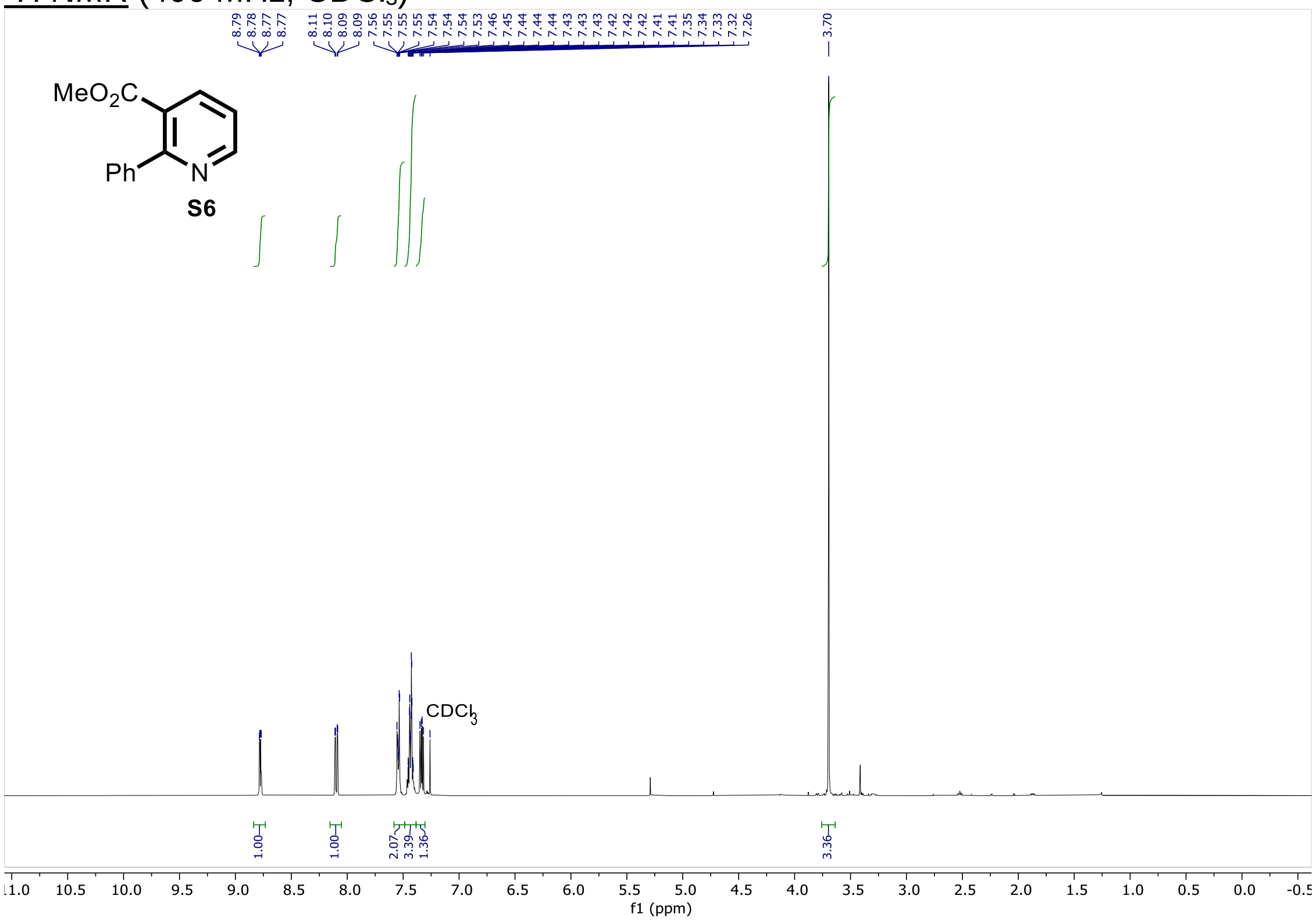

^{13}C{^{1}H} NMR (101 MHz, $CDCl_3$)

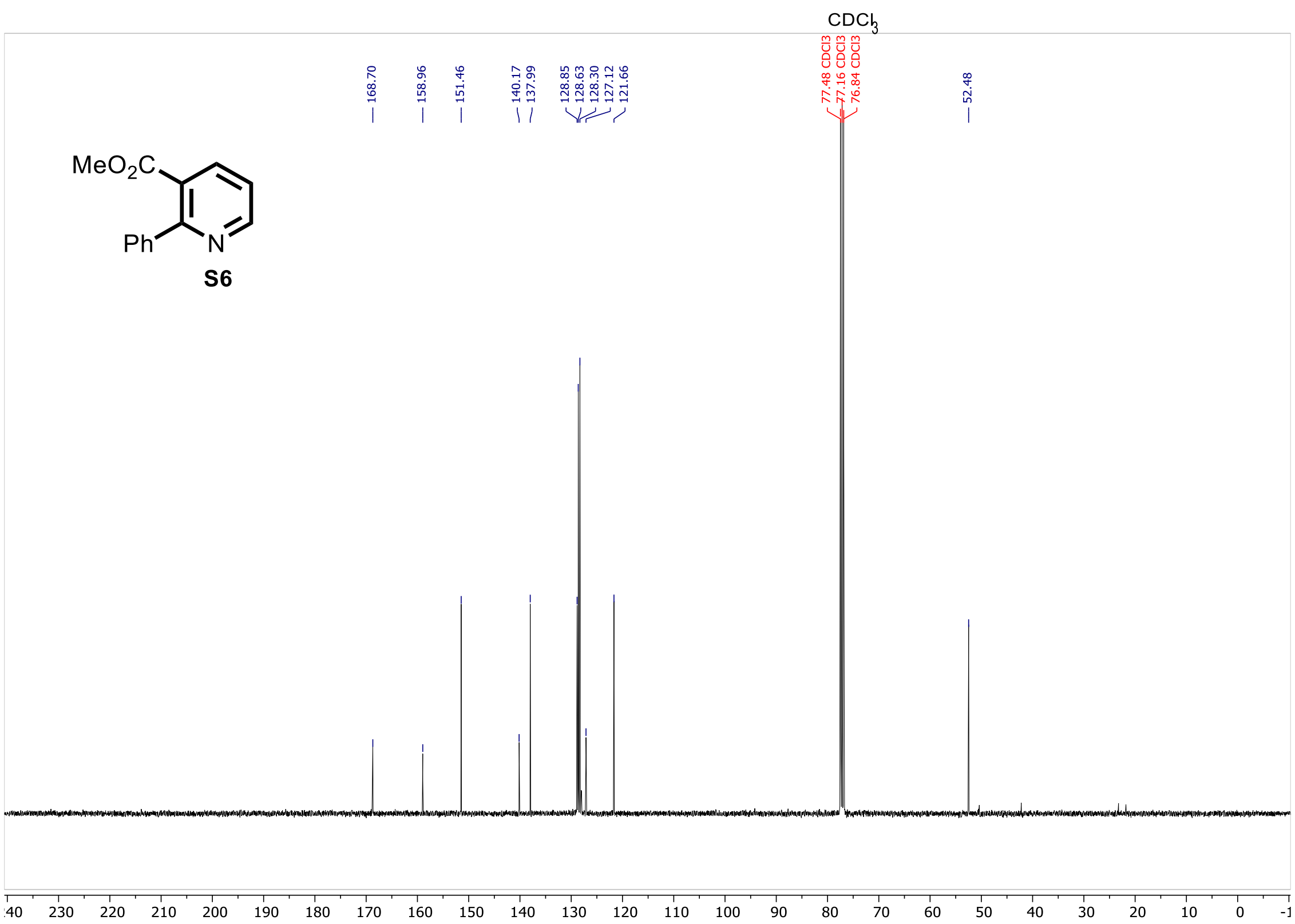

^{1}H NMR (400 MHz, $CDCl_3$)

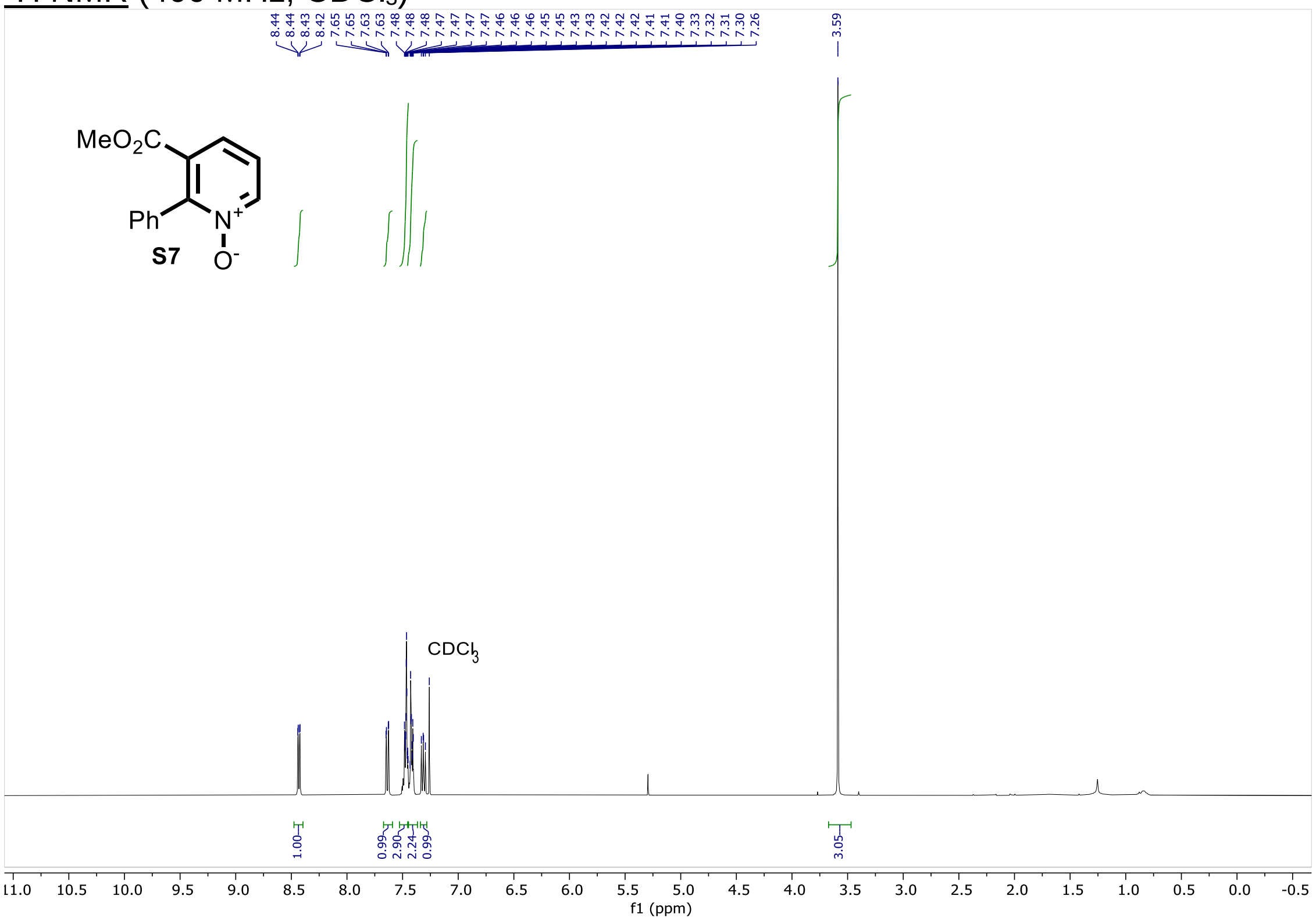

$^{13}C\{^{1}H\}$ NMR (101 MHz, $CDCl_3$)

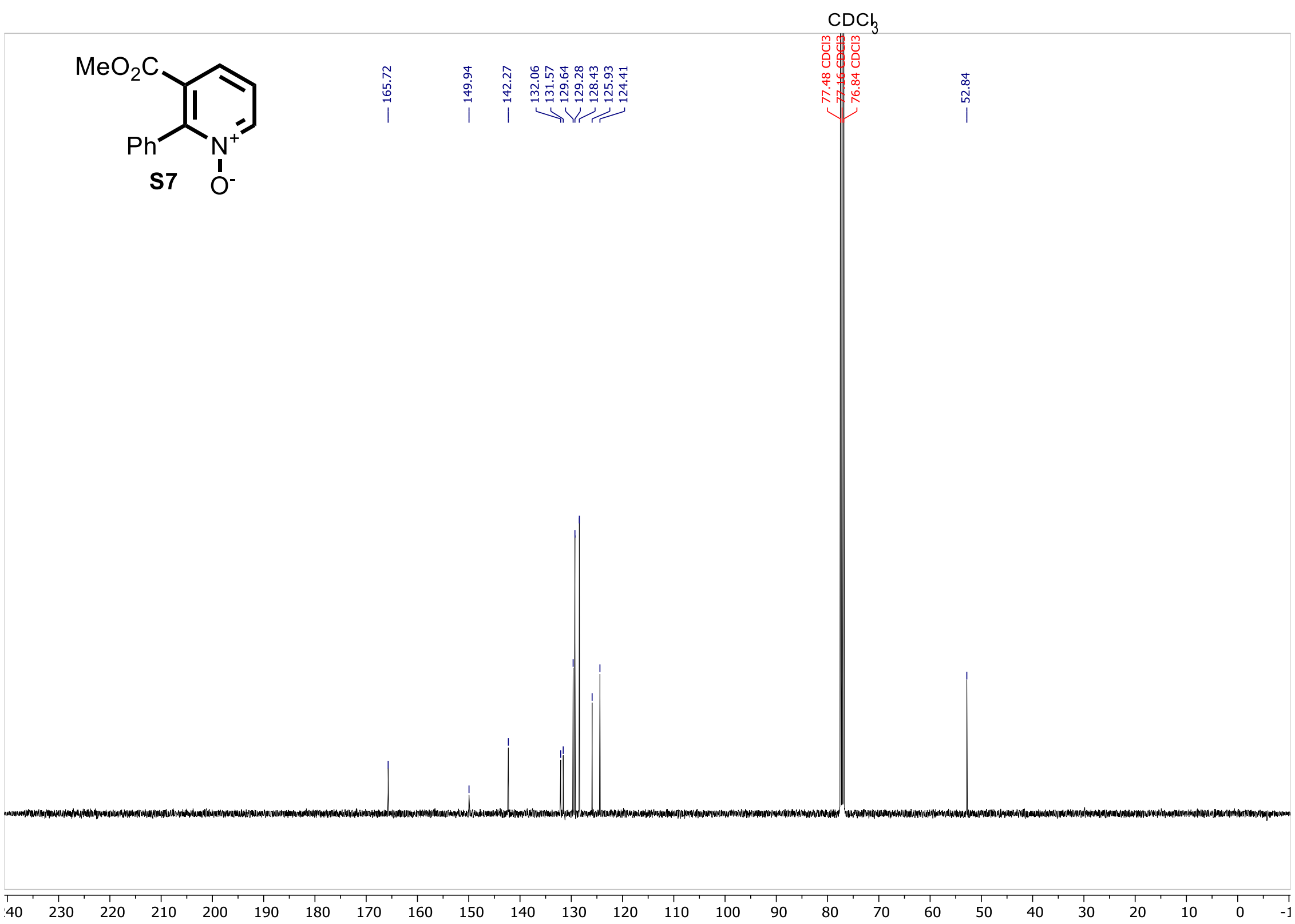

^{1}H NMR (400 MHz, $CDCl_3$)

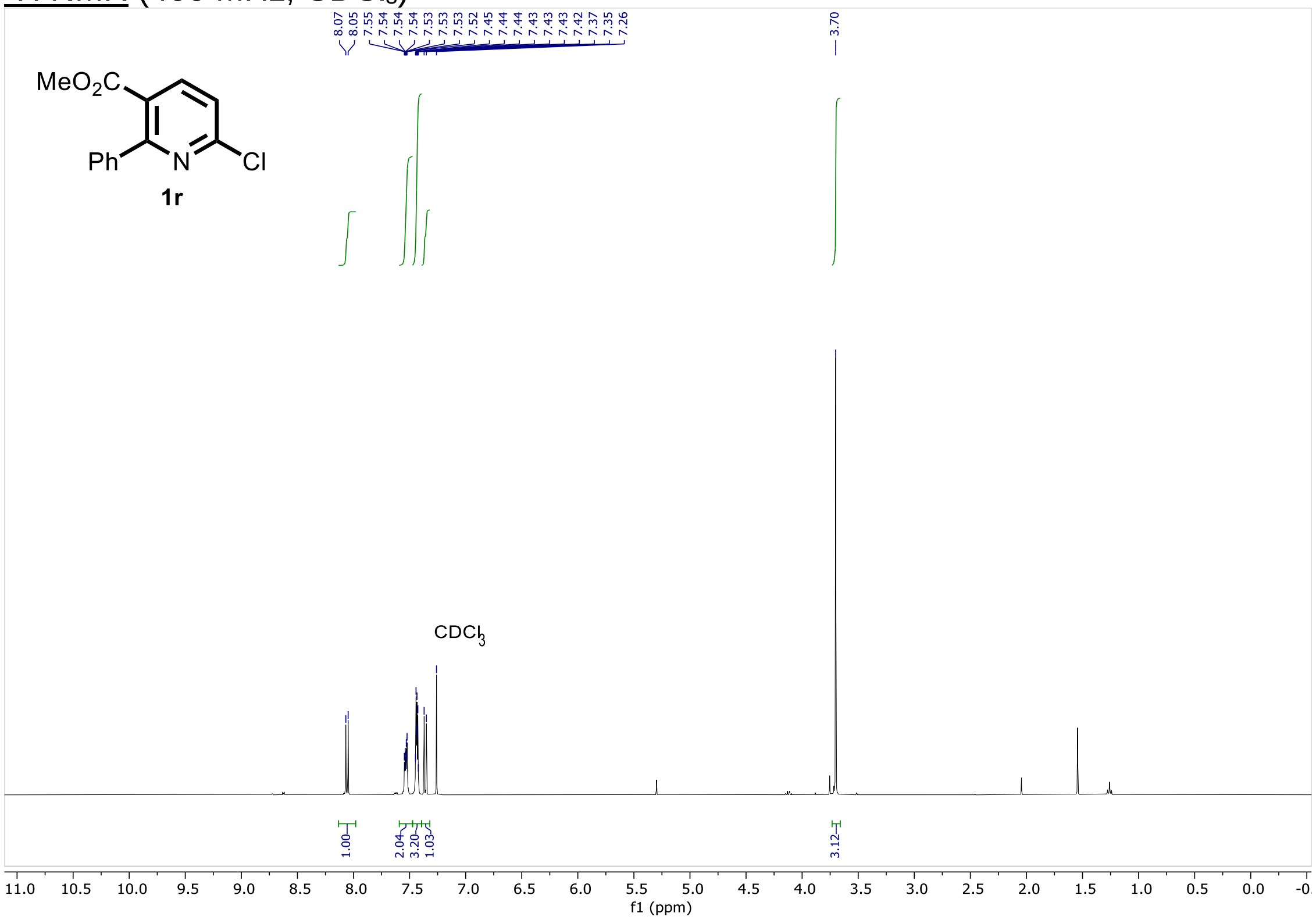

$^{13}C\{^{1}H\}$ NMR (101 MHz, $CDCl_3$)

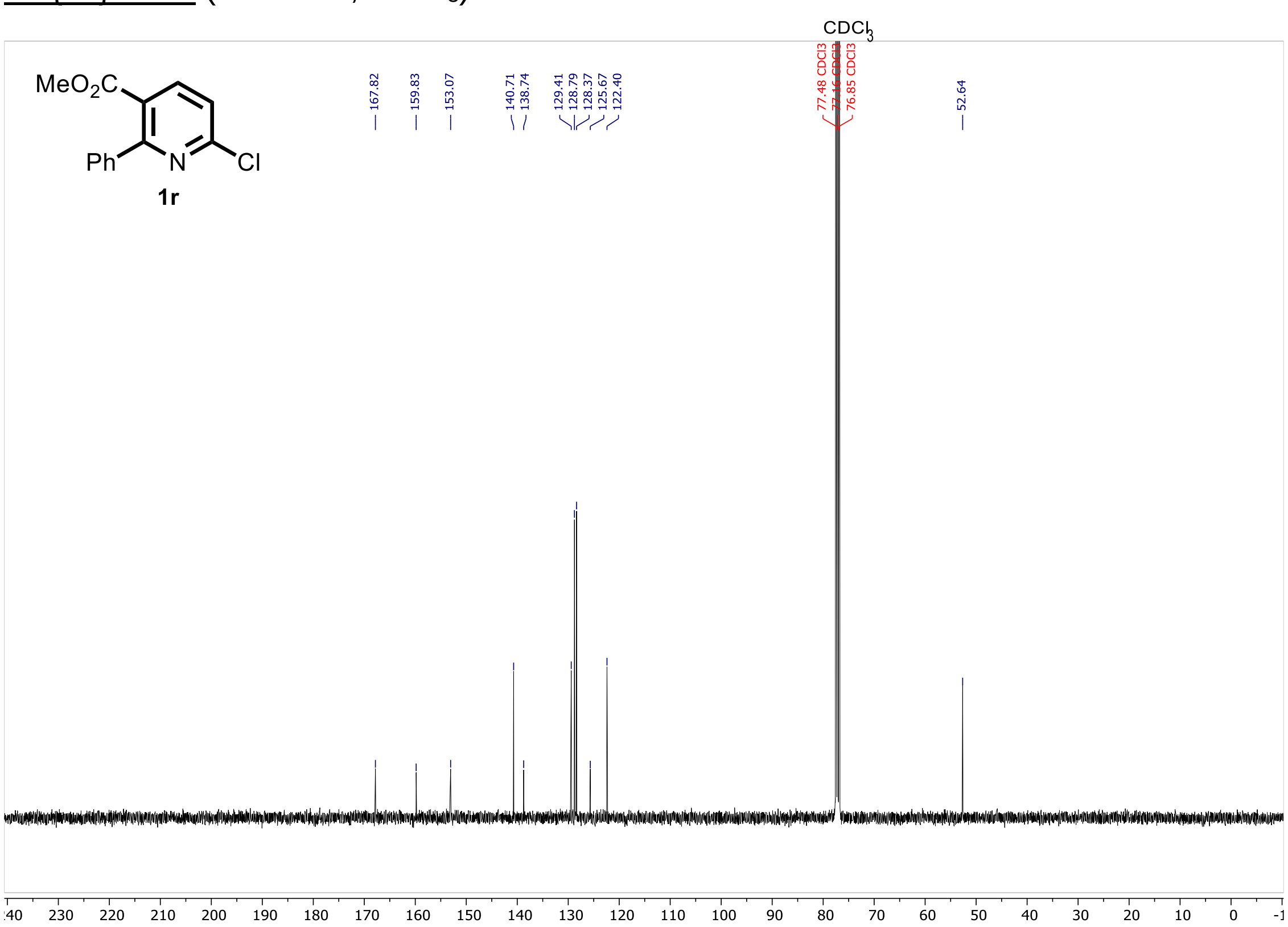

^{1}H NMR (400 MHz, $CDCl_3$)

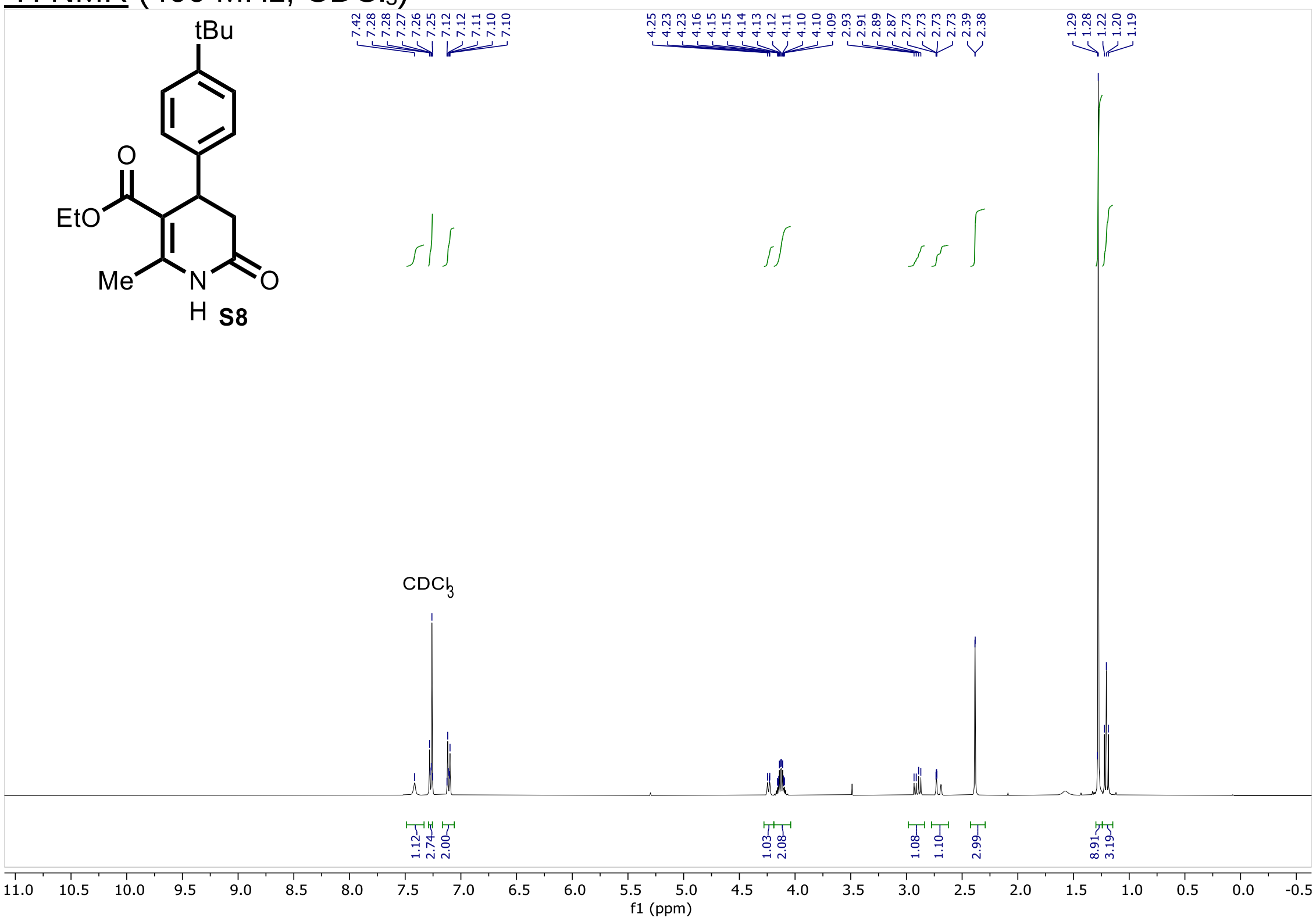

$^{13}C\{^{1}H\}$ NMR (101 MHz, $CDCl_3$)

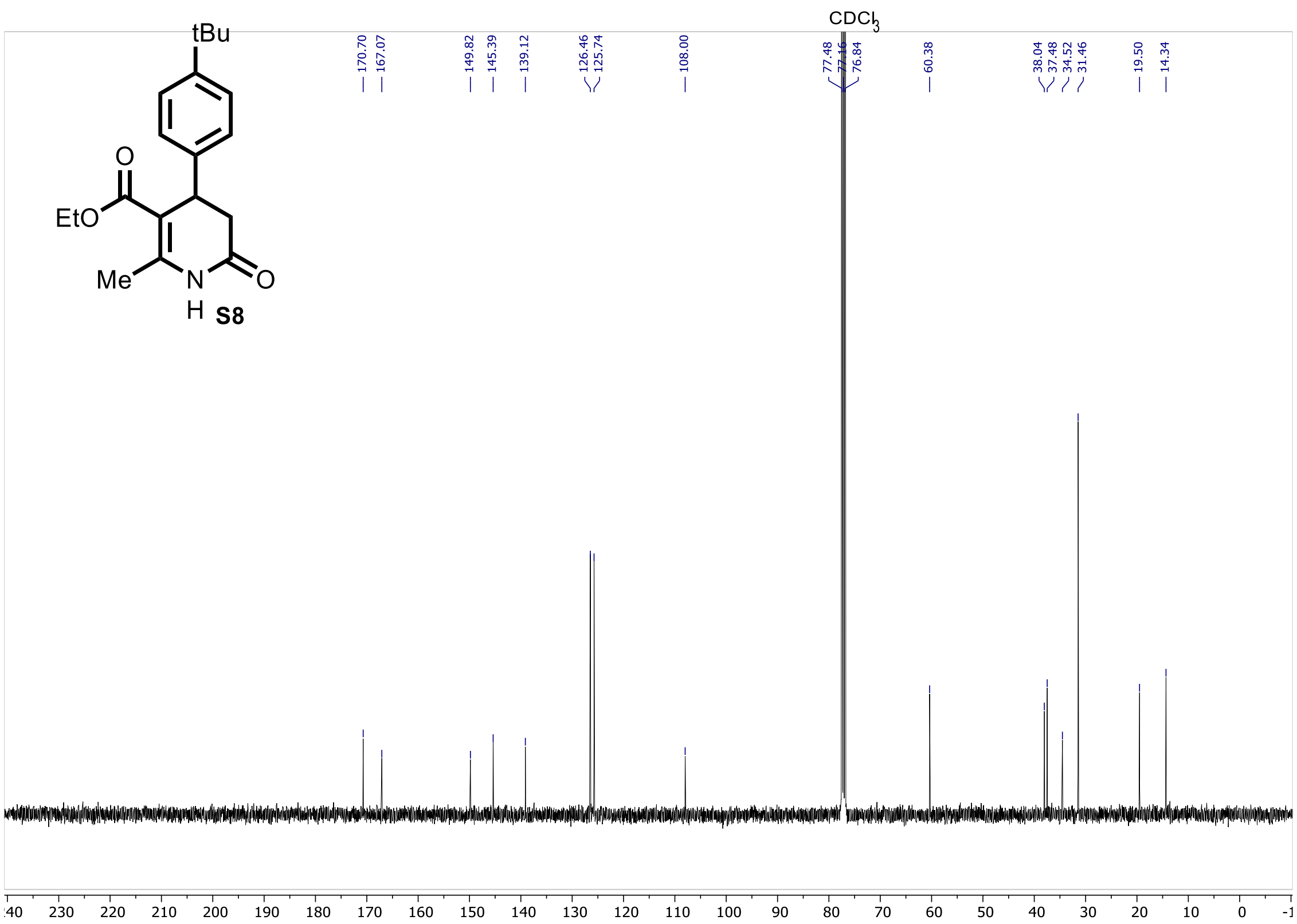

^{1}H NMR (400 MHz, $CDCl_3$)

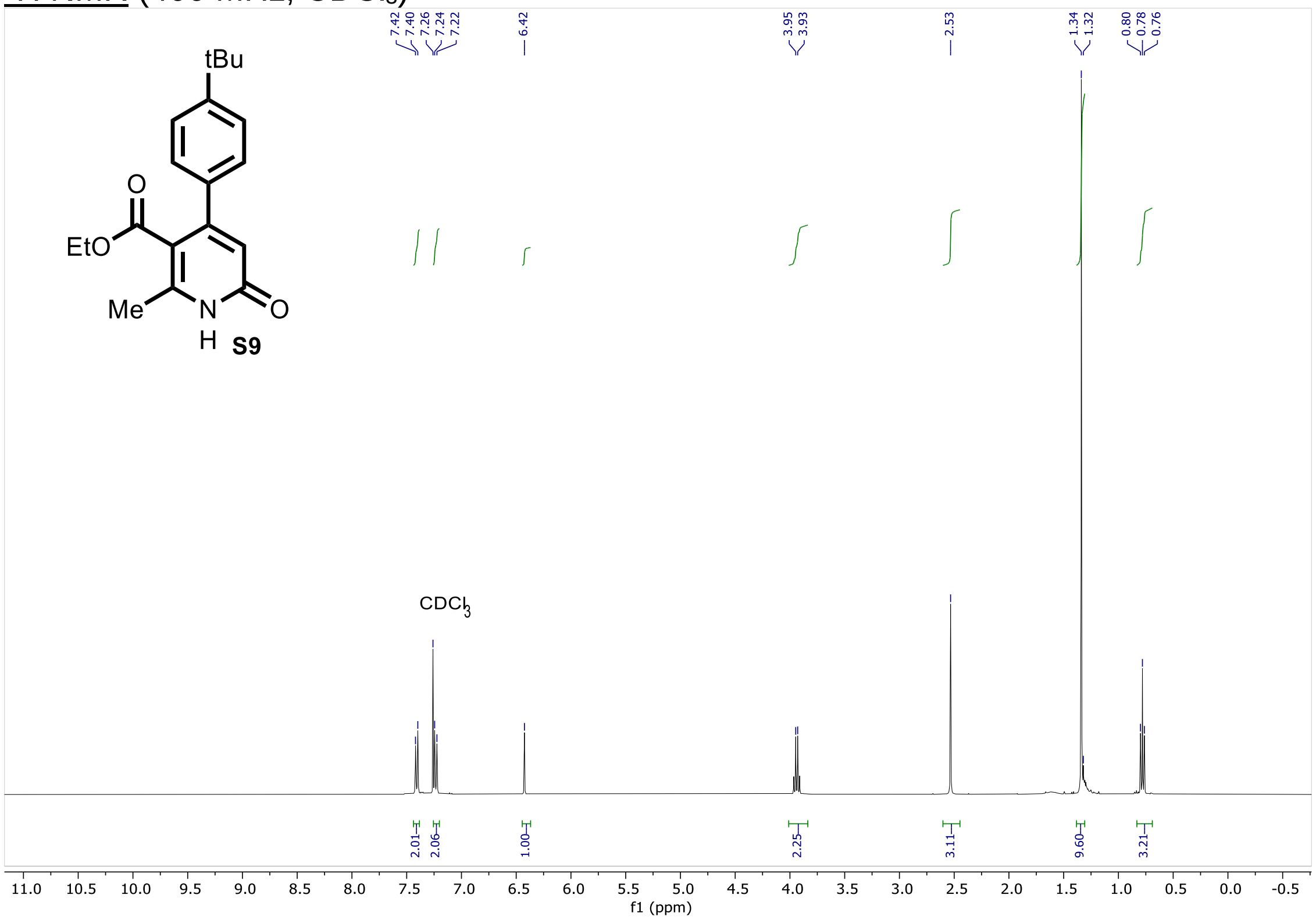

$^{13}C\{^{1}H\}$ NMR (101 MHz, $CDCl_3$)

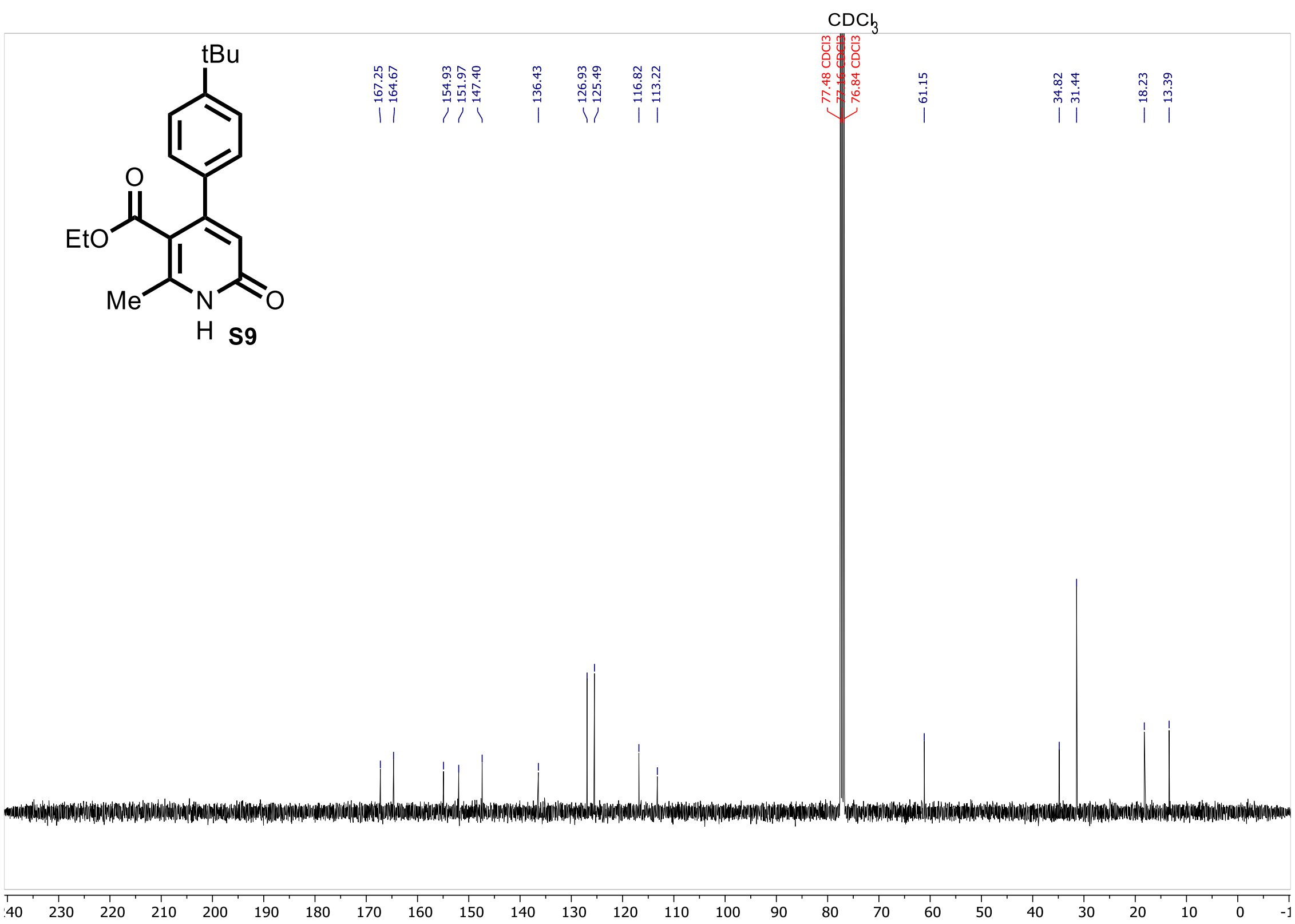

^{1}H NMR (400 MHz, $CDCl_3$)

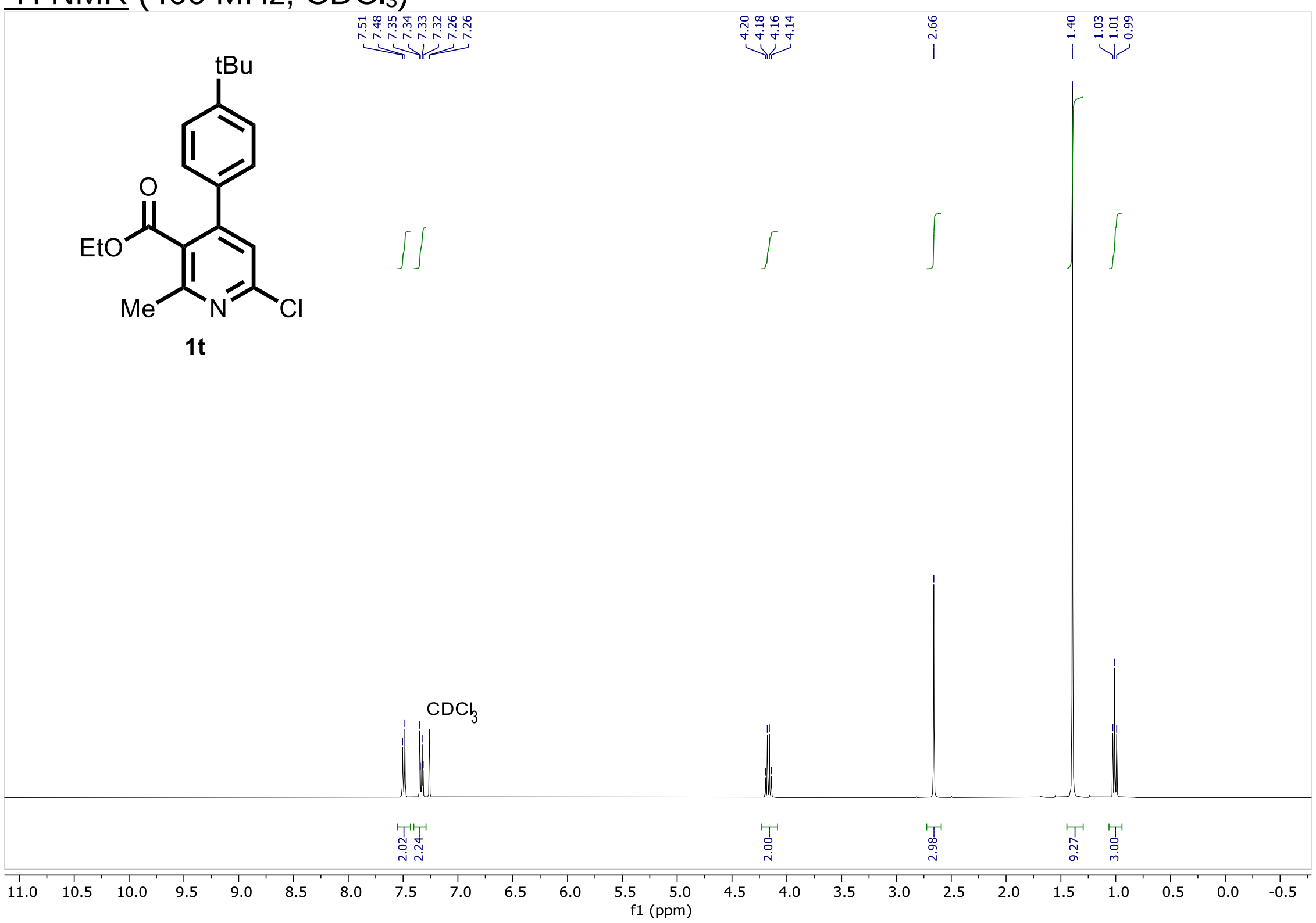

$^{13}C\{^{1}H\}$ NMR (101 MHz, $CDCl_3$)

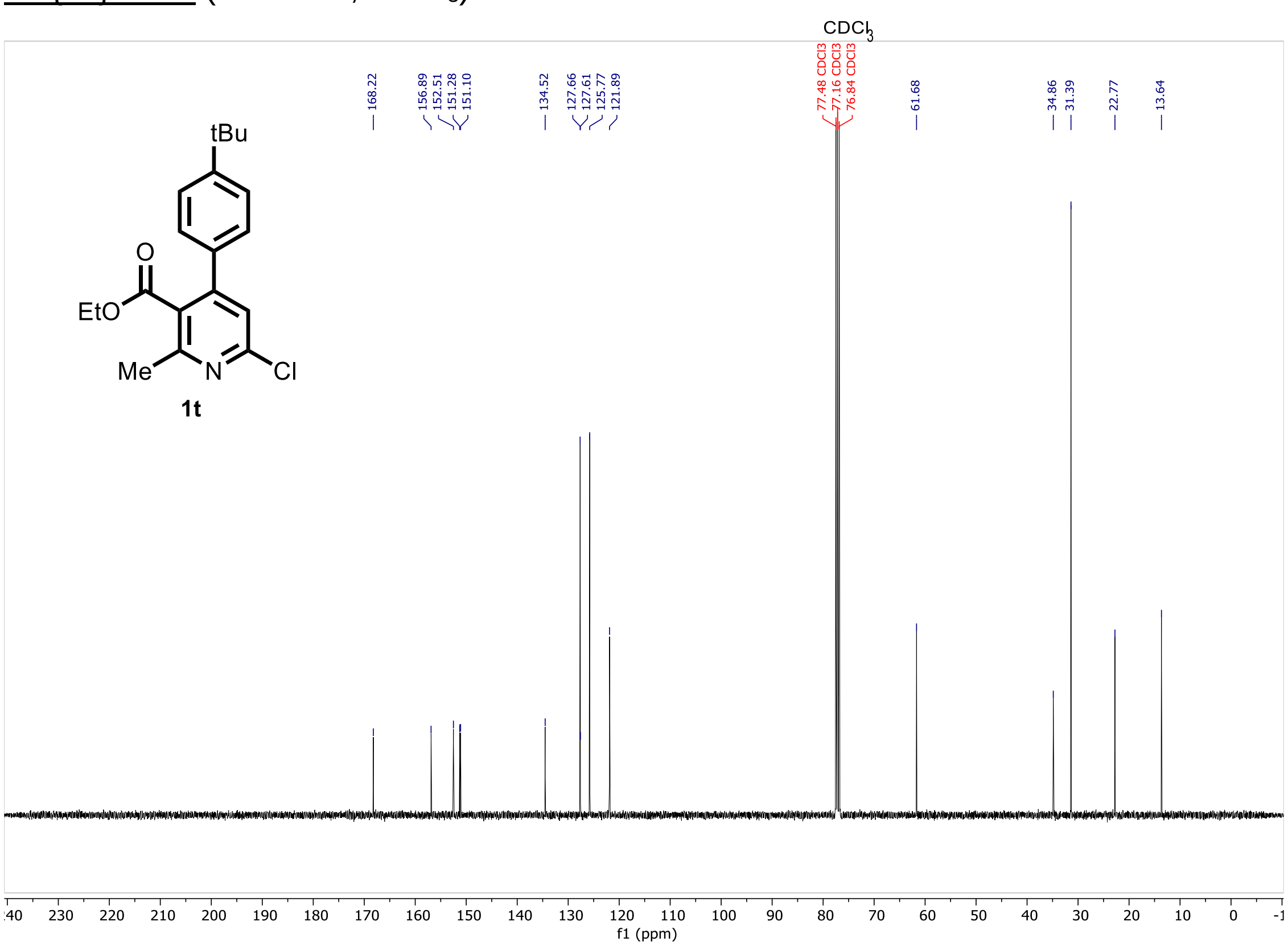

^{1}H NMR (500 MHz, $CDCl_3$)

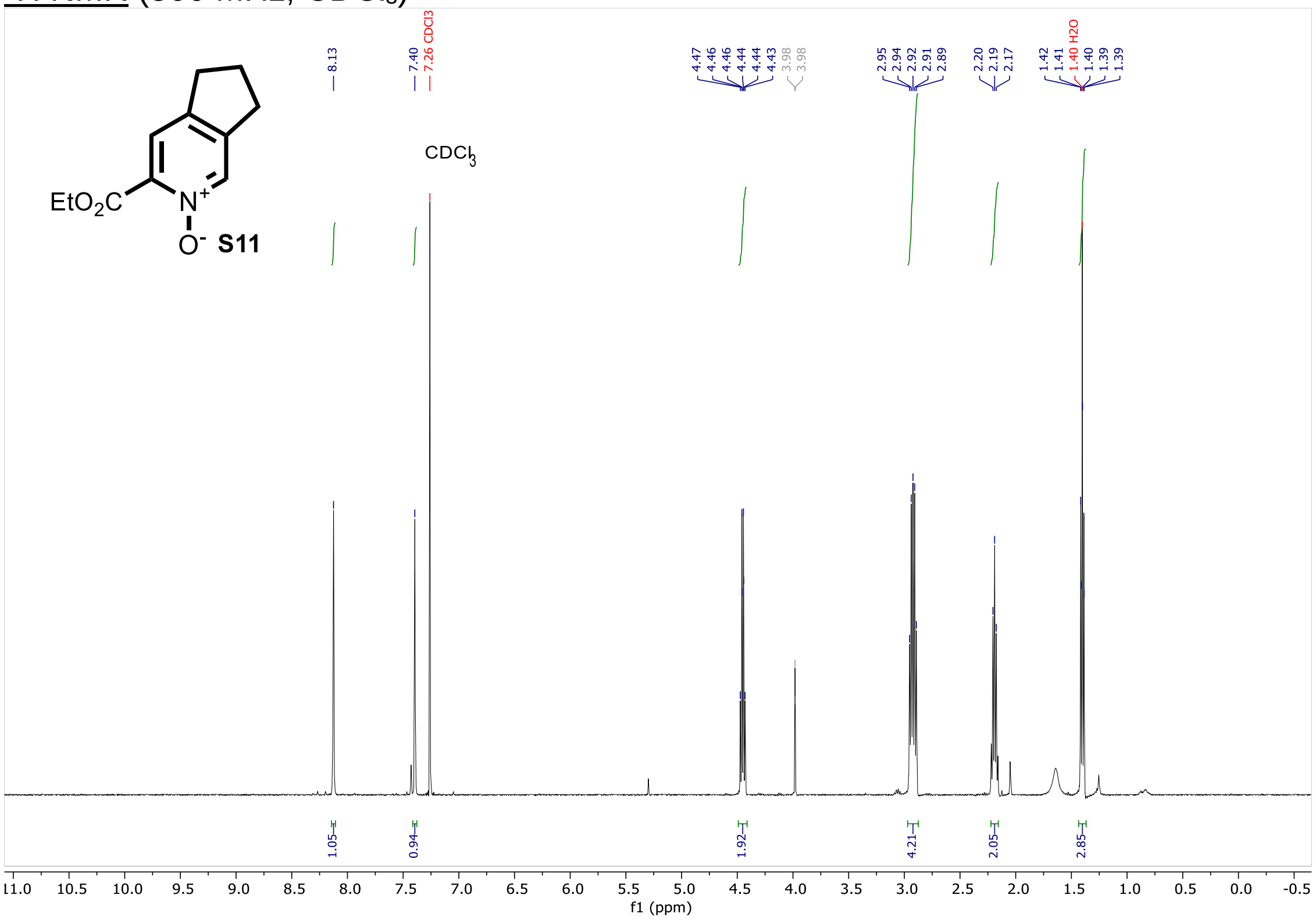

^{13}C{^{1}H} NMR (151 MHz, $CDCl_3$)

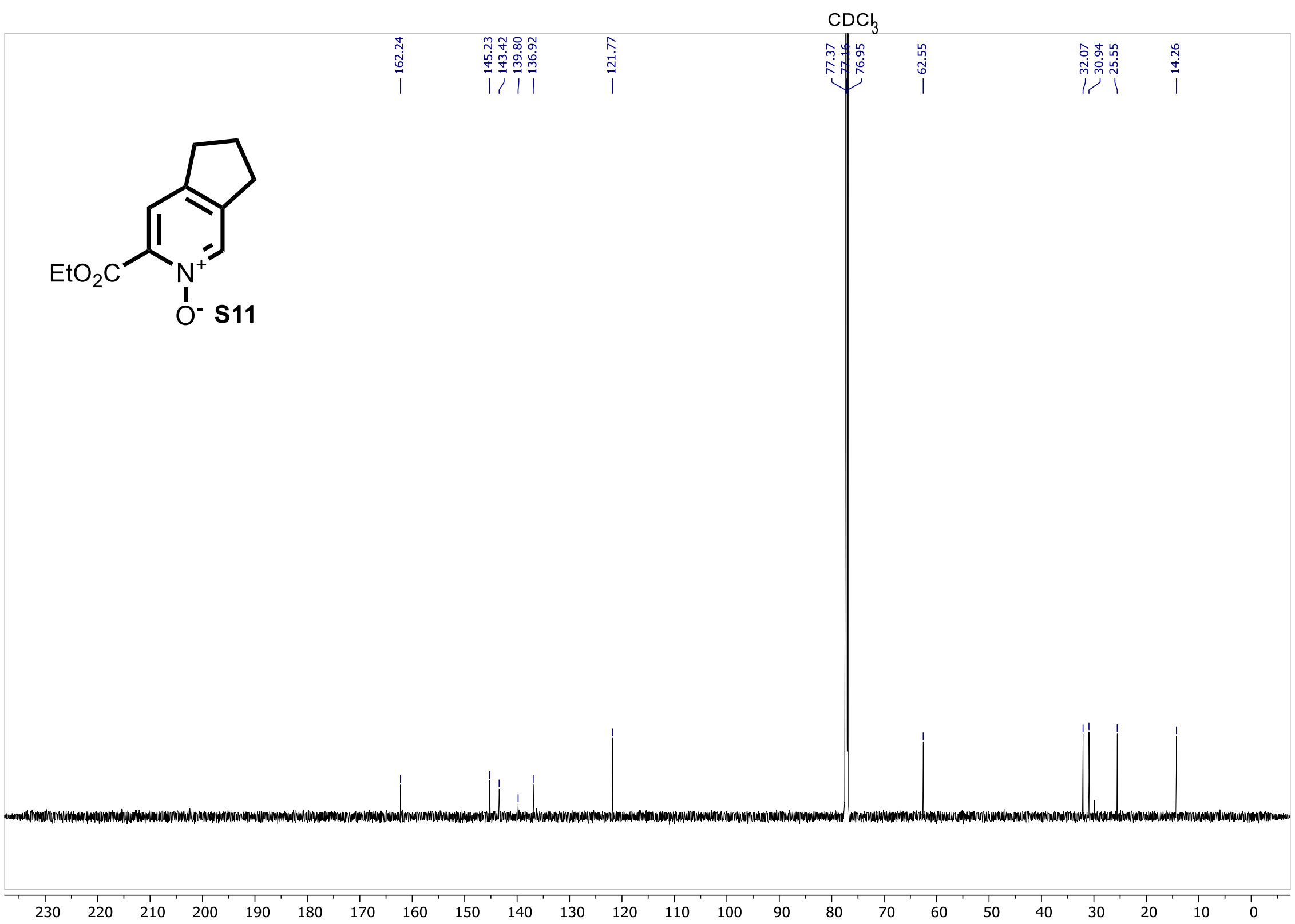

^{1}H NMR (400 MHz, $CDCl_3$)

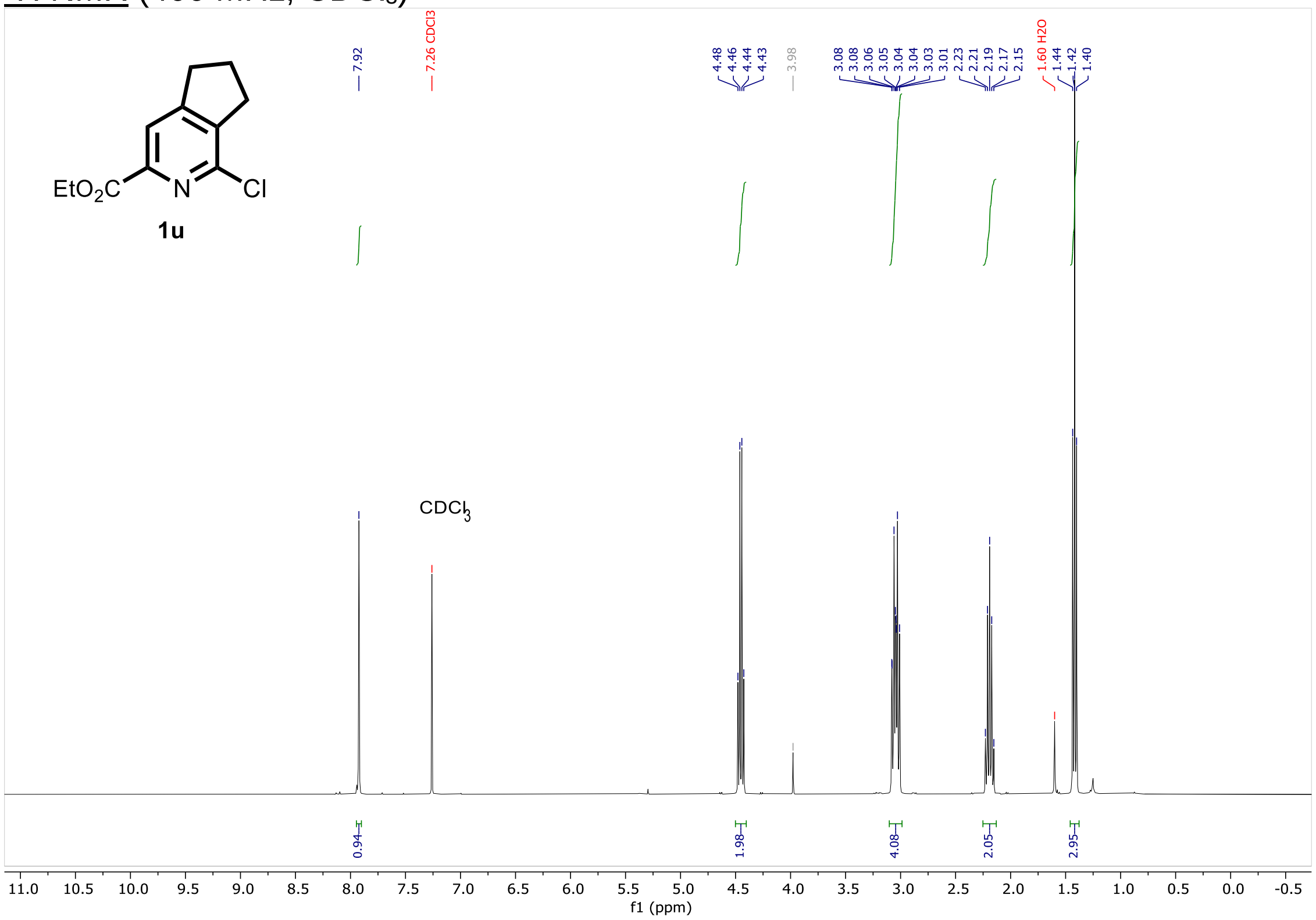

$^{13}C\{^{1}H\}$ NMR (101 MHz, $CDCl_3$)

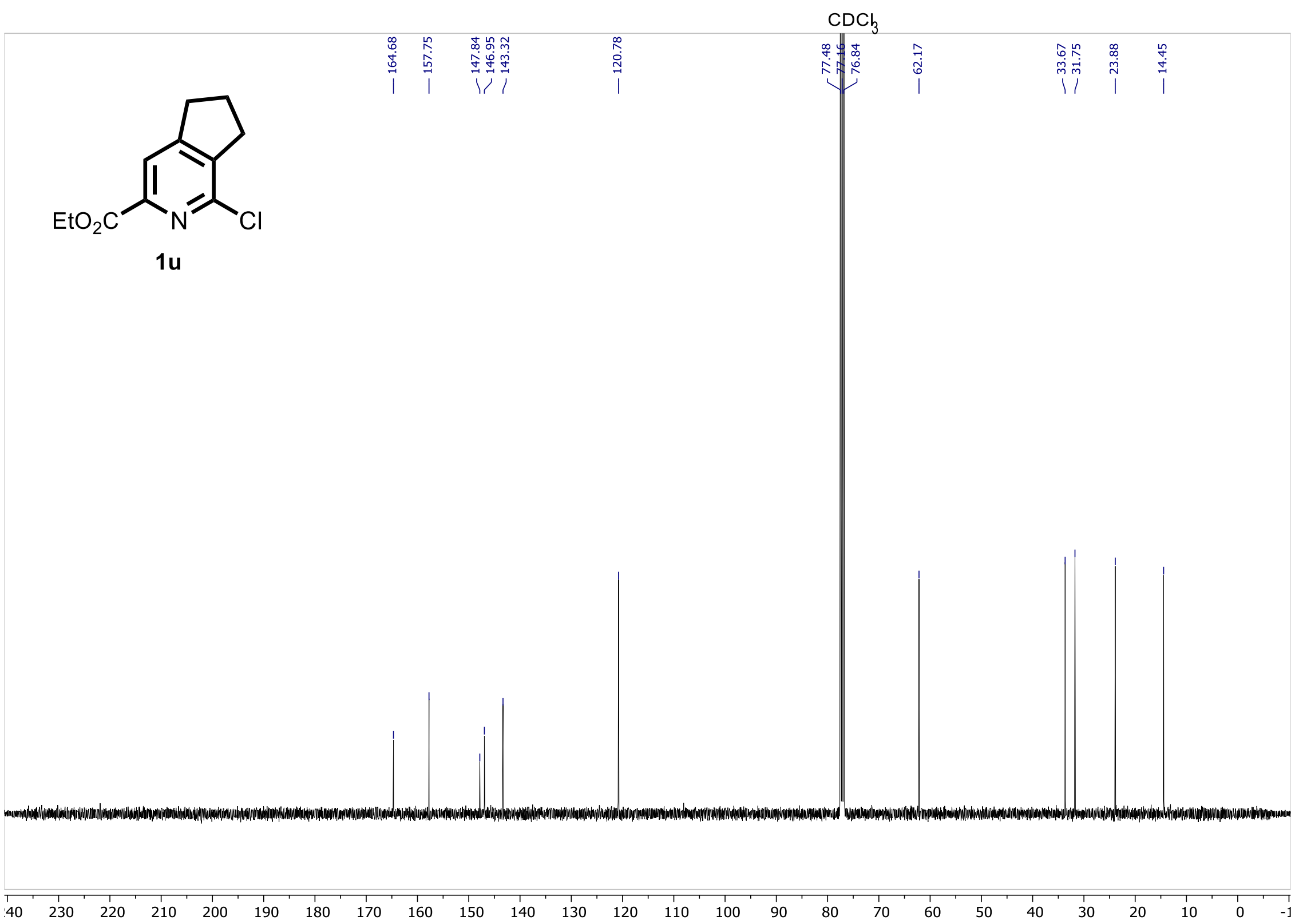

^{1}H NMR (400 MHz, $CDCl_3$)

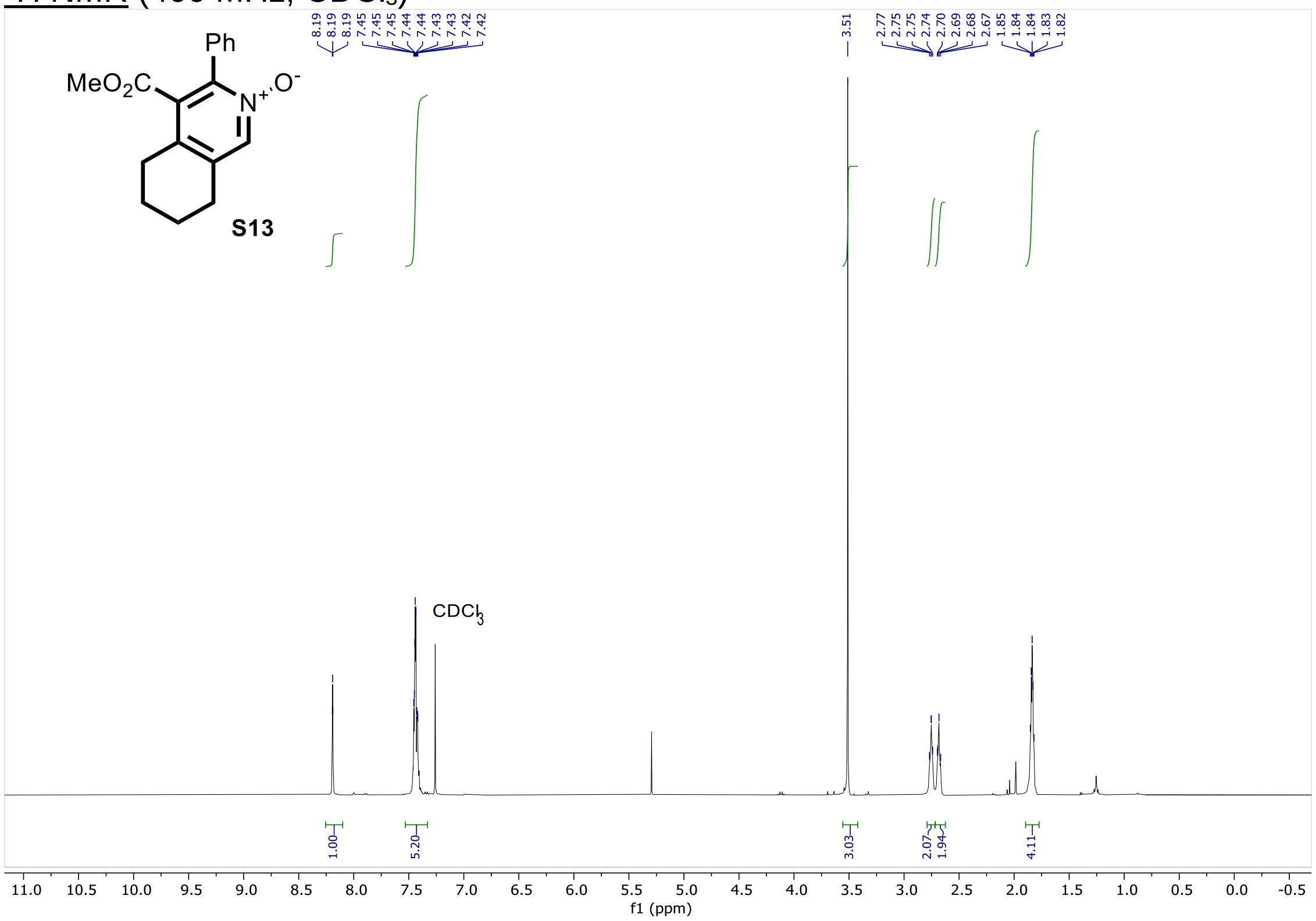

$^{13}C\{^1H\}$ NMR (101 MHz, $CDCl_3$)

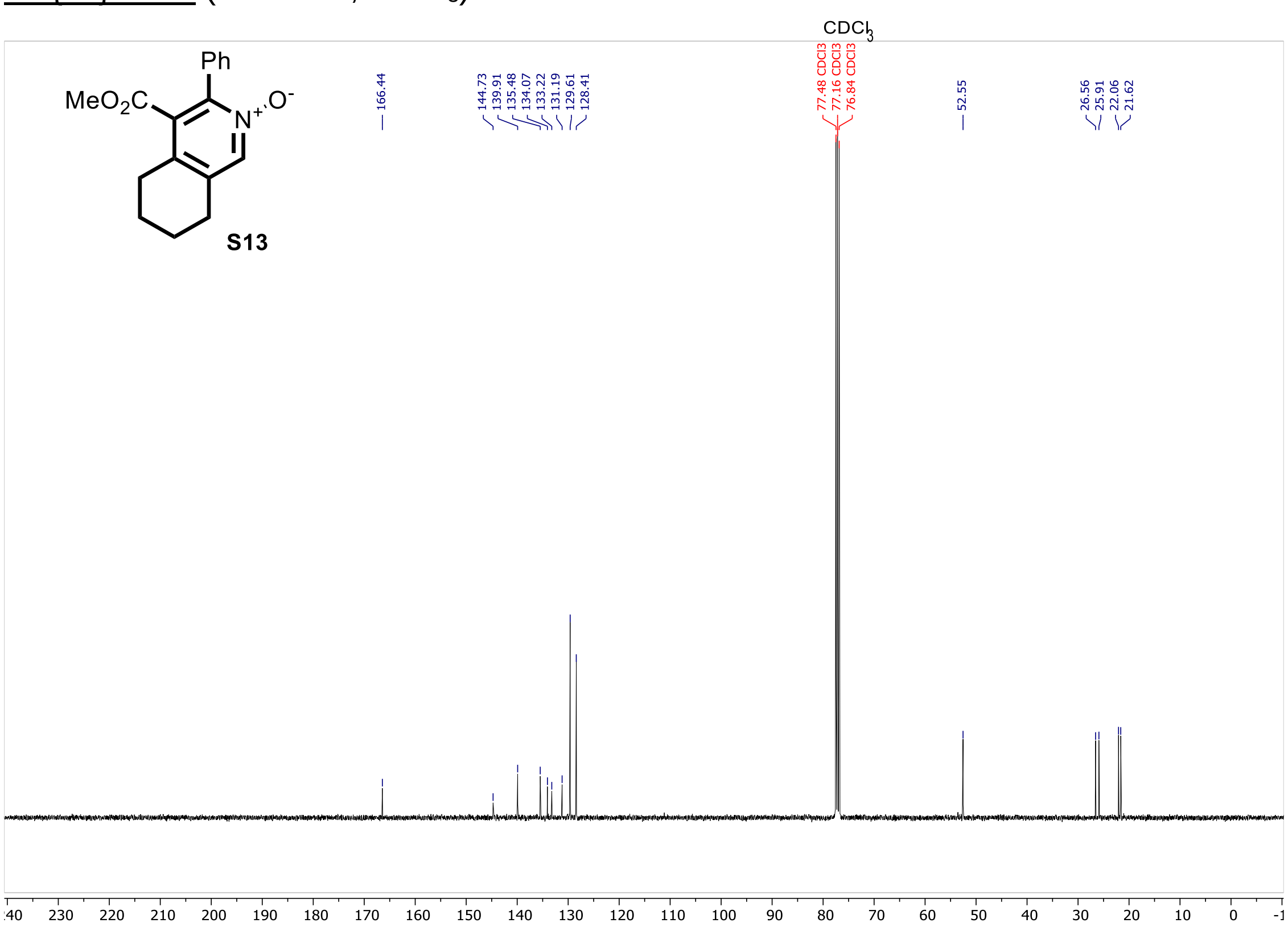

^{1}H NMR (400 MHz, $CDCl_3$)

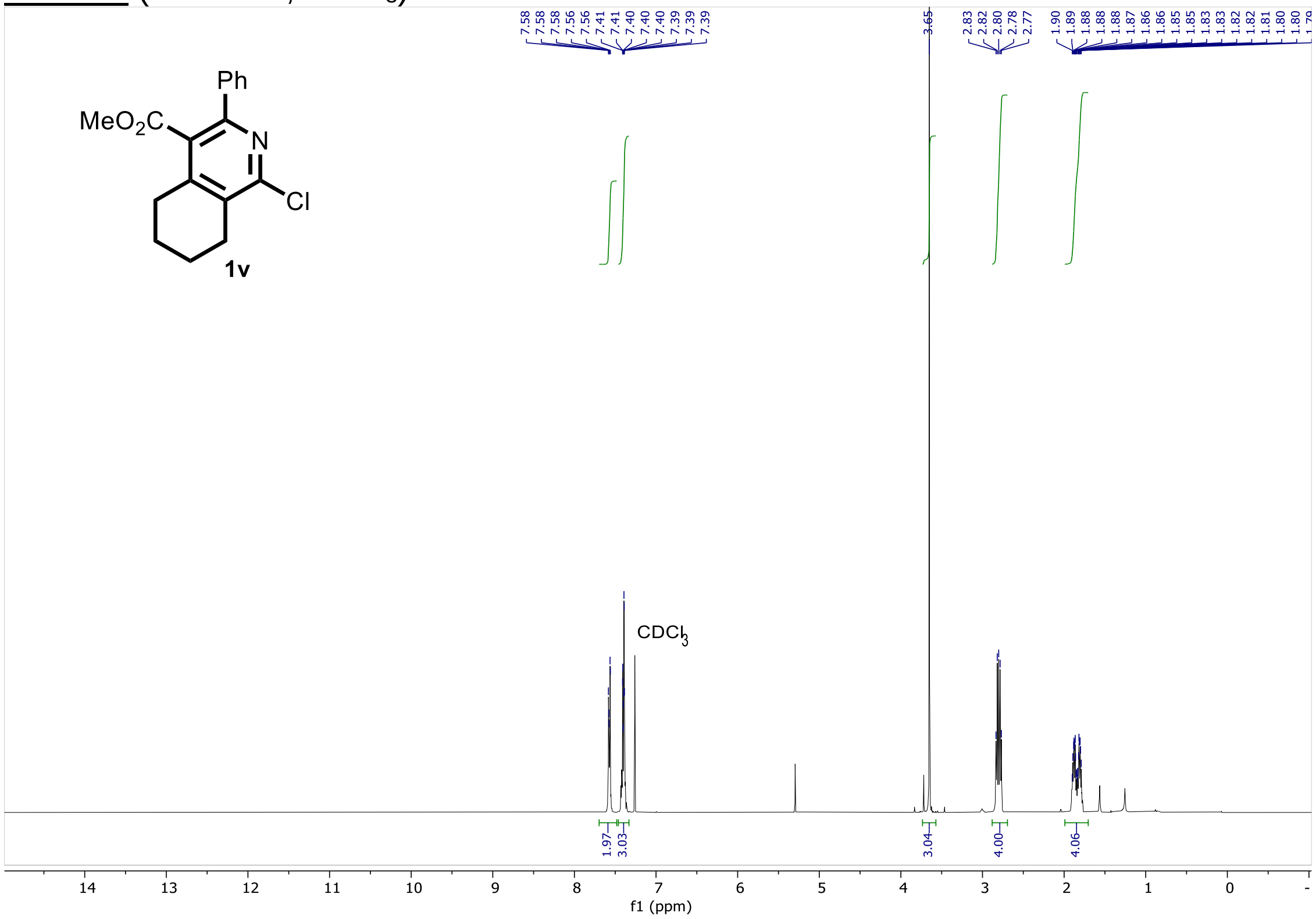

^{13}C{^{1}H} NMR (101 MHz, $CDCl_3$)

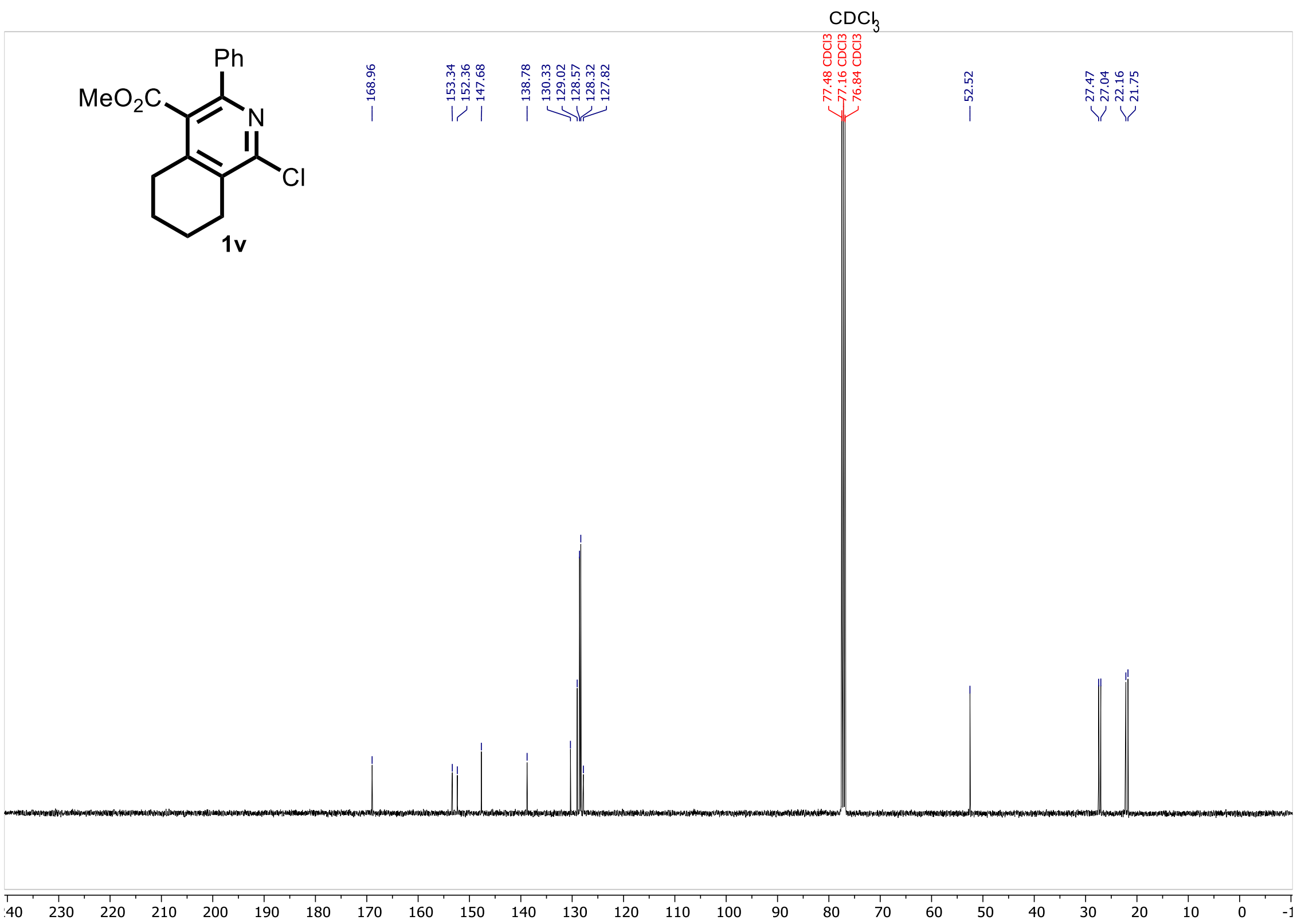

^{1}H NMR (400 MHz, $CDCl_3$)

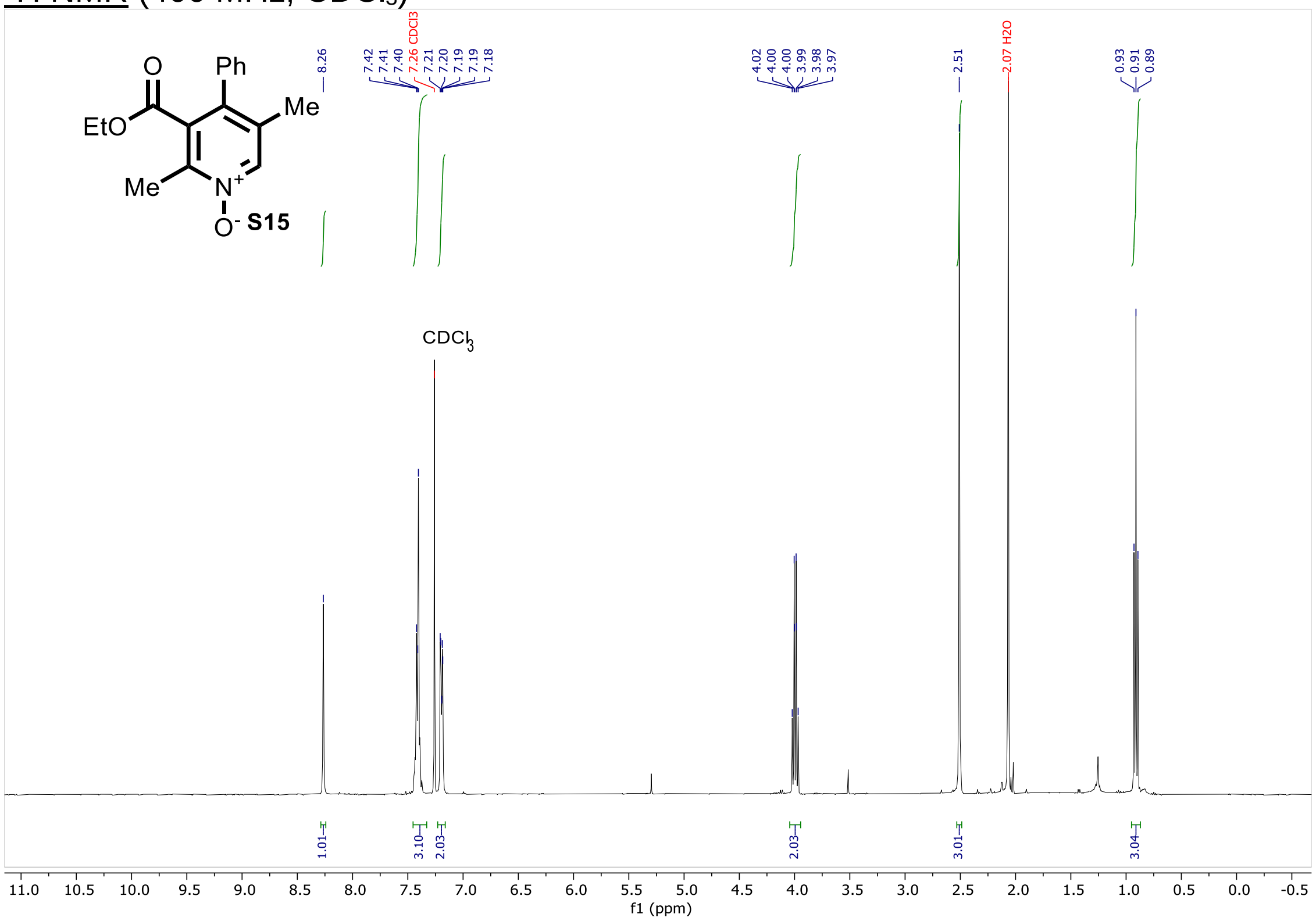

^{13}C{^{1}H} NMR (101 MHz, $CDCl_3$)

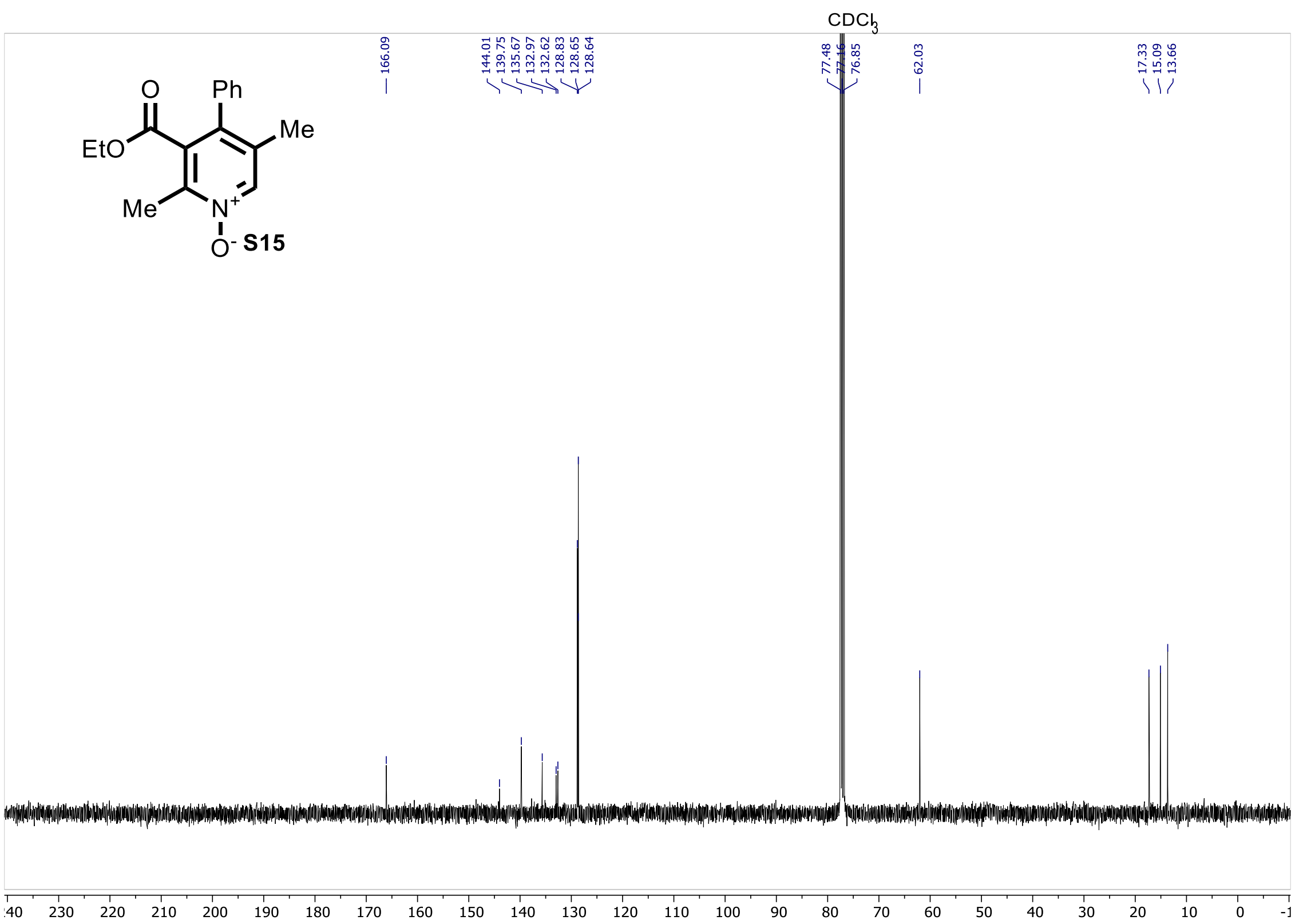

^{1}H NMR (400 MHz, $CDCl_3$)

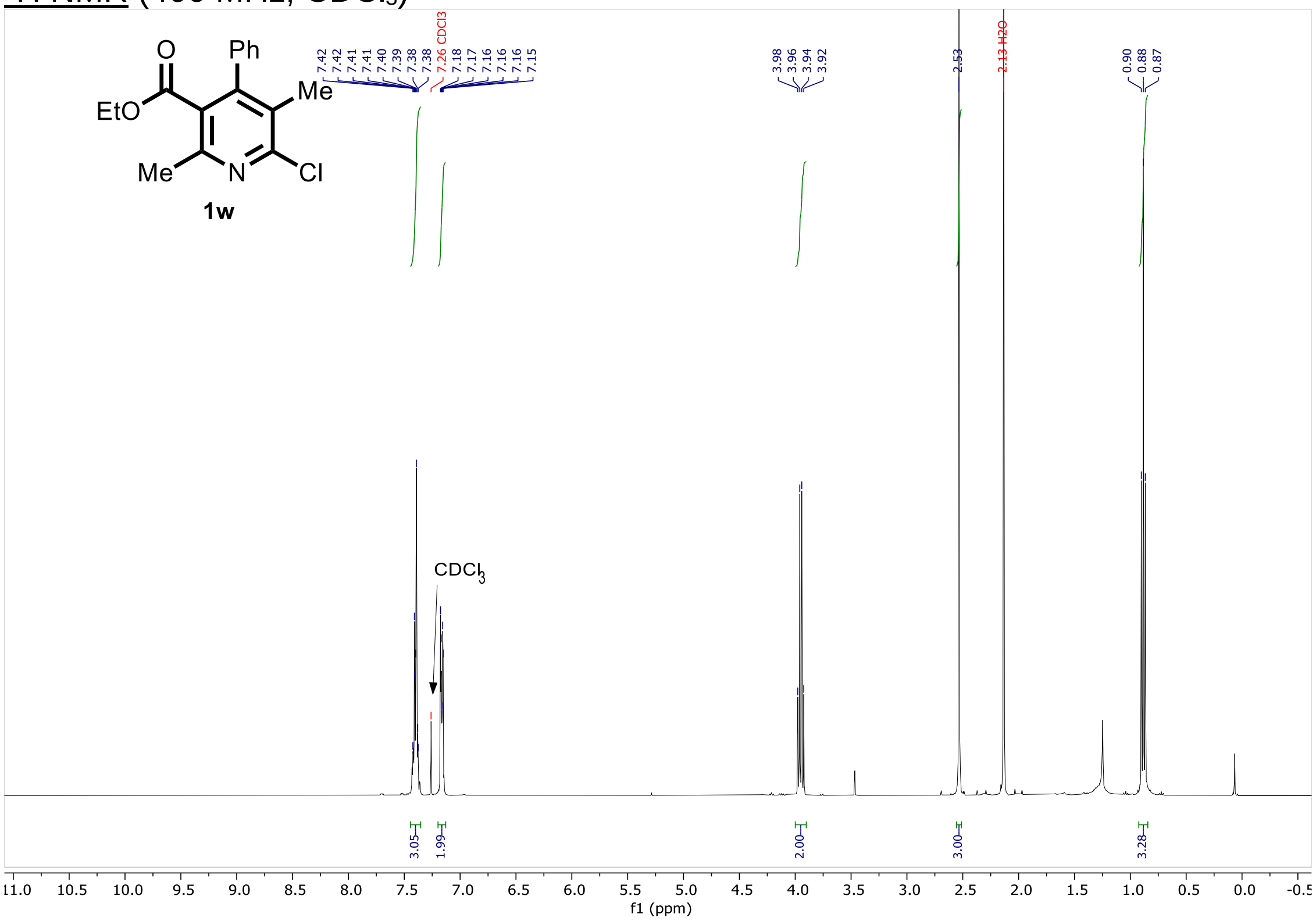

$^{13}C\{^{1}H\}$ NMR (101 MHz, $CDCl_3$)

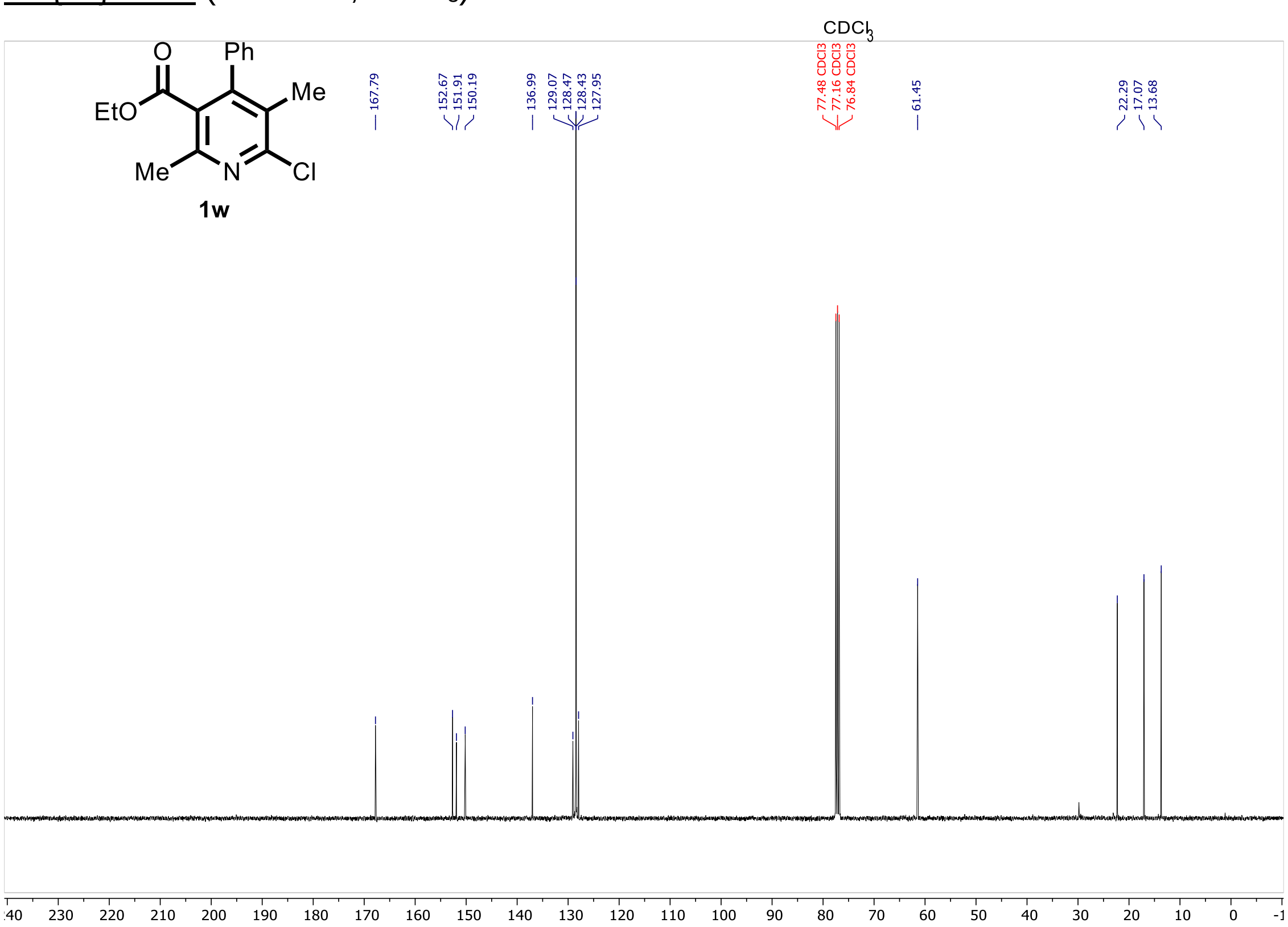

^{1}H NMR (400 MHz, $CDCl_3$)

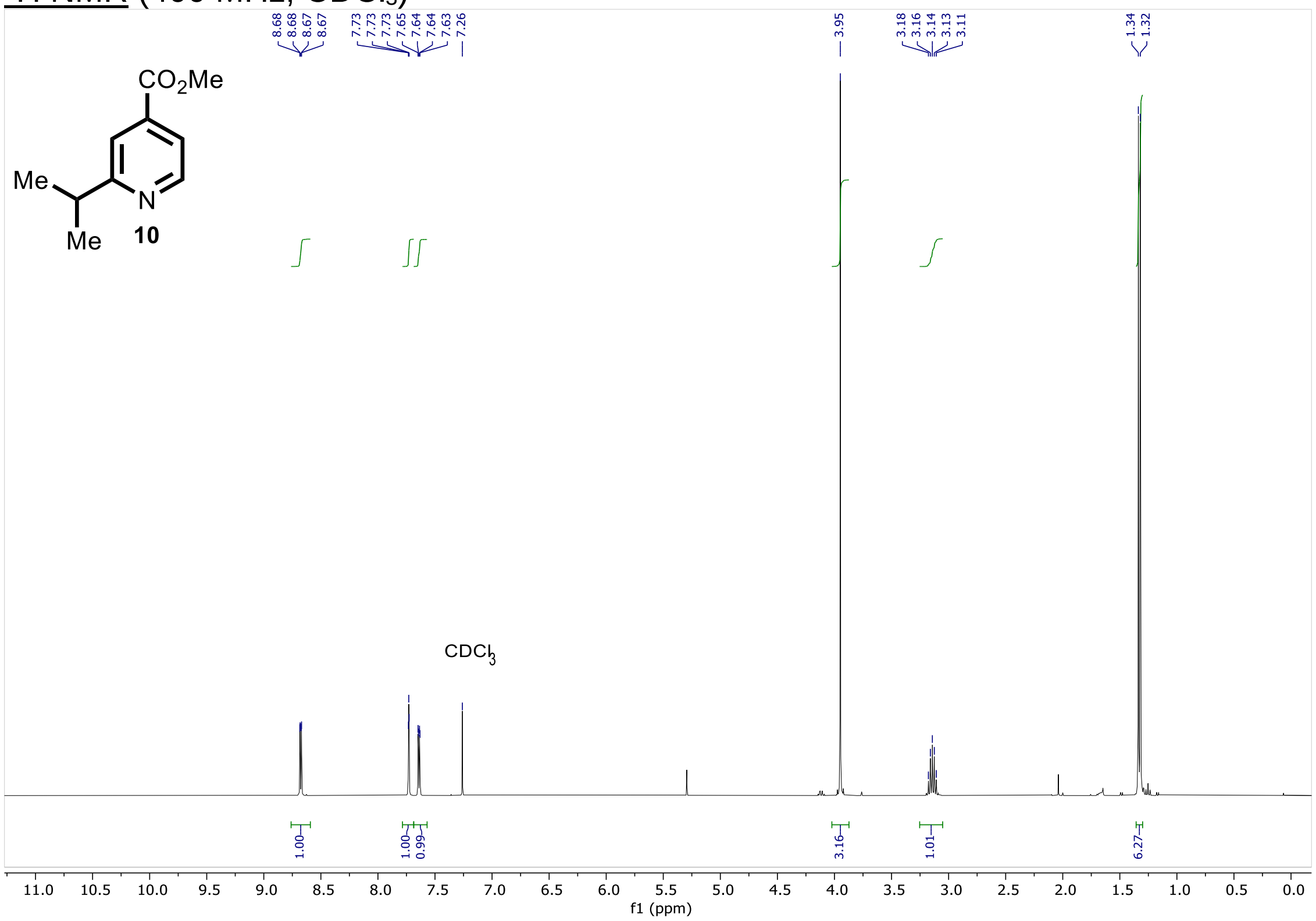

$^{13}C\{^{1}H\}$ NMR (101 MHz, $CDCl_3$)

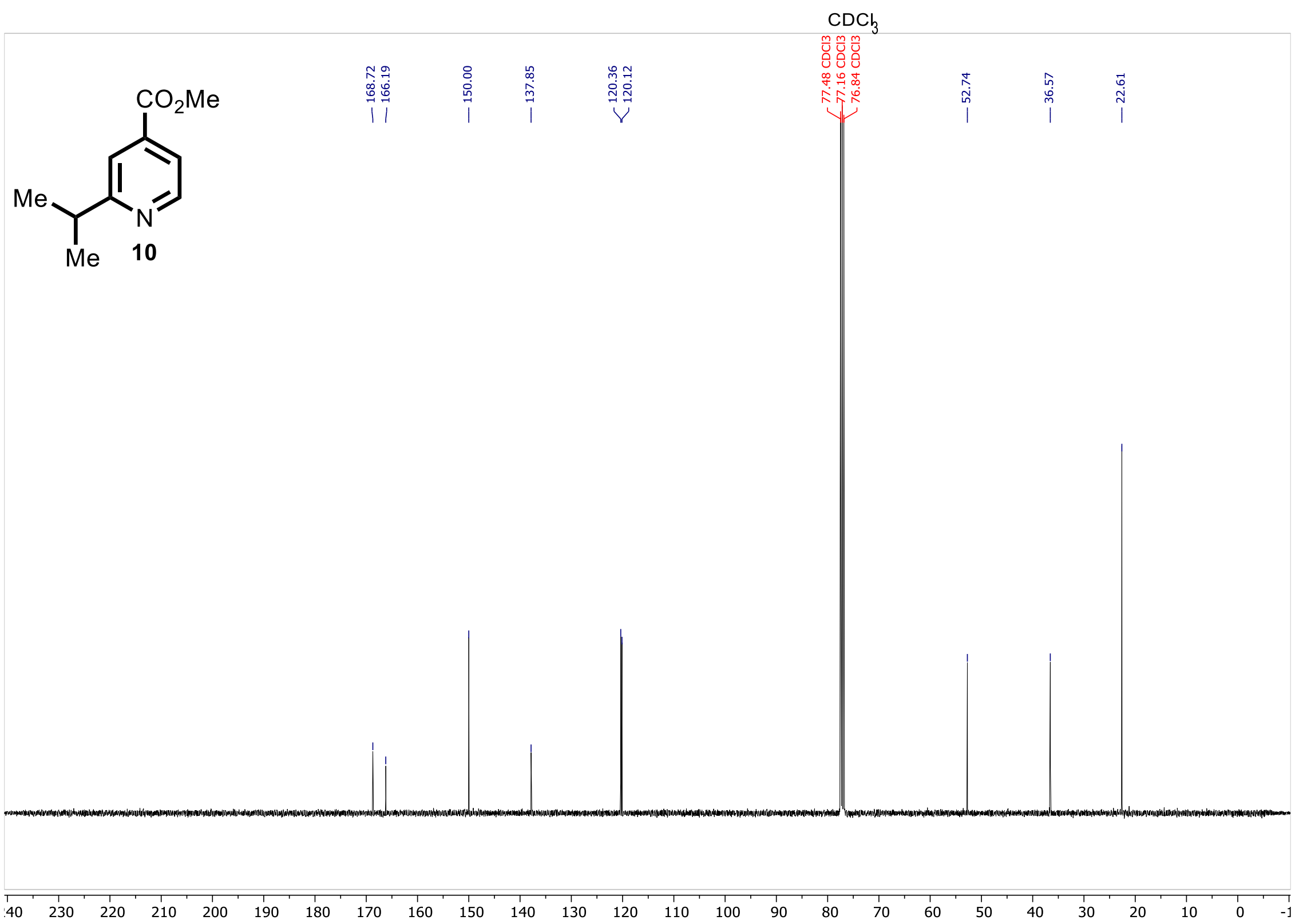

^{1}H NMR (400 MHz, $CDCl_3$)

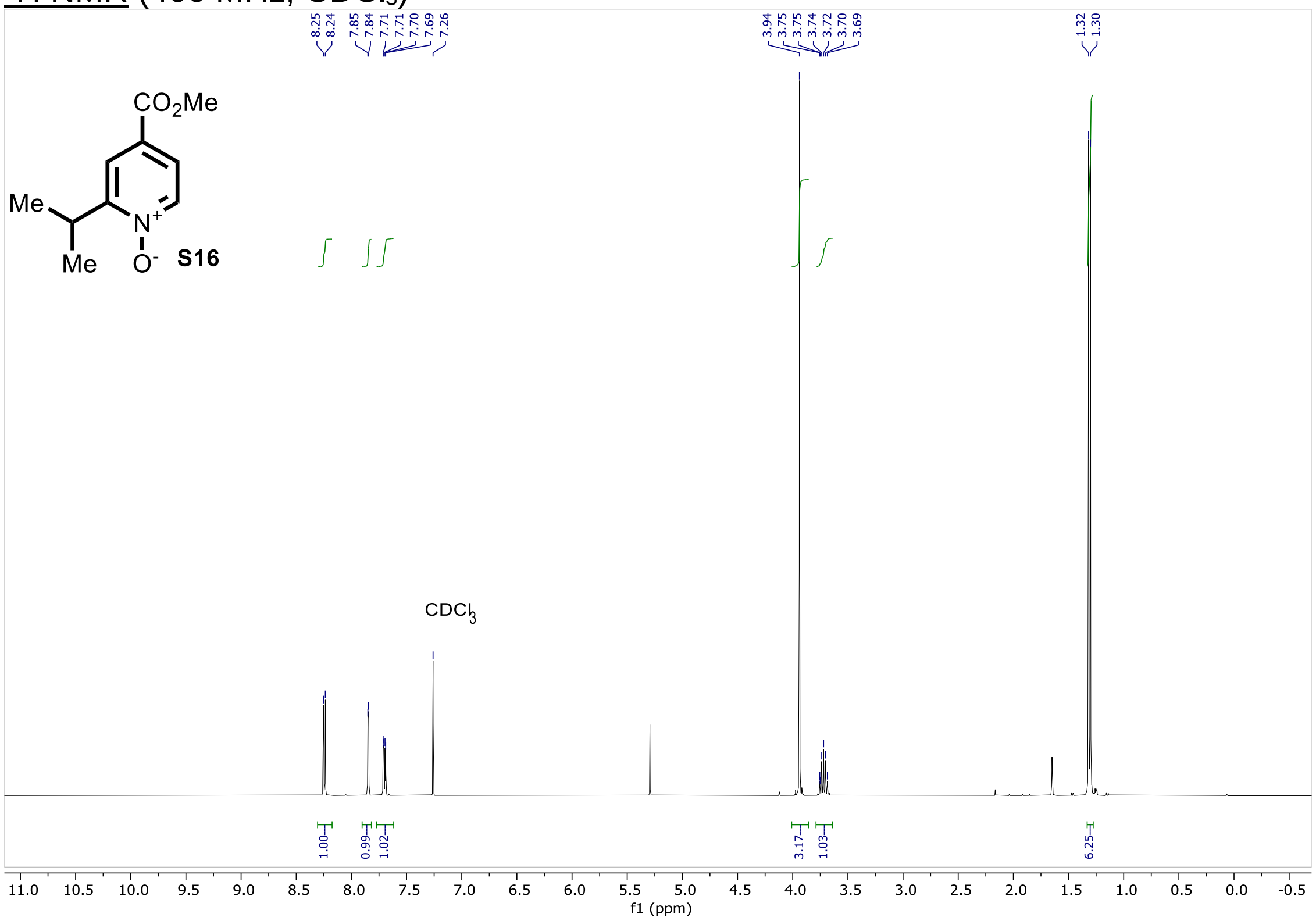

^{13}C{^{1}H} NMR (101 MHz, $CDCl_3$)

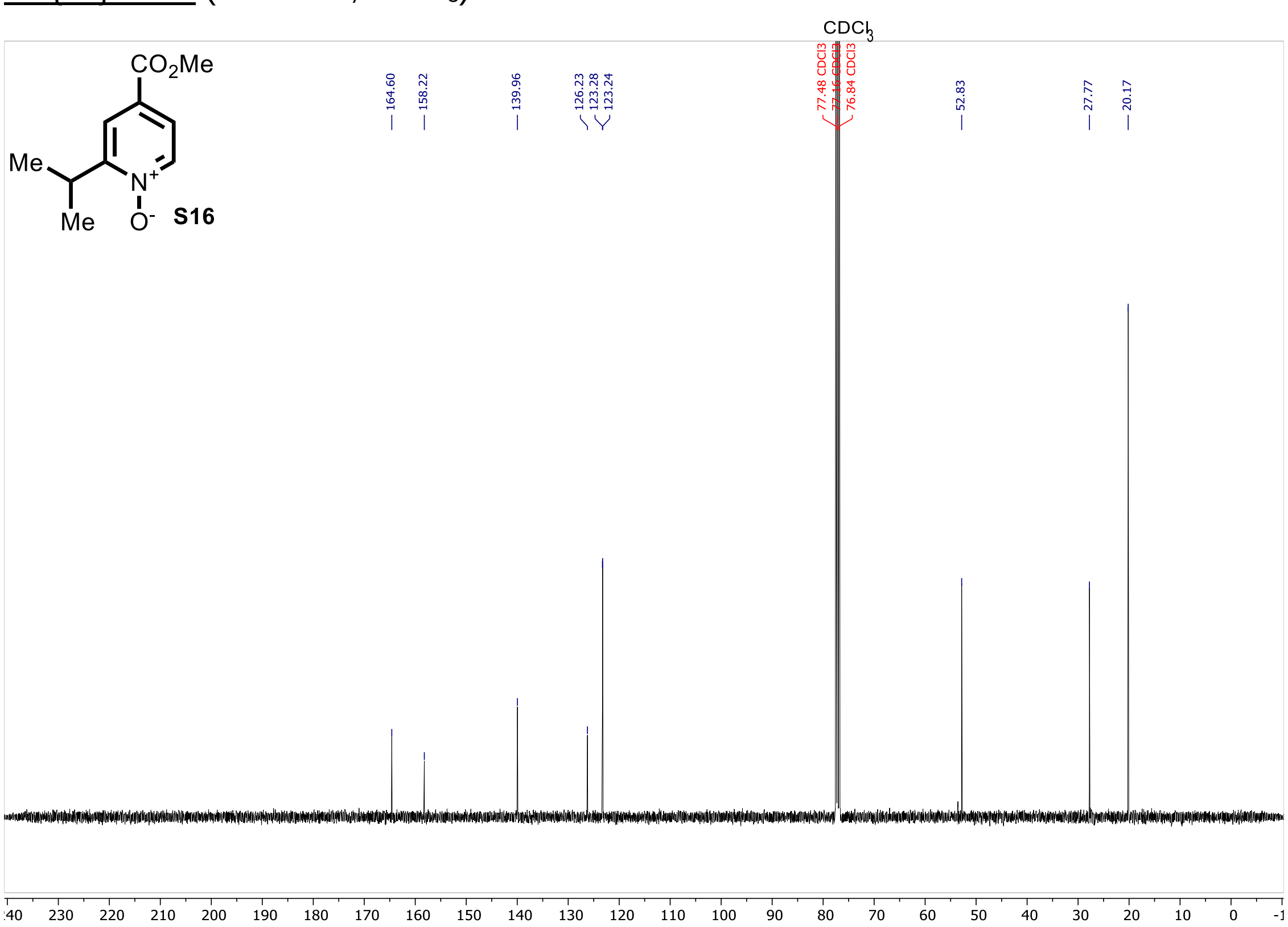

<u>^{1}H NMR</u> (400 MHz, $CDCl_3$)

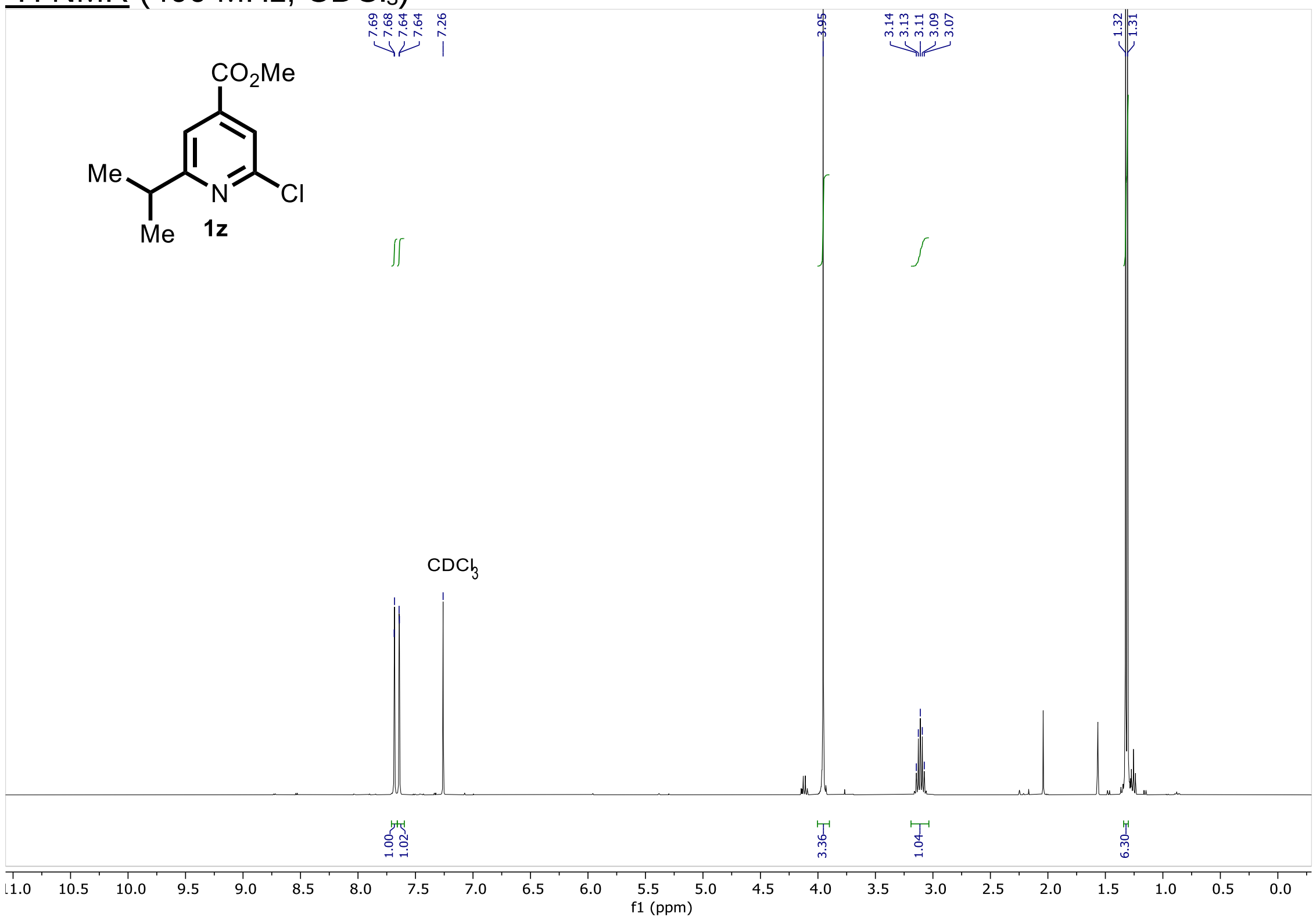

<u>^{13}C{^{1}H} NMR</u> (101 MHz, $CDCl_3$)

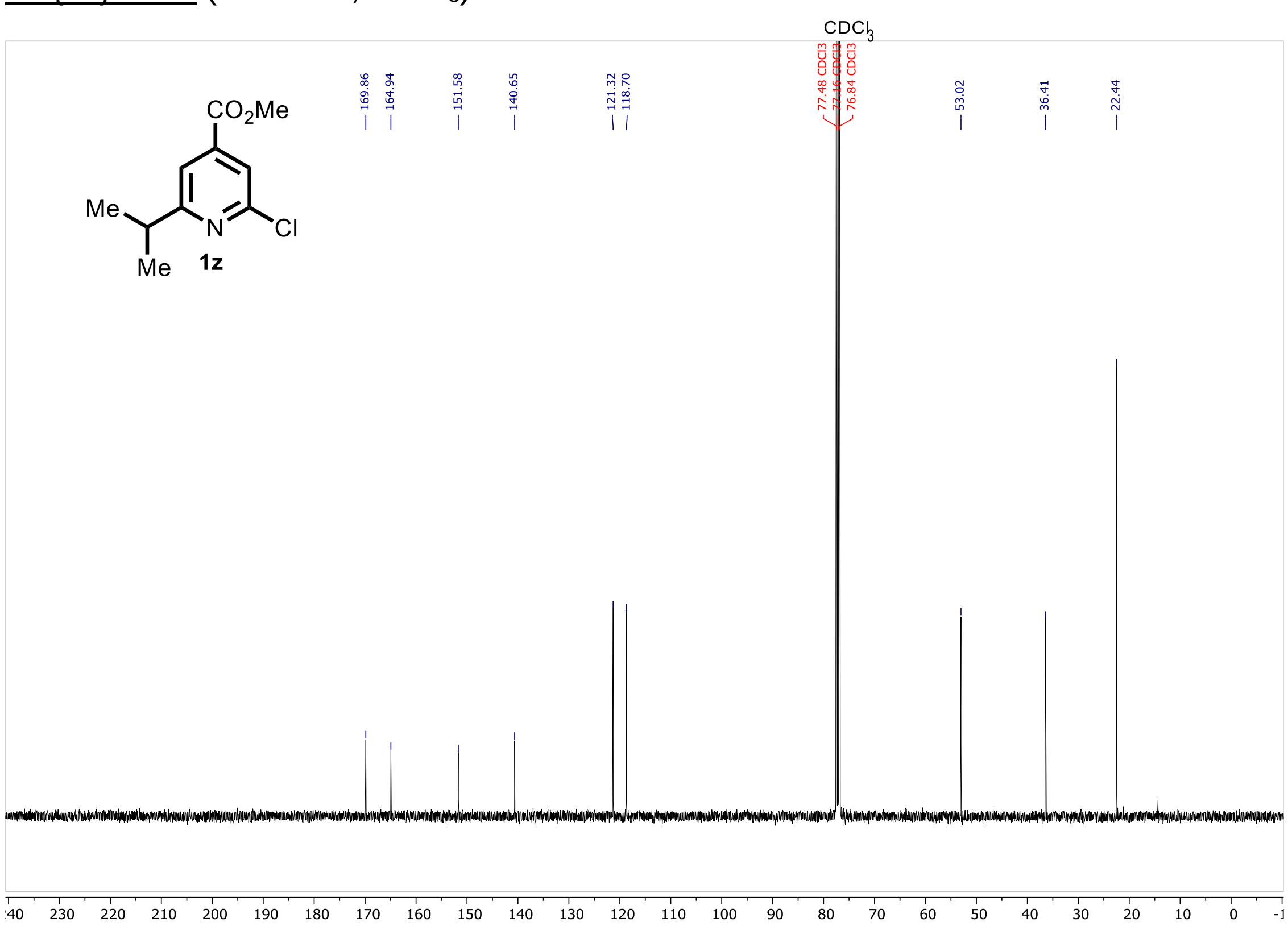

^{1}H NMR (400 MHz, $CDCl_3$)

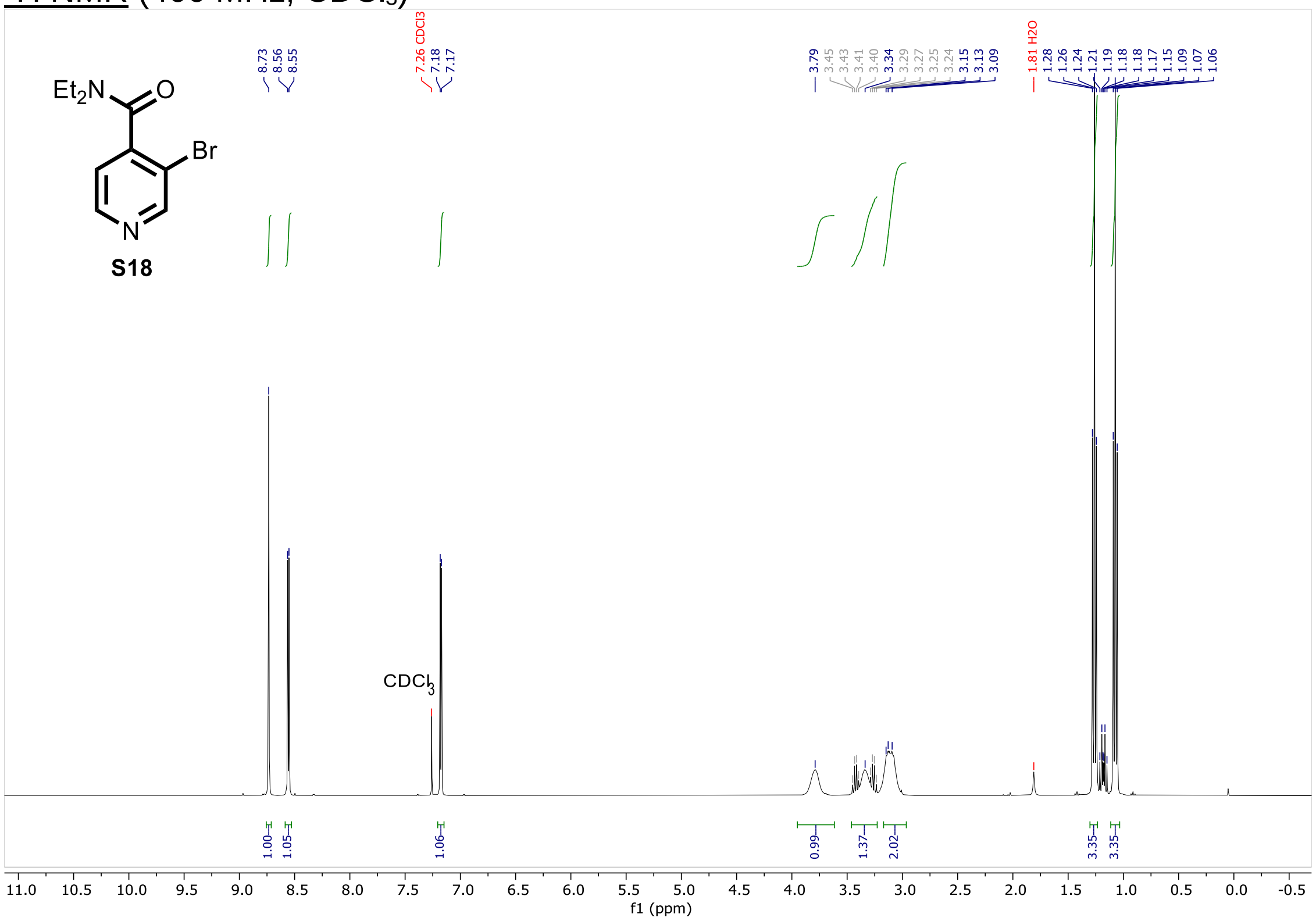

$^{13}C\{^{1}H\}$ NMR (101 MHz, $CDCl_3$)

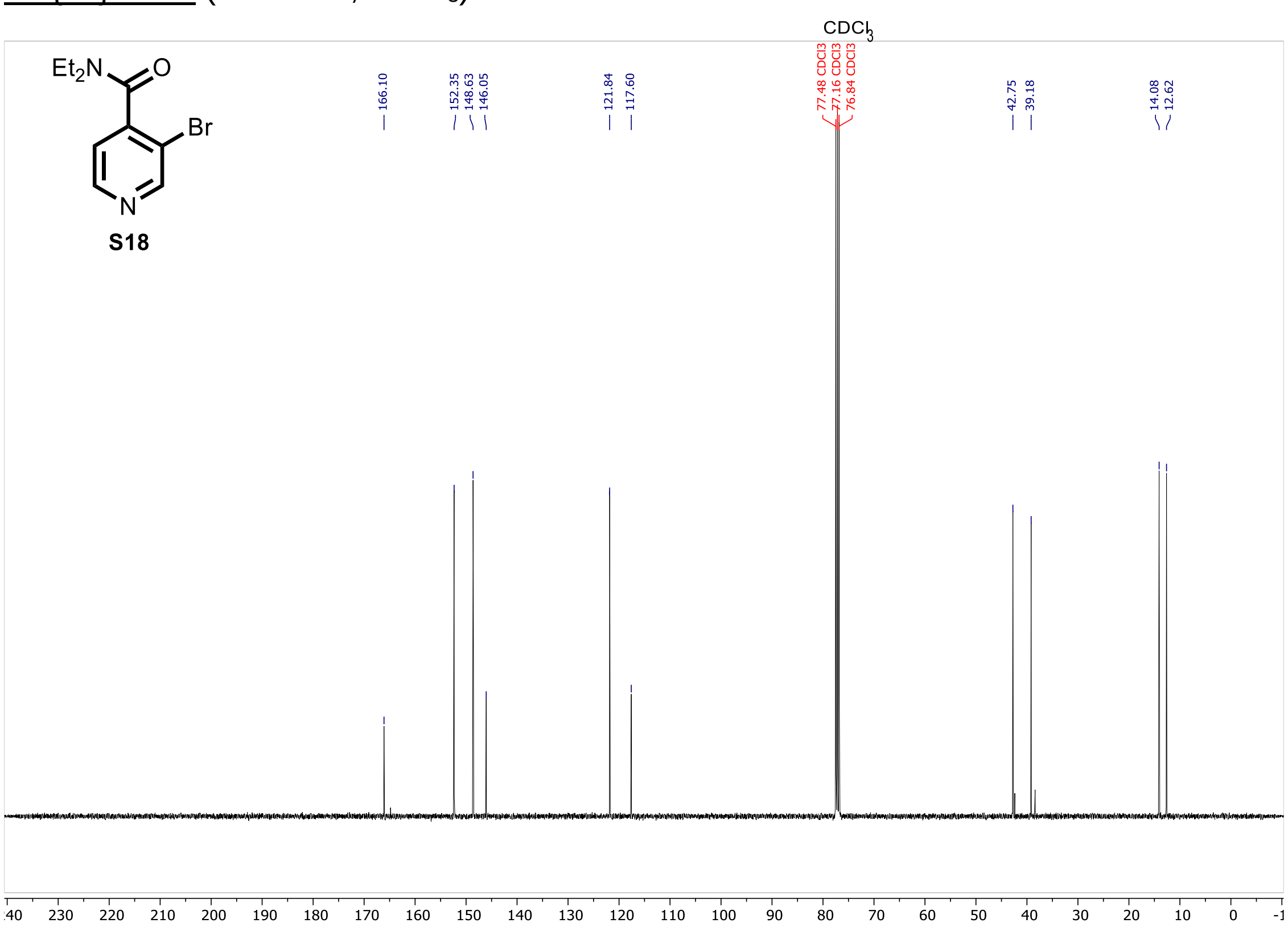

^{1}H NMR (400 MHz, $CDCl_3$)

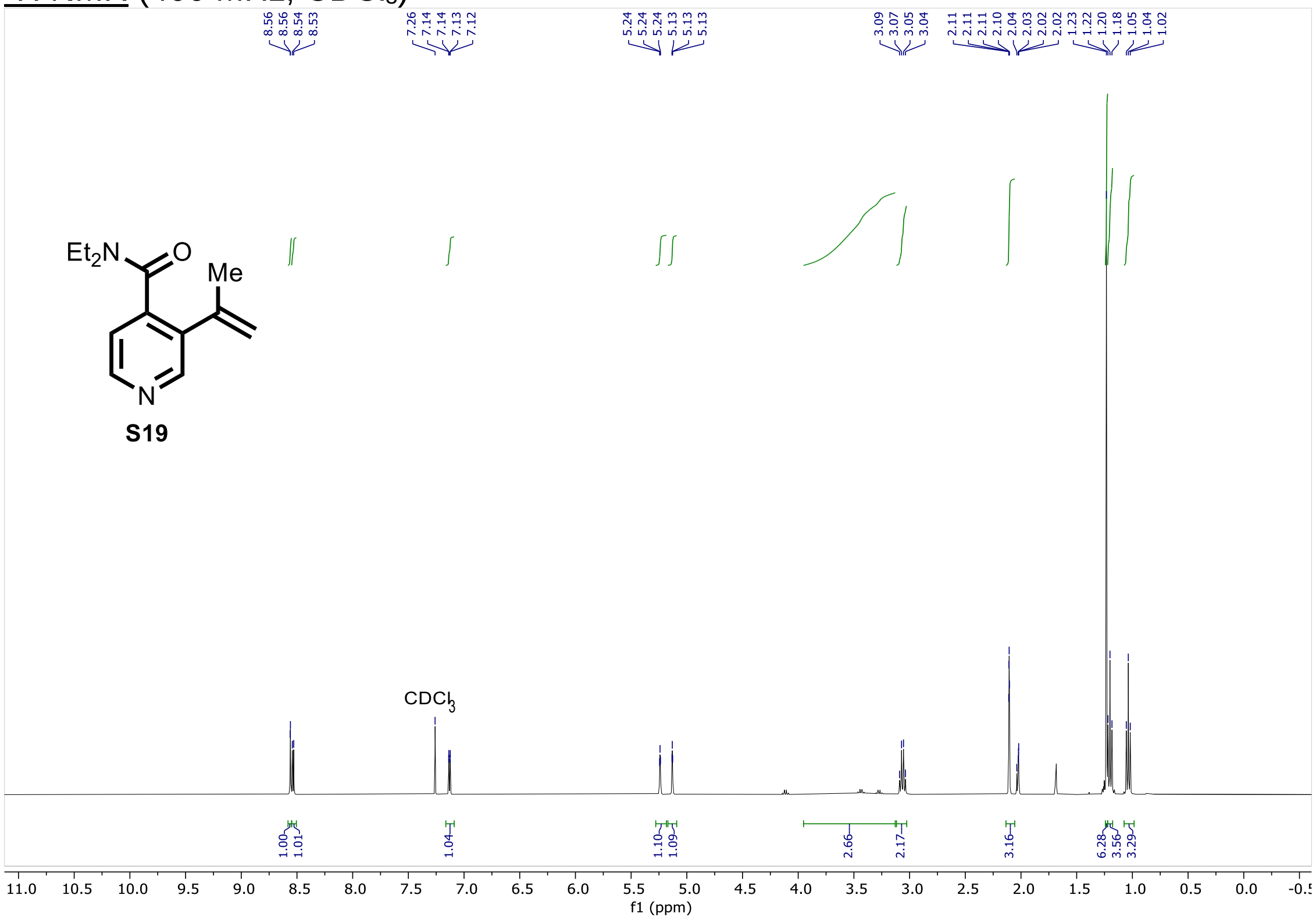

S19

^{13}C{^{1}H} NMR (101 MHz, $CDCl_3$)

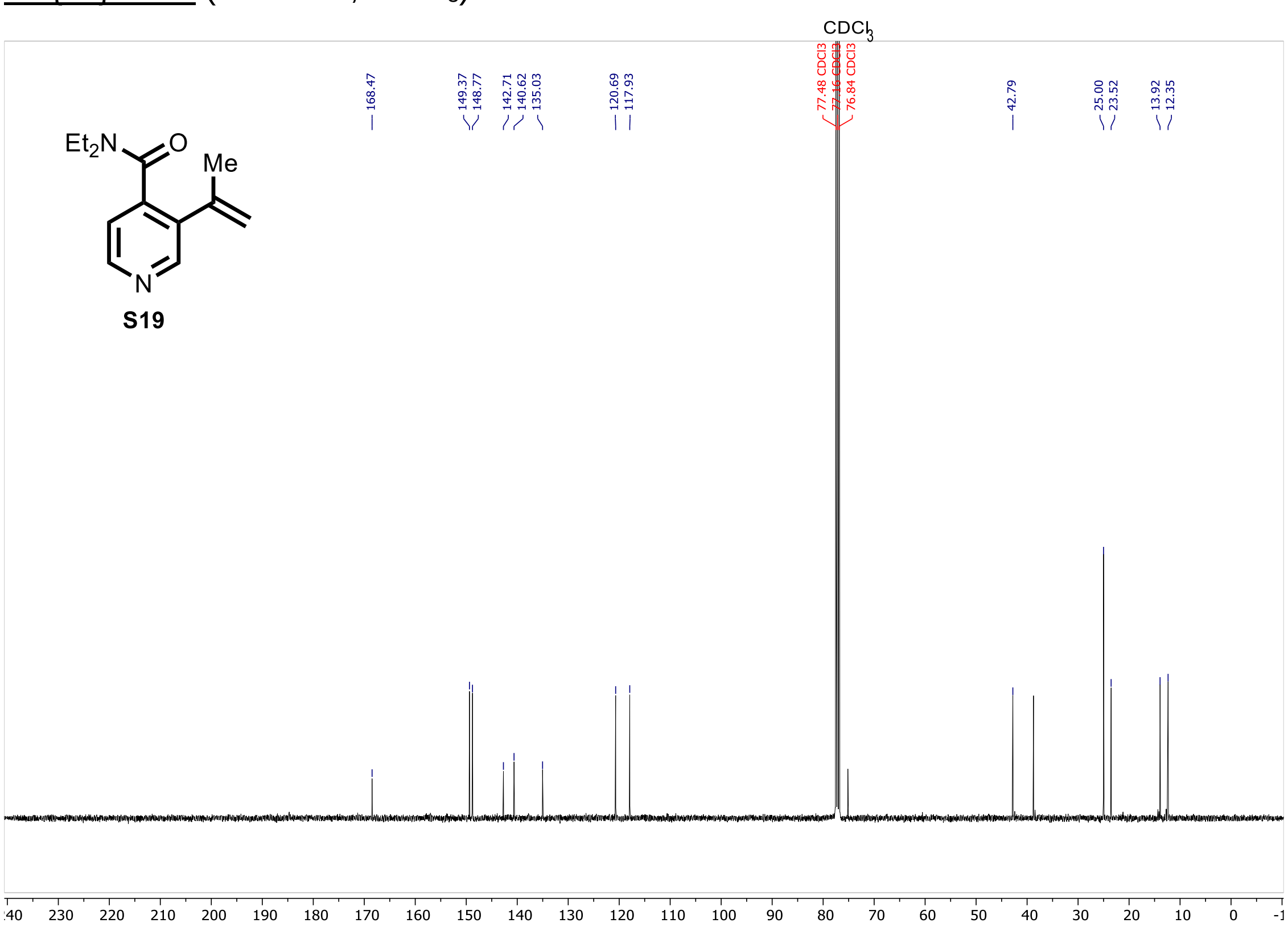

S19

^{1}H NMR (400 MHz, $CDCl_3$)

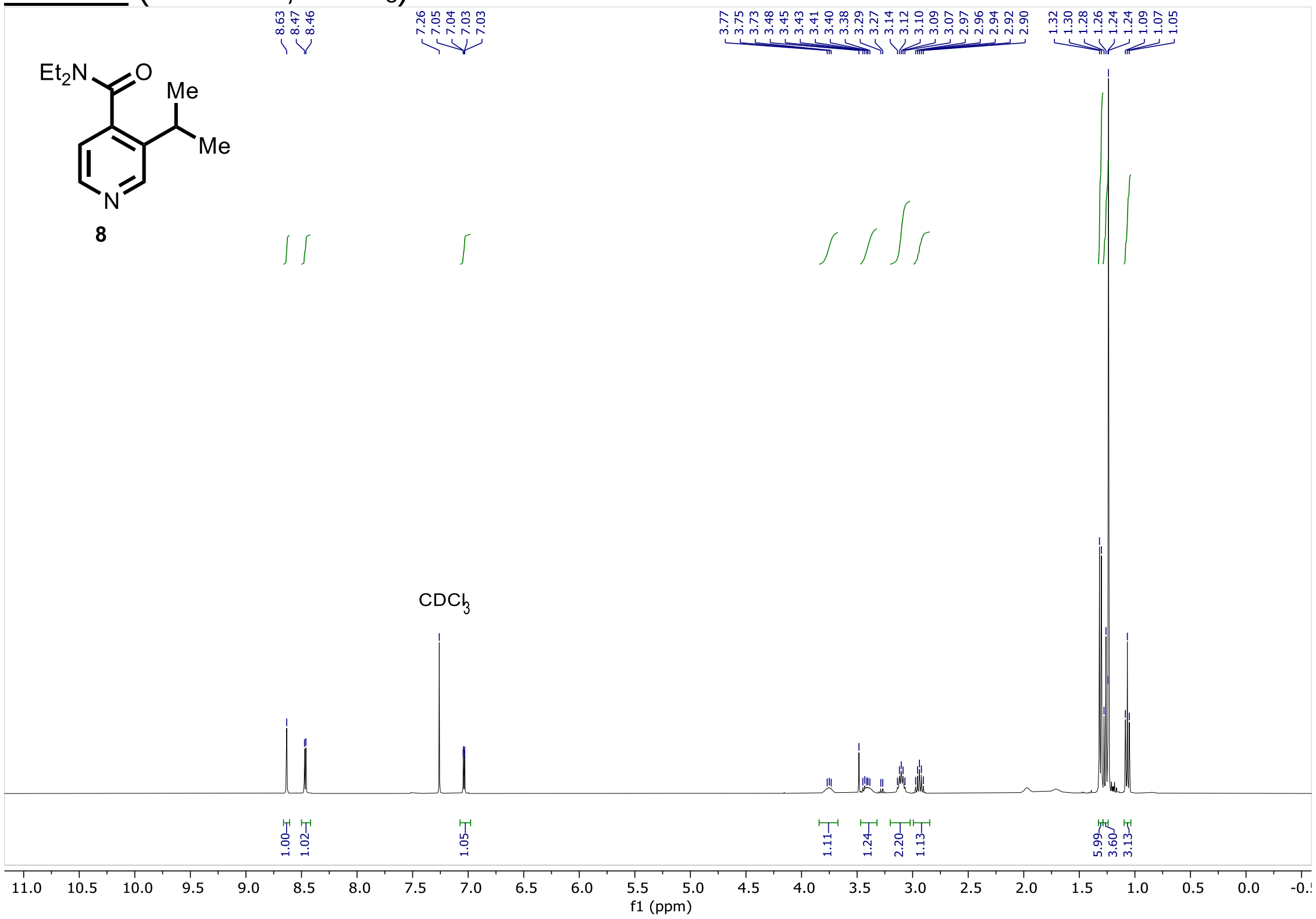

$^{13}C\{^{1}H\}$ NMR (101 MHz, $CDCl_3$)

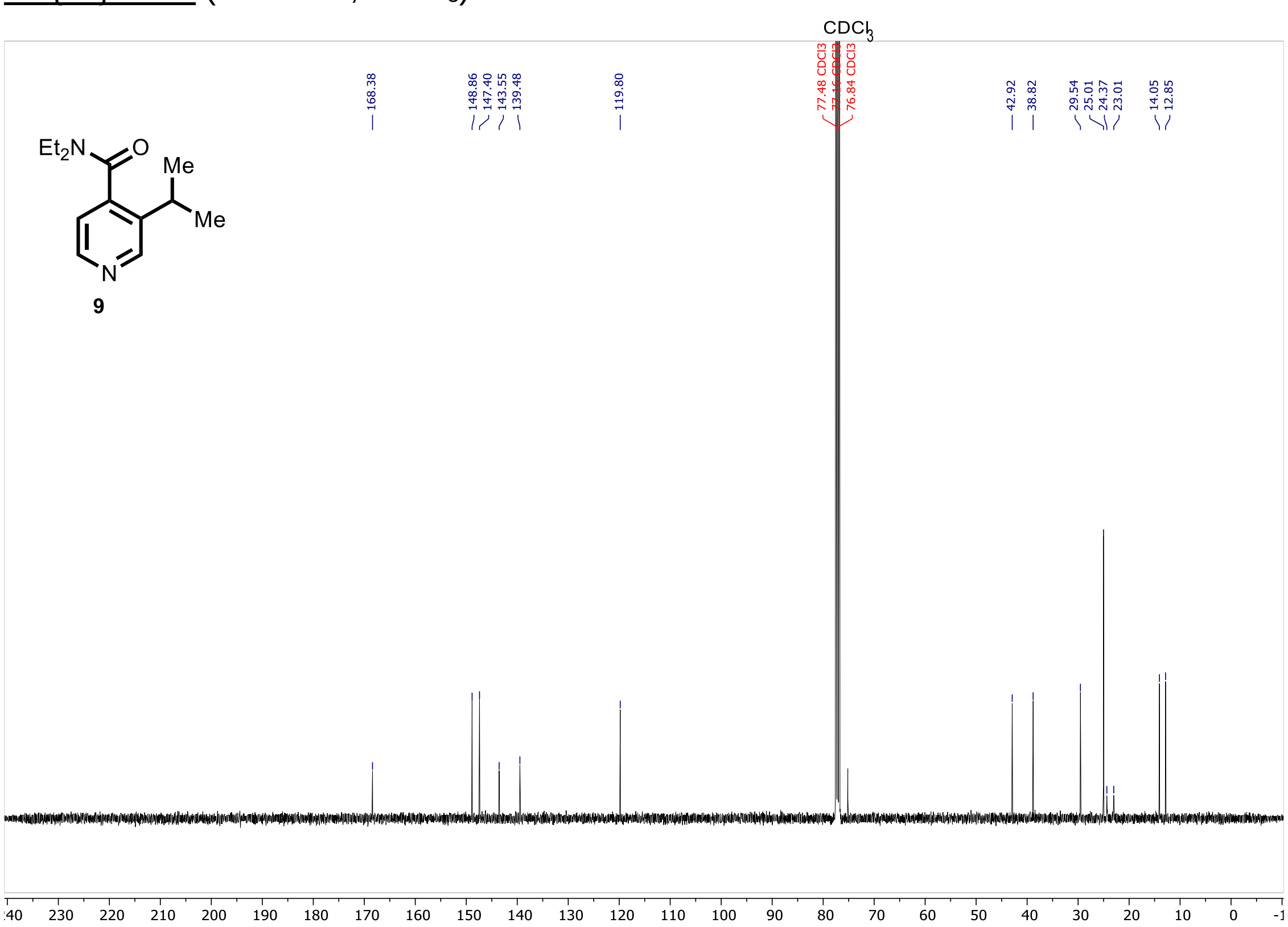

^{1}H NMR (600 MHz, $CDCl_3$)

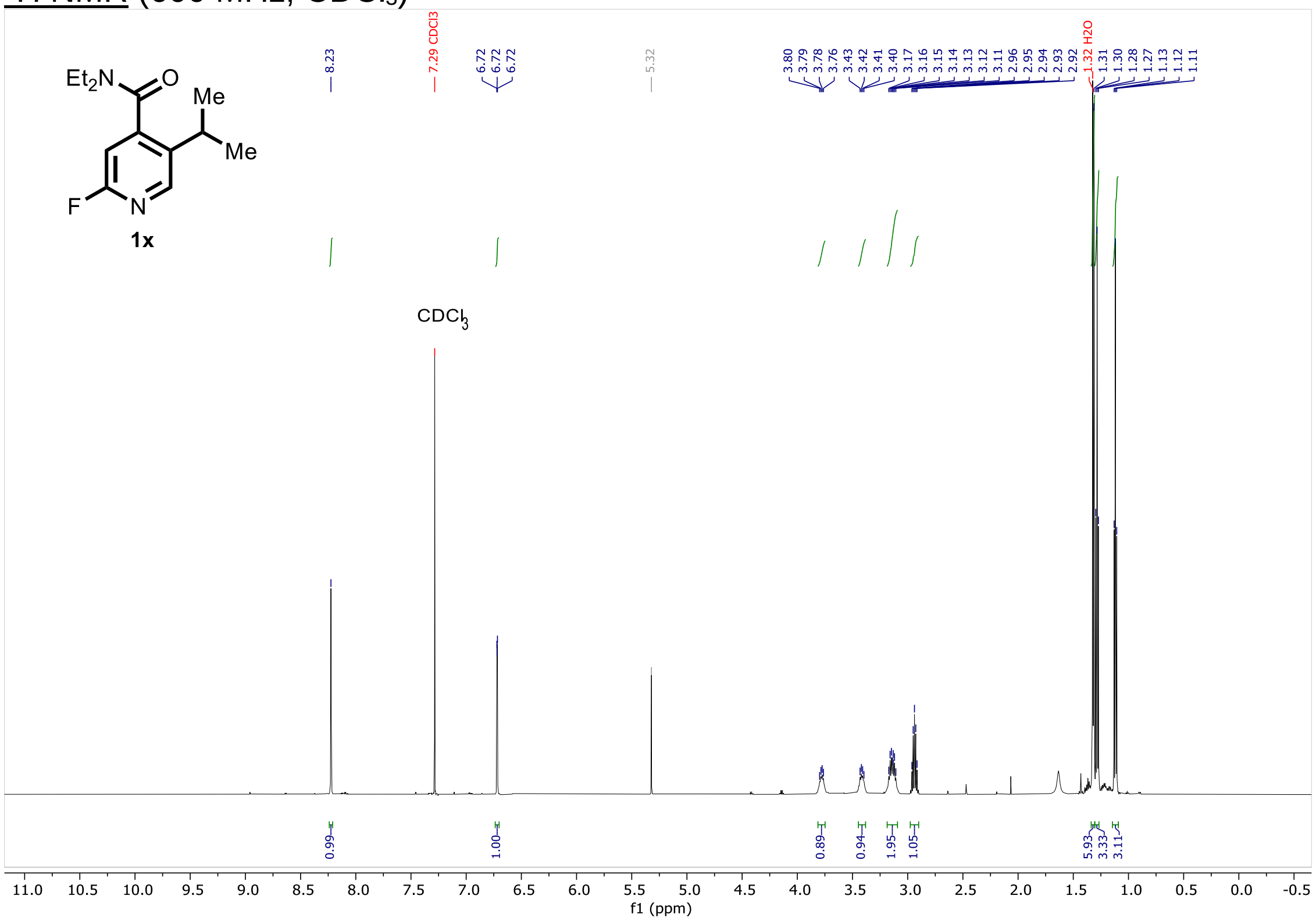

^{19}F NMR (565 MHz, $CDCl_3$)

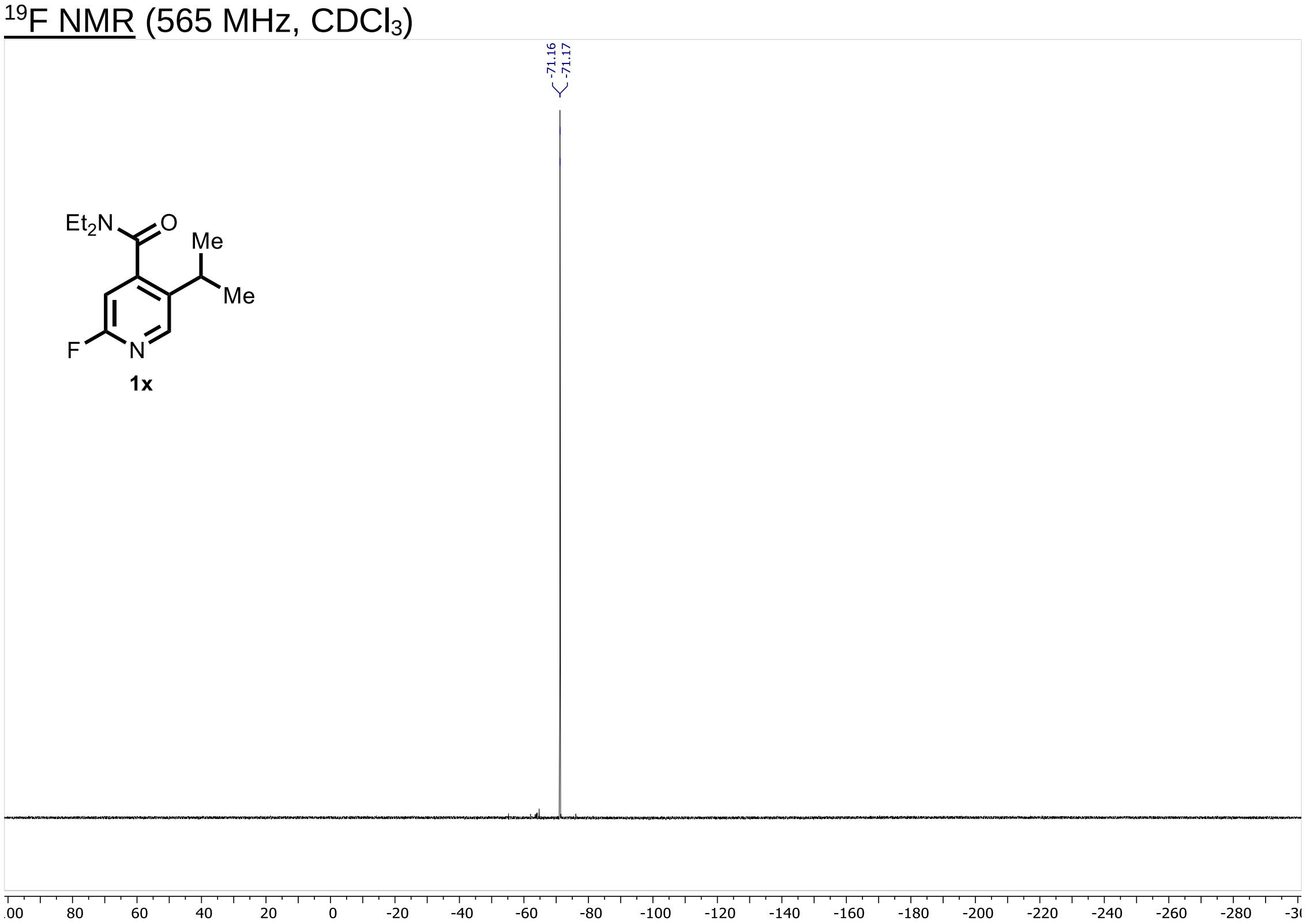

$^{13}C\{^1H\}$ NMR (151 MHz, $CDCl_3$)

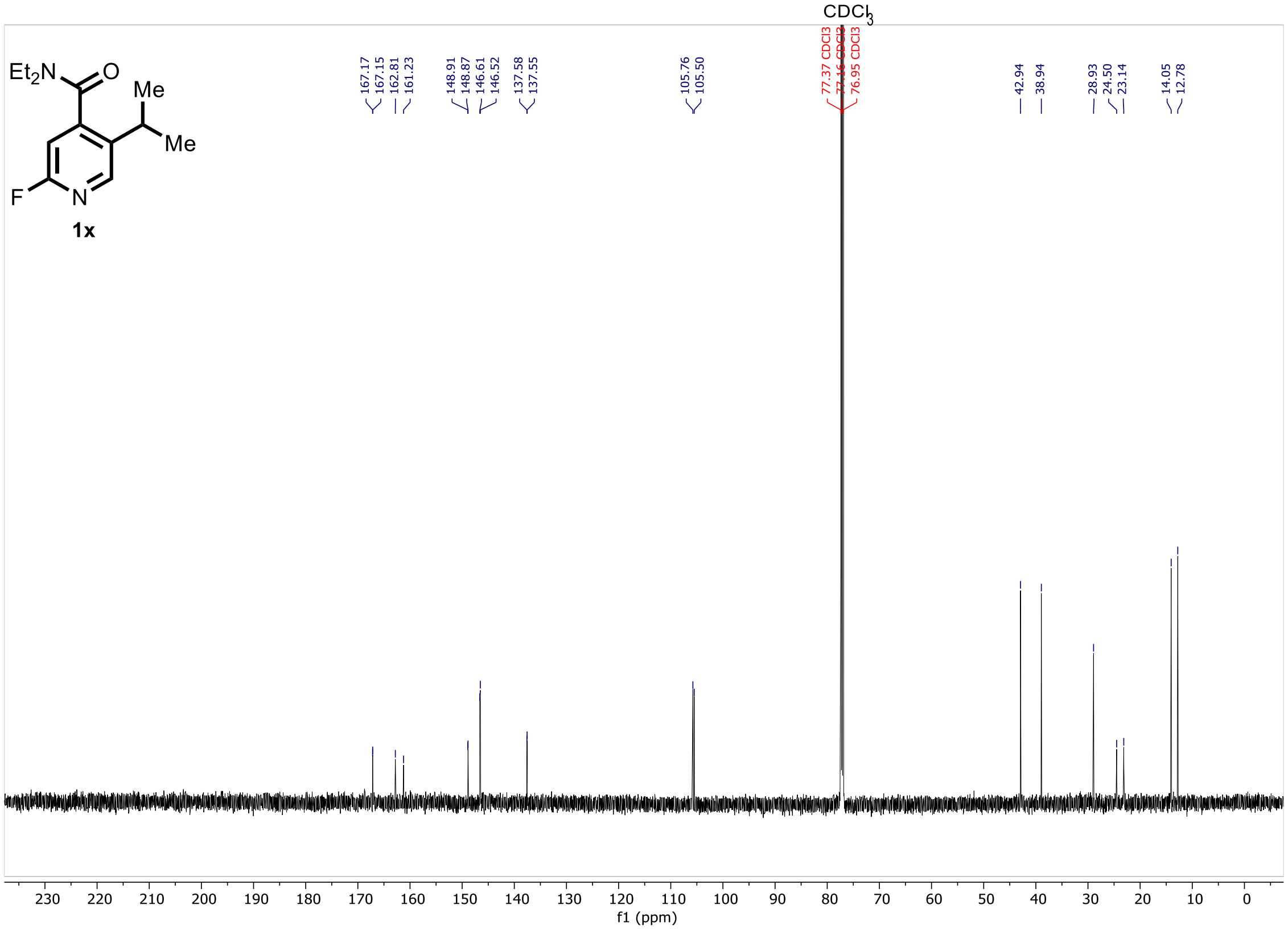

^1H NMR (600 MHz, $CDCl_3$)

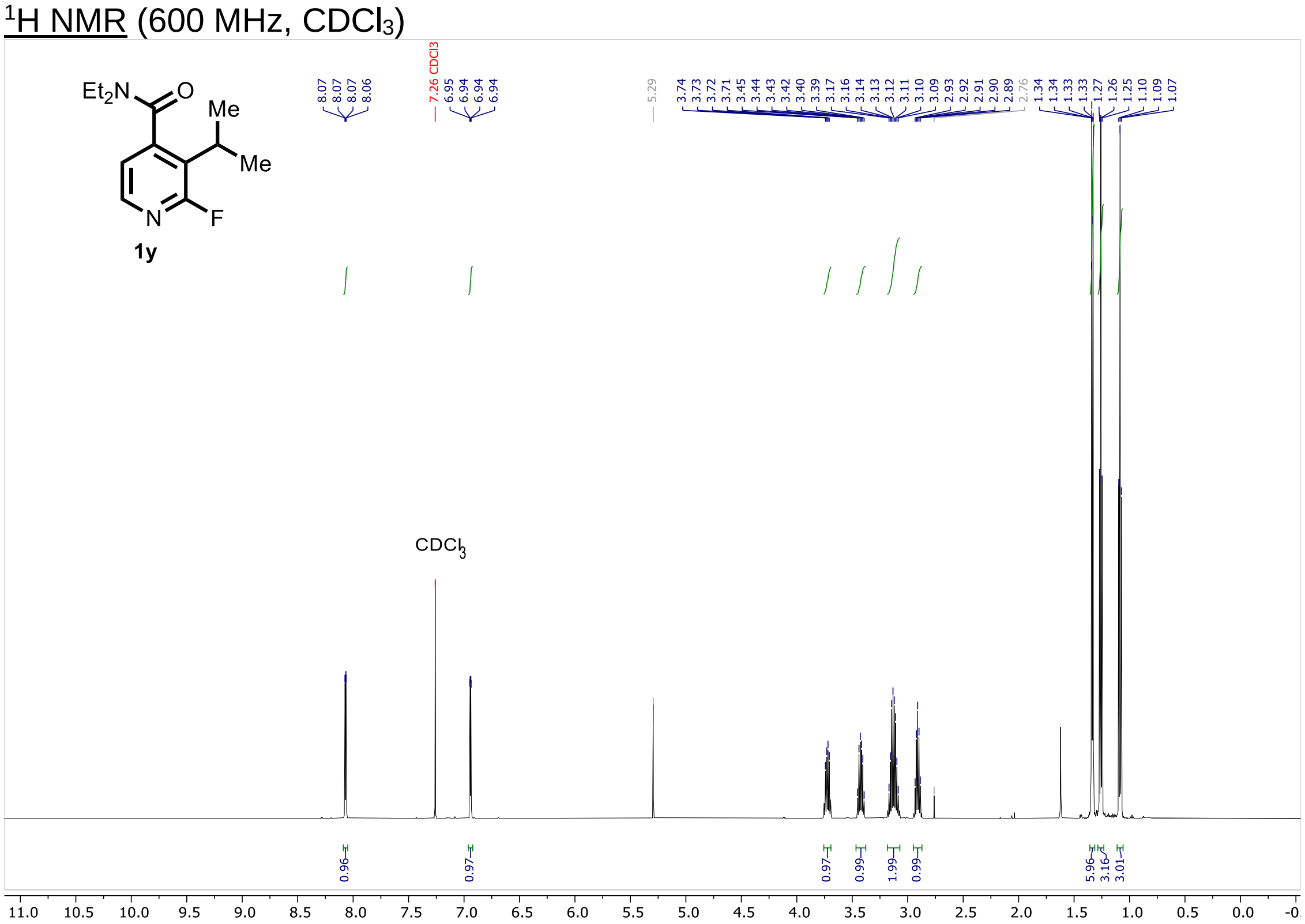

^{19}F NMR (565 MHz, $CDCl_3$)

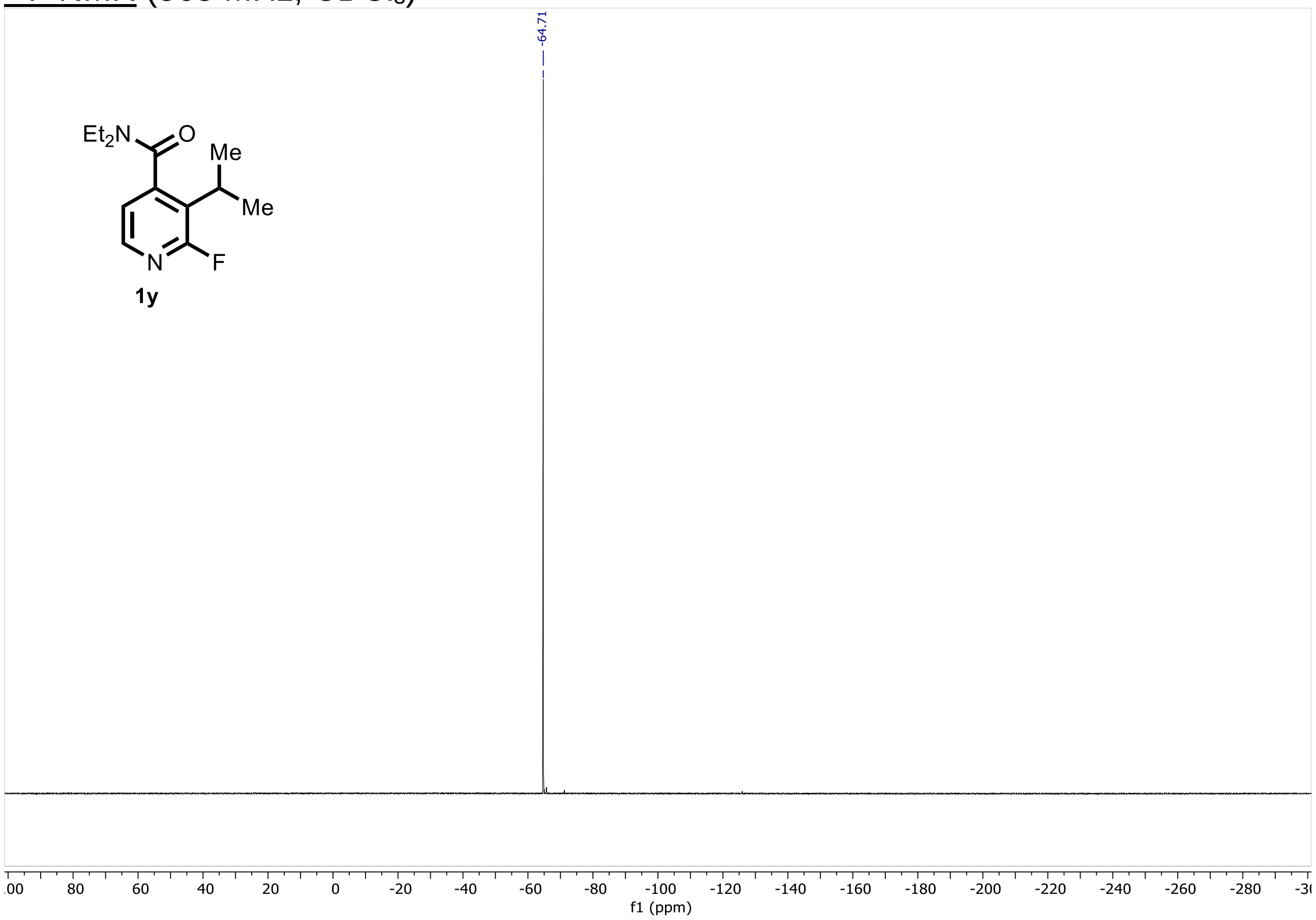

^{13}C{^{1}H} NMR (151 MHz, $CDCl_3$)

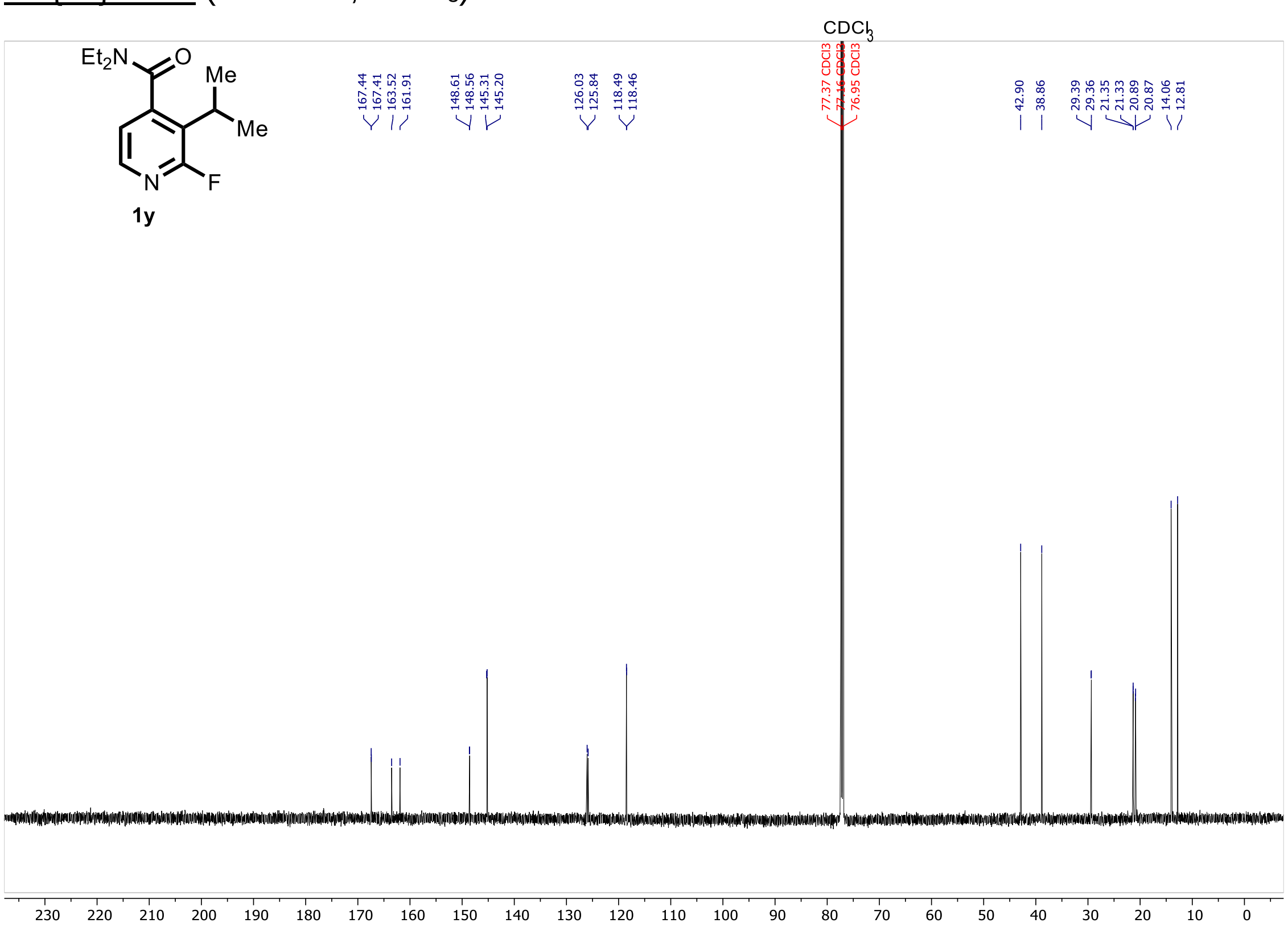

^{1}H NMR (400 MHz, $CDCl_3$)

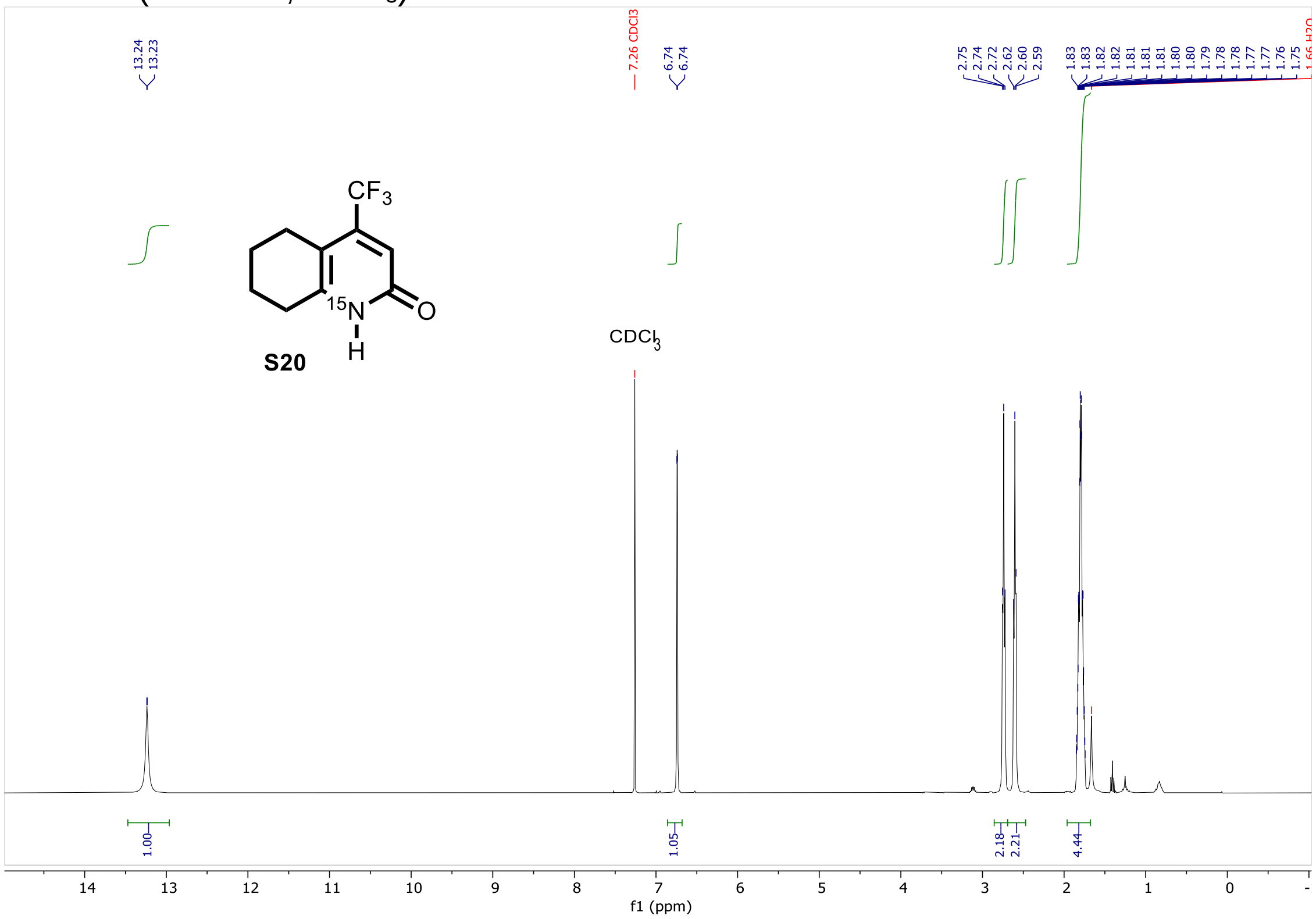

^{19}F NMR (376 MHz, $CDCl_3$)

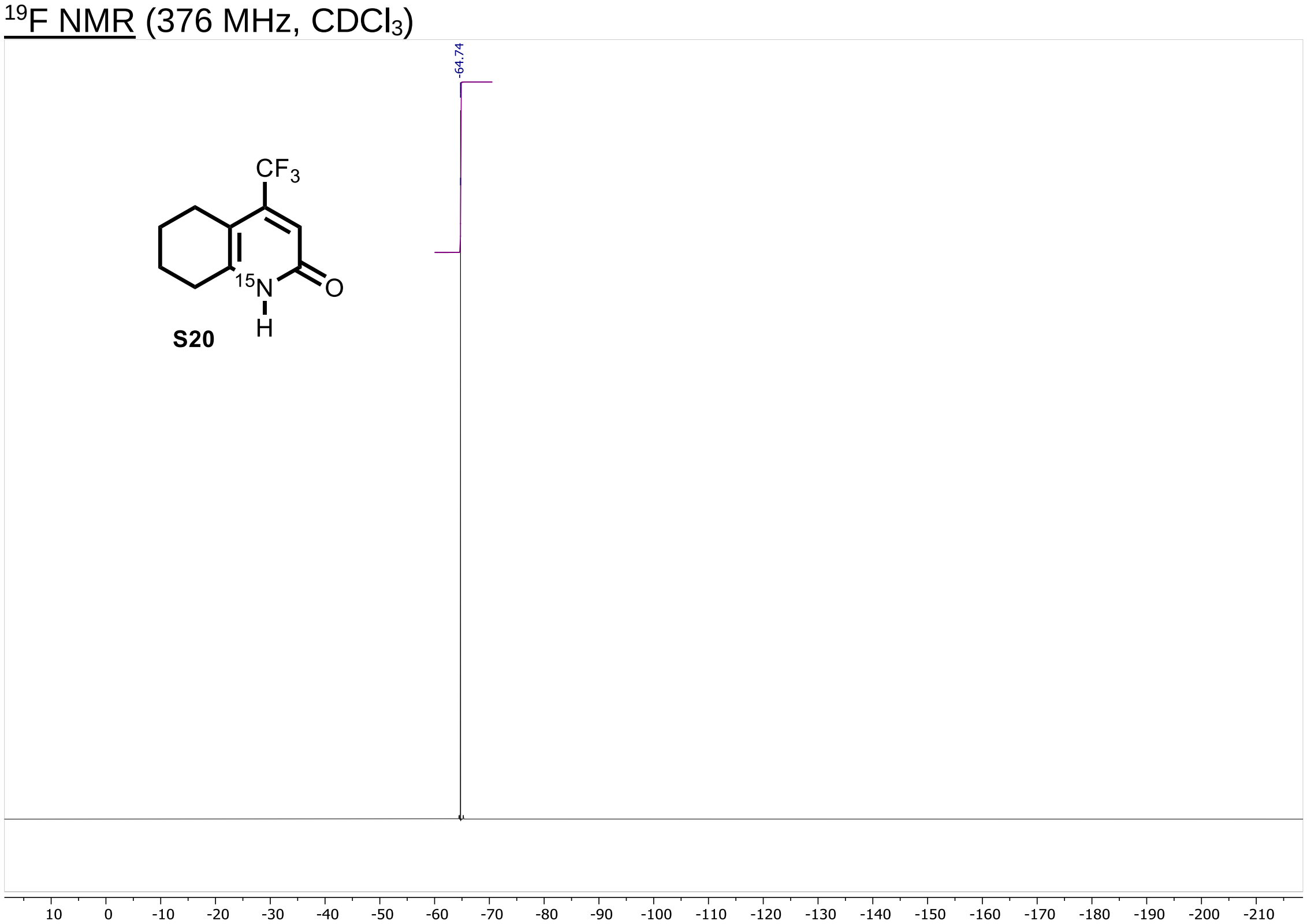

13C{1H} NMR (101 MHz, $CDCl_3$)

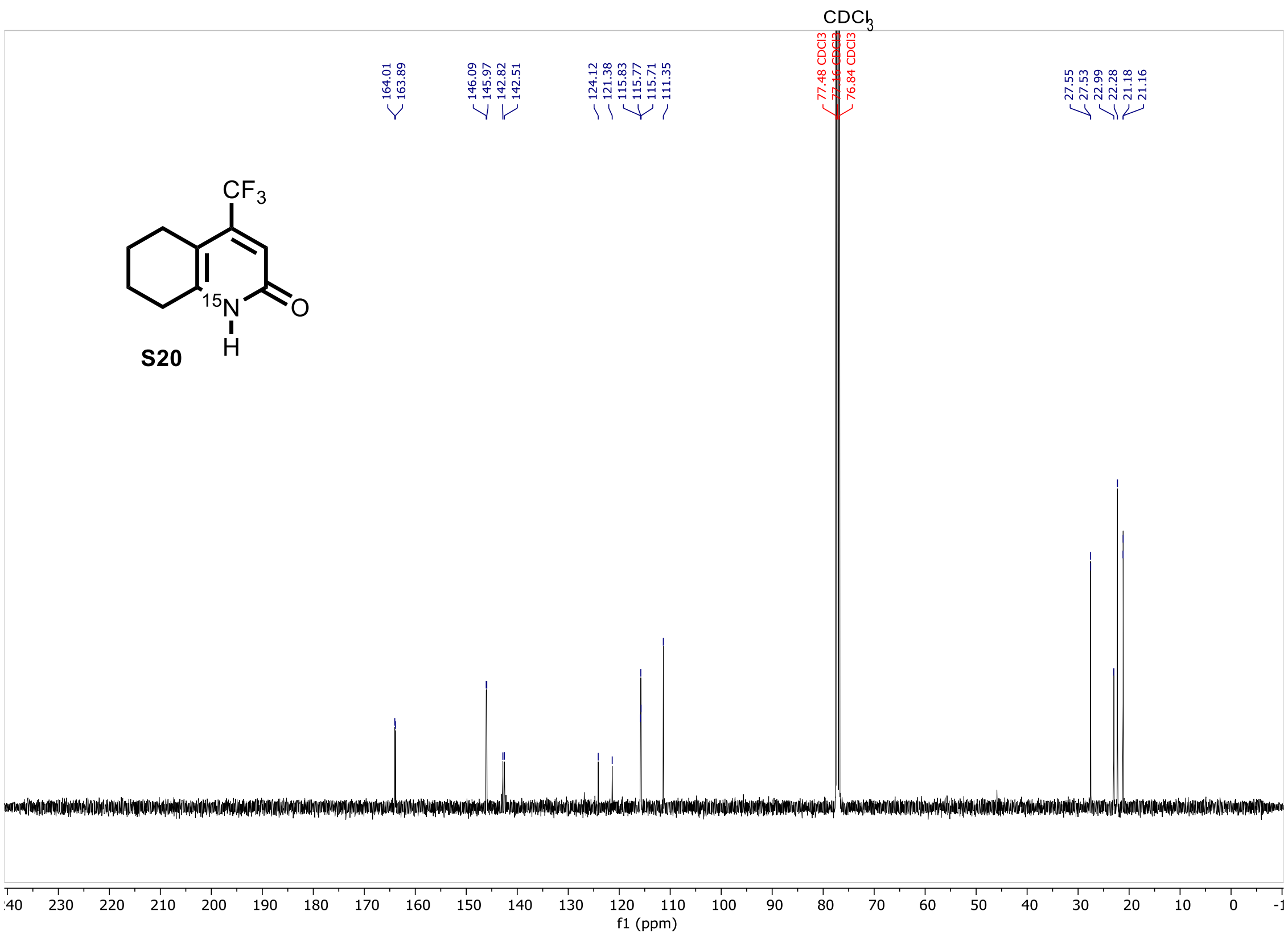

15N-1H-HMBC ($CDCl_3$)

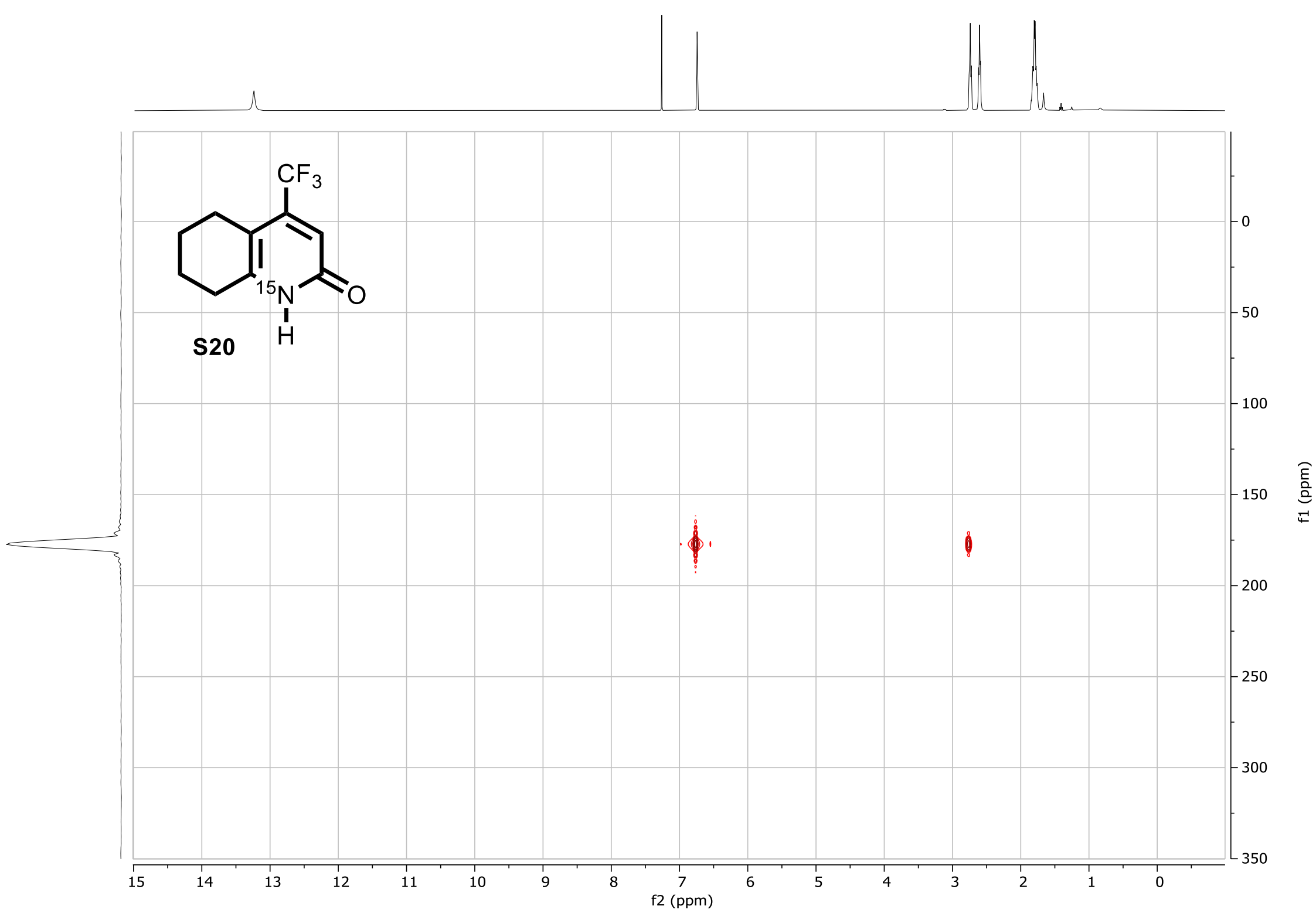

^{1}H NMR (400 MHz, $CDCl_3$)

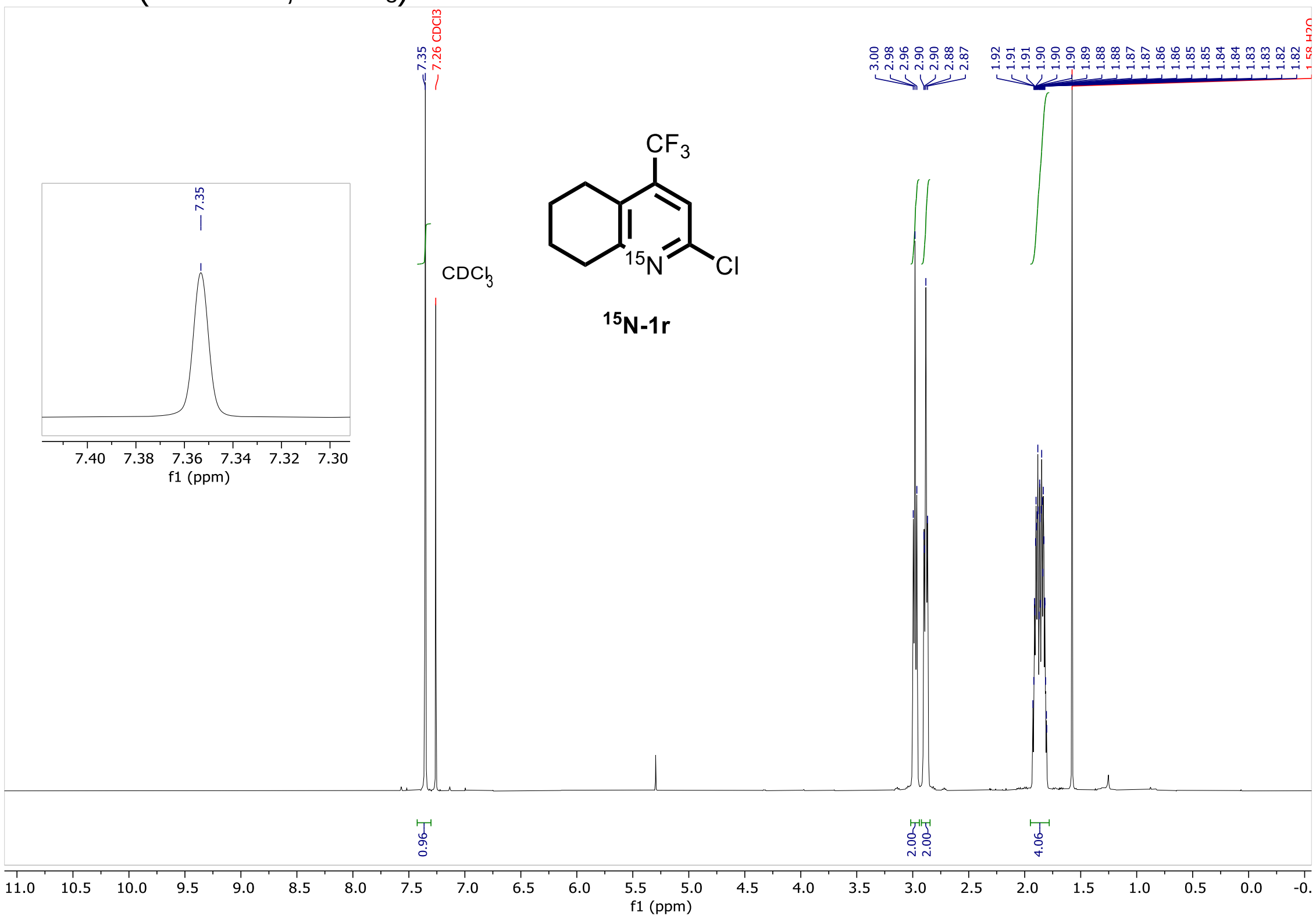

^{19}F NMR (376 MHz, $CDCl_3$)

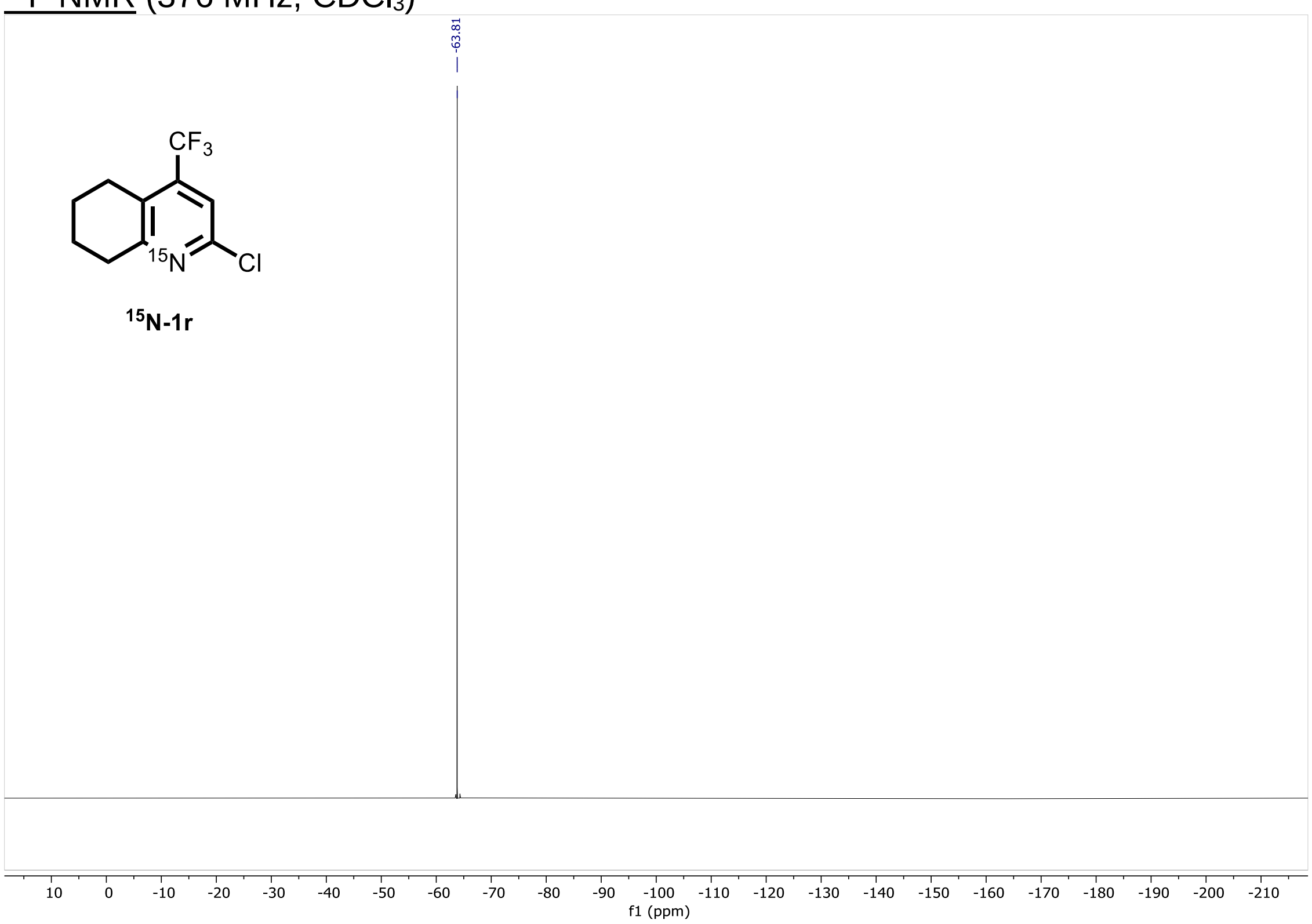

$^{13}C\{^{1}H\}$ NMR (101 MHz, $CDCl_3$)

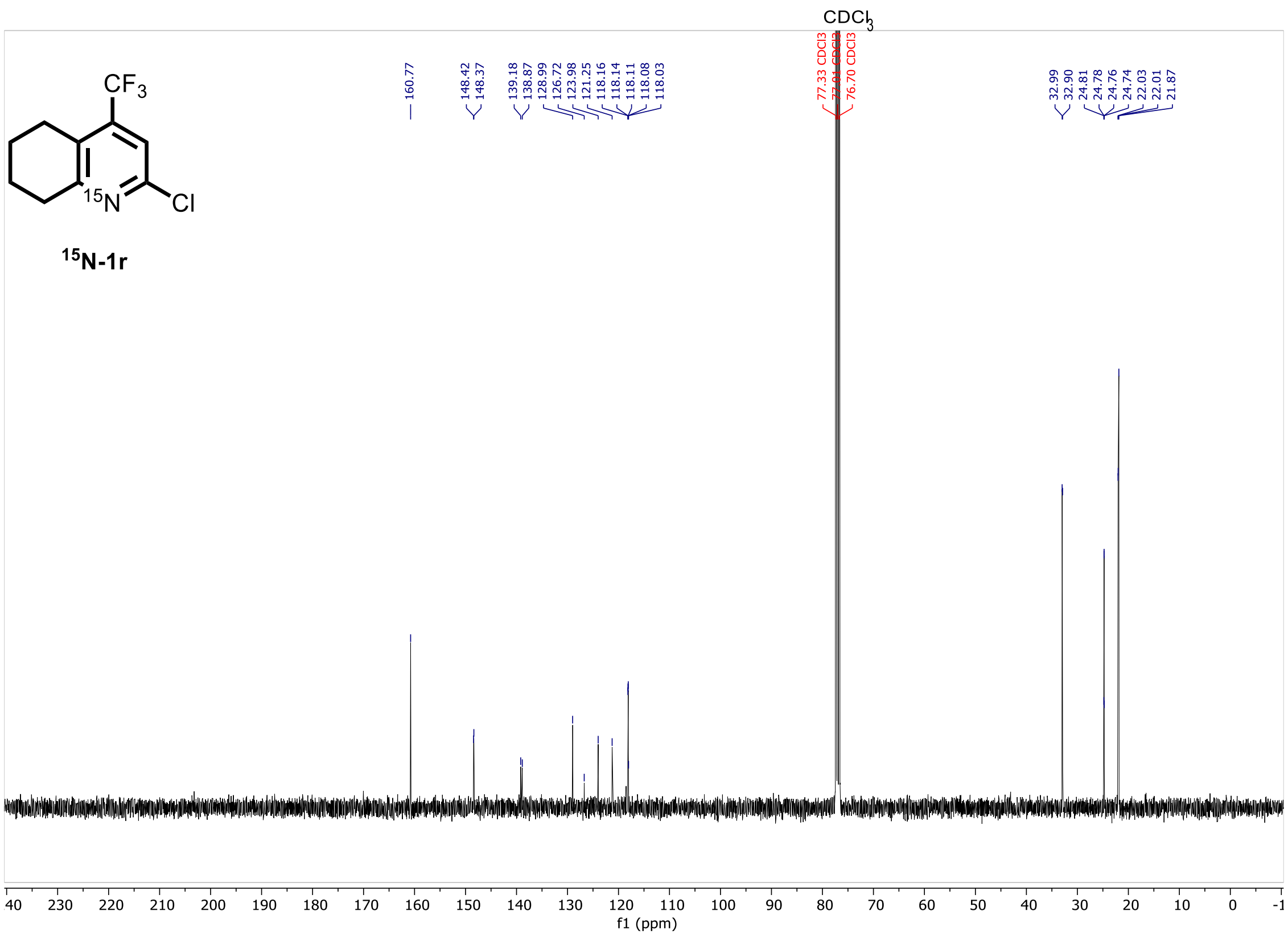

^{15}N-^{1}H-HMBC ($CDCl_3$)

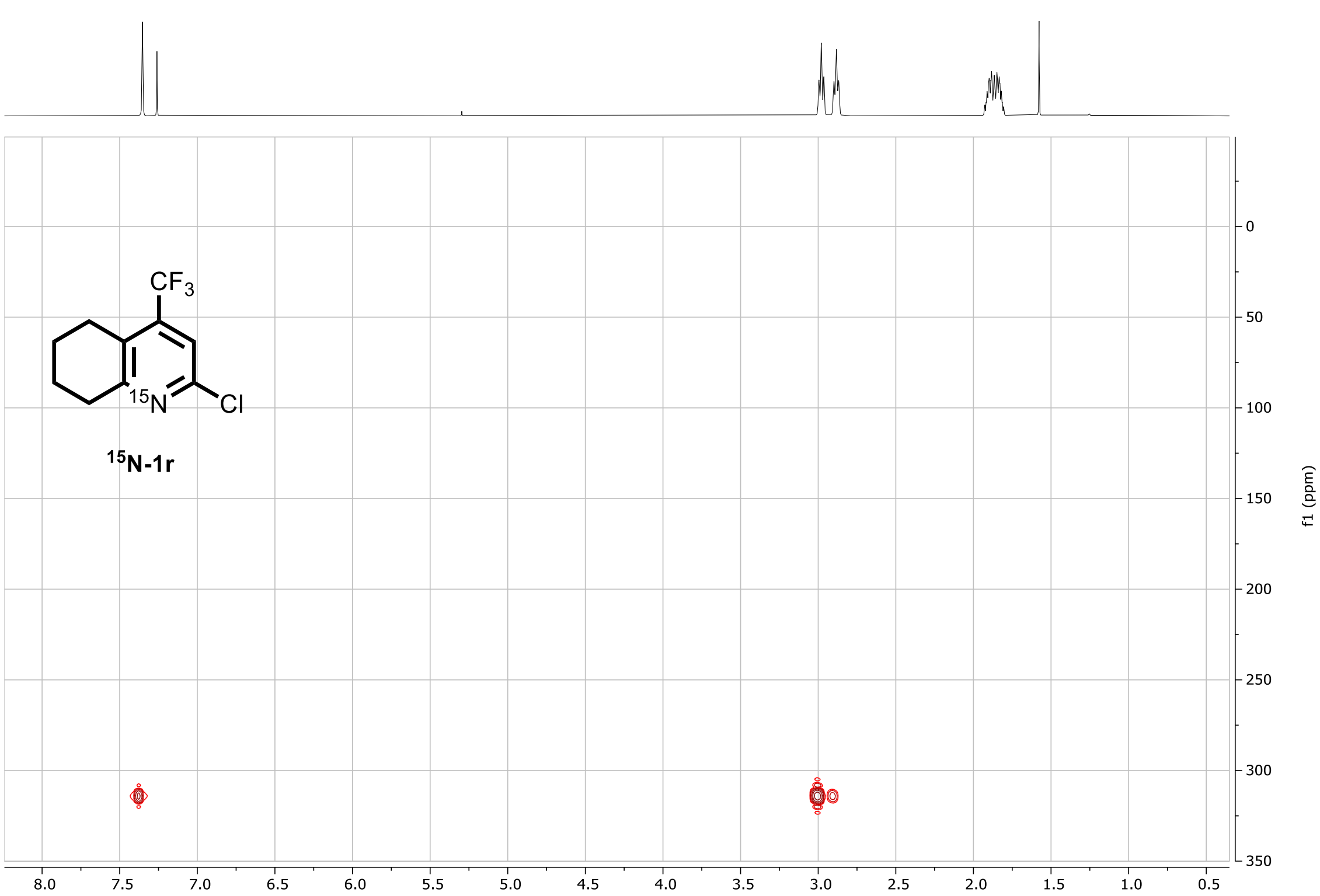

Daicel CHIRALPAK IC column hexane:IPA 80:20, 0.8 mL/min, λ = 254 nm

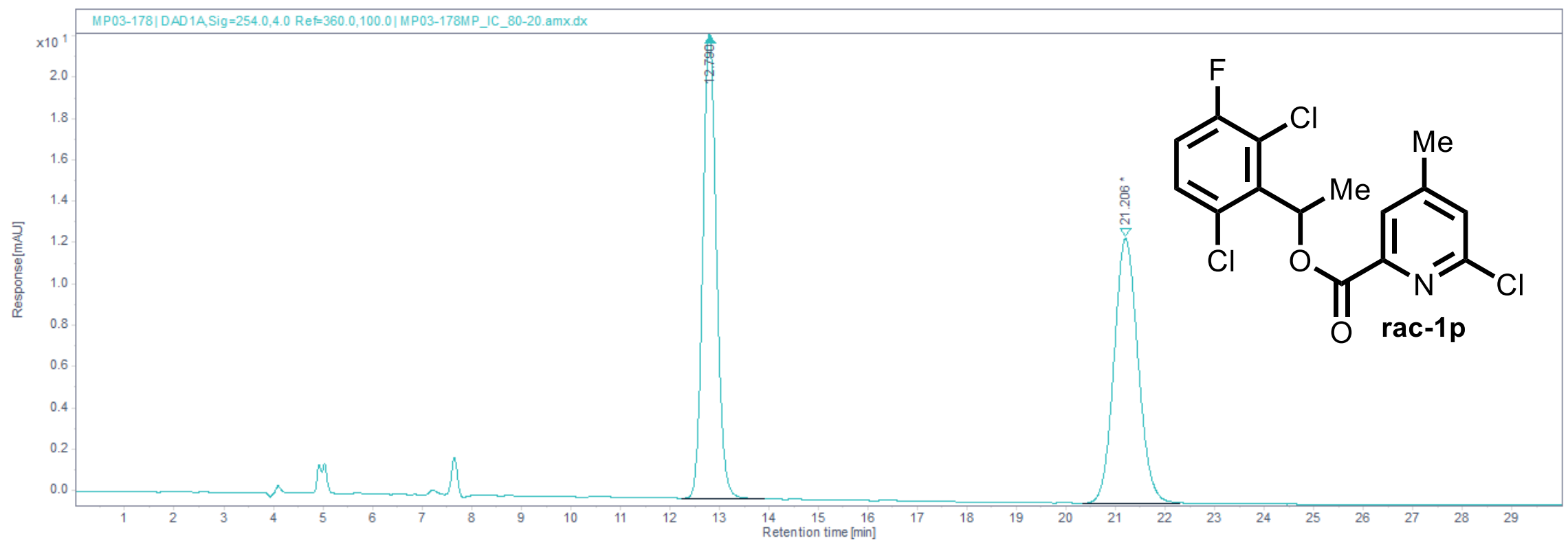

1	DAD1A,Sig=254.0,4.0 Ref=360.0,100.0	12.790	432.671	50.068	22.687	63.89	12.232	13.918
2	DAD1A,Sig=254.0,4.0 Ref=360.0,100.0	21.206	431.489	49.932	12.821	36.11	20.352	22.295

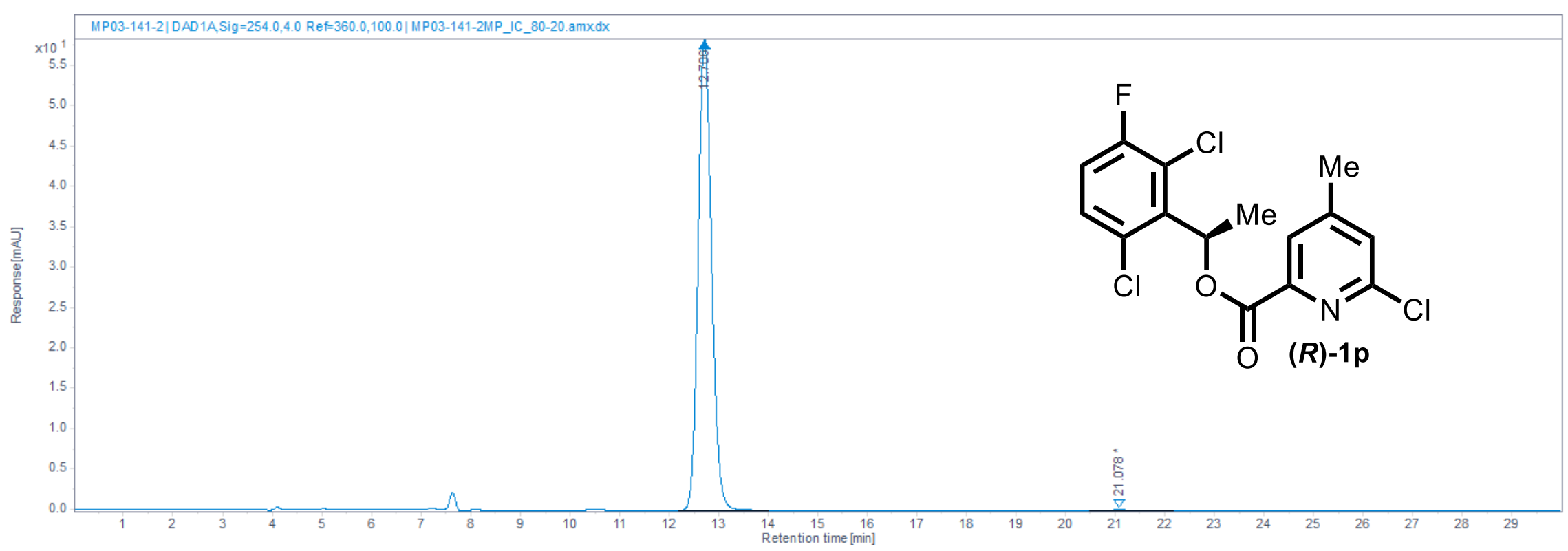

1	DAD1A,Sig=254.0,4.0 Ref=360.0,100.0	12.706	1100.584	99.821	58.487	99.89	12.182	14.015
2	DAD1A,Sig=254.0,4.0 Ref=360.0,100.0	21.078	1.974	0.179	0.065	0.11	20.487	22.190

DAICEL CHIRALPAK IA column hexane:IPA 80:20, 0.8 mL/min, λ = 230 nm

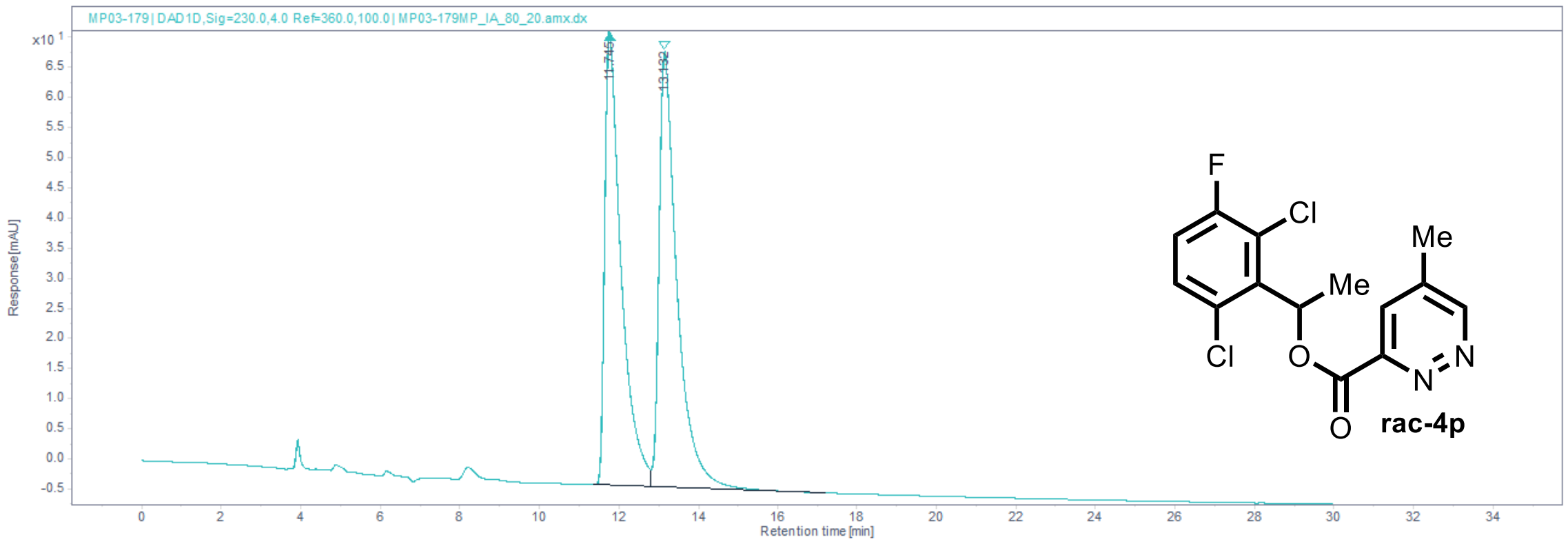

1	DAD1D,Sig=230.0,4.0 Ref=360.0,100.0	11.745	2157.391	48.652	75.469	51.09	11.369	12.787
2	DAD1D,Sig=230.0,4.0 Ref=360.0,100.0	13.132	2276.967	51.348	72.245	48.91	12.787	17.202

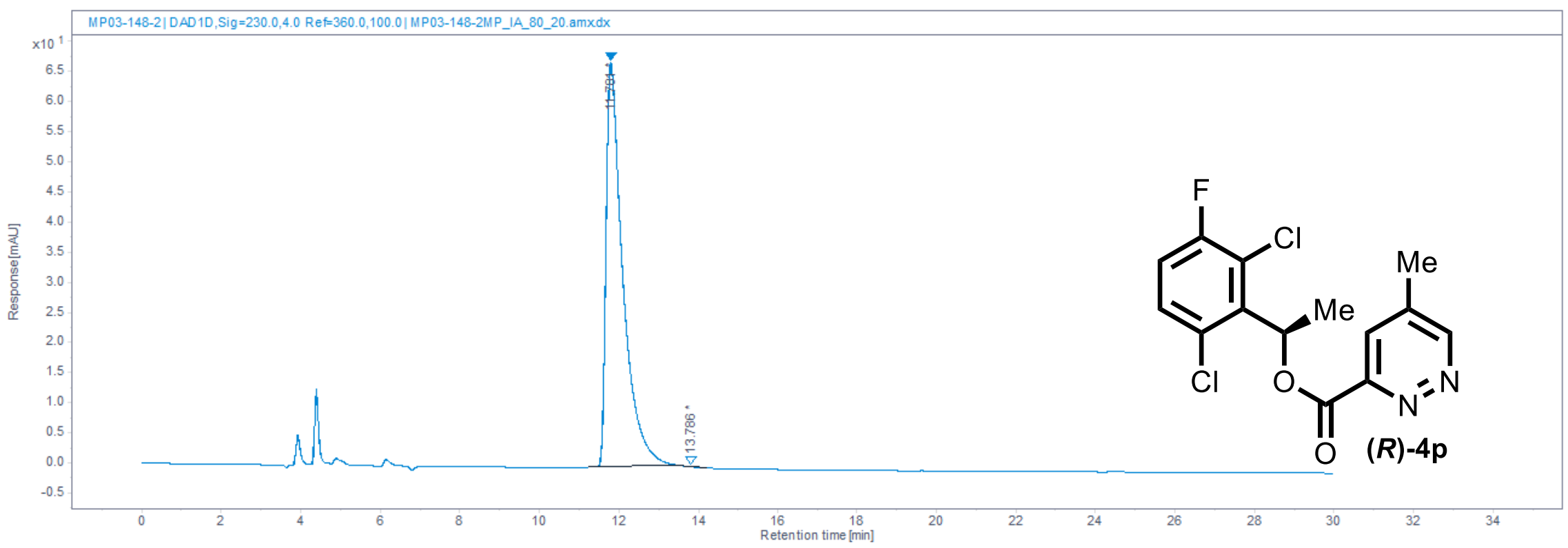

1	DAD1D,Sig=230.0,4.0 Ref=360.0,100.0	11.781	1954.618	99.879	67.097	99.89	11.247	13.436
2	DAD1D,Sig=230.0,4.0 Ref=360.0,100.0	13.786	2.364	0.121	0.076	0.11	13.436	14.227

References and Notes

1. D. C. Blakemore, L. Castro, I. Churcher, D. C. Rees, A. W. Thomas, D. M. Wilson, A. Wood, Organic synthesis provides opportunities to transform drug discovery. *Nat. Chem.* **10**, 383–394 (2018). doi:10.1038/s41557-018-0021-z Medline

2. L. D. Pennington, P. N. Collier, E. Comer, Harnessing the necessary nitrogen atom in chemical biology and drug discovery. *Med. Chem. Res.* **32**, 1278–1293 (2023). doi:10.1007/s00044-023-03073-3

3. T. Hoffmann, M. Gastreich, The next level in chemical space navigation: Going far beyond enumerable compound libraries. *Drug Discov. Today* **24**, 1148–1156 (2019). doi:10.1016/j.drudis.2019.02.013 Medline

4. J. Krieger, D. Li, D. Papanikolaou, Missing Novelty in Drug Development. *Rev. Financ. Stud.* **35**, 636–679 (2022). doi:10.1093/rfs/hhab024

5. J. Jurczyk, J. Woo, S. F. Kim, B. D. Dherange, R. Sarpong, M. D. Levin, Single-atom logic for heterocycle editing. *Nat. Synth.* **1**, 352–364 (2022). doi:10.1038/s44160-022-00052-1 Medline

6. Z.-Q. Liu, Q. Zhang, Y.-L. Liu, X.-Q. Yu, R.-H. Chui, L.-L. Zhang, B. Zhao, L.-Y. Ma, Recent contributions of pyridazine as a privileged scaffold of anticancer agents in medicinal chemistry: An updated review. *Bioorg. Med. Chem.* **111**, 117847 (2024). doi:10.1016/j.bmc.2024.117847 Medline

7. N. A. Meanwell, The pyridazine heterocycle in molecular recognition and drug discovery. *Med. Chem. Res.* **32**, 1853–1921 (2023). doi:10.1007/s00044-023-03035-9 Medline

8. C. G. Wermuth, Are pyridazines privileged structures? *MedChemComm* **2**, 935–941 (2011). doi:10.1039/C1MD00074H

9. C. Lamberth, Pyridazine Chemistry in Crop Protection. *J. Heterocycl. Chem.* **54**, 2974–2984 (2017). doi:10.1002/jhet.2945

10. C. M. Marshall, J. G. Federice, C. N. Bell, P. B. Cox, J. T. Njardarson, An Update on the Nitrogen Heterocycle Compositions and Properties of U.S. FDA-Approved Pharmaceuticals (2013–2023). *J. Med. Chem.* **67**, 11622–11655 (2024). doi:10.1021/acs.jmedchem.4c01122 Medline

11. A. R. Dwivedi, S. Jaiswal, D. Kukkar, R. Kumar, T. G. Singh, M. P. Singh, A. M. Gaidhane, S. Lakhanpal, K. N. Prasad, B. Kumar, A decade of pyridine-containing heterocycles in US FDA approved drugs: A medicinal chemistry-based analysis. *RSC Med. Chem.* **16**, 12–36 (2024). doi:10.1039/D4MD00632A Medline

12. L. Horn, J. R. Infante, K. L. Reckamp, G. R. Blumenschein, T. A. Leal, S. N. Waqar, B. J. Gitlitz, R. E. Sanborn, J. G. Whisenant, L. Du, J. W. Neal, J. P. Gockerman, G. Dukart, K. Harrow, C. Liang, J. J. Gibbons, A. Holzhausen, C. M. Lovly, H. A. Wakelee, Ensartinib (X-396) in ALK-Positive Non–Small Cell Lung Cancer: Results from a First-in-Human Phase I/II, Multicenter Study. *Clin. Cancer Res.* **24**, 2771–2779 (2018). doi:10.1158/1078-0432.CCR-17-2398 Medline

13. S. T. Wrobleski, R. Moslin, S. Lin, Y. Zhang, S. Spergel, J. Kempson, J. S. Tokarski, J. Strnad, A. Zupa-Fernandez, L. Cheng, D. Shuster, K. Gillooly, X. Yang, E. Heimrich, K. W. McIntyre, C. Chaudhry, J. Khan, M. Ruzanov, J. Tredup, D. Mulligan, D. Xie, H. Sun, C. Huang, C. D'Arienzo, N. Aranibar, M. Chiney, A. Chimalakonda, W. J. Pitts, L. Lombardo, P. H. Carter, J. R. Burke, D. S. Weinstein, Highly Selective Inhibition of Tyrosine Kinase 2 (TYK2) for the Treatment of Autoimmune Diseases: Discovery of the Allosteric Inhibitor BMS-986165. *J. Med. Chem.* **62**, 8973–8995 (2019). doi:10.1021/acs.jmedchem.9b00444 Medline

14. X.-F. Wu, *Transition Metal-Catalyzed Pyridine Synthesis* (Elsevier, 2016), pp. i–iii.

15. P. Singh, Ed., *Recent Developments in the Synthesis and Applications of Pyridines* (Elsevier, 2022).

16. E. Scriven, Ed., *Pyridines: From Lab to Production* (Elsevier, 2013).

17. D. A. Evans, G. C. Andrews, Allylic sulfoxides. Useful intermediates in organic synthesis. *Acc. Chem. Res.* **7**, 147–155 (1974). doi:10.1021/ar50077a004

18. J. A. Joule, K. Mills, *Heterocyclic Chemistry* (Wiley, ed. 5, 2010).

19. J. Zhang, V. Shukla, D. L. Boger, Inverse Electron Demand Diels-Alder Reactions of Heterocyclic Azadienes, 1-Aza-1,3-Butadienes, Cyclopropenone Ketals, and Related Systems. A Retrospective. *J. Org. Chem.* **84**, 9397–9445 (2019). doi:10.1021/acs.joc.9b00834 Medline

20. J. Woo, A. H. Christian, S. A. Burgess, Y. Jiang, U. F. Mansoor, M. D. Levin, Scaffold hopping by net photochemical carbon deletion of azaarenes. *Science* **376**, 527–532 (2022). doi:10.1126/science.abo4282 Medline

21. J. Woo, C. Stein, A. H. Christian, M. D. Levin, Carbon-to-nitrogen single-atom transmutation of azaarenes. *Nature* **623**, 77–82 (2023). doi:10.1038/s41586-023-06613-4 Medline

22. H. Weber, T. Rohn, Synthese und Photoisomerisierung von sterisch gehinderten 2,6-Dialkylpyridin-N-oxiden / Synthesis and Photoisomerization of Sterically Hindered 2,6-Dialkylpyridine-N-oxides. *Z. Naturforsch. B* **45**, 701–706 (1990). doi:10.1515/znb-1990-0519

23. T. J. Pearson, R. Shimazumi, J. L. Driscoll, B. D. Dherange, D.-I. Park, M. D. Levin, Aromatic nitrogen scanning by *ipso*-selective nitrene internalization. *Science* **381**, 1474–1479 (2023). doi:10.1126/science.adj5331 Medline

24. A. Reisinger, R. Koch, P. V. Bernhardt, C. Wentrup, 1*H*-1,3-diazepines, 5*H*-1,3-diazepines, 1,3-diazepinones, and 2,4-diazabicyclo[3.2.0]heptenes. *Org. Biomol. Chem.* **2**, 1227–1238 (2004). doi:10.1039/B317099C Medline

25. T. Okamoto, M. Hirobe, Chemistry of Pyridine N-imines. *J. Synth. Org. Chem. Jpn.* **26**, 746–757 (1968). doi:10.5059/yukigoseikyokaishi.26.746

26. C. W. Rees, M. Yelland, Reactive intermediates. Part XVIII. An *N*-aminopyridone-to-pyridazine rearrangement; a new decarbonylation reaction. *J. Chem. Soc. Perkin Trans. 1* **1972**, 77–82 (1972). doi:10.1039/p19720000077

27. R. A. Abramovitch, C. Dupuy, Photolysis of 2-azidopyridine 1-oxides. A convenient synthesis of 1,2-oxazines. *J. Chem. Soc. Chem. Commun.* **1981**, 36–37 (1981). doi:10.1039/c39810000036

28. R. A. Abramovitch, B. W. Cue Jr., Ring contraction of 2-azidopyridine 1-oxides and related compounds. 2-Cyano-1-hydroxypyrroles and -imidazoles. *J. Am. Chem. Soc.* **98**, 1478–1486 (1976). doi:10.1021/ja00422a034

29. J. Schweng, E. Zbiral, Synthese von cyclischen Vinylaziden. *Justus Liebigs Ann. Chem.* **1978**, 1089–1095 (1978). doi:10.1002/jlac.197819780706

30. P. Cmoch, H. Korczak, L. Stefaniak, G. A. Webb, ^{1}H, ^{13}C and ^{15}N NMR and IR studies of halogen-substituted tetrazolo[1,5-*a*]pyridines. *J. Phys. Org. Chem.* **12**, 470–478 (1999). doi:10.1002/(SICI)1099-1395(199906)12:6<470:AID-POC151>3.0.CO;2-U

31. E. M. Arnett, R. Reich, Electronic effects on the Menshutkin reaction. A complete kinetic and thermodynamic dissection of alkyl transfer to 3- and 4-substituted pyridines. *J. Am. Chem. Soc.* **102**, 5892–5902 (1980). doi:10.1021/ja00538a031

32. T. Kiguchi, J. L. Schuppiser, J. C. Schwaller, J. Streith, Photochemical syntheses of 1,2-diazepines. 11. Regiospecific synthesis of 1,2-dihydro-1,2-diazepin-3-ones. *J. Org. Chem.* **45**, 5095–5100 (1980). doi:10.1021/jo01313a016

33. T. Eichenberger, H. Balli, Neue Heterocyclen: Über Tetrazinodi(heteroarene). Synthese und Struktur. *Helv. Chim. Acta* **69**, 1521–1530 (1986). doi:10.1002/hlca.19860690624

34. A. Fanourakis, Y. Ali, L. Chen, P. Q. Kelly, A. J. Bracken, C. B. Kelly, M. D. Levin, Strategic Atom Replacement Enables Regiocontrol in Pyrazole Alkylation. *Nature* **641**, 646–652 (2025). doi:10.1038/s41586-025-08951-x

35. T. B. Phan, H. Mayr, Nucleophilic reactivity of the azide ion in various solvents. *J. Phys. Org. Chem.* **19**, 706–713 (2006). doi:10.1002/poc.1063

36. D. S. Treitler, S. Leung, How Dangerous Is Too Dangerous? A Perspective on Azide Chemistry. *J. Org. Chem.* **87**, 11293–11295 (2022). doi:10.1021/acs.joc.2c01402 Medline

37. H. R. Memarian, M. Kalantari, Steric and electronic substitution effects on the thermal oxidation of 5-carboethoxy-2-oxo-1,2,3,4-tetrahydropyridines. *J. Iran. Chem. Soc.* **14**, 143–155 (2017). doi:10.1007/s13738-016-0966-z

38. Y. Yamamoto, K. Kinpara, T. Saigoku, H. Takagishi, S. Okuda, H. Nishiyama, K. Itoh, Cp*RuCl-catalyzed [2 + 2 + 2] cycloadditions of α,ω-diynes with electron-deficient carbon–heteroatom multiple bonds leading to heterocycles. *J. Am. Chem. Soc.* **127**, 605–613 (2005). doi:10.1021/ja045694g Medline

39. K. R. Roesch, H. Zhang, R. C. Larock, Synthesis of isoquinolines and pyridines by the palladium-catalyzed iminoannulation of internal alkynes. *J. Org. Chem.* **66**, 8042–8051 (2001). doi:10.1021/jo0105540 Medline

40. Z. Song, X. Huang, W. Yi, W. Zhang, One-Pot Reactions for Modular Synthesis of Polysubstituted and Fused Pyridines. *Org. Lett.* **18**, 5640–5643 (2016). doi:10.1021/acs.orglett.6b02883 Medline

41. S. Wang, Z. Geng, R. Guo, J. Li, D. Zou, Y. Wu, Y. Wu, Efficient synthesis of 5-substituted-3-pyridazine carbonitrile via regioselective Reissert-type reaction. *Tetrahedron Lett.* **57**, 3067–3070 (2016). doi:10.1016/j.tetlet.2016.05.037

42. F. Strieth-Kalthoff, F. Glorius, Triplet Energy Transfer Photocatalysis: Unlocking the Next Level. *Chem* **6**, 1888–1903 (2020). doi:10.1016/j.chempr.2020.07.010

43. M. Köckinger, C. A. Hone, C. O. Kappe, HCN on Tap: On-Demand Continuous Production of Anhydrous HCN for Organic Synthesis. *Org. Lett.* **21**, 5326–5330 (2019). doi:10.1021/acs.orglett.9b01941 Medline

44. D. L. Boger, R. S. Coleman, J. S. Panek, D. Yohannes, Thermal cycloaddition of dimethyl 1,2,4,5-tetrazine-3,6-dicarboxylate with electron-rich olefins: 1,2-diazine and pyrrole introduction. Preparation of octamethylporphin (OMP). *J. Org. Chem.* **49**, 4405–4409 (1984). doi:10.1021/jo00197a015

45. P. S. Fier, J. F. Hartwig, Selective C-H fluorination of pyridines and diazines inspired by a classic amination reaction. *Science* **342**, 956–960 (2013). doi:10.1126/science.1243759 Medline

46. F. O'Hara, D. G. Blackmond, P. S. Baran, Radical-based regioselective C–H functionalization of electron-deficient heteroarenes: Scope, tunability, and predictability. *J. Am. Chem. Soc.* **135**, 12122–12134 (2013). doi:10.1021/ja406223k Medline

47. J. D. Galloway, D. N. Mai, R. D. Baxter, Silver-Catalyzed Minisci Reactions Using Selectfluor as a Mild Oxidant. *Org. Lett.* **19**, 5772–5775 (2017). doi:10.1021/acs.orglett.7b02706 Medline

48. J. P. Jacobsen, O. Snerling, E. J. Pedersen, J. Tormod Nielsen, K. Schaumburg, Hydrogen NMR spectra of pyridazine and pyrazole containing ^{15}N. *J. Magn. Reson.* **1969**, 130–138 (1973).

49. G. L. Bartholomew, L. J. Karas, R. M. Eason, C. S. Yeung, M. S. Sigman, R. Sarpong, Cheminformatic Analysis of Core-Atom Transformations in Pharmaceutically Relevant Heteroaromatics. *J. Med. Chem.* **68**, 6027–6040 (2025). doi:10.1021/acs.jmedchem.4c02839 Medline

50. M. K. Bogdos, B. Morandi, EveRplot: A Web-Based Shiny Application for Creating Energy vs Reaction Coordinate Diagrams. *J. Chem. Educ.* **100**, 3641–3644 (2023). doi:10.1021/acs.jchemed.3c00319

51. O. V. Dolomanov, L. J. Bourhis, R. J. Gildea, J. K. Howard, H. Puschmann, *OLEX2*: A complete structure solution, refinement and analysis program. *J. Appl. Cryst.* **42**, 339–341 (2009). doi:10.1107/S0021889808042726

52. H. M. H. Nguyen, D. C. Thomas, M. A. Hart, K. R. Steenback, J. N. Levy, A. McNally, Synthesis of ^{15}N-Pyridines and Higher Mass Isotopologs via Zincke Imine Intermediates. *J. Am. Chem. Soc.* **146**, 2944–2949 (2024). doi:10.1021/jacs.3c12445 Medline

53. Y. Shen, Y. Gu, R. Martin, sp^3 C–H Arylation and Alkylation Enabled by the Synergy of Triplet Excited Ketones and Nickel Catalysts. *J. Am. Chem. Soc.* **140**, 12200–12209 (2018). doi:10.1021/jacs.8b07405 Medline

54. E. P. Farney, S. J. Chapman, W. B. Swords, M. D. Torelli, R. J. Hamers, T. P. Yoon, Discovery and Elucidation of Counteranion Dependence in Photoredox Catalysis. *J. Am. Chem. Soc.* **141**, 6385–6391 (2019). doi:10.1021/jacs.9b01885 Medline

55. K. Nottingham, C. Patel, J. Levy, A. Mcnally, X. Zhang, Phosphine Reagents for Azine Fluoroalkylation, US Patent US11780861B2 (2022).

56. T. T. Tung, T. H. Jakobsen, T. T. Dao, A. T. Fuglsang, M. Givskov, S. B. Christensen, J. Nielsen, Fusaric acid and analogues as Gram-negative bacterial quorum sensing inhibitors. *Eur. J. Med. Chem.* **126**, 1011–1020 (2017). doi:10.1016/j.ejmech.2016.11.044 Medline

57. A. R. Bena, E. G. Bakalbassis, M. M. Sigalas, I. N. Lykakis, One-Pot Synthetic Approach to 3-Carboxyl- and 3-Ketopyridines in Aqueous Media. *J. Org. Chem.* **88**, 8055–8068 (2023). doi:10.1021/acs.joc.3c00067 Medline

58. J. D. Vasta, R. T. Raines, Selective inhibition of prolyl 4-hydroxylases by bipyridinedicarboxylates. *Bioorg. Med. Chem.* **23**, 3081–3090 (2015). doi:10.1016/j.bmc.2015.05.003 Medline

59. M. J. Frisch, G. W. Trucks, H. B. Schlegel, G. E. Scuseria, M. A. Robb, J. R. Cheeseman, G. Scalmani, V. Barone, G. A. Petersson, H. Nakatsuji, X. Li, M. Caricato, A. V. Marenich, J. Bloino, B. G. Janesko, R. Gomperts, B. Mennucci, H. P. Hratchian, J. V. Ortiz, A. F. Izmaylov, J. L. Sonnenberg, D. Williams-Young, F. Ding, F. Lipparini, F. Egidi, J. Goings, B. Peng, A. Petrone, T. Henderson, D. Ranasinghe, V. G. Zakrzewski, J. Gao, N. Rega, G. Zheng, W. Liang, M. Hada, M. Ehara, K. Toyota, R. Fukuda, J. Hasegawa, M. Ishida, T. Nakajima, Y. Honda, O. Kitao, H. Nakai, T. Vreven, K. Throssell, J. A. Montgomery Jr., J. E. Peralta, F. Ogliaro, M. J. Bearpark, J. J. Heyd, E. N. Brothers, K. N. Kudin, V. N. Staroverov, T. A. Keith, R. Kobayashi, J. Normand, K. Raghavachari, A. P. Rendell, J. C. Burant, S. S. Iyengar, J. Tomasi, M. Cossi, J. M. Millam, M. Klene, C. Adamo, R. Cammi, J. W. Ochterski, R. L. Martin, K. Morokuma, O. Farkas, J. B. Foresman, D. J. Fox, Gaussian 16, Revision A.03 (2016).

60. J. Tomasi, B. Mennucci, R. Cammi, Quantum mechanical continuum solvation models. *Chem. Rev.* **105**, 2999–3093 (2005). doi:10.1021/cr9904009 Medline

61. S. Grimme, Supramolecular binding thermodynamics by dispersion-corrected density functional theory. *Chemistry* **18**, 9955–9964 (2012). doi:10.1002/chem.201200497 Medline

62. Y.-P. Li, J. Gomes, S. Mallikarjun Sharada, A. T. Bell, M. Head-Gordon, Improved Force-Field Parameters for QM/MM Simulations of the Energies of Adsorption for Molecules in Zeolites and a Free Rotor Correction to the Rigid Rotor Harmonic Oscillator Model for Adsorption Enthalpies. *J. Phys. Chem. C* **119**, 1840–1850 (2015). doi:10.1021/jp509921r

63. G. Luchini, J. V. Alegre-Requena, I. Funes-Ardoiz, R. S. Paton, GoodVibes: Automated thermochemistry for heterogeneous computational chemistry data. *F1000 Res.* **9**, 291 (2020). doi:10.12688/f1000research.22758.1
